# Supplementary material for: The disparities and development trajectories of nations in achieving the sustainable development goals
Source: Nat Commun. 2025 Jan 28;16:1107. doi: 10.1038/s41467-025-56076-6 (PMC11775216; doi:10.1038/s41467-025-56076-6)
Supplement: Supplementary file 1 — Supplementary Information [file 41467_2025_56076_MOESM1_ESM.pdf]

Supplementary Information for
The Disparities and Development Trajectories of Nations in Achieving the Sustainable Development Goals

Fengmei Ma[1,2], Heming Wang[2,3*], Asaf Tzachor[4,5*], César A. Hidalgo[6,7], Heinz Schandl[2,8], Yue Zhang[3], Jingling Zhang[9], Wei-Qiang Chen[1,10]*, Yanzhi Zhao[11], Yong-Guan Zhu[1,10,12], Bojie Fu[12]

[1] Key Lab of Urban Environment and Health, Institute of Urban Environment, Chinese Academy of Sciences, Xiamen, 335400, China

[2] Commonwealth Scientific and Industrial Research Organisation (CSIRO), Canberra, ACT 2601, Australia

[3] State Environmental Protection Key Laboratory of Eco-Industry, Northeastern University, Shenyang, 110819, China

[4] School of Sustainability, Reichman University (IDC Herzliya), Herzliya, 4610101, Israel.

[5] Centre for the Study of Existential Risk (CSER), University of Cambridge, Cambridge, CB2 1SB, United Kingdom

[6] Toulouse School of Economics, University of Toulouse Capitole, Toulouse, 31000, France

[7] Center for Collective Learning, ANITI, IRIT, University of Toulouse, 21 All. de Brienne, 31000 Toulouse, France & CIAS, Corvinus University of Budapest, Budapest, Közraktár u. 4-6, 1093, Hungary

[8] Graduate School of Environmental Studies, Nagoya University, Nagoya, Japan

[9] LEREPS - Laboratoire d'Etude et de Recherche sur l'Economie, les Politiques et les Systèmes Sociaux; Institut d'Études Politiques [IEP] - Toulouse, 31000, France

[10] University of Chinese Academy of Sciences, Beijing, 101408, China

[11] Institute of Carbon Neutrality Technology and Policy, Shenyang University, Shenyang 110044, China.

[12] State Key Laboratory of Urban and Regional Ecology, Research Center for Eco-Environmental Sciences, Chinese Academy of Sciences, Beijing, China

* Corresponding authors: Heming Wang (email: wanghm@mail.neu.edu.cn), Asaf Tzachor (email: atzachor@runi.ac.il), Wei-Qiang Chen (email: wqchen@iue.ac.cn)

Content

1. Supplementary Tables 1-22
2. Supplementary Figures 1-177

Supplementary Tables

Supplementary Table 1 | Country list with values of SDG index score and country sustainability index (CSI) in 4 sustainability stages.......... 9

Supplementary Table 2 | The RCA and SDG scores of SDG indicators in the 1st window group of countries in 2000.......... 13

Supplementary Table 3 | The RCA and SDG scores of SDG indicators in the 40th window group of countries in 2000.......... 18

Supplementary Table 4 | The RCA and SDG scores of SDG indicators in the 80th window group of countries in 2000.......... 22

Supplementary Table 5 | The RCA and SDG scores of SDG indicators in the 117th window group of countries in 2000.......... 26

Supplementary Table 6 | The RCA and SDG scores of SDG indicators in the 1st window group of countries in 2015.......... 30

Supplementary Table 7 | The RCA and SDG scores of SDG indicators in the 40th window group of countries in 2015.......... 34

Supplementary Table 8 | The RCA and SDG scores of SDG indicators in the 80th window group of countries in 2015.......... 38

Supplementary Table 9 | The RCA and SDG scores of SDG indicators in the 117th window group of countries in 2015.......... 41

Supplementary Table 10 | The RCA and SDG scores of SDG indicators in the 1st window group of countries in 2022.......... 45

Supplementary Table 11 | The RCA and SDG scores of SDG indicators in the 40th window group of countries in 2022.......... 49

Supplementary Table 12 | The RCA and SDG scores of SDG indicators in the 80th window group of countries in 2022.......... 53

Supplementary Table 13 | The RCA and SDG scores of SDG indicators in the 117th window group of countries in 2022.......... 57

Supplementary Table 14 | The changes in RCA of SDG indicators in each sustainability stage in 2000.......... 61

Supplementary Table 15 | The changes in RCA of SDG indicators for each sustainability stage in 2015.......... 64

Supplementary Table 16 | The changes in RCA of SDG indicators for each sustainability stage in 2022.......... 67

Supplementary Table 17 | The top 10 SDG indicators with the most increasement in RCA for each sustainability stage in 2000.......... 70

Supplementary Table 18 | The top 10 SDG indicators with the most decrease in RCA for each sustainability stage in 2000.......... 71

Supplementary Table 19 | The top 10 SDG indicators with the most increasement in RCA for each sustainability stage in 2015. 72
Supplementary Table 20 | The top 10 SDG indicators with the most decrease in RCA for each sustainability stage in 2015. 73
Supplementary Table 21 | The top 10 SDG indicators with the most increasement in RCA for each sustainability stage in 2022. 74
Supplementary Table 22 | The top 10 SDG indicators with the most decrease in RCA for each sustainability stage in 2022. 75

Supplementary Figures

Supplementary Figure 1 | The SDG space with 17 SDG labels.. 76

Supplementary Figure 2 | The SDG space by communities in 2022 77

Supplementary Figure 3 | The evolution of SDG space.. 78

Supplementary Figure 4 | The trends of the trade-off similarity between SDG indicators.. 79

Supplementary Figure 5 | The evolution of country space and similarity of countries. 80

Supplementary Figure 6 | The evolution of SDG indicators along with the ranking of countries' SDG scores (a) and GDP per capita (b) in 2022 with moving-window size = 1. 81

Supplementary Figure 7 | The evolution of SDG indicators alongside the ranking of countries' SDG scores in 2022 with varying moving-window sizes.. 82

Supplementary Figure 8 | The relationship between the country sustainability index (CSI) and the SDG index of countries in 2022. 83

Supplementary Figure 9 | Sensitivity analysis of SDG indicators' similarity in 2022. 84

Supplementary Figure 10 | The SDG space in 2022 with non-overlapping indicators.. 85

Supplementary Figure 11 | The SDG space in 2022 using UN SDG data.. 86

Supplementary Figure 12 | The SDG space of Luxembourg... 87

Supplementary Figure 13 | The SDG space of Norway... 88

Supplementary Figure 14 | The SDG space of Ireland 89

Supplementary Figure 15 | The SDG space of Switzerland. 90

Supplementary Figure 16 | The SDG space of Qatar 91

Supplementary Figure 17 | The SDG space of Singapore. 92

Supplementary Figure 18 | The SDG space of United States. 93

Supplementary Figure 19 | The SDG space of Iceland. 94

Supplementary Figure 20 | The SDG space of Denmark. 95

Supplementary Figure 21 | The SDG space of Australia. 96

Supplementary Figure 22 | The SDG space of Netherlands 97

Supplementary Figure 23 | The SDG space of Sweden. 98

Supplementary Figure 24 | The SDG space of Israel. 99

Supplementary Figure 25 | The SDG space of Canada. 100

Supplementary Figure 26 | The SDG space of United Arab Emirates. 101

Supplementary Figure 27 | The SDG space of Austria. 102

Supplementary Figure 28 | The SDG space of Finland 103

Supplementary Figure 29 | The SDG space of Belgium. 104

Supplementary Figure 30 | The SDG space of Germany. 105

Supplementary Figure 31 | The SDG space of New Zealand 106

Supplementary Figure 32 | The SDG space of United Kingdom. 107

Supplementary Figure 33 | The SDG space of Kuwait. 108
Supplementary Figure 34 | The SDG space of France. 109
Supplementary Figure 35 | The SDG space of Italy 110
Supplementary Figure 36 | The SDG space of Malta 111
Supplementary Figure 37 | The SDG space of Japan. 112
Supplementary Figure 38 | The SDG space of Korea, Rep. 113
Supplementary Figure 39 | The SDG space of Cyprus. 114
Supplementary Figure 40 | The SDG space of Bahamas. 115
Supplementary Figure 41 | The SDG space of Saudi Arabia. 116
Supplementary Figure 42 | The SDG space of Bahrain. 117
Supplementary Figure 43 | The SDG space of Spain. 118
Supplementary Figure 44 | The SDG space of Slovenia. 119
Supplementary Figure 45 | The SDG space of Estonia. 120
Supplementary Figure 46 | The SDG space of Czechia. 121
Supplementary Figure 47 | The SDG space of Lithuania 122
Supplementary Figure 48 | The SDG space of Oman. 123
Supplementary Figure 49 | The SDG space of Portugal. 124
Supplementary Figure 50 | The SDG space of Latvia 125
Supplementary Figure 51 | The SDG space of Slovak Republic. 126
Supplementary Figure 52 | The SDG space of Greece 127
Supplementary Figure 53 | The SDG space of Uruguay. 128
Supplementary Figure 54 | The SDG space of Barbados. 129
Supplementary Figure 55 | The SDG space of Poland. 130
Supplementary Figure 56 | The SDG space of Croatia. 131
Supplementary Figure 57 | The SDG space of Hungary. 132
Supplementary Figure 58 | The SDG space of Guyana. 133
Supplementary Figure 59 | The SDG space of Panama. 134
Supplementary Figure 60 | The SDG space of Romania. 135
Supplementary Figure 61 | The SDG space of Chile. 136
Supplementary Figure 62 | The SDG space of Russian Federation. 137
Supplementary Figure 63 | The SDG space of Bulgaria. 138
Supplementary Figure 64 | The SDG space of Argentina. 139
Supplementary Figure 65 | The SDG space of Costa Rica. 140
Supplementary Figure 66 | The SDG space of China. 141
Supplementary Figure 67 | The SDG space of Malaysia. 142
Supplementary Figure 68 | The SDG space of Maldives. 143
Supplementary Figure 69 | The SDG space of Mexico 144
Supplementary Figure 70 | The SDG space of Kazakhstan. 145

Supplementary Figure 71 | The SDG space of Turkey. 146
Supplementary Figure 72 | The SDG space of Mauritius. 147
Supplementary Figure 73 | The SDG space of Dominican Republic. 148
Supplementary Figure 74 | The SDG space of Montenegro. 149
Supplementary Figure 75 | The SDG space of Serbia. 150
Supplementary Figure 76 | The SDG space of Cuba. 151
Supplementary Figure 77 | The SDG space of Brazil. 152
Supplementary Figure 78 | The SDG space of Gabon. 153
Supplementary Figure 79 | The SDG space of Turkmenistan. 154
Supplementary Figure 80 | The SDG space of Belarus. 155
Supplementary Figure 81 | The SDG space of Azerbaijan. 156
Supplementary Figure 82 | The SDG space of Botswana. 157
Supplementary Figure 83 | The SDG space of Bosnia and Herzegovina. 158
Supplementary Figure 84 | The SDG space of Peru. 159
Supplementary Figure 85 | The SDG space of Armenia. 160
Supplementary Figure 86 | The SDG space of Belize. 161
Supplementary Figure 87 | The SDG space of Thailand. 162
Supplementary Figure 88 | The SDG space of Albania. 163
Supplementary Figure 89 | The SDG space of South Africa. 164
Supplementary Figure 90 | The SDG space of Colombia. 165
Supplementary Figure 91 | The SDG space of North Macedonia. 166
Supplementary Figure 92 | The SDG space of Ecuador. 167
Supplementary Figure 93 | The SDG space of Paraguay. 168
Supplementary Figure 94 | The SDG space of Jamaica. 169
Supplementary Figure 95 | The SDG space of Iraq. 170
Supplementary Figure 96 | The SDG space of Suriname. 171
Supplementary Figure 97 | The SDG space of Moldova. 172
Supplementary Figure 98 | The SDG space of Lebanon. 173
Supplementary Figure 99 | The SDG space of Guatemala. 174
Supplementary Figure 100 | The SDG space of Fiji. 175
Supplementary Figure 101 | The SDG space of El Salvador. 176
Supplementary Figure 102 | The SDG space of Mongolia. 177
Supplementary Figure 103 | The SDG space of Namibia. 178
Supplementary Figure 104 | The SDG space of Indonesia. 179
Supplementary Figure 105 | The SDG space of Iran. 180
Supplementary Figure 106 | The SDG space of Ukraine. 181
Supplementary Figure 107 | The SDG space of Algeria. 182
Supplementary Figure 108 | The SDG space of Jordan. 183

Supplementary Figure 109 | The SDG space of Egypt, Arab Rep.. 184
Supplementary Figure 110 | The SDG space of Vietnam. 185
Supplementary Figure 111 | The SDG space of Eswatini. 186
Supplementary Figure 112 | The SDG space of Cabo Verde. 187
Supplementary Figure 113 | The SDG space of Tunisia. 188
Supplementary Figure 114 | The SDG space of Bolivia. 189
Supplementary Figure 115 | The SDG space of Philippines. 190
Supplementary Figure 116 | The SDG space of Morocco. 191
Supplementary Figure 117 | The SDG space of Sri Lanka. 192
Supplementary Figure 118 | The SDG space of Bhutan. 193
Supplementary Figure 119 | The SDG space of Djibouti 194
Supplementary Figure 120 | The SDG space of Papua New Guinea. 195
Supplementary Figure 121 | The SDG space of Honduras. 196
Supplementary Figure 122 | The SDG space of Angola. 197
Supplementary Figure 123 | The SDG space of Bangladesh. 198
Supplementary Figure 124 | The SDG space of Congo, Rep.. 199
Supplementary Figure 125 | The SDG space of Cote d'Ivoire. 200
Supplementary Figure 126 | The SDG space of India. 201
Supplementary Figure 127 | The SDG space of Nicaragua. 202
Supplementary Figure 128 | The SDG space of Uzbekistan. 203
Supplementary Figure 129 | The SDG space of Ghana. 204
Supplementary Figure 130 | The SDG space of Nigeria. 205
Supplementary Figure 131 | The SDG space of Kenya. 206
Supplementary Figure 132 | The SDG space of Mauritania. 207
Supplementary Figure 133 | The SDG space of Lao PDR. 208
Supplementary Figure 134 | The SDG space of Cambodia. 209
Supplementary Figure 135 | The SDG space of Haiti. 210
Supplementary Figure 136 | The SDG space of Zimbabwe. 211
Supplementary Figure 137 | The SDG space of Kyrgyz Republic 212
Supplementary Figure 138 | The SDG space of Senegal. 213
Supplementary Figure 139 | The SDG space of Pakistan. 214
Supplementary Figure 140 | The SDG space of Cameroon. 215
Supplementary Figure 141 | The SDG space of Guinea. 216
Supplementary Figure 142 | The SDG space of Comoros. 217
Supplementary Figure 143 | The SDG space of Zambia. 218
Supplementary Figure 144 | The SDG space of Nepal. 219
Supplementary Figure 145 | The SDG space of Benin. 220
Supplementary Figure 146 | The SDG space of Tanzania. 221

Supplementary Figure 147 | The SDG space of Myanmar. 222
Supplementary Figure 148 | The SDG space of Sudan.. 223
Supplementary Figure 149 | The SDG space of Tajikistan.. 224
Supplementary Figure 150 | The SDG space of Ethiopia. 225
Supplementary Figure 151 | The SDG space of Lesotho... 226
Supplementary Figure 152 | The SDG space of Rwanda... 227
Supplementary Figure 153 | The SDG space of Uganda. 228
Supplementary Figure 154 | The SDG space of Togo. 229
Supplementary Figure 155 | The SDG space of Mali. 230
Supplementary Figure 155 | The SDG space of Burkina Faso. 231
Supplementary Figure 157 | The SDG space of Gambia. 232
Supplementary Figure 158 | The SDG space of Guinea. 233
Supplementary Figure 159 | The SDG space of Liberia. 234
Supplementary Figure 160 | The SDG space of Chad. 235
Supplementary Figure 161 | The SDG space of Congo, Dem. Rep.. 236
Supplementary Figure 162 | The SDG space of Yemen, Rep.. 237
Supplementary Figure 163 | The SDG space of Malawi... 238
Supplementary Figure 164 | The SDG space of Somalia... 239
Supplementary Figure 165 | The SDG space of Niger... 240
Supplementary Figure 166 | The SDG space of Mozambique... 241
Supplementary Figure 167 | The SDG space of Syrian Arab Republic..................................... 242
Supplementary Figure 168 | The SDG space of Madagascar... 243
Supplementary Figure 169 | The SDG space of Afghanistan. 244
Supplementary Figure 170 | The SDG space of Sierra Leone. 245
Supplementary Figure 171 | The SDG space of Central African Republic................................. 246
Supplementary Figure 172 | The SDG space of Burundi... 247
Supplementary Figure 173 | The SDG space of South Sudan.. 248
Supplementary Figure 174 | The SDG space of Venezuela, RB... 249
Supplementary Figure 174 | The SDG space of Brunei Darussalam. 250
Supplementary Figure 175 | The SDG space of Georgia... 251
Supplementary Figure 176 | The SDG space of Sao Tome and Principe................................... 252
Supplementary Figure 177 | The SDG space of Trinidad and Tobago. 253

Supplementary Tables

Supplementary Table 1 | Country list with values of SDG score and country sustainability index (CSI) in 4 sustainability stages.

Supplementary Table 1 | Country list with values of SDG score and country sustainability index (CSI) in 4 sustainability stages. Note: Countries are grouped evenly into 4 stages based on their SDG scores in 2022.

SDG rank	Country code	Country name	SDG score in 2022	Sustainability stage in 2022	CSI in 2022
**1**	FIN	Finland	86.8	4	1.5
**2**	SWE	Sweden	86	4	1.7
**3**	DNK	Denmark	85.7	4	1.5
**4**	DEU	Germany	83.4	4	1.6
**5**	AUT	Austria	82.3	4	1.5
**6**	FRA	France	82	4	1.6
**7**	NOR	Norway	82	4	1.5
**8**	CZE	Czechia	81.9	4	1.5
**9**	POL	Poland	81.8	4	1.7
**10**	EST	Estonia	81.7	4	1.6
**11**	GBR	United Kingdom	81.7	4	1.5
**12**	HRV	Croatia	81.5	4	1.3
**13**	SVN	Slovenia	81	4	1.7
**14**	LVA	Latvia	80.7	4	1.4
**15**	CHE	Switzerland	80.5	4	1.1
**16**	ESP	Spain	80.4	4	1.6
**17**	IRL	Ireland	80.1	4	1.6
**18**	PRT	Portugal	80	4	1.6
**19**	BEL	Belgium	79.5	4	1.5
**20**	HUN	Hungary	79.4	4	1.1
**21**	JPN	Japan	79.4	4	1.5
**22**	NLD	Netherlands	79.4	4	1.6
**23**	SVK	Slovak Republic	79.1	4	1.4
**24**	ITA	Italy	78.8	4	1.7
**25**	MDA	Moldova	78.6	4	-0.2
**26**	CAN	Canada	78.5	4	1.4
**27**	GRC	Greece	78.4	4	1.5
**28**	NZL	New Zealand	78.4	4	1.4
**29**	ISL	Iceland	78.3	4	1.0
**30**	CHL	Chile	78.2	4	1.5
**31**	KOR	Korea, Rep.	78.1	4	1.5
**32**	URY	Uruguay	77.7	4	1.1
**33**	LUX	Luxembourg	77.6	4	1.2
**34**	BLR	Belarus	77.5	4	0.5
**35**	ROU	Romania	77.5	4	1.1
**36**	SRB	Serbia	77.3	4	0.7
**37**	LTU	Lithuania	76.8	4	1.1
**38**	UKR	Ukraine	76.5	4	0.2
**39**	AUS	Australia	75.9	4	1.5

**SDG rank**	**Country code**	**Country name**	**SDG score in 2022**	**Sustainability stage in 2022**	**CSI in 2022**
**40**	USA	United States	75.9	4	1.7
**41**	MLT	Malta	75.5	4	1.0
**42**	GEO	Georgia	75	3	0.2
**43**	THA	Thailand	74.7	3	0.7
**44**	BGR	Bulgaria	74.6	3	0.5
**45**	KGZ	Kyrgyz Republic	74.4	3	-0.6
**46**	CUB	Cuba	74.1	3	-0.4
**47**	BIH	Bosnia and Herzegovina	74	3	0.1
**48**	ISR	Israel	74	3	1.1
**49**	RUS	Russian Federation	73.8	3	0.4
**50**	ARG	Argentina	73.7	3	0.4
**51**	BRA	Brazil	73.7	3	0.6
**52**	CRI	Costa Rica	73.6	3	1.0
**53**	ALB	Albania	73.5	3	-0.2
**54**	AZE	Azerbaijan	73.5	3	-0.8
**55**	ARM	Armenia	73.3	3	-0.4
**56**	VNM	Vietnam	73.3	3	-0.2
**57**	FJI	Fiji	72.9	3	0.2
**58**	CYP	Cyprus	72.5	3	1.4
**59**	MKD	North Macedonia	72.5	3	-0.4
**60**	TUN	Tunisia	72.5	3	0.2
**61**	BTN	Bhutan	72.3	3	-0.7
**62**	DOM	Dominican Republic	72.1	3	-0.4
**63**	CHN	China	72	3	-0.1
**64**	SGP	Singapore	71.8	3	1.0
**65**	PER	Peru	71.7	3	0.0
**66**	KAZ	Kazakhstan	71.6	3	0.0
**67**	MNE	Montenegro	71.4	3	0.7
**68**	MDV	Maldives	71.3	3	0.2
**69**	UZB	Uzbekistan	71.1	3	-0.3
**70**	MAR	Morocco	70.9	3	-0.3
**71**	DZA	Algeria	70.8	3	-0.4
**72**	TUR	Türkiye	70.8	3	0.6
**73**	SLV	El Salvador	70.7	3	-0.1
**74**	ECU	Ecuador	70.4	3	-0.2
**75**	IDN	Indonesia	70.2	3	-0.4
**76**	COL	Colombia	70.1	3	0.0
**77**	JOR	Jordan	69.9	3	-0.2
**78**	MYS	Malaysia	69.8	3	0.4
**79**	MEX	Mexico	69.7	3	0.4
**80**	ARE	United Arab Emirates	69.7	3	1.1
**81**	EGY	Egypt, Arab Rep.	69.6	3	-0.3
**82**	JAM	Jamaica	69.6	3	-0.1
**83**	BRB	Barbados	69.4	2	0.5
**84**	LKA	Sri Lanka	69.4	2	-0.5
**85**	TJK	Tajikistan	69.2	2	-0.6
**86**	IRN	Iran, Islamic Rep.	69.1	2	0.0

SDG rank	Country code	Country name	SDG score in 2022	Sustainability stage in 2022	CSI in 2022
**87**	BOL	Bolivia	68.9	2	-0.9
**88**	CPV	Cabo Verde	68.8	2	0.1
**89**	PRY	Paraguay	68.8	2	-0.4
**90**	OMN	Oman	68.6	2	0.3
**91**	TKM	Turkmenistan	68.5	2	-0.4
**92**	SUR	Suriname	68.2	2	0.1
**93**	MUS	Mauritius	68	2	0.4
**94**	SAU	Saudi Arabia	67.7	2	0.7
**95**	LBN	Lebanon	67.5	2	-0.2
**96**	GUY	Guyana	67.4	2	0.1
**97**	PAN	Panama	67.3	2	0.3
**98**	PHL	Philippines	67.1	2	-0.7
**99**	NPL	Nepal	66.5	2	-0.8
**100**	QAT	Qatar	66.2	2	0.8
**101**	BGD	Bangladesh	65.9	2	-1.0
**102**	BRN	Brunei Darussalam	65.7	2	0.2
**103**	KHM	Cambodia	64.8	2	-1.1
**104**	IRQ	Iraq	64.8	2	-0.8
**105**	NIC	Nicaragua	64.8	2	-0.8
**106**	MNG	Mongolia	64.7	2	-0.2
**107**	BLZ	Belize	64.6	2	-0.5
**108**	KWT	Kuwait	64.4	2	0.7
**109**	NAM	Namibia	64.3	2	-0.7
**110**	ZAF	South Africa	64	2	0.4
**111**	BHR	Bahrain	63.7	2	0.4
**112**	IND	India	63.4	2	-0.7
**113**	GAB	Gabon	63.1	2	-0.7
**114**	LAO	Lao PDR	63	2	-1.2
**115**	TTO	Trinidad and Tobago	63	2	0.6
**116**	HND	Honduras	62.9	2	-0.9
**117**	VEN	Venezuela, RB	62.9	2	-0.3
**118**	BWA	Botswana	62.7	2	-0.6
**119**	STP	Sao Tome and Principe	62.7	2	-0.3
**120**	CIV	Cote d'Ivoire	62.3	2	-1.3
**121**	GHA	Ghana	61.8	2	-0.9
**122**	SEN	Senegal	61.8	2	-1.1
**123**	BHS	Bahamas, The	60.9	2	0.1
**124**	KEN	Kenya	60.9	2	-1.0
**125**	MMR	Myanmar	60.4	1	-1.0
**126**	RWA	Rwanda	60.2	1	-0.9
**127**	GTM	Guatemala	59.4	1	-1.0
**128**	PAK	Pakistan	59	1	-0.8
**129**	GMB	Gambia, The	58.3	1	-1.3
**130**	SYR	Syrian Arab Republic	58.2	1	-0.5
**131**	MLI	Mali	58	1	-1.3
**132**	SWZ	Eswatini	57.9	1	0.1
**133**	MRT	Mauritania	57.2	1	-1.0

SDG rank	Country code	Country name	SDG score in 2022	Sustainability stage in 2022	CSI in 2022
**134**	TZA	Tanzania	56.8	1	-1.1
**135**	MWI	Malawi	56.3	1	-1.2
**136**	TGO	Togo	56.3	1	-1.4
**137**	SLE	Sierra Leone	55.7	1	-1.3
**138**	ZWE	Zimbabwe	55.6	1	-1.1
**139**	BEN	Benin	55.1	1	-1.2
**140**	CMR	Cameroon	55.1	1	-1.4
**141**	UGA	Uganda	55	1	-1.1
**142**	GIN	Guinea	54.9	1	-1.3
**143**	LSO	Lesotho	54.9	1	-0.3
**144**	ETH	Ethiopia	54.5	1	-1.0
**145**	NGA	Nigeria	54.3	1	-1.1
**146**	ZMB	Zambia	54.3	1	-1.3
**147**	BDI	Burundi	53.9	1	-1.4
**148**	PNG	Papua New Guinea	53.6	1	-1.2
**149**	DJI	Djibouti	52.7	1	-0.9
**150**	MOZ	Mozambique	52.7	1	-1.3
**151**	COG	Congo, Rep.	52.6	1	-1.2
**152**	HTI	Haiti	52.6	1	-1.2
**153**	BFA	Burkina Faso	52.4	1	-1.2
**154**	COM	Comoros	51.7	1	-0.6
**155**	AGO	Angola	50.8	1	-1.4
**156**	MDG	Madagascar	50.3	1	-1.4
**157**	LBR	Liberia	49.9	1	-0.9
**158**	AFG	Afghanistan	49	1	-1.2
**159**	COD	Congo, Dem. Rep.	48.6	1	-1.5
**160**	SDN	Sudan	48.6	1	-0.7
**161**	NER	Niger	48.3	1	-1.1
**162**	SOM	Somalia	48	1	-1.2
**163**	YEM	Yemen, Rep.	46.8	1	-1.0
**164**	TCD	Chad	45.3	1	-1.4
**165**	CAF	Central African Republic	40.4	1	-1.5
**166**	SSD	South Sudan	38.7	1	-1.3

Supplementary Table 2 | The RCA and SDG scores of SDG indicators in the 1st window group of countries in 2000. Note: The SDG indicators listed by the rank order of revealed comparative advantage (RCA). The RCA results are generated by the moving-window approach with window size of 50 (see Methods).

Year	SDG indicators	Indicator name	Window group	Rank of RCA in this group	Average RCA in this group	Average SDG score in this group	Rank of SDG score in this group	Rank difference (score-RCA) for window group
**2000**	9.A	Articles published in academic journals (per 1,000 population)	1	1	1.17	51.7	79	-78
**2000**	9.B	Population using the internet (%)	1	2	1.10	18.4	95	-93
**2000**	6.D	Anthropogenic wastewater that receives treatment (%)	1	3	0.94	53.0	78	-75
**2000**	9.E	Expenditure on research and development (% of GDP)	1	4	0.91	34.2	90	-86
**2000**	9.D	Mobile broadband subscriptions (per 100 population)	1	5	0.86	17.8	96	-91
**2000**	3.M	Universal health coverage (UHC) index of service coverage (worst 0-100 best)	1	6	0.74	45.0	85	-79
**2000**	16.E	Expropriations are lawful and adequately compensated (worst 0 - 1 best)	1	7	0.60	58.2	72	-65
**2000**	9.G	The Times Higher Education Universities Ranking: Average score of top 3 universities (worst 0-100 best)	1	8	0.60	49.5	82	-74
**2000**	14.C	Mean area that is protected in marine sites important to biodiversity (%)	1	9	0.56	37.3	88	-79
**2000**	8.A	Adults with an account at a bank or other financial institution or with a mobile-money-service provider (% of population aged 15 or over)	1	10	0.54	76.2	46	-36
**2000**	16.B	Children involved in child labor (% of population aged 5 to 14)	1	11	0.51	97.4	10	1
**2000**	1.A	Poverty headcount ratio at $3.65/day (2017 PPP, %)	1	12	0.45	96.3	16	-4
**2000**	8.E	Fundamental labor rights are effectively guaranteed (worst 0–1 best)	1	13	0.44	75.4	50	-37
**2000**	10.B	Palma ratio	1	14	0.41	70.4	55	-41
**2000**	4.A	Participation rate in pre-primary organized learning (% of children aged 4 to 6)	1	15	0.40	88.2	30	-15
**2000**	14.B	Ocean Health Index: Clean Waters score (worst 0-100 best)	1	16	0.40	56.1	74	-58
**2000**	11.C	Proportion of urban population living in slums (%)	1	17	0.39	96.7	13	4

Year	SDG indicators	Indicator name	Window group	Rank of RCA in this group	Average RCA in this group	Average SDG score in this group	Rank of SDG score in this group	Rank difference (score-RCA) for window group
**2000**	16.A	Timeliness of administrative proceedings (worst 0 - 1 best)	1	18	0.35	67.2	61	-43
**2000**	3.G	Neonatal mortality rate (per 1,000 live births)	1	19	0.34	87.3	33	-14
**2000**	7.A	Population with access to clean fuels and technology for cooking (%)	1	20	0.32	93.2	22	-2
**2000**	2.E	Prevalence of stunting in children under 5 years of age (%)	1	21	0.31	88.8	28	-7
**2000**	4.B	Literacy rate (% of population aged 15 to 24)	1	22	0.29	98.4	5	17
**2000**	16.C	Corruption Perceptions Index (worst 0-100 best)	1	23	0.29	64.0	66	-43
**2000**	4.D	Lower secondary completion rate (%)	1	24	0.29	89.7	26	-2
**2000**	3.D	Life expectancy at birth (years)	1	25	0.27	74.2	52	-27
**2000**	9.C	Logistics Performance Index: Quality of trade and transport-related infrastructure (worst 1-5 best)	1	26	0.27	70.4	56	-30
**2000**	3.C	New HIV infections (per 1,000 uninfected population)	1	27	0.27	97.6	8	19
**2000**	17.C	Statistical Performance Index (worst 0-100 best)	1	28	0.25	70.7	54	-26
**2000**	2.A	Cereal yield (tonnes per hectare of harvested land)	1	29	0.25	55.5	75	-46
**2000**	3.L	Mortality rate, under-5 (per 1,000 live births)	1	30	0.24	93.5	21	9
**2000**	16.G	Access to and affordability of justice (worst 0–1 best)	1	31	0.23	79.7	41	-10
**2000**	6.B	Population using at least basic sanitation services (%)	1	32	0.23	94.2	20	12
**2000**	3.A	Births attended by skilled health personnel (%)	1	33	0.20	99.0	3	30
**2000**	3.I	Subjective well-being (average ladder score, worst 0-10 best)	1	34	0.19	67.6	60	-26
**2000**	14.E	Fish caught from overexploited or collapsed stocks (% of total catch)	1	35	0.18	68.4	59	-24
**2000**	7.C	Population with access to electricity (%)	1	36	0.17	99.2	2	34
**2000**	2.G	Prevalence of undernourishment (%)	1	37	0.16	96.3	17	20
**2000**	5.D	Seats held by women in national parliament (%)	1	38	0.16	32.6	91	-53

Year	SDG indicators	Indicator name	Window group	Rank of RCA in this group	Average RCA in this group	Average SDG score in this group	Rank of SDG score in this group	Rank difference (score-RCA) for window group
**2000**	6.E	Population using at least basic drinking water services (%)	1	39	0.15	96.4	14	25
**2000**	3.F	Age-standardized death rate due to cardiovascular disease, cancer, diabetes, or chronic respiratory disease in adults aged 30–70 years (%)	1	40	0.15	53.1	77	-37
**2000**	16.D	Unsentenced detainees (% of prison population)	1	41	0.15	70.2	57	-16
**2000**	3.B	Adolescent fertility rate (births per 1,000 females aged 15 to 19)	1	42	0.15	84.2	36	6
**2000**	17.B	Government spending on health and education (% of GDP)	1	43	0.14	62.3	68	-25
**2000**	5.B	Demand for family planning satisfied by modern methods (% of females aged 15 to 49)	1	44	0.13	65.2	64	-20
**2000**	9.F	Rural population with access to all-season roads (%)	1	45	0.12	97.5	9	36
**2000**	7.D	Renewable energy share in total final energy consumption (%)	1	46	0.11	26.2	93	-47
**2000**	10.A	Gini coefficient	1	47	0.11	79.6	42	5
**2000**	4.C	Net primary enrollment rate (%)	1	48	0.11	96.4	15	33
**2000**	16.H	Press Freedom Index (worst 0-100 best)	1	49	0.11	80.7	39	10
**2000**	2.H	Prevalence of wasting in children under 5 years of age (%)	1	50	0.10	88.5	29	21
**2000**	3.E	Maternal mortality rate (per 100,000 live births)	1	51	0.10	98.0	6	45
**2000**	2.C	Exports of hazardous pesticides (tonnes per million population)	1	52	0.10	88.2	32	20
**2000**	5.A	Ratio of female-to-male mean years of education received (%)	1	53	0.10	89.4	27	26
**2000**	1.B	Poverty headcount ratio at $2.15/day (2017 PPP, %)	1	54	0.09	98.9	4	50
**2000**	3.K	Traffic deaths (per 100,000 population)	1	55	0.07	62.5	67	-12
**2000**	11.A	Access to improved water source, piped (% of urban population)	1	56	0.06	95.7	18	38
**2000**	15.B	Mean area that is protected in freshwater sites important to biodiversity (%)	1	57	0.05	39.0	86	-29
**2000**	14.F	Fish caught by trawling or dredging (%)	1	58	0.05	70.1	58	0
**2000**	8.F	Victims of modern slavery (per 1,000 population)	1	59	0.04	86.2	34	25

Year	SDG indicators	Indicator name	Window group	Rank of RCA in this group	Average RCA in this group	Average SDG score in this group	Rank of SDG score in this group	Rank difference (score-RCA) for window group
**2000**	3.N	Surviving infants who received 2 WHO-recommended vaccines (%)	1	60	0.04	82.8	38	22
**2000**	3.J	Incidence of tuberculosis (per 100,000 population)	1	61	0.02	92.8	23	38
**2000**	15.D	Permanent deforestation (% of forest area, 3-year average)	1	62	0.02	98.0	7	55
**2000**	16.F	Homicides (per 100,000 population)	1	63	0.02	90.6	25	38
**2000**	16.J	Birth registrations with civil authority (% of children under age 5)	1	64	0.01	99.8	1	63
**2000**	3.H	Age-standardized death rate attributable to household air pollution and ambient air pollution (per 100,000 population)	1	65	0.01	92.0	24	41
**2000**	8.B	Adjusted GDP growth (%)	1	66	0.01	75.4	49	17
**2000**	11.B	Annual mean concentration of particulate matter of less than 2.5 microns in diameter (PM2.5) (μg/m³)	1	67	-0.01	85.8	35	32
**2000**	15.C	Mean area that is protected in terrestrial sites important to biodiversity (%)	1	68	-0.03	36.6	89	-21
**2000**	2.D	Sustainable Nitrogen Management Index (best 0-1.41 worst)	1	69	-0.04	28.1	92	-23
**2000**	7.B	CO2 emissions from fuel combustion per total electricity output (MtCO2 TWh)	1	70	-0.04	76.2	47	23
**2000**	13.A	CO2/emissions embodied in fossil fuel exports (kg/capita)	1	71	-0.05	94.5	19	52
**2000**	11.D	Satisfaction with public transport (%)	1	72	-0.05	62.2	69	3
**2000**	16.I	Population who feel safe walking alone at night in the city or area where they live (%)	1	73	-0.08	53.6	76	-3
**2000**	5.C	Ratio of female-to-male labor force participation rate (%)	1	74	-0.08	66.0	63	11
**2000**	14.D	Fish caught that are then discarded (%)	1	75	-0.11	45.5	84	-9
**2000**	15.E	Red List Index of species survival (worst 0-1 best)	1	76	-0.13	75.9	48	28
**2000**	12.G	Production-based SO2 emissions (kg/capita)	1	77	-0.13	96.9	12	65
**2000**	8.C	Fatal work-related accidents embodied in imports (per 100,000 population)	1	78	-0.13	97.1	11	67
**2000**	6.A	Freshwater withdrawal (% of available freshwater resources)	1	79	-0.14	83.5	37	42
**2000**	14.A	Marine biodiversity threats embodied in imports (per million population)	1	80	-0.16	88.2	31	49

Year	SDG indicators	Indicator name	Window group	Rank of RCA in this group	Average RCA in this group	Average SDG score in this group	Rank of SDG score in this group	Rank difference (score-RCA) for window group
**2000**	12.F	SO2 emissions embodied in imports (kg/capita)	1	81	-0.20	80.5	40	41
**2000**	8.G	Unemployment rate (% of total labor force, ages 15+)	1	82	-0.21	67.1	62	20
**2000**	8.D	Victims of modern slavery embodied in imports (per 100,000 population)	1	83	-0.22	75.0	51	32
**2000**	6.C	Scarce water consumption embodied in imports (m3 H2O eq/capita)	1	84	-0.23	70.7	53	31
**2000**	12.D	Nitrogen emissions embodied in imports (kg/capita)	1	85	-0.26	60.3	71	14
**2000**	15.A	Terrestrial and freshwater biodiversity threats embodied in imports (per million population)	1	86	-0.27	77.3	44	42
**2000**	17.A	Corporate Tax Haven Score (best 0-100 worst)	1	87	-0.28	76.8	45	42
**2000**	16.K	Exports of major conventional weapons (TIV constant million USD per 100,000 population)	1	88	-0.28	79.4	43	45
**2000**	12.C	Municipal solid waste (kg/capita/day)	1	89	-0.29	64.5	65	24
**2000**	2.B	Prevalence of obesity, BMI ≥ 30 (% of adult population)	1	90	-0.32	58.0	73	17
**2000**	12.E	Production-based nitrogen emissions (kg/capita)	1	91	-0.32	50.2	81	10
**2000**	13.B	CO2 emissions from fossil fuel combustion and cement production (tCO2/capita)	1	92	-0.35	61.6	70	22
**2000**	13.C	CO2 emissions embodied in imports (tCO2/capita)	1	93	-0.40	48.6	83	10
**2000**	12.B	Exports of plastic waste (kg/capita)	1	94	-0.43	50.5	80	14
**2000**	12.A	Electronic waste (kg/capita)	1	95	-0.49	38.5	87	8
**2000**	2.F	Human Trophic Level (best 2-3 worst)	1	96	-0.60	19.9	94	2

Supplementary Table 3 | The RCA and SDG scores of SDG indicators in the 40th window group of countries in 2000. Note: The SDG indicators listed by the rank order of revealed comparative advantage (RCA). The RCA results are generated by the moving-window approach with window size of 50 (see Methods).

Year	SDG indicators	Indicator name	Window group	Rank of RCA in this group	Average RCA in this group	Average SDG score in this group	Rank of SDG score in this group	Rank difference (score-RCA) for window group
**2000**	14.E	Fish caught from overexploited or collapsed stocks (% of total catch)	40	1	0.59	76.9	38	-37
**2000**	16.B	Children involved in child labor (% of population aged 5 to 14)	40	2	0.58	86.6	22	-20
**2000**	1.A	Poverty headcount ratio at $3.65/day (2017 PPP, %)	40	3	0.47	83.4	26	-23
**2000**	3.C	New HIV infections (per 1,000 uninfected population)	40	4	0.40	94.4	6	-2
**2000**	4.B	Literacy rate (% of population aged 15 to 24)	40	5	0.39	94.6	5	0
**2000**	14.F	Fish caught by trawling or dredging (%)	40	6	0.31	75.1	43	-37
**2000**	14.B	Ocean Health Index: Clean Waters score (worst 0-100 best)	40	7	0.30	44.3	73	-66
**2000**	7.C	Population with access to electricity (%)	40	8	0.28	93.6	8	0
**2000**	2.C	Exports of hazardous pesticides (tonnes per million population)	40	9	0.28	88.1	17	-8
**2000**	3.A	Births attended by skilled health personnel (%)	40	10	0.25	89.3	13	-3
**2000**	11.C	Proportion of urban population living in slums (%)	40	11	0.25	74.3	45	-34
**2000**	6.B	Population using at least basic sanitation services (%)	40	12	0.24	81.5	30	-18
**2000**	7.A	Population with access to clean fuels and technology for cooking (%)	40	13	0.24	74.8	44	-31
**2000**	3.L	Mortality rate, under-5 (per 1,000 live births)	40	14	0.24	80.0	33	-19
**2000**	1.B	Poverty headcount ratio at $2.15/day (2017 PPP, %)	40	15	0.23	95.4	4	11
**2000**	3.D	Life expectancy at birth (years)	40	16	0.23	61.5	62	-46
**2000**	14.D	Fish caught that are then discarded (%)	40	17	0.22	53.7	66	-49
**2000**	4.A	Participation rate in pre-primary organized learning (% of children aged 4 to 6)	40	18	0.22	65.8	54	-36
**2000**	16.D	Unsentenced detainees (% of prison population)	40	19	0.21	63.2	60	-41
**2000**	4.D	Lower secondary completion rate (%)	40	20	0.21	72.1	46	-26
**2000**	16.G	Access to and affordability of justice (worst 0–1 best)	40	21	0.21	67.9	51	-30
**2000**	4.C	Net primary enrollment rate (%)	40	22	0.20	90.2	12	10
**2000**	3.E	Maternal mortality rate (per 100,000 live births)	40	23	0.20	92.2	10	13
**2000**	6.E	Population using at least basic drinking water services (%)	40	24	0.20	86.0	23	1
**2000**	5.A	Ratio of female-to-male mean years of education received (%)	40	25	0.19	82.8	27	-2
**2000**	14.C	Mean area that is protected in marine sites important to biodiversity (%)	40	26	0.18	24.4	88	-62
**2000**	3.N	Surviving infants who received 2 WHO-recommended vaccines (%)	40	27	0.18	81.1	31	-4

Year	SDG indicators	Indicator name	Window group	Rank of RCA in this group	Average RCA in this group	Average SDG score in this group	Rank of SDG score in this group	Rank difference (score-RCA) for window group
**2000**	2.G	Prevalence of undernourishment (%)	40	28	0.16	82.3	28	0
**2000**	2.E	Prevalence of stunting in children under 5 years of age (%)	40	29	0.15	67.2	52	-23
**2000**	3.G	Neonatal mortality rate (per 1,000 live births)	40	30	0.14	63.8	58	-28
**2000**	16.J	Birth registrations with civil authority (% of children under age 5)	40	31	0.14	97.2	3	28
**2000**	11.A	Access to improved water source, piped (% of urban population)	40	32	0.14	89.2	14	18
**2000**	11.D	Satisfaction with public transport (%)	40	33	0.14	64.0	57	-24
**2000**	3.B	Adolescent fertility rate (births per 1,000 females aged 15 to 19)	40	34	0.13	70.6	48	-14
**2000**	3.J	Incidence of tuberculosis (per 100,000 population)	40	35	0.13	87.7	20	15
**2000**	8.F	Victims of modern slavery (per 1,000 population)	40	36	0.12	79.9	34	2
**2000**	12.B	Exports of plastic waste (kg/capita)	40	37	0.11	85.8	24	13
**2000**	7.D	Renewable energy share in total final energy consumption (%)	40	38	0.11	22.3	89	-51
**2000**	5.B	Demand for family planning satisfied by modern methods (% of females aged 15 to 49)	40	39	0.10	54.4	64	-25
**2000**	3.K	Traffic deaths (per 100,000 population)	40	40	0.09	54.2	65	-25
**2000**	9.F	Rural population with access to all-season roads (%)	40	41	0.09	81.8	29	12
**2000**	13.A	CO2/emissions embodied in fossil fuel exports (kg/capita)	40	42	0.09	93.7	7	35
**2000**	13.C	CO2 emissions embodied in imports (tCO2/capita)	40	43	0.09	80.2	32	11
**2000**	8.E	Fundamental labor rights are effectively guaranteed (worst 0–1 best)	40	44	0.08	49.2	70	-26
**2000**	3.I	Subjective well-being (average ladder score, worst 0-10 best)	40	45	0.08	51.7	68	-23
**2000**	3.H	Age-standardized death rate attributable to household air pollution and ambient air pollution (per 100,000 population)	40	46	0.07	83.7	25	21
**2000**	15.D	Permanent deforestation (% of forest area, 3-year average)	40	47	0.07	87.8	18	29
**2000**	16.A	Timeliness of administrative proceedings (worst 0 - 1 best)	40	48	0.06	45.8	72	-24
**2000**	12.E	Production-based nitrogen emissions (kg/capita)	40	49	0.04	70.1	49	0
**2000**	16.F	Homicides (per 100,000 population)	40	50	0.04	78.7	37	13
**2000**	12.D	Nitrogen emissions embodied in imports (kg/capita)	40	51	0.04	75.8	40	11
**2000**	2.H	Prevalence of wasting in children under 5 years of age (%)	40	52	0.03	71.5	47	5
**2000**	12.F	SO2 emissions embodied in imports (kg/capita)	40	53	0.02	91.7	11	42
**2000**	11.B	Annual mean concentration of particulate matter of less than 2.5 microns in diameter (PM2.5) (μg/m³)	40	54	0.02	75.4	41	13
**2000**	9.G	The Times Higher Education Universities Ranking: Average score of top 3 universities (worst 0-100 best)	40	55	0.01	26.4	85	-30

Year	SDG indicators	Indicator name	Window group	Rank of RCA in this group	Average RCA in this group	Average SDG score in this group	Rank of SDG score in this group	Rank difference (score-RCA) for window group
**2000**	8.D	Victims of modern slavery embodied in imports (per 100,000 population)	40	56	0.01	86.9	21	35
**2000**	7.B	CO2 emissions from fuel combustion per total electricity output (MtCO2 TWh)	40	57	0.01	69.4	50	7
**2000**	8.C	Fatal work-related accidents embodied in imports (per 100,000 population)	40	58	0.00	98.9	1	57
**2000**	12.A	Electronic waste (kg/capita)	40	59	0.00	66.6	53	6
**2000**	12.G	Production-based SO2 emissions (kg/capita)	40	60	-0.01	97.2	2	58
**2000**	8.A	Adults with an account at a bank or other financial institution or with a mobile-money-service provider (% of population aged 15 or over)	40	61	-0.01	41.7	77	-16
**2000**	10.A	Gini coefficient	40	62	-0.01	61.2	63	-1
**2000**	3.F	Age-standardized death rate due to cardiovascular disease, cancer, diabetes, or chronic respiratory disease in adults aged 30–70 years (%)	40	63	-0.01	39.7	78	-15
**2000**	8.B	Adjusted GDP growth (%)	40	64	-0.01	63.6	59	5
**2000**	14.A	Marine biodiversity threats embodied in imports (per million population)	40	65	-0.02	88.5	16	49
**2000**	2.D	Sustainable Nitrogen Management Index (best 0-1.41 worst)	40	66	-0.02	24.5	87	-21
**2000**	6.C	Scarce water consumption embodied in imports (m3 H2O eq/capita)	40	67	-0.02	79.8	35	32
**2000**	17.C	Statistical Performance Index (worst 0-100 best)	40	68	-0.02	47.8	71	-3
**2000**	2.A	Cereal yield (tonnes per hectare of harvested land)	40	69	-0.03	36.4	81	-12
**2000**	16.K	Exports of major conventional weapons (TIV constant million USD per 100,000 population)	40	70	-0.03	93.3	9	61
**2000**	12.C	Municipal solid waste (kg/capita/day)	40	71	-0.03	76.0	39	32
**2000**	17.A	Corporate Tax Haven Score (best 0-100 worst)	40	72	-0.03	88.7	15	57
**2000**	16.E	Expropriations are lawful and adequately compensated (worst 0 - 1 best)	40	73	-0.04	30.0	83	-10
**2000**	13.B	CO2 emissions from fossil fuel combustion and cement production (tCO2/capita)	40	74	-0.04	79.6	36	38
**2000**	15.A	Terrestrial and freshwater biodiversity threats embodied in imports (per million population)	40	75	-0.04	87.7	19	56
**2000**	15.B	Mean area that is protected in freshwater sites important to biodiversity (%)	40	76	-0.06	29.3	84	-8
**2000**	17.B	Government spending on health and education (% of GDP)	40	77	-0.06	43.5	75	2
**2000**	3.M	Universal health coverage (UHC) index of service coverage (worst 0-100 best)	40	78	-0.08	20.3	90	-12
**2000**	6.A	Freshwater withdrawal (% of available freshwater resources)	40	79	-0.09	75.1	42	37
**2000**	10.B	Palma ratio	40	80	-0.10	38.5	79	1

Year	SDG indicators	Indicator name	Window group	Rank of RCA in this group	Average RCA in this group	Average SDG score in this group	Rank of SDG score in this group	Rank difference (score-RCA) for window group
**2000**	9.C	Logistics Performance Index: Quality of trade and transport-related infrastructure (worst 1-5 best)	40	81	-0.10	42.5	76	5
**2000**	16.I	Population who feel safe walking alone at night in the city or area where they live (%)	40	82	-0.10	44.1	74	8
**2000**	2.B	Prevalence of obesity, BMI ≥ 30 (% of adult population)	40	83	-0.12	64.6	56	27
**2000**	15.E	Red List Index of species survival (worst 0-1 best)	40	84	-0.12	64.9	55	29
**2000**	16.C	Corruption Perceptions Index (worst 0-100 best)	40	85	-0.14	36.2	82	3
**2000**	8.G	Unemployment rate (% of total labor force, ages 15+)	40	86	-0.14	62.5	61	25
**2000**	16.H	Press Freedom Index (worst 0-100 best)	40	87	-0.16	51.9	67	20
**2000**	2.F	Human Trophic Level (best 2-3 worst)	40	88	-0.16	37.4	80	8
**2000**	5.C	Ratio of female-to-male labor force participation rate (%)	40	89	-0.18	50.0	69	20
**2000**	15.C	Mean area that is protected in terrestrial sites important to biodiversity (%)	40	90	-0.21	24.9	86	4
**2000**	5.D	Seats held by women in national parliament (%)	40	91	-0.23	18.0	91	0
**2000**	9.E	Expenditure on research and development (% of GDP)	40	92	-0.36	9.8	92	0
**2000**	9.D	Mobile broadband subscriptions (per 100 population)	40	93	-0.46	4.4	95	-2
**2000**	9.B	Population using the internet (%)	40	94	-0.54	3.3	96	-2
**2000**	6.D	Anthropogenic wastewater that receives treatment (%)	40	95	-0.60	9.5	93	2
**2000**	9.A	Articles published in academic journals (per 1,000 population)	40	96	-0.62	7.7	94	2

Supplementary Table 4 | The RCA and SDG scores of SDG indicators in the 80th window group of countries in 2000. Note: The SDG indicators listed by the rank order of revealed comparative advantage (RCA). The RCA results are generated by the moving-window approach with window size of 50 (see Methods).

Year	SDG indicators	Indicator name	Window group	Rank of RCA in this group	Average RCA in this group	Average SDG score in this group	Rank of SDG score in this group	Rank difference (score-RCA) for window group
**2000**	14.E	Fish caught from overexploited or collapsed stocks (% of total catch)	80	1	0.90	81.5	14	-13
**2000**	14.D	Fish caught that are then discarded (%)	80	2	0.49	56.3	53	-51
**2000**	2.C	Exports of hazardous pesticides (tonnes per million population)	80	3	0.44	88.7	9	-6
**2000**	16.B	Children involved in child labor (% of population aged 5 to 14)	80	4	0.41	68.6	36	-32
**2000**	14.F	Fish caught by trawling or dredging (%)	80	5	0.40	71.0	31	-26
**2000**	12.B	Exports of plastic waste (kg/capita)	80	6	0.36	93.3	6	0
**2000**	4.B	Literacy rate (% of population aged 15 to 24)	80	7	0.36	81.9	13	-6
**2000**	2.F	Human Trophic Level (best 2-3 worst)	80	8	0.34	51.6	60	-52
**2000**	3.C	New HIV infections (per 1,000 uninfected population)	80	9	0.32	77.5	22	-13
**2000**	11.D	Satisfaction with public transport (%)	80	10	0.29	64.5	43	-33
**2000**	14.B	Ocean Health Index: Clean Waters score (worst 0-100 best)	80	11	0.28	38.3	75	-64
**2000**	12.A	Electronic waste (kg/capita)	80	12	0.26	73.5	28	-16
**2000**	13.C	CO2 emissions embodied in imports (tCO2/capita)	80	13	0.25	79.3	20	-7
**2000**	12.E	Production-based nitrogen emissions (kg/capita)	80	14	0.23	70.1	34	-20
**2000**	5.B	Demand for family planning satisfied by modern methods (% of females aged 15 to 49)	80	15	0.21	52.7	57	-42
**2000**	8.F	Victims of modern slavery (per 1,000 population)	80	16	0.20	76.2	23	-7
**2000**	14.A	Marine biodiversity threats embodied in imports (per million population)	80	17	0.19	93.5	4	13
**2000**	8.D	Victims of modern slavery embodied in imports (per 100,000 population)	80	18	0.19	87.3	10	8
**2000**	12.F	SO2 emissions embodied in imports (kg/capita)	80	19	0.19	91.2	8	11
**2000**	12.D	Nitrogen emissions embodied in imports (kg/capita)	80	20	0.18	74.1	27	-7
**2000**	1.B	Poverty headcount ratio at $2.15/day (2017 PPP, %)	80	21	0.18	80.6	17	4
**2000**	12.C	Municipal solid waste (kg/capita/day)	80	22	0.18	80.8	16	6
**2000**	15.A	Terrestrial and freshwater biodiversity threats embodied in imports (per million population)	80	23	0.17	93.4	5	18
**2000**	8.C	Fatal work-related accidents embodied in imports (per 100,000 population)	80	24	0.17	99.2	1	23
**2000**	16.G	Access to and affordability of justice (worst 0–1 best)	80	25	0.17	58.4	52	-27

Year	SDG indicators	Indicator name	Window group	Rank of RCA in this group	Average RCA in this group	Average SDG score in this group	Rank of SDG score in this group	Rank difference (score-RCA) for window group
**2000**	11.A	Access to improved water source, piped (% of urban population)	80	26	0.16	79.4	19	7
**2000**	12.G	Production-based SO2 emissions (kg/capita)	80	27	0.16	96.9	2	25
**2000**	6.C	Scarce water consumption embodied in imports (m3 H2O eq/capita)	80	28	0.15	80.1	18	10
**2000**	16.K	Exports of major conventional weapons (TIV constant million USD per 100,000 population)	80	29	0.15	96.4	3	26
**2000**	17.A	Corporate Tax Haven Score (best 0-100 worst)	80	30	0.15	92.3	7	23
**2000**	4.C	Net primary enrollment rate (%)	80	31	0.14	75.1	25	6
**2000**	8.G	Unemployment rate (% of total labor force, ages 15+)	80	32	0.14	72.1	29	3
**2000**	16.F	Homicides (per 100,000 population)	80	33	0.13	74.7	26	7
**2000**	16.J	Birth registrations with civil authority (% of children under age 5)	80	34	0.13	84.7	12	22
**2000**	13.A	CO2/emissions embodied in fossil fuel exports (kg/capita)	80	35	0.13	85.6	11	24
**2000**	11.C	Proportion of urban population living in slums (%)	80	36	0.13	59.7	49	-13
**2000**	15.D	Permanent deforestation (% of forest area, 3-year average)	80	37	0.12	81.4	15	22
**2000**	3.E	Maternal mortality rate (per 100,000 live births)	80	38	0.12	75.7	24	14
**2000**	16.D	Unsentenced detainees (% of prison population)	80	39	0.11	51.0	61	-22
**2000**	14.C	Mean area that is protected in marine sites important to biodiversity (%)	80	40	0.11	20.2	87	-47
**2000**	2.B	Prevalence of obesity, BMI ≥ 30 (% of adult population)	80	41	0.10	70.5	32	9
**2000**	13.B	CO2 emissions from fossil fuel combustion and cement production (tCO2/capita)	80	42	0.10	78.9	21	21
**2000**	7.B	CO2 emissions from fuel combustion per total electricity output (MtCO2 TWh)	80	43	0.10	65.4	42	1
**2000**	3.N	Surviving infants who received 2 WHO-recommended vaccines (%)	80	44	0.10	67.4	38	6
**2000**	16.I	Population who feel safe walking alone at night in the city or area where they live (%)	80	45	0.09	47.3	64	-19
**2000**	16.A	Timeliness of administrative proceedings (worst 0 - 1 best)	80	46	0.08	41.6	72	-26
**2000**	5.A	Ratio of female-to-male mean years of education received (%)	80	47	0.08	67.9	37	10
**2000**	3.K	Traffic deaths (per 100,000 population)	80	48	0.06	45.9	67	-19
**2000**	7.C	Population with access to electricity (%)	80	49	0.05	68.7	35	14
**2000**	6.E	Population using at least basic drinking water services (%)	80	50	0.05	66.8	39	11
**2000**	8.B	Adjusted GDP growth (%)	80	51	0.05	59.3	50	1
**2000**	2.H	Prevalence of wasting in children under 5 years of age (%)	80	52	0.05	64.0	46	6
**2000**	3.I	Subjective well-being (average ladder score, worst 0-10 best)	80	53	0.04	44.8	69	-16

Year	SDG indicators	Indicator name	Window group	Rank of RCA in this group	Average RCA in this group	Average SDG score in this group	Rank of SDG score in this group	Rank difference (score-RCA) for window group
**2000**	4.A	Participation rate in pre-primary organized learning (% of children aged 4 to 6)	80	54	0.04	50.5	63	-9
**2000**	2.G	Prevalence of undernourishment (%)	80	55	0.04	65.5	41	14
**2000**	3.L	Mortality rate, under-5 (per 1,000 live births)	80	56	0.02	59.0	51	5
**2000**	3.H	Age-standardized death rate attributable to household air pollution and ambient air pollution (per 100,000 population)	80	57	0.02	70.4	33	24
**2000**	10.A	Gini coefficient	80	58	0.02	52.6	58	0
**2000**	8.E	Fundamental labor rights are effectively guaranteed (worst 0–1 best)	80	59	0.01	40.8	73	-14
**2000**	3.A	Births attended by skilled health personnel (%)	80	60	0.01	64.5	44	16
**2000**	4.D	Lower secondary completion rate (%)	80	61	0.00	53.1	56	5
**2000**	9.F	Rural population with access to all-season roads (%)	80	62	0.00	66.0	40	22
**2000**	15.E	Red List Index of species survival (worst 0-1 best)	80	63	0.00	64.4	45	18
**2000**	3.B	Adolescent fertility rate (births per 1,000 females aged 15 to 19)	80	64	-0.01	54.3	55	9
**2000**	2.D	Sustainable Nitrogen Management Index (best 0-1.41 worst)	80	65	-0.01	21.6	86	-21
**2000**	6.A	Freshwater withdrawal (% of available freshwater resources)	80	66	-0.01	71.5	30	36
**2000**	15.B	Mean area that is protected in freshwater sites important to biodiversity (%)	80	67	-0.02	27.5	83	-16
**2000**	7.A	Population with access to clean fuels and technology for cooking (%)	80	68	-0.03	52.4	59	9
**2000**	3.D	Life expectancy at birth (years)	80	69	-0.04	43.0	71	-2
**2000**	2.A	Cereal yield (tonnes per hectare of harvested land)	80	70	-0.04	31.9	79	-9
**2000**	9.C	Logistics Performance Index: Quality of trade and transport-related infrastructure (worst 1-5 best)	80	71	-0.05	39.7	74	-3
**2000**	11.B	Annual mean concentration of particulate matter of less than 2.5 microns in diameter (PM2.5) (μg/m³)	80	72	-0.06	61.9	48	24
**2000**	3.G	Neonatal mortality rate (per 1,000 live births)	80	73	-0.06	46.6	66	7
**2000**	16.H	Press Freedom Index (worst 0-100 best)	80	74	-0.06	50.8	62	12
**2000**	17.B	Government spending on health and education (% of GDP)	80	75	-0.06	38.2	76	-1
**2000**	6.B	Population using at least basic sanitation services (%)	80	76	-0.07	54.9	54	22
**2000**	16.E	Expropriations are lawful and adequately compensated (worst 0 - 1 best)	80	77	-0.07	26.1	84	-7
**2000**	3.J	Incidence of tuberculosis (per 100,000 population)	80	78	-0.07	63.9	47	31
**2000**	3.F	Age-standardized death rate due to cardiovascular disease, cancer, diabetes, or chronic respiratory disease in adults aged 30–70 years (%)	80	79	-0.08	32.4	78	1
**2000**	15.C	Mean area that is protected in terrestrial sites important to biodiversity (%)	80	80	-0.10	25.4	85	-5

Year	SDG indicators	Indicator name	Window group	Rank of RCA in this group	Average RCA in this group	Average SDG score in this group	Rank of SDG score in this group	Rank difference (score-RCA) for window group
**2000**	5.C	Ratio of female-to-male labor force participation rate (%)	80	81	-0.12	47.2	65	16
**2000**	2.E	Prevalence of stunting in children under 5 years of age (%)	80	82	-0.12	45.7	68	14
**2000**	16.C	Corruption Perceptions Index (worst 0-100 best)	80	83	-0.13	31.7	80	3
**2000**	1.A	Poverty headcount ratio at $3.65/day (2017 PPP, %)	80	84	-0.14	44.4	70	14
**2000**	8.A	Adults with an account at a bank or other financial institution or with a mobile-money-service provider (% of population aged 15 or over)	80	85	-0.15	31.6	81	4
**2000**	5.D	Seats held by women in national parliament (%)	80	86	-0.16	17.4	88	-2
**2000**	10.B	Palma ratio	80	87	-0.17	29.0	82	5
**2000**	17.C	Statistical Performance Index (worst 0-100 best)	80	88	-0.19	35.1	77	11
**2000**	7.D	Renewable energy share in total final energy consumption (%)	80	89	-0.24	13.9	89	0
**2000**	3.M	Universal health coverage (UHC) index of service coverage (worst 0-100 best)	80	90	-0.44	11.1	90	0
**2000**	9.E	Expenditure on research and development (% of GDP)	80	91	-0.54	6.2	93	-2
**2000**	9.G	The Times Higher Education Universities Ranking: Average score of top 3 universities (worst 0-100 best)	80	92	-0.56	10.0	91	1
**2000**	6.D	Anthropogenic wastewater that receives treatment (%)	80	93	-0.57	8.4	92	1
**2000**	9.D	Mobile broadband subscriptions (per 100 population)	80	94	-0.62	2.7	95	-1
**2000**	9.B	Population using the internet (%)	80	95	-0.78	1.4	96	-1
**2000**	9.A	Articles published in academic journals (per 1,000 population)	80	96	-0.81	3.4	94	2

Supplementary Table 5 | The RCA and SDG scores of SDG indicators in the 117th window group of countries in 2000. Note: The SDG indicators listed by the rank order of revealed comparative advantage (RCA). The RCA results are generated by the moving-window approach with window size of 50 (see Methods).

Year	SDG indicators	Indicator name	Window group	Rank of RCA in this group	Average RCA in this group	Average SDG score in this group	Rank of SDG score in this group	Rank difference (score-RCA) for window group
**2000**	14.E	Fish caught from overexploited or collapsed stocks (% of total catch)	117	1	1.39	83.9	21	-20
**2000**	2.F	Human Trophic Level (best 2-3 worst)	117	2	1.26	71.2	26	-24
**2000**	14.D	Fish caught that are then discarded (%)	117	3	1.14	69.0	28	-25
**2000**	12.A	Electronic waste (kg/capita)	117	4	1.01	95.1	14	-10
**2000**	13.C	CO2 emissions embodied in imports (tCO2/capita)	117	5	0.91	96.7	12	-7
**2000**	12.E	Production-based nitrogen emissions (kg/capita)	117	6	0.91	88.7	20	-14
**2000**	2.C	Exports of hazardous pesticides (tonnes per million population)	117	7	0.89	94.1	15	-8
**2000**	12.D	Nitrogen emissions embodied in imports (kg/capita)	117	8	0.85	93.0	16	-8
**2000**	2.B	Prevalence of obesity, BMI ≥ 30 (% of adult population)	117	9	0.79	92.9	17	-8
**2000**	12.B	Exports of plastic waste (kg/capita)	117	10	0.77	99.1	6	4
**2000**	14.F	Fish caught by trawling or dredging (%)	117	11	0.75	71.7	25	-14
**2000**	6.C	Scarce water consumption embodied in imports (m3 H2O eq/capita)	117	12	0.73	96.2	13	-1
**2000**	13.B	CO2 emissions from fossil fuel combustion and cement production (tCO2/capita)	117	13	0.71	98.2	9	4
**2000**	12.C	Municipal solid waste (kg/capita/day)	117	14	0.67	91.2	18	-4
**2000**	13.A	CO2/emissions embodied in fossil fuel exports (kg/capita)	117	15	0.66	99.5	4	11
**2000**	8.D	Victims of modern slavery embodied in imports (per 100,000 population)	117	16	0.65	97.2	11	5
**2000**	12.F	SO2 emissions embodied in imports (kg/capita)	117	17	0.60	98.5	8	9
**2000**	6.A	Freshwater withdrawal (% of available freshwater resources)	117	18	0.55	89.4	19	-1
**2000**	17.A	Corporate Tax Haven Score (best 0-100 worst)	117	19	0.54	99.1	5	14
**2000**	5.C	Ratio of female-to-male labor force participation rate (%)	117	20	0.54	66.2	29	-9
**2000**	14.A	Marine biodiversity threats embodied in imports (per million population)	117	21	0.53	98.6	7	14
**2000**	15.A	Terrestrial and freshwater biodiversity threats embodied in imports (per million population)	117	22	0.53	98.1	10	12
**2000**	14.C	Mean area that is protected in marine sites important to biodiversity (%)	117	23	0.52	23.4	67	-44
**2000**	16.K	Exports of major conventional weapons (TIV constant million USD per 100,000 population)	117	24	0.49	100.0	1	23
**2000**	12.G	Production-based SO2 emissions (kg/capita)	117	25	0.49	99.6	3	22

Year	SDG indicators	Indicator name	Window group	Rank of RCA in this group	Average RCA in this group	Average SDG score in this group	Rank of SDG score in this group	Rank difference (score-RCA) for window group
**2000**	8.C	Fatal work-related accidents embodied in imports (per 100,000 population)	117	26	0.47	99.9	2	24
**2000**	16.F	Homicides (per 100,000 population)	117	27	0.45	79.0	22	5
**2000**	8.G	Unemployment rate (% of total labor force, ages 15+)	117	28	0.43	73.5	24	4
**2000**	15.C	Mean area that is protected in terrestrial sites important to biodiversity (%)	117	29	0.41	31.8	54	-25
**2000**	10.A	Gini coefficient	117	30	0.38	58.9	33	-3
**2000**	15.E	Red List Index of species survival (worst 0-1 best)	117	31	0.37	69.9	27	4
**2000**	15.D	Permanent deforestation (% of forest area, 3-year average)	117	32	0.36	77.4	23	9
**2000**	3.C	New HIV infections (per 1,000 uninfected population)	117	33	0.34	62.0	31	2
**2000**	16.I	Population who feel safe walking alone at night in the city or area where they live (%)	117	34	0.33	45.6	43	-9
**2000**	15.B	Mean area that is protected in freshwater sites important to biodiversity (%)	117	35	0.30	28.4	56	-21
**2000**	16.G	Access to and affordability of justice (worst 0–1 best)	117	36	0.24	50.4	38	-2
**2000**	16.H	Press Freedom Index (worst 0-100 best)	117	37	0.17	50.7	37	0
**2000**	2.D	Sustainable Nitrogen Management Index (best 0-1.41 worst)	117	38	0.17	21.4	71	-33
**2000**	8.F	Victims of modern slavery (per 1,000 population)	117	39	0.17	59.8	32	7
**2000**	11.A	Access to improved water source, piped (% of urban population)	117	40	0.13	62.4	30	10
**2000**	11.B	Annual mean concentration of particulate matter of less than 2.5 microns in diameter (PM2.5) (μg/m³)	117	41	0.11	58.0	34	7
**2000**	7.B	CO2 emissions from fuel combustion per total electricity output (MtCO2 TWh)	117	42	0.09	52.6	36	6
**2000**	5.D	Seats held by women in national parliament (%)	117	43	0.07	17.5	78	-35
**2000**	16.B	Children involved in child labor (% of population aged 5 to 14)	117	44	0.06	40.7	45	-1
**2000**	8.E	Fundamental labor rights are effectively guaranteed (worst 0–1 best)	117	45	0.04	34.4	52	-7
**2000**	8.B	Adjusted GDP growth (%)	117	46	0.03	47.3	41	5
**2000**	16.A	Timeliness of administrative proceedings (worst 0 - 1 best)	117	47	0.02	31.8	53	-6
**2000**	10.B	Palma ratio	117	48	-0.02	29.5	55	-7
**2000**	16.D	Unsentenced detainees (% of prison population)	117	49	-0.02	37.4	48	1
**2000**	7.D	Renewable energy share in total final energy consumption (%)	117	50	-0.03	13.8	82	-32
**2000**	14.B	Ocean Health Index: Clean Waters score (worst 0-100 best)	117	51	-0.06	22.5	68	-17
**2000**	1.B	Poverty headcount ratio at $2.15/day (2017 PPP, %)	117	52	-0.08	50.0	39	13
**2000**	16.J	Birth registrations with civil authority (% of children under age 5)	117	53	-0.09	55.3	35	18

Year	SDG indicators	Indicator name	Window group	Rank of RCA in this group	Average RCA in this group	Average SDG score in this group	Rank of SDG score in this group	Rank difference (score-RCA) for window group
**2000**	3.H	Age-standardized death rate attributable to household air pollution and ambient air pollution (per 100,000 population)	117	54	-0.10	49.8	40	14
**2000**	4.B	Literacy rate (% of population aged 15 to 24)	117	55	-0.10	43.7	44	11
**2000**	17.B	Government spending on health and education (% of GDP)	117	56	-0.12	28.2	58	-2
**2000**	11.D	Satisfaction with public transport (%)	117	57	-0.13	34.7	51	6
**2000**	3.J	Incidence of tuberculosis (per 100,000 population)	117	58	-0.14	46.1	42	16
**2000**	3.K	Traffic deaths (per 100,000 population)	117	59	-0.21	27.7	59	0
**2000**	2.H	Prevalence of wasting in children under 5 years of age (%)	117	60	-0.21	38.4	47	13
**2000**	17.C	Statistical Performance Index (worst 0-100 best)	117	61	-0.22	27.5	60	1
**2000**	3.F	Age-standardized death rate due to cardiovascular disease, cancer, diabetes, or chronic respiratory disease in adults aged 30–70 years (%)	117	62	-0.23	21.0	72	-10
**2000**	16.E	Expropriations are lawful and adequately compensated (worst 0 - 1 best)	117	63	-0.26	17.3	80	-17
**2000**	9.F	Rural population with access to all-season roads (%)	117	64	-0.26	39.7	46	18
**2000**	2.G	Prevalence of undernourishment (%)	117	65	-0.29	36.6	49	16
**2000**	16.C	Corruption Perceptions Index (worst 0-100 best)	117	66	-0.30	20.9	73	-7
**2000**	9.C	Logistics Performance Index: Quality of trade and transport-related infrastructure (worst 1-5 best)	117	67	-0.33	22.3	69	-2
**2000**	4.C	Net primary enrollment rate (%)	117	68	-0.35	36.2	50	18
**2000**	3.I	Subjective well-being (average ladder score, worst 0-10 best)	117	69	-0.35	21.9	70	-1
**2000**	2.A	Cereal yield (tonnes per hectare of harvested land)	117	70	-0.36	17.4	79	-9
**2000**	3.N	Surviving infants who received 2 WHO-recommended vaccines (%)	117	71	-0.43	28.3	57	14
**2000**	5.B	Demand for family planning satisfied by modern methods (% of females aged 15 to 49)	117	72	-0.45	19.5	75	-3
**2000**	5.A	Ratio of female-to-male mean years of education received (%)	117	73	-0.45	27.2	62	11
**2000**	11.C	Proportion of urban population living in slums (%)	117	74	-0.46	23.7	65	9
**2000**	3.B	Adolescent fertility rate (births per 1,000 females aged 15 to 19)	117	75	-0.48	23.5	66	9
**2000**	4.A	Participation rate in pre-primary organized learning (% of children aged 4 to 6)	117	76	-0.49	20.8	74	2
**2000**	3.A	Births attended by skilled health personnel (%)	117	77	-0.50	25.7	63	14
**2000**	3.E	Maternal mortality rate (per 100,000 live births)	117	78	-0.51	27.4	61	17
**2000**	6.E	Population using at least basic drinking water services (%)	117	79	-0.53	24.3	64	15
**2000**	2.E	Prevalence of stunting in children under 5 years of age (%)	117	80	-0.56	17.7	77	3
**2000**	9.E	Expenditure on research and development (% of GDP)	117	81	-0.60	4.3	90	-9

Year	SDG indicators	Indicator name	Window group	Rank of RCA in this group	Average RCA in this group	Average SDG score in this group	Rank of SDG score in this group	Rank difference (score-RCA) for window group
**2000**	8.A	Adults with an account at a bank or other financial institution or with a mobile-money-service provider (% of population aged 15 or over)	117	82	-0.61	11.8	85	-3
**2000**	6.B	Population using at least basic sanitation services (%)	117	83	-0.67	15.9	81	2
**2000**	7.C	Population with access to electricity (%)	117	84	-0.67	18.1	76	8
**2000**	4.D	Lower secondary completion rate (%)	117	85	-0.69	13.7	83	2
**2000**	3.D	Life expectancy at birth (years)	117	86	-0.74	9.5	86	0
**2000**	3.L	Mortality rate, under-5 (per 1,000 live births)	117	87	-0.74	12.3	84	3
**2000**	3.G	Neonatal mortality rate (per 1,000 live births)	117	88	-0.78	8.9	87	1
**2000**	1.A	Poverty headcount ratio at $3.65/day (2017 PPP, %)	117	89	-0.79	8.7	89	0
**2000**	7.A	Population with access to clean fuels and technology for cooking (%)	117	90	-0.80	8.8	88	2
**2000**	9.G	The Times Higher Education Universities Ranking: Average score of top 3 universities (worst 0-100 best)	117	91	-0.82	3.2	91	0
**2000**	6.D	Anthropogenic wastewater that receives treatment (%)	117	92	-0.87	1.9	92	0
**2000**	9.A	Articles published in academic journals (per 1,000 population)	117	93	-0.95	0.7	93	0
**2000**	9.D	Mobile broadband subscriptions (per 100 population)	117	94	-0.99	0.1	95	-1
**2000**	9.B	Population using the internet (%)	117	95	-0.99	0.1	96	-1
**2000**	3.M	Universal health coverage (UHC) index of service coverage (worst 0-100 best)	117	96	-0.99	0.2	94	2

Supplementary Table 6 | The RCA and SDG scores of SDG indicators in the 1st window group of countries in 2015. Note: The SDG indicators listed by the rank order of revealed comparative advantage (RCA). The RCA results are generated by the moving-window approach with window size of 50 (see Methods).

Year	SDG indicators	Indicator name	Window group	Rank of RCA in this group	Average RCA in this group	Average SDG score in this group	Rank of SDG score in this group	Rank difference (score-RCA) for window group
**2015**	6.D	Anthropogenic wastewater that receives treatment (%)	1	1	1.04	54.5	84	-83
**2015**	9.A	Articles published in academic journals (per 1,000 population)	1	2	0.92	77.1	50	-48
**2015**	9.E	Expenditure on research and development (% of GDP)	1	3	0.91	41.3	91	-88
**2015**	9.G	The Times Higher Education Universities Ranking: Average score of top 3 universities (worst 0-100 best)	1	4	0.66	50.0	89	-85
**2015**	14.C	Mean area that is protected in marine sites important to biodiversity (%)	1	5	0.64	65.2	71	-66
**2015**	16.E	Expropriations are lawful and adequately compensated (worst 0 - 1 best)	1	6	0.63	61.0	81	-75
**2015**	16.B	Children involved in child labor (% of population aged 5 to 14)	1	7	0.52	96.4	18	-11
**2015**	8.E	Fundamental labor rights are effectively guaranteed (worst 0–1 best)	1	8	0.44	74.5	56	-48
**2015**	8.A	Adults with an account at a bank or other financial institution or with a mobile-money-service provider (% of population aged 15 or over)	1	9	0.44	80.6	44	-35
**2015**	4.A	Participation rate in pre-primary organized learning (% of children aged 4 to 6)	1	10	0.41	92.5	28	-18
**2015**	1.A	Poverty headcount ratio at $3.65/day (2017 PPP, %)	1	11	0.38	97.2	12	-1
**2015**	10.B	Palma ratio	1	12	0.38	76.7	52	-40
**2015**	16.A	Timeliness of administrative proceedings (worst 0 - 1 best)	1	13	0.36	66.3	70	-57
**2015**	3.M	Universal health coverage (UHC) index of service coverage (worst 0-100 best)	1	14	0.31	63.8	77	-63
**2015**	9.B	Population using the internet (%)	1	15	0.31	72.7	62	-47
**2015**	16.C	Corruption Perceptions Index (worst 0-100 best)	1	16	0.30	64.5	76	-60
**2015**	9.D	Mobile broadband subscriptions (per 100 population)	1	17	0.28	73.0	60	-43
**2015**	7.D	Renewable energy share in total final energy consumption (%)	1	18	0.27	34.8	95	-77
**2015**	16.G	Access to and affordability of justice (worst 0–1 best)	1	19	0.26	80.7	42	-23
**2015**	17.C	Statistical Performance Index (worst 0-100 best)	1	20	0.26	70.4	66	-46
**2015**	2.A	Cereal yield (tonnes per hectare of harvested land)	1	21	0.25	69.7	68	-47
**2015**	9.C	Logistics Performance Index: Quality of trade and transport-related infrastructure (worst 1-5 best)	1	22	0.24	76.1	53	-31
**2015**	3.I	Subjective well-being (average ladder score, worst 0-10 best)	1	23	0.24	69.3	69	-46
**2015**	3.K	Traffic deaths (per 100,000 population)	1	24	0.23	81.9	41	-17
**2015**	4.B	Literacy rate (% of population aged 15 to 24)	1	25	0.23	98.6	7	18
**2015**	7.A	Population with access to clean fuels and technology for cooking (%)	1	26	0.23	97.3	11	15
**2015**	11.C	Proportion of urban population living in slums (%)	1	27	0.23	97.4	10	17

Year	SDG indicators	Indicator name	Window group	Rank of RCA in this group	Average RCA in this group	Average SDG score in this group	Rank of SDG score in this group	Rank difference (score-RCA) for window group
**2015**	14.E	Fish caught from overexploited or collapsed stocks (% of total catch)	1	28	0.21	61.7	80	-52
**2015**	14.B	Ocean Health Index: Clean Waters score (worst 0-100 best)	1	29	0.21	42.7	90	-61
**2015**	16.D	Unsentenced detainees (% of prison population)	1	30	0.20	76.9	51	-21
**2015**	17.B	Government spending on health and education (% of GDP)	1	31	0.18	72.1	63	-32
**2015**	3.C	New HIV infections (per 1,000 uninfected population)	1	32	0.18	98.1	8	24
**2015**	15.B	Mean area that is protected in freshwater sites important to biodiversity (%)	1	33	0.16	64.7	74	-41
**2015**	3.F	Age-standardized death rate due to cardiovascular disease, cancer, diabetes, or chronic respiratory disease in adults aged 30–70 years (%)	1	34	0.15	74.0	58	-24
**2015**	3.B	Adolescent fertility rate (births per 1,000 females aged 15 to 19)	1	35	0.15	88.4	35	0
**2015**	3.G	Neonatal mortality rate (per 1,000 live births)	1	36	0.14	93.2	27	9
**2015**	4.D	Lower secondary completion rate (%)	1	37	0.14	94.3	24	13
**2015**	3.D	Life expectancy at birth (years)	1	38	0.13	85.3	39	-1
**2015**	15.C	Mean area that is protected in terrestrial sites important to biodiversity (%)	1	39	0.13	63.0	78	-39
**2015**	16.H	Press Freedom Index (worst 0-100 best)	1	40	0.13	78.6	46	-6
**2015**	2.C	Exports of hazardous pesticides (tonnes per million population)	1	41	0.13	89.6	32	9
**2015**	2.E	Prevalence of stunting in children under 5 years of age (%)	1	42	0.12	91.4	30	12
**2015**	9.F	Rural population with access to all-season roads (%)	1	43	0.11	96.3	19	24
**2015**	5.B	Demand for family planning satisfied by modern methods (% of females aged 15 to 49)	1	44	0.11	72.7	61	-17
**2015**	10.A	Gini coefficient	1	45	0.10	84.1	40	5
**2015**	6.B	Population using at least basic sanitation services (%)	1	46	0.10	96.0	22	24
**2015**	1.B	Poverty headcount ratio at $2.15/day (2017 PPP, %)	1	47	0.07	99.2	3	44
**2015**	11.A	Access to improved water source, piped (% of urban population)	1	48	0.06	96.6	17	31
**2015**	8.F	Victims of modern slavery (per 1,000 population)	1	49	0.06	86.3	37	12
**2015**	2.H	Prevalence of wasting in children under 5 years of age (%)	1	50	0.06	89.8	31	19
**2015**	2.D	Sustainable Nitrogen Management Index (best 0-1.41 worst)	1	51	0.06	38.2	94	-43
**2015**	2.G	Prevalence of undernourishment (%)	1	52	0.06	98.9	4	48
**2015**	16.F	Homicides (per 100,000 population)	1	53	0.05	93.7	26	27
**2015**	7.C	Population with access to electricity (%)	1	54	0.04	99.9	1	53
**2015**	6.E	Population using at least basic drinking water services (%)	1	55	0.04	97.5	9	46
**2015**	3.L	Mortality rate, under-5 (per 1,000 live births)	1	56	0.03	97.1	13	43
**2015**	3.A	Births attended by skilled health personnel (%)	1	57	0.03	98.8	5	52
**2015**	16.J	Birth registrations with civil authority (% of children under age 5)	1	58	0.02	99.8	2	56
**2015**	11.B	Annual mean concentration of particulate matter of less than 2.5 microns in diameter (PM2.5) (μg/m³)	1	59	0.02	88.8	34	25
**2015**	3.H	Age-standardized death rate attributable to household air pollution and ambient air pollution (per 100,000 population)	1	60	0.02	91.9	29	31

Year	SDG indicators	Indicator name	Window group	Rank of RCA in this group	Average RCA in this group	Average SDG score in this group	Rank of SDG score in this group	Rank difference (score-RCA) for window group
**2015**	15.D	Permanent deforestation (% of forest area, 3-year average)	1	61	0.02	96.7	16	45
**2015**	16.I	Population who feel safe walking alone at night in the city or area where they live (%)	1	62	0.02	59.4	82	-20
**2015**	5.A	Ratio of female-to-male mean years of education received (%)	1	63	0.01	96.1	21	42
**2015**	14.F	Fish caught by trawling or dredging (%)	1	64	0.01	73.5	59	5
**2015**	5.D	Seats held by women in national parliament (%)	1	65	0.01	50.5	88	-23
**2015**	8.B	Adjusted GDP growth (%)	1	66	0.01	74.7	55	11
**2015**	3.E	Maternal mortality rate (per 100,000 live births)	1	67	0.00	98.7	6	61
**2015**	4.C	Net primary enrollment rate (%)	1	68	-0.01	96.3	20	48
**2015**	11.D	Satisfaction with public transport (%)	1	69	-0.01	65.1	73	-4
**2015**	3.J	Incidence of tuberculosis (per 100,000 population)	1	70	-0.02	95.0	23	47
**2015**	14.D	Fish caught that are then discarded (%)	1	71	-0.05	56.5	83	-12
**2015**	13.A	CO2/emissions embodied in fossil fuel exports (kg/capita)	1	72	-0.05	94.2	25	47
**2015**	3.N	Surviving infants who received 2 WHO-recommended vaccines (%)	1	73	-0.06	87.8	36	37
**2015**	5.C	Ratio of female-to-male labor force participation rate (%)	1	74	-0.07	71.7	64	10
**2015**	15.E	Red List Index of species survival (worst 0-1 best)	1	75	-0.08	74.3	57	18
**2015**	7.B	CO2 emissions from fuel combustion per total electricity output (MtCO2 TWh)	1	76	-0.09	78.3	47	29
**2015**	6.A	Freshwater withdrawal (% of available freshwater resources)	1	77	-0.11	85.4	38	39
**2015**	12.G	Production-based SO2 emissions (kg/capita)	1	78	-0.11	97.1	14	64
**2015**	8.C	Fatal work-related accidents embodied in imports (per 100,000 population)	1	79	-0.12	96.9	15	64
**2015**	14.A	Marine biodiversity threats embodied in imports (per million population)	1	80	-0.14	88.9	33	47
**2015**	8.G	Unemployment rate (% of total labor force, ages 15+)	1	81	-0.18	70.0	67	14
**2015**	12.F	SO2 emissions embodied in imports (kg/capita)	1	82	-0.19	80.7	43	39
**2015**	8.D	Victims of modern slavery embodied in imports (per 100,000 population)	1	83	-0.21	75.6	54	29
**2015**	6.C	Scarce water consumption embodied in imports (m3 H2O eq/capita)	1	84	-0.21	71.5	65	19
**2015**	12.D	Nitrogen emissions embodied in imports (kg/capita)	1	85	-0.24	61.9	79	6
**2015**	15.A	Terrestrial and freshwater biodiversity threats embodied in imports (per million population)	1	86	-0.26	77.2	49	37
**2015**	17.A	Corporate Tax Haven Score (best 0-100 worst)	1	87	-0.27	77.3	48	39
**2015**	12.E	Production-based nitrogen emissions (kg/capita)	1	88	-0.27	54.4	85	3
**2015**	16.K	Exports of major conventional weapons (TIV constant million USD per 100,000 population)	1	89	-0.28	79.0	45	44
**2015**	12.C	Municipal solid waste (kg/capita/day)	1	90	-0.28	64.6	75	15
**2015**	13.B	CO2 emissions from fossil fuel combustion and cement production (tCO2/capita)	1	91	-0.30	65.1	72	19
**2015**	13.C	CO2 emissions embodied in imports (tCO2/capita)	1	92	-0.36	52.1	86	6
**2015**	2.B	Prevalence of obesity, BMI ≥ 30 (% of adult population)	1	93	-0.39	39.3	92	1

Year	SDG indicators	Indicator name	Window group	Rank of RCA in this group	Average RCA in this group	Average SDG score in this group	Rank of SDG score in this group	Rank difference (score-RCA) for window group
**2015**	12.B	Exports of plastic waste (kg/capita)	1	94	-0.42	50.9	87	7
**2015**	12.A	Electronic waste (kg/capita)	1	95	-0.49	38.6	93	2
**2015**	2.F	Human Trophic Level (best 2-3 worst)	1	96	-0.65	17.2	96	0

Supplementary Table 7 | The RCA and SDG scores of SDG indicators in the 40th window group of countries in 2015. Note: The SDG indicators listed by the rank order of revealed comparative advantage (RCA). The RCA results are generated by the moving-window approach with window size of 50 (see Methods).

Year	SDG indicators	Indicator name	Window group	Rank of RCA in this group	Average RCA in this group	Average SDG score in this group	Rank of SDG score in this group	Rank difference (score-RCA) for window group
**2015**	14.E	Fish caught from overexploited or collapsed stocks (% of total catch)	40	1	0.57	69.2	54	-53
**2015**	16.B	Children involved in child labor (% of population aged 5 to 14)	40	2	0.56	84.9	30	-28
**2015**	1.A	Poverty headcount ratio at $3.65/day (2017 PPP, %)	40	3	0.43	86.4	27	-24
**2015**	4.B	Literacy rate (% of population aged 15 to 24)	40	4	0.35	95.0	10	-6
**2015**	3.C	New HIV infections (per 1,000 uninfected population)	40	5	0.33	97.1	4	1
**2015**	2.C	Exports of hazardous pesticides (tonnes per million population)	40	6	0.28	89.6	22	-16
**2015**	14.B	Ocean Health Index: Clean Waters score (worst 0-100 best)	40	7	0.27	38.8	85	-78
**2015**	7.A	Population with access to clean fuels and technology for cooking (%)	40	8	0.24	84.8	32	-24
**2015**	14.F	Fish caught by trawling or dredging (%)	40	9	0.23	76.6	46	-37
**2015**	11.C	Proportion of urban population living in slums (%)	40	10	0.23	84.3	33	-23
**2015**	1.B	Poverty headcount ratio at $2.15/day (2017 PPP, %)	40	11	0.22	96.8	5	6
**2015**	6.B	Population using at least basic sanitation services (%)	40	12	0.20	90.1	20	-8
**2015**	3.M	Universal health coverage (UHC) index of service coverage (worst 0-100 best)	40	13	0.20	50.3	73	-60
**2015**	16.D	Unsentenced detainees (% of prison population)	40	14	0.19	66.5	58	-44
**2015**	4.D	Lower secondary completion rate (%)	40	15	0.19	85.1	29	-14
**2015**	16.G	Access to and affordability of justice (worst 0–1 best)	40	16	0.19	66.7	57	-41
**2015**	11.A	Access to improved water source, piped (% of urban population)	40	17	0.19	93.3	13	4
**2015**	7.C	Population with access to electricity (%)	40	18	0.18	98.3	2	16
**2015**	11.D	Satisfaction with public transport (%)	40	19	0.16	66.1	59	-40
**2015**	4.A	Participation rate in pre-primary organized learning (% of children aged 4 to 6)	40	20	0.16	66.1	60	-40
**2015**	3.A	Births attended by skilled health personnel (%)	40	21	0.15	95.5	8	13
**2015**	2.G	Prevalence of undernourishment (%)	40	22	0.15	93.6	11	11
**2015**	8.F	Victims of modern slavery (per 1,000 population)	40	23	0.15	81.0	38	-15
**2015**	16.J	Birth registrations with civil authority (% of children under age 5)	40	24	0.14	96.7	6	18
**2015**	13.A	CO2/emissions embodied in fossil fuel exports (kg/capita)	40	25	0.14	96.7	7	18
**2015**	6.E	Population using at least basic drinking water services (%)	40	26	0.13	91.8	16	10
**2015**	13.C	CO2 emissions embodied in imports (tCO2/capita)	40	27	0.13	82.4	36	-9
**2015**	3.G	Neonatal mortality rate (per 1,000 live births)	40	28	0.13	79.5	40	-12

Year	SDG indicators	Indicator name	Window group	Rank of RCA in this group	Average RCA in this group	Average SDG score in this group	Rank of SDG score in this group	Rank difference (score-RCA) for window group
**2015**	3.B	Adolescent fertility rate (births per 1,000 females aged 15 to 19)	40	29	0.13	74.3	49	-20
**2015**	3.D	Life expectancy at birth (years)	40	30	0.12	72.8	50	-20
**2015**	3.E	Maternal mortality rate (per 100,000 live births)	40	31	0.12	95.1	9	22
**2015**	2.E	Prevalence of stunting in children under 5 years of age (%)	40	32	0.12	78.2	43	-11
**2015**	4.C	Net primary enrollment rate (%)	40	33	0.11	93.5	12	21
**2015**	3.L	Mortality rate, under-5 (per 1,000 live births)	40	34	0.11	89.6	21	13
**2015**	12.B	Exports of plastic waste (kg/capita)	40	35	0.11	85.6	28	7
**2015**	2.D	Sustainable Nitrogen Management Index (best 0-1.41 worst)	40	36	0.10	34.8	89	-53
**2015**	16.F	Homicides (per 100,000 population)	40	37	0.10	83.5	35	2
**2015**	5.A	Ratio of female-to-male mean years of education received (%)	40	38	0.09	89.4	23	15
**2015**	12.D	Nitrogen emissions embodied in imports (kg/capita)	40	39	0.09	79.0	41	-2
**2015**	9.B	Population using the internet (%)	40	40	0.09	51.9	71	-31
**2015**	3.J	Incidence of tuberculosis (per 100,000 population)	40	41	0.09	90.3	18	23
**2015**	14.C	Mean area that is protected in marine sites important to biodiversity (%)	40	42	0.09	38.3	86	-44
**2015**	3.I	Subjective well-being (average ladder score, worst 0-10 best)	40	43	0.08	52.9	69	-26
**2015**	14.D	Fish caught that are then discarded (%)	40	44	0.07	53.8	67	-23
**2015**	15.D	Permanent deforestation (% of forest area, 3-year average)	40	45	0.07	87.4	26	19
**2015**	9.F	Rural population with access to all-season roads (%)	40	46	0.07	79.8	39	7
**2015**	10.A	Gini coefficient	40	47	0.06	69.6	52	-5
**2015**	3.N	Surviving infants who received 2 WHO-recommended vaccines (%)	40	48	0.06	84.9	31	17
**2015**	9.D	Mobile broadband subscriptions (per 100 population)	40	49	0.06	51.9	70	-21
**2015**	8.E	Fundamental labor rights are effectively guaranteed (worst 0–1 best)	40	50	0.06	48.0	80	-30
**2015**	16.A	Timeliness of administrative proceedings (worst 0 - 1 best)	40	51	0.06	45.0	82	-31
**2015**	3.H	Age-standardized death rate attributable to household air pollution and ambient air pollution (per 100,000 population)	40	52	0.05	81.8	37	15
**2015**	3.K	Traffic deaths (per 100,000 population)	40	53	0.05	59.4	63	-10
**2015**	8.D	Victims of modern slavery embodied in imports (per 100,000 population)	40	54	0.05	88.7	24	30
**2015**	12.F	SO2 emissions embodied in imports (kg/capita)	40	55	0.04	92.4	14	41
**2015**	12.E	Production-based nitrogen emissions (kg/capita)	40	56	0.04	69.8	51	5
**2015**	5.B	Demand for family planning satisfied by modern methods (% of females aged 15 to 49)	40	57	0.04	58.9	64	-7
**2015**	6.C	Scarce water consumption embodied in imports (m3 H2O eq/capita)	40	58	0.04	83.6	34	24
**2015**	7.B	CO2 emissions from fuel combustion per total electricity output (MtCO2 TWh)	40	59	0.03	76.8	45	14
**2015**	12.A	Electronic waste (kg/capita)	40	60	0.03	67.8	56	4

Year	SDG indicators	Indicator name	Window group	Rank of RCA in this group	Average RCA in this group	Average SDG score in this group	Rank of SDG score in this group	Rank difference (score-RCA) for window group
**2015**	8.A	Adults with an account at a bank or other financial institution or with a mobile-money-service provider (% of population aged 15 or over)	40	61	0.02	50.1	74	-13
**2015**	17.C	Statistical Performance Index (worst 0-100 best)	40	62	0.02	49.6	76	-14
**2015**	3.F	Age-standardized death rate due to cardiovascular disease, cancer, diabetes, or chronic respiratory disease in adults aged 30–70 years (%)	40	63	0.02	56.7	66	-3
**2015**	2.H	Prevalence of wasting in children under 5 years of age (%)	40	64	0.02	75.4	48	16
**2015**	10.B	Palma ratio	40	65	0.01	48.1	79	-14
**2015**	8.C	Fatal work-related accidents embodied in imports (per 100,000 population)	40	66	0.01	98.8	1	65
**2015**	11.B	Annual mean concentration of particulate matter of less than 2.5 microns in diameter (PM2.5) (μg/m³)	40	67	0.01	75.7	47	20
**2015**	12.G	Production-based SO2 emissions (kg/capita)	40	68	0.01	97.5	3	65
**2015**	16.I	Population who feel safe walking alone at night in the city or area where they live (%)	40	69	0.01	50.1	75	-6
**2015**	17.B	Government spending on health and education (% of GDP)	40	70	0.01	53.2	68	2
**2015**	14.A	Marine biodiversity threats embodied in imports (per million population)	40	71	0.01	90.3	19	52
**2015**	9.G	The Times Higher Education Universities Ranking: Average score of top 3 universities (worst 0-100 best)	40	72	0.00	26.6	93	-21
**2015**	2.A	Cereal yield (tonnes per hectare of harvested land)	40	73	0.00	48.3	78	-5
**2015**	12.C	Municipal solid waste (kg/capita/day)	40	74	0.00	77.6	44	30
**2015**	17.A	Corporate Tax Haven Score (best 0-100 worst)	40	75	0.00	90.9	17	58
**2015**	8.B	Adjusted GDP growth (%)	40	76	-0.01	63.2	61	15
**2015**	15.A	Terrestrial and freshwater biodiversity threats embodied in imports (per million population)	40	77	-0.02	88.6	25	52
**2015**	16.K	Exports of major conventional weapons (TIV constant million USD per 100,000 population)	40	78	-0.03	92.0	15	63
**2015**	13.B	CO2 emissions from fossil fuel combustion and cement production (tCO2/capita)	40	79	-0.03	78.2	42	37
**2015**	9.C	Logistics Performance Index: Quality of trade and transport-related infrastructure (worst 1-5 best)	40	80	-0.06	50.3	72	8
**2015**	8.G	Unemployment rate (% of total labor force, ages 15+)	40	81	-0.07	68.9	55	26
**2015**	2.F	Human Trophic Level (best 2-3 worst)	40	82	-0.07	41.2	84	-2
**2015**	16.E	Expropriations are lawful and adequately compensated (worst 0 - 1 best)	40	83	-0.09	29.7	91	-8
**2015**	15.E	Red List Index of species survival (worst 0-1 best)	40	84	-0.09	62.4	62	22
**2015**	7.D	Renewable energy share in total final energy consumption (%)	40	85	-0.10	21.1	94	-9
**2015**	5.C	Ratio of female-to-male labor force participation rate (%)	40	86	-0.10	58.9	65	21
**2015**	9.E	Expenditure on research and development (% of GDP)	40	87	-0.11	17.1	95	-8
**2015**	5.D	Seats held by women in national parliament (%)	40	88	-0.12	38.0	87	1

Year	SDG indicators	Indicator name	Window group	Rank of RCA in this group	Average RCA in this group	Average SDG score in this group	Rank of SDG score in this group	Rank difference (score-RCA) for window group
**2015**	16.C	Corruption Perceptions Index (worst 0-100 best)	40	89	-0.13	36.7	88	1
**2015**	15.B	Mean area that is protected in freshwater sites important to biodiversity (%)	40	90	-0.14	41.9	83	7
**2015**	6.A	Freshwater withdrawal (% of available freshwater resources)	40	91	-0.16	69.2	53	38
**2015**	2.B	Prevalence of obesity, BMI ≥ 30 (% of adult population)	40	92	-0.16	45.6	81	11
**2015**	16.H	Press Freedom Index (worst 0-100 best)	40	93	-0.17	49.5	77	16
**2015**	9.A	Articles published in academic journals (per 1,000 population)	40	94	-0.23	27.0	92	2
**2015**	15.C	Mean area that is protected in terrestrial sites important to biodiversity (%)	40	95	-0.33	32.6	90	5
**2015**	6.D	Anthropogenic wastewater that receives treatment (%)	40	96	-0.53	11.2	96	0

Supplementary Table 8 | The RCA and SDG scores of SDG indicators in the 80th window group of countries in 2015. Note: The SDG indicators listed by the rank order of revealed comparative advantage (RCA). The RCA results are generated by the moving-window approach with window size of 50 (see Methods).

Year	SDG indicators	Indicator name	Window group	Rank of RCA in this group	Average RCA in this group	Average SDG score in this group	Rank of SDG score in this group	Rank difference (score-RCA) for window group
**2015**	14.E	Fish caught from overexploited or collapsed stocks (% of total catch)	80	1	0.83	71.2	39	-38
**2015**	14.B	Ocean Health Index: Clean Waters score (worst 0-100 best)	80	2	0.48	40.0	80	-78
**2015**	2.C	Exports of hazardous pesticides (tonnes per million population)	80	3	0.48	90.1	7	-4
**2015**	16.B	Children involved in child labor (% of population aged 5 to 14)	80	4	0.47	70.0	43	-39
**2015**	14.D	Fish caught that are then discarded (%)	80	5	0.43	64.4	51	-46
**2015**	14.F	Fish caught by trawling or dredging (%)	80	6	0.42	78.6	29	-23
**2015**	12.B	Exports of plastic waste (kg/capita)	80	7	0.37	94.1	4	3
**2015**	4.B	Literacy rate (% of population aged 15 to 24)	80	8	0.36	85.2	15	-7
**2015**	2.F	Human Trophic Level (best 2-3 worst)	80	9	0.33	52.9	64	-55
**2015**	11.D	Satisfaction with public transport (%)	80	10	0.29	65.3	49	-39
**2015**	3.C	New HIV infections (per 1,000 uninfected population)	80	11	0.28	83.7	18	-7
**2015**	12.A	Electronic waste (kg/capita)	80	12	0.25	73.3	35	-23
**2015**	1.B	Poverty headcount ratio at $2.15/day (2017 PPP, %)	80	13	0.21	85.8	12	1
**2015**	13.C	CO2 emissions embodied in imports (tCO2/capita)	80	14	0.17	75.8	30	-16
**2015**	12.D	Nitrogen emissions embodied in imports (kg/capita)	80	15	0.16	74.6	32	-17
**2015**	8.F	Victims of modern slavery (per 1,000 population)	80	16	0.16	73.8	34	-18
**2015**	12.C	Municipal solid waste (kg/capita/day)	80	17	0.16	80.5	24	-7
**2015**	12.F	SO2 emissions embodied in imports (kg/capita)	80	18	0.16	90.7	5	13
**2015**	8.D	Victims of modern slavery embodied in imports (per 100,000 population)	80	19	0.16	86.7	10	9
**2015**	5.B	Demand for family planning satisfied by modern methods (% of females aged 15 to 49)	80	20	0.15	57.7	59	-39
**2015**	8.C	Fatal work-related accidents embodied in imports (per 100,000 population)	80	21	0.15	98.7	1	20
**2015**	11.C	Proportion of urban population living in slums (%)	80	22	0.15	69.6	44	-22
**2015**	12.E	Production-based nitrogen emissions (kg/capita)	80	23	0.14	67.6	47	-24
**2015**	11.A	Access to improved water source, piped (% of urban population)	80	24	0.14	79.9	26	-2
**2015**	3.N	Surviving infants who received 2 WHO-recommended vaccines (%)	80	25	0.14	81.4	22	3
**2015**	4.C	Net primary enrollment rate (%)	80	26	0.14	85.5	13	13
**2015**	12.G	Production-based SO2 emissions (kg/capita)	80	27	0.14	96.8	2	25
**2015**	13.A	CO2/emissions embodied in fossil fuel exports (kg/capita)	80	28	0.14	86.3	11	17
**2015**	16.K	Exports of major conventional weapons (TIV constant million USD per 100,000 population)	80	29	0.14	96.2	3	26
**2015**	5.A	Ratio of female-to-male mean years of education received (%)	80	30	0.14	82.7	20	10

Year	SDG indicators	Indicator name	Window group	Rank of RCA in this group	Average RCA in this group	Average SDG score in this group	Rank of SDG score in this group	Rank difference (score-RCA) for window group
**2015**	16.G	Access to and affordability of justice (worst 0–1 best)	80	31	0.13	56.8	61	-30
**2015**	2.G	Prevalence of undernourishment (%)	80	32	0.13	81.4	21	11
**2015**	7.C	Population with access to electricity (%)	80	33	0.13	83.8	17	16
**2015**	16.J	Birth registrations with civil authority (% of children under age 5)	80	34	0.13	85.3	14	20
**2015**	3.A	Births attended by skilled health personnel (%)	80	35	0.12	83.0	19	16
**2015**	6.E	Population using at least basic drinking water services (%)	80	36	0.12	80.9	23	13
**2015**	14.A	Marine biodiversity threats embodied in imports (per million population)	80	37	0.12	89.0	9	28
**2015**	3.E	Maternal mortality rate (per 100,000 live births)	80	38	0.12	84.7	16	22
**2015**	6.C	Scarce water consumption embodied in imports (m3 H2O eq/capita)	80	39	0.12	79.2	27	12
**2015**	17.A	Corporate Tax Haven Score (best 0-100 worst)	80	40	0.11	90.4	6	34
**2015**	16.F	Homicides (per 100,000 population)	80	41	0.11	75.6	31	10
**2015**	16.I	Population who feel safe walking alone at night in the city or area where they live (%)	80	42	0.11	49.7	68	-26
**2015**	15.A	Terrestrial and freshwater biodiversity threats embodied in imports (per million population)	80	43	0.11	89.6	8	35
**2015**	15.D	Permanent deforestation (% of forest area, 3-year average)	80	44	0.11	80.4	25	19
**2015**	14.C	Mean area that is protected in marine sites important to biodiversity (%)	80	45	0.10	33.8	88	-43
**2015**	4.D	Lower secondary completion rate (%)	80	46	0.10	70.2	42	4
**2015**	3.L	Mortality rate, under-5 (per 1,000 live births)	80	47	0.09	78.8	28	19
**2015**	6.B	Population using at least basic sanitation services (%)	80	48	0.09	72.8	36	12
**2015**	8.G	Unemployment rate (% of total labor force, ages 15+)	80	49	0.08	71.8	37	12
**2015**	16.D	Unsentenced detainees (% of prison population)	80	50	0.08	52.7	65	-15
**2015**	16.A	Timeliness of administrative proceedings (worst 0 - 1 best)	80	51	0.08	40.5	78	-27
**2015**	7.B	CO2 emissions from fuel combustion per total electricity output (MtCO2 TWh)	80	52	0.07	70.8	40	12
**2015**	2.B	Prevalence of obesity, BMI ≥ 30 (% of adult population)	80	53	0.07	51.9	66	-13
**2015**	7.A	Population with access to clean fuels and technology for cooking (%)	80	54	0.06	64.5	50	4
**2015**	8.B	Adjusted GDP growth (%)	80	55	0.06	59.7	58	-3
**2015**	10.A	Gini coefficient	80	56	0.05	61.5	54	2
**2015**	4.A	Participation rate in pre-primary organized learning (% of children aged 4 to 6)	80	57	0.05	53.5	63	-6
**2015**	2.H	Prevalence of wasting in children under 5 years of age (%)	80	58	0.04	67.8	45	13
**2015**	3.I	Subjective well-being (average ladder score, worst 0-10 best)	80	59	0.04	44.9	71	-12
**2015**	3.B	Adolescent fertility rate (births per 1,000 females aged 15 to 19)	80	60	0.03	60.7	56	4
**2015**	1.A	Poverty headcount ratio at $3.65/day (2017 PPP, %)	80	61	0.03	56.4	62	-1
**2015**	9.F	Rural population with access to all-season roads (%)	80	62	0.03	67.8	46	16
**2015**	13.B	CO2 emissions from fossil fuel combustion and cement production (tCO2/capita)	80	63	0.03	74.0	33	30

Year	SDG indicators	Indicator name	Window group	Rank of RCA in this group	Average RCA in this group	Average SDG score in this group	Rank of SDG score in this group	Rank difference (score-RCA) for window group
**2015**	8.E	Fundamental labor rights are effectively guaranteed (worst 0–1 best)	80	64	0.02	41.0	77	-13
**2015**	3.H	Age-standardized death rate attributable to household air pollution and ambient air pollution (per 100,000 population)	80	65	0.02	70.3	41	24
**2015**	3.G	Neonatal mortality rate (per 1,000 live births)	80	66	0.01	63.8	52	14
**2015**	2.E	Prevalence of stunting in children under 5 years of age (%)	80	67	0.01	62.7	53	14
**2015**	5.D	Seats held by women in national parliament (%)	80	68	-0.01	38.0	84	-16
**2015**	15.E	Red List Index of species survival (worst 0-1 best)	80	69	-0.02	60.4	57	12
**2015**	2.A	Cereal yield (tonnes per hectare of harvested land)	80	70	-0.02	42.0	74	-4
**2015**	3.D	Life expectancy at birth (years)	80	71	-0.02	56.8	60	11
**2015**	2.D	Sustainable Nitrogen Management Index (best 0-1.41 worst)	80	72	-0.03	27.2	91	-19
**2015**	3.M	Universal health coverage (UHC) index of service coverage (worst 0-100 best)	80	73	-0.03	36.0	85	-12
**2015**	16.E	Expropriations are lawful and adequately compensated (worst 0 - 1 best)	80	74	-0.04	27.6	90	-16
**2015**	3.J	Incidence of tuberculosis (per 100,000 population)	80	75	-0.04	71.5	38	37
**2015**	9.D	Mobile broadband subscriptions (per 100 population)	80	76	-0.05	41.3	76	0
**2015**	17.B	Government spending on health and education (% of GDP)	80	77	-0.05	44.4	72	5
**2015**	9.C	Logistics Performance Index: Quality of trade and transport-related infrastructure (worst 1-5 best)	80	78	-0.06	44.1	73	5
**2015**	8.A	Adults with an account at a bank or other financial institution or with a mobile-money-service provider (% of population aged 15 or over)	80	79	-0.07	40.2	79	0
**2015**	3.K	Traffic deaths (per 100,000 population)	80	80	-0.07	47.1	69	11
**2015**	9.B	Population using the internet (%)	80	81	-0.08	38.9	83	-2
**2015**	15.C	Mean area that is protected in terrestrial sites important to biodiversity (%)	80	82	-0.08	39.0	82	0
**2015**	11.B	Annual mean concentration of particulate matter of less than 2.5 microns in diameter (PM2.5) (μg/m³)	80	83	-0.08	61.3	55	28
**2015**	15.B	Mean area that is protected in freshwater sites important to biodiversity (%)	80	84	-0.08	39.6	81	3
**2015**	6.A	Freshwater withdrawal (% of available freshwater resources)	80	85	-0.09	66.5	48	37
**2015**	16.C	Corruption Perceptions Index (worst 0-100 best)	80	86	-0.10	33.3	89	-3
**2015**	5.C	Ratio of female-to-male labor force participation rate (%)	80	87	-0.11	51.6	67	20
**2015**	16.H	Press Freedom Index (worst 0-100 best)	80	88	-0.13	46.5	70	18
**2015**	10.B	Palma ratio	80	89	-0.14	35.9	86	3
**2015**	3.F	Age-standardized death rate due to cardiovascular disease, cancer, diabetes, or chronic respiratory disease in adults aged 30–70 years (%)	80	90	-0.16	41.8	75	15
**2015**	17.C	Statistical Performance Index (worst 0-100 best)	80	91	-0.17	35.8	87	4
**2015**	7.D	Renewable energy share in total final energy consumption (%)	80	92	-0.23	15.5	92	0
**2015**	6.D	Anthropogenic wastewater that receives treatment (%)	80	93	-0.47	10.0	95	-2
**2015**	9.E	Expenditure on research and development (% of GDP)	80	94	-0.49	8.3	96	-2

Year	SDG indicators	Indicator name	Window group	Rank of RCA in this group	Average RCA in this group	Average SDG score in this group	Rank of SDG score in this group	Rank difference (score-RCA) for window group
**2015**	9.G	The Times Higher Education Universities Ranking: Average score of top 3 universities (worst 0-100 best)	80	95	-0.53	10.8	94	1
**2015**	9.A	Articles published in academic journals (per 1,000 population)	80	96	-0.54	13.9	93	3

Supplementary Table 9 | The RCA and SDG scores of SDG indicators in the 117th window group of countries in 2015. Note: The SDG indicators listed by the rank order of revealed comparative advantage (RCA). The RCA results are generated by the moving-window approach with window size of 50 (see Methods).

Year	SDG indicators	Indicator name	Window group	Rank of RCA in this group	Average RCA in this group	Average SDG score in this group	Rank of SDG score in this group	Rank difference (score-RCA) for window group
**2015**	14.E	Fish caught from overexploited or collapsed stocks (% of total catch)	117	1	1.41	78.4	23	-22
**2015**	2.F	Human Trophic Level (best 2-3 worst)	117	2	1.12	69.5	28	-26
**2015**	14.D	Fish caught that are then discarded (%)	117	3	1.04	78.3	24	-21
**2015**	2.B	Prevalence of obesity, BMI ≥ 30 (% of adult population)	117	4	0.92	77.6	25	-21
**2015**	12.A	Electronic waste (kg/capita)	117	5	0.91	93.2	15	-10
**2015**	2.C	Exports of hazardous pesticides (tonnes per million population)	117	6	0.85	95.9	12	-6
**2015**	14.F	Fish caught by trawling or dredging (%)	117	7	0.83	84.2	20	-13
**2015**	13.C	CO2 emissions embodied in imports (tCO2/capita)	117	8	0.82	94.4	13	-5
**2015**	12.E	Production-based nitrogen emissions (kg/capita)	117	9	0.79	85.7	19	-10
**2015**	12.D	Nitrogen emissions embodied in imports (kg/capita)	117	10	0.75	90.2	16	-6
**2015**	12.B	Exports of plastic waste (kg/capita)	117	11	0.70	99.0	5	6
**2015**	13.B	CO2 emissions from fossil fuel combustion and cement production (tCO2/capita)	117	12	0.66	97.0	9	3
**2015**	6.C	Scarce water consumption embodied in imports (m3 H2O eq/capita)	117	13	0.65	94.2	14	-1
**2015**	8.D	Victims of modern slavery embodied in imports (per 100,000 population)	117	14	0.61	96.7	10	4
**2015**	12.C	Municipal solid waste (kg/capita/day)	117	15	0.60	89.8	17	-2
**2015**	13.A	CO2/emissions embodied in fossil fuel exports (kg/capita)	117	16	0.60	99.5	3	13
**2015**	12.F	SO2 emissions embodied in imports (kg/capita)	117	17	0.56	98.1	6	11
**2015**	3.C	New HIV infections (per 1,000 uninfected population)	117	18	0.54	81.6	21	-3
**2015**	15.A	Terrestrial and freshwater biodiversity threats embodied in imports (per million population)	117	19	0.49	97.9	7	12
**2015**	17.A	Corporate Tax Haven Score (best 0-100 worst)	117	20	0.47	97.0	8	12

Year	SDG indicators	Indicator name	Window group	Rank of RCA in this group	Average RCA in this group	Average SDG score in this group	Rank of SDG score in this group	Rank difference (score-RCA) for window group
**2015**	14.A	Marine biodiversity threats embodied in imports (per million population)	117	21	0.45	96.6	11	10
**2015**	16.K	Exports of major conventional weapons (TIV constant million USD per 100,000 population)	117	22	0.45	100.0	1	21
**2015**	6.A	Freshwater withdrawal (% of available freshwater resources)	117	23	0.45	86.0	18	5
**2015**	12.G	Production-based SO2 emissions (kg/capita)	117	24	0.45	99.3	4	20
**2015**	8.C	Fatal work-related accidents embodied in imports (per 100,000 population)	117	25	0.44	99.7	2	23
**2015**	5.C	Ratio of female-to-male labor force participation rate (%)	117	26	0.38	66.3	29	-3
**2015**	15.D	Permanent deforestation (% of forest area, 3-year average)	117	27	0.35	78.8	22	5
**2015**	15.E	Red List Index of species survival (worst 0-1 best)	117	28	0.34	66.2	30	-2
**2015**	8.G	Unemployment rate (% of total labor force, ages 15+)	117	29	0.33	72.7	27	2
**2015**	16.F	Homicides (per 100,000 population)	117	30	0.32	75.8	26	4
**2015**	10.A	Gini coefficient	117	31	0.28	60.9	34	-3
**2015**	14.C	Mean area that is protected in marine sites important to biodiversity (%)	117	32	0.23	32.6	68	-36
**2015**	14.B	Ocean Health Index: Clean Waters score (worst 0-100 best)	117	33	0.19	26.5	78	-45
**2015**	16.G	Access to and affordability of justice (worst 0–1 best)	117	34	0.17	49.9	45	-11
**2015**	15.B	Mean area that is protected in freshwater sites important to biodiversity (%)	117	35	0.17	40.4	54	-19
**2015**	15.C	Mean area that is protected in terrestrial sites important to biodiversity (%)	117	36	0.15	40.2	55	-19
**2015**	5.D	Seats held by women in national parliament (%)	117	37	0.14	35.8	61	-24
**2015**	7.B	CO2 emissions from fuel combustion per total electricity output (MtCO2 TWh)	117	38	0.13	61.8	32	6
**2015**	8.F	Victims of modern slavery (per 1,000 population)	117	39	0.13	59.6	35	4
**2015**	16.H	Press Freedom Index (worst 0-100 best)	117	40	0.10	47.5	49	-9
**2015**	16.A	Timeliness of administrative proceedings (worst 0 - 1 best)	117	41	0.09	34.8	65	-24
**2015**	11.A	Access to improved water source, piped (% of urban population)	117	42	0.08	62.2	31	11
**2015**	11.B	Annual mean concentration of particulate matter of less than 2.5 microns in diameter (PM2.5) (μg/m³)	117	43	0.07	58.1	37	6
**2015**	16.I	Population who feel safe walking alone at night in the city or area where they live (%)	117	44	0.06	38.0	60	-16
**2015**	8.E	Fundamental labor rights are effectively guaranteed (worst 0–1 best)	117	45	0.04	35.8	62	-17
**2015**	7.D	Renewable energy share in total final energy consumption (%)	117	46	0.02	17.4	86	-40
**2015**	16.B	Children involved in child labor (% of population aged 5 to 14)	117	47	0.00	40.1	57	-10
**2015**	16.D	Unsentenced detainees (% of prison population)	117	48	-0.01	41.3	52	-4
**2015**	2.D	Sustainable Nitrogen Management Index (best 0-1.41 worst)	117	49	-0.02	23.5	80	-31
**2015**	8.B	Adjusted GDP growth (%)	117	50	-0.02	46.5	50	0

Year	SDG indicators	Indicator name	Window group	Rank of RCA in this group	Average RCA in this group	Average SDG score in this group	Rank of SDG score in this group	Rank difference (score-RCA) for window group
**2015**	4.C	Net primary enrollment rate (%)	117	51	-0.02	61.7	33	18
**2015**	3.J	Incidence of tuberculosis (per 100,000 population)	117	52	-0.03	58.5	36	16
**2015**	4.B	Literacy rate (% of population aged 15 to 24)	117	53	-0.04	51.0	44	9
**2015**	10.B	Palma ratio	117	54	-0.08	32.8	67	-13
**2015**	1.B	Poverty headcount ratio at $2.15/day (2017 PPP, %)	117	55	-0.09	53.5	40	15
**2015**	2.H	Prevalence of wasting in children under 5 years of age (%)	117	56	-0.09	49.8	46	10
**2015**	3.H	Age-standardized death rate attributable to household air pollution and ambient air pollution (per 100,000 population)	117	57	-0.09	51.6	43	14
**2015**	2.G	Prevalence of undernourishment (%)	117	58	-0.09	56.2	38	20
**2015**	3.N	Surviving infants who received 2 WHO-recommended vaccines (%)	117	59	-0.12	53.3	41	18
**2015**	16.J	Birth registrations with civil authority (% of children under age 5)	117	60	-0.13	54.6	39	21
**2015**	17.B	Government spending on health and education (% of GDP)	117	61	-0.15	32.6	69	-8
**2015**	3.A	Births attended by skilled health personnel (%)	117	62	-0.16	52.2	42	20
**2015**	5.B	Demand for family planning satisfied by modern methods (% of females aged 15 to 49)	117	63	-0.18	35.1	64	-1
**2015**	11.D	Satisfaction with public transport (%)	117	64	-0.20	34.1	66	-2
**2015**	5.A	Ratio of female-to-male mean years of education received (%)	117	65	-0.21	48.2	48	17
**2015**	3.F	Age-standardized death rate due to cardiovascular disease, cancer, diabetes, or chronic respiratory disease in adults aged 30–70 years (%)	117	66	-0.22	31.7	70	-4
**2015**	11.C	Proportion of urban population living in slums (%)	117	67	-0.24	39.8	58	9
**2015**	9.C	Logistics Performance Index: Quality of trade and transport-related infrastructure (worst 1-5 best)	117	68	-0.24	29.8	76	-8
**2015**	3.E	Maternal mortality rate (per 100,000 live births)	117	69	-0.24	48.8	47	22
**2015**	17.C	Statistical Performance Index (worst 0-100 best)	117	70	-0.27	26.7	77	-7
**2015**	9.F	Rural population with access to all-season roads (%)	117	71	-0.27	40.6	53	18
**2015**	3.L	Mortality rate, under-5 (per 1,000 live births)	117	72	-0.27	44.1	51	21
**2015**	2.E	Prevalence of stunting in children under 5 years of age (%)	117	73	-0.30	35.5	63	10
**2015**	16.E	Expropriations are lawful and adequately compensated (worst 0 - 1 best)	117	74	-0.32	17.3	87	-13
**2015**	16.C	Corruption Perceptions Index (worst 0-100 best)	117	75	-0.33	21.2	83	-8
**2015**	6.E	Population using at least basic drinking water services (%)	117	76	-0.34	40.1	56	20
**2015**	3.B	Adolescent fertility rate (births per 1,000 females aged 15 to 19)	117	77	-0.37	31.0	72	5
**2015**	3.D	Life expectancy at birth (years)	117	78	-0.37	30.3	73	5
**2015**	3.K	Traffic deaths (per 100,000 population)	117	79	-0.38	26.3	79	0
**2015**	7.C	Population with access to electricity (%)	117	80	-0.38	38.2	59	21
**2015**	2.A	Cereal yield (tonnes per hectare of harvested land)	117	81	-0.38	22.0	81	0
**2015**	3.I	Subjective well-being (average ladder score, worst 0-10 best)	117	82	-0.39	21.3	82	0
**2015**	4.D	Lower secondary completion rate (%)	117	83	-0.43	31.2	71	12

Year	SDG indicators	Indicator name	Window group	Rank of RCA in this group	Average RCA in this group	Average SDG score in this group	Rank of SDG score in this group	Rank difference (score-RCA) for window group
**2015**	3.G	Neonatal mortality rate (per 1,000 live births)	117	84	-0.44	29.8	75	9
**2015**	6.B	Population using at least basic sanitation services (%)	117	85	-0.46	30.3	74	11
**2015**	8.A	Adults with an account at a bank or other financial institution or with a mobile-money-service provider (% of population aged 15 or over)	117	86	-0.54	17.0	88	-2
**2015**	4.A	Participation rate in pre-primary organized learning (% of children aged 4 to 6)	117	87	-0.54	19.8	84	3
**2015**	9.D	Mobile broadband subscriptions (per 100 population)	117	88	-0.62	14.3	89	-1
**2015**	9.E	Expenditure on research and development (% of GDP)	117	89	-0.62	5.4	93	-4
**2015**	7.A	Population with access to clean fuels and technology for cooking (%)	117	90	-0.62	19.1	85	5
**2015**	9.B	Population using the internet (%)	117	91	-0.65	12.7	90	1
**2015**	3.M	Universal health coverage (UHC) index of service coverage (worst 0-100 best)	117	92	-0.71	9.4	92	0
**2015**	1.A	Poverty headcount ratio at $3.65/day (2017 PPP, %)	117	93	-0.75	11.9	91	2
**2015**	6.D	Anthropogenic wastewater that receives treatment (%)	117	94	-0.82	2.8	94	0
**2015**	9.G	The Times Higher Education Universities Ranking: Average score of top 3 universities (worst 0-100 best)	117	95	-0.88	2.3	96	-1
**2015**	9.A	Articles published in academic journals (per 1,000 population)	117	96	-0.91	2.4	95	1

Supplementary Table 10 | The RCA and SDG scores of SDG indicators in the 1st window group of countries in 2022. Note: The SDG indicators listed by the rank order of revealed comparative advantage (RCA). The RCA results are generated by the moving-window approach with window size of 50 (see Methods).

Year	SDG indicators	Indicator name	Window group	Rank of RCA in this group	Average RCA in this group	Average SDG score in this group	Rank of SDG score in this group	Rank difference (score-RCA) for window group
**2022**	6.D	Anthropogenic wastewater that receives treatment (%)	1	1	1.01	53.0	89	-88
**2022**	9.E	Expenditure on research and development (% of GDP)	1	2	0.93	44.4	91	-89
**2022**	9.A	Articles published in academic journals (per 1,000 population)	1	3	0.70	82.9	47	-44
**2022**	16.E	Expropriations are lawful and adequately compensated (worst 0 - 1 best)	1	4	0.67	56.8	86	-82
**2022**	14.C	Mean area that is protected in marine sites important to biodiversity (%)	1	5	0.60	66.4	74	-69
**2022**	16.B	Children involved in child labor (% of population aged 5 to 14)	1	6	0.53	96.5	18	-12
**2022**	9.G	The Times Higher Education Universities Ranking: Average score of top 3 universities (worst 0-100 best)	1	7	0.48	74.5	61	-54
**2022**	8.E	Fundamental labor rights are effectively guaranteed (worst 0–1 best)	1	8	0.43	72.1	68	-60
**2022**	14.B	Ocean Health Index: Clean Waters score (worst 0-100 best)	1	9	0.39	56.8	87	-78
**2022**	16.A	Timeliness of administrative proceedings (worst 0 - 1 best)	1	10	0.38	65.3	76	-66
**2022**	16.H	Press Freedom Index (worst 0-100 best)	1	11	0.37	66.4	73	-62
**2022**	7.D	Renewable energy share in total final energy consumption (%)	1	12	0.37	38.0	93	-81
**2022**	4.A	Participation rate in pre-primary organized learning (% of children aged 4 to 6)	1	13	0.36	90.3	34	-21
**2022**	10.B	Palma ratio	1	14	0.35	78.8	51	-37
**2022**	1.A	Poverty headcount ratio at $3.65/day (2017 PPP, %)	1	15	0.34	98.0	12	3
**2022**	8.A	Adults with an account at a bank or other financial institution or with a mobile-money-service provider (% of population aged 15 or over)	1	16	0.33	90.9	33	-17
**2022**	16.C	Corruption Perceptions Index (worst 0-100 best)	1	17	0.30	62.5	79	-62
**2022**	3.I	Subjective well-being (average ladder score, worst 0-10 best)	1	18	0.27	73.4	64	-46
**2022**	3.M	Universal health coverage (UHC) index of service coverage (worst 0-100 best)	1	19	0.26	66.8	72	-53
**2022**	3.K	Traffic deaths (per 100,000 population)	1	20	0.26	84.8	43	-23
**2022**	16.G	Access to and affordability of justice (worst 0–1 best)	1	21	0.26	84.7	44	-23
**2022**	9.C	Logistics Performance Index: Quality of trade and transport-related infrastructure (worst 1-5 best)	1	22	0.25	73.6	62	-40
**2022**	2.A	Cereal yield (tonnes per hectare of harvested land)	1	23	0.24	72.1	67	-44
**2022**	11.C	Proportion of urban population living in slums (%)	1	24	0.21	98.3	9	15
**2022**	7.A	Population with access to clean fuels and technology for cooking (%)	1	25	0.19	96.5	19	6

Year	SDG indicators	Indicator name	Window group	Rank of RCA in this group	Average RCA in this group	Average SDG score in this group	Rank of SDG score in this group	Rank difference (score-RCA) for window group
**2022**	16.D	Unsentenced detainees (% of prison population)	1	26	0.19	75.5	58	-32
**2022**	4.B	Literacy rate (% of population aged 15 to 24)	1	27	0.18	99.2	4	23
**2022**	14.E	Fish caught from overexploited or collapsed stocks (% of total catch)	1	28	0.18	61.4	81	-53
**2022**	17.B	Government spending on health and education (% of GDP)	1	29	0.17	77.6	53	-24
**2022**	3.C	New HIV infections (per 1,000 uninfected population)	1	30	0.15	98.4	8	22
**2022**	2.C	Exports of hazardous pesticides (tonnes per million population)	1	31	0.14	89.8	36	-5
**2022**	15.B	Mean area that is protected in freshwater sites important to biodiversity (%)	1	32	0.13	65.8	75	-43
**2022**	9.F	Rural population with access to all-season roads (%)	1	33	0.13	96.6	17	16
**2022**	3.F	Age-standardized death rate due to cardiovascular disease, cancer, diabetes, or chronic respiratory disease in adults aged 30–70 years (%)	1	34	0.13	76.6	56	-22
**2022**	4.D	Lower secondary completion rate (%)	1	35	0.12	95.6	24	11
**2022**	16.I	Population who feel safe walking alone at night in the city or area where they live (%)	1	36	0.12	68.1	71	-35
**2022**	17.C	Statistical Performance Index (worst 0-100 best)	1	37	0.12	82.9	46	-9
**2022**	3.B	Adolescent fertility rate (births per 1,000 females aged 15 to 19)	1	38	0.12	91.5	30	8
**2022**	3.D	Life expectancy at birth (years)	1	39	0.11	87.4	39	0
**2022**	3.G	Neonatal mortality rate (per 1,000 live births)	1	40	0.11	95.1	25	15
**2022**	15.C	Mean area that is protected in terrestrial sites important to biodiversity (%)	1	41	0.11	63.9	78	-37
**2022**	9.D	Mobile broadband subscriptions (per 100 population)	1	42	0.11	93.9	28	14
**2022**	10.A	Gini coefficient	1	43	0.10	84.9	42	1
**2022**	9.B	Population using the internet (%)	1	44	0.10	87.2	40	4
**2022**	2.G	Prevalence of undernourishment (%)	1	45	0.09	99.0	5	40
**2022**	11.A	Access to improved water source, piped (% of urban population)	1	46	0.08	97.4	13	33
**2022**	5.B	Demand for family planning satisfied by modern methods (% of females aged 15 to 49)	1	47	0.08	72.4	66	-19
**2022**	6.B	Population using at least basic sanitation services (%)	1	48	0.08	96.5	20	28
**2022**	1.B	Poverty headcount ratio at $2.15/day (2017 PPP, %)	1	49	0.08	99.4	3	46
**2022**	2.E	Prevalence of stunting in children under 5 years of age (%)	1	50	0.08	92.2	29	21
**2022**	8.F	Victims of modern slavery (per 1,000 population)	1	51	0.07	86.0	41	10
**2022**	15.D	Permanent deforestation (% of forest area, 3-year average)	1	52	0.07	96.9	15	37
**2022**	16.F	Homicides (per 100,000 population)	1	53	0.05	94.8	26	27
**2022**	11.B	Annual mean concentration of particulate matter of less than 2.5 microns in diameter (PM2.5) (μg/m³)	1	54	0.03	91.0	32	22
**2022**	16.J	Birth registrations with civil authority (% of children under age 5)	1	55	0.03	99.8	2	53
**2022**	8.B	Adjusted GDP growth (%)	1	56	0.03	75.5	60	-4
**2022**	2.H	Prevalence of wasting in children under 5 years of age (%)	1	57	0.03	90.1	35	22

Year	SDG indicators	Indicator name	Window group	Rank of RCA in this group	Average RCA in this group	Average SDG score in this group	Rank of SDG score in this group	Rank difference (score-RCA) for window group
**2022**	3.H	Age-standardized death rate attributable to household air pollution and ambient air pollution (per 100,000 population)	1	58	0.03	91.4	31	27
**2022**	5.D	Seats held by women in national parliament (%)	1	59	0.02	61.2	82	-23
**2022**	6.E	Population using at least basic drinking water services (%)	1	60	0.02	98.1	11	49
**2022**	2.D	Sustainable Nitrogen Management Index (best 0-1.41 worst)	1	61	0.02	35.6	95	-34
**2022**	14.F	Fish caught by trawling or dredging (%)	1	62	0.01	72.9	65	-3
**2022**	3.N	Surviving infants who received 2 WHO-recommended vaccines (%)	1	63	0.00	83.8	45	18
**2022**	3.L	Mortality rate, under-5 (per 1,000 live births)	1	64	0.00	98.1	10	54
**2022**	5.A	Ratio of female-to-male mean years of education received (%)	1	65	0.00	97.1	14	51
**2022**	7.C	Population with access to electricity (%)	1	66	0.00	100.0	1	65
**2022**	3.E	Maternal mortality rate (per 100,000 live births)	1	67	-0.01	98.9	6	61
**2022**	4.C	Net primary enrollment rate (%)	1	68	-0.01	95.8	23	45
**2022**	3.A	Births attended by skilled health personnel (%)	1	69	-0.01	98.8	7	62
**2022**	14.D	Fish caught that are then discarded (%)	1	70	-0.02	58.0	85	-15
**2022**	3.J	Incidence of tuberculosis (per 100,000 population)	1	71	-0.02	96.2	21	50
**2022**	13.A	CO2/emissions embodied in fossil fuel exports (kg/capita)	1	72	-0.04	94.0	27	45
**2022**	5.C	Ratio of female-to-male labor force participation rate (%)	1	73	-0.04	75.5	59	14
**2022**	15.E	Red List Index of species survival (worst 0-1 best)	1	74	-0.05	73.4	63	11
**2022**	11.D	Satisfaction with public transport (%)	1	75	-0.06	62.2	80	-5
**2022**	6.A	Freshwater withdrawal (% of available freshwater resources)	1	76	-0.06	87.6	38	38
**2022**	7.B	CO2 emissions from fuel combustion per total electricity output (MtCO2 TWh)	1	77	-0.07	78.8	50	27
**2022**	8.G	Unemployment rate (% of total labor force, ages 15+)	1	78	-0.09	79.8	49	29
**2022**	12.G	Production-based SO2 emissions (kg/capita)	1	79	-0.10	96.9	16	63
**2022**	8.C	Fatal work-related accidents embodied in imports (per 100,000 population)	1	80	-0.11	96.2	22	58
**2022**	14.A	Marine biodiversity threats embodied in imports (per million population)	1	81	-0.13	88.9	37	44
**2022**	12.F	SO2 emissions embodied in imports (kg/capita)	1	82	-0.18	80.9	48	34
**2022**	8.D	Victims of modern slavery embodied in imports (per 100,000 population)	1	83	-0.19	75.9	57	26
**2022**	6.C	Scarce water consumption embodied in imports (m3 H2O eq/capita)	1	84	-0.20	71.5	69	15
**2022**	15.A	Terrestrial and freshwater biodiversity threats embodied in imports (per million population)	1	85	-0.25	77.4	54	31
**2022**	17.A	Corporate Tax Haven Score (best 0-100 worst)	1	86	-0.25	77.7	52	34
**2022**	12.D	Nitrogen emissions embodied in imports (kg/capita)	1	87	-0.27	59.0	84	3
**2022**	13.B	CO2 emissions from fossil fuel combustion and cement production (tCO2/capita)	1	88	-0.27	68.5	70	18
**2022**	12.E	Production-based nitrogen emissions (kg/capita)	1	89	-0.27	54.5	88	1
**2022**	12.C	Municipal solid waste (kg/capita/day)	1	90	-0.27	64.6	77	13

Year	SDG indicators	Indicator name	Window group	Rank of RCA in this group	Average RCA in this group	Average SDG score in this group	Rank of SDG score in this group	Rank difference (score-RCA) for window group
**2022**	16.K	Exports of major conventional weapons (TIV constant million USD per 100,000 population)	1	91	-0.29	77.4	55	36
**2022**	12.B	Exports of plastic waste (kg/capita)	1	92	-0.34	60.5	83	9
**2022**	13.C	CO2 emissions embodied in imports (tCO2/capita)	1	93	-0.37	51.1	90	3
**2022**	2.B	Prevalence of obesity, BMI ≥ 30 (% of adult population)	1	94	-0.38	38.5	92	2
**2022**	12.A	Electronic waste (kg/capita)	1	95	-0.50	35.7	94	1
**2022**	2.F	Human Trophic Level (best 2-3 worst)	1	96	-0.65	17.1	96	0

Supplementary Table 11 | The RCA and SDG scores of SDG indicators in the 40th window group of countries in 2022. Note: The SDG indicators listed by the rank order of revealed comparative advantage (RCA). The RCA results are generated by the moving-window approach with window size of 50 (see Methods).

Year	SDG indicators	Indicator name	Window group	Rank of RCA in this group	Average RCA in this group	Average SDG score in this group	Rank of SDG score in this group	Rank difference (score-RCA) for window group
**2022**	14.E	Fish caught from overexploited or collapsed stocks (% of total catch)	40	1	0.56	70.1	58	-57
**2022**	16.B	Children involved in child labor (% of population aged 5 to 14)	40	2	0.54	84.2	32	-30
**2022**	1.A	Poverty headcount ratio at $3.65/day (2017 PPP, %)	40	3	0.39	88.9	26	-23
**2022**	14.B	Ocean Health Index: Clean Waters score (worst 0-100 best)	40	4	0.32	46.8	80	-76
**2022**	3.C	New HIV infections (per 1,000 uninfected population)	40	5	0.30	97.7	4	1
**2022**	2.C	Exports of hazardous pesticides (tonnes per million population)	40	6	0.29	89.3	25	-19
**2022**	4.B	Literacy rate (% of population aged 15 to 24)	40	7	0.29	97.2	7	0
**2022**	7.A	Population with access to clean fuels and technology for cooking (%)	40	8	0.23	86.8	29	-21
**2022**	14.F	Fish caught by trawling or dredging (%)	40	9	0.23	76.9	50	-41
**2022**	4.A	Participation rate in pre-primary organized learning (% of children aged 4 to 6)	40	10	0.22	70.5	57	-47
**2022**	1.B	Poverty headcount ratio at $2.15/day (2017 PPP, %)	40	11	0.21	97.8	3	8
**2022**	11.C	Proportion of urban population living in slums (%)	40	12	0.21	86.2	30	-18
**2022**	16.G	Access to and affordability of justice (worst 0–1 best)	40	13	0.20	72.5	55	-42
**2022**	4.D	Lower secondary completion rate (%)	40	14	0.19	88.3	27	-13
**2022**	11.A	Access to improved water source, piped (% of urban population)	40	15	0.18	93.4	12	3
**2022**	6.B	Population using at least basic sanitation services (%)	40	16	0.18	92.0	17	-1
**2022**	3.M	Universal health coverage (UHC) index of service coverage (worst 0-100 best)	40	17	0.17	54.7	75	-58
**2022**	14.C	Mean area that is protected in marine sites important to biodiversity (%)	40	18	0.15	42.9	86	-68
**2022**	2.G	Prevalence of undernourishment (%)	40	19	0.15	92.7	14	5
**2022**	16.D	Unsentenced detainees (% of prison population)	40	20	0.15	65.1	61	-41
**2022**	8.F	Victims of modern slavery (per 1,000 population)	40	21	0.14	81.0	38	-17
**2022**	16.J	Birth registrations with civil authority (% of children under age 5)	40	22	0.13	96.1	8	14
**2022**	7.C	Population with access to electricity (%)	40	23	0.13	99.7	1	22
**2022**	9.B	Population using the internet (%)	40	24	0.13	78.5	44	-20
**2022**	13.C	CO2 emissions embodied in imports (tCO2/capita)	40	25	0.12	82.6	34	-9
**2022**	6.E	Population using at least basic drinking water services (%)	40	26	0.11	94.2	11	15
**2022**	3.G	Neonatal mortality rate (per 1,000 live births)	40	27	0.11	83.8	33	-6
**2022**	3.B	Adolescent fertility rate (births per 1,000 females aged 15 to 19)	40	28	0.11	79.7	43	-15

Year	SDG indicators	Indicator name	Window group	Rank of RCA in this group	Average RCA in this group	Average SDG score in this group	Rank of SDG score in this group	Rank difference (score-RCA) for window group
**2022**	13.A	CO2/emissions embodied in fossil fuel exports (kg/capita)	40	29	0.11	94.9	10	19
**2022**	14.D	Fish caught that are then discarded (%)	40	30	0.11	56.5	73	-43
**2022**	3.A	Births attended by skilled health personnel (%)	40	31	0.11	97.4	5	26
**2022**	16.A	Timeliness of administrative proceedings (worst 0 - 1 best)	40	32	0.10	46.2	82	-50
**2022**	3.D	Life expectancy at birth (years)	40	33	0.10	75.8	52	-19
**2022**	16.F	Homicides (per 100,000 population)	40	34	0.09	85.5	31	3
**2022**	12.B	Exports of plastic waste (kg/capita)	40	35	0.09	90.1	24	11
**2022**	3.E	Maternal mortality rate (per 100,000 live births)	40	36	0.08	94.9	9	27
**2022**	3.I	Subjective well-being (average ladder score, worst 0-10 best)	40	37	0.08	55.5	74	-37
**2022**	10.A	Gini coefficient	40	38	0.08	72.7	54	-16
**2022**	4.C	Net primary enrollment rate (%)	40	39	0.08	91.6	20	19
**2022**	9.D	Mobile broadband subscriptions (per 100 population)	40	40	0.08	80.6	40	0
**2022**	2.E	Prevalence of stunting in children under 5 years of age (%)	40	41	0.08	80.7	39	2
**2022**	16.I	Population who feel safe walking alone at night in the city or area where they live (%)	40	42	0.08	57.3	72	-30
**2022**	9.F	Rural population with access to all-season roads (%)	40	43	0.08	80.3	41	2
**2022**	5.A	Ratio of female-to-male mean years of education received (%)	40	44	0.07	91.8	18	26
**2022**	3.L	Mortality rate, under-5 (per 1,000 live births)	40	45	0.07	92.0	15	30
**2022**	12.D	Nitrogen emissions embodied in imports (kg/capita)	40	46	0.07	77.9	46	0
**2022**	11.D	Satisfaction with public transport (%)	40	47	0.07	62.3	65	-18
**2022**	3.K	Traffic deaths (per 100,000 population)	40	48	0.07	62.6	64	-16
**2022**	10.B	Palma ratio	40	49	0.07	53.9	76	-27
**2022**	12.E	Production-based nitrogen emissions (kg/capita)	40	50	0.06	71.6	56	-6
**2022**	3.N	Surviving infants who received 2 WHO-recommended vaccines (%)	40	51	0.05	76.7	51	0
**2022**	3.J	Incidence of tuberculosis (per 100,000 population)	40	52	0.04	90.1	23	29
**2022**	2.D	Sustainable Nitrogen Management Index (best 0-1.41 worst)	40	53	0.04	32.6	91	-38
**2022**	7.B	CO2 emissions from fuel combustion per total electricity output (MtCO2 TWh)	40	54	0.04	77.9	47	7
**2022**	3.H	Age-standardized death rate attributable to household air pollution and ambient air pollution (per 100,000 population)	40	55	0.04	81.5	37	18
**2022**	17.C	Statistical Performance Index (worst 0-100 best)	40	56	0.04	67.5	59	-3
**2022**	3.F	Age-standardized death rate due to cardiovascular disease, cancer, diabetes, or chronic respiratory disease in adults aged 30–70 years (%)	40	57	0.04	61.8	67	-10
**2022**	8.D	Victims of modern slavery embodied in imports (per 100,000 population)	40	58	0.03	87.8	28	30
**2022**	12.F	SO2 emissions embodied in imports (kg/capita)	40	59	0.03	91.7	19	40
**2022**	15.D	Permanent deforestation (% of forest area, 3-year average)	40	60	0.03	82.1	35	25

Year	SDG indicators	Indicator name	Window group	Rank of RCA in this group	Average RCA in this group	Average SDG score in this group	Rank of SDG score in this group	Rank difference (score-RCA) for window group
**2022**	8.A	Adults with an account at a bank or other financial institution or with a mobile-money-service provider (% of population aged 15 or over)	40	61	0.02	62.0	66	-5
**2022**	6.C	Scarce water consumption embodied in imports (m3 H2O eq/capita)	40	62	0.02	81.9	36	26
**2022**	14.A	Marine biodiversity threats embodied in imports (per million population)	40	63	0.02	92.0	16	47
**2022**	5.B	Demand for family planning satisfied by modern methods (% of females aged 15 to 49)	40	64	0.02	59.5	68	-4
**2022**	12.A	Electronic waste (kg/capita)	40	65	0.01	64.9	62	3
**2022**	8.C	Fatal work-related accidents embodied in imports (per 100,000 population)	40	66	0.01	98.5	2	64
**2022**	17.B	Government spending on health and education (% of GDP)	40	67	0.01	58.3	71	-4
**2022**	11.B	Annual mean concentration of particulate matter of less than 2.5 microns in diameter (PM2.5) (μg/m³)	40	68	0.01	77.7	48	20
**2022**	2.H	Prevalence of wasting in children under 5 years of age (%)	40	69	0.01	78.0	45	24
**2022**	8.B	Adjusted GDP growth (%)	40	70	0.00	64.7	63	7
**2022**	12.G	Production-based SO2 emissions (kg/capita)	40	71	0.00	97.2	6	65
**2022**	8.E	Fundamental labor rights are effectively guaranteed (worst 0–1 best)	40	72	0.00	44.7	84	-12
**2022**	9.G	The Times Higher Education Universities Ranking: Average score of top 3 universities (worst 0-100 best)	40	73	0.00	45.0	83	-10
**2022**	15.A	Terrestrial and freshwater biodiversity threats embodied in imports (per million population)	40	74	-0.01	90.7	22	52
**2022**	17.A	Corporate Tax Haven Score (best 0-100 worst)	40	75	-0.01	90.7	21	54
**2022**	12.C	Municipal solid waste (kg/capita/day)	40	76	-0.01	77.5	49	27
**2022**	2.A	Cereal yield (tonnes per hectare of harvested land)	40	77	-0.03	50.0	78	-1
**2022**	16.K	Exports of major conventional weapons (TIV constant million USD per 100,000 population)	40	78	-0.03	92.9	13	65
**2022**	13.B	CO2 emissions from fossil fuel combustion and cement production (tCO2/capita)	40	79	-0.03	79.8	42	37
**2022**	5.D	Seats held by women in national parliament (%)	40	80	-0.04	50.4	77	3
**2022**	8.G	Unemployment rate (% of total labor force, ages 15+)	40	81	-0.05	73.1	53	28
**2022**	9.C	Logistics Performance Index: Quality of trade and transport-related infrastructure (worst 1-5 best)	40	82	-0.06	48.7	79	3
**2022**	2.F	Human Trophic Level (best 2-3 worst)	40	83	-0.06	42.0	87	-4
**2022**	7.D	Renewable energy share in total final energy consumption (%)	40	84	-0.06	22.7	94	-10
**2022**	16.C	Corruption Perceptions Index (worst 0-100 best)	40	85	-0.08	38.0	89	-4
**2022**	9.A	Articles published in academic journals (per 1,000 population)	40	86	-0.09	38.6	88	-2
**2022**	15.B	Mean area that is protected in freshwater sites important to biodiversity (%)	40	87	-0.10	46.6	81	6

Year	SDG indicators	Indicator name	Window group	Rank of RCA in this group	Average RCA in this group	Average SDG score in this group	Rank of SDG score in this group	Rank difference (score-RCA) for window group
**2022**	16.E	Expropriations are lawful and adequately compensated (worst 0 - 1 best)	40	88	-0.10	26.9	93	-5
**2022**	15.E	Red List Index of species survival (worst 0-1 best)	40	89	-0.13	58.4	70	19
**2022**	5.C	Ratio of female-to-male labor force participation rate (%)	40	90	-0.14	58.6	69	21
**2022**	9.E	Expenditure on research and development (% of GDP)	40	91	-0.15	17.4	95	-4
**2022**	2.B	Prevalence of obesity, BMI ≥ 30 (% of adult population)	40	92	-0.18	44.3	85	7
**2022**	6.A	Freshwater withdrawal (% of available freshwater resources)	40	93	-0.20	65.8	60	33
**2022**	16.H	Press Freedom Index (worst 0-100 best)	40	94	-0.27	30.5	92	2
**2022**	15.C	Mean area that is protected in terrestrial sites important to biodiversity (%)	40	95	-0.30	36.3	90	5
**2022**	6.D	Anthropogenic wastewater that receives treatment (%)	40	96	-0.48	12.0	96	0

Supplementary Table 12 | The RCA and SDG scores of SDG indicators in the 80th window group of countries in 2022. Note: The SDG indicators listed by the rank order of revealed comparative advantage (RCA). The RCA results are generated by the moving-window approach with window size of 50 (see Methods).

Year	SDG indicators	Indicator name	Window group	Rank of RCA in this group	Average RCA in this group	Average SDG score in this group	Rank of SDG score in this group	Rank difference (score-RCA) for window group
**2022**	14.E	Fish caught from overexploited or collapsed stocks (% of total catch)	80	1	0.84	74.1	36	-35
**2022**	14.F	Fish caught by trawling or dredging (%)	80	2	0.49	82.3	23	-21
**2022**	16.B	Children involved in child labor (% of population aged 5 to 14)	80	3	0.48	71.7	38	-35
**2022**	14.D	Fish caught that are then discarded (%)	80	4	0.48	66.9	52	-48
**2022**	2.C	Exports of hazardous pesticides (tonnes per million population)	80	5	0.46	90.5	9	-4
**2022**	3.C	New HIV infections (per 1,000 uninfected population)	80	6	0.35	90.8	7	-1
**2022**	4.B	Literacy rate (% of population aged 15 to 24)	80	7	0.35	90.5	10	-3
**2022**	2.F	Human Trophic Level (best 2-3 worst)	80	8	0.34	53.6	67	-59
**2022**	14.B	Ocean Health Index: Clean Waters score (worst 0-100 best)	80	9	0.31	40.9	83	-74
**2022**	12.B	Exports of plastic waste (kg/capita)	80	10	0.30	95.1	4	6
**2022**	12.A	Electronic waste (kg/capita)	80	11	0.25	71.4	39	-28
**2022**	11.D	Satisfaction with public transport (%)	80	12	0.25	64.5	55	-43
**2022**	13.C	CO2 emissions embodied in imports (tCO2/capita)	80	13	0.23	81.0	24	-11
**2022**	1.B	Poverty headcount ratio at $2.15/day (2017 PPP, %)	80	14	0.22	88.7	13	1
**2022**	12.D	Nitrogen emissions embodied in imports (kg/capita)	80	15	0.22	78.9	29	-14
**2022**	12.F	SO2 emissions embodied in imports (kg/capita)	80	16	0.17	92.1	6	10
**2022**	8.D	Victims of modern slavery embodied in imports (per 100,000 population)	80	17	0.17	88.0	14	3
**2022**	8.F	Victims of modern slavery (per 1,000 population)	80	18	0.17	74.8	33	-15
**2022**	12.E	Production-based nitrogen emissions (kg/capita)	80	19	0.17	70.1	47	-28
**2022**	16.G	Access to and affordability of justice (worst 0–1 best)	80	20	0.16	61.8	60	-40
**2022**	14.C	Mean area that is protected in marine sites important to biodiversity (%)	80	21	0.16	37.5	86	-65
**2022**	12.C	Municipal solid waste (kg/capita/day)	80	22	0.15	80.7	26	-4
**2022**	8.C	Fatal work-related accidents embodied in imports (per 100,000 population)	80	23	0.15	98.8	1	22
**2022**	7.C	Population with access to electricity (%)	80	24	0.15	90.0	11	13
**2022**	13.A	CO2/emissions embodied in fossil fuel exports (kg/capita)	80	25	0.15	88.0	15	10
**2022**	16.K	Exports of major conventional weapons (TIV constant million USD per 100,000 population)	80	26	0.14	97.4	2	24
**2022**	2.G	Prevalence of undernourishment (%)	80	27	0.14	80.7	25	2
**2022**	16.J	Birth registrations with civil authority (% of children under age 5)	80	28	0.14	86.1	17	11
**2022**	12.G	Production-based SO2 emissions (kg/capita)	80	29	0.13	96.8	3	26

Year	SDG indicators	Indicator name	Window group	Rank of RCA in this group	Average RCA in this group	Average SDG score in this group	Rank of SDG score in this group	Rank difference (score-RCA) for window group
**2022**	11.A	Access to improved water source, piped (% of urban population)	80	30	0.13	80.5	27	3
**2022**	17.A	Corporate Tax Haven Score (best 0-100 worst)	80	31	0.13	92.8	5	26
**2022**	11.C	Proportion of urban population living in slums (%)	80	32	0.13	70.5	44	-12
**2022**	6.E	Population using at least basic drinking water services (%)	80	33	0.12	84.4	20	13
**2022**	6.C	Scarce water consumption embodied in imports (m3 H2O eq/capita)	80	34	0.12	80.0	28	6
**2022**	8.E	Fundamental labor rights are effectively guaranteed (worst 0–1 best)	80	35	0.12	44.1	79	-44
**2022**	3.A	Births attended by skilled health personnel (%)	80	36	0.12	87.4	16	20
**2022**	4.C	Net primary enrollment rate (%)	80	37	0.12	84.5	19	18
**2022**	15.A	Terrestrial and freshwater biodiversity threats embodied in imports (per million population)	80	38	0.11	90.6	8	30
**2022**	14.A	Marine biodiversity threats embodied in imports (per million population)	80	39	0.11	89.5	12	27
**2022**	5.A	Ratio of female-to-male mean years of education received (%)	80	40	0.11	84.2	21	19
**2022**	1.A	Poverty headcount ratio at $3.65/day (2017 PPP, %)	80	41	0.10	63.5	58	-17
**2022**	6.B	Population using at least basic sanitation services (%)	80	42	0.10	76.2	31	11
**2022**	5.B	Demand for family planning satisfied by modern methods (% of females aged 15 to 49)	80	43	0.10	57.0	64	-21
**2022**	3.N	Surviving infants who received 2 WHO-recommended vaccines (%)	80	44	0.10	71.1	40	4
**2022**	3.E	Maternal mortality rate (per 100,000 live births)	80	45	0.09	85.0	18	27
**2022**	2.B	Prevalence of obesity, BMI ≥ 30 (% of adult population)	80	46	0.09	52.6	69	-23
**2022**	4.D	Lower secondary completion rate (%)	80	47	0.09	72.6	37	10
**2022**	3.L	Mortality rate, under-5 (per 1,000 live births)	80	48	0.09	82.7	22	26
**2022**	16.F	Homicides (per 100,000 population)	80	49	0.07	74.7	34	15
**2022**	7.B	CO2 emissions from fuel combustion per total electricity output (MtCO2 TWh)	80	50	0.07	71.1	41	9
**2022**	15.D	Permanent deforestation (% of forest area, 3-year average)	80	51	0.07	75.9	32	19
**2022**	9.D	Mobile broadband subscriptions (per 100 population)	80	52	0.06	70.2	45	7
**2022**	2.E	Prevalence of stunting in children under 5 years of age (%)	80	53	0.06	70.0	48	5
**2022**	16.D	Unsentenced detainees (% of prison population)	80	54	0.05	52.1	70	-16
**2022**	10.A	Gini coefficient	80	55	0.05	62.8	59	-4
**2022**	7.A	Population with access to clean fuels and technology for cooking (%)	80	56	0.04	65.3	53	3
**2022**	9.B	Population using the internet (%)	80	57	0.03	63.6	57	0
**2022**	8.B	Adjusted GDP growth (%)	80	58	0.03	58.6	62	-4
**2022**	9.F	Rural population with access to all-season roads (%)	80	59	0.03	68.2	51	8
**2022**	8.G	Unemployment rate (% of total labor force, ages 15+)	80	60	0.03	70.6	42	18
**2022**	3.B	Adolescent fertility rate (births per 1,000 females aged 15 to 19)	80	61	0.03	65.2	54	7
**2022**	3.G	Neonatal mortality rate (per 1,000 live births)	80	62	0.03	68.8	49	13

Year	SDG indicators	Indicator name	Window group	Rank of RCA in this group	Average RCA in this group	Average SDG score in this group	Rank of SDG score in this group	Rank difference (score-RCA) for window group
**2022**	3.H	Age-standardized death rate attributable to household air pollution and ambient air pollution (per 100,000 population)	80	63	0.02	70.6	43	20
**2022**	4.A	Participation rate in pre-primary organized learning (% of children aged 4 to 6)	80	64	0.01	52.1	71	-7
**2022**	3.I	Subjective well-being (average ladder score, worst 0-10 best)	80	65	0.01	45.5	76	-11
**2022**	3.J	Incidence of tuberculosis (per 100,000 population)	80	66	0.01	76.9	30	36
**2022**	8.A	Adults with an account at a bank or other financial institution or with a mobile-money-service provider (% of population aged 15 or over)	80	67	0.01	54.3	66	1
**2022**	2.H	Prevalence of wasting in children under 5 years of age (%)	80	68	0.00	68.4	50	18
**2022**	13.B	CO2 emissions from fossil fuel combustion and cement production (tCO2/capita)	80	69	0.00	74.2	35	34
**2022**	15.B	Mean area that is protected in freshwater sites important to biodiversity (%)	80	70	0.00	45.4	77	-7
**2022**	2.A	Cereal yield (tonnes per hectare of harvested land)	80	71	0.00	45.2	78	-7
**2022**	16.I	Population who feel safe walking alone at night in the city or area where they live (%)	80	72	0.00	47.2	75	-3
**2022**	2.D	Sustainable Nitrogen Management Index (best 0-1.41 worst)	80	73	0.00	27.8	90	-17
**2022**	3.D	Life expectancy at birth (years)	80	74	-0.01	60.6	61	13
**2022**	16.A	Timeliness of administrative proceedings (worst 0 - 1 best)	80	75	-0.02	36.5	87	-12
**2022**	3.M	Universal health coverage (UHC) index of service coverage (worst 0-100 best)	80	76	-0.02	40.5	84	-8
**2022**	15.E	Red List Index of species survival (worst 0-1 best)	80	77	-0.03	57.8	63	14
**2022**	17.B	Government spending on health and education (% of GDP)	80	78	-0.03	50.0	72	6
**2022**	6.A	Freshwater withdrawal (% of available freshwater resources)	80	79	-0.04	70.1	46	33
**2022**	16.E	Expropriations are lawful and adequately compensated (worst 0 - 1 best)	80	80	-0.05	24.9	92	-12
**2022**	3.K	Traffic deaths (per 100,000 population)	80	81	-0.06	48.9	73	8
**2022**	11.B	Annual mean concentration of particulate matter of less than 2.5 microns in diameter (PM2.5) (μg/m³)	80	82	-0.07	63.7	56	26
**2022**	15.C	Mean area that is protected in terrestrial sites important to biodiversity (%)	80	83	-0.08	41.6	82	1
**2022**	5.D	Seats held by women in national parliament (%)	80	84	-0.08	43.2	80	4
**2022**	17.C	Statistical Performance Index (worst 0-100 best)	80	85	-0.08	53.2	68	17
**2022**	3.F	Age-standardized death rate due to cardiovascular disease, cancer, diabetes, or chronic respiratory disease in adults aged 30–70 years (%)	80	86	-0.09	48.1	74	12
**2022**	9.C	Logistics Performance Index: Quality of trade and transport-related infrastructure (worst 1-5 best)	80	87	-0.09	41.6	81	6
**2022**	5.C	Ratio of female-to-male labor force participation rate (%)	80	88	-0.10	54.8	65	23
**2022**	16.C	Corruption Perceptions Index (worst 0-100 best)	80	89	-0.11	32.3	88	1

Year	SDG indicators	Indicator name	Window group	Rank of RCA in this group	Average RCA in this group	Average SDG score in this group	Rank of SDG score in this group	Rank difference (score-RCA) for window group
**2022**	10.B	Palma ratio	80	90	-0.16	37.5	85	5
**2022**	9.G	The Times Higher Education Universities Ranking: Average score of top 3 universities (worst 0-100 best)	80	91	-0.24	30.3	89	2
**2022**	16.H	Press Freedom Index (worst 0-100 best)	80	92	-0.27	27.4	91	1
**2022**	7.D	Renewable energy share in total final energy consumption (%)	80	93	-0.36	14.0	94	-1
**2022**	9.A	Articles published in academic journals (per 1,000 population)	80	94	-0.37	23.6	93	1
**2022**	9.E	Expenditure on research and development (% of GDP)	80	95	-0.55	8.2	95	0
**2022**	6.D	Anthropogenic wastewater that receives treatment (%)	80	96	-0.68	6.1	96	0

Supplementary Table 13 | The RCA and SDG scores of SDG indicators in the 117th window group of countries in 2022. Note: The SDG indicators listed by the rank order of revealed comparative advantage (RCA). The RCA results are generated by the moving-window approach with window size of 50 (see Methods).

Year	SDG indicators	Indicator name	Window group	Rank of RCA in this group	Average RCA in this group	Average SDG score in this group	Rank of SDG score in this group	Rank difference (score-RCA) for window group
**2022**	14.E	Fish caught from overexploited or collapsed stocks (% of total catch)	117	1	1.46	81.7	22	-21
**2022**	2.F	Human Trophic Level (best 2-3 worst)	117	2	1.09	68.9	28	-26
**2022**	14.D	Fish caught that are then discarded (%)	117	3	0.95	75.4	25	-22
**2022**	12.A	Electronic waste (kg/capita)	117	4	0.95	92.4	15	-11
**2022**	14.F	Fish caught by trawling or dredging (%)	117	5	0.90	89.0	18	-13
**2022**	2.B	Prevalence of obesity, BMI ≥ 30 (% of adult population)	117	6	0.89	75.1	26	-20
**2022**	2.C	Exports of hazardous pesticides (tonnes per million population)	117	7	0.82	95.8	12	-5
**2022**	13.C	CO2 emissions embodied in imports (tCO2/capita)	117	8	0.80	95.1	13	-5
**2022**	12.E	Production-based nitrogen emissions (kg/capita)	117	9	0.77	86.5	20	-11
**2022**	12.D	Nitrogen emissions embodied in imports (kg/capita)	117	10	0.77	92.2	16	-6
**2022**	6.C	Scarce water consumption embodied in imports (m3 H2O eq/capita)	117	11	0.64	93.8	14	-3
**2022**	13.B	CO2 emissions from fossil fuel combustion and cement production (tCO2/capita)	117	12	0.63	97.0	8	4
**2022**	12.B	Exports of plastic waste (kg/capita)	117	13	0.60	99.1	5	8
**2022**	8.D	Victims of modern slavery embodied in imports (per 100,000 population)	117	14	0.59	96.6	9	5
**2022**	12.C	Municipal solid waste (kg/capita/day)	117	15	0.59	89.8	17	-2
**2022**	13.A	CO2/emissions embodied in fossil fuel exports (kg/capita)	117	16	0.57	99.5	3	13
**2022**	3.C	New HIV infections (per 1,000 uninfected population)	117	17	0.56	86.0	21	-4
**2022**	12.F	SO2 emissions embodied in imports (kg/capita)	117	18	0.54	97.9	6	12
**2022**	15.A	Terrestrial and freshwater biodiversity threats embodied in imports (per million population)	117	19	0.48	97.9	7	12
**2022**	6.A	Freshwater withdrawal (% of available freshwater resources)	117	20	0.46	87.0	19	1
**2022**	17.A	Corporate Tax Haven Score (best 0-100 worst)	117	21	0.45	96.1	10	11
**2022**	16.K	Exports of major conventional weapons (TIV constant million USD per 100,000 population)	117	22	0.44	100.0	1	21
**2022**	14.A	Marine biodiversity threats embodied in imports (per million population)	117	23	0.44	96.1	11	12
**2022**	12.G	Production-based SO2 emissions (kg/capita)	117	24	0.43	99.2	4	20
**2022**	8.C	Fatal work-related accidents embodied in imports (per 100,000 population)	117	25	0.43	99.7	2	23
**2022**	5.C	Ratio of female-to-male labor force participation rate (%)	117	26	0.36	68.1	29	-3
**2022**	15.D	Permanent deforestation (% of forest area, 3-year average)	117	27	0.34	75.8	24	3

Year	SDG indicators	Indicator name	Window group	Rank of RCA in this group	Average RCA in this group	Average SDG score in this group	Rank of SDG score in this group	Rank difference (score-RCA) for window group
**2022**	15.E	Red List Index of species survival (worst 0-1 best)	117	28	0.34	64.8	32	-4
**2022**	14.C	Mean area that is protected in marine sites important to biodiversity (%)	117	29	0.34	37.1	67	-38
**2022**	16.F	Homicides (per 100,000 population)	117	30	0.31	76.8	23	7
**2022**	15.B	Mean area that is protected in freshwater sites important to biodiversity (%)	117	31	0.29	47.2	52	-21
**2022**	10.A	Gini coefficient	117	32	0.24	60.5	37	-5
**2022**	8.G	Unemployment rate (% of total labor force, ages 15+)	117	33	0.22	69.7	27	6
**2022**	15.C	Mean area that is protected in terrestrial sites important to biodiversity (%)	117	34	0.21	44.9	56	-22
**2022**	16.G	Access to and affordability of justice (worst 0–1 best)	117	35	0.21	55.6	41	-6
**2022**	8.F	Victims of modern slavery (per 1,000 population)	117	36	0.13	60.8	36	0
**2022**	16.D	Unsentenced detainees (% of prison population)	117	37	0.12	47.7	50	-13
**2022**	7.B	CO2 emissions from fuel combustion per total electricity output (MtCO2 TWh)	117	38	0.12	61.8	33	5
**2022**	8.E	Fundamental labor rights are effectively guaranteed (worst 0–1 best)	117	39	0.08	37.3	65	-26
**2022**	16.A	Timeliness of administrative proceedings (worst 0 - 1 best)	117	40	0.07	34.3	72	-32
**2022**	5.D	Seats held by women in national parliament (%)	117	41	0.07	40.6	60	-19
**2022**	11.A	Access to improved water source, piped (% of urban population)	117	42	0.07	61.8	34	8
**2022**	11.B	Annual mean concentration of particulate matter of less than 2.5 microns in diameter (PM2.5) (μg/m³)	117	43	0.06	59.9	38	5
**2022**	4.C	Net primary enrollment rate (%)	117	44	0.06	67.4	30	14
**2022**	3.J	Incidence of tuberculosis (per 100,000 population)	117	45	0.04	65.8	31	14
**2022**	4.B	Literacy rate (% of population aged 15 to 24)	117	46	0.04	59.0	39	7
**2022**	2.H	Prevalence of wasting in children under 5 years of age (%)	117	47	-0.01	56.9	40	7
**2022**	16.B	Children involved in child labor (% of population aged 5 to 14)	117	48	-0.01	40.2	61	-13
**2022**	8.B	Adjusted GDP growth (%)	117	49	-0.03	46.6	53	-4
**2022**	2.D	Sustainable Nitrogen Management Index (best 0-1.41 worst)	117	50	-0.03	23.1	82	-32
**2022**	16.H	Press Freedom Index (worst 0-100 best)	117	51	-0.05	29.0	79	-28
**2022**	3.A	Births attended by skilled health personnel (%)	117	52	-0.07	61.6	35	17
**2022**	10.B	Palma ratio	117	53	-0.08	35.0	71	-18
**2022**	1.B	Poverty headcount ratio at $2.15/day (2017 PPP, %)	117	54	-0.09	55.5	42	12
**2022**	5.B	Demand for family planning satisfied by modern methods (% of females aged 15 to 49)	117	55	-0.11	39.4	62	-7
**2022**	3.H	Age-standardized death rate attributable to household air pollution and ambient air pollution (per 100,000 population)	117	56	-0.11	50.6	48	8
**2022**	17.C	Statistical Performance Index (worst 0-100 best)	117	57	-0.12	42.6	58	-1
**2022**	11.D	Satisfaction with public transport (%)	117	58	-0.13	37.2	66	-8
**2022**	5.A	Ratio of female-to-male mean years of education received (%)	117	59	-0.13	55.4	43	16
**2022**	16.J	Birth registrations with civil authority (% of children under age 5)	117	60	-0.14	54.9	44	16

Year	SDG indicators	Indicator name	Window group	Rank of RCA in this group	Average RCA in this group	Average SDG score in this group	Rank of SDG score in this group	Rank difference (score-RCA) for window group
**2022**	16.I	Population who feel safe walking alone at night in the city or area where they live (%)	117	61	-0.14	32.6	76	-15
**2022**	3.N	Surviving infants who received 2 WHO-recommended vaccines (%)	117	62	-0.14	47.4	51	11
**2022**	14.B	Ocean Health Index: Clean Waters score (worst 0-100 best)	117	63	-0.15	22.0	86	-23
**2022**	3.L	Mortality rate, under-5 (per 1,000 live births)	117	64	-0.16	53.7	46	18
**2022**	17.B	Government spending on health and education (% of GDP)	117	65	-0.16	36.0	68	-3
**2022**	3.E	Maternal mortality rate (per 100,000 live births)	117	66	-0.17	54.6	45	21
**2022**	11.C	Proportion of urban population living in slums (%)	117	67	-0.17	45.0	55	12
**2022**	2.G	Prevalence of undernourishment (%)	117	68	-0.18	51.0	47	21
**2022**	2.E	Prevalence of stunting in children under 5 years of age (%)	117	69	-0.19	44.5	57	12
**2022**	7.D	Renewable energy share in total final energy consumption (%)	117	70	-0.19	14.1	91	-21
**2022**	3.F	Age-standardized death rate due to cardiovascular disease, cancer, diabetes, or chronic respiratory disease in adults aged 30–70 years (%)	117	71	-0.21	34.3	73	-2
**2022**	9.C	Logistics Performance Index: Quality of trade and transport-related infrastructure (worst 1-5 best)	117	72	-0.26	28.3	80	-8
**2022**	16.E	Expropriations are lawful and adequately compensated (worst 0 - 1 best)	117	73	-0.26	17.7	89	-16
**2022**	9.F	Rural population with access to all-season roads (%)	117	74	-0.27	40.9	59	15
**2022**	7.C	Population with access to electricity (%)	117	75	-0.27	47.7	49	26
**2022**	6.E	Population using at least basic drinking water services (%)	117	76	-0.28	46.1	54	22
**2022**	16.C	Corruption Perceptions Index (worst 0-100 best)	117	77	-0.30	22.4	85	-8
**2022**	3.D	Life expectancy at birth (years)	117	78	-0.30	35.8	69	9
**2022**	8.A	Adults with an account at a bank or other financial institution or with a mobile-money-service provider (% of population aged 15 or over)	117	79	-0.32	31.2	78	1
**2022**	9.D	Mobile broadband subscriptions (per 100 population)	117	80	-0.34	37.8	63	17
**2022**	3.G	Neonatal mortality rate (per 1,000 live births)	117	81	-0.34	37.3	64	17
**2022**	3.B	Adolescent fertility rate (births per 1,000 females aged 15 to 19)	117	82	-0.36	34.1	74	8
**2022**	4.D	Lower secondary completion rate (%)	117	83	-0.37	35.6	70	13
**2022**	9.B	Population using the internet (%)	117	84	-0.38	32.4	77	7
**2022**	2.A	Cereal yield (tonnes per hectare of harvested land)	117	85	-0.41	22.5	84	1
**2022**	6.B	Population using at least basic sanitation services (%)	117	86	-0.43	33.1	75	11
**2022**	3.K	Traffic deaths (per 100,000 population)	117	87	-0.47	23.1	83	4
**2022**	4.A	Participation rate in pre-primary organized learning (% of children aged 4 to 6)	117	88	-0.48	23.7	81	7
**2022**	3.I	Subjective well-being (average ladder score, worst 0-10 best)	117	89	-0.49	19.3	88	1
**2022**	7.A	Population with access to clean fuels and technology for cooking (%)	117	90	-0.62	20.0	87	3
**2022**	3.M	Universal health coverage (UHC) index of service coverage (worst 0-100 best)	117	91	-0.65	12.6	92	-1
**2022**	1.A	Poverty headcount ratio at $3.65/day (2017 PPP, %)	117	92	-0.67	16.6	90	2

Year	SDG indicators	Indicator name	Window group	Rank of RCA in this group	Average RCA in this group	Average SDG score in this group	Rank of SDG score in this group	Rank difference (score-RCA) for window group
**2022**	9.E	Expenditure on research and development (% of GDP)	117	93	-0.68	5.0	94	-1
**2022**	9.G	The Times Higher Education Universities Ranking: Average score of top 3 universities (worst 0-100 best)	117	94	-0.70	10.5	93	1
**2022**	6.D	Anthropogenic wastewater that receives treatment (%)	117	95	-0.82	2.8	96	-1
**2022**	9.A	Articles published in academic journals (per 1,000 population)	117	96	-0.85	4.7	95	1

Supplementary Table 14 | The changes in revealed comparative advantage (RCA) of SDG indicators in each sustainability stage in 2000.

Year	SDG indicator	Indicator name	RCA in Stage 1	RCA in Stage 2	RCA in Stage 3	RCA in Stage 4	GSI
**2000**	1.A	Poverty headcount ratio at $3.65/day (2017 PPP, %)	-0.86	-0.07	0.49	0.46	0.99
**2000**	1.B	Poverty headcount ratio at $2.15/day (2017 PPP, %)	-0.14	0.19	0.24	0.08	0.40
**2000**	2.A	Cereal yield (tonnes per hectare of harvested land)	-0.44	-0.06	0.00	0.34	0.56
**2000**	2.B	Prevalence of obesity, BMI ≥ 30 (% of adult population)	0.85	0.05	-0.12	-0.36	-1.74
**2000**	2.C	Exports of hazardous pesticides (tonnes per million population)	1.01	0.38	0.27	0.10	0.13
**2000**	2.D	Sustainable Nitrogen Management Index (best 0-1.41 worst)	0.15	-0.12	0.00	-0.03	-0.58
**2000**	2.E	Prevalence of stunting in children under 5 years of age (%)	-0.57	-0.09	0.15	0.32	1.20
**2000**	2.F	Human Trophic Level (best 2-3 worst)	1.34	0.25	-0.17	-0.69	-1.71
**2000**	2.G	Prevalence of undernourishment (%)	-0.35	0.10	0.17	0.17	1.02
**2000**	2.H	Prevalence of wasting in children under 5 years of age (%)	-0.23	0.08	-0.01	0.11	0.70
**2000**	3.A	Births attended by skilled health personnel (%)	-0.53	0.07	0.24	0.17	0.98
**2000**	3.B	Adolescent fertility rate (births per 1,000 females aged 15 to 19)	-0.53	0.01	0.18	0.17	0.78
**2000**	3.C	New HIV infections (per 1,000 uninfected population)	0.34	0.35	0.41	0.25	-0.10
**2000**	3.D	Life expectancy at birth (years)	-0.80	0.01	0.21	0.28	1.12
**2000**	3.E	Maternal mortality rate (per 100,000 live births)	-0.61	0.17	0.20	0.08	0.94
**2000**	3.F	Age-standardized death rate due to cardiovascular disease, cancer, diabetes, or chronic respiratory disease in adults aged 30–70 years (%)	-0.23	-0.08	-0.06	0.20	0.39
**2000**	3.G	Neonatal mortality rate (per 1,000 live births)	-0.84	0.01	0.14	0.38	1.24
**2000**	3.H	Age-standardized death rate attributable to household air pollution and ambient air pollution (per 100,000 population)	-0.10	0.03	0.05	0.00	0.31
**2000**	3.I	Subjective well-being (average ladder score, worst 0-10 best)	-0.39	0.10	0.05	0.25	0.79
**2000**	3.J	Incidence of tuberculosis (per 100,000 population)	-0.11	-0.04	0.12	0.02	0.19
**2000**	3.K	Traffic deaths (per 100,000 population)	-0.34	0.08	0.13	0.07	0.28
**2000**	3.L	Mortality rate, under-5 (per 1,000 live births)	-0.82	0.08	0.23	0.24	1.21
**2000**	3.M	Universal health coverage (UHC) index of service coverage (worst 0-100 best)	-1.00	-0.38	-0.08	0.91	1.40
**2000**	3.N	Surviving infants who received 2 WHO-recommended vaccines (%)	-0.44	0.14	0.15	0.02	0.16
**2000**	4.A	Participation rate in pre-primary organized learning (% of children aged 4 to 6)	-0.61	0.05	0.21	0.42	0.88
**2000**	4.B	Literacy rate (% of population aged 15 to 24)	-0.17	0.38	0.39	0.26	-0.08
**2000**	4.C	Net primary enrollment rate (%)	-0.45	0.20	0.20	0.09	0.51
**2000**	4.D	Lower secondary completion rate (%)	-0.77	0.04	0.23	0.32	1.05
**2000**	5.A	Ratio of female-to-male mean years of education received (%)	-0.47	0.12	0.16	0.08	0.54
**2000**	5.B	Demand for family planning satisfied by modern methods (% of females aged 15 to 49)	-0.52	0.22	0.07	0.17	0.61
**2000**	5.C	Ratio of female-to-male labor force participation rate (%)	0.62	-0.17	-0.15	-0.09	-1.19
**2000**	5.D	Seats held by women in national parliament (%)	0.06	-0.15	-0.23	0.29	-0.03
**2000**	6.A	Freshwater withdrawal (% of available freshwater resources)	0.61	-0.09	-0.12	-0.15	-1.14
**2000**	6.B	Population using at least basic sanitation services (%)	-0.69	-0.01	0.24	0.22	1.17
**2000**	6.C	Scarce water consumption embodied in imports (m3 H2O eq/capita)	0.77	0.10	0.01	-0.27	-1.57
**2000**	6.D	Anthropogenic wastewater that receives treatment (%)	-0.88	-0.50	-0.56	1.32	1.51

Year	SDG indicator	Indicator name	RCA in Stage 1	RCA in Stage 2	RCA in Stage 3	RCA in Stage 4	GSI
**2000**	6.E	Population using at least basic drinking water services (%)	-0.61	0.10	0.19	0.14	0.98
**2000**	7.A	Population with access to clean fuels and technology for cooking (%)	-0.85	0.06	0.19	0.35	1.19
**2000**	7.B	CO2 emissions from fuel combustion per total electricity output (MtCO2 TWh)	0.05	0.09	0.03	-0.08	-0.85
**2000**	7.C	Population with access to electricity (%)	-0.76	0.10	0.28	0.14	0.99
**2000**	7.D	Renewable energy share in total final energy consumption (%)	-0.03	-0.19	0.16	0.04	-0.15
**2000**	8.A	Adults with an account at a bank or other financial institution or with a mobile-money-service provider (% of population aged 15 or over)	-0.64	-0.14	-0.02	0.66	1.23
**2000**	8.B	Adjusted GDP growth (%)	0.02	0.06	-0.01	0.00	-0.42
**2000**	8.C	Fatal work-related accidents embodied in imports (per 100,000 population)	0.50	0.16	0.00	-0.16	-1.59
**2000**	8.D	Victims of modern slavery embodied in imports (per 100,000 population)	0.69	0.15	0.02	-0.27	-1.51
**2000**	8.E	Fundamental labor rights are effectively guaranteed (worst 0–1 best)	0.09	0.05	0.07	0.45	0.42
**2000**	8.F	Victims of modern slavery (per 1,000 population)	0.16	0.19	0.14	0.03	-0.14
**2000**	8.G	Unemployment rate (% of total labor force, ages 15+)	0.45	0.14	-0.16	-0.22	-1.28
**2000**	9.A	Articles published in academic journals (per 1,000 population)	-0.96	-0.78	-0.59	1.60	1.99
**2000**	9.B	Population using the internet (%)	-1.00	-0.74	-0.55	1.55	1.81
**2000**	9.C	Logistics Performance Index: Quality of trade and transport-related infrastructure (worst 1-5 best)	-0.36	-0.02	-0.11	0.37	0.89
**2000**	9.D	Mobile broadband subscriptions (per 100 population)	-0.98	-0.55	-0.37	1.25	1.60
**2000**	9.E	Expenditure on research and development (% of GDP)	-0.62	-0.56	-0.34	1.20	1.21
**2000**	9.F	Rural population with access to all-season roads (%)	-0.29	0.03	0.08	0.11	0.57
**2000**	9.G	The Times Higher Education Universities Ranking: Average score of top 3 universities (worst 0-100 best)	-0.79	-0.63	0.09	0.80	0.80
**2000**	10.A	Gini coefficient	0.36	0.01	0.04	0.13	-0.29
**2000**	10.B	Palma ratio	-0.08	-0.11	-0.01	0.49	0.35
**2000**	11.A	Access to improved water source, piped (% of urban population)	0.10	0.19	0.13	0.05	-0.35
**2000**	11.B	Annual mean concentration of particulate matter of less than 2.5 microns in diameter (PM2.5) (μg/m³)	0.13	-0.06	0.00	-0.01	-0.55
**2000**	11.C	Proportion of urban population living in slums (%)	-0.51	0.16	0.21	0.39	1.16
**2000**	11.D	Satisfaction with public transport (%)	-0.19	0.30	0.15	-0.09	-0.29
**2000**	12.A	Electronic waste (kg/capita)	1.07	0.22	0.02	-0.59	-1.53
**2000**	12.B	Exports of plastic waste (kg/capita)	0.79	0.34	0.10	-0.53	-1.19
**2000**	12.C	Municipal solid waste (kg/capita/day)	0.70	0.15	-0.03	-0.32	-1.58
**2000**	12.D	Nitrogen emissions embodied in imports (kg/capita)	0.89	0.11	0.08	-0.32	-1.45
**2000**	12.E	Production-based nitrogen emissions (kg/capita)	0.96	0.17	0.08	-0.42	-1.58
**2000**	12.F	SO2 emissions embodied in imports (kg/capita)	0.64	0.16	0.02	-0.25	-1.59
**2000**	12.G	Production-based SO2 emissions (kg/capita)	0.52	0.14	-0.01	-0.15	-1.65
**2000**	13.A	CO2/emissions embodied in fossil fuel exports (kg/capita)	0.67	0.07	0.12	-0.09	-0.91
**2000**	13.B	CO2 emissions from fossil fuel combustion and cement production (tCO2/capita)	0.75	0.05	-0.01	-0.43	-1.66
**2000**	13.C	CO2 emissions embodied in imports (tCO2/capita)	0.96	0.19	0.11	-0.51	-1.50
**2000**	14.A	Marine biodiversity threats embodied in imports (per million population)	0.56	0.17	-0.04	-0.19	-1.33

Year	SDG indicator	Indicator name	RCA in Stage 1	RCA in Stage 2	RCA in Stage 3	RCA in Stage 4	GSI
**2000**	14.B	Ocean Health Index: Clean Waters score (worst 0-100 best)	-0.08	0.35	0.27	0.43	0.65
**2000**	14.C	Mean area that is protected in marine sites important to biodiversity (%)	0.69	-0.11	0.20	0.51	0.02
**2000**	14.D	Fish caught that are then discarded (%)	1.08	0.44	0.29	-0.15	-0.43
**2000**	14.E	Fish caught from overexploited or collapsed stocks (% of total catch)	1.49	0.88	0.57	0.18	-0.19
**2000**	14.F	Fish caught by trawling or dredging (%)	0.83	0.45	0.28	0.00	-0.37
**2000**	15.A	Terrestrial and freshwater biodiversity threats embodied in imports (per million population)	0.56	0.16	-0.06	-0.32	-1.58
**2000**	15.B	Mean area that is protected in freshwater sites important to biodiversity (%)	0.39	-0.04	-0.05	0.07	-0.48
**2000**	15.C	Mean area that is protected in terrestrial sites important to biodiversity (%)	0.52	-0.15	-0.25	-0.02	-0.53
**2000**	15.D	Permanent deforestation (% of forest area, 3-year average)	0.39	0.11	0.08	0.01	-0.64
**2000**	15.E	Red List Index of species survival (worst 0-1 best)	0.43	0.00	-0.09	-0.13	-1.02
**2000**	16.A	Timeliness of administrative proceedings (worst 0 - 1 best)	0.05	0.14	0.07	0.42	0.20
**2000**	16.B	Children involved in child labor (% of population aged 5 to 14)	0.03	0.48	0.56	0.50	0.48
**2000**	16.C	Corruption Perceptions Index (worst 0-100 best)	-0.30	-0.07	-0.13	0.35	0.69
**2000**	16.D	Unsentenced detainees (% of prison population)	-0.01	0.12	0.22	0.12	0.27
**2000**	16.E	Expropriations are lawful and adequately compensated (worst 0 - 1 best)	-0.23	-0.03	-0.06	0.74	0.60
**2000**	16.F	Homicides (per 100,000 population)	0.50	0.12	0.04	0.02	0.10
**2000**	16.G	Access to and affordability of justice (worst 0–1 best)	0.26	0.15	0.20	0.21	0.13
**2000**	16.H	Press Freedom Index (worst 0-100 best)	0.22	-0.08	-0.17	0.12	-0.15
**2000**	16.I	Population who feel safe walking alone at night in the city or area where they live (%)	0.35	0.10	-0.06	-0.08	-0.28
**2000**	16.J	Birth registrations with civil authority (% of children under age 5)	-0.10	0.13	0.14	-0.01	-0.39
**2000**	16.K	Exports of major conventional weapons (TIV constant million USD per 100,000 population)	0.52	0.13	-0.02	-0.33	-1.60
**2000**	17.A	Corporate Tax Haven Score (best 0-100 worst)	0.57	0.13	-0.04	-0.32	-1.37
**2000**	17.B	Government spending on health and education (% of GDP)	-0.10	-0.02	-0.05	0.19	0.54
**2000**	17.C	Statistical Performance Index (worst 0-100 best)	-0.20	-0.18	0.00	0.26	0.61

Supplementary Table 15 | The changes in revealed comparative advantage (RCA) of SDG indicators for each sustainability stage in 2015.

Year	SDG indicator	Indicator name	RCA in Stage 1	RCA in Stage 2	RCA in Stage 3	RCA in Stage 4	GSI
**2015**	1.A	Poverty headcount ratio at $3.65/day (2017 PPP, %)	-0.83	0.07	0.48	0.39	1.13
**2015**	1.B	Poverty headcount ratio at $2.15/day (2017 PPP, %)	-0.12	0.22	0.22	0.06	0.36
**2015**	2.A	Cereal yield (tonnes per hectare of harvested land)	-0.46	0.05	-0.03	0.31	0.67
**2015**	2.B	Prevalence of obesity, BMI ≥ 30 (% of adult population)	1.00	0.03	-0.17	-0.41	-1.82
**2015**	2.C	Exports of hazardous pesticides (tonnes per million population)	0.96	0.44	0.25	0.12	0.21
**2015**	2.D	Sustainable Nitrogen Management Index (best 0-1.41 worst)	-0.05	-0.04	0.02	0.00	-0.63
**2015**	2.E	Prevalence of stunting in children under 5 years of age (%)	-0.32	0.02	0.11	0.13	1.13
**2015**	2.F	Human Trophic Level (best 2-3 worst)	1.26	0.30	-0.14	-0.73	-1.70
**2015**	2.G	Prevalence of undernourishment (%)	-0.14	0.14	0.15	0.04	0.60
**2015**	2.H	Prevalence of wasting in children under 5 years of age (%)	-0.11	0.02	0.02	0.07	0.51
**2015**	3.A	Births attended by skilled health personnel (%)	-0.19	0.12	0.14	0.01	0.10
**2015**	3.B	Adolescent fertility rate (births per 1,000 females aged 15 to 19)	-0.45	0.03	0.13	0.16	0.94
**2015**	3.C	New HIV infections (per 1,000 uninfected population)	0.56	0.33	0.31	0.16	-0.24
**2015**	3.D	Life expectancy at birth (years)	-0.42	0.02	0.12	0.15	1.20
**2015**	3.E	Maternal mortality rate (per 100,000 live births)	-0.29	0.13	0.11	-0.01	-0.11
**2015**	3.F	Age-standardized death rate due to cardiovascular disease, cancer, diabetes, or chronic respiratory disease in adults aged 30–70 years (%)	-0.23	-0.11	0.03	0.19	0.65
**2015**	3.G	Neonatal mortality rate (per 1,000 live births)	-0.50	0.06	0.13	0.15	1.33
**2015**	3.H	Age-standardized death rate attributable to household air pollution and ambient air pollution (per 100,000 population)	-0.12	0.02	0.05	0.02	0.73
**2015**	3.I	Subjective well-being (average ladder score, worst 0-10 best)	-0.41	0.07	0.07	0.27	1.02
**2015**	3.J	Incidence of tuberculosis (per 100,000 population)	0.01	-0.04	0.07	-0.02	-0.24
**2015**	3.K	Traffic deaths (per 100,000 population)	-0.45	-0.04	0.05	0.30	0.97
**2015**	3.L	Mortality rate, under-5 (per 1,000 live births)	-0.33	0.12	0.11	0.03	0.61
**2015**	3.M	Universal health coverage (UHC) index of service coverage (worst 0-100 best)	-0.80	-0.02	0.20	0.33	1.31
**2015**	3.N	Surviving infants who received 2 WHO-recommended vaccines (%)	-0.14	0.14	0.04	-0.05	-0.55
**2015**	4.A	Participation rate in pre-primary organized learning (% of children aged 4 to 6)	-0.57	0.07	0.22	0.41	1.11
**2015**	4.B	Literacy rate (% of population aged 15 to 24)	-0.10	0.36	0.35	0.21	-0.21
**2015**	4.C	Net primary enrollment rate (%)	-0.03	0.16	0.10	-0.03	-0.51
**2015**	4.D	Lower secondary completion rate (%)	-0.50	0.15	0.18	0.13	1.03
**2015**	5.A	Ratio of female-to-male mean years of education received (%)	-0.25	0.16	0.08	0.00	0.04
**2015**	5.B	Demand for family planning satisfied by modern methods (% of females aged 15 to 49)	-0.25	0.12	0.03	0.12	0.49
**2015**	5.C	Ratio of female-to-male labor force participation rate (%)	0.43	-0.15	-0.09	-0.05	-1.00
**2015**	5.D	Seats held by women in national parliament (%)	0.20	-0.10	-0.10	0.04	-0.19
**2015**	6.A	Freshwater withdrawal (% of available freshwater resources)	0.49	-0.06	-0.13	-0.09	-0.98
**2015**	6.B	Population using at least basic sanitation services (%)	-0.52	0.10	0.20	0.09	1.08
**2015**	6.C	Scarce water consumption embodied in imports (m3 H2O eq/capita)	0.74	0.07	0.03	-0.27	-1.64
**2015**	6.D	Anthropogenic wastewater that receives treatment (%)	-0.79	-0.56	-0.51	1.30	1.67

Year	SDG indicator	Indicator name	RCA in Stage 1	RCA in Stage 2	RCA in Stage 3	RCA in Stage 4	GSI
**2015**	6.E	Population using at least basic drinking water services (%)	-0.40	0.15	0.13	0.03	0.64
**2015**	7.A	Population with access to clean fuels and technology for cooking (%)	-0.70	0.10	0.22	0.23	1.15
**2015**	7.B	CO2 emissions from fuel combustion per total electricity output (MtCO2 TWh)	0.13	0.06	0.02	-0.11	-1.03
**2015**	7.C	Population with access to electricity (%)	-0.45	0.17	0.18	0.02	0.35
**2015**	7.D	Renewable energy share in total final energy consumption (%)	-0.02	-0.28	-0.11	0.32	-0.08
**2015**	8.A	Adults with an account at a bank or other financial institution or with a mobile-money-service provider (% of population aged 15 or over)	-0.60	0.01	0.02	0.52	1.38
**2015**	8.B	Adjusted GDP growth (%)	-0.02	0.07	-0.03	0.02	-0.27
**2015**	8.C	Fatal work-related accidents embodied in imports (per 100,000 population)	0.48	0.14	0.01	-0.14	-1.60
**2015**	8.D	Victims of modern slavery embodied in imports (per 100,000 population)	0.67	0.13	0.03	-0.26	-1.58
**2015**	8.E	Fundamental labor rights are effectively guaranteed (worst 0–1 best)	0.06	0.03	0.08	0.47	0.47
**2015**	8.F	Victims of modern slavery (per 1,000 population)	0.14	0.16	0.13	0.05	-0.02
**2015**	8.G	Unemployment rate (% of total labor force, ages 15+)	0.36	0.08	-0.09	-0.19	-1.41
**2015**	9.A	Articles published in academic journals (per 1,000 population)	-0.92	-0.51	-0.24	1.15	1.93
**2015**	9.B	Population using the internet (%)	-0.71	-0.04	0.11	0.37	1.46
**2015**	9.C	Logistics Performance Index: Quality of trade and transport-related infrastructure (worst 1-5 best)	-0.26	-0.04	-0.04	0.31	1.15
**2015**	9.D	Mobile broadband subscriptions (per 100 population)	-0.66	0.01	0.08	0.32	1.11
**2015**	9.E	Expenditure on research and development (% of GDP)	-0.67	-0.53	-0.06	1.05	1.58
**2015**	9.F	Rural population with access to all-season roads (%)	-0.31	0.03	0.08	0.13	0.99
**2015**	9.G	The Times Higher Education Universities Ranking: Average score of top 3 universities (worst 0-100 best)	-0.86	-0.44	0.02	0.80	1.03
**2015**	10.A	Gini coefficient	0.31	0.05	0.05	0.11	-0.25
**2015**	10.B	Palma ratio	-0.03	-0.13	0.02	0.43	0.53
**2015**	11.A	Access to improved water source, piped (% of urban population)	0.09	0.13	0.17	0.06	0.01
**2015**	11.B	Annual mean concentration of particulate matter of less than 2.5 microns in diameter (PM2.5) (μg/m³)	0.09	-0.11	0.03	0.03	-0.10
**2015**	11.C	Proportion of urban population living in slums (%)	-0.27	0.14	0.24	0.23	1.26
**2015**	11.D	Satisfaction with public transport (%)	-0.27	0.28	0.15	-0.04	0.06
**2015**	12.A	Electronic waste (kg/capita)	1.01	0.19	0.03	-0.59	-1.60
**2015**	12.B	Exports of plastic waste (kg/capita)	0.75	0.34	0.10	-0.54	-1.23
**2015**	12.C	Municipal solid waste (kg/capita/day)	0.66	0.13	-0.01	-0.33	-1.65
**2015**	12.D	Nitrogen emissions embodied in imports (kg/capita)	0.84	0.11	0.08	-0.31	-1.46
**2015**	12.E	Production-based nitrogen emissions (kg/capita)	0.89	0.10	0.05	-0.33	-1.53
**2015**	12.F	SO2 emissions embodied in imports (kg/capita)	0.61	0.14	0.03	-0.23	-1.61
**2015**	12.G	Production-based SO2 emissions (kg/capita)	0.49	0.12	0.00	-0.13	-1.70
**2015**	13.A	CO2/emissions embodied in fossil fuel exports (kg/capita)	0.63	0.09	0.12	-0.07	-0.88
**2015**	13.B	CO2 emissions from fossil fuel combustion and cement production (tCO2/capita)	0.72	-0.02	-0.05	-0.35	-1.74
**2015**	13.C	CO2 emissions embodied in imports (tCO2/capita)	0.90	0.12	0.12	-0.46	-1.55
**2015**	14.A	Marine biodiversity threats embodied in imports (per million population)	0.52	0.09	0.01	-0.17	-1.37

Year	SDG indicator	Indicator name	RCA in Stage 1	RCA in Stage 2	RCA in Stage 3	RCA in Stage 4	GSI
**2015**	14.B	Ocean Health Index: Clean Waters score (worst 0-100 best)	0.21	0.54	0.22	0.21	0.14
**2015**	14.C	Mean area that is protected in marine sites important to biodiversity (%)	0.25	0.12	0.07	0.71	0.28
**2015**	14.D	Fish caught that are then discarded (%)	1.08	0.42	0.09	0.00	-0.48
**2015**	14.E	Fish caught from overexploited or collapsed stocks (% of total catch)	1.61	0.82	0.55	0.17	-0.12
**2015**	14.F	Fish caught by trawling or dredging (%)	1.02	0.41	0.21	0.00	-0.35
**2015**	15.A	Terrestrial and freshwater biodiversity threats embodied in imports (per million population)	0.54	0.08	-0.02	-0.30	-1.62
**2015**	15.B	Mean area that is protected in freshwater sites important to biodiversity (%)	0.25	-0.05	-0.12	0.25	-0.20
**2015**	15.C	Mean area that is protected in terrestrial sites important to biodiversity (%)	0.22	-0.08	-0.35	0.21	-0.23
**2015**	15.D	Permanent deforestation (% of forest area, 3-year average)	0.42	0.05	0.07	0.02	-0.36
**2015**	15.E	Red List Index of species survival (worst 0-1 best)	0.44	-0.05	-0.13	-0.09	-0.66
**2015**	16.A	Timeliness of administrative proceedings (worst 0 - 1 best)	0.07	0.11	0.05	0.45	0.34
**2015**	16.B	Children involved in child labor (% of population aged 5 to 14)	-0.03	0.46	0.58	0.53	0.55
**2015**	16.C	Corruption Perceptions Index (worst 0-100 best)	-0.35	-0.08	-0.14	0.42	0.87
**2015**	16.D	Unsentenced detainees (% of prison population)	0.03	0.03	0.22	0.17	0.58
**2015**	16.E	Expropriations are lawful and adequately compensated (worst 0 - 1 best)	-0.32	-0.06	-0.05	0.80	0.73
**2015**	16.F	Homicides (per 100,000 population)	0.41	0.07	0.07	0.06	0.26
**2015**	16.G	Access to and affordability of justice (worst 0–1 best)	0.23	0.12	0.19	0.26	0.43
**2015**	16.H	Press Freedom Index (worst 0-100 best)	0.13	-0.08	-0.17	0.18	0.05
**2015**	16.I	Population who feel safe walking alone at night in the city or area where they live (%)	0.09	0.12	-0.06	0.07	0.05
**2015**	16.J	Birth registrations with civil authority (% of children under age 5)	-0.16	0.15	0.15	0.00	-0.11
**2015**	16.K	Exports of major conventional weapons (TIV constant million USD per 100,000 population)	0.49	0.11	-0.03	-0.31	-1.61
**2015**	17.A	Corporate Tax Haven Score (best 0-100 worst)	0.54	0.10	0.00	-0.33	-1.42
**2015**	17.B	Government spending on health and education (% of GDP)	-0.12	-0.05	-0.02	0.19	0.44
**2015**	17.C	Statistical Performance Index (worst 0-100 best)	-0.28	-0.16	0.02	0.29	0.91

Supplementary Table 16 | The changes in revealed comparative advantage (RCA) of SDG indicators for each sustainability stage in 2022.

Year	SDG indicator	Indicator name	RCA in Stage 1	RCA in Stage 2	RCA in Stage 3	RCA in Stage 4	GSI
**2015**	1.A	Poverty headcount ratio at $3.65/day (2017 PPP, %)	-0.83	0.07	0.48	0.39	1.13
**2015**	1.B	Poverty headcount ratio at $2.15/day (2017 PPP, %)	-0.12	0.22	0.22	0.06	0.36
**2015**	2.A	Cereal yield (tonnes per hectare of harvested land)	-0.46	0.05	-0.03	0.31	0.67
**2015**	2.B	Prevalence of obesity, BMI ≥ 30 (% of adult population)	1.00	0.03	-0.17	-0.41	-1.82
**2015**	2.C	Exports of hazardous pesticides (tonnes per million population)	0.96	0.44	0.25	0.12	0.21
**2015**	2.D	Sustainable Nitrogen Management Index (best 0-1.41 worst)	-0.05	-0.04	0.02	0.00	-0.63
**2015**	2.E	Prevalence of stunting in children under 5 years of age (%)	-0.32	0.02	0.11	0.13	1.13
**2015**	2.F	Human Trophic Level (best 2-3 worst)	1.26	0.30	-0.14	-0.73	-1.70
**2015**	2.G	Prevalence of undernourishment (%)	-0.14	0.14	0.15	0.04	0.60
**2015**	2.H	Prevalence of wasting in children under 5 years of age (%)	-0.11	0.02	0.02	0.07	0.51
**2015**	3.A	Births attended by skilled health personnel (%)	-0.19	0.12	0.14	0.01	0.10
**2015**	3.B	Adolescent fertility rate (births per 1,000 females aged 15 to 19)	-0.45	0.03	0.13	0.16	0.94
**2015**	3.C	New HIV infections (per 1,000 uninfected population)	0.56	0.33	0.31	0.16	-0.24
**2015**	3.D	Life expectancy at birth (years)	-0.42	0.02	0.12	0.15	1.20
**2015**	3.E	Maternal mortality rate (per 100,000 live births)	-0.29	0.13	0.11	-0.01	-0.11
**2015**	3.F	Age-standardized death rate due to cardiovascular disease, cancer, diabetes, or chronic respiratory disease in adults aged 30–70 years (%)	-0.23	-0.11	0.03	0.19	0.65
**2015**	3.G	Neonatal mortality rate (per 1,000 live births)	-0.50	0.06	0.13	0.15	1.33
**2015**	3.H	Age-standardized death rate attributable to household air pollution and ambient air pollution (per 100,000 population)	-0.12	0.02	0.05	0.02	0.73
**2015**	3.I	Subjective well-being (average ladder score, worst 0-10 best)	-0.41	0.07	0.07	0.27	1.02
**2015**	3.J	Incidence of tuberculosis (per 100,000 population)	0.01	-0.04	0.07	-0.02	-0.24
**2015**	3.K	Traffic deaths (per 100,000 population)	-0.45	-0.04	0.05	0.30	0.97
**2015**	3.L	Mortality rate, under-5 (per 1,000 live births)	-0.33	0.12	0.11	0.03	0.61
**2015**	3.M	Universal health coverage (UHC) index of service coverage (worst 0-100 best)	-0.80	-0.02	0.20	0.33	1.31
**2015**	3.N	Surviving infants who received 2 WHO-recommended vaccines (%)	-0.14	0.14	0.04	-0.05	-0.55
**2015**	4.A	Participation rate in pre-primary organized learning (% of children aged 4 to 6)	-0.57	0.07	0.22	0.41	1.11
**2015**	4.B	Literacy rate (% of population aged 15 to 24)	-0.10	0.36	0.35	0.21	-0.21
**2015**	4.C	Net primary enrollment rate (%)	-0.03	0.16	0.10	-0.03	-0.51
**2015**	4.D	Lower secondary completion rate (%)	-0.50	0.15	0.18	0.13	1.03
**2015**	5.A	Ratio of female-to-male mean years of education received (%)	-0.25	0.16	0.08	0.00	0.04
**2015**	5.B	Demand for family planning satisfied by modern methods (% of females aged 15 to 49)	-0.25	0.12	0.03	0.12	0.49
**2015**	5.C	Ratio of female-to-male labor force participation rate (%)	0.43	-0.15	-0.09	-0.05	-1.00
**2015**	5.D	Seats held by women in national parliament (%)	0.20	-0.10	-0.10	0.04	-0.19
**2015**	6.A	Freshwater withdrawal (% of available freshwater resources)	0.49	-0.06	-0.13	-0.09	-0.98
**2015**	6.B	Population using at least basic sanitation services (%)	-0.52	0.10	0.20	0.09	1.08
**2015**	6.C	Scarce water consumption embodied in imports (m3 H2O eq/capita)	0.74	0.07	0.03	-0.27	-1.64
**2015**	6.D	Anthropogenic wastewater that receives treatment (%)	-0.79	-0.56	-0.51	1.30	1.67

Year	SDG indicator	Indicator name	RCA in Stage 1	RCA in Stage 2	RCA in Stage 3	RCA in Stage 4	GSI
**2015**	6.E	Population using at least basic drinking water services (%)	-0.40	0.15	0.13	0.03	0.64
**2015**	7.A	Population with access to clean fuels and technology for cooking (%)	-0.70	0.10	0.22	0.23	1.15
**2015**	7.B	CO2 emissions from fuel combustion per total electricity output (MtCO2 TWh)	0.13	0.06	0.02	-0.11	-1.03
**2015**	7.C	Population with access to electricity (%)	-0.45	0.17	0.18	0.02	0.35
**2015**	7.D	Renewable energy share in total final energy consumption (%)	-0.02	-0.28	-0.11	0.32	-0.08
**2015**	8.A	Adults with an account at a bank or other financial institution or with a mobile-money-service provider (% of population aged 15 or over)	-0.60	0.01	0.02	0.52	1.38
**2015**	8.B	Adjusted GDP growth (%)	-0.02	0.07	-0.03	0.02	-0.27
**2015**	8.C	Fatal work-related accidents embodied in imports (per 100,000 population)	0.48	0.14	0.01	-0.14	-1.60
**2015**	8.D	Victims of modern slavery embodied in imports (per 100,000 population)	0.67	0.13	0.03	-0.26	-1.58
**2015**	8.E	Fundamental labor rights are effectively guaranteed (worst 0–1 best)	0.06	0.03	0.08	0.47	0.47
**2015**	8.F	Victims of modern slavery (per 1,000 population)	0.14	0.16	0.13	0.05	-0.02
**2015**	8.G	Unemployment rate (% of total labor force, ages 15+)	0.36	0.08	-0.09	-0.19	-1.41
**2015**	9.A	Articles published in academic journals (per 1,000 population)	-0.92	-0.51	-0.24	1.15	1.93
**2015**	9.B	Population using the internet (%)	-0.71	-0.04	0.11	0.37	1.46
**2015**	9.C	Logistics Performance Index: Quality of trade and transport-related infrastructure (worst 1-5 best)	-0.26	-0.04	-0.04	0.31	1.15
**2015**	9.D	Mobile broadband subscriptions (per 100 population)	-0.66	0.01	0.08	0.32	1.11
**2015**	9.E	Expenditure on research and development (% of GDP)	-0.67	-0.53	-0.06	1.05	1.58
**2015**	9.F	Rural population with access to all-season roads (%)	-0.31	0.03	0.08	0.13	0.99
**2015**	9.G	The Times Higher Education Universities Ranking: Average score of top 3 universities (worst 0-100 best)	-0.86	-0.44	0.02	0.80	1.03
**2015**	10.A	Gini coefficient	0.31	0.05	0.05	0.11	-0.25
**2015**	10.B	Palma ratio	-0.03	-0.13	0.02	0.43	0.53
**2015**	11.A	Access to improved water source, piped (% of urban population)	0.09	0.13	0.17	0.06	0.01
**2015**	11.B	Annual mean concentration of particulate matter of less than 2.5 microns in diameter (PM2.5) (μg/m³)	0.09	-0.11	0.03	0.03	-0.10
**2015**	11.C	Proportion of urban population living in slums (%)	-0.27	0.14	0.24	0.23	1.26
**2015**	11.D	Satisfaction with public transport (%)	-0.27	0.28	0.15	-0.04	0.06
**2015**	12.A	Electronic waste (kg/capita)	1.01	0.19	0.03	-0.59	-1.60
**2015**	12.B	Exports of plastic waste (kg/capita)	0.75	0.34	0.10	-0.54	-1.23
**2015**	12.C	Municipal solid waste (kg/capita/day)	0.66	0.13	-0.01	-0.33	-1.65
**2015**	12.D	Nitrogen emissions embodied in imports (kg/capita)	0.84	0.11	0.08	-0.31	-1.46
**2015**	12.E	Production-based nitrogen emissions (kg/capita)	0.89	0.10	0.05	-0.33	-1.53
**2015**	12.F	SO2 emissions embodied in imports (kg/capita)	0.61	0.14	0.03	-0.23	-1.61
**2015**	12.G	Production-based SO2 emissions (kg/capita)	0.49	0.12	0.00	-0.13	-1.70
**2015**	13.A	CO2/emissions embodied in fossil fuel exports (kg/capita)	0.63	0.09	0.12	-0.07	-0.88
**2015**	13.B	CO2 emissions from fossil fuel combustion and cement production (tCO2/capita)	0.72	-0.02	-0.05	-0.35	-1.74
**2015**	13.C	CO2 emissions embodied in imports (tCO2/capita)	0.90	0.12	0.12	-0.46	-1.55
**2015**	14.A	Marine biodiversity threats embodied in imports (per million population)	0.52	0.09	0.01	-0.17	-1.37

Year	SDG indicator	Indicator name	RCA in Stage 1	RCA in Stage 2	RCA in Stage 3	RCA in Stage 4	GSI
**2015**	14.B	Ocean Health Index: Clean Waters score (worst 0-100 best)	0.21	0.54	0.22	0.21	0.14
**2015**	14.C	Mean area that is protected in marine sites important to biodiversity (%)	0.25	0.12	0.07	0.71	0.28
**2015**	14.D	Fish caught that are then discarded (%)	1.08	0.42	0.09	0.00	-0.48
**2015**	14.E	Fish caught from overexploited or collapsed stocks (% of total catch)	1.61	0.82	0.55	0.17	-0.12
**2015**	14.F	Fish caught by trawling or dredging (%)	1.02	0.41	0.21	0.00	-0.35
**2015**	15.A	Terrestrial and freshwater biodiversity threats embodied in imports (per million population)	0.54	0.08	-0.02	-0.30	-1.62
**2015**	15.B	Mean area that is protected in freshwater sites important to biodiversity (%)	0.25	-0.05	-0.12	0.25	-0.20
**2015**	15.C	Mean area that is protected in terrestrial sites important to biodiversity (%)	0.22	-0.08	-0.35	0.21	-0.23
**2015**	15.D	Permanent deforestation (% of forest area, 3-year average)	0.42	0.05	0.07	0.02	-0.36
**2015**	15.E	Red List Index of species survival (worst 0-1 best)	0.44	-0.05	-0.13	-0.09	-0.66
**2015**	16.A	Timeliness of administrative proceedings (worst 0 - 1 best)	0.07	0.11	0.05	0.45	0.34
**2015**	16.B	Children involved in child labor (% of population aged 5 to 14)	-0.03	0.46	0.58	0.53	0.55
**2015**	16.C	Corruption Perceptions Index (worst 0-100 best)	-0.35	-0.08	-0.14	0.42	0.87
**2015**	16.D	Unsentenced detainees (% of prison population)	0.03	0.03	0.22	0.17	0.58
**2015**	16.E	Expropriations are lawful and adequately compensated (worst 0 - 1 best)	-0.32	-0.06	-0.05	0.80	0.73
**2015**	16.F	Homicides (per 100,000 population)	0.41	0.07	0.07	0.06	0.26
**2015**	16.G	Access to and affordability of justice (worst 0–1 best)	0.23	0.12	0.19	0.26	0.43
**2015**	16.H	Press Freedom Index (worst 0-100 best)	0.13	-0.08	-0.17	0.18	0.05
**2015**	16.I	Population who feel safe walking alone at night in the city or area where they live (%)	0.09	0.12	-0.06	0.07	0.05
**2015**	16.J	Birth registrations with civil authority (% of children under age 5)	-0.16	0.15	0.15	0.00	-0.11
**2015**	16.K	Exports of major conventional weapons (TIV constant million USD per 100,000 population)	0.49	0.11	-0.03	-0.31	-1.61
**2015**	17.A	Corporate Tax Haven Score (best 0-100 worst)	0.54	0.10	0.00	-0.33	-1.42
**2015**	17.B	Government spending on health and education (% of GDP)	-0.12	-0.05	-0.02	0.19	0.44
**2015**	17.C	Statistical Performance Index (worst 0-100 best)	-0.28	-0.16	0.02	0.29	0.91

Supplementary Table 17 | The top 10 SDG indicators with the most increasement in revealed comparative advantage (RCA) for each sustainability stage in 2000.

	RCA increase from Stage 1 to Stage 2 in 2000			RCA increase from Stage 2 to Stage 3 in 2000		
**Rank**	**SDG indicators**	**Indicator name**	**RCA change**	**SDG indicators**	**Indicator name**	**RCA change**
**1**	7.A	Population with access to clean fuels and technology for cooking (%)	0.91	9.G	The Times Higher Education Universities Ranking: Average score of top 3 universities (worst 0-100 best)	0.72
**2**	3.L	Mortality rate, under-5 (per 1,000 live births)	0.89	1.A	Poverty headcount ratio at $3.65/day (2017 PPP, %)	0.55
**3**	7.C	Population with access to electricity (%)	0.86	7.D	Renewable energy share in total final energy consumption (%)	0.35
**4**	3.G	Neonatal mortality rate (per 1,000 live births)	0.85	14.C	Mean area that is protected in marine sites important to biodiversity (%)	0.31
**5**	3.D	Life expectancy at birth (years)	0.81	3.M	Universal health coverage (UHC) index of service coverage (worst 0-100 best)	0.30
**6**	4.D	Lower secondary completion rate (%)	0.81	6.B	Population using at least basic sanitation services (%)	0.25
**7**	1.A	Poverty headcount ratio at $3.65/day (2017 PPP, %)	0.80	2.E	Prevalence of stunting in children under 5 years of age (%)	0.23
**8**	3.E	Maternal mortality rate (per 100,000 live births)	0.78	9.E	Expenditure on research and development (% of GDP)	0.22
**9**	5.B	Demand for family planning satisfied by modern methods (% of females aged 15 to 49)	0.74	3.D	Life expectancy at birth (years)	0.20
**10**	6.E	Population using at least basic drinking water services (%)	0.71	4.D	Lower secondary completion rate (%)	0.20
	**RCA increase from Stage 3 to Stage 4 in 2000**			**RCA increase from Stage 1 to Stage 4 in 2000**		
**Rank**	**SDG indicators**	**Indicator name**	**RCA change**	**SDG indicators**	**Indicator name**	**RCA change**
**1**	9.A	Articles published in academic journals (per 1,000 population)	2.19	9.A	Articles published in academic journals (per 1,000 population)	2.56
**2**	9.B	Population using the internet (%)	2.10	9.B	Population using the internet (%)	2.55
**3**	6.D	Anthropogenic wastewater that receives treatment (%)	1.88	9.D	Mobile broadband subscriptions (per 100 population)	2.23
**4**	9.D	Mobile broadband subscriptions (per 100 population)	1.62	6.D	Anthropogenic wastewater that receives treatment (%)	2.20
**5**	9.E	Expenditure on research and development (% of GDP)	1.54	3.M	Universal health coverage (UHC) index of service coverage (worst 0-100 best)	1.91
**6**	3.M	Universal health coverage (UHC) index of service coverage (worst 0-100 best)	0.99	9.E	Expenditure on research and development (% of GDP)	1.82
**7**	16.E	Expropriations are lawful and adequately compensated (worst 0 - 1 best)	0.79	9.G	The Times Higher Education Universities Ranking: Average score of top 3 universities (worst 0-100 best)	1.58
**8**	9.G	The Times Higher Education Universities Ranking: Average score of top 3 universities (worst 0-100 best)	0.71	1.A	Poverty headcount ratio at $3.65/day (2017 PPP, %)	1.32
**9**	8.A	Adults with an account at a bank or other financial institution or with a mobile-money-service provider (% of population aged 15 or over)	0.68	8.A	Adults with an account at a bank or other financial institution or with a mobile-money-service provider (% of population aged 15 or over)	1.30
**10**	5.D	Seats held by women in national parliament (%)	0.52	3.G	Neonatal mortality rate (per 1,000 live births)	1.23

Supplementary Table 18 | The top 10 SDG indicators with the most decrease in revealed comparative advantage (RCA) for each sustainability stage in 2000.

	RCA decrease from Stage 1 to Stage 2 in 2000			RCA decrease from Stage 2 to Stage 3 in 2000		
**Rank**	**SDG indicators**	**Indicator name**	**RCA change**	**SDG indicators**	**Indicator name**	**RCA change**
**1**	2.F	Human Trophic Level (best 2-3 worst)	-1.09	2.F	Human Trophic Level (best 2-3 worst)	-0.42
**2**	12.A	Electronic waste (kg/capita)	-0.85	14.E	Fish caught from overexploited or collapsed stocks (% of total catch)	-0.31
**3**	2.B	Prevalence of obesity, BMI ≥ 30 (% of adult population)	-0.80	8.G	Unemployment rate (% of total labor force, ages 15+)	-0.30
**4**	14.C	Mean area that is protected in marine sites important to biodiversity (%)	-0.79	12.B	Exports of plastic waste (kg/capita)	-0.25
**5**	5.C	Ratio of female-to-male labor force participation rate (%)	-0.79	15.A	Terrestrial and freshwater biodiversity threats embodied in imports (per million population)	-0.22
**6**	12.E	Production-based nitrogen emissions (kg/capita)	-0.79	14.A	Marine biodiversity threats embodied in imports (per million population)	-0.20
**7**	12.D	Nitrogen emissions embodied in imports (kg/capita)	-0.78	12.A	Electronic waste (kg/capita)	-0.20
**8**	13.C	CO2 emissions embodied in imports (tCO2/capita)	-0.77	2.B	Prevalence of obesity, BMI ≥ 30 (% of adult population)	-0.18
**9**	13.B	CO2 emissions from fossil fuel combustion and cement production (tCO2/capita)	-0.70	12.C	Municipal solid waste (kg/capita/day)	-0.17
**10**	6.A	Freshwater withdrawal (% of available freshwater resources)	-0.70	17.A	Corporate Tax Haven Score (best 0-100 worst)	-0.17

	RCA decrease from Stage 3 to Stage 4 in 2000			RCA decrease from Stage 1 to Stage 4 in 2000		
**Rank**	**SDG indicators**	**Indicator name**	**RCA change**	**SDG indicators**	**Indicator name**	**RCA change**
**1**	12.B	Exports of plastic waste (kg/capita)	-0.63	2.F	Human Trophic Level (best 2-3 worst)	-2.02
**2**	12.A	Electronic waste (kg/capita)	-0.62	12.A	Electronic waste (kg/capita)	-1.66
**3**	13.C	CO2 emissions embodied in imports (tCO2/capita)	-0.62	13.C	CO2 emissions embodied in imports (tCO2/capita)	-1.47
**4**	2.F	Human Trophic Level (best 2-3 worst)	-0.51	12.E	Production-based nitrogen emissions (kg/capita)	-1.38
**5**	12.E	Production-based nitrogen emissions (kg/capita)	-0.49	12.B	Exports of plastic waste (kg/capita)	-1.32
**6**	14.D	Fish caught that are then discarded (%)	-0.45	14.E	Fish caught from overexploited or collapsed stocks (% of total catch)	-1.31
**7**	13.B	CO2 emissions from fossil fuel combustion and cement production (tCO2/capita)	-0.42	14.D	Fish caught that are then discarded (%)	-1.23
**8**	12.D	Nitrogen emissions embodied in imports (kg/capita)	-0.41	12.D	Nitrogen emissions embodied in imports (kg/capita)	-1.22
**9**	14.E	Fish caught from overexploited or collapsed stocks (% of total catch)	-0.39	2.B	Prevalence of obesity, BMI ≥ 30 (% of adult population)	-1.21
**10**	16.K	Exports of major conventional weapons (TIV constant million USD per 100,000 population)	-0.32	13.B	CO2 emissions from fossil fuel combustion and cement production (tCO2/capita)	-1.18

Supplementary Table 19 | The top 10 SDG indicators with the most increasement in revealed comparative advantage (RCA) for each sustainability stage in 2015.

	RCA increase from Stage 1 to Stage 2 in 2015			RCA increase from Stage 2 to Stage 3 in 2015		
**Rank**	**SDG indicators**	**Indicator name**	**RCA change**	**SDG indicators**	**Indicator name**	**RCA change**
**1**	1.A	Poverty headcount ratio at $3.65/day (2017 PPP, %)	0.90	9.E	Expenditure on research and development (% of GDP)	0.46
**2**	7.A	Population with access to clean fuels and technology for cooking (%)	0.80	9.G	The Times Higher Education Universities Ranking: Average score of top 3 universities (worst 0-100 best)	0.45
**3**	3.M	Universal health coverage (UHC) index of service coverage (worst 0-100 best)	0.79	1.A	Poverty headcount ratio at $3.65/day (2017 PPP, %)	0.41
**4**	9.B	Population using the internet (%)	0.67	9.A	Articles published in academic journals (per 1,000 population)	0.27
**5**	9.D	Mobile broadband subscriptions (per 100 population)	0.67	3.M	Universal health coverage (UHC) index of service coverage (worst 0-100 best)	0.22
**6**	4.D	Lower secondary completion rate (%)	0.65	16.D	Unsentenced detainees (% of prison population)	0.19
**7**	4.A	Participation rate in pre-primary organized learning (% of children aged 4 to 6)	0.64	17.C	Statistical Performance Index (worst 0-100 best)	0.18
**8**	7.C	Population with access to electricity (%)	0.62	7.D	Renewable energy share in total final energy consumption (%)	0.17
**9**	6.B	Population using at least basic sanitation services (%)	0.62	9.B	Population using the internet (%)	0.15
**10**	8.A	Adults with an account at a bank or other financial institution or with a mobile-money-service provider (% of population aged 15 or over)	0.60	4.A	Participation rate in pre-primary organized learning (% of children aged 4 to 6)	0.15

	RCA increase from Stage 3 to Stage 4 in 2015			RCA increase from Stage 1 to Stage 4 in 2015		
**Rank**	**SDG indicators**	**Indicator name**	**RCA change**	**SDG indicators**	**Indicator name**	**RCA change**
**1**	6.D	Anthropogenic wastewater that receives treatment (%)	1.81	6.D	Anthropogenic wastewater that receives treatment (%)	2.09
**2**	9.A	Articles published in academic journals (per 1,000 population)	1.38	9.A	Articles published in academic journals (per 1,000 population)	2.07
**3**	9.E	Expenditure on research and development (% of GDP)	1.12	9.E	Expenditure on research and development (% of GDP)	1.72
**4**	16.E	Expropriations are lawful and adequately compensated (worst 0 - 1 best)	0.85	9.G	The Times Higher Education Universities Ranking: Average score of top 3 universities (worst 0-100 best)	1.66
**5**	9.G	The Times Higher Education Universities Ranking: Average score of top 3 universities (worst 0-100 best)	0.79	1.A	Poverty headcount ratio at $3.65/day (2017 PPP, %)	1.21
**6**	14.C	Mean area that is protected in marine sites important to biodiversity (%)	0.64	3.M	Universal health coverage (UHC) index of service coverage (worst 0-100 best)	1.14
**7**	15.C	Mean area that is protected in terrestrial sites important to biodiversity (%)	0.56	16.E	Expropriations are lawful and adequately compensated (worst 0 - 1 best)	1.12
**8**	16.C	Corruption Perceptions Index (worst 0-100 best)	0.55	8.A	Adults with an account at a bank or other financial institution or with a mobile-money-service provider (% of population aged 15 or over)	1.12
**9**	8.A	Adults with an account at a bank or other financial institution or with a mobile-money-service provider (% of population aged 15 or over)	0.50	9.B	Population using the internet (%)	1.08
**10**	7.D	Renewable energy share in total final energy consumption (%)	0.43	4.A	Participation rate in pre-primary organized learning (% of children aged 4 to 6)	0.98

Supplementary Table 20 | The top 10 SDG indicators with the most decrease in revealed comparative advantage (RCA) for each sustainability stage in 2015.

	RCA decrease from Stage 1 to Stage 2 in 2015			RCA decrease from Stage 2 to Stage 3 in 2015		
**Rank**	**SDG indicators**	**Indicator name**	**RCA change**	**SDG indicators**	**Indicator name**	**RCA change**
**1**	2.B	Prevalence of obesity, BMI ≥ 30 (% of adult population)	-0.97	2.F	Human Trophic Level (best 2-3 worst)	-0.44
**2**	2.F	Human Trophic Level (best 2-3 worst)	-0.96	14.D	Fish caught that are then discarded (%)	-0.33
**3**	12.A	Electronic waste (kg/capita)	-0.82	14.B	Ocean Health Index: Clean Waters score (worst 0-100 best)	-0.31
**4**	14.E	Fish caught from overexploited or collapsed stocks (% of total catch)	-0.79	14.E	Fish caught from overexploited or collapsed stocks (% of total catch)	-0.27
**5**	12.E	Production-based nitrogen emissions (kg/capita)	-0.79	15.C	Mean area that is protected in terrestrial sites important to biodiversity (%)	-0.27
**6**	13.C	CO2 emissions embodied in imports (tCO2/capita)	-0.78	12.B	Exports of plastic waste (kg/capita)	-0.25
**7**	13.B	CO2 emissions from fossil fuel combustion and cement production (tCO2/capita)	-0.74	14.F	Fish caught by trawling or dredging (%)	-0.21
**8**	12.D	Nitrogen emissions embodied in imports (kg/capita)	-0.73	2.B	Prevalence of obesity, BMI ≥ 30 (% of adult population)	-0.20
**9**	6.C	Scarce water consumption embodied in imports (m3 H2O eq/capita)	-0.66	2.C	Exports of hazardous pesticides (tonnes per million population)	-0.19
**10**	14.D	Fish caught that are then discarded (%)	-0.66	16.I	Population who feel safe walking alone at night in the city or area where they live (%)	-0.18

	RCA decrease from Stage 3 to Stage 4 in 2015			RCA decrease from Stage 1 to Stage 4 in 2015		
**Rank**	**SDG indicators**	**Indicator name**	**RCA change**	**SDG indicators**	**Indicator name**	**RCA change**
**1**	12.B	Exports of plastic waste (kg/capita)	-0.64	2.F	Human Trophic Level (best 2-3 worst)	-1.99
**2**	12.A	Electronic waste (kg/capita)	-0.62	12.A	Electronic waste (kg/capita)	-1.59
**3**	2.F	Human Trophic Level (best 2-3 worst)	-0.59	14.E	Fish caught from overexploited or collapsed stocks (% of total catch)	-1.44
**4**	13.C	CO2 emissions embodied in imports (tCO2/capita)	-0.58	2.B	Prevalence of obesity, BMI ≥ 30 (% of adult population)	-1.41
**5**	12.D	Nitrogen emissions embodied in imports (kg/capita)	-0.40	13.C	CO2 emissions embodied in imports (tCO2/capita)	-1.36
**6**	12.E	Production-based nitrogen emissions (kg/capita)	-0.38	12.B	Exports of plastic waste (kg/capita)	-1.29
**7**	14.E	Fish caught from overexploited or collapsed stocks (% of total catch)	-0.38	12.E	Production-based nitrogen emissions (kg/capita)	-1.22
**8**	17.A	Corporate Tax Haven Score (best 0-100 worst)	-0.33	12.D	Nitrogen emissions embodied in imports (kg/capita)	-1.16
**9**	12.C	Municipal solid waste (kg/capita/day)	-0.32	14.D	Fish caught that are then discarded (%)	-1.08
**10**	13.B	CO2 emissions from fossil fuel combustion and cement production (tCO2/capita)	-0.30	13.B	CO2 emissions from fossil fuel combustion and cement production (tCO2/capita)	-1.07

Supplementary Table 21 | The top 10 SDG indicators with the most increasement in revealed comparative advantage (RCA) for each sustainability stage in 2022.

	RCA increase from Stage 1 to Stage 2 in 2022			RCA increase from Stage 2 to Stage 3 in 2022		
**Rank**	**SDG indicators**	**Indicator name**	**RCA change**	**SDG indicators**	**Indicator name**	**RCA change**
**1**	1.A	Poverty headcount ratio at $3.65/day (2017 PPP, %)	0.88	9.E	Expenditure on research and development (% of GDP)	0.48
**2**	7.A	Population with access to clean fuels and technology for cooking (%)	0.76	9.G	The Times Higher Education Universities Ranking: Average score of top 3 universities (worst 0-100 best)	0.33
**3**	3.M	Universal health coverage (UHC) index of service coverage (worst 0-100 best)	0.72	10.B	Palma ratio	0.32
**4**	4.D	Lower secondary completion rate (%)	0.59	6.D	Anthropogenic wastewater that receives treatment (%)	0.27
**5**	9.A	Articles published in academic journals (per 1,000 population)	0.58	9.A	Articles published in academic journals (per 1,000 population)	0.24
**6**	6.B	Population using at least basic sanitation services (%)	0.58	1.A	Poverty headcount ratio at $3.65/day (2017 PPP, %)	0.23
**7**	3.I	Subjective well-being (average ladder score, worst 0-10 best)	0.55	16.D	Unsentenced detainees (% of prison population)	0.23
**8**	14.B	Ocean Health Index: Clean Waters score (worst 0-100 best)	0.55	7.D	Renewable energy share in total final energy consumption (%)	0.19
**9**	9.B	Population using the internet (%)	0.53	4.A	Participation rate in pre-primary organized learning (% of children aged 4 to 6)	0.19
**10**	9.D	Mobile broadband subscriptions (per 100 population)	0.53	17.C	Statistical Performance Index (worst 0-100 best)	0.15
	**RCA increase from Stage 3 to Stage 4 in 2022**			**RCA increase from Stage 1 to Stage 4 in 2022**		
**Rank**	**SDG indicators**	**Indicator name**	**RCA change**	**SDG indicators**	**Indicator name**	**RCA change**
**1**	6.D	Anthropogenic wastewater that receives treatment (%)	1.73	6.D	Anthropogenic wastewater that receives treatment (%)	2.12
**2**	9.E	Expenditure on research and development (% of GDP)	1.16	9.E	Expenditure on research and development (% of GDP)	1.83
**3**	16.E	Expropriations are lawful and adequately compensated (worst 0 - 1 best)	0.96	9.A	Articles published in academic journals (per 1,000 population)	1.75
**4**	9.A	Articles published in academic journals (per 1,000 population)	0.93	9.G	The Times Higher Education Universities Ranking: Average score of top 3 universities (worst 0-100 best)	1.33
**5**	16.H	Press Freedom Index (worst 0-100 best)	0.78	16.E	Expropriations are lawful and adequately compensated (worst 0 - 1 best)	1.30
**6**	8.E	Fundamental labor rights are effectively guaranteed (worst 0–1 best)	0.54	1.A	Poverty headcount ratio at $3.65/day (2017 PPP, %)	1.05
**7**	14.C	Mean area that is protected in marine sites important to biodiversity (%)	0.53	3.M	Universal health coverage (UHC) index of service coverage (worst 0-100 best)	0.97
**8**	9.G	The Times Higher Education Universities Ranking: Average score of top 3 universities (worst 0-100 best)	0.50	7.A	Population with access to clean fuels and technology for cooking (%)	0.86
**9**	16.C	Corruption Perceptions Index (worst 0-100 best)	0.49	4.A	Participation rate in pre-primary organized learning (% of children aged 4 to 6)	0.83
**10**	15.C	Mean area that is protected in terrestrial sites important to biodiversity (%)	0.47	3.K	Traffic deaths (per 100,000 population)	0.80

Supplementary Table 22 | The top 10 SDG indicators with the most decrease in revealed comparative advantage (RCA) for each sustainability stage in 2022.

	RCA decrease from Stage 1 to Stage 2 in 2022			RCA decrease from Stage 2 to Stage 3 in 2022		
**Rank**	**SDG indicators**	**Indicator name**	**RCA change**	**SDG indicators**	**Indicator name**	**RCA change**
**1**	2.B	Prevalence of obesity, BMI ≥ 30 (% of adult population)	-0.93	2.F	Human Trophic Level (best 2-3 worst)	-0.50
**2**	12.A	Electronic waste (kg/capita)	-0.84	14.D	Fish caught that are then discarded (%)	-0.37
**3**	14.E	Fish caught from overexploited or collapsed stocks (% of total catch)	-0.80	14.E	Fish caught from overexploited or collapsed stocks (% of total catch)	-0.37
**4**	2.F	Human Trophic Level (best 2-3 worst)	-0.79	14.F	Fish caught by trawling or dredging (%)	-0.35
**5**	12.E	Production-based nitrogen emissions (kg/capita)	-0.73	2.B	Prevalence of obesity, BMI ≥ 30 (% of adult population)	-0.27
**6**	13.B	CO2 emissions from fossil fuel combustion and cement production (tCO2/capita)	-0.73	2.C	Exports of hazardous pesticides (tonnes per million population)	-0.26
**7**	13.C	CO2 emissions embodied in imports (tCO2/capita)	-0.70	15.C	Mean area that is protected in terrestrial sites important to biodiversity (%)	-0.25
**8**	12.D	Nitrogen emissions embodied in imports (kg/capita)	-0.70	11.D	Satisfaction with public transport (%)	-0.23
**9**	6.C	Scarce water consumption embodied in imports (m3 H2O eq/capita)	-0.64	8.E	Fundamental labor rights are effectively guaranteed (worst 0–1 best)	-0.21
**10**	14.D	Fish caught that are then discarded (%)	-0.54	12.B	Exports of plastic waste (kg/capita)	-0.21

	RCA decrease from Stage 3 to Stage 4 in 2022			RCA decrease from Stage 1 to Stage 4 in 2022		
**Rank**	**SDG indicators**	**Indicator name**	**RCA change**	**SDG indicators**	**Indicator name**	**RCA change**
**1**	2.F	Human Trophic Level (best 2-3 worst)	-0.60	2.F	Human Trophic Level (best 2-3 worst)	-1.89
**2**	12.A	Electronic waste (kg/capita)	-0.59	12.A	Electronic waste (kg/capita)	-1.63
**3**	13.C	CO2 emissions embodied in imports (tCO2/capita)	-0.58	14.E	Fish caught from overexploited or collapsed stocks (% of total catch)	-1.47
**4**	12.B	Exports of plastic waste (kg/capita)	-0.52	2.B	Prevalence of obesity, BMI ≥ 30 (% of adult population)	-1.39
**5**	12.D	Nitrogen emissions embodied in imports (kg/capita)	-0.42	13.C	CO2 emissions embodied in imports (tCO2/capita)	-1.37
**6**	12.E	Production-based nitrogen emissions (kg/capita)	-0.37	12.D	Nitrogen emissions embodied in imports (kg/capita)	-1.23
**7**	12.C	Municipal solid waste (kg/capita/day)	-0.30	12.E	Production-based nitrogen emissions (kg/capita)	-1.17
**8**	14.E	Fish caught from overexploited or collapsed stocks (% of total catch)	-0.29	12.B	Exports of plastic waste (kg/capita)	-1.07
**9**	17.A	Corporate Tax Haven Score (best 0-100 worst)	-0.29	14.D	Fish caught that are then discarded (%)	-1.01
**10**	6.C	Scarce water consumption embodied in imports (m3 H2O eq/capita)	-0.28	13.B	CO2 emissions from fossil fuel combustion and cement production (tCO2/capita)	-1.00

Supplementary Figures

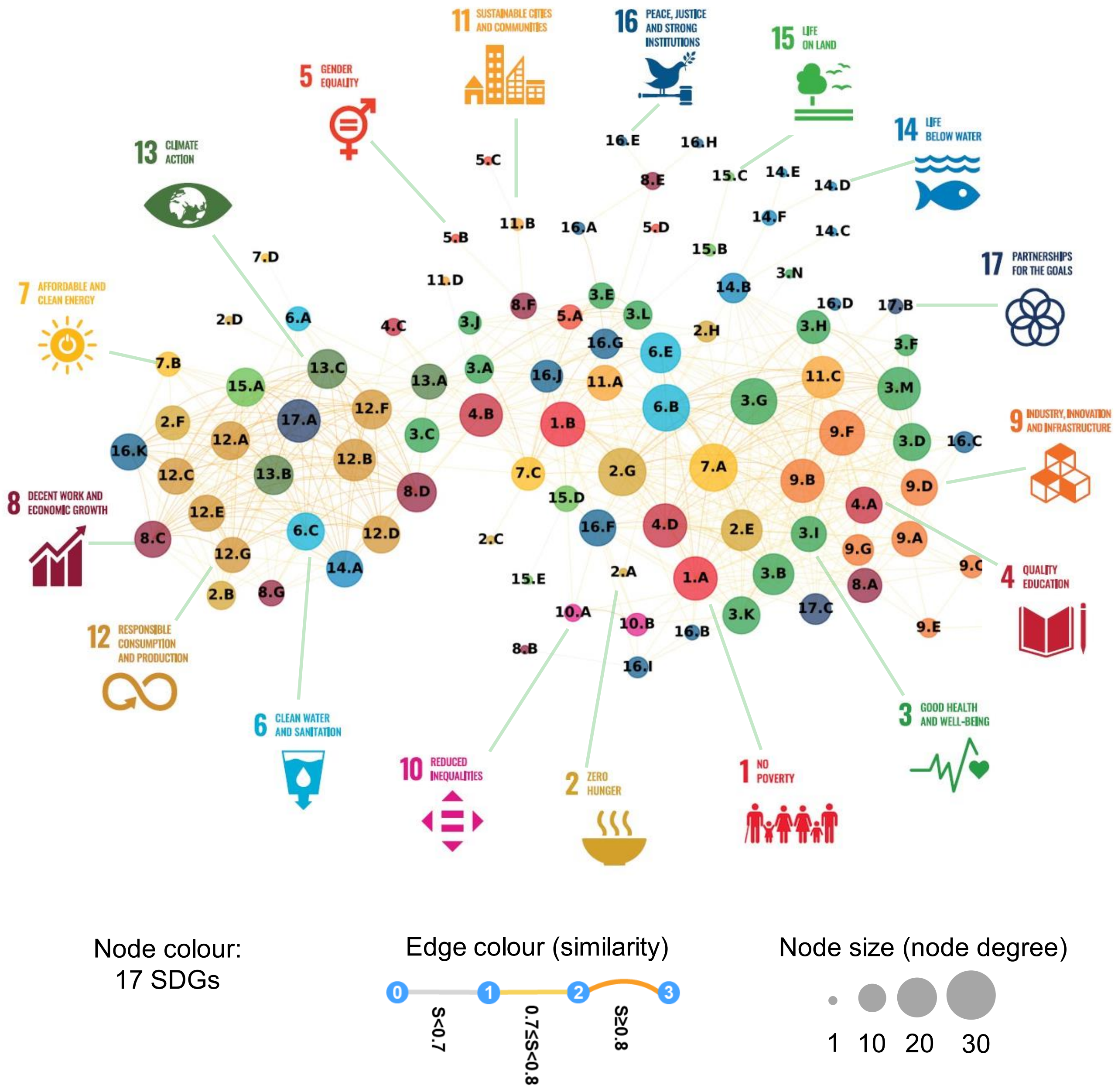

Supplementary Figure 1 | The SDG space with 17 SDG labels. The colour of the node represents the 17 SDGs it belongs to.

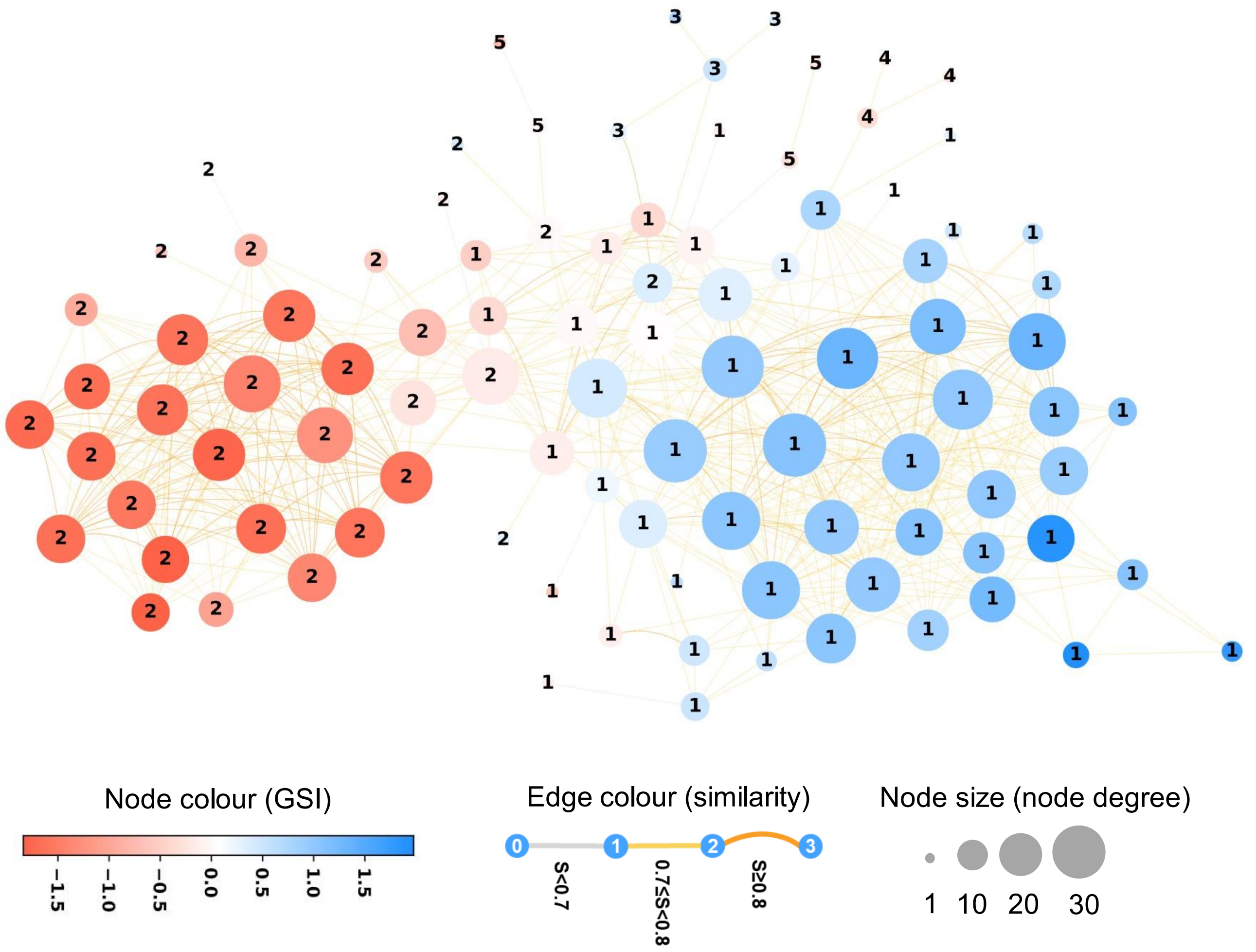

Supplementary Figure 2 | The SDG space by communities in 2022. The number in node represents the community it belongs to. The node colour represents goal sustainability index (GSI).

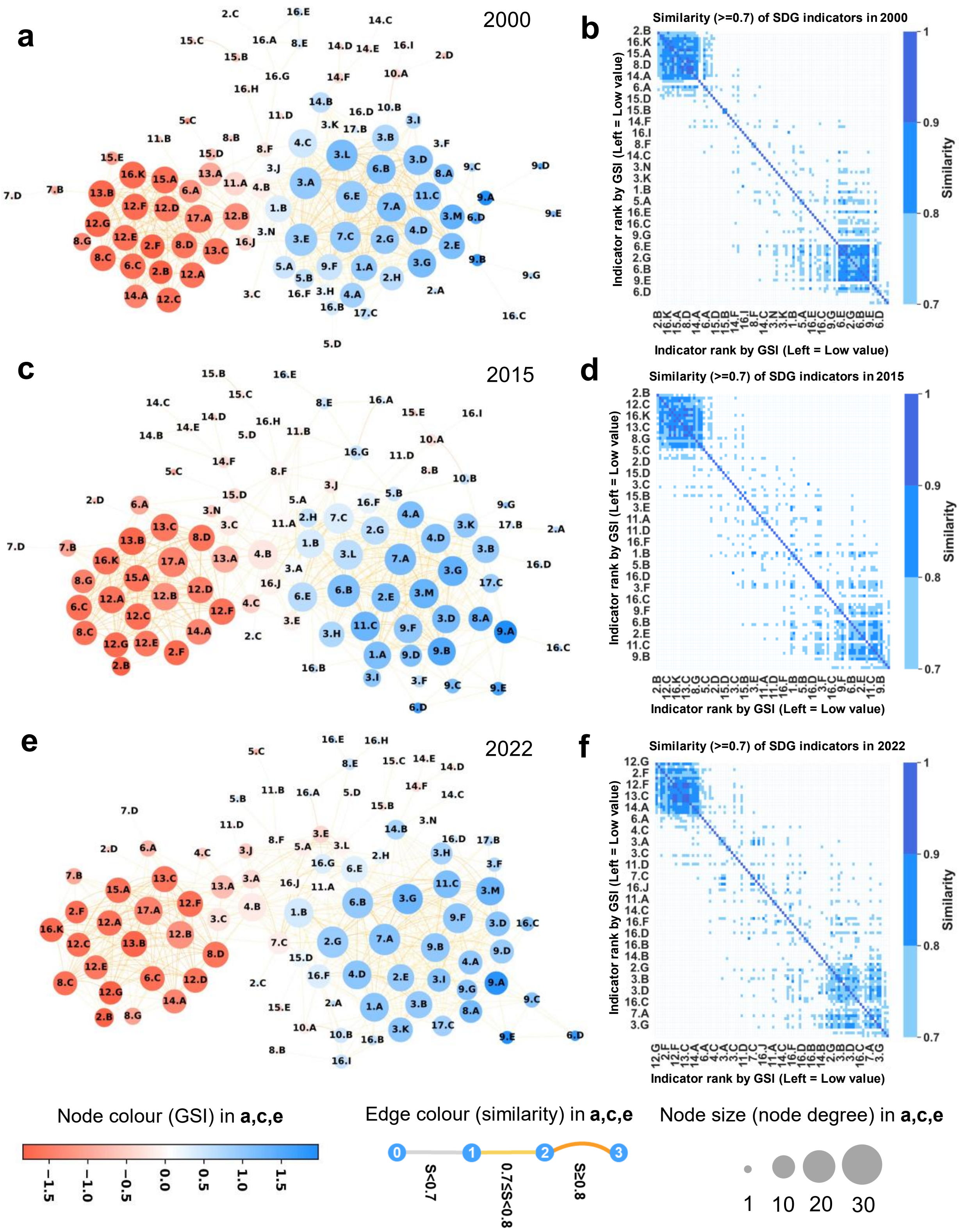

Supplementary Figure 3 | The evolution of SDG space. Panels **a, c,** and **e** show the global SDG space in 2000, 2015, and 2022, respectively. Panels **b, d,** and **f** display the similarity of SDG indicators in 2000, 2015, and 2022, respectively. GSI represents goal sustainability index.

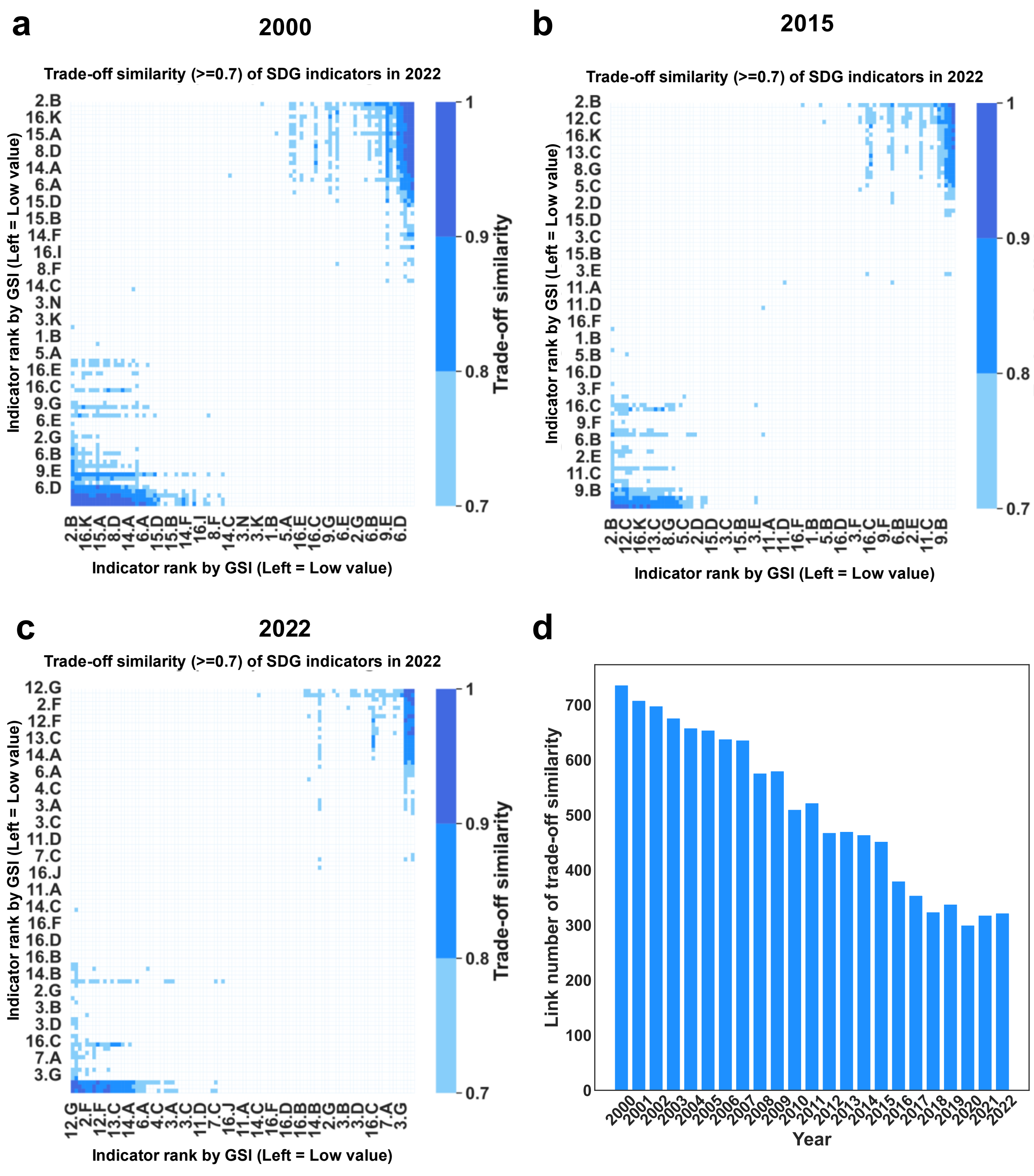

Supplementary Figure 4 | The trends of the trade-off similarity between SDG indicators. Panels **a, b, c,** The heatmap of the trade-off (negative) similarity (>=0.7) of SDG indicators in 2000, 2015, and 2022. **d** The link number with the trade-off similarity (>=0.7) between SDG indicators during 2000-2022. GSI represents goal sustainability index.

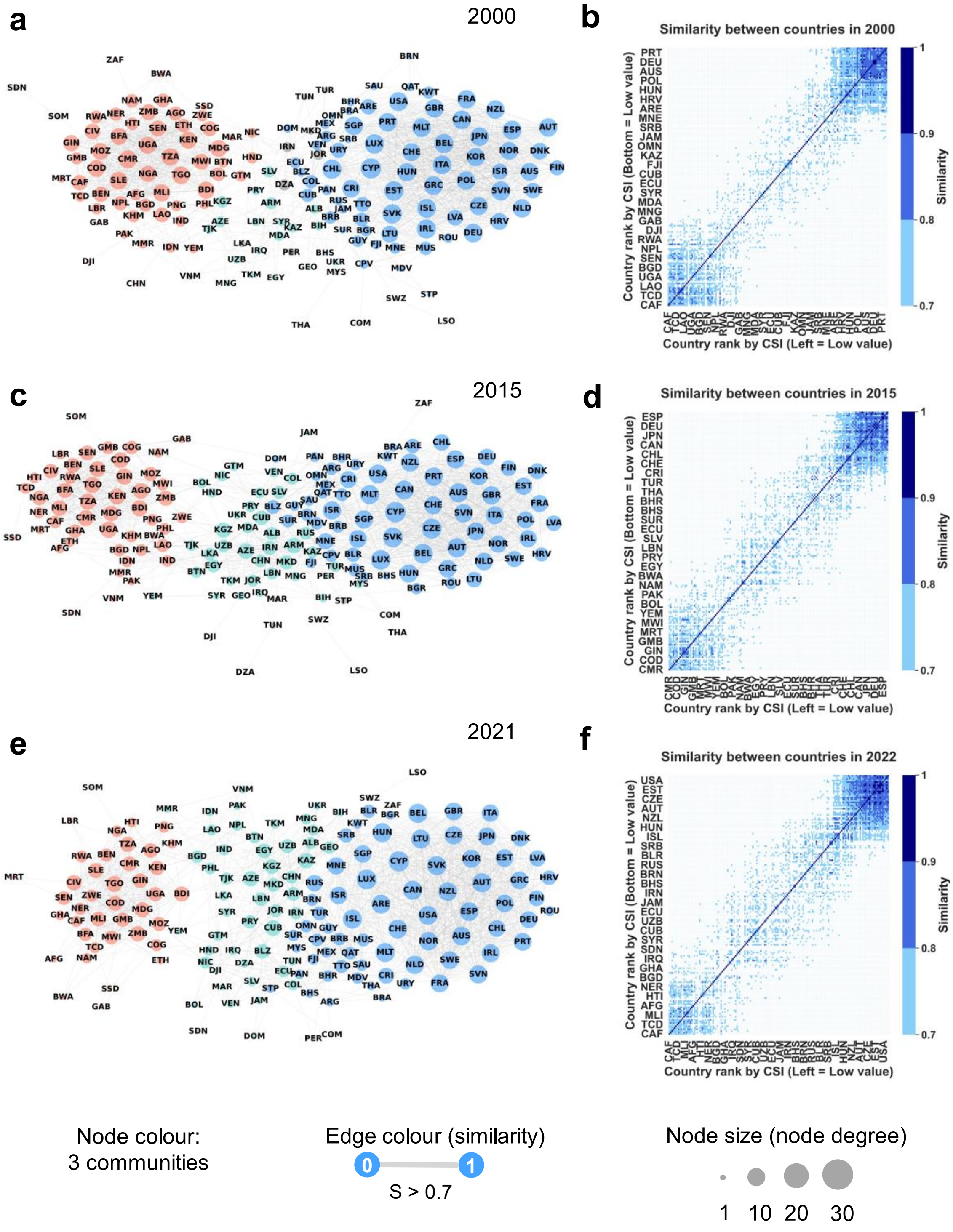

Supplementary Figure 5 | The evolution of country space and similarity of countries. a, **b** Year 2000. **c**, **d** Year 2015. **e, f** Year 2022. The node's colour represents three communities, and the node size is the node degree, representing the number of edges connected to the node (see Methods).

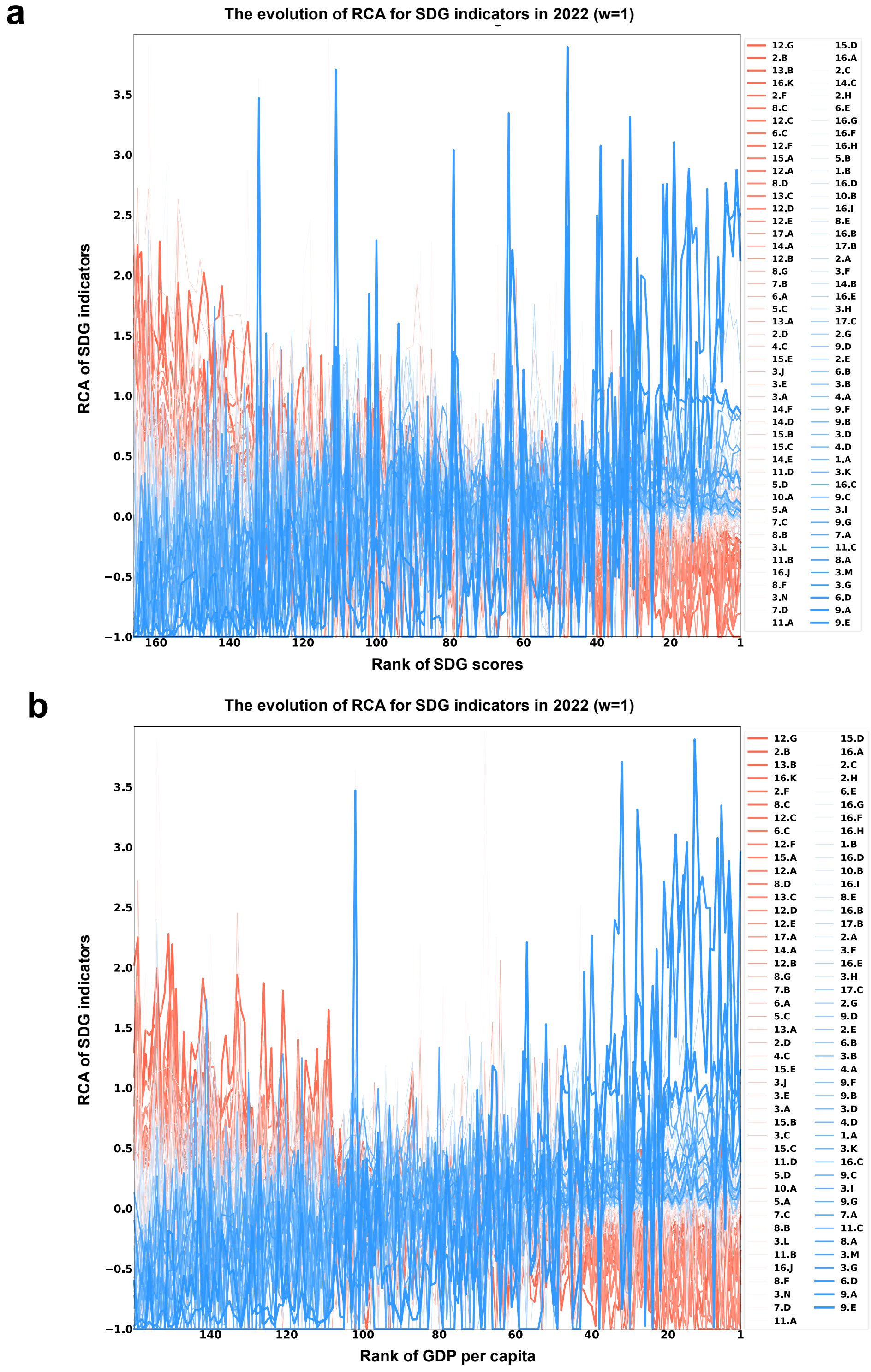

Supplementary Figure 6 | The evolution of SDG indicators along with the ranking of countries' SDG scores (a) and GDP per capita (b) in 2022 with moving-window size = 1. The GDP per capita of countries are measured by current US$.

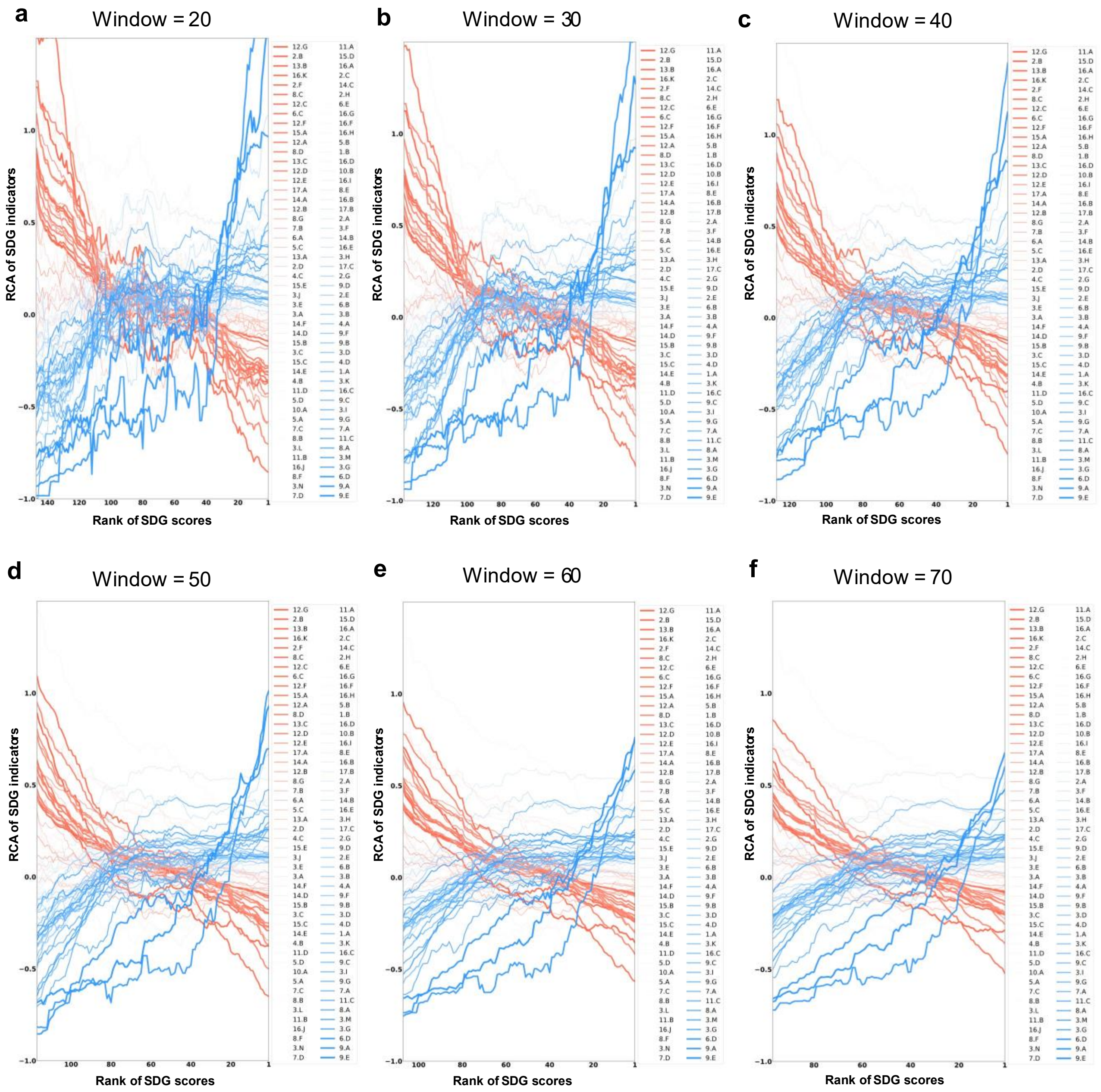

Supplementary Figure 7 | The evolution of SDG indicators alongside the ranking of countries' SDG scores in 2022 with varying moving-window sizes. Panels **a, b, c, d, e**, and **f** display the results using moving-window sizes of 20, 30, 40, 50, 60, and 70, respectively. RCA represents revealed comparative advantage.

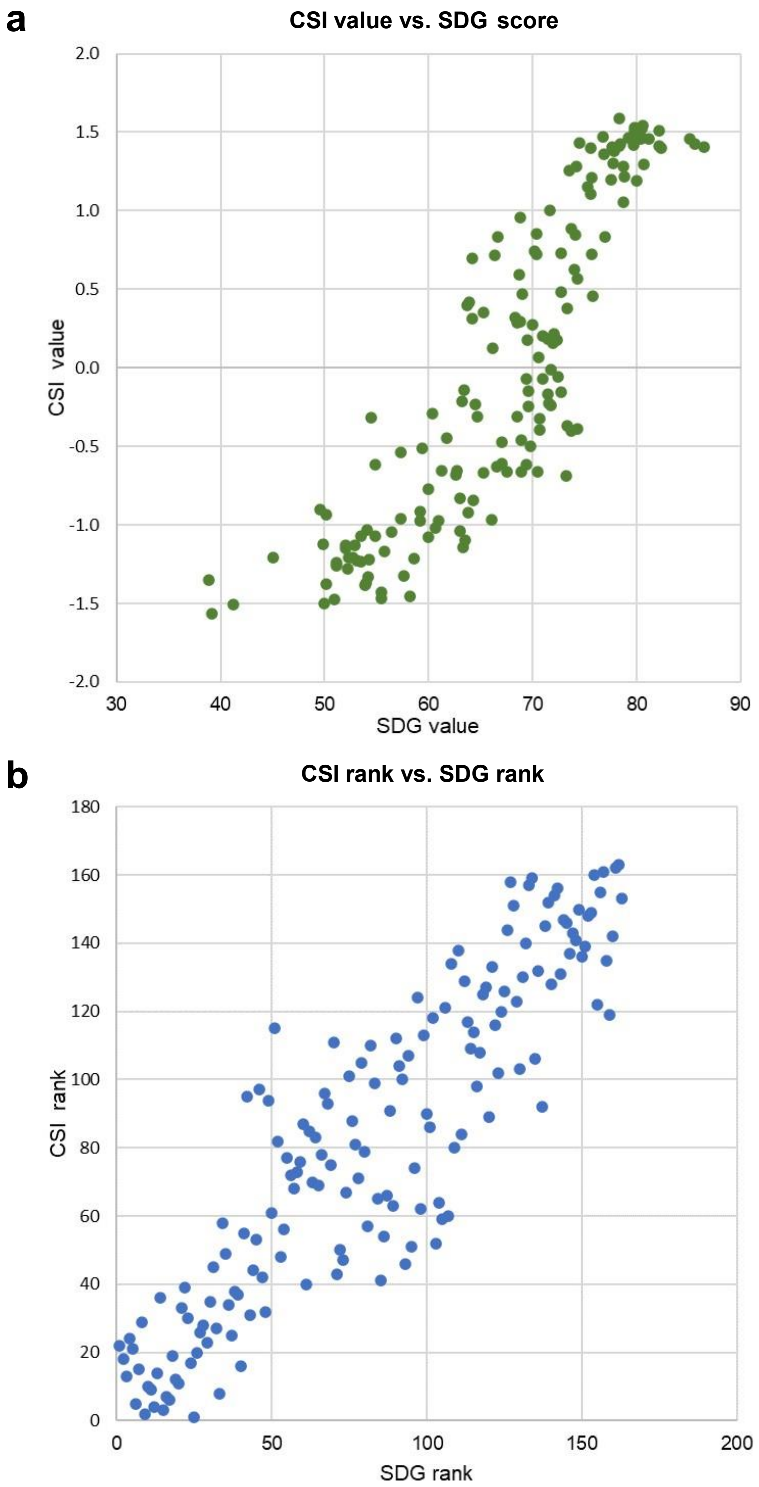

Supplementary Figure 8 | The relationship between the country sustainability index (CSI) and the SDG index of countries in 2022. Panel **a** shows the CSI value versus SDG index value. Panel **b** indicates the CSI rank versus the SDG index rank.

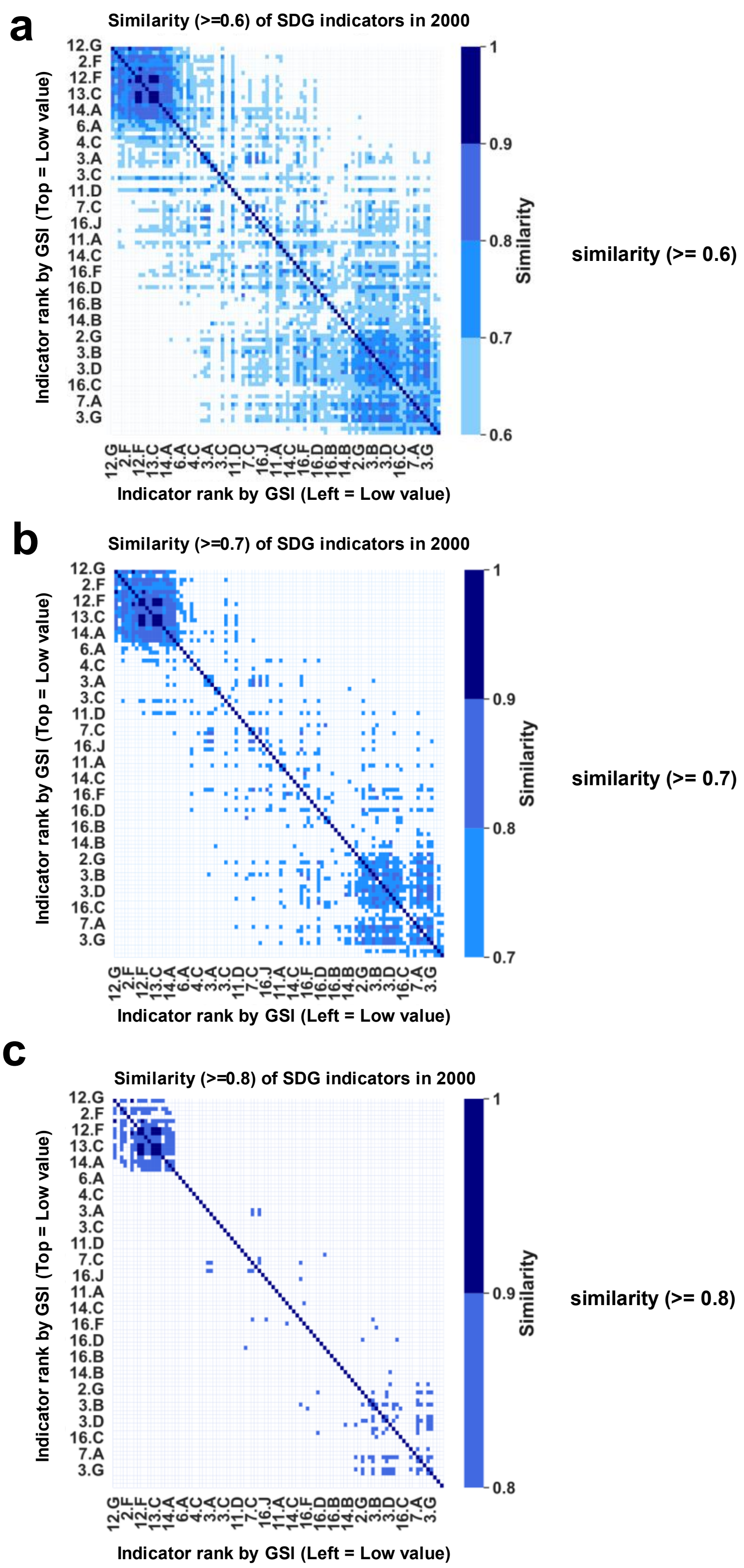

Supplementary Figure 9 | Sensitivity analysis of SDG indicators' similarity in 2022. Panel **a,** SDG indicators with similarity ⩾ 0.6. Panel **b,** SDG indicators with similarity ⩾ 0.7. Panel **c,** SDG indicators with similarity ⩾ 0.8.

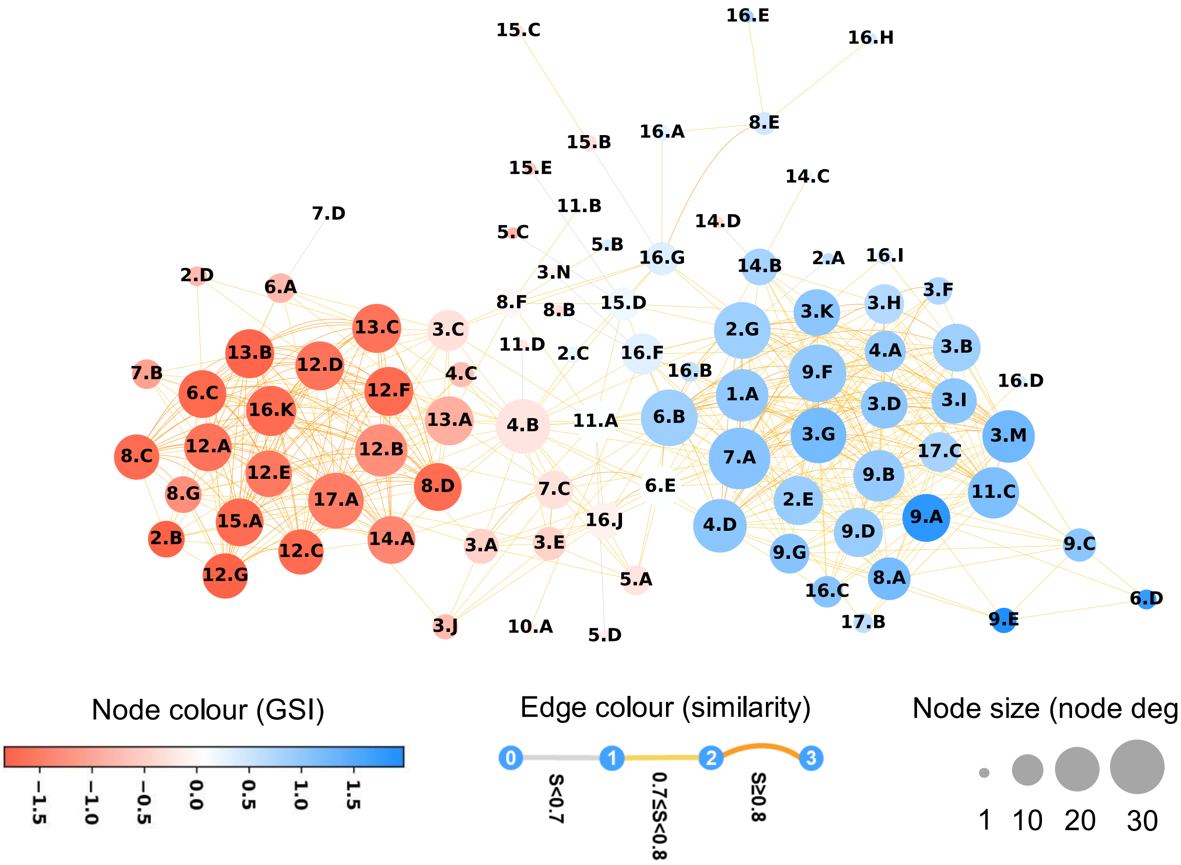

Supplementary Figure 10 | The SDG space in 2022 with non-overlapping indicators. We screened six groups of similar indicators: (1.A and 1.B), (2.B and 2.F), (2.E and 2.H), (3.G and 3.L), (10.A and 10.B), and (14.D, 14.E, and 14.F). For the robustness check, we retained only the non-overlapping indicators within these groups. The node colour represents Goal Sustainability Index (GSI), and the node size corresponds to the node degree, indicating the number of edges connected to the node.

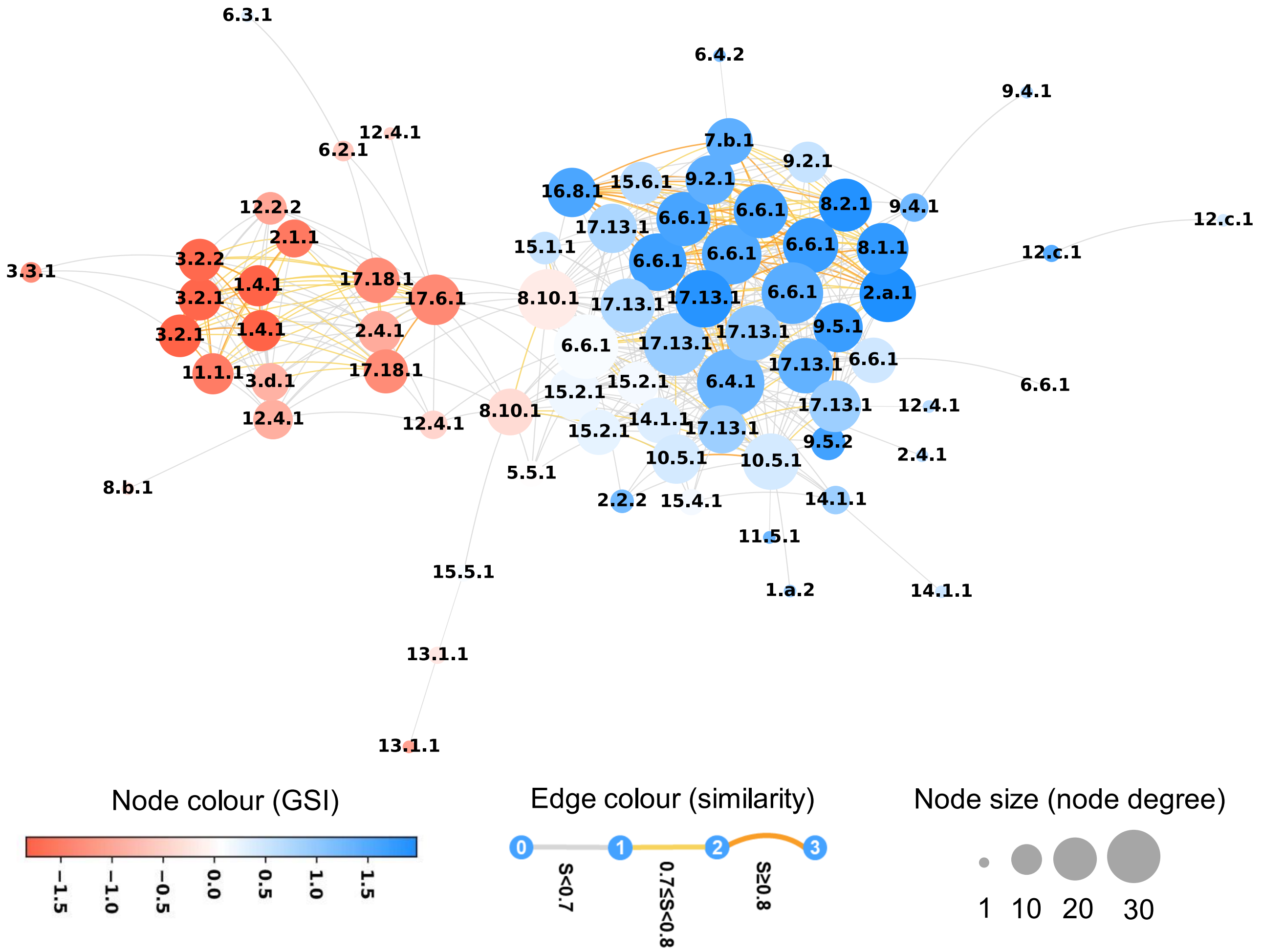

Supplementary Figure 11 | The SDG space in 2022 using UN SDG data. The node colour represents Goal Sustainability Index (GSI), and the node size indicates the node degree, representing the number of edges connected to the node. This SDG space was produced using data from the official United Nations Statistics Division (UNSD) SDG targets, which were matched with the 96 SDG indicators (see Tables 1-2 in Methods). Data source: https://unstats.un.org/sdgs/dataportal/database.

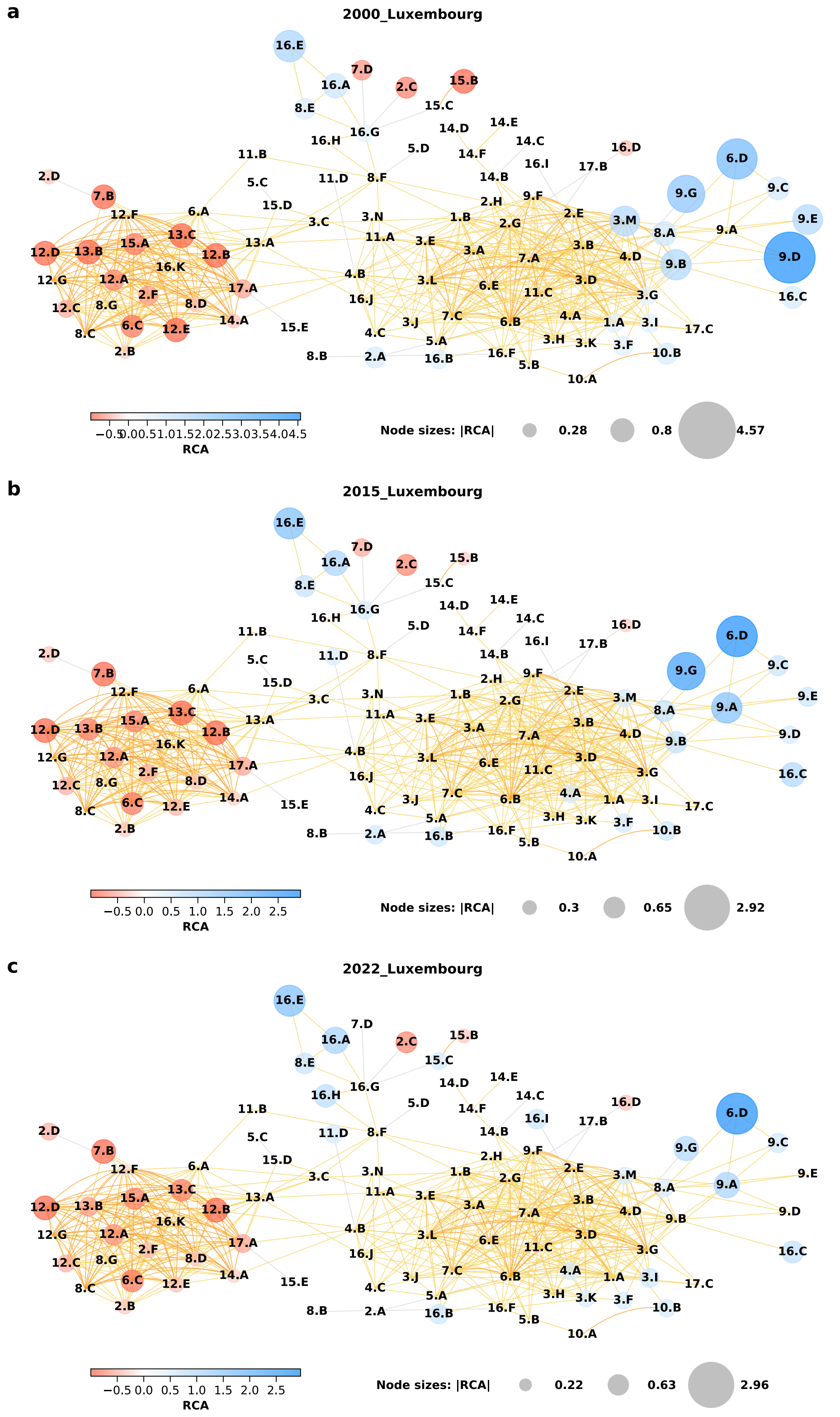

Supplementary Figure 12 | The SDG space of Luxembourg. Panels **a, b, c,** The SDG space in 2000, 2015, and 2022. The nodes in blue and orange represent the top 20 and bottom 20 SDG indicators in revealed comparative advantage (RCA) values, respectively. The node size represents the absolute value of RCA. From Supplementary Figure 12 to 177, countries are ranked by GDP/capita (current US$, 2022).

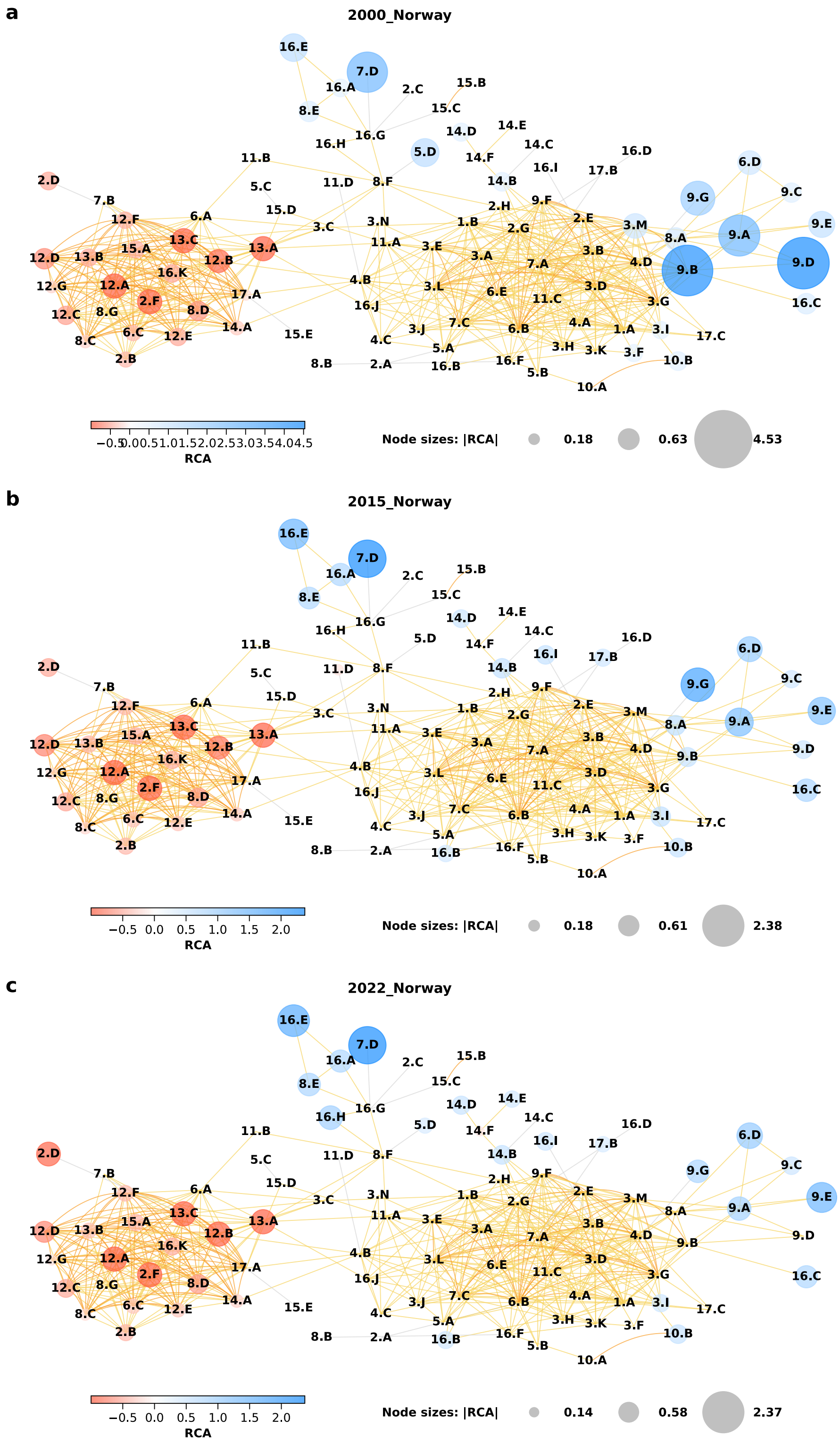

Supplementary Figure 13 | The SDG space of Norway. Panels **a, b, c,** The SDG space in 2000, 2015, and 2022. The nodes in blue and orange represent the top 20 and bottom 20 SDG indicators in revealed comparative advantage (RCA) values, respectively. The node size represents the absolute value of RCA. From Supplementary Figure 12 to 177, countries are ranked by GDP/capita (current US$, 2022).

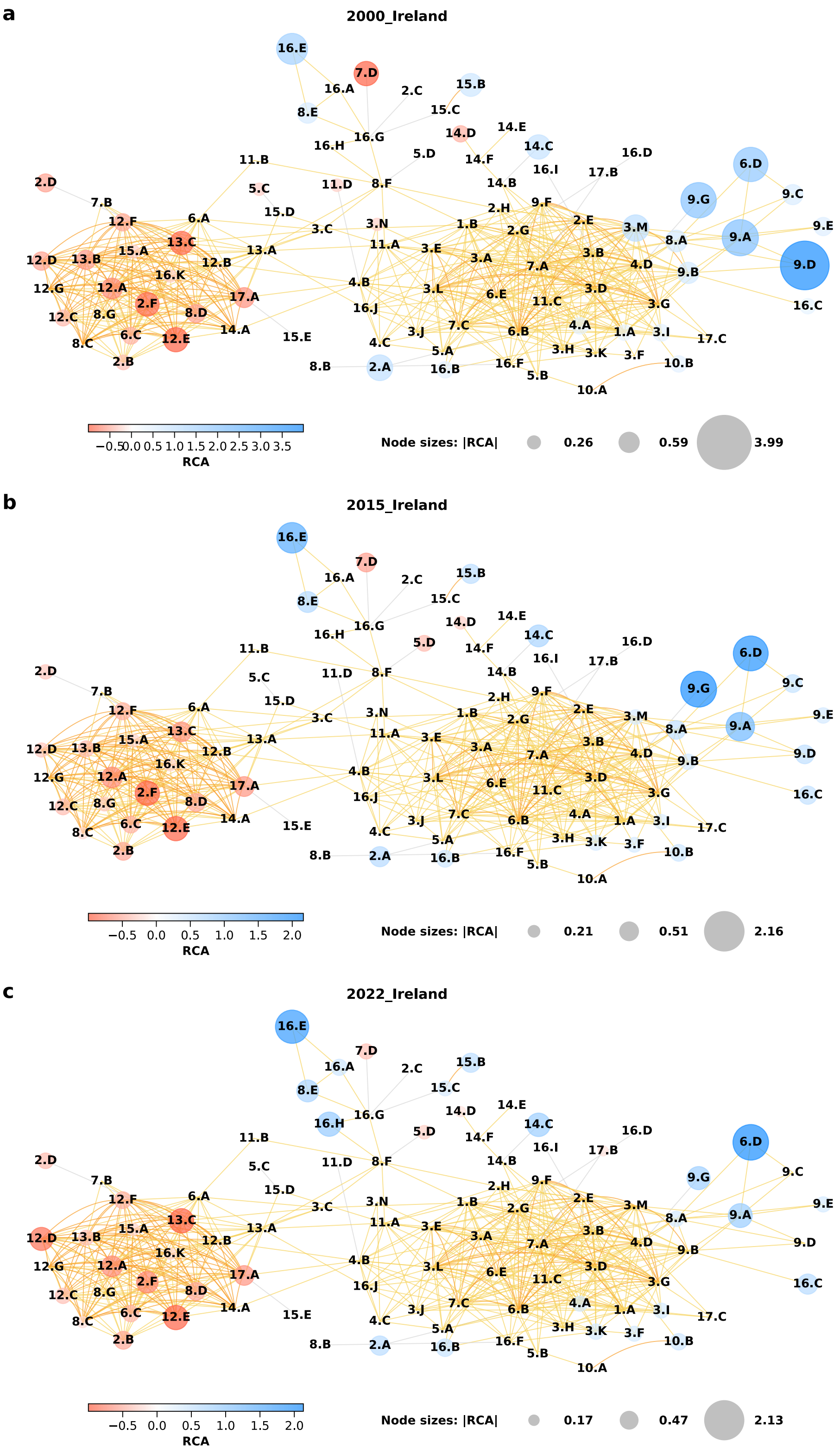

Supplementary Figure 14 | The SDG space of Ireland. Panels **a, b, c,** The SDG space in 2000, 2015, and 2022. The nodes in blue and orange represent the top 20 and bottom 20 SDG indicators in revealed comparative advantage (RCA) values, respectively. The node size represents the absolute value of RCA. From Supplementary Figure 12 to 177, countries are ranked by GDP/capita (current US$, 2022).

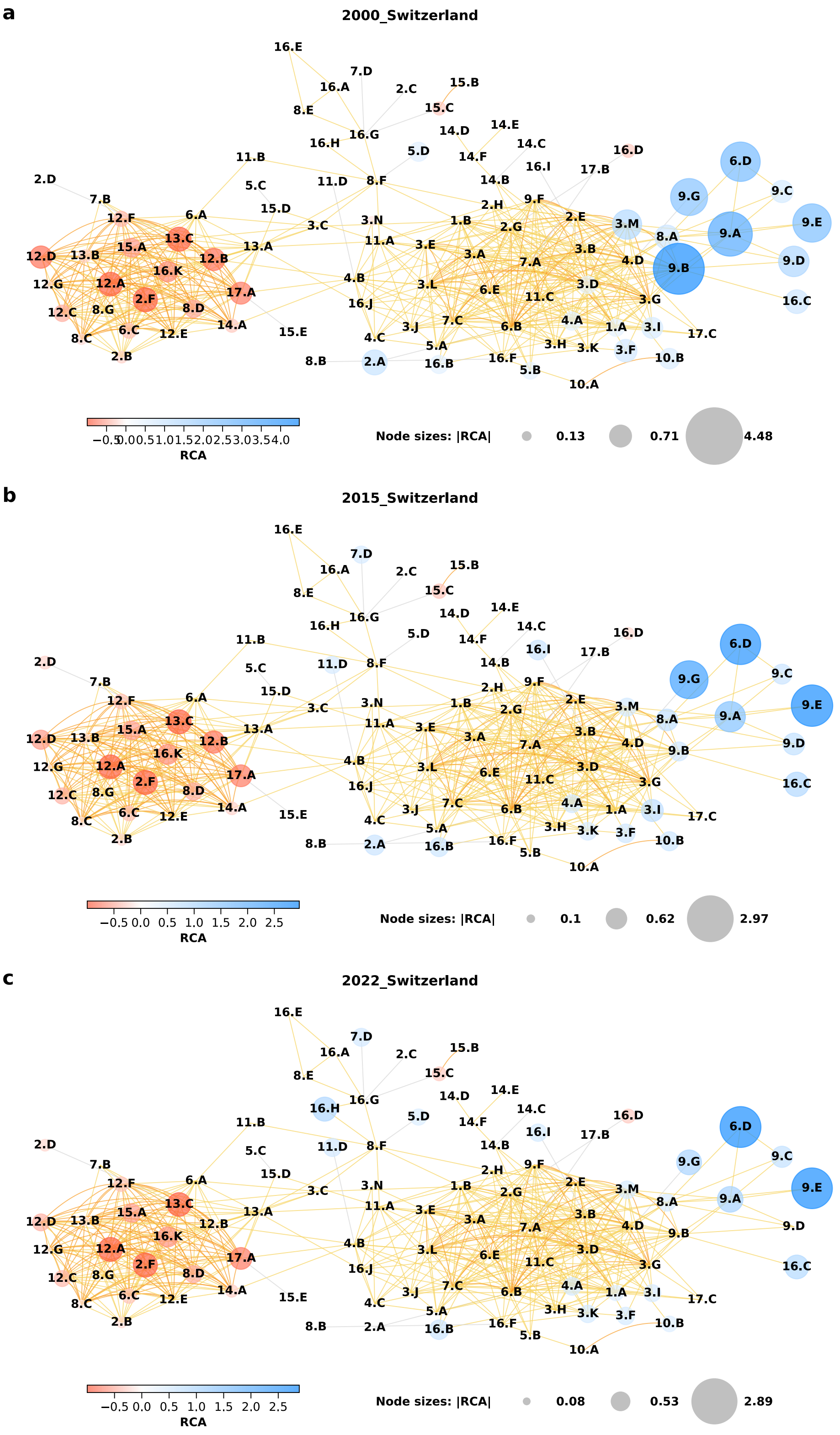

Supplementary Figure 15 | The SDG space of Switzerland. Panels **a, b, c,** The SDG space in 2000, 2015, and 2022. The nodes in blue and orange represent the top 20 and bottom 20 SDG indicators in revealed comparative advantage (RCA) values, respectively. The node size represents the absolute value of RCA. From Supplementary Figure 12 to 177, countries are ranked by GDP/capita (current US$, 2022).

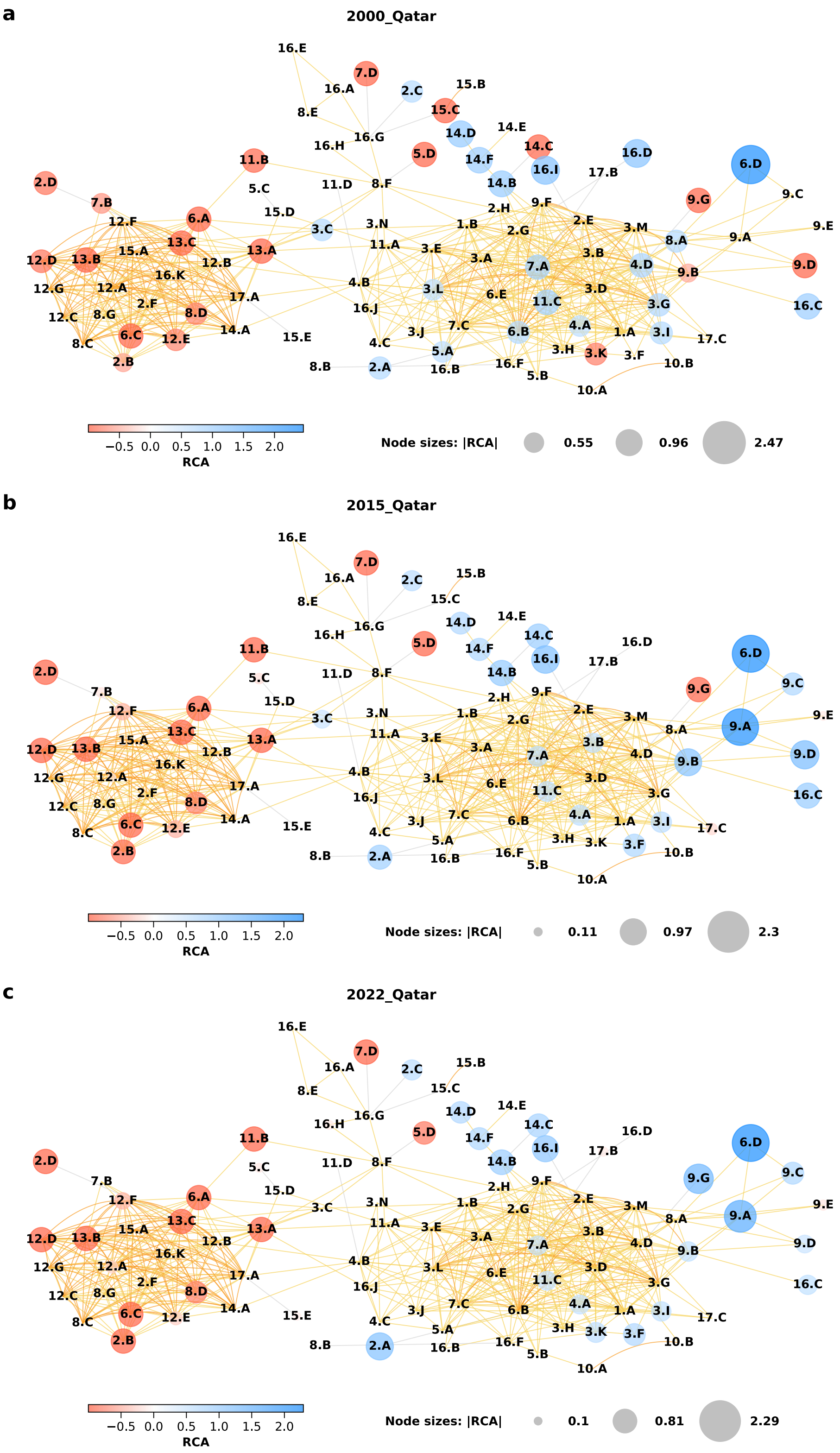

Supplementary Figure 16 | The SDG space of Qatar. Panels **a, b, c,** The SDG space in 2000, 2015, and 2022. The nodes in blue and orange represent the top 20 and bottom 20 SDG indicators in revealed comparative advantage (RCA) values, respectively. The node size represents the absolute value of RCA. From Supplementary Figure 12 to 177, countries are ranked by GDP/capita (current US$, 2022).

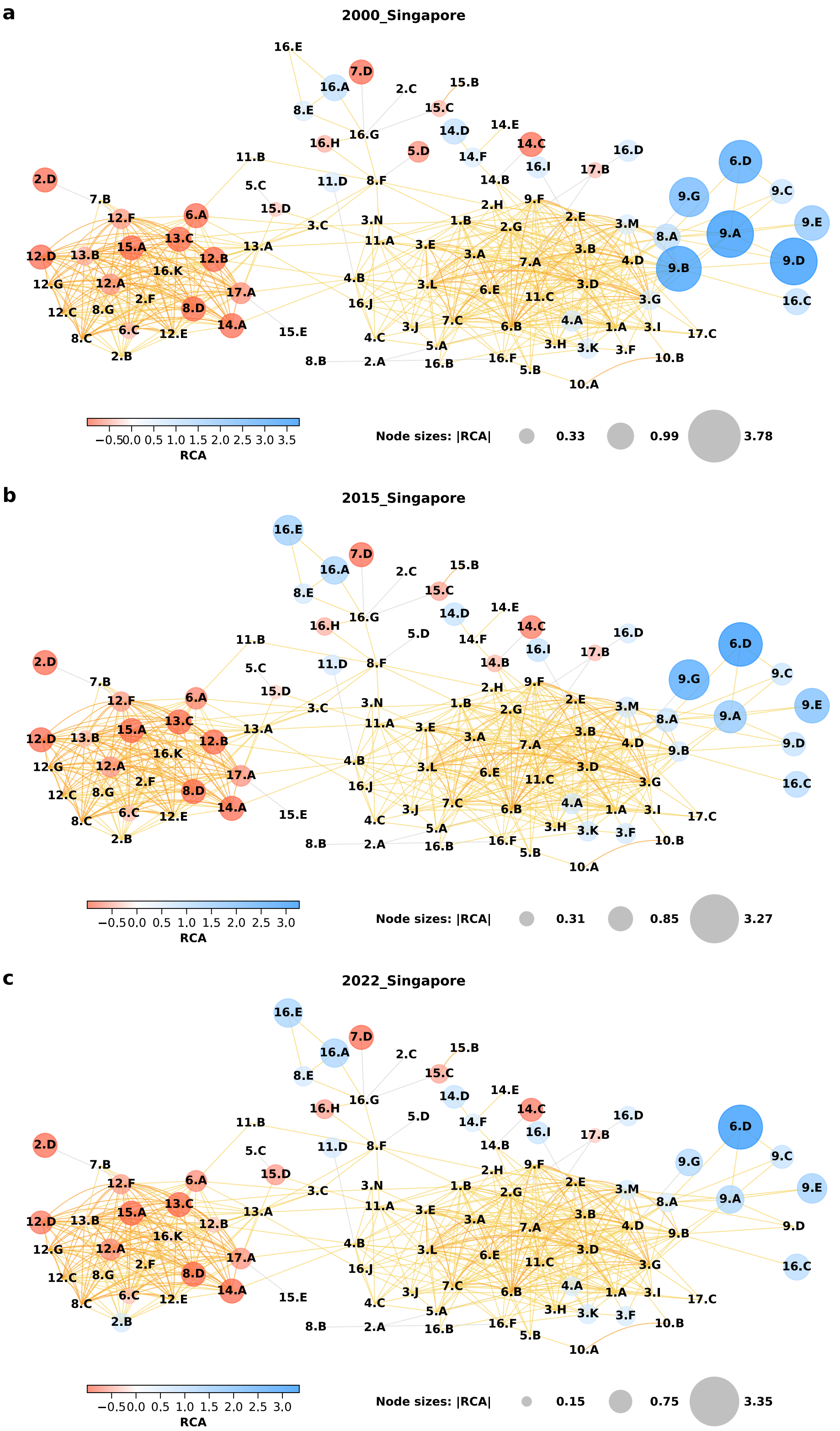

Supplementary Figure 17 | The SDG space of Singapore. Panels **a, b, c,**, The SDG space in 2000, 2015, and 2022. The nodes in blue and orange represent the top 20 and bottom 20 SDG indicators in revealed comparative advantage (RCA) values, respectively. The node size represents the absolute value of RCA. From Supplementary Figure 12 to 177, countries are ranked by GDP/capita (current US$, 2022).

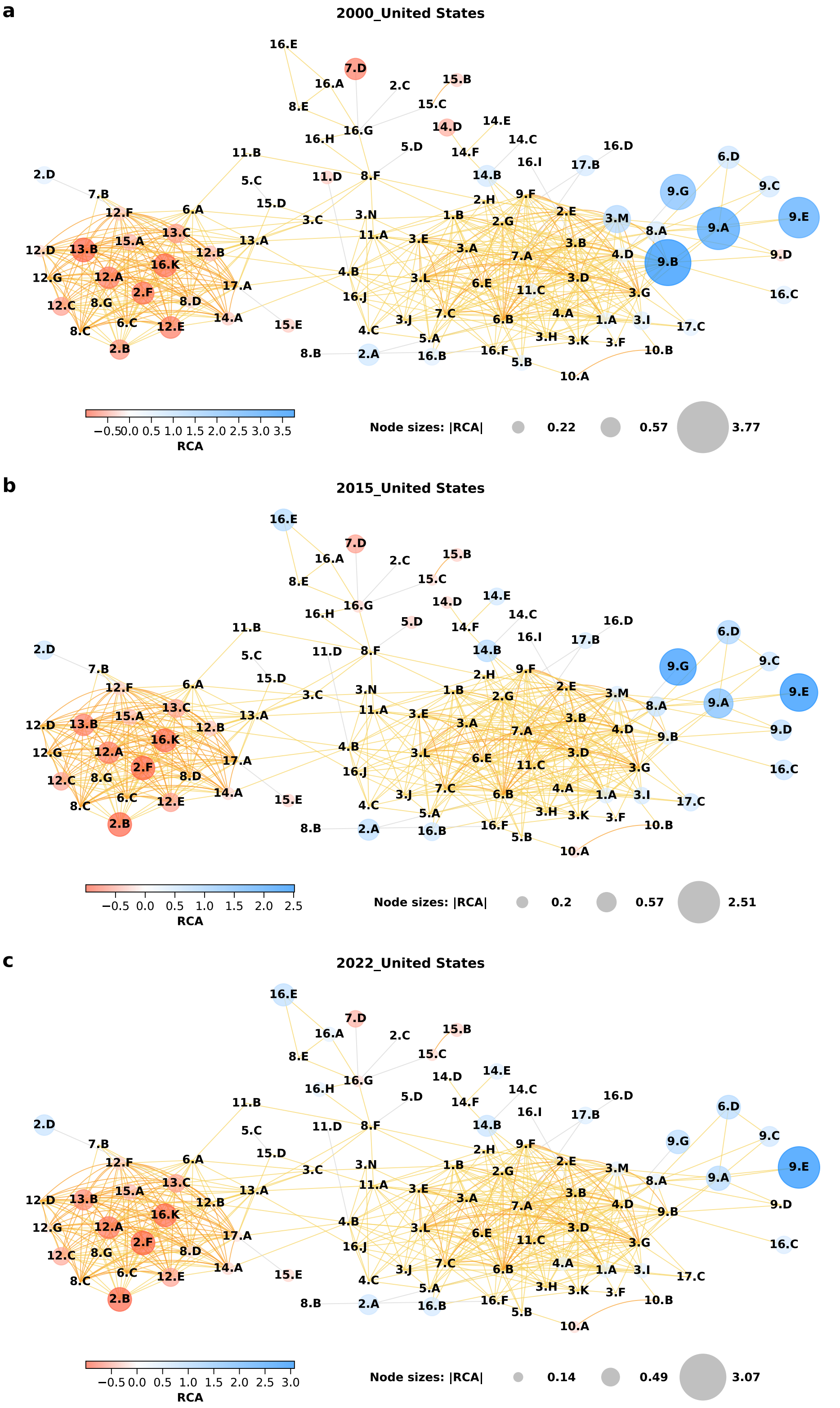

Supplementary Figure 18 | The SDG space of United States. Panels **a, b, c,** The SDG space in 2000, 2015, and 2022. The nodes in blue and orange represent the top 20 and bottom 20 SDG indicators in revealed comparative advantage (RCA) values, respectively. The node size represents the absolute value of RCA. From Supplementary Figure 12 to 177, countries are ranked by GDP/capita (current US$, 2022).

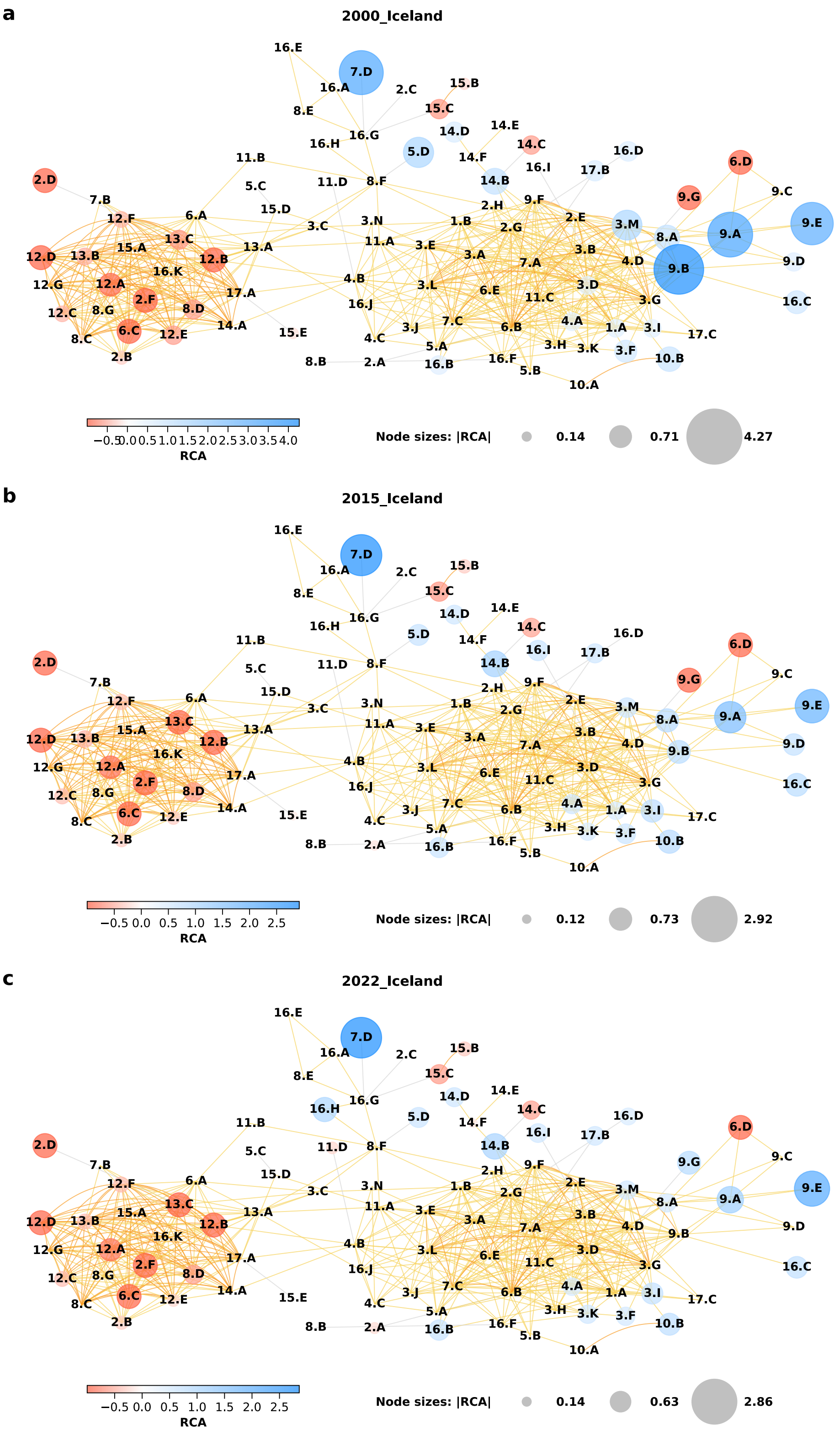

Supplementary Figure 19 | The SDG space of Iceland. Panels **a, b, c,** The SDG space in 2000, 2015, and 2022. The nodes in blue and orange represent the top 20 and bottom 20 SDG indicators in revealed comparative advantage (RCA) values, respectively. The node size represents the absolute value of RCA. From Supplementary Figure 12 to 177, countries are ranked by GDP/capita (current US$, 2022).

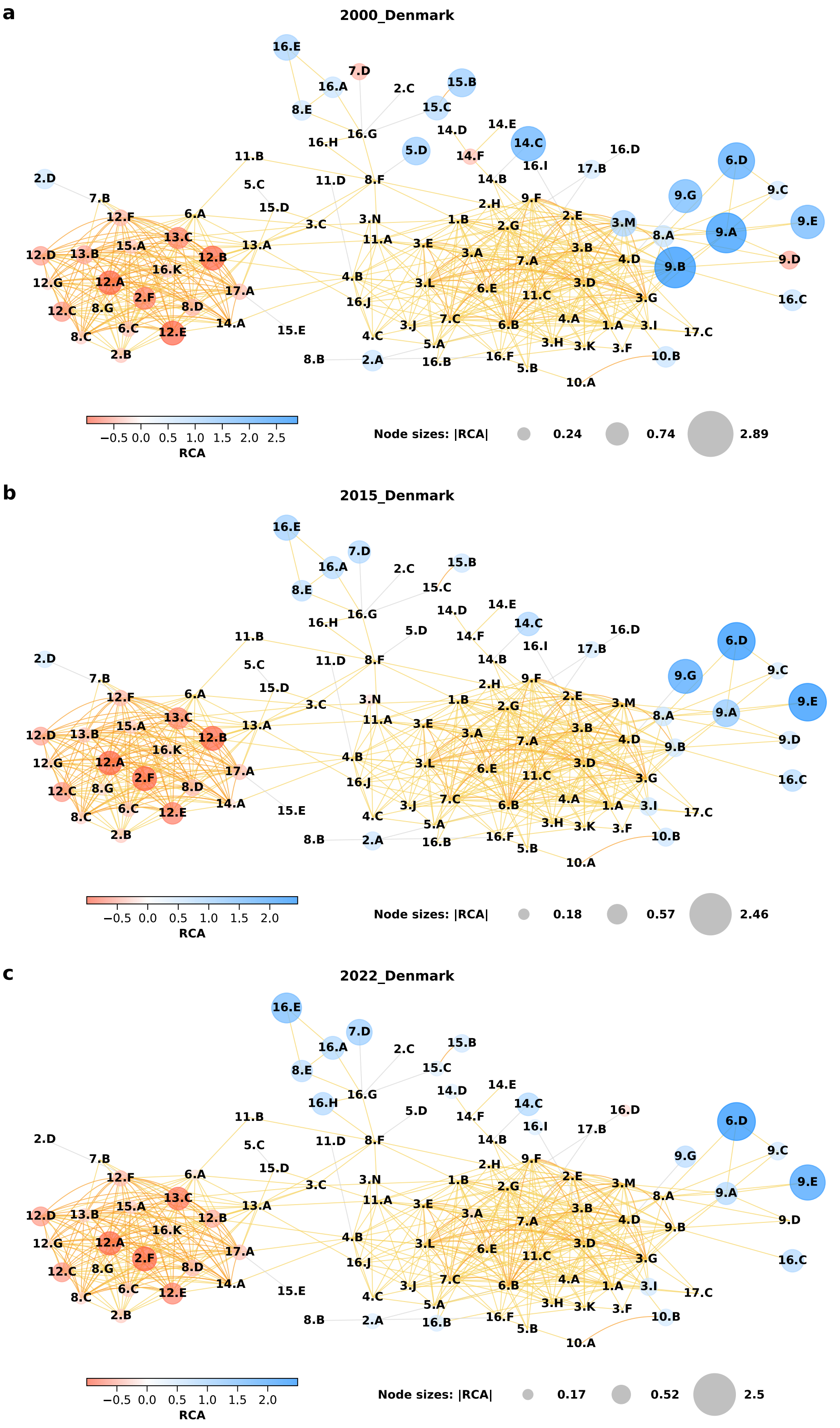

Supplementary Figure 20 | The SDG space of Denmark. Panels **a, b, c,** The SDG space in 2000, 2015, and 2022. The nodes in blue and orange represent the top 20 and bottom 20 SDG indicators in revealed comparative advantage (RCA) values, respectively. The node size represents the absolute value of RCA. From Supplementary Figure 12 to 177, countries are ranked by GDP/capita (current US$, 2022).

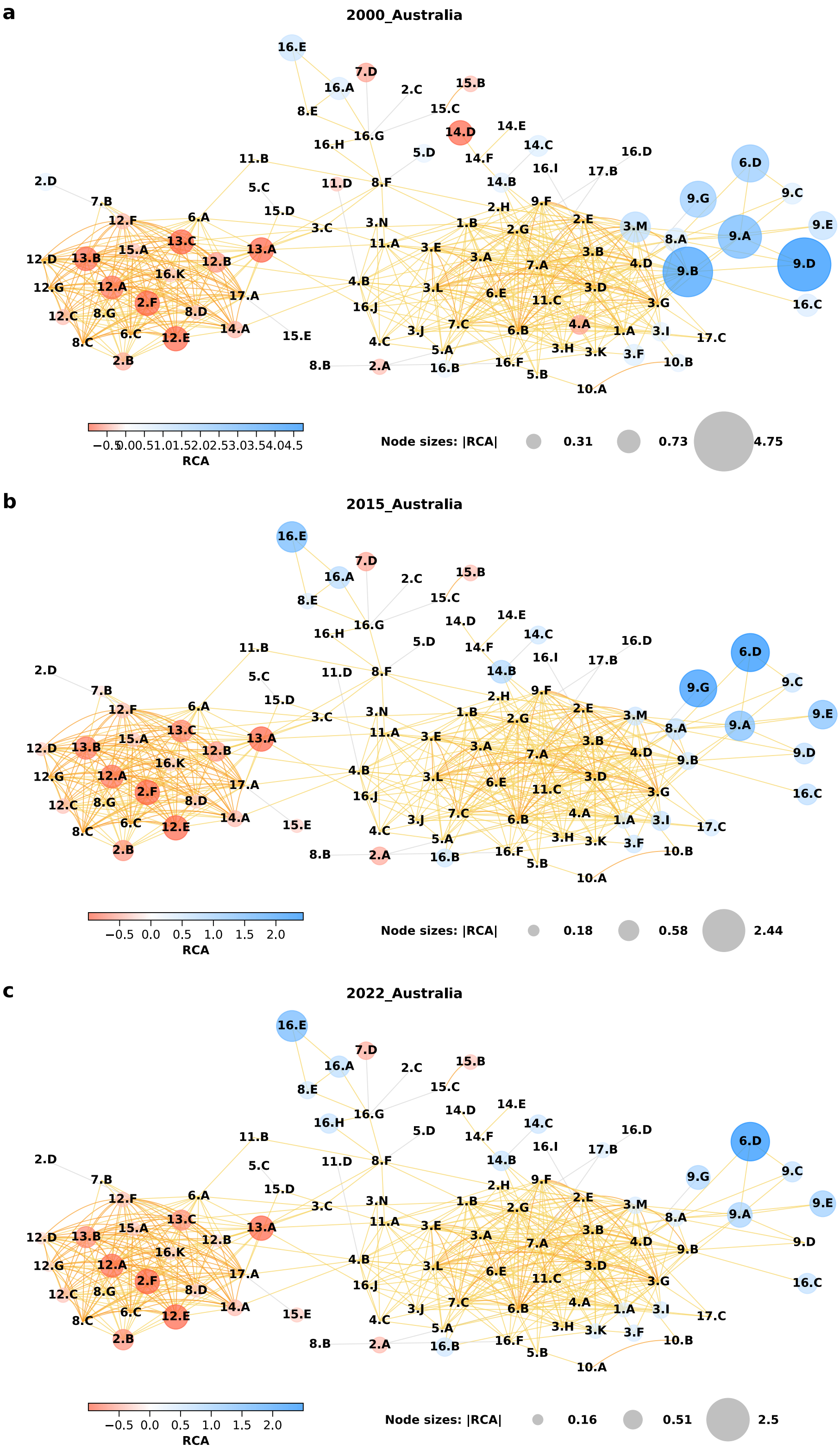

Supplementary Figure 21 | The SDG space of Australia. Panels **a, b, c,** The SDG space in 2000, 2015, and 2022. The nodes in blue and orange represent the top 20 and bottom 20 SDG indicators in revealed comparative advantage (RCA) values, respectively. The node size represents the absolute value of RCA. From Supplementary Figure 12 to 177, countries are ranked by GDP/capita (current US$, 2022).

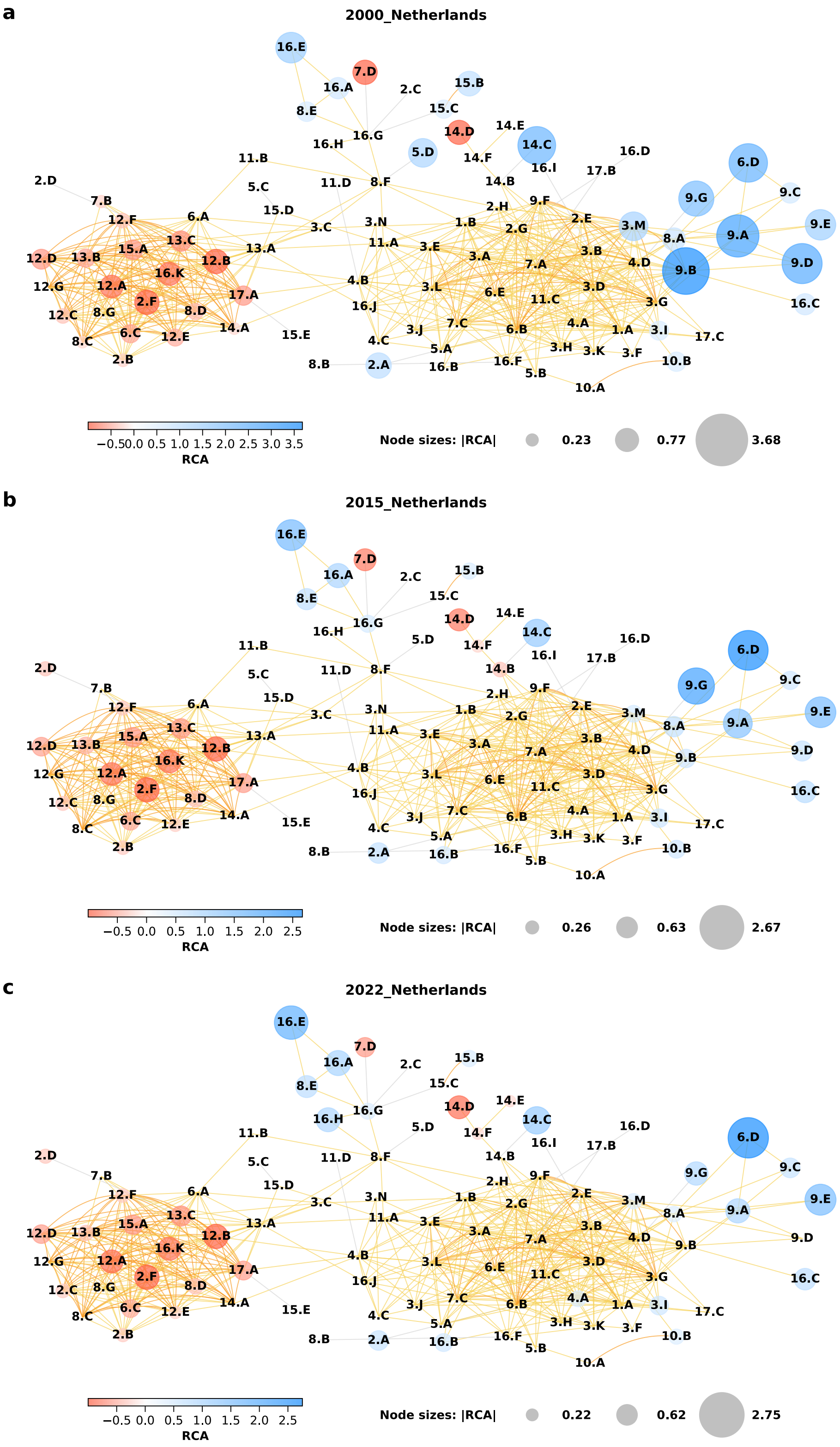

Supplementary Figure 22 | The SDG space of Netherlands. Panels **a, b, c,** The SDG space in 2000, 2015, and 2022. The nodes in blue and orange represent the top 20 and bottom 20 SDG indicators in revealed comparative advantage (RCA) values, respectively. The node size represents the absolute value of RCA. From Supplementary Figure 12 to 177, countries are ranked by GDP/capita (current US$, 2022).

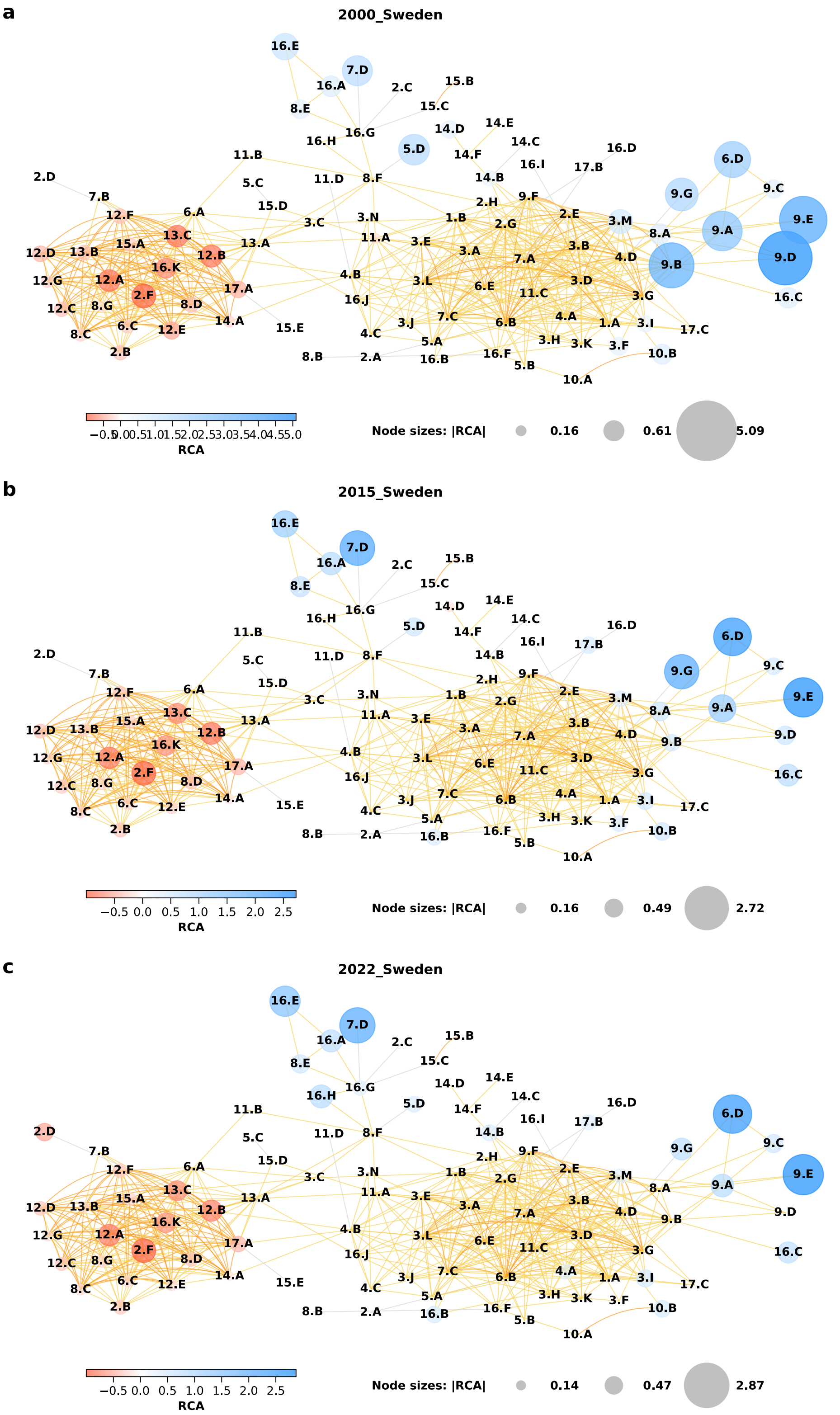

Supplementary Figure 23 | The SDG space of Sweden. Panels **a, b, c,** The SDG space in 2000, 2015, and 2022. The nodes in blue and orange represent the top 20 and bottom 20 SDG indicators in revealed comparative advantage (RCA) values, respectively. The node size represents the absolute value of RCA. From Supplementary Figure 12 to 177, countries are ranked by GDP/capita (current US$, 2022).

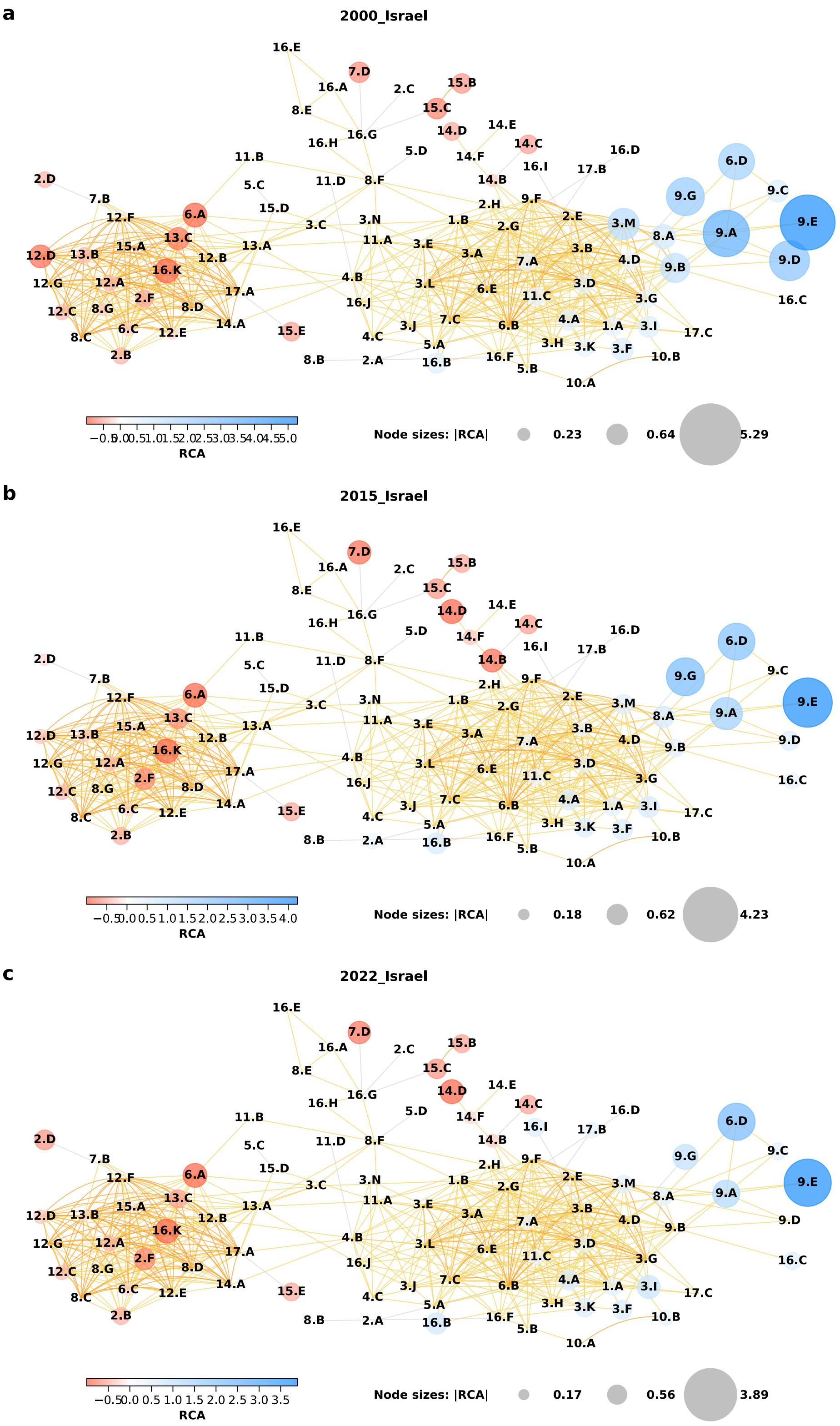

Supplementary Figure 24 | The SDG space of Israel. Panels **a, b, c,** The SDG space in 2000, 2015, and 2022. The nodes in blue and orange represent the top 20 and bottom 20 SDG indicators in revealed comparative advantage (RCA) values, respectively. The node size represents the absolute value of RCA. From Supplementary Figure 12 to 177, countries are ranked by GDP/capita (current US$, 2022).

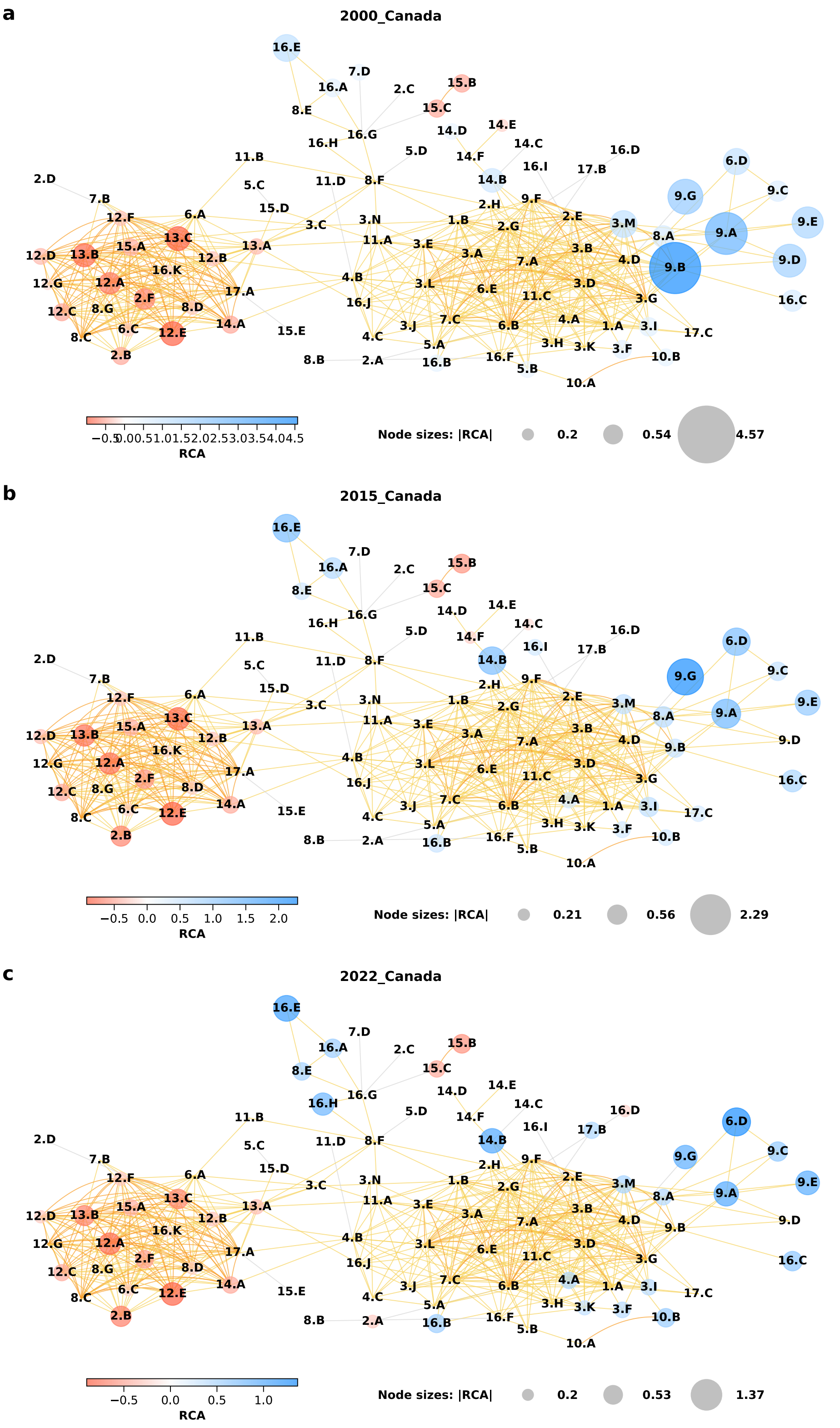

Supplementary Figure 25 | The SDG space of Canada. Panels **a, b, c,** The SDG space in 2000, 2015, and 2022. The nodes in blue and orange represent the top 20 and bottom 20 SDG indicators in revealed comparative advantage (RCA) values, respectively. The node size represents the absolute value of RCA. From Supplementary Figure 12 to 177, countries are ranked by GDP/capita (current US$, 2022).

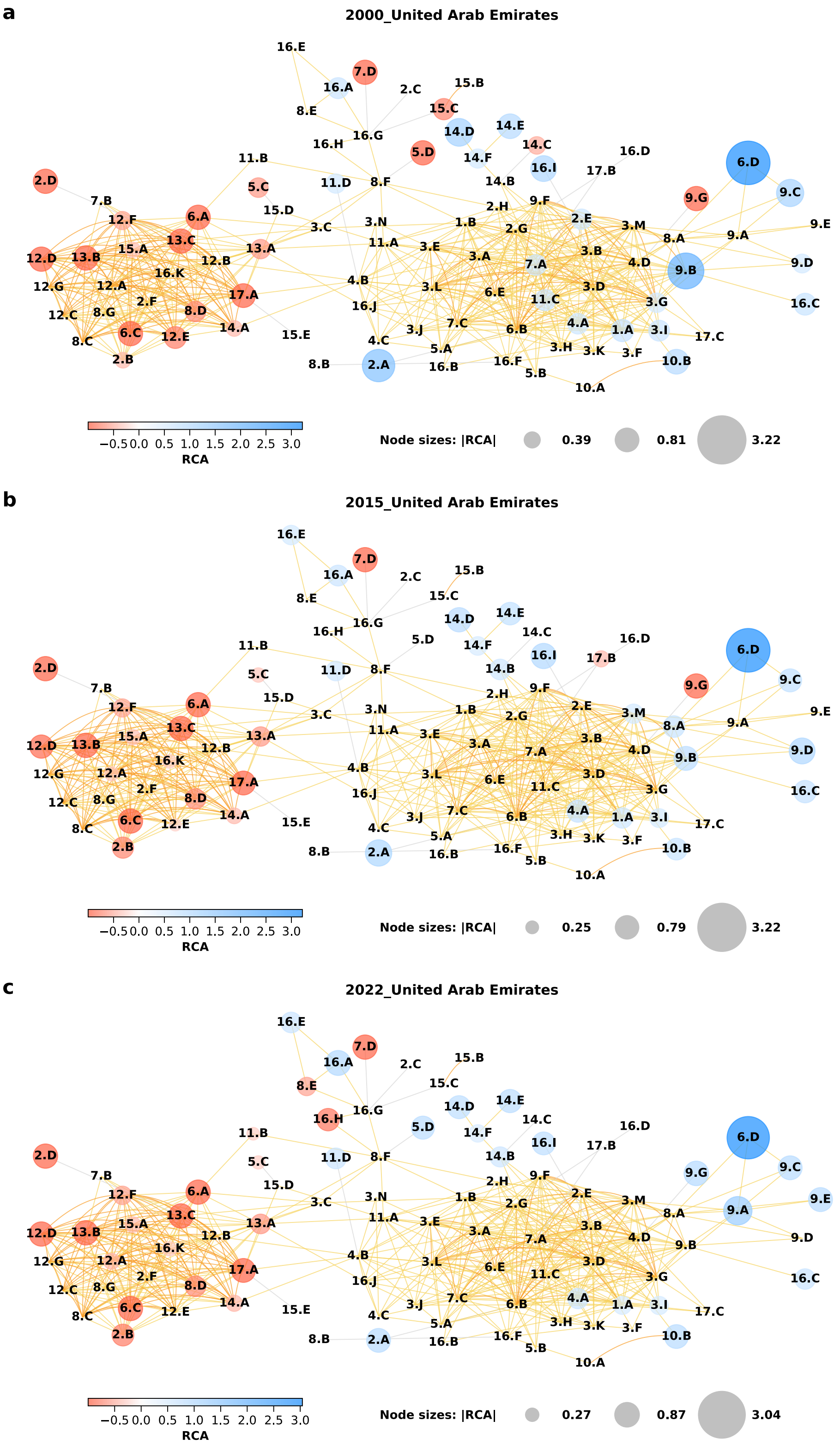

Supplementary Figure 26 | The SDG space of United Arab Emirates. Panels **a, b, c,** The SDG space in 2000, 2015, and 2022. The nodes in blue and orange represent the top 20 and bottom 20 SDG indicators in revealed comparative advantage (RCA) values, respectively. The node size represents the absolute value of RCA. From Supplementary Figure 12 to 177, countries are ranked by GDP/capita (current US$, 2022).

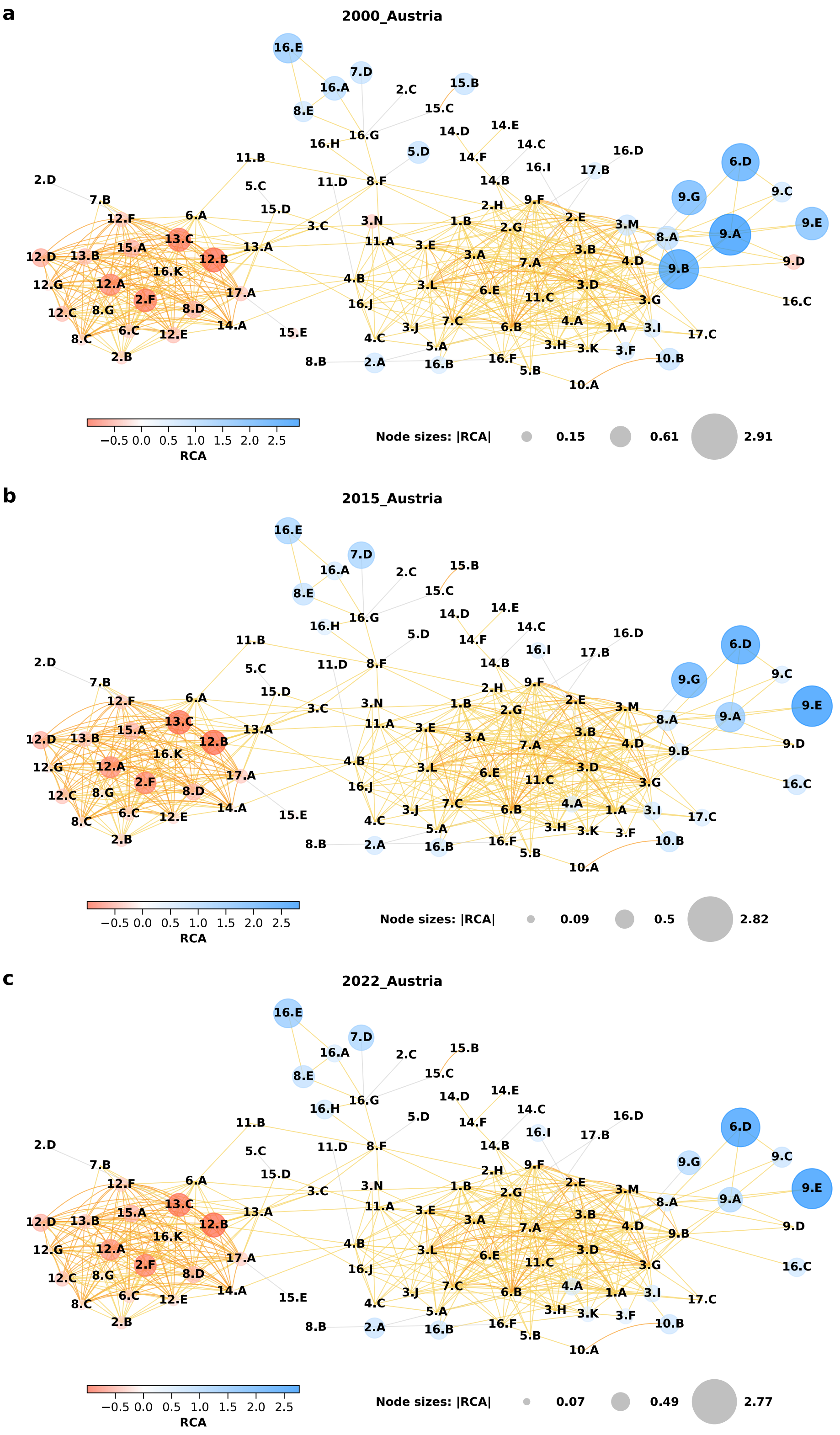

Supplementary Figure 27 | The SDG space of Austria. Panels **a, b, c,** The SDG space in 2000, 2015, and 2022. The nodes in blue and orange represent the top 20 and bottom 20 SDG indicators in revealed comparative advantage (RCA) values, respectively. The node size represents the absolute value of RCA. From Supplementary Figure 12 to 177, countries are ranked by GDP/capita (current US$, 2022).

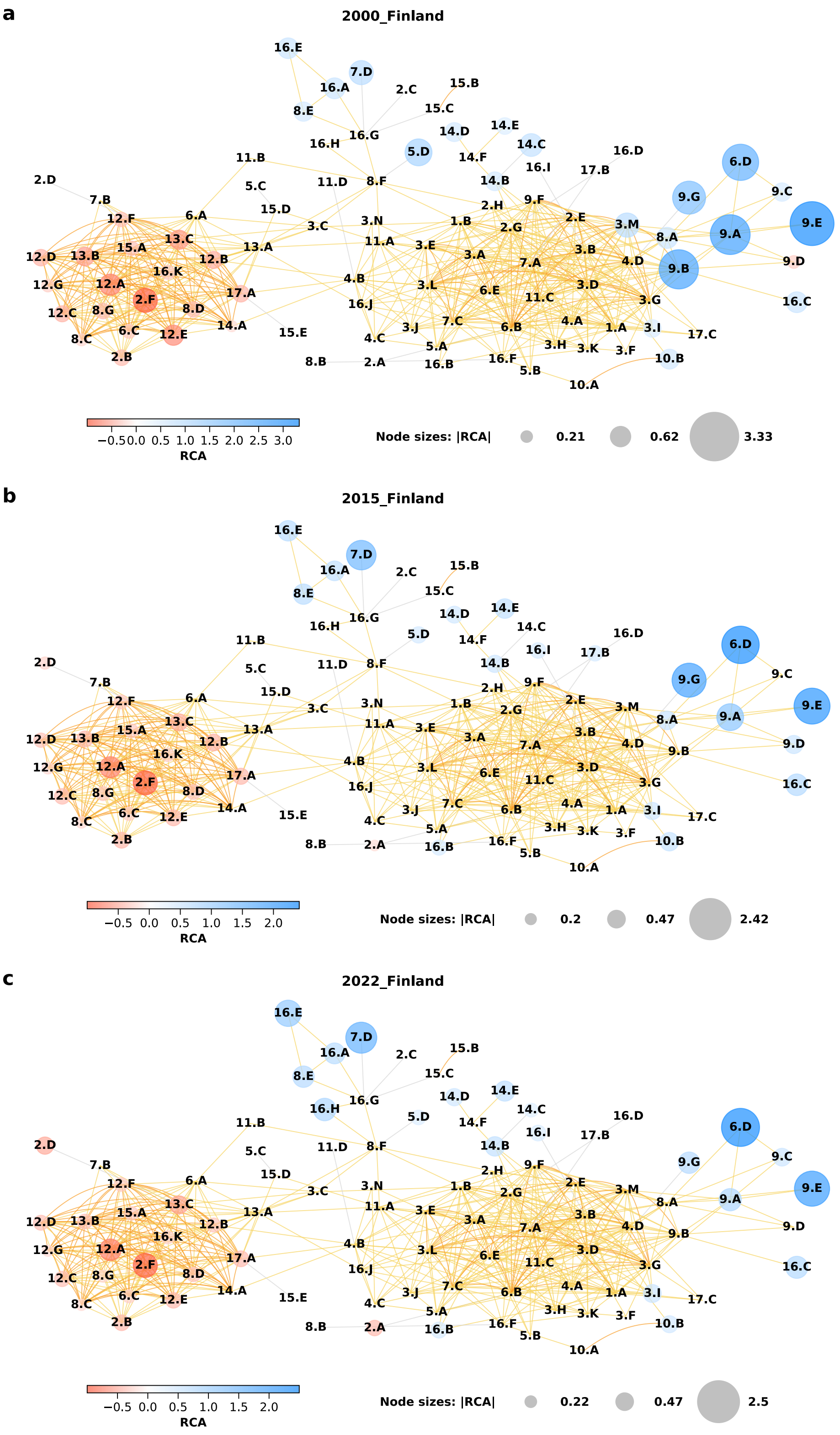

Supplementary Figure 28 | The SDG space of Finland. Panels **a, b, c,** The SDG space in 2000, 2015, and 2022. The nodes in blue and orange represent the top 20 and bottom 20 SDG indicators in revealed comparative advantage (RCA) values, respectively. The node size represents the absolute value of RCA. From Supplementary Figure 12 to 177, countries are ranked by GDP/capita (current US$, 2022).

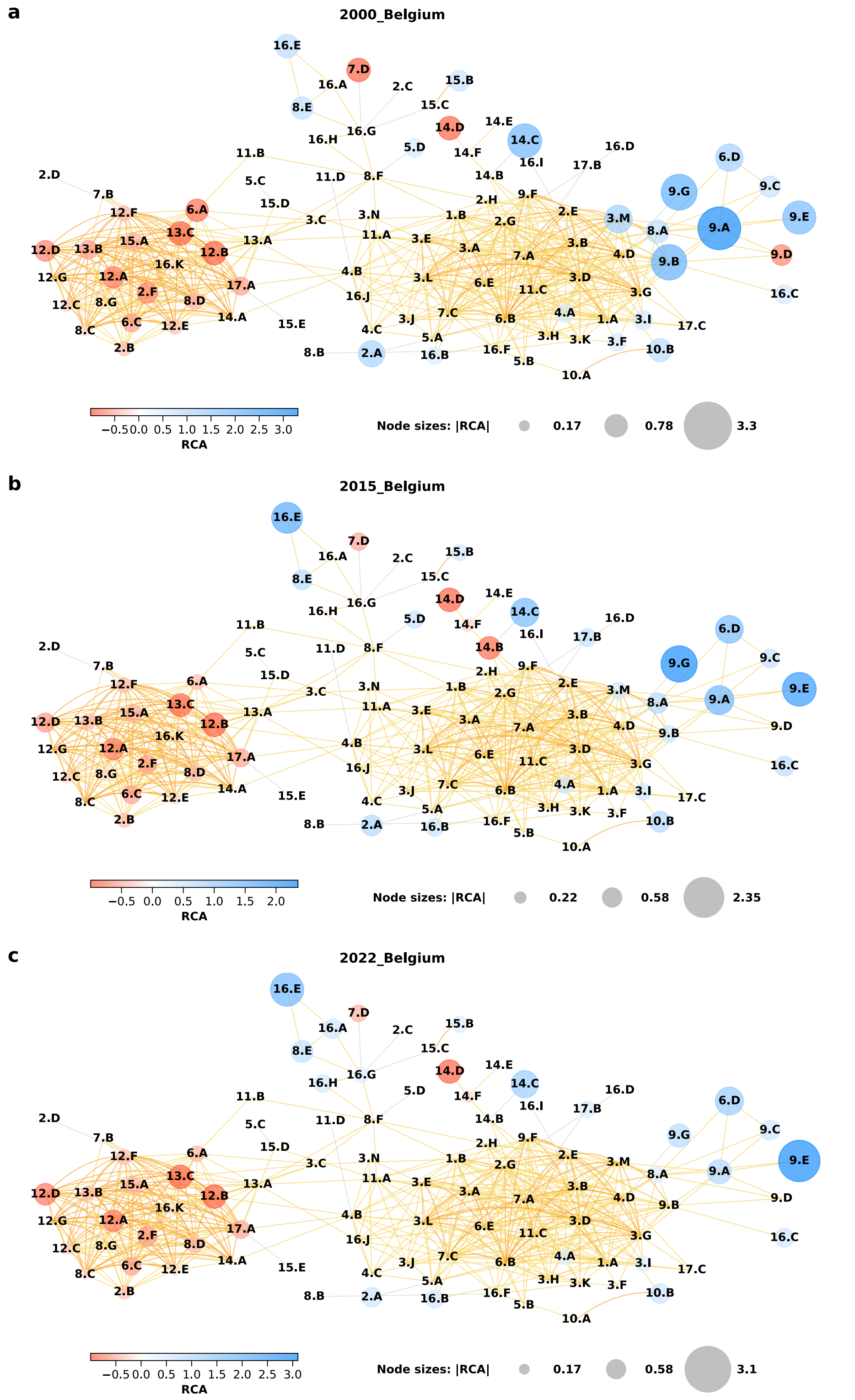

Supplementary Figure 29 | The SDG space of Belgium. Panels **a, b, c,** The SDG space in 2000, 2015, and 2022. The nodes in blue and orange represent the top 20 and bottom 20 SDG indicators in revealed comparative advantage (RCA) values, respectively. The node size represents the absolute value of RCA. From Supplementary Figure 12 to 177, countries are ranked by GDP/capita (current US$, 2022).

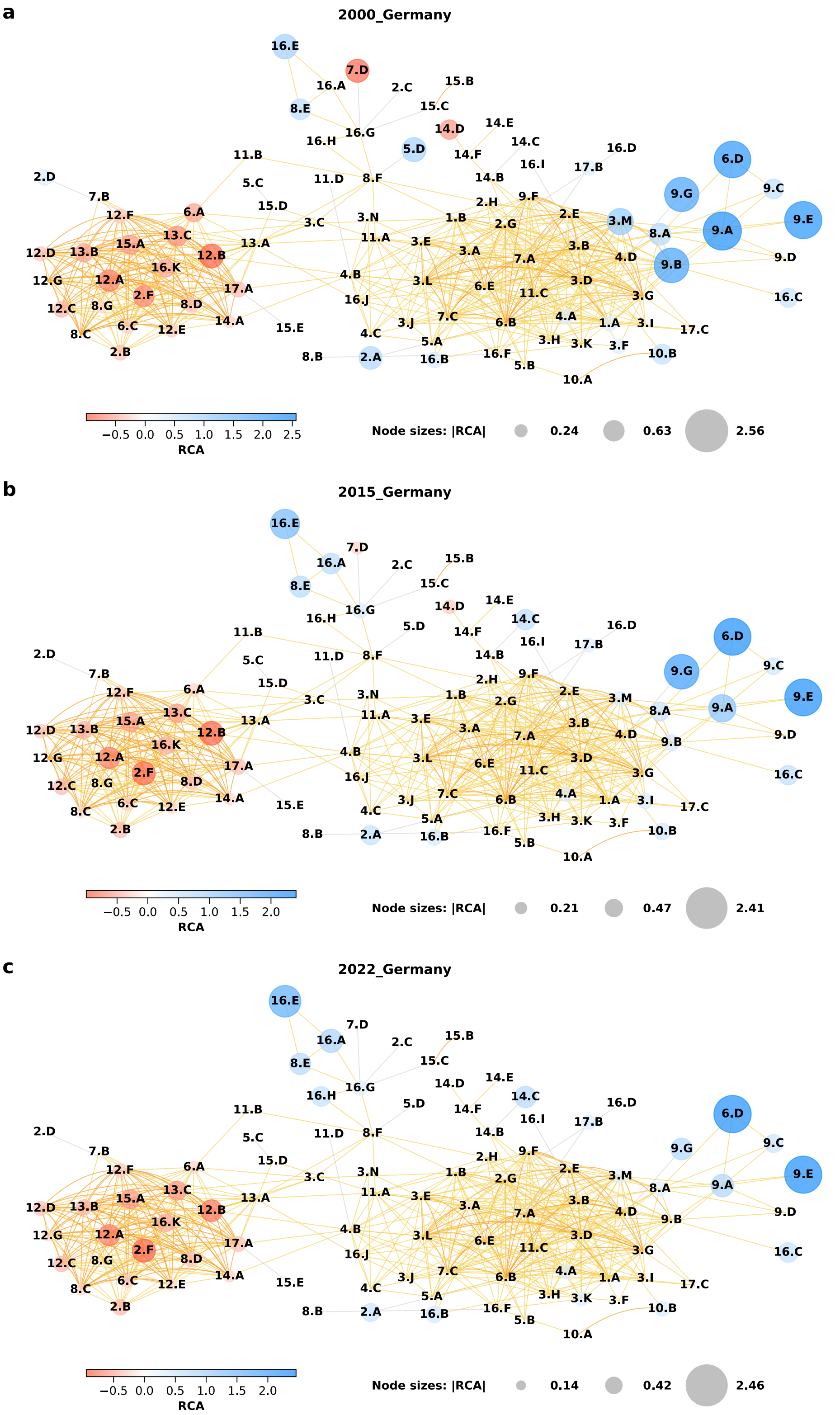

Supplementary Figure 30 | The SDG space of Germany. Panels **a, b, c,** The SDG space in 2000, 2015, and 2022. The nodes in blue and orange represent the top 20 and bottom 20 SDG indicators in revealed comparative advantage (RCA) values, respectively. The node size represents the absolute value of RCA. From Supplementary Figure 12 to 177, countries are ranked by GDP/capita (current US$, 2022).

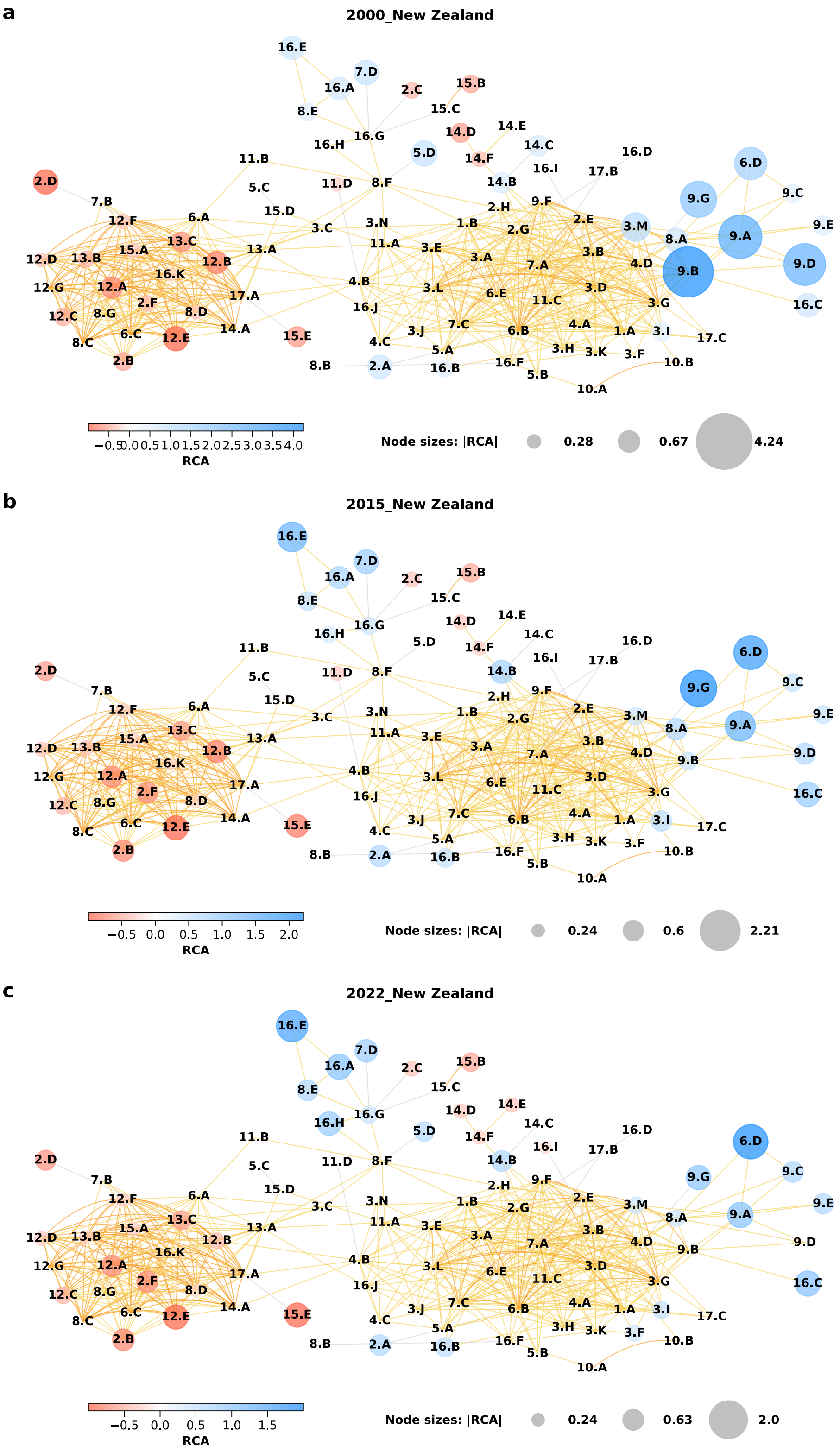

Supplementary Figure 31 | The SDG space of New Zealand. Panels **a, b, c,** The SDG space in 2000, 2015, and 2022. The nodes in blue and orange represent the top 20 and bottom 20 SDG indicators in revealed comparative advantage (RCA) values, respectively. The node size represents the absolute value of RCA. From Supplementary Figure 12 to 177, countries are ranked by GDP/capita (current US$, 2022).

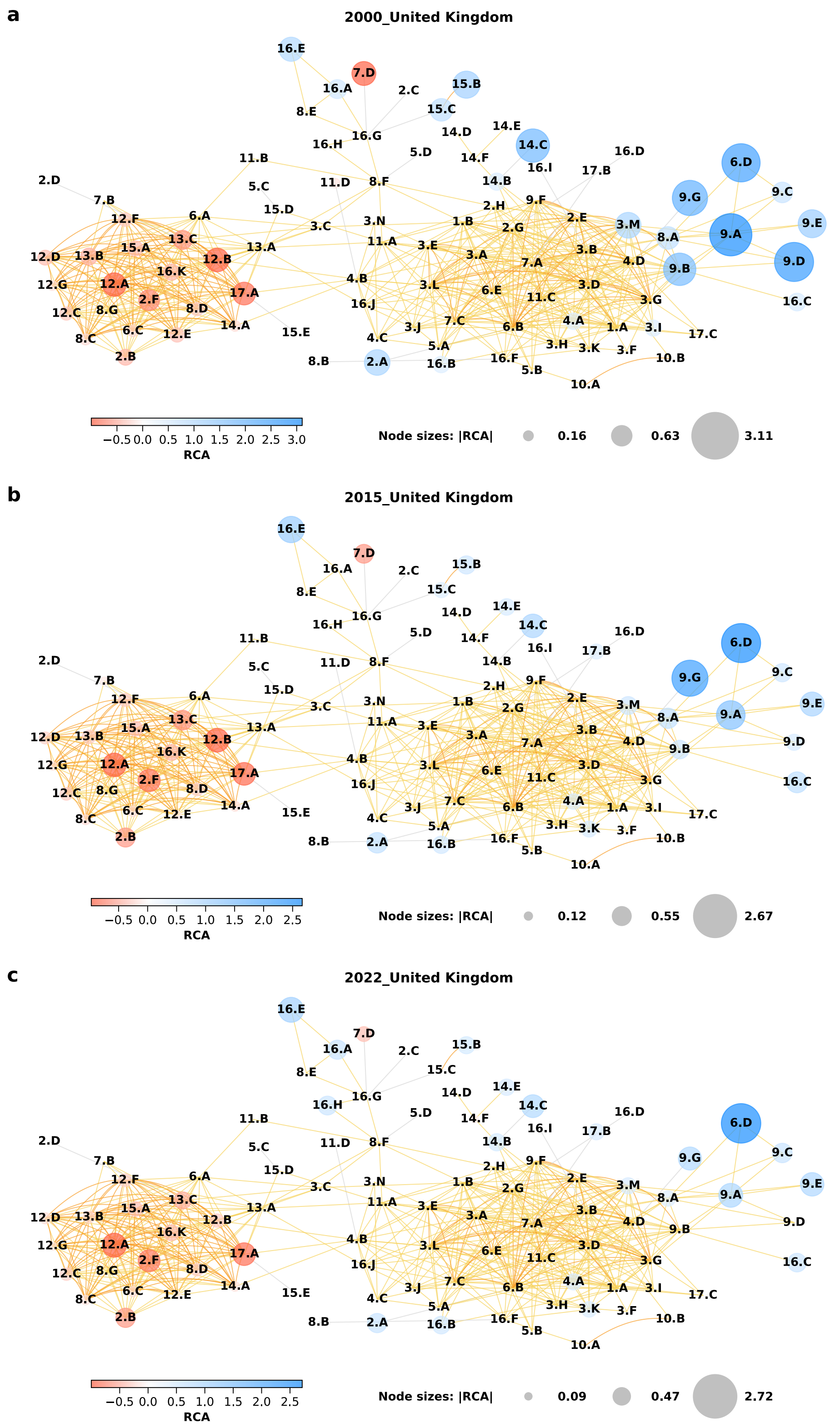

Supplementary Figure 32 | The SDG space of United Kingdom. Panels **a, b, c,** The SDG space in 2000, 2015, and 2022. The nodes in blue and orange represent the top 20 and bottom 20 SDG indicators in revealed comparative advantage (RCA) values, respectively. The node size represents the absolute value of RCA. From Supplementary Figure 12 to 177, countries are ranked by GDP/capita (current US$, 2022).

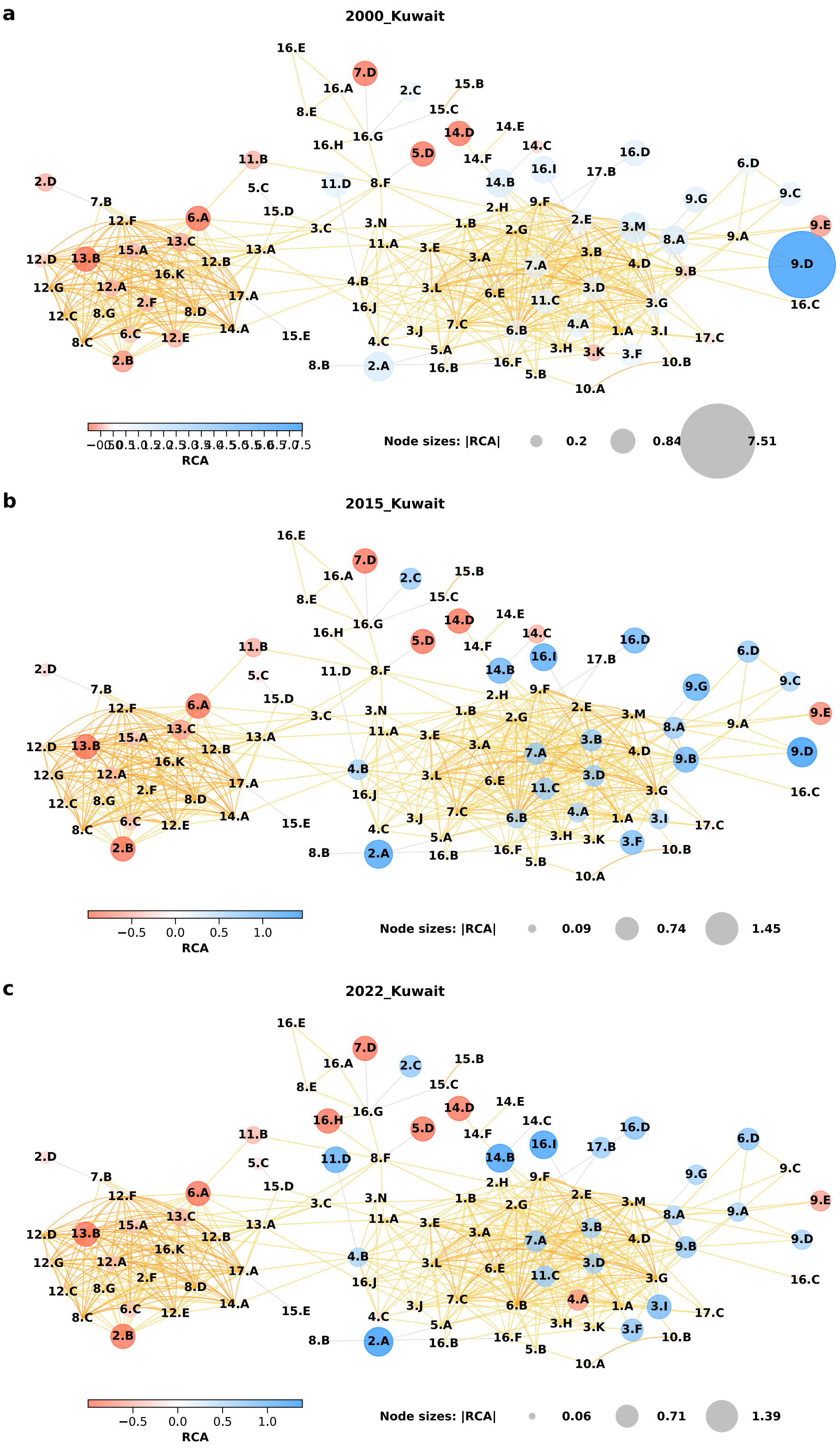

Supplementary Figure 33 | The SDG space of Kuwait. Panels **a, b, c,** The SDG space in 2000, 2015, and 2022. The nodes in blue and orange represent the top 20 and bottom 20 SDG indicators in revealed comparative advantage (RCA) values, respectively. The node size represents the absolute value of RCA. From Supplementary Figure 12 to 177, countries are ranked by GDP/capita (current US$, 2022).

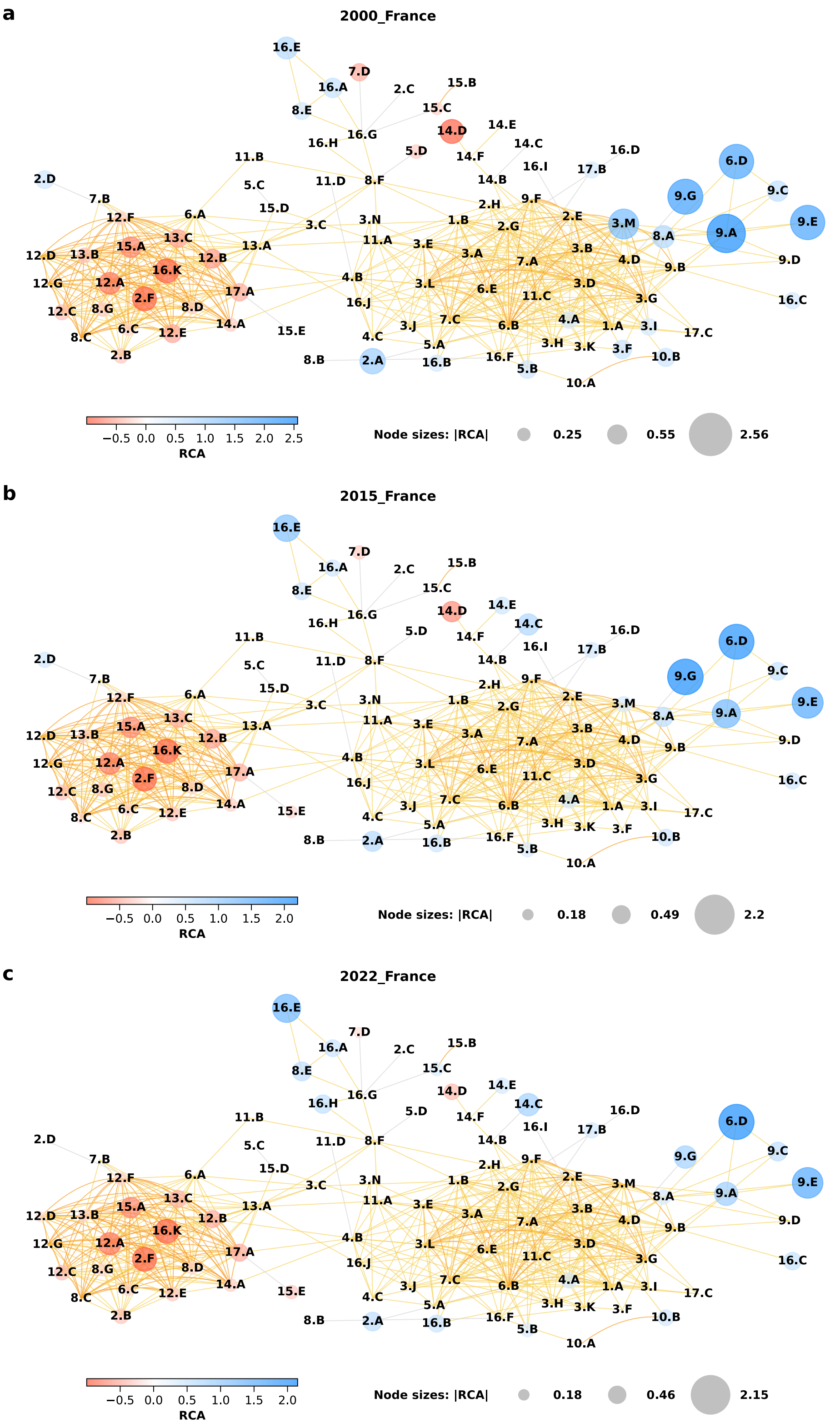

Supplementary Figure 34 | The SDG space of France. Panels **a, b, c,** The SDG space in 2000, 2015, and 2022. The nodes in blue and orange represent the top 20 and bottom 20 SDG indicators in revealed comparative advantage (RCA) values, respectively. The node size represents the absolute value of RCA. From Supplementary Figure 12 to 177, countries are ranked by GDP/capita (current US$, 2022).

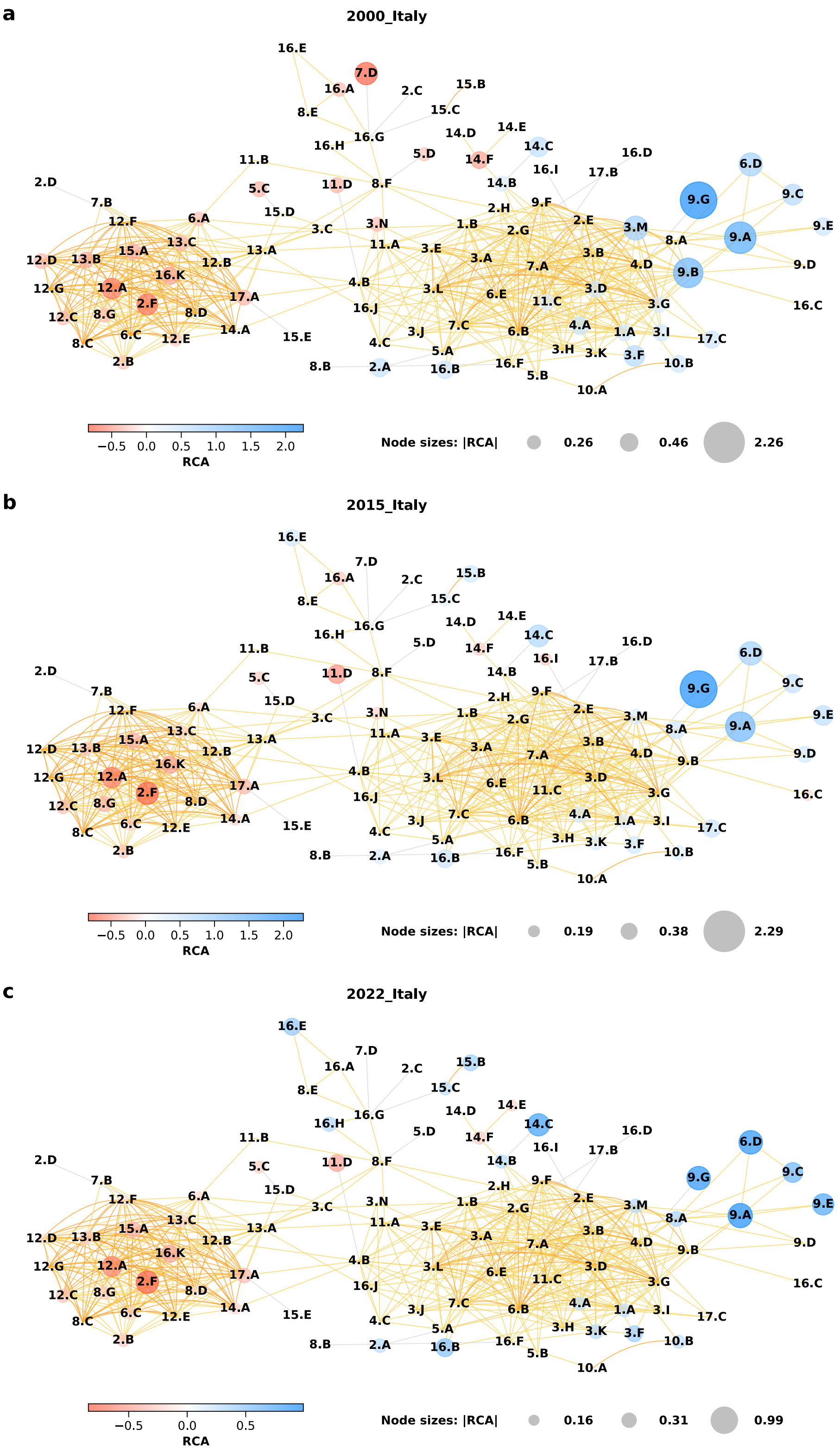

Supplementary Figure 35 | The SDG space of Italy. Panels **a, b, c,** The SDG space in 2000, 2015, and 2022. The nodes in blue and orange represent the top 20 and bottom 20 SDG indicators in revealed comparative advantage (RCA) values, respectively. The node size represents the absolute value of RCA. From Supplementary Figure 12 to 177, countries are ranked by GDP/capita (current US$, 2022).

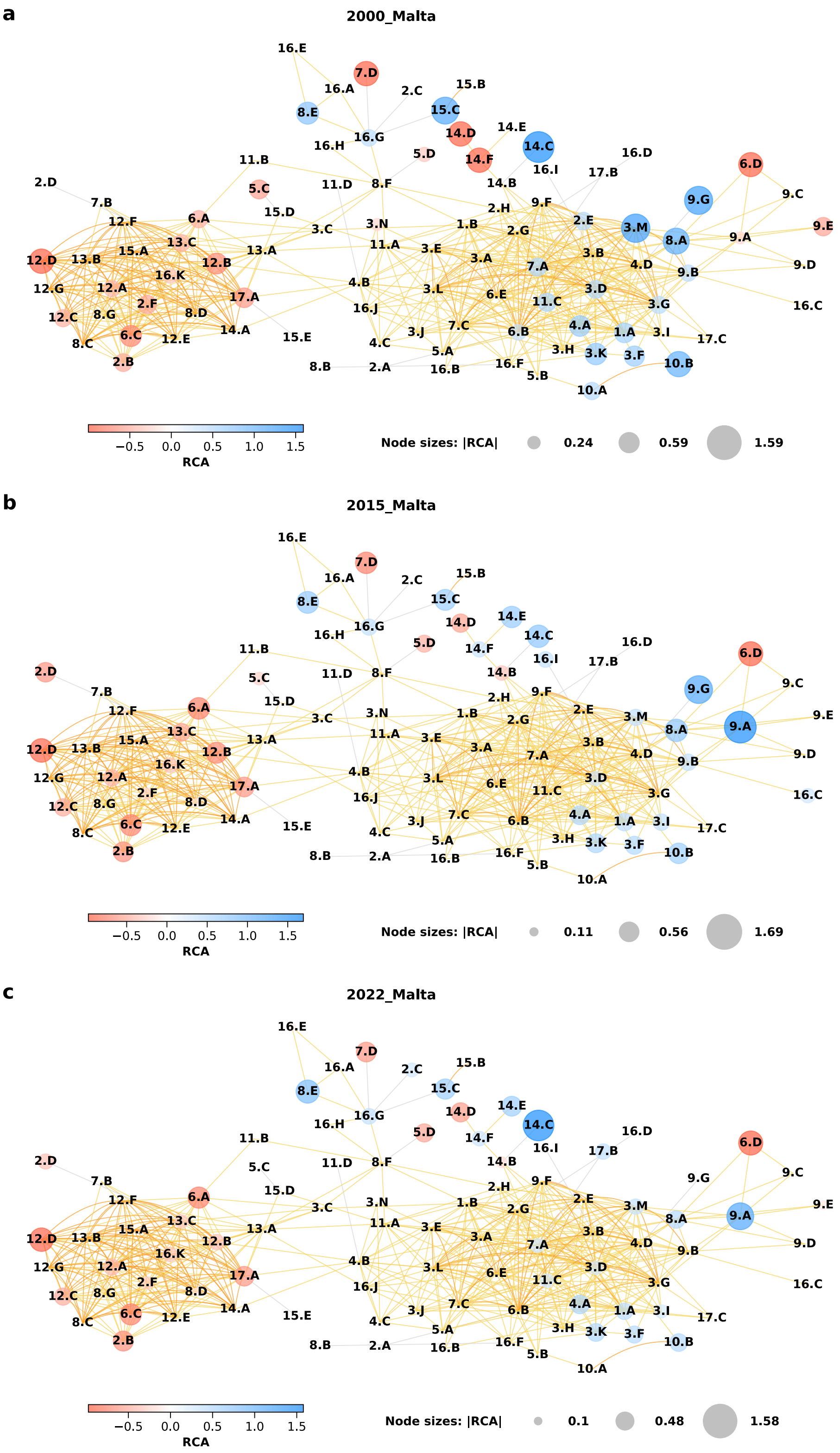

Supplementary Figure 36 | The SDG space of Malta. Panels **a, b, c,** The SDG space in 2000, 2015, and 2022. The nodes in blue and orange represent the top 20 and bottom 20 SDG indicators in revealed comparative advantage (RCA) values, respectively. The node size represents the absolute value of RCA. From Supplementary Figure 12 to 177, countries are ranked by GDP/capita (current US$, 2022).

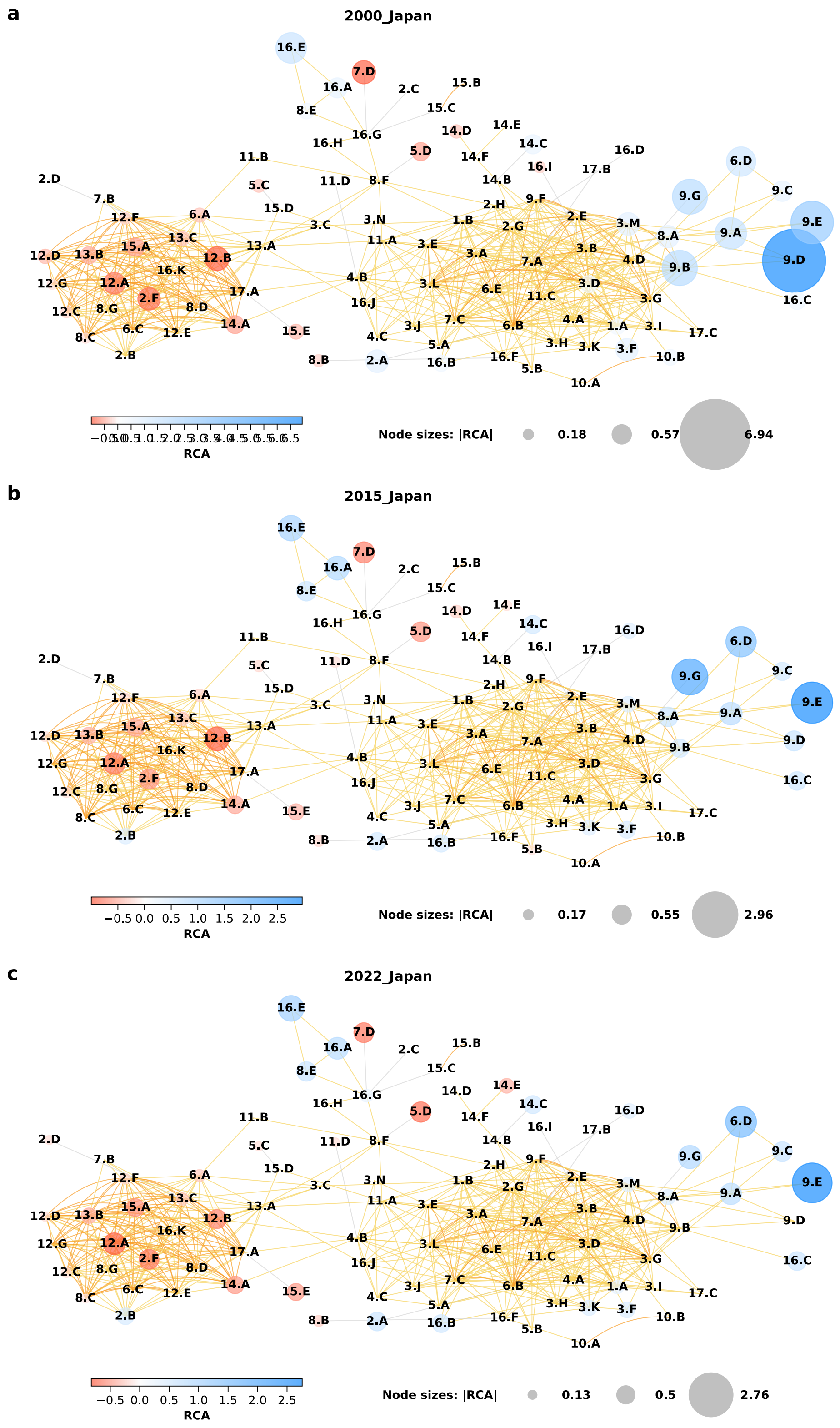

Supplementary Figure 37 | The SDG space of Japan. Panels **a, b, c,** The SDG space in 2000, 2015, and 2022. The nodes in blue and orange represent the top 20 and bottom 20 SDG indicators in revealed comparative advantage (RCA) values, respectively. The node size represents the absolute value of RCA. From Supplementary Figure 12 to 177, countries are ranked by GDP/capita (current US$, 2022).

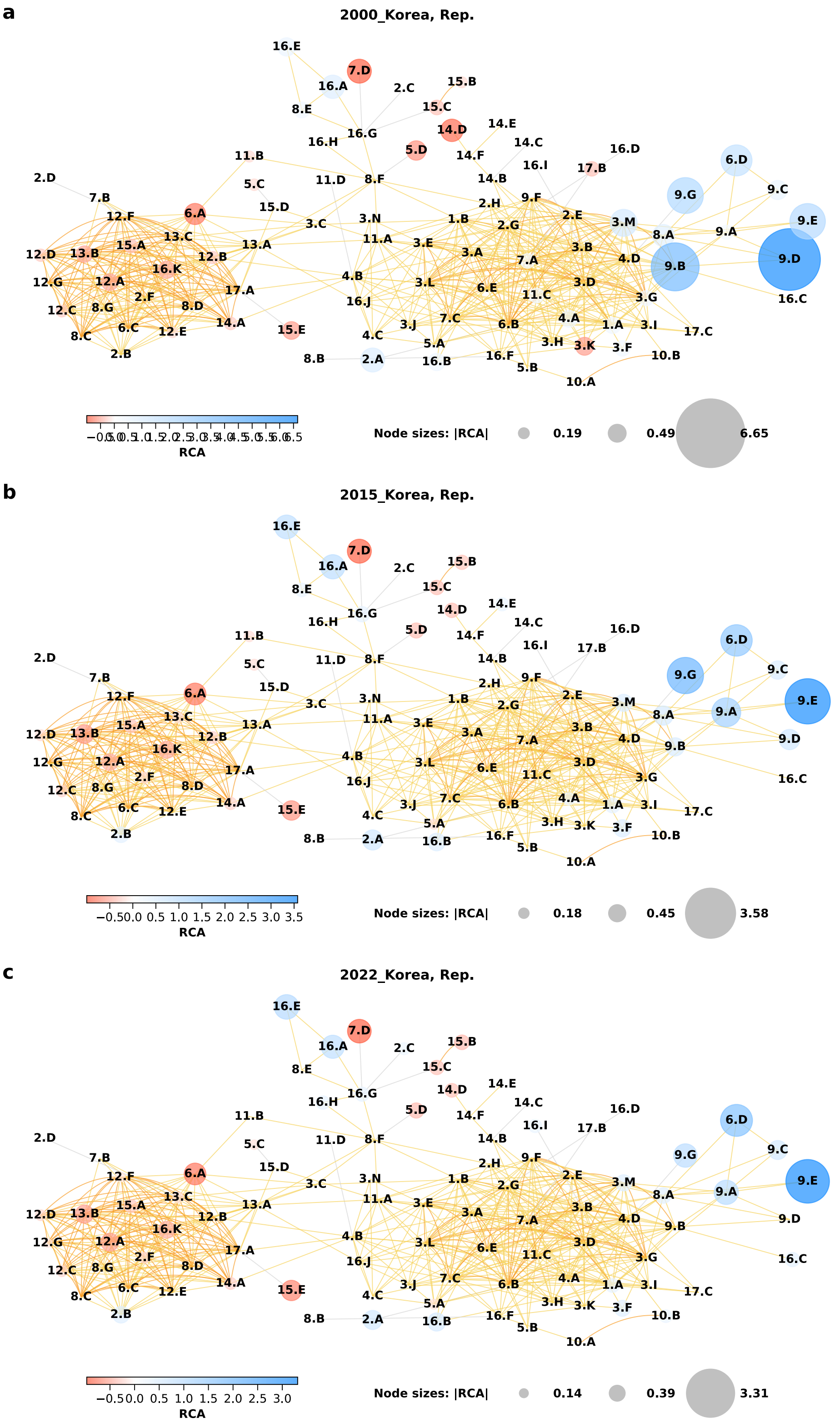

Supplementary Figure 38 | The SDG space of Korea, Rep.. Panels **a, b, c,** The SDG space in 2000, 2015, and 2022. The nodes in blue and orange represent the top 20 and bottom 20 SDG indicators in revealed comparative advantage (RCA) values, respectively. The node size represents the absolute value of RCA. From Supplementary Figure 12 to 177, countries are ranked by GDP/capita (current US$, 2022).

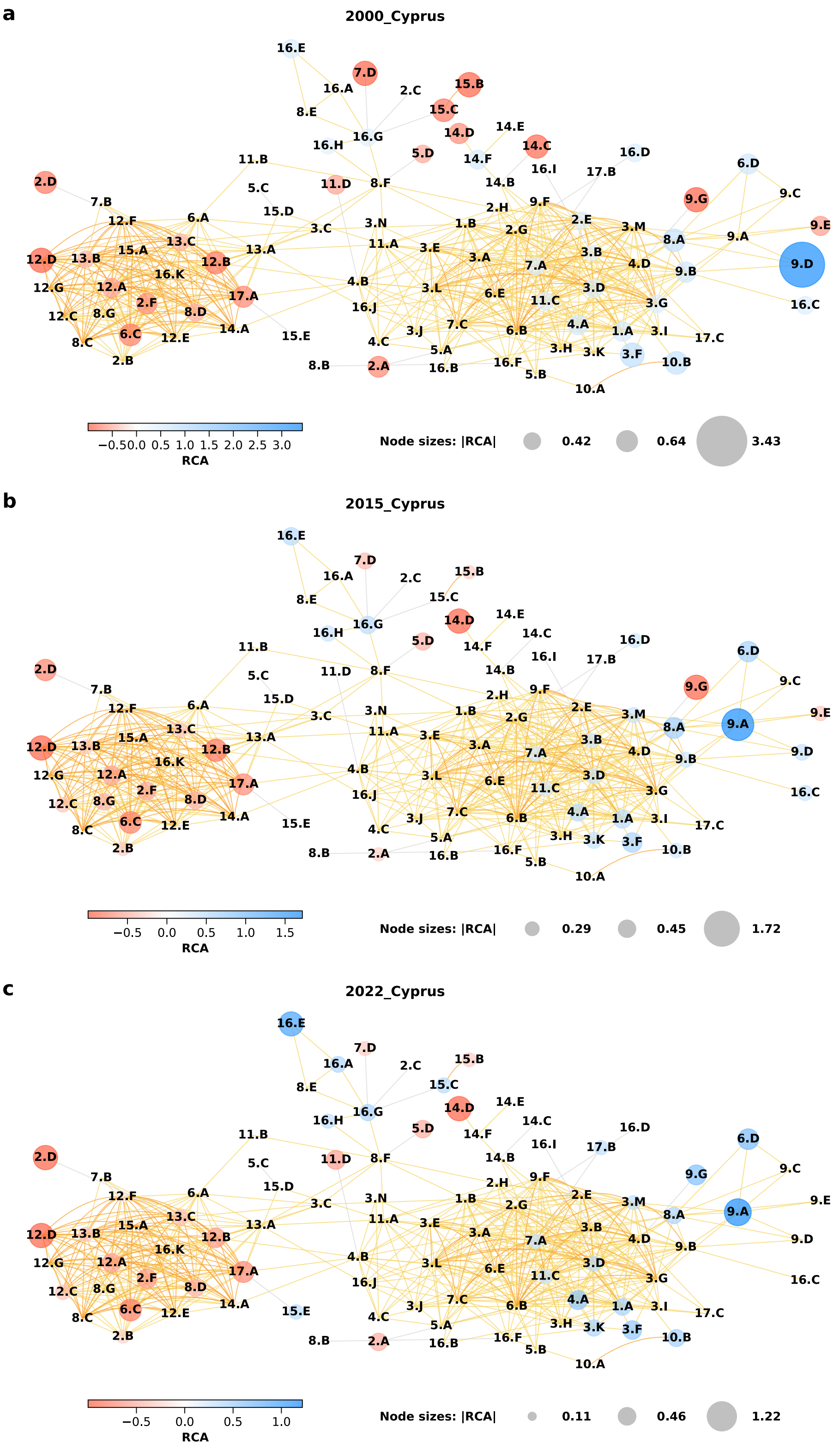

Supplementary Figure 39 | The SDG space of Cyprus. Panels **a, b, c,** The SDG space in 2000, 2015, and 2022. The nodes in blue and orange represent the top 20 and bottom 20 SDG indicators in revealed comparative advantage (RCA) values, respectively. The node size represents the absolute value of RCA. From Supplementary Figure 12 to 177, countries are ranked by GDP/capita (current US$, 2022).

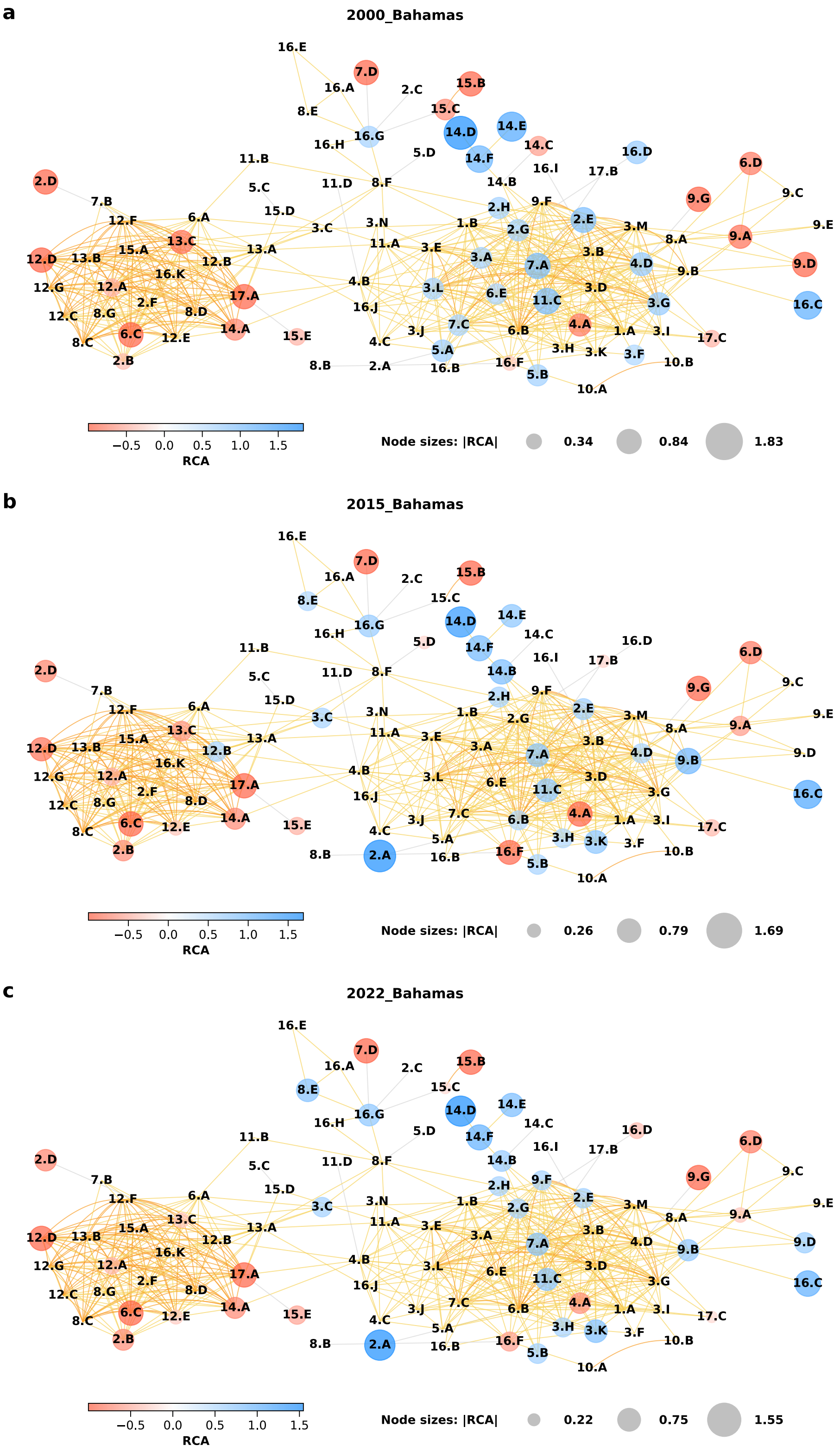

Supplementary Figure 40 | The SDG space of Bahamas. Panels **a, b, c,** The SDG space in 2000, 2015, and 2022. The nodes in blue and orange represent the top 20 and bottom 20 SDG indicators in revealed comparative advantage (RCA) values, respectively. The node size represents the absolute value of RCA. From Supplementary Figure 12 to 177, countries are ranked by GDP/capita (current US$, 2022).

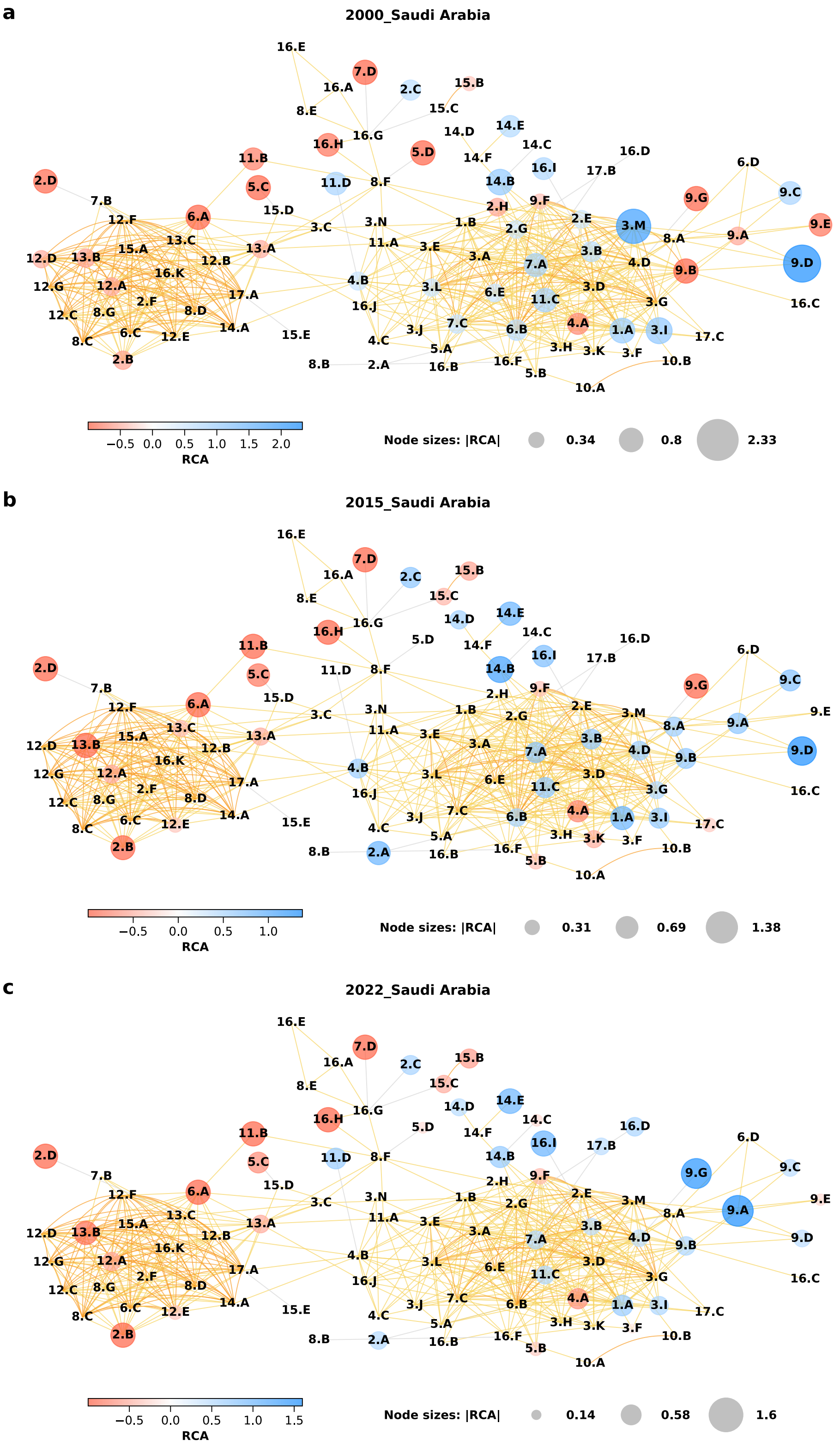

Supplementary Figure 41 | The SDG space of Saudi Arabia. Panels **a, b, c,** The SDG space in 2000, 2015, and 2022. The nodes in blue and orange represent the top 20 and bottom 20 SDG indicators in revealed comparative advantage (RCA) values, respectively. The node size represents the absolute value of RCA. From Supplementary Figure 12 to 177, countries are ranked by GDP/capita (current US$, 2022).

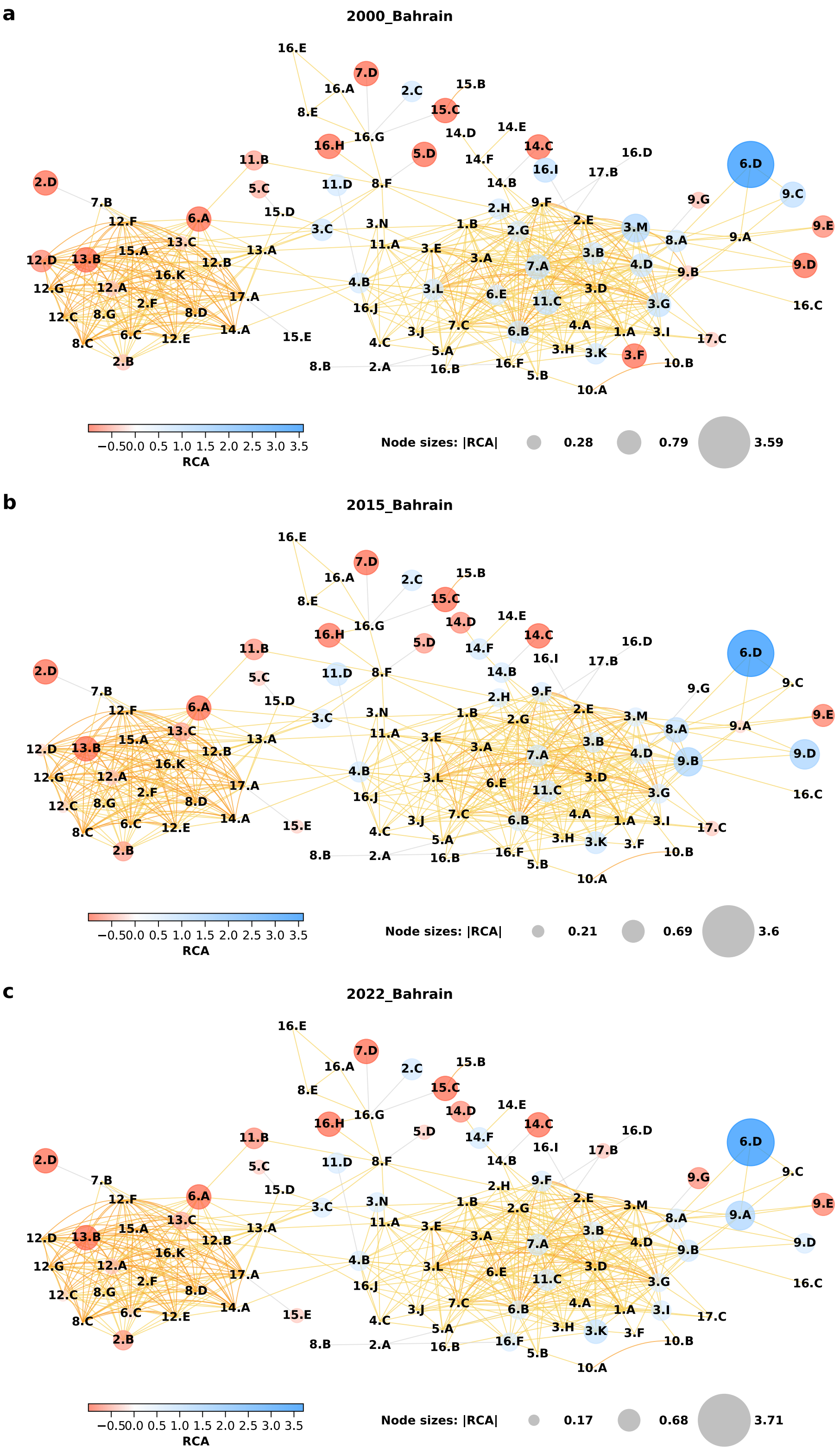

Supplementary Figure 42 | The SDG space of Bahrain. Panels **a, b, c,** The SDG space in 2000, 2015, and 2022. The nodes in blue and orange represent the top 20 and bottom 20 SDG indicators in revealed comparative advantage (RCA) values, respectively. The node size represents the absolute value of RCA. From Supplementary Figure 12 to 177, countries are ranked by GDP/capita (current US$, 2022).

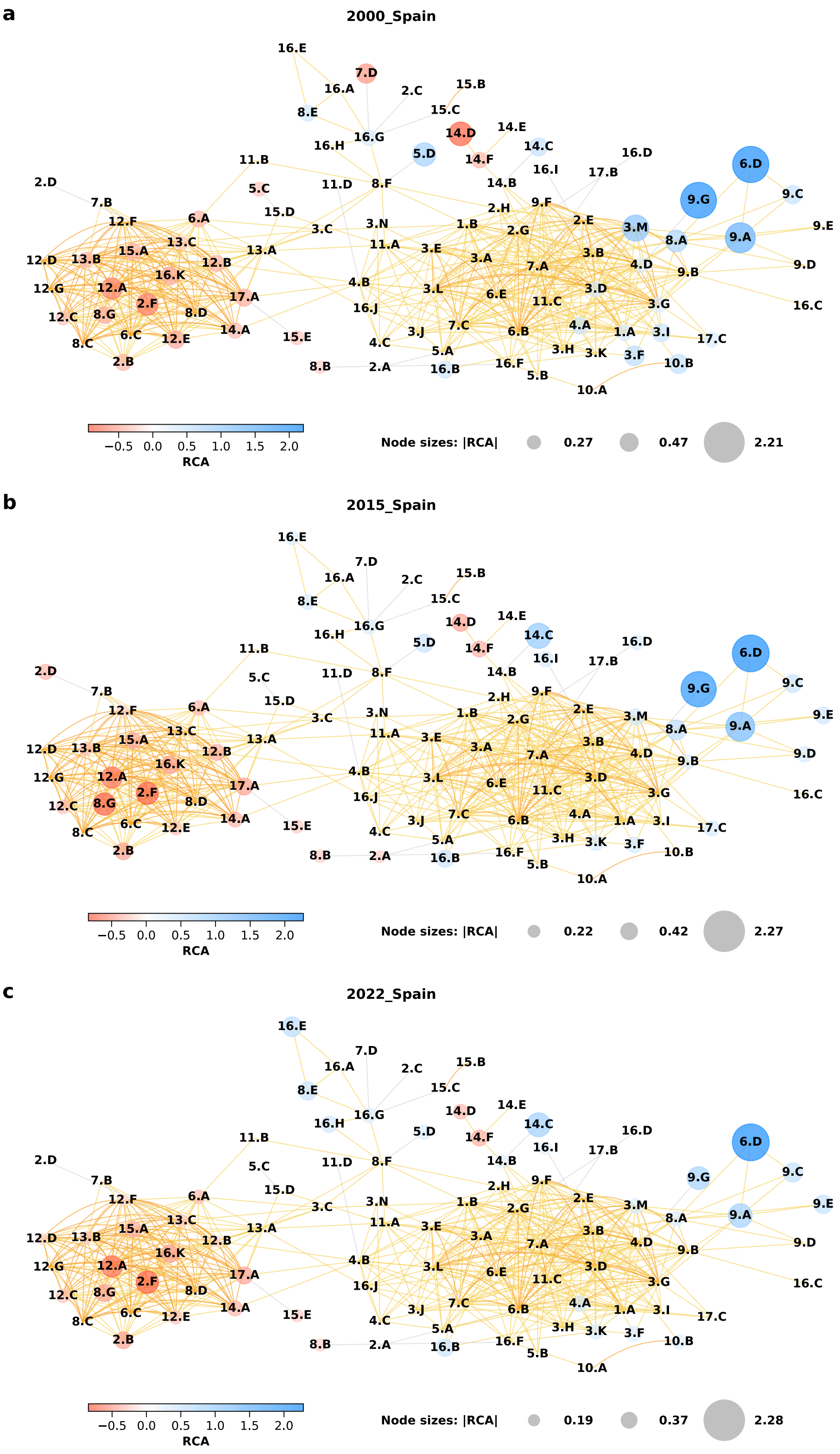

Supplementary Figure 43 | The SDG space of Spain. Panels **a, b, c,** The SDG space in 2000, 2015, and 2022. The nodes in blue and orange represent the top 20 and bottom 20 SDG indicators in revealed comparative advantage (RCA) values, respectively. The node size represents the absolute value of RCA. From Supplementary Figure 12 to 177, countries are ranked by GDP/capita (current US$, 2022).

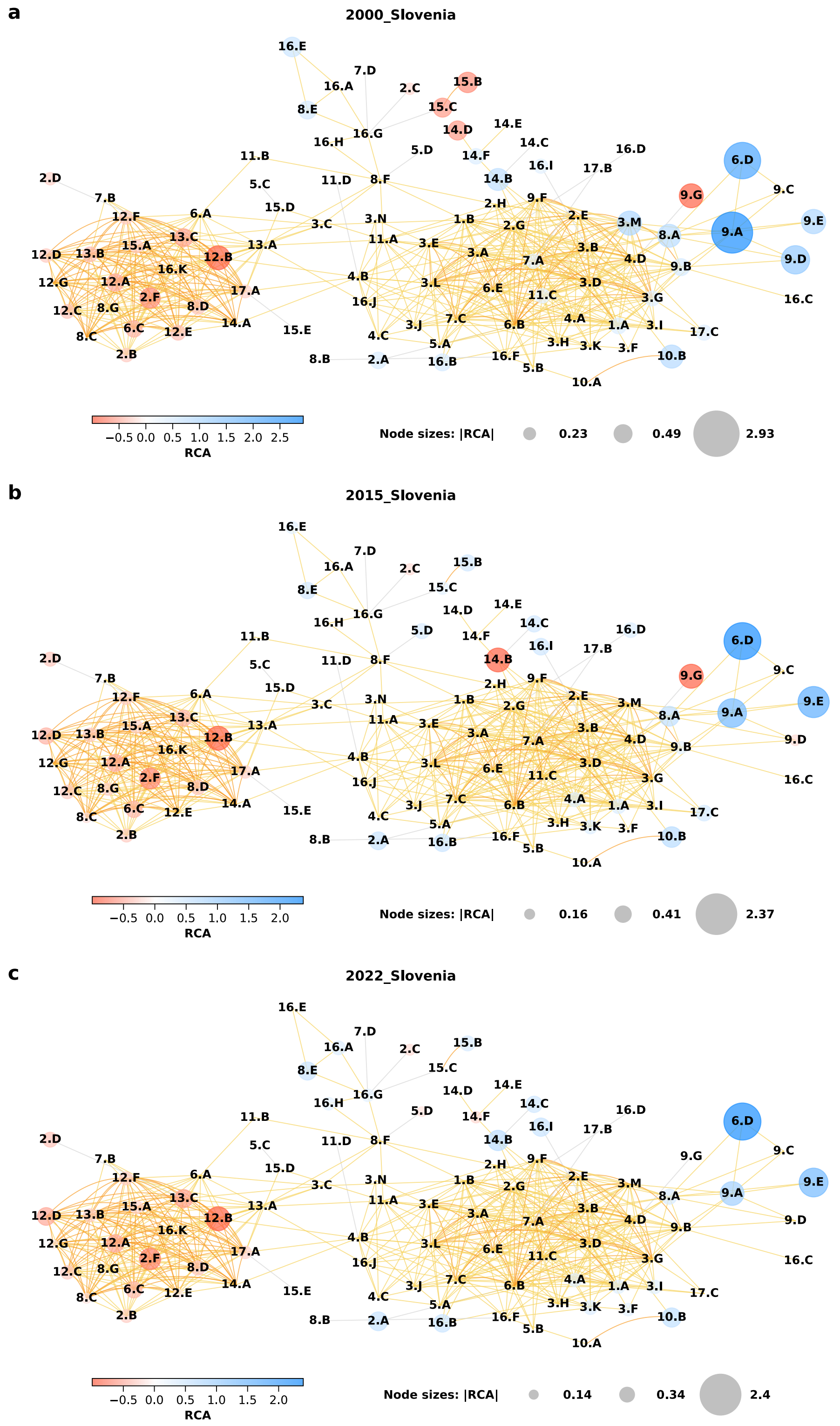

Supplementary Figure 44 | The SDG space of Slovenia. Panels **a, b, c,** The SDG space in 2000, 2015, and 2022. The nodes in blue and orange represent the top 20 and bottom 20 SDG indicators in revealed comparative advantage (RCA) values, respectively. The node size represents the absolute value of RCA. From Supplementary Figure 12 to 177, countries are ranked by GDP/capita (current US$, 2022).

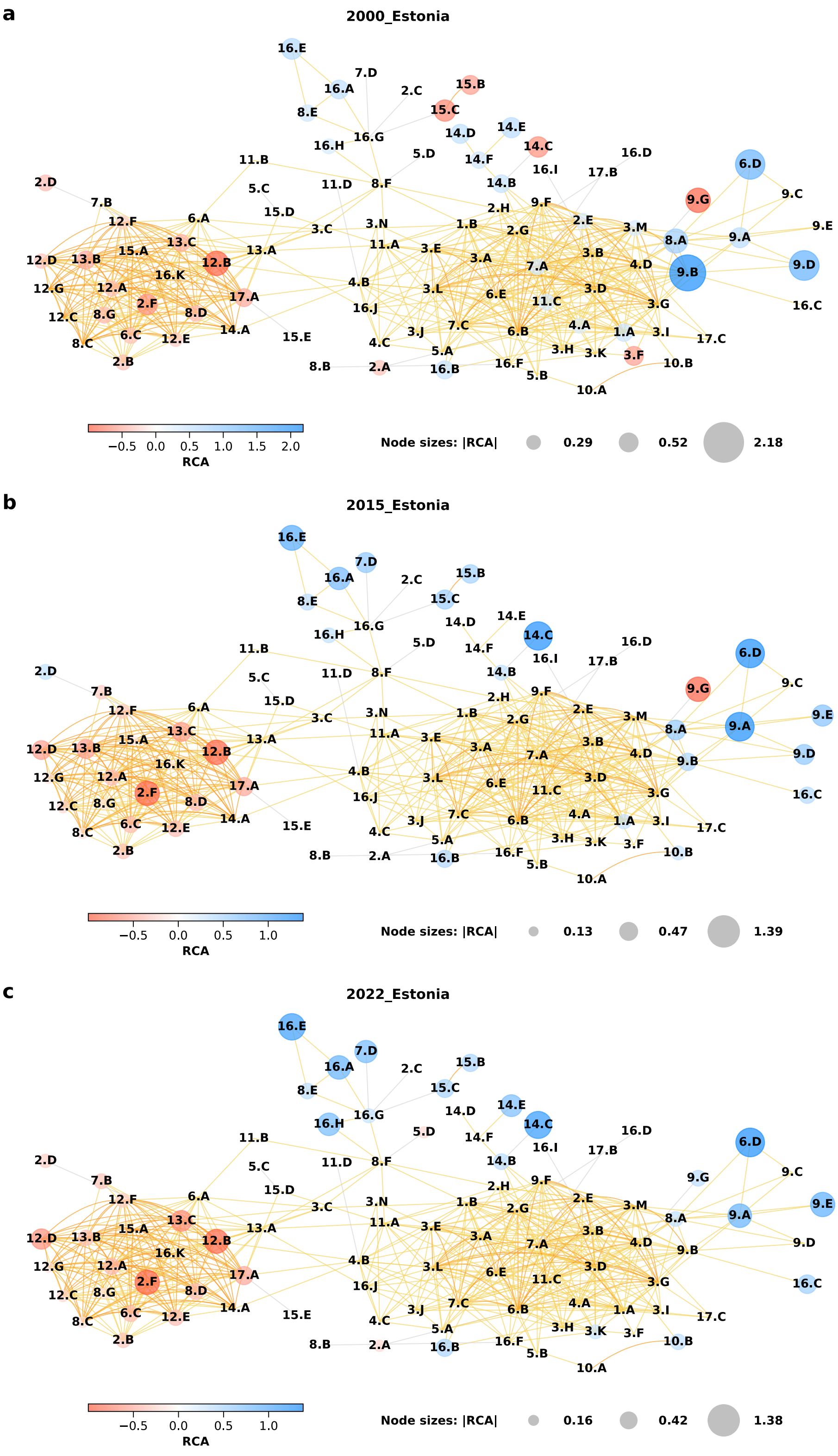

Supplementary Figure 45 | The SDG space of Estonia. Panels **a, b, c,** The SDG space in 2000, 2015, and 2022. The nodes in blue and orange represent the top 20 and bottom 20 SDG indicators in revealed comparative advantage (RCA) values, respectively. The node size represents the absolute value of RCA. From Supplementary Figure 12 to 177, countries are ranked by GDP/capita (current US$, 2022).

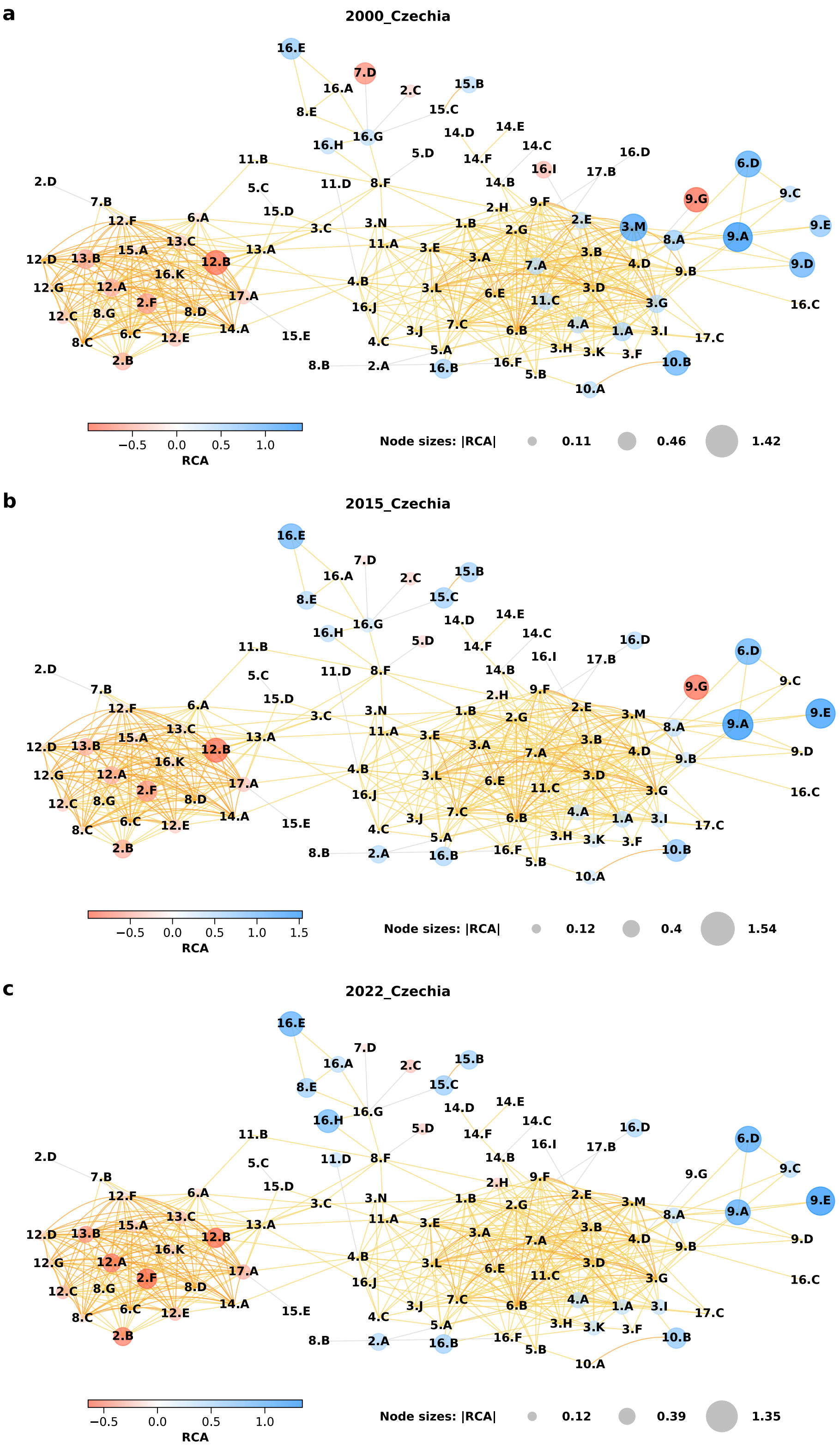

Supplementary Figure 46 | The SDG space of Czechia. Panels **a, b, c,** The SDG space in 2000, 2015, and 2022. The nodes in blue and orange represent the top 20 and bottom 20 SDG indicators in revealed comparative advantage (RCA) values, respectively. The node size represents the absolute value of RCA. From Supplementary Figure 12 to 177, countries are ranked by GDP/capita (current US$, 2022).

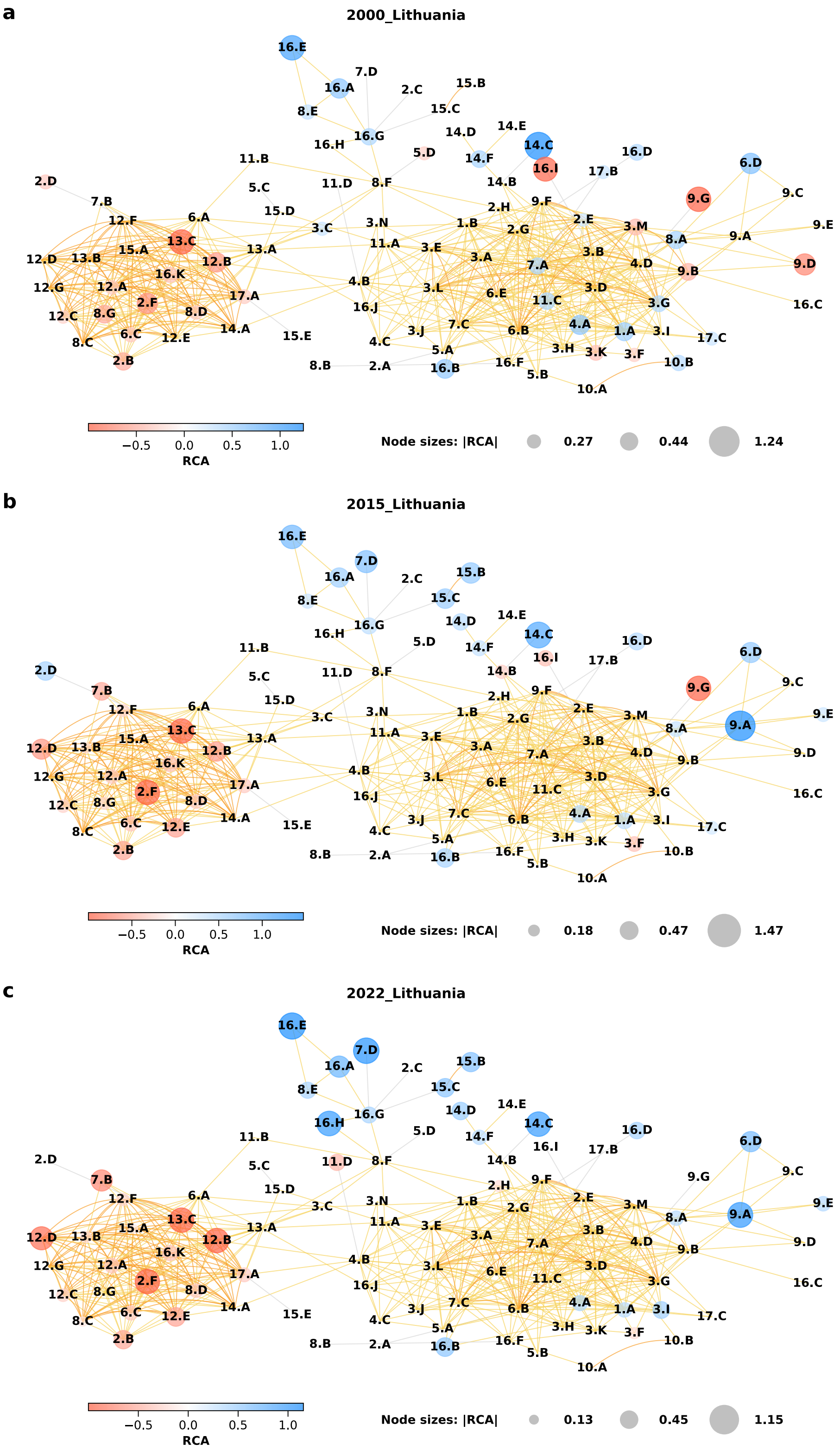

Supplementary Figure 47 | The SDG space of Lithuania. Panels **a, b, c,** The SDG space in 2000, 2015, and 2022. The nodes in blue and orange represent the top 20 and bottom 20 SDG indicators in revealed comparative advantage (RCA) values, respectively. The node size represents the absolute value of RCA. From Supplementary Figure 12 to 177, countries are ranked by GDP/capita (current US$, 2022).

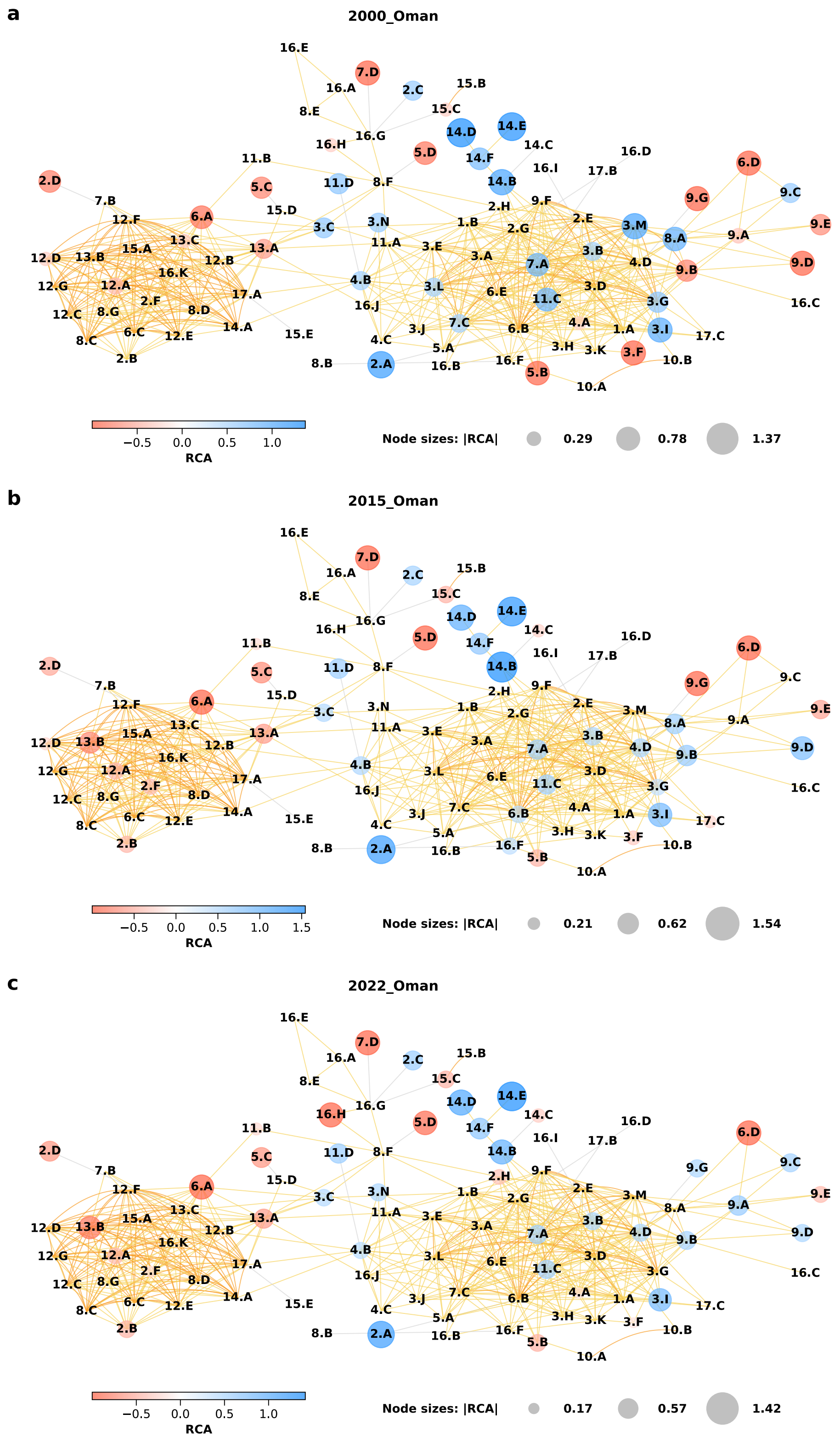

Supplementary Figure 48 | The SDG space of Oman. Panels **a, b, c,** The SDG space in 2000, 2015, and 2022. The nodes in blue and orange represent the top 20 and bottom 20 SDG indicators in revealed comparative advantage (RCA) values, respectively. The node size represents the absolute value of RCA. From Supplementary Figure 12 to 177, countries are ranked by GDP/capita (current US$, 2022).

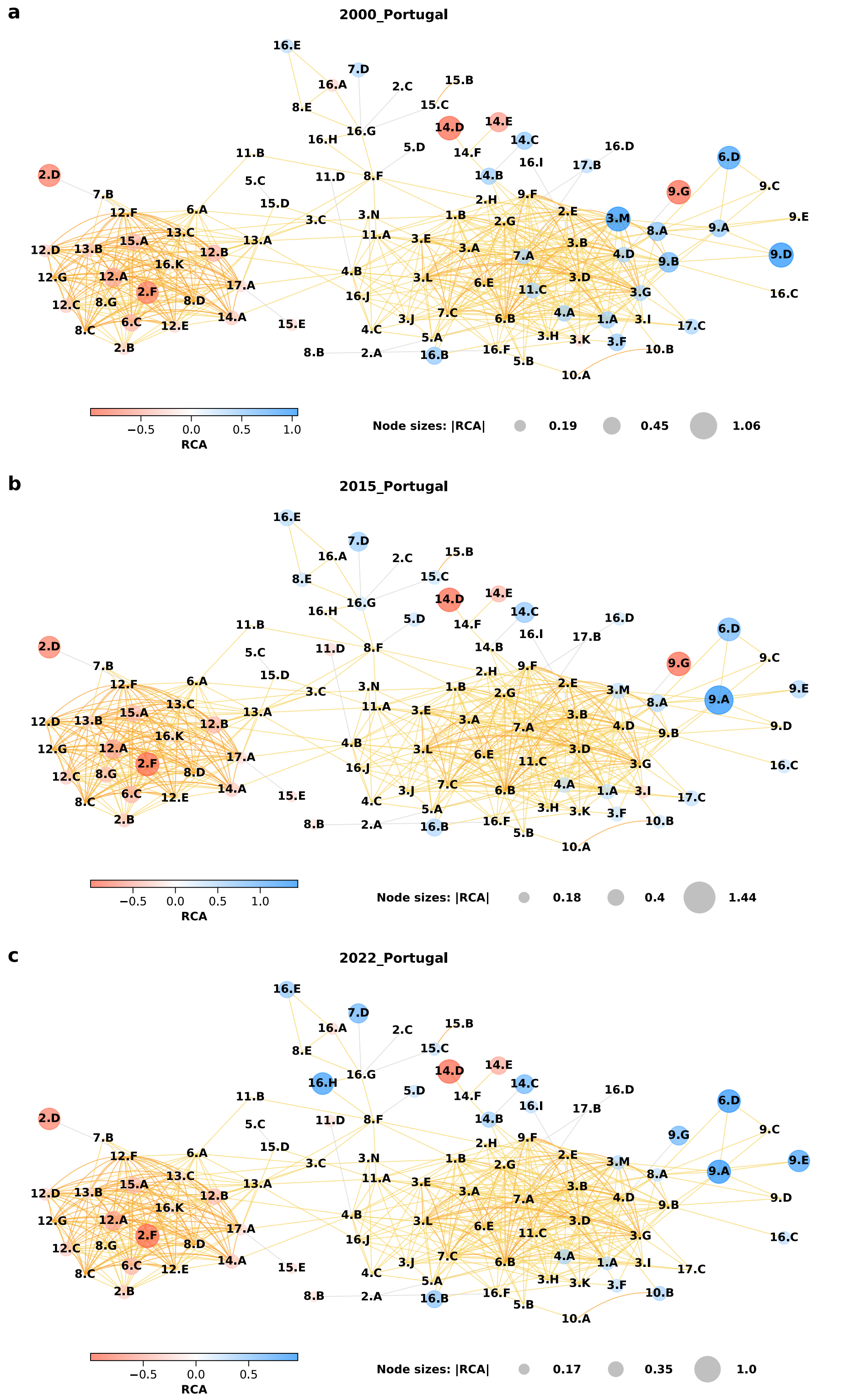

Supplementary Figure 49 | The SDG space of Portugal. Panels **a, b, c,** The SDG space in 2000, 2015, and 2022. The nodes in blue and orange represent the top 20 and bottom 20 SDG indicators in revealed comparative advantage (RCA) values, respectively. The node size represents the absolute value of RCA. From Supplementary Figure 12 to 177, countries are ranked by GDP/capita (current US$, 2022).

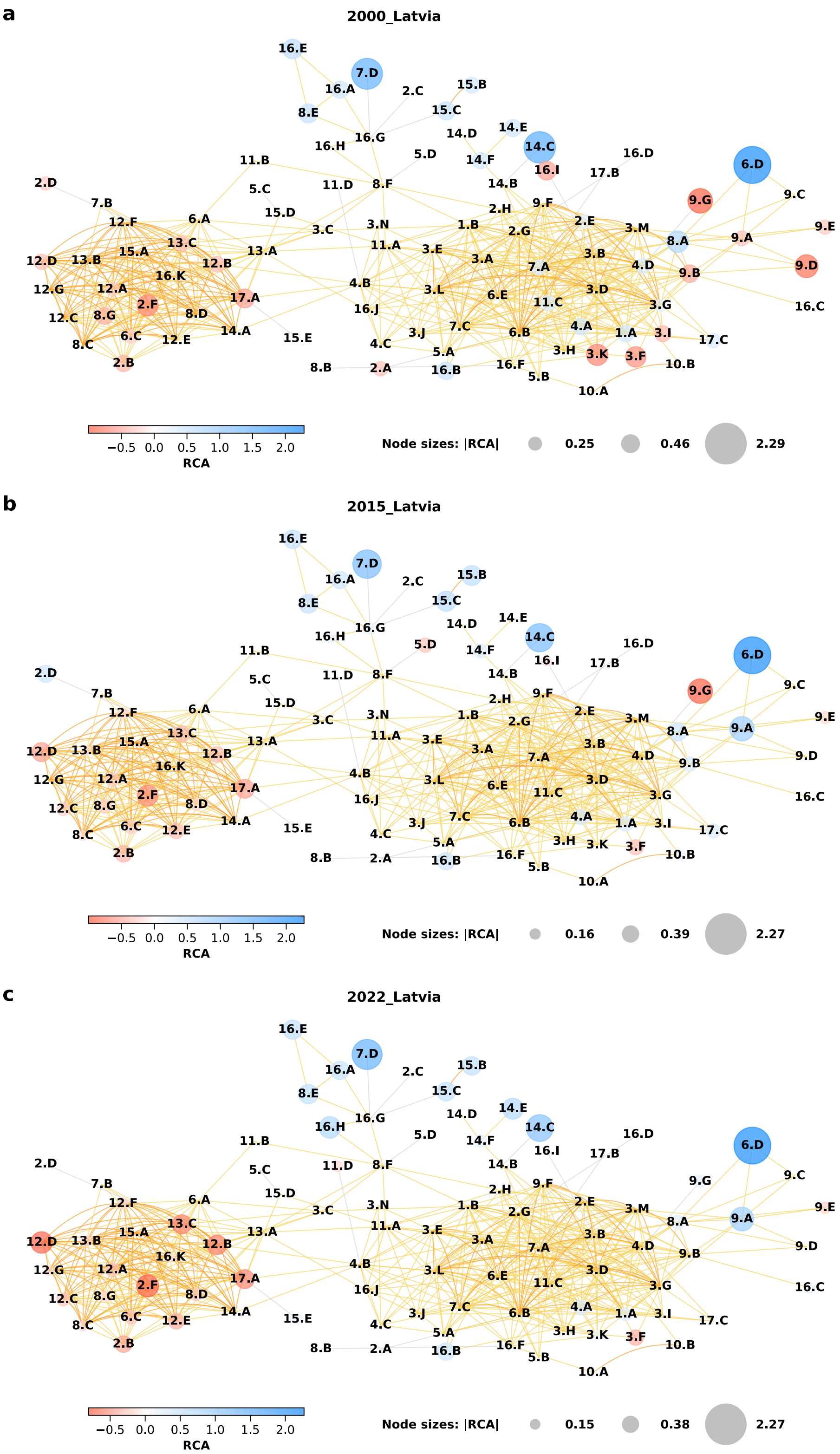

Supplementary Figure 50 | The SDG space of Latvia. Panels **a, b, c,** The SDG space in 2000, 2015, and 2022. The nodes in blue and orange represent the top 20 and bottom 20 SDG indicators in revealed comparative advantage (RCA) values, respectively. The node size represents the absolute value of RCA. From Supplementary Figure 12 to 177, countries are ranked by GDP/capita (current US$, 2022).

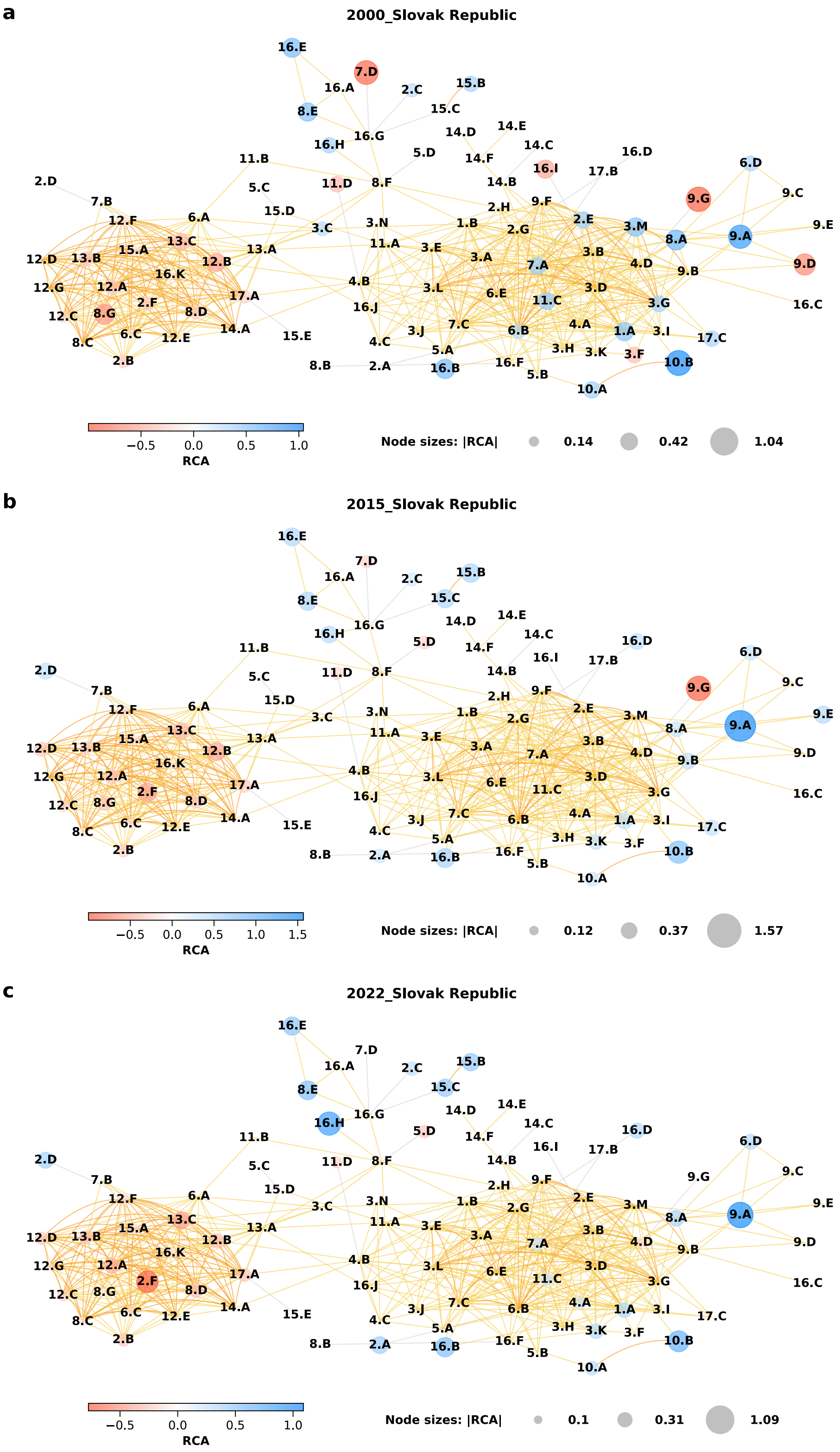

Supplementary Figure 51 | The SDG space of Slovak Republic. Panels **a, b, c,** The SDG space in 2000, 2015, and 2022. The nodes in blue and orange represent the top 20 and bottom 20 SDG indicators in revealed comparative advantage (RCA) values, respectively. The node size represents the absolute value of RCA. From Supplementary Figure 12 to 177, countries are ranked by GDP/capita (current US$, 2022).

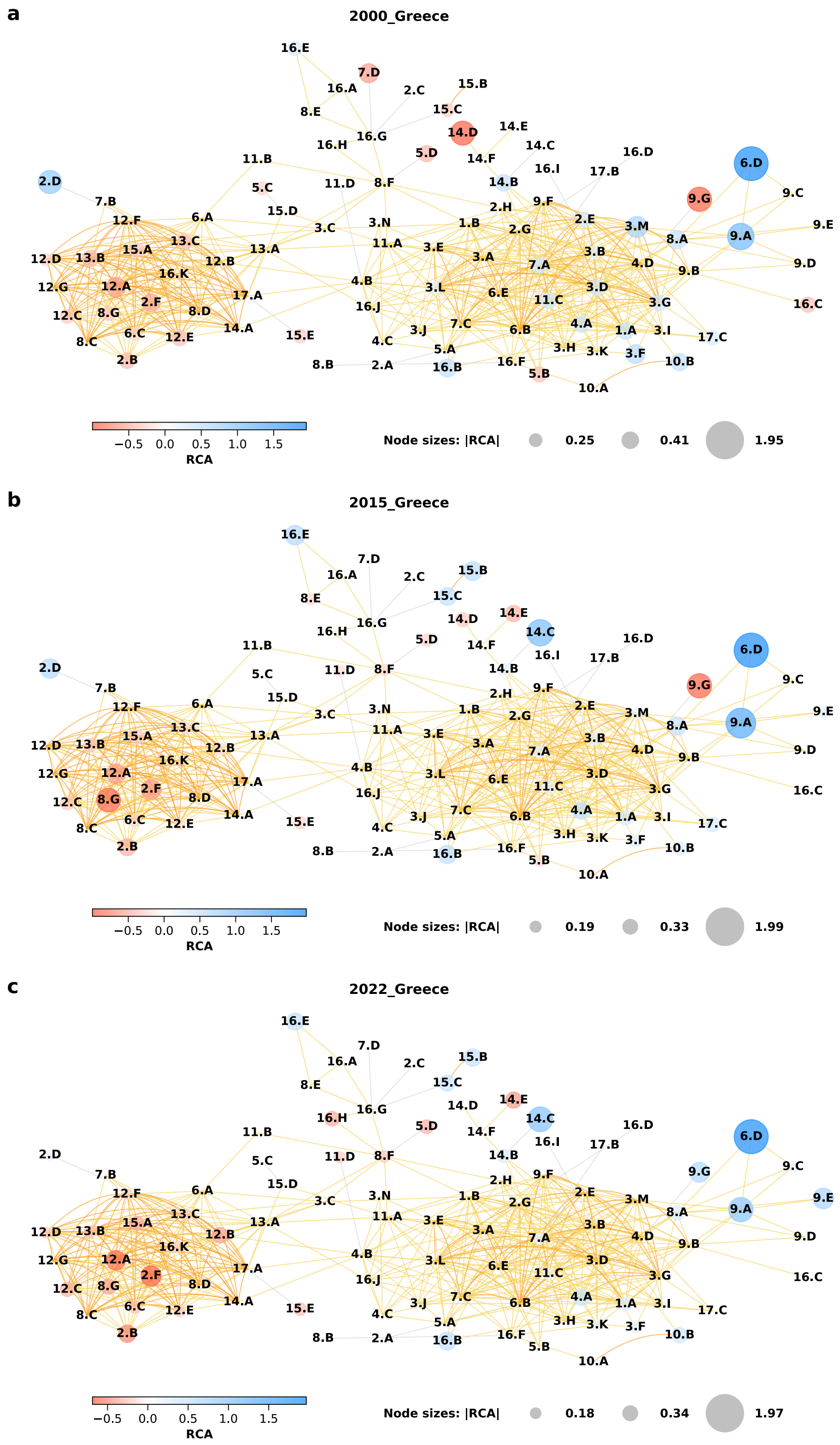

Supplementary Figure 52 | The SDG space of Greece. Panels **a, b, c,** The SDG space in 2000, 2015, and 2022. The nodes in blue and orange represent the top 20 and bottom 20 SDG indicators in revealed comparative advantage (RCA) values, respectively. The node size represents the absolute value of RCA. From Supplementary Figure 12 to 177, countries are ranked by GDP/capita (current US$, 2022).

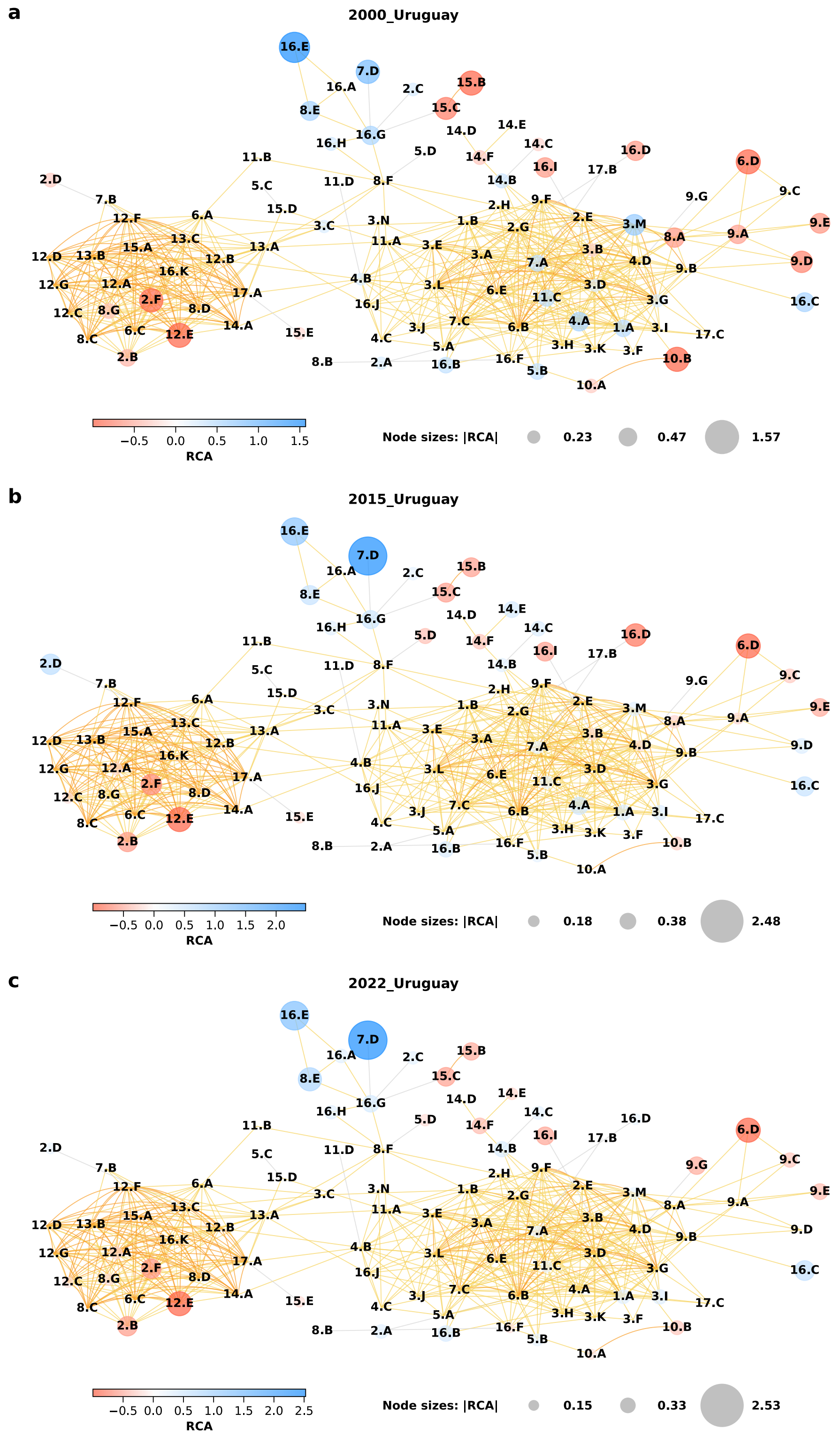

Supplementary Figure 53 | The SDG space of Uruguay. Panels **a, b, c,** The SDG space in 2000, 2015, and 2022. The nodes in blue and orange represent the top 20 and bottom 20 SDG indicators in revealed comparative advantage (RCA) values, respectively. The node size represents the absolute value of RCA. From Supplementary Figure 12 to 177, countries are ranked by GDP/capita (current US$, 2022).

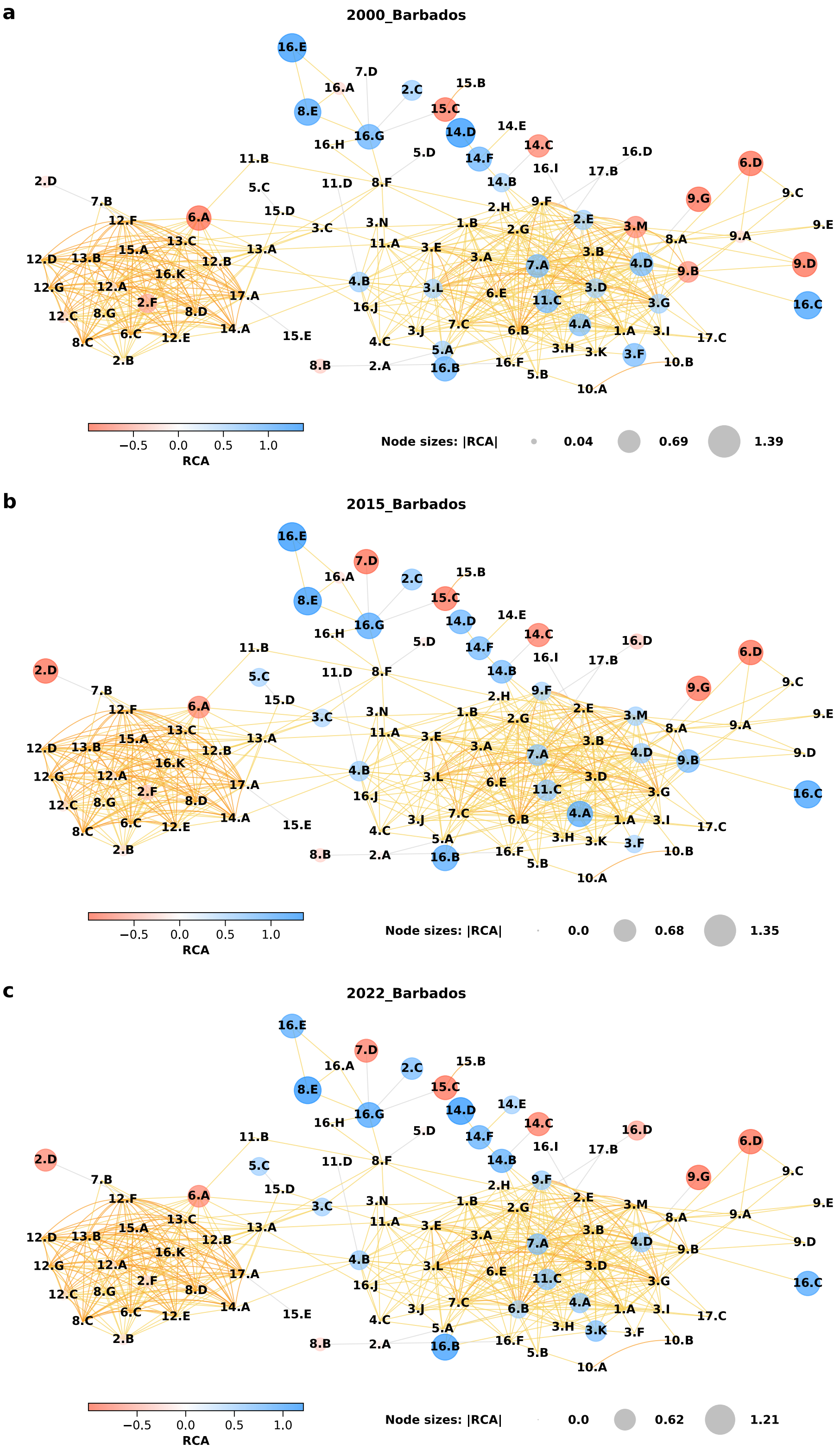

Supplementary Figure 54 | The SDG space of Barbados. Panels **a, b, c,** The SDG space in 2000, 2015, and 2022. The nodes in blue and orange represent the top 20 and bottom 20 SDG indicators in revealed comparative advantage (RCA) values, respectively. The node size represents the absolute value of RCA. From Supplementary Figure 12 to 177, countries are ranked by GDP/capita (current US$, 2022).

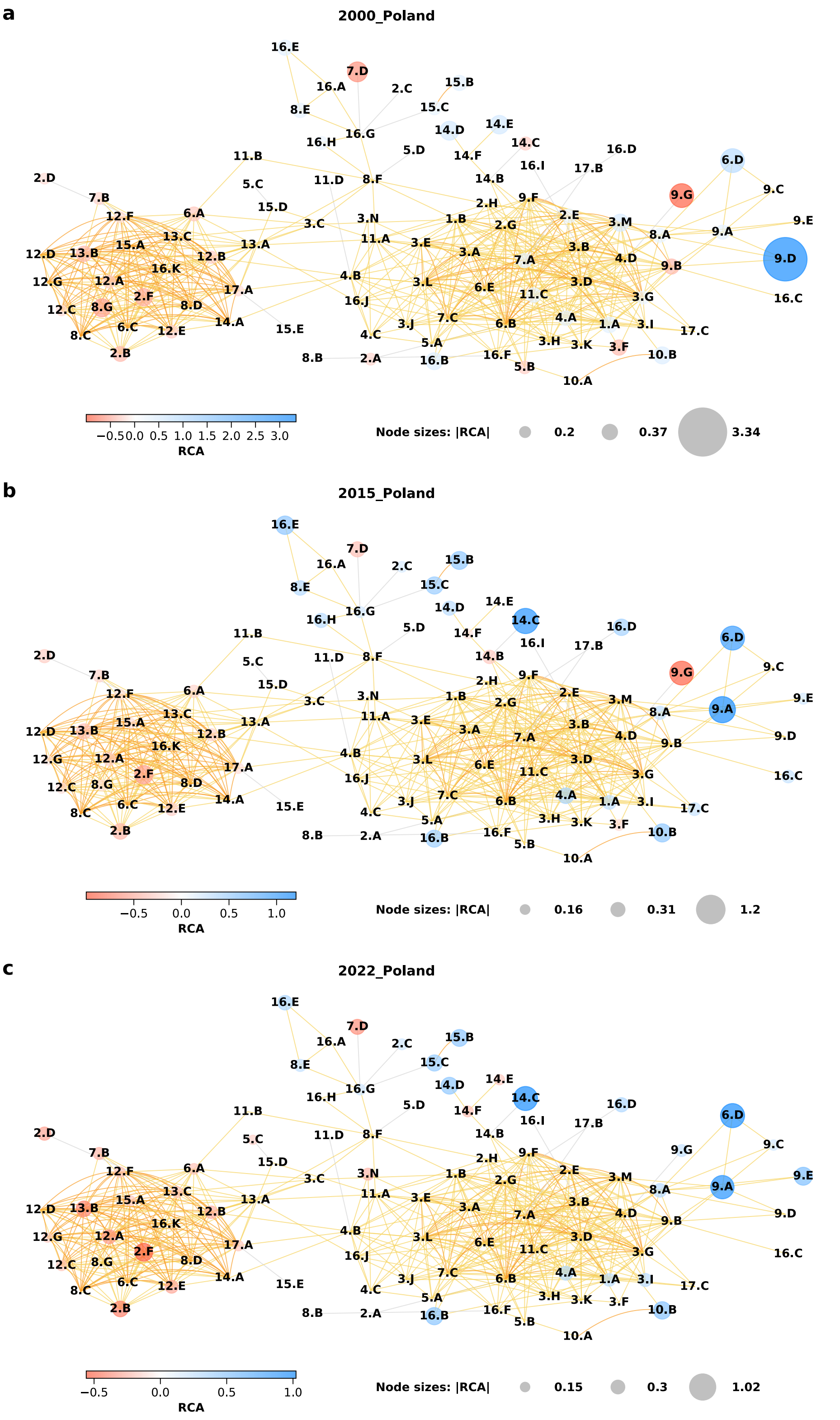

Supplementary Figure 55 | The SDG space of Poland. Panels **a, b, c,** The SDG space in 2000, 2015, and 2022. The nodes in blue and orange represent the top 20 and bottom 20 SDG indicators in revealed comparative advantage (RCA) values, respectively. The node size represents the absolute value of RCA. From Supplementary Figure 12 to 177, countries are ranked by GDP/capita (current US$, 2022).

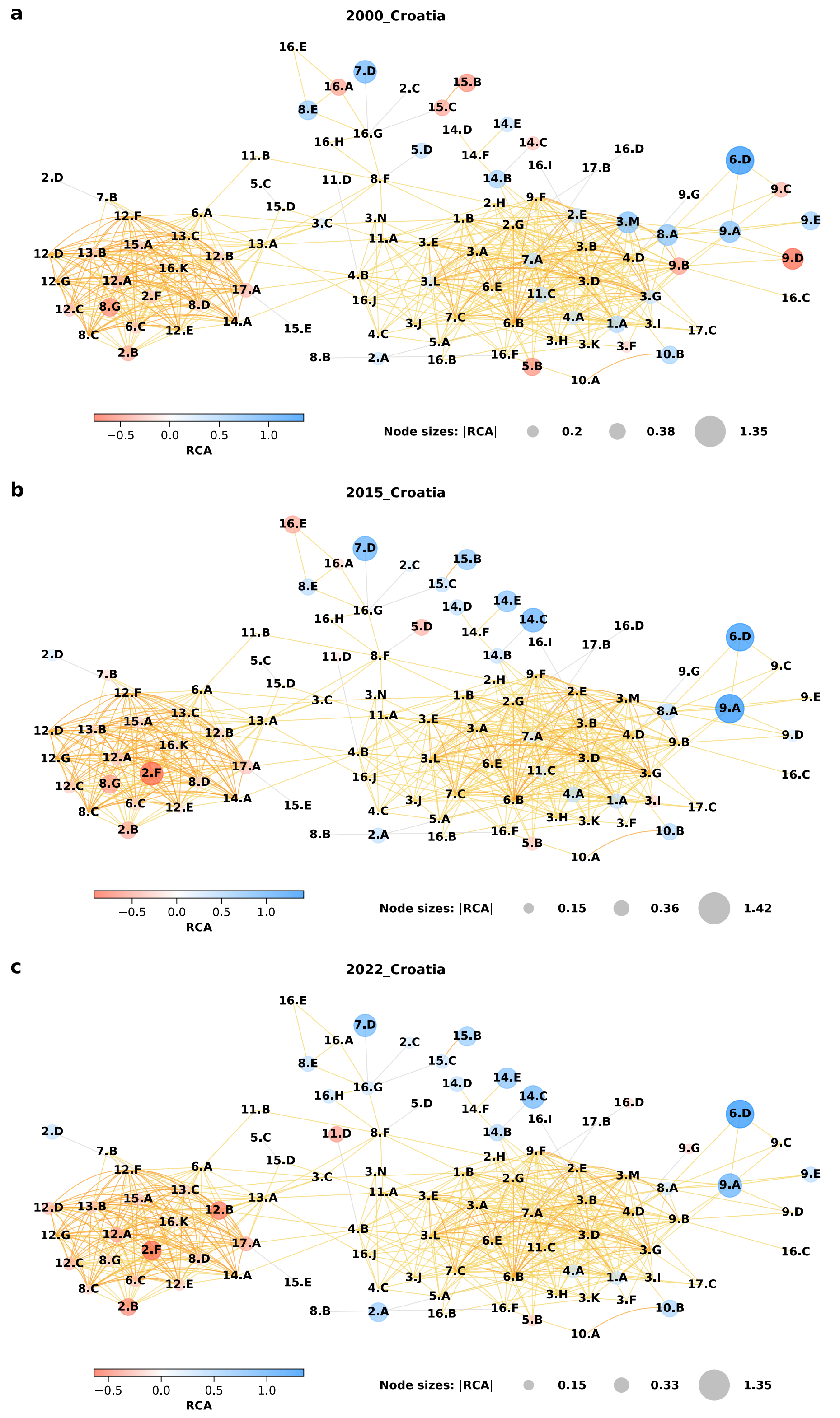

Supplementary Figure 56 | The SDG space of Croatia. Panels **a, b, c,** The SDG space in 2000, 2015, and 2022. The nodes in blue and orange represent the top 20 and bottom 20 SDG indicators in revealed comparative advantage (RCA) values, respectively. The node size represents the absolute value of RCA. From Supplementary Figure 12 to 177, countries are ranked by GDP/capita (current US$, 2022).

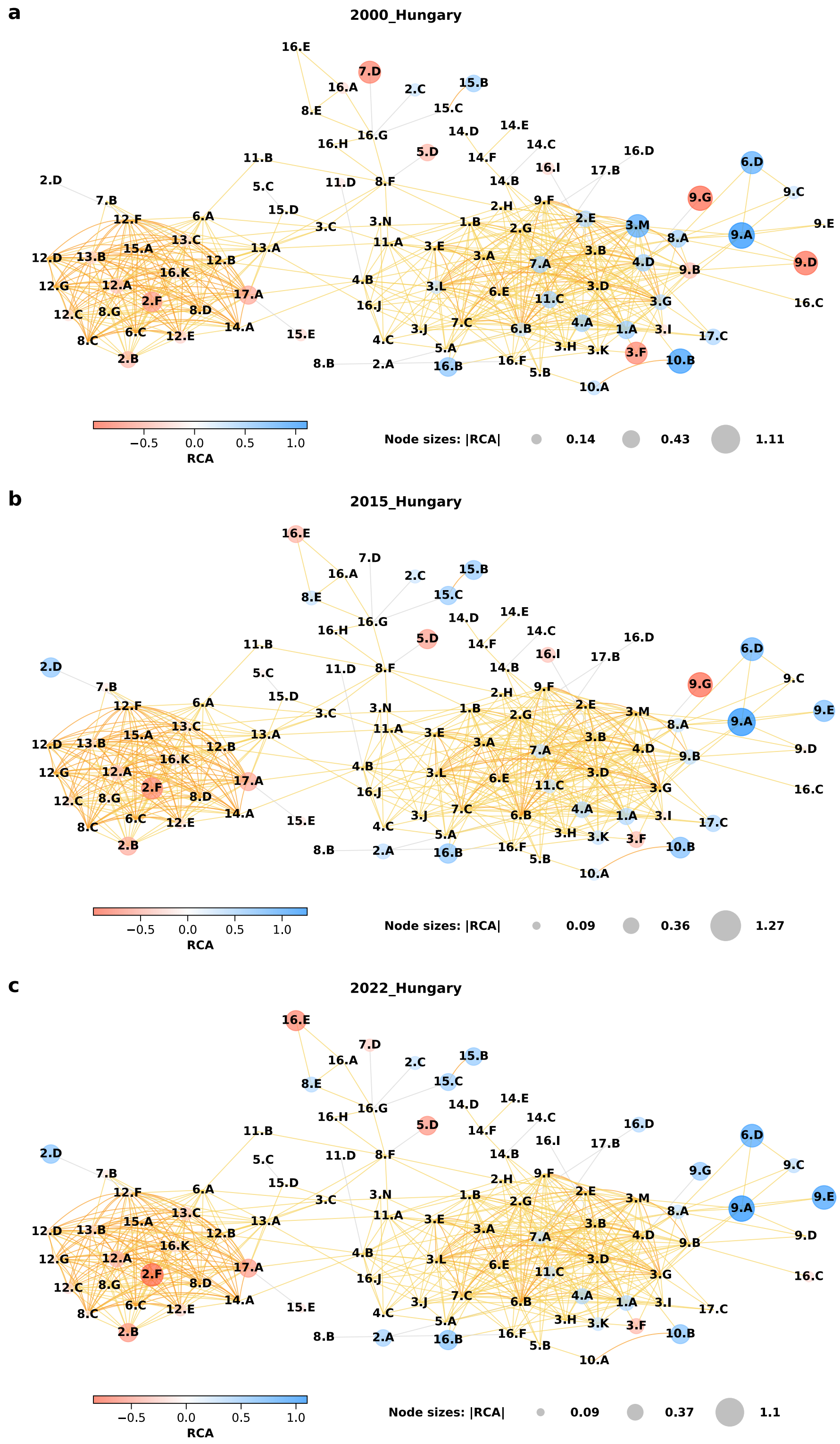

Supplementary Figure 57 | The SDG space of Hungary. Panels **a, b, c,** The SDG space in 2000, 2015, and 2022. The nodes in blue and orange represent the top 20 and bottom 20 SDG indicators in revealed comparative advantage (RCA) values, respectively. The node size represents the absolute value of RCA. From Supplementary Figure 12 to 177, countries are ranked by GDP/capita (current US$, 2022).

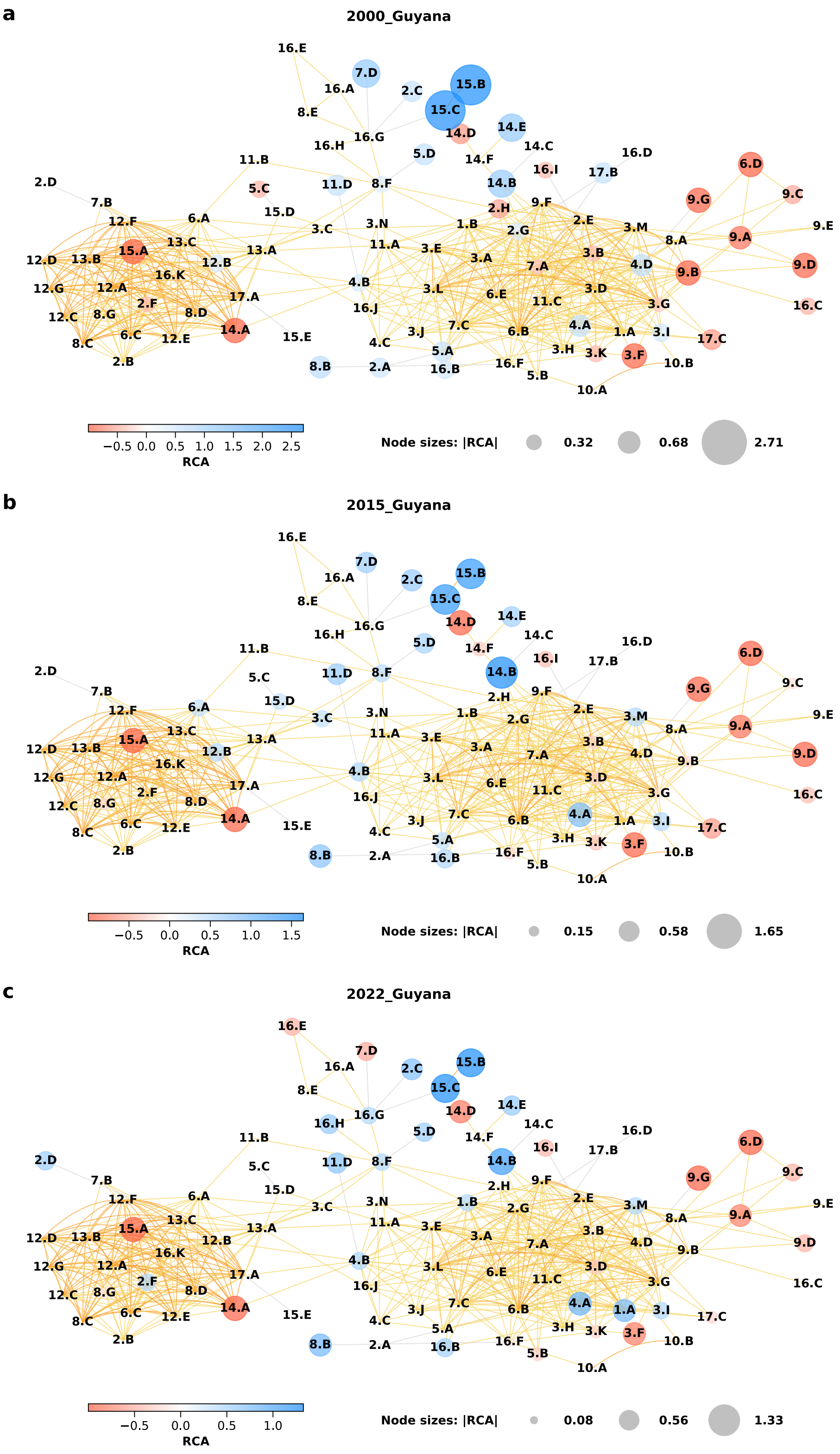

Supplementary Figure 58 | The SDG space of Guyana. Panels **a, b, c,** The SDG space in 2000, 2015, and 2022. The nodes in blue and orange represent the top 20 and bottom 20 SDG indicators in revealed comparative advantage (RCA) values, respectively. The node size represents the absolute value of RCA. From Supplementary Figure 12 to 177, countries are ranked by GDP/capita (current US$, 2022).

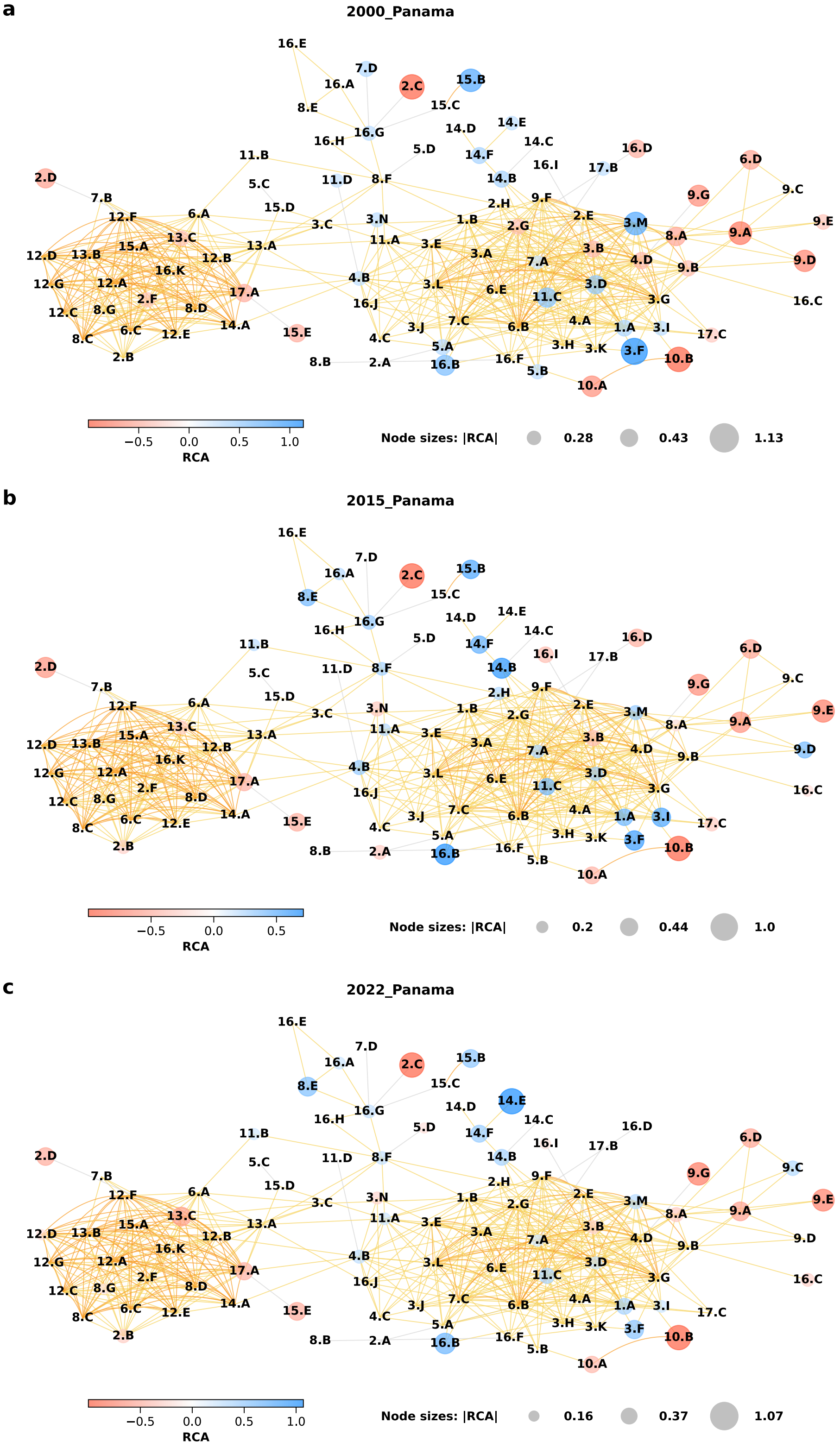

Supplementary Figure 59 | The SDG space of Panama. Panels **a, b, c,** The SDG space in 2000, 2015, and 2022. The nodes in blue and orange represent the top 20 and bottom 20 SDG indicators in revealed comparative advantage (RCA) values, respectively. The node size represents the absolute value of RCA. From Supplementary Figure 12 to 177, countries are ranked by GDP/capita (current US$, 2022).

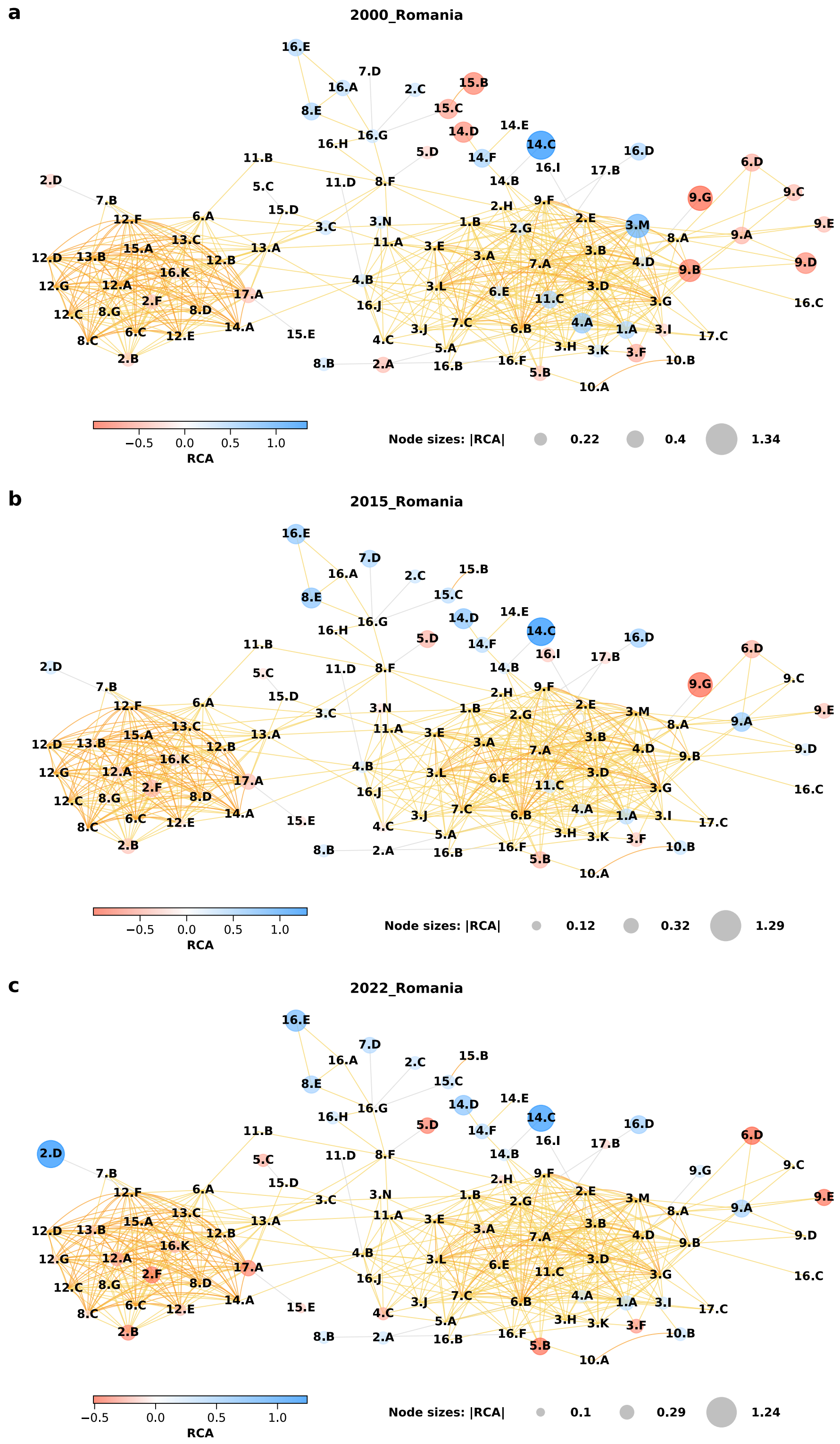

Supplementary Figure 60 | The SDG space of Romania. Panels **a, b, c,** The SDG space in 2000, 2015, and 2022. The nodes in blue and orange represent the top 20 and bottom 20 SDG indicators in revealed comparative advantage (RCA) values, respectively. The node size represents the absolute value of RCA. From Supplementary Figure 12 to 177, countries are ranked by GDP/capita (current US$, 2022).

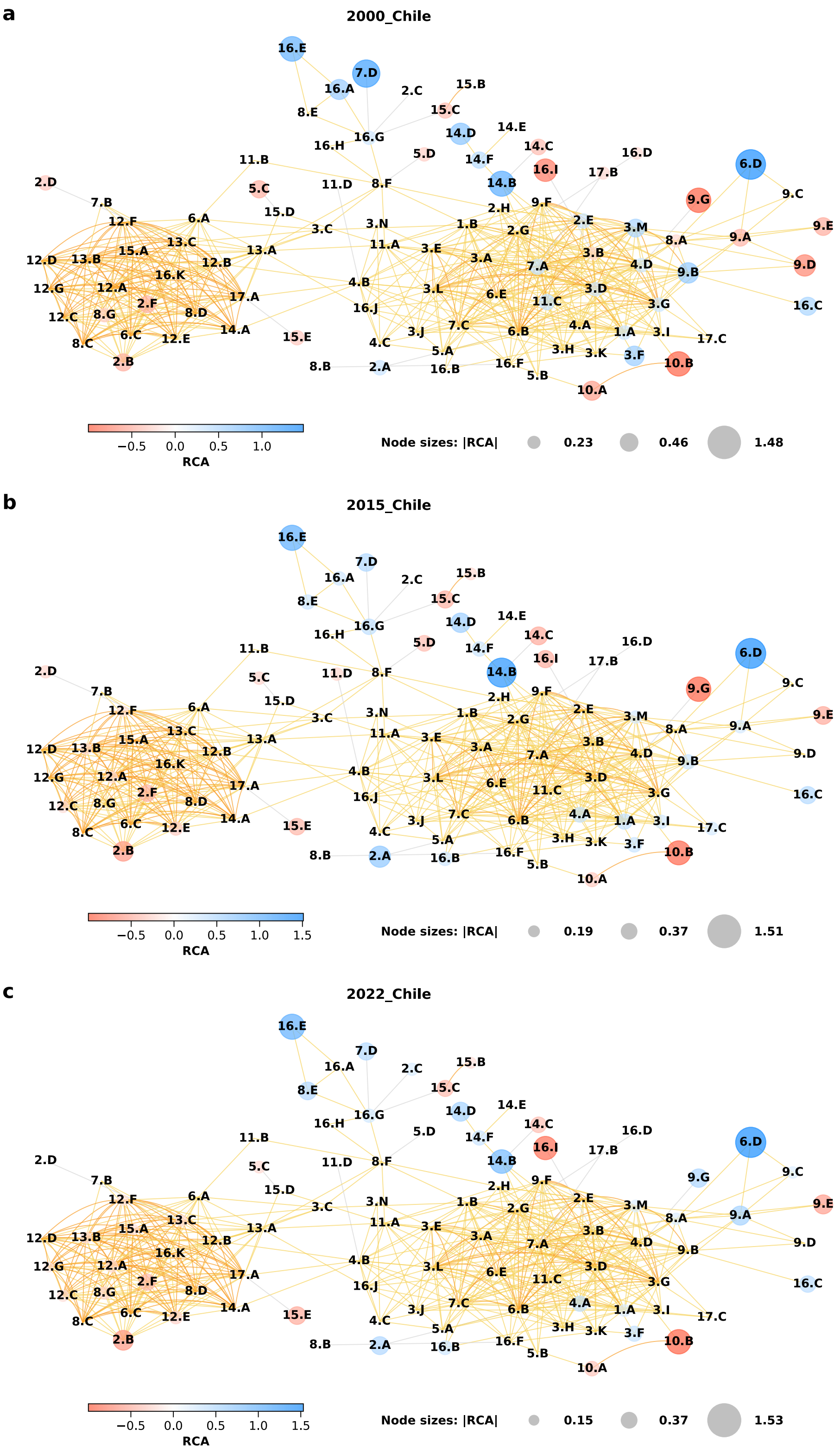

Supplementary Figure 61 | The SDG space of Chile. Panels **a, b, c,** The SDG space in 2000, 2015, and 2022. The nodes in blue and orange represent the top 20 and bottom 20 SDG indicators in revealed comparative advantage (RCA) values, respectively. The node size represents the absolute value of RCA. From Supplementary Figure 12 to 177, countries are ranked by GDP/capita (current US$, 2022).

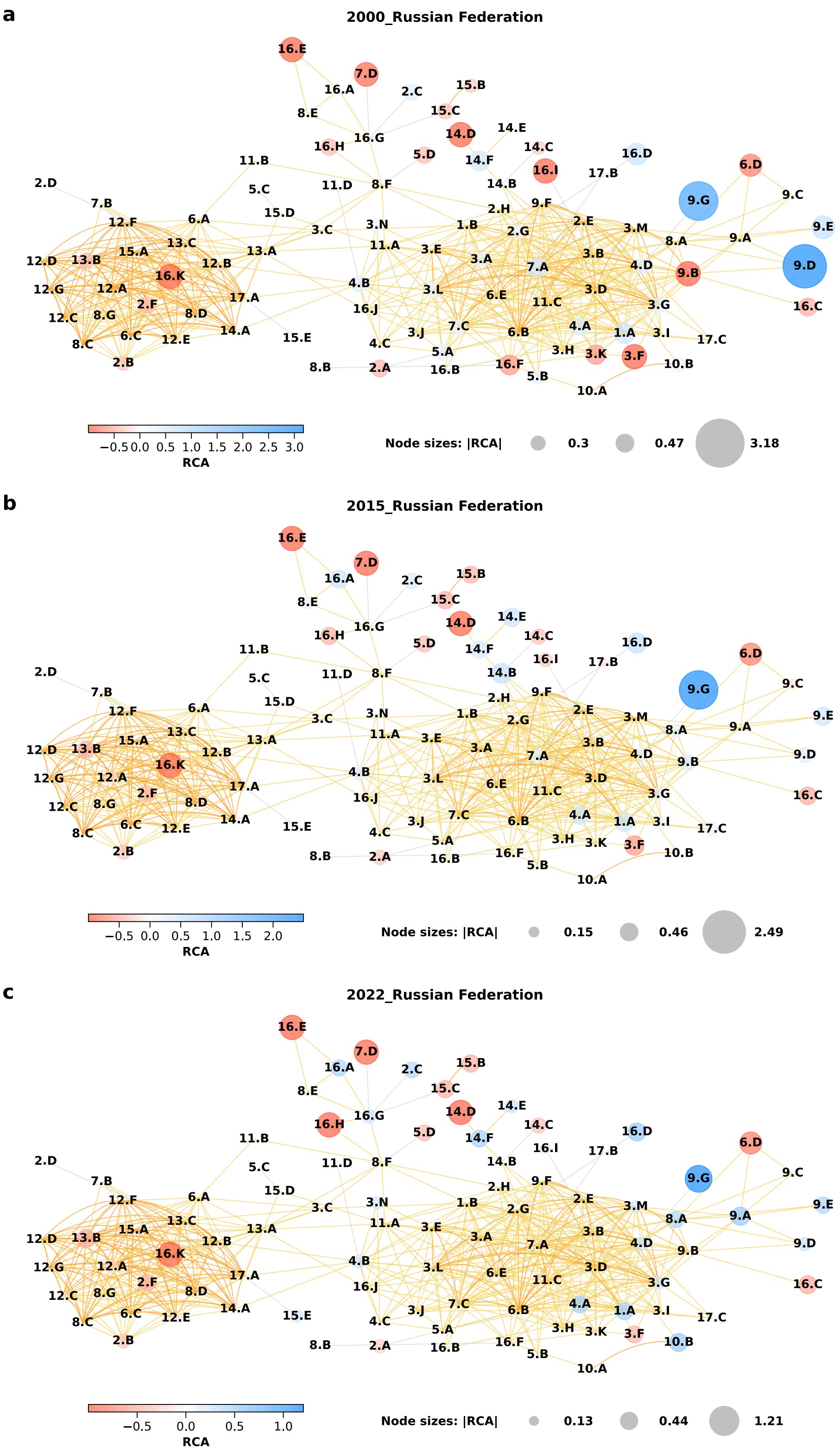

Supplementary Figure 62 | The SDG space of Russian Federation. Panels **a, b, c,** The SDG space in 2000, 2015, and 2022. The nodes in blue and orange represent the top 20 and bottom 20 SDG indicators in revealed comparative advantage (RCA) values, respectively. The node size represents the absolute value of RCA. From Supplementary Figure 12 to 177, countries are ranked by GDP/capita (current US$, 2022).

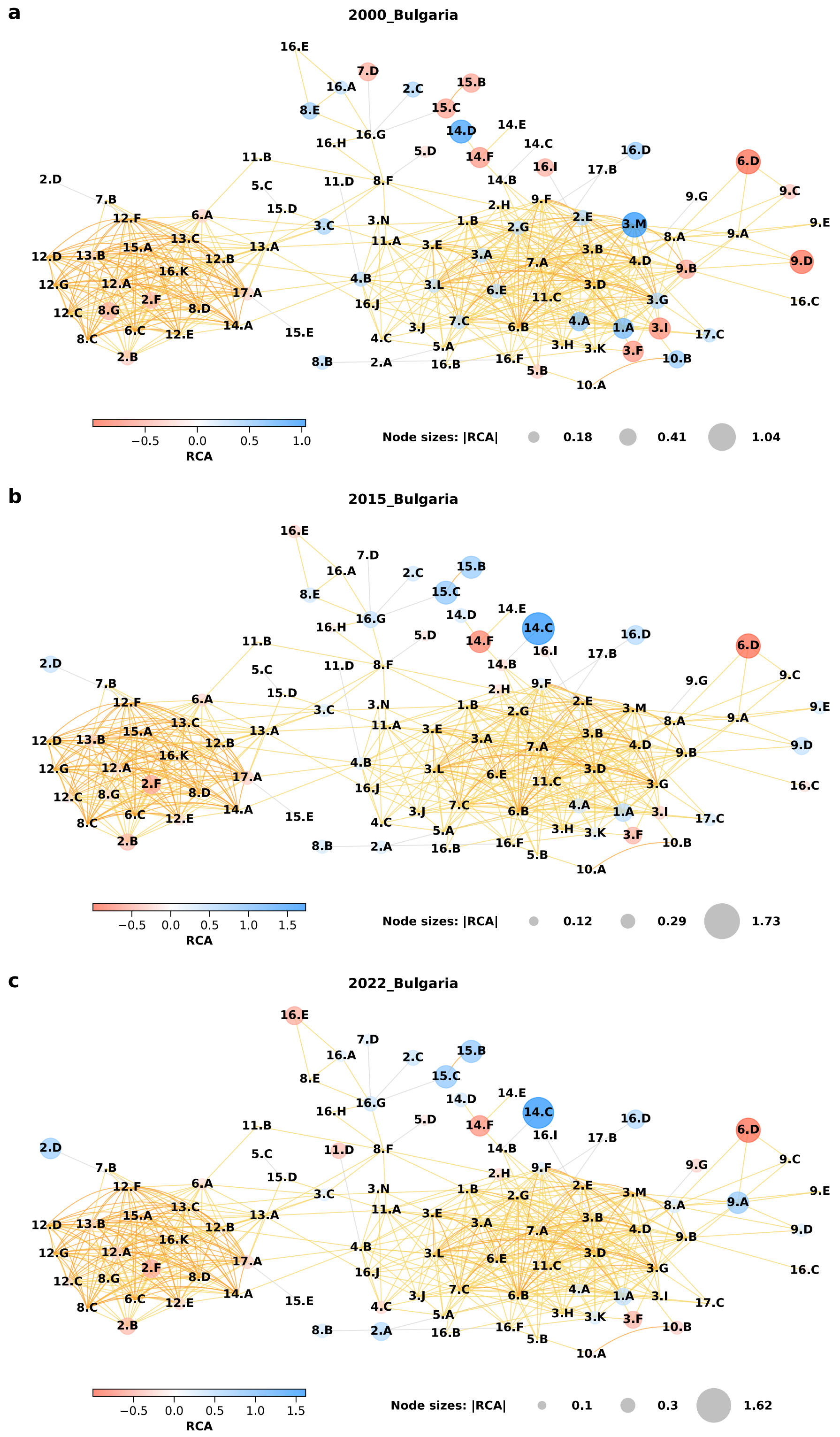

Supplementary Figure 63 | The SDG space of Bulgaria. Panels **a, b, c,** The SDG space in 2000, 2015, and 2022. The nodes in blue and orange represent the top 20 and bottom 20 SDG indicators in revealed comparative advantage (RCA) values, respectively. The node size represents the absolute value of RCA. From Supplementary Figure 12 to 177, countries are ranked by GDP/capita (current US$, 2022).

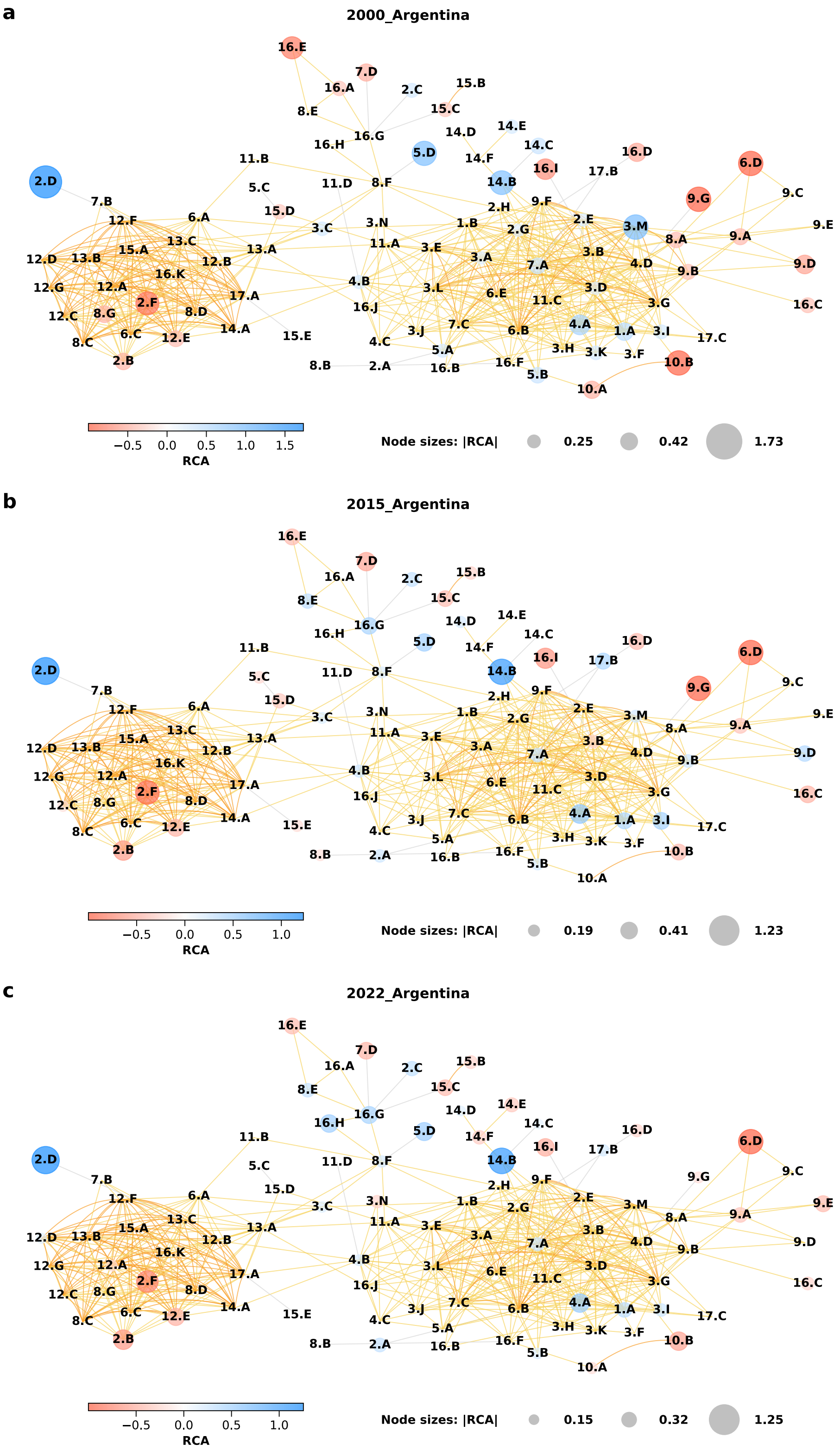

Supplementary Figure 64 | The SDG space of Argentina. Panels **a, b, c,** The SDG space in 2000, 2015, and 2022. The nodes in blue and orange represent the top 20 and bottom 20 SDG indicators in revealed comparative advantage (RCA) values, respectively. The node size represents the absolute value of RCA. From Supplementary Figure 12 to 177, countries are ranked by GDP/capita (current US$, 2022).

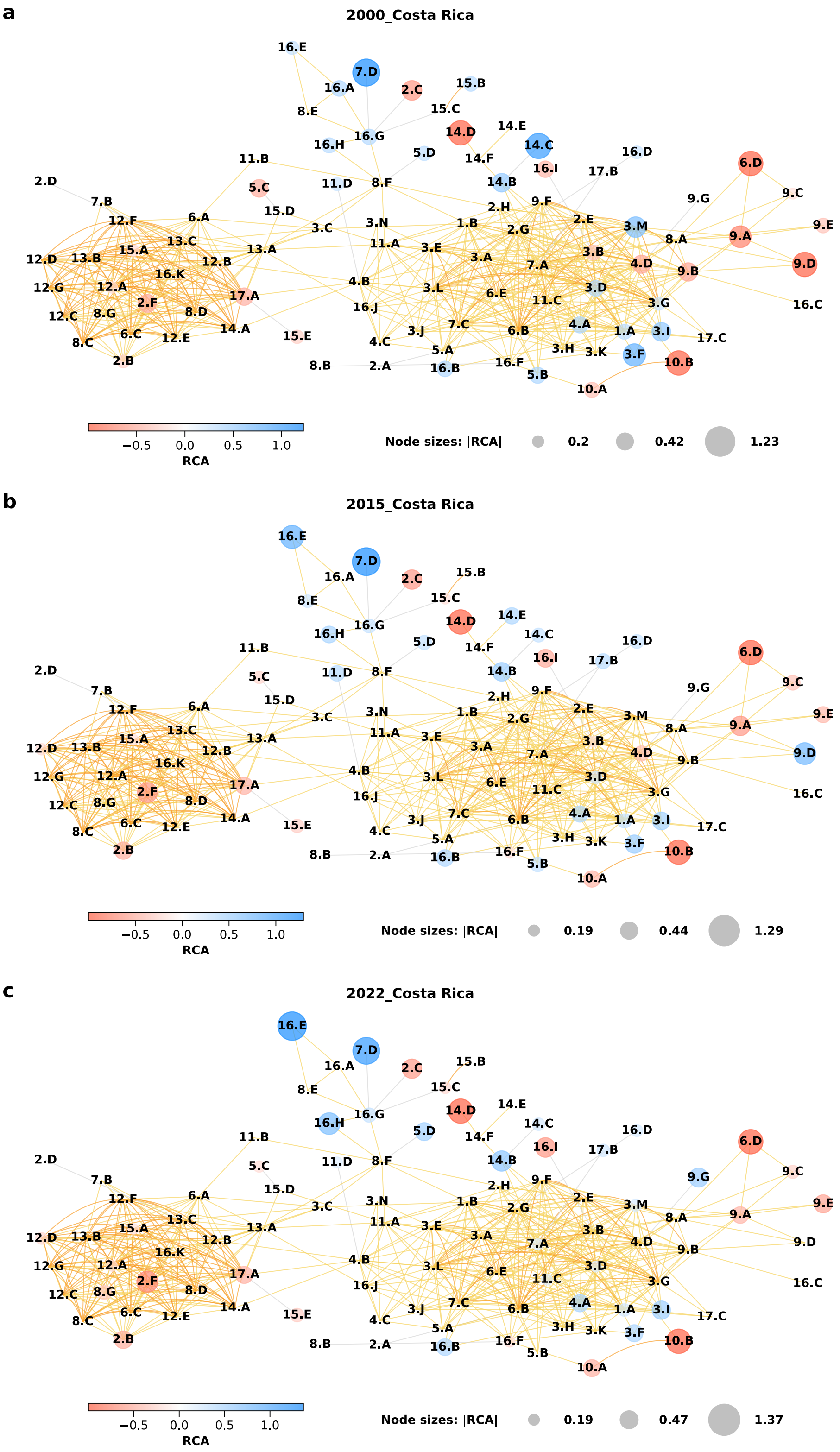

Supplementary Figure 65 | The SDG space of Costa Rica. Panels **a, b, c,** The SDG space in 2000, 2015, and 2022. The nodes in blue and orange represent the top 20 and bottom 20 SDG indicators in revealed comparative advantage (RCA) values, respectively. The node size represents the absolute value of RCA. From Supplementary Figure 12 to 177, countries are ranked by GDP/capita (current US$, 2022).

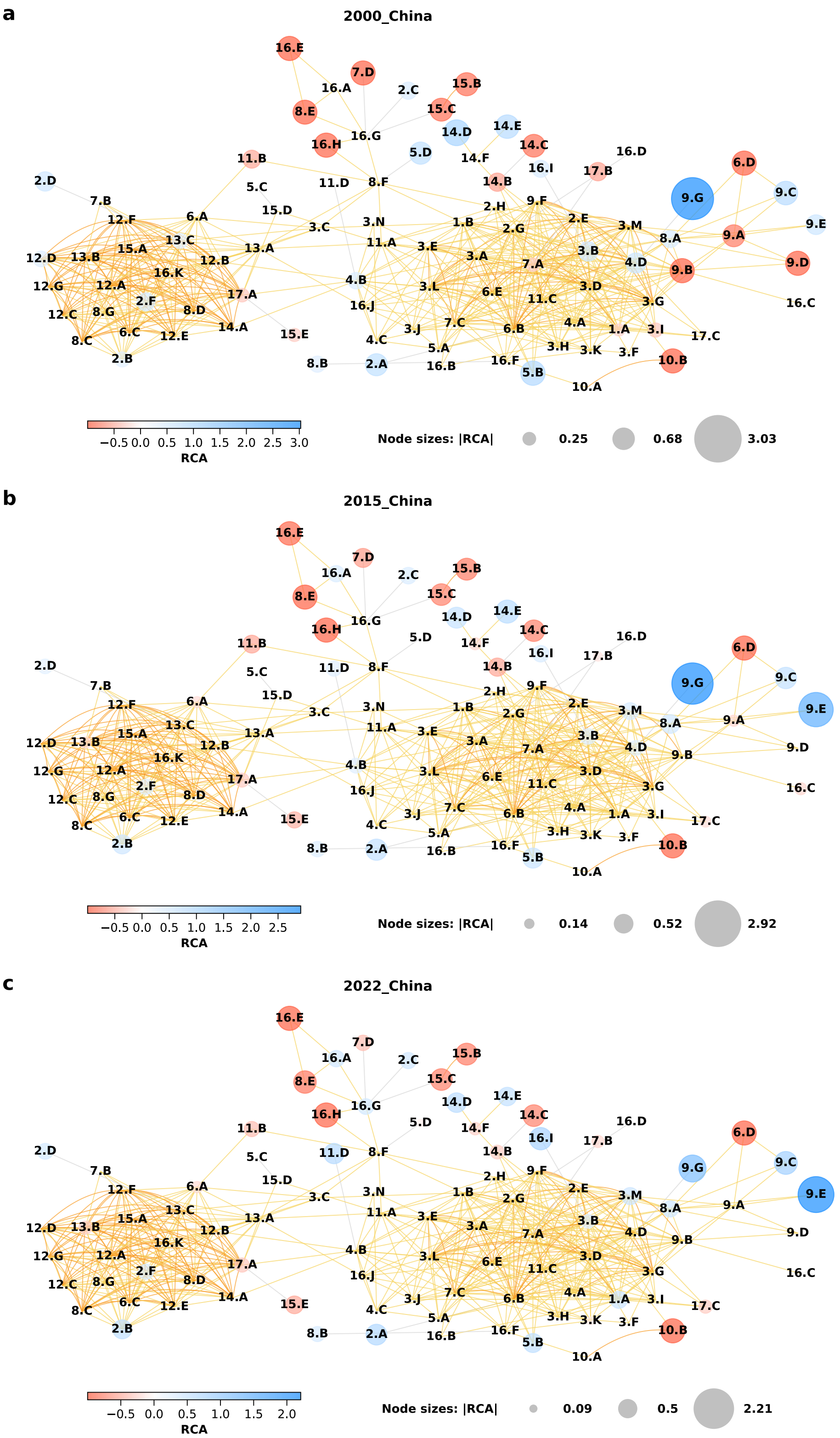

Supplementary Figure 66 | The SDG space of China. Panels **a, b, c,** The SDG space in 2000, 2015, and 2022. The nodes in blue and orange represent the top 20 and bottom 20 SDG indicators in revealed comparative advantage (RCA) values, respectively. The node size represents the absolute value of RCA. From Supplementary Figure 12 to 177, countries are ranked by GDP/capita (current US$, 2022).

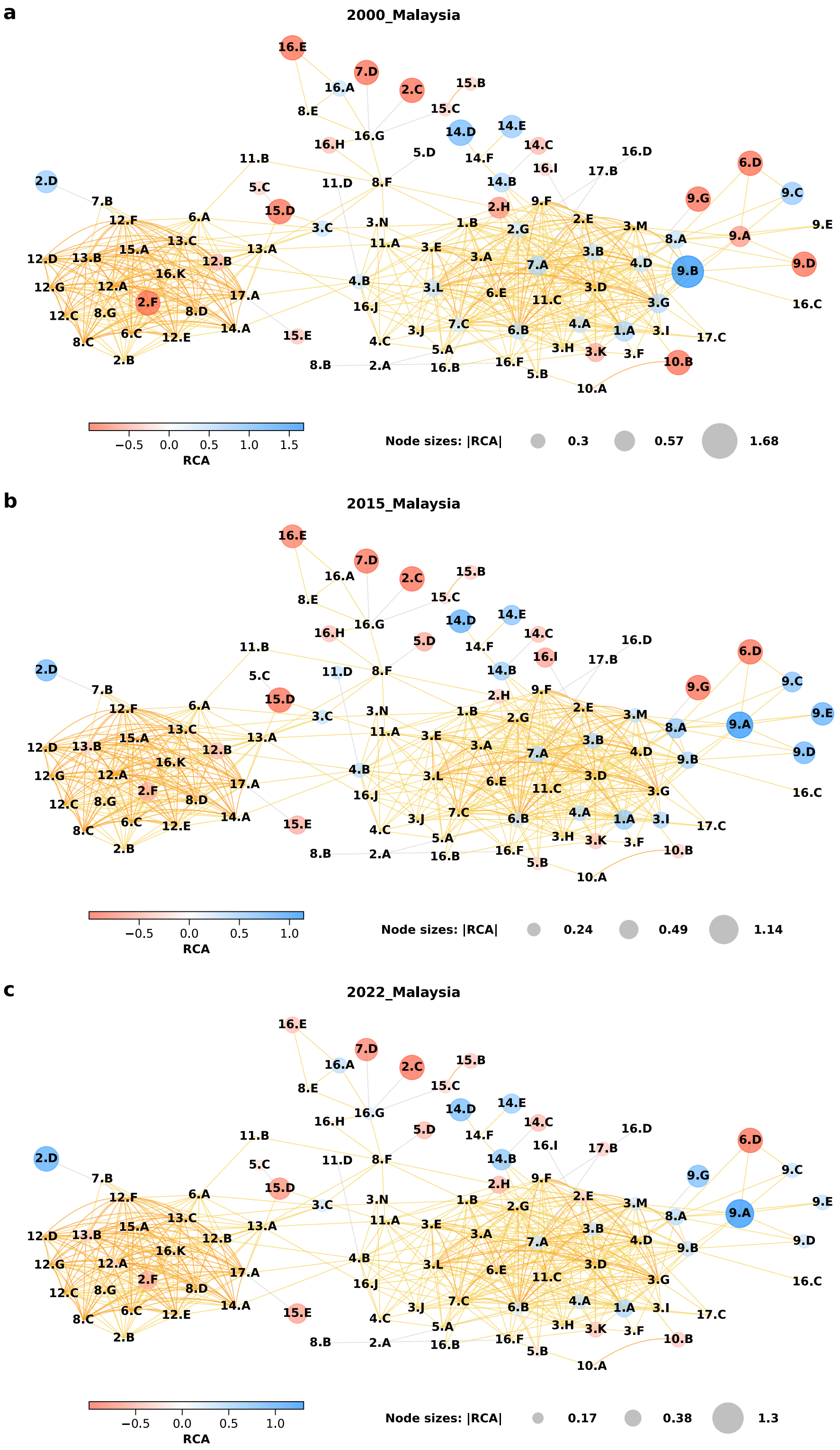

Supplementary Figure 67 | The SDG space of Malaysia. Panels **a, b, c,** The SDG space in 2000, 2015, and 2022. The nodes in blue and orange represent the top 20 and bottom 20 SDG indicators in revealed comparative advantage (RCA) values, respectively. The node size represents the absolute value of RCA. From Supplementary Figure 12 to 177, countries are ranked by GDP/capita (current US$, 2022).

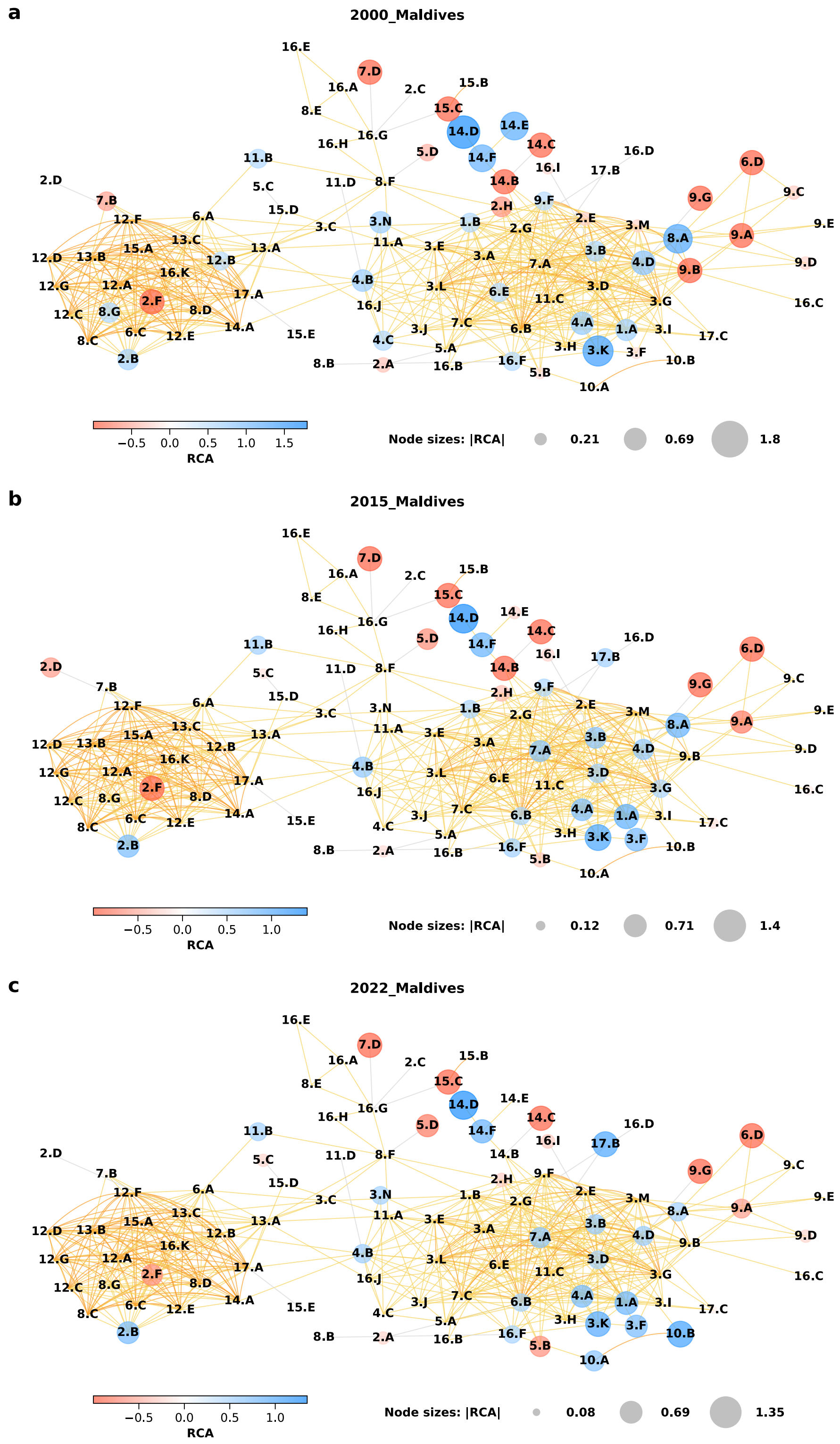

Supplementary Figure 68 | The SDG space of Maldives. Panels **a, b, c,** The SDG space in 2000, 2015, and 2022. The nodes in blue and orange represent the top 20 and bottom 20 SDG indicators in revealed comparative advantage (RCA) values, respectively. The node size represents the absolute value of RCA. From Supplementary Figure 12 to 177, countries are ranked by GDP/capita (current US$, 2022).

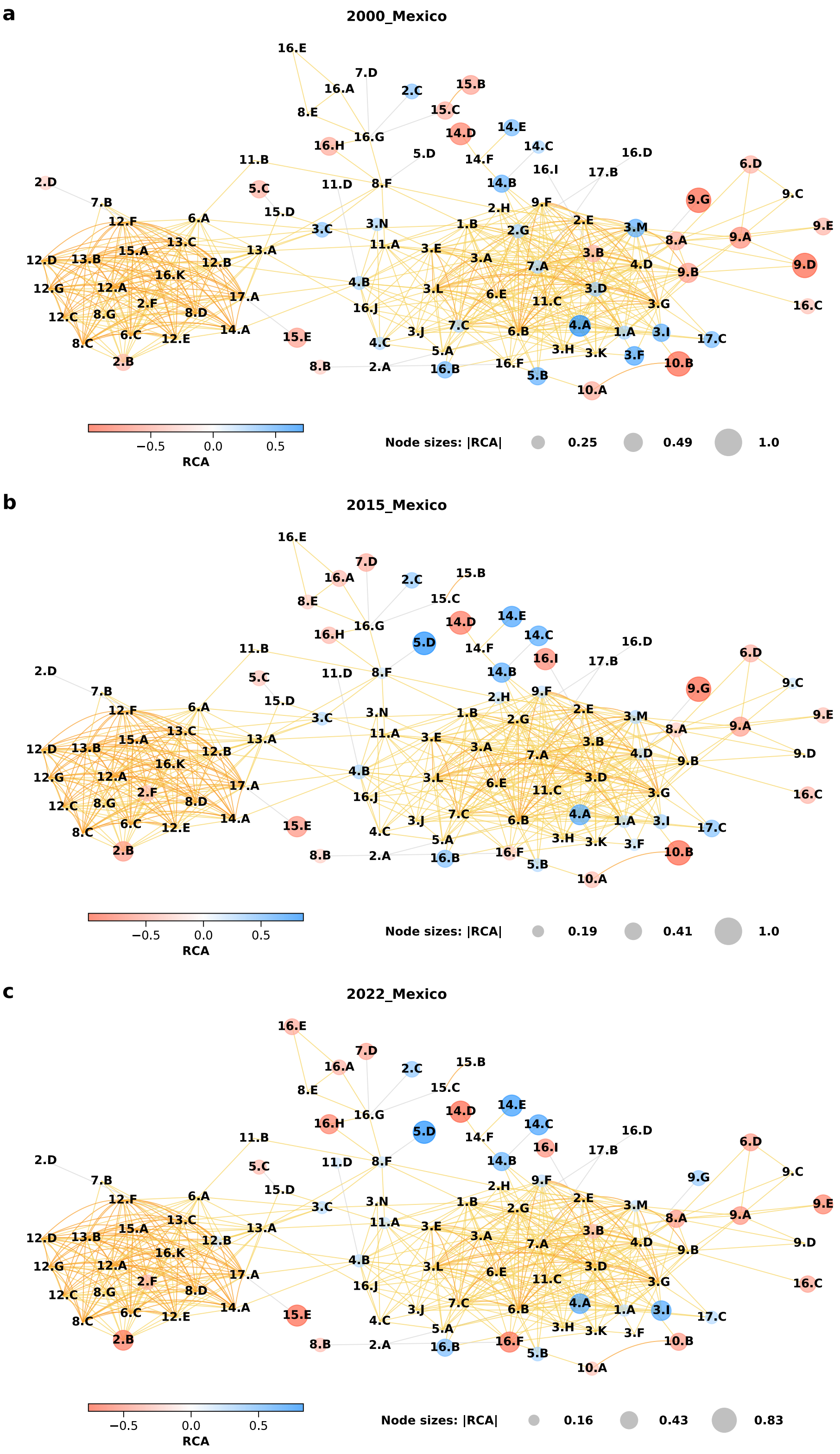

Supplementary Figure 69 | The SDG space of Mexico. Panels **a, b, c,** The SDG space in 2000, 2015, and 2022. The nodes in blue and orange represent the top 20 and bottom 20 SDG indicators in revealed comparative advantage (RCA) values, respectively. The node size represents the absolute value of RCA. From Supplementary Figure 12 to 177, countries are ranked by GDP/capita (current US$, 2022).

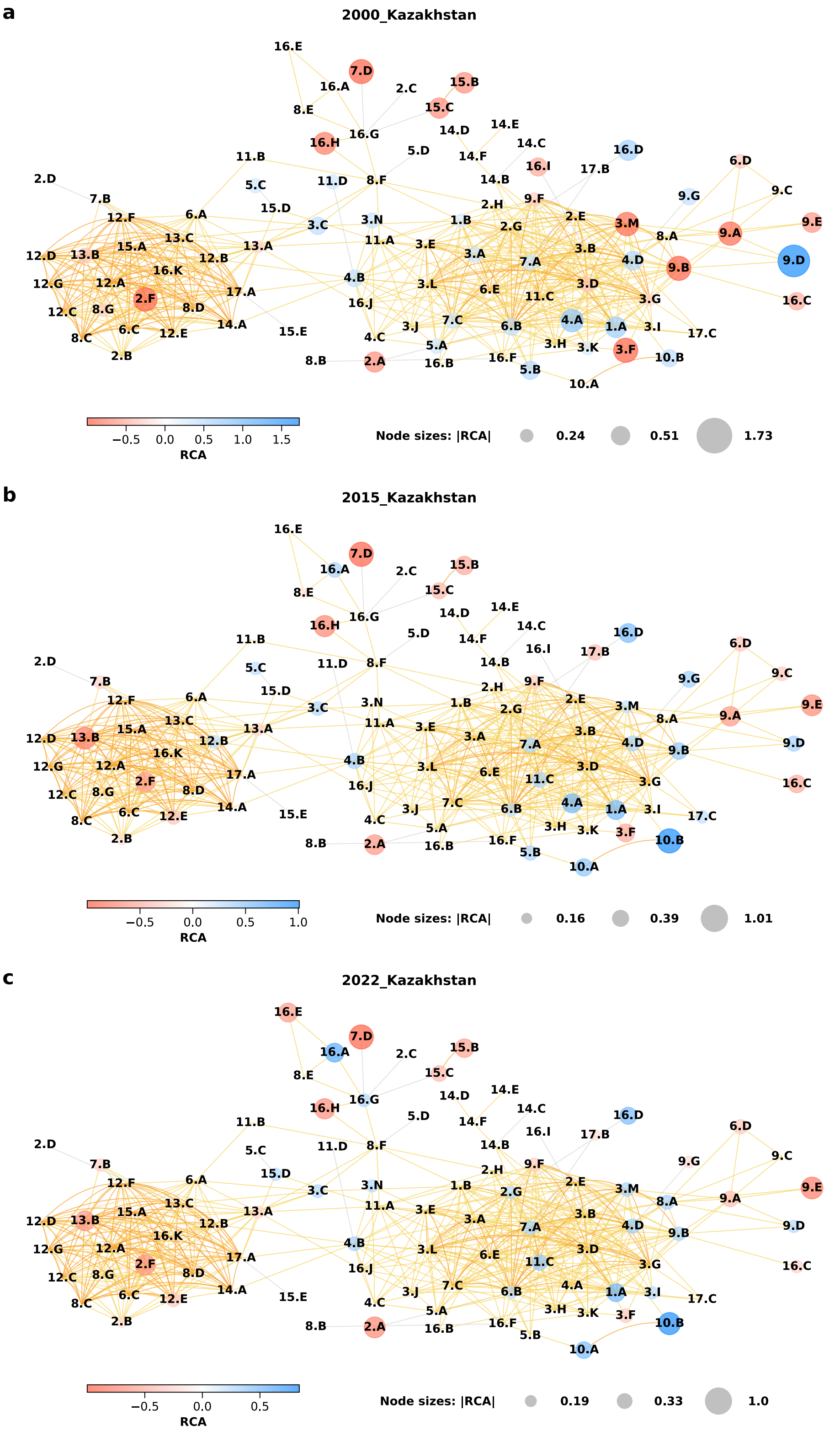

Supplementary Figure 70 | The SDG space of Kazakhstan. Panels **a, b, c,** The SDG space in 2000, 2015, and 2022. The nodes in blue and orange represent the top 20 and bottom 20 SDG indicators in revealed comparative advantage (RCA) values, respectively. The node size represents the absolute value of RCA. From Supplementary Figure 12 to 177, countries are ranked by GDP/capita (current US$, 2022).

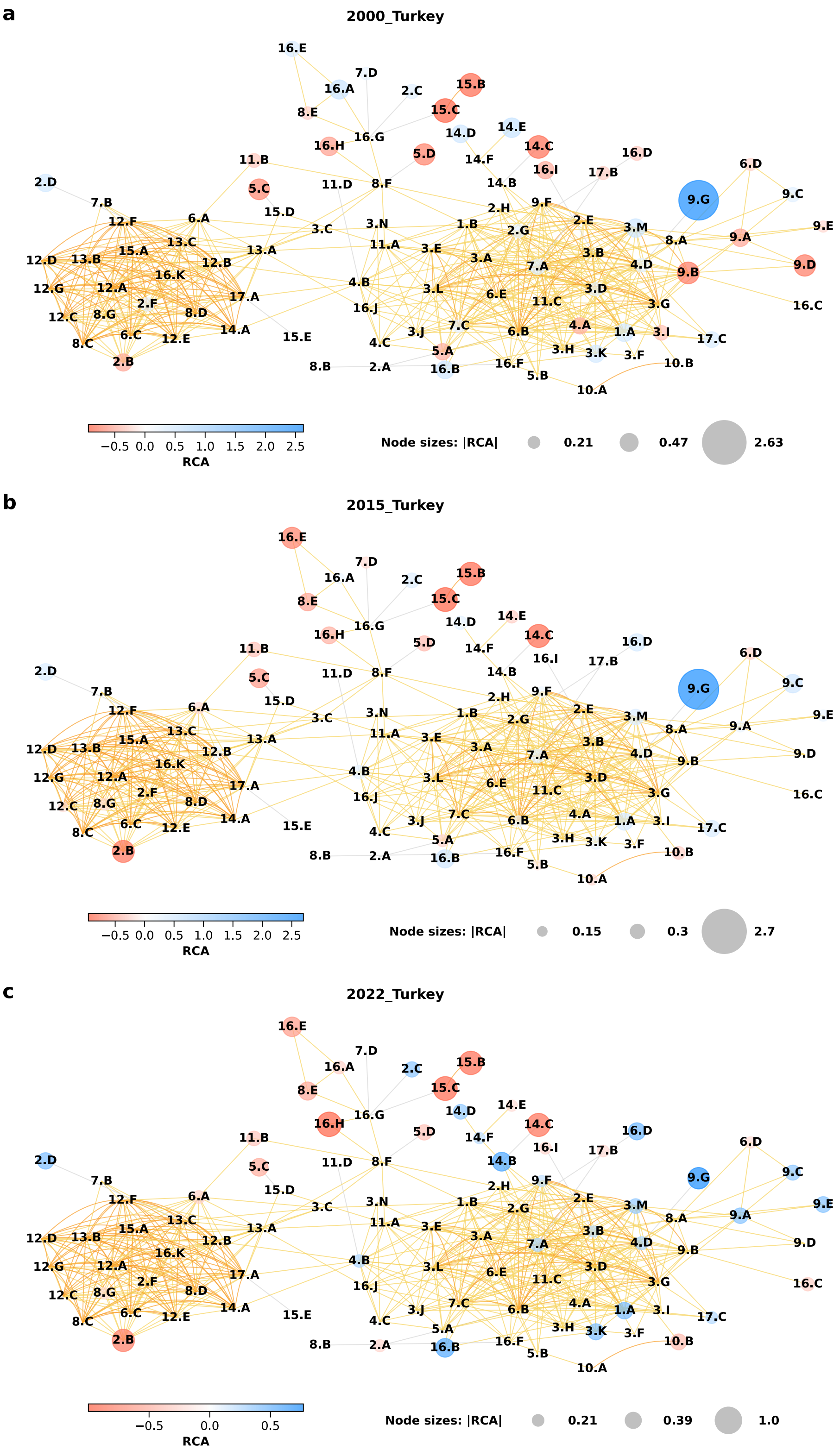

Supplementary Figure 71 | The SDG space of Turkey. Panels **a, b, c,** The SDG space in 2000, 2015, and 2022. The nodes in blue and orange represent the top 20 and bottom 20 SDG indicators in revealed comparative advantage (RCA) values, respectively. The node size represents the absolute value of RCA. From Supplementary Figure 12 to 177, countries are ranked by GDP/capita (current US$, 2022).

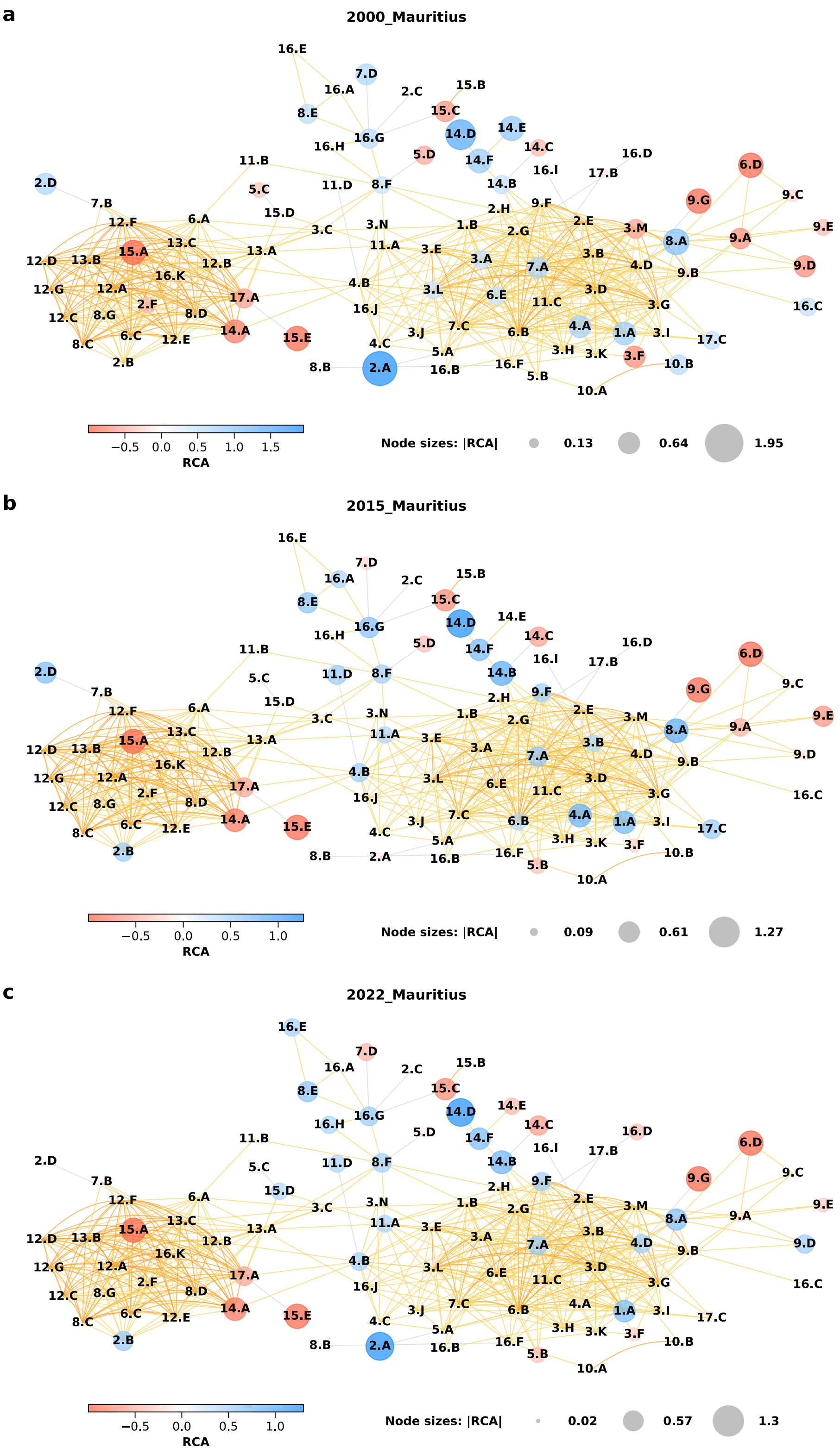

Supplementary Figure 72 | The SDG space of Mauritius. Panels **a, b, c,** The SDG space in 2000, 2015, and 2022. The nodes in blue and orange represent the top 20 and bottom 20 SDG indicators in revealed comparative advantage (RCA) values, respectively. The node size represents the absolute value of RCA. From Supplementary Figure 12 to 177, countries are ranked by GDP/capita (current US$, 2022).

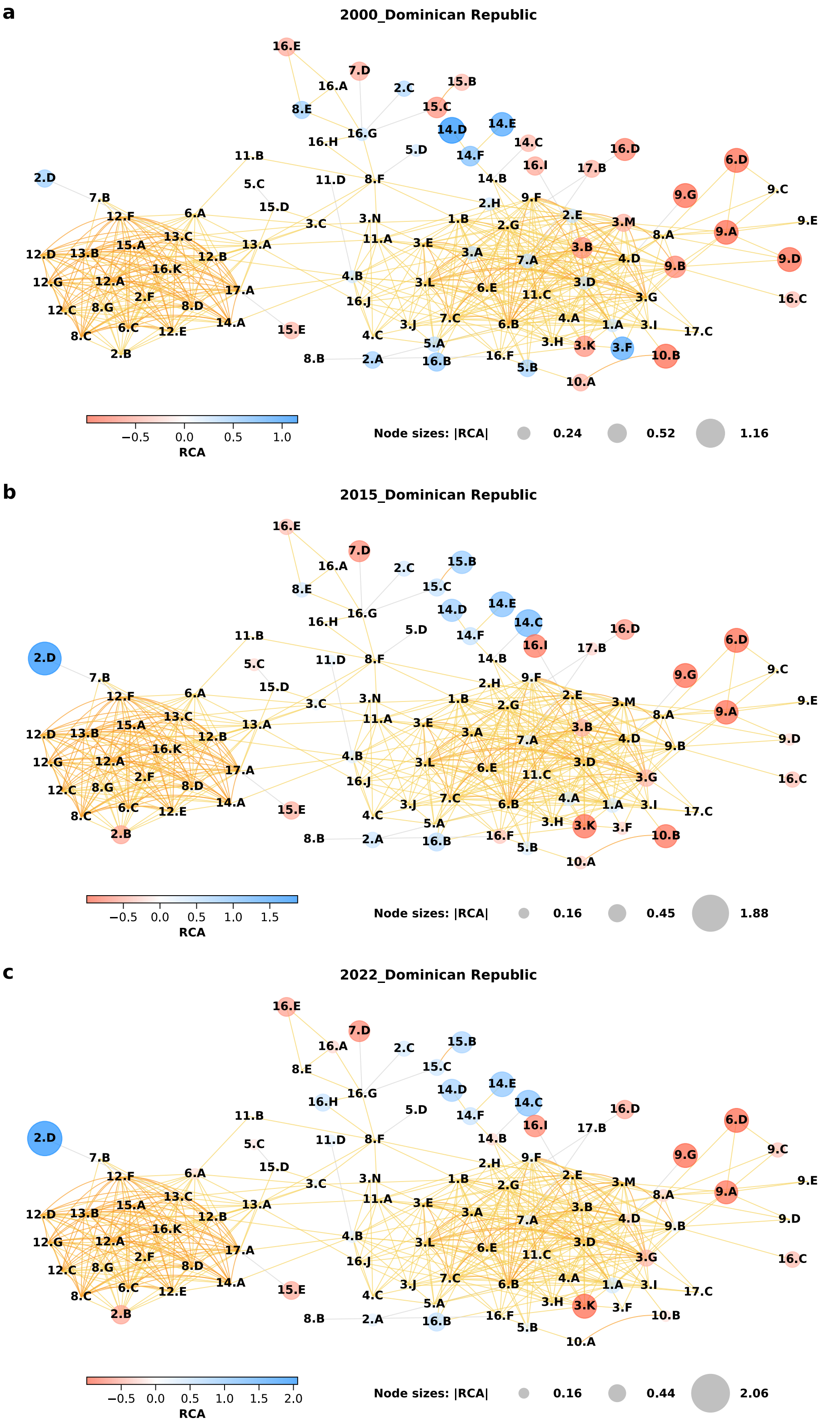

Supplementary Figure 73 | The SDG space of Dominican Republic. Panels **a, b, c,** The SDG space in 2000, 2015, and 2022. The nodes in blue and orange represent the top 20 and bottom 20 SDG indicators in revealed comparative advantage (RCA) values, respectively. The node size represents the absolute value of RCA. From Supplementary Figure 12 to 177, countries are ranked by GDP/capita (current US$, 2022).

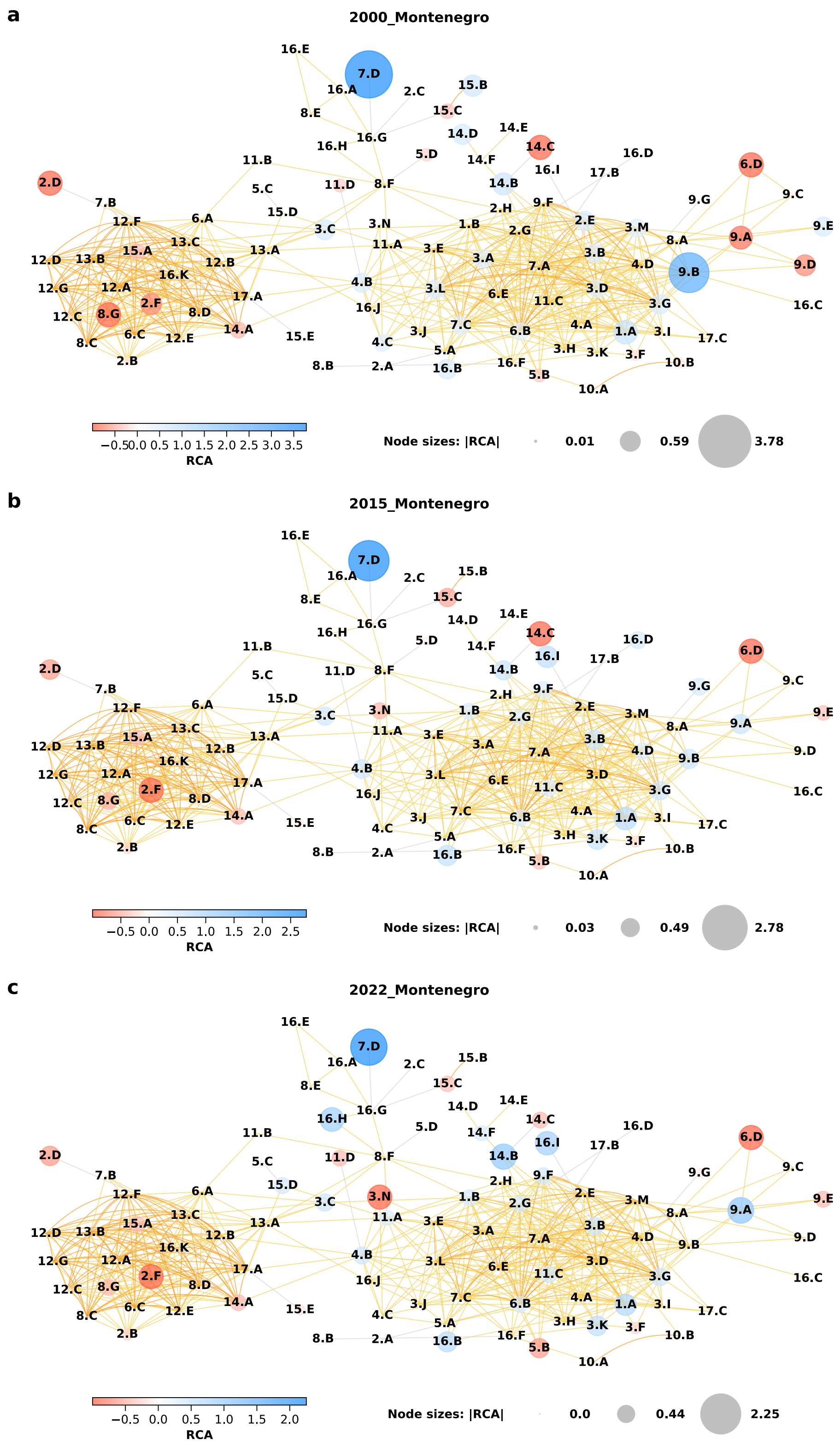

Supplementary Figure 74 | The SDG space of Montenegro. Panels **a, b, c,** The SDG space in 2000, 2015, and 2022. The nodes in blue and orange represent the top 20 and bottom 20 SDG indicators in revealed comparative advantage (RCA) values, respectively. The node size represents the absolute value of RCA. From Supplementary Figure 12 to 177, countries are ranked by GDP/capita (current US$, 2022).

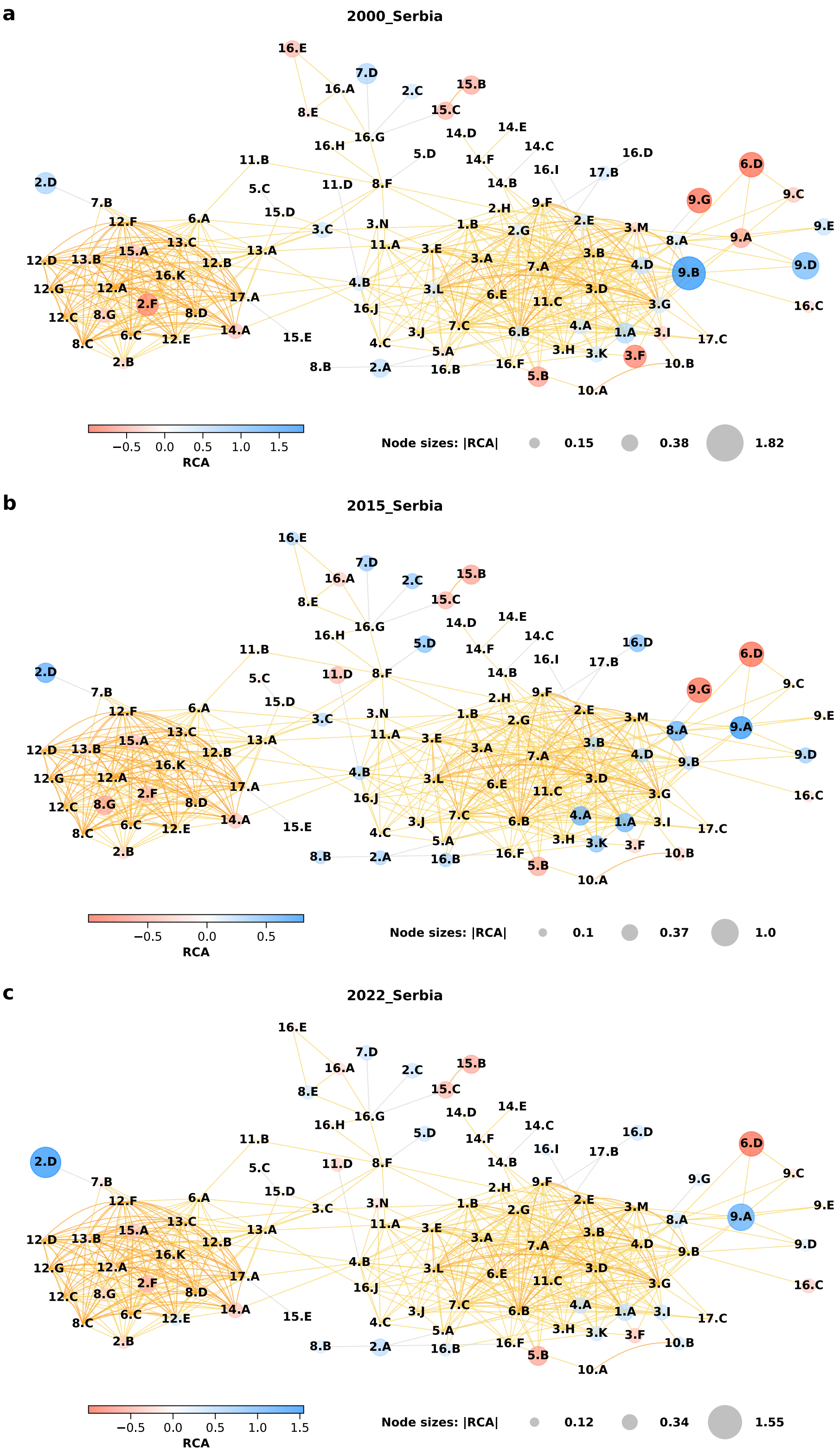

Supplementary Figure 75 | The SDG space of Serbia. Panels **a, b, c,** The SDG space in 2000, 2015, and 2022. The nodes in blue and orange represent the top 20 and bottom 20 SDG indicators in revealed comparative advantage (RCA) values, respectively. The node size represents the absolute value of RCA. From Supplementary Figure 12 to 177, countries are ranked by GDP/capita (current US$, 2022).

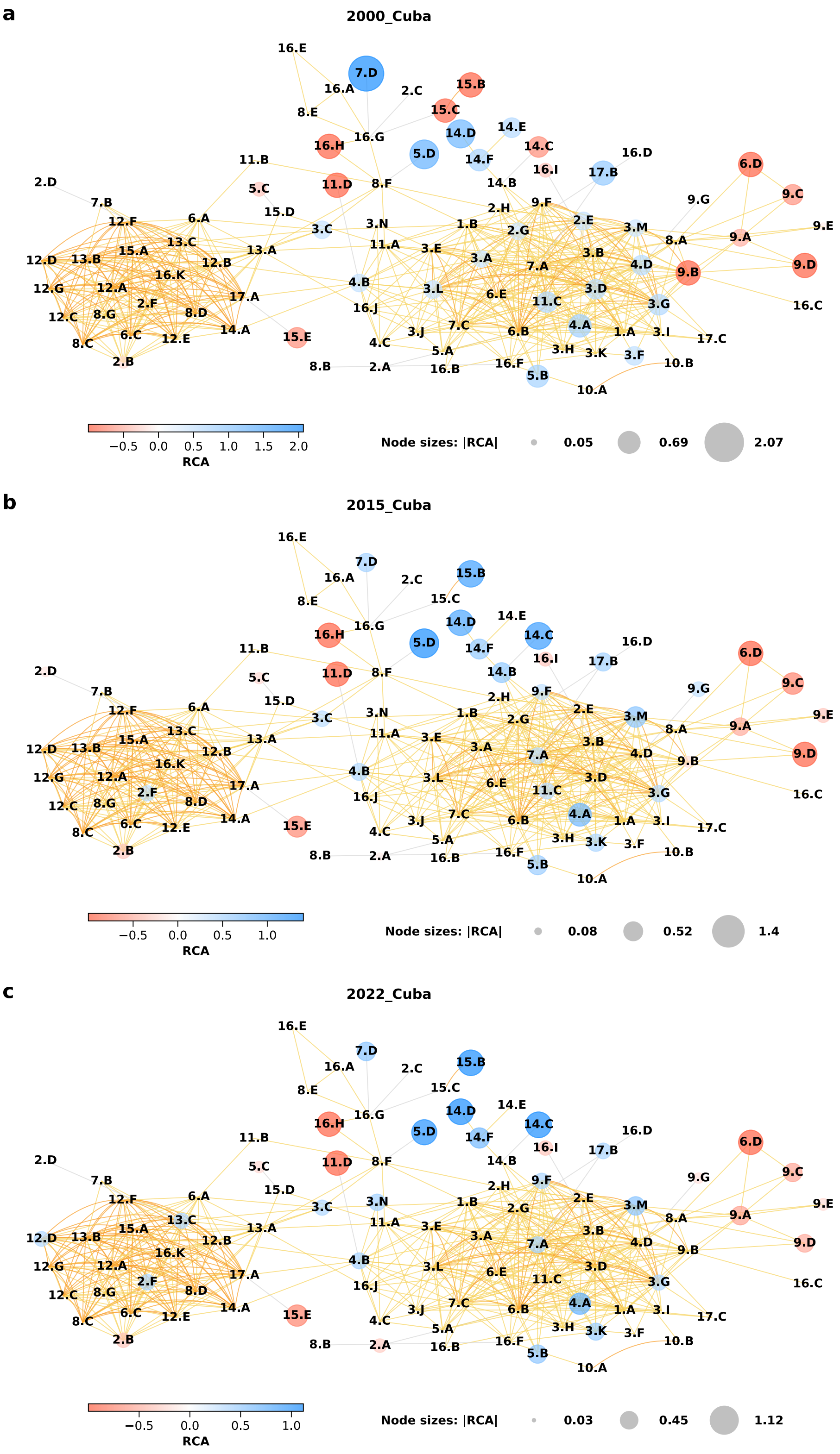

Supplementary Figure 76 | The SDG space of Cuba. Panels **a, b, c,** The SDG space in 2000, 2015, and 2022. The nodes in blue and orange represent the top 20 and bottom 20 SDG indicators in revealed comparative advantage (RCA) values, respectively. The node size represents the absolute value of RCA. From Supplementary Figure 12 to 177, countries are ranked by GDP/capita (current US$, 2022).

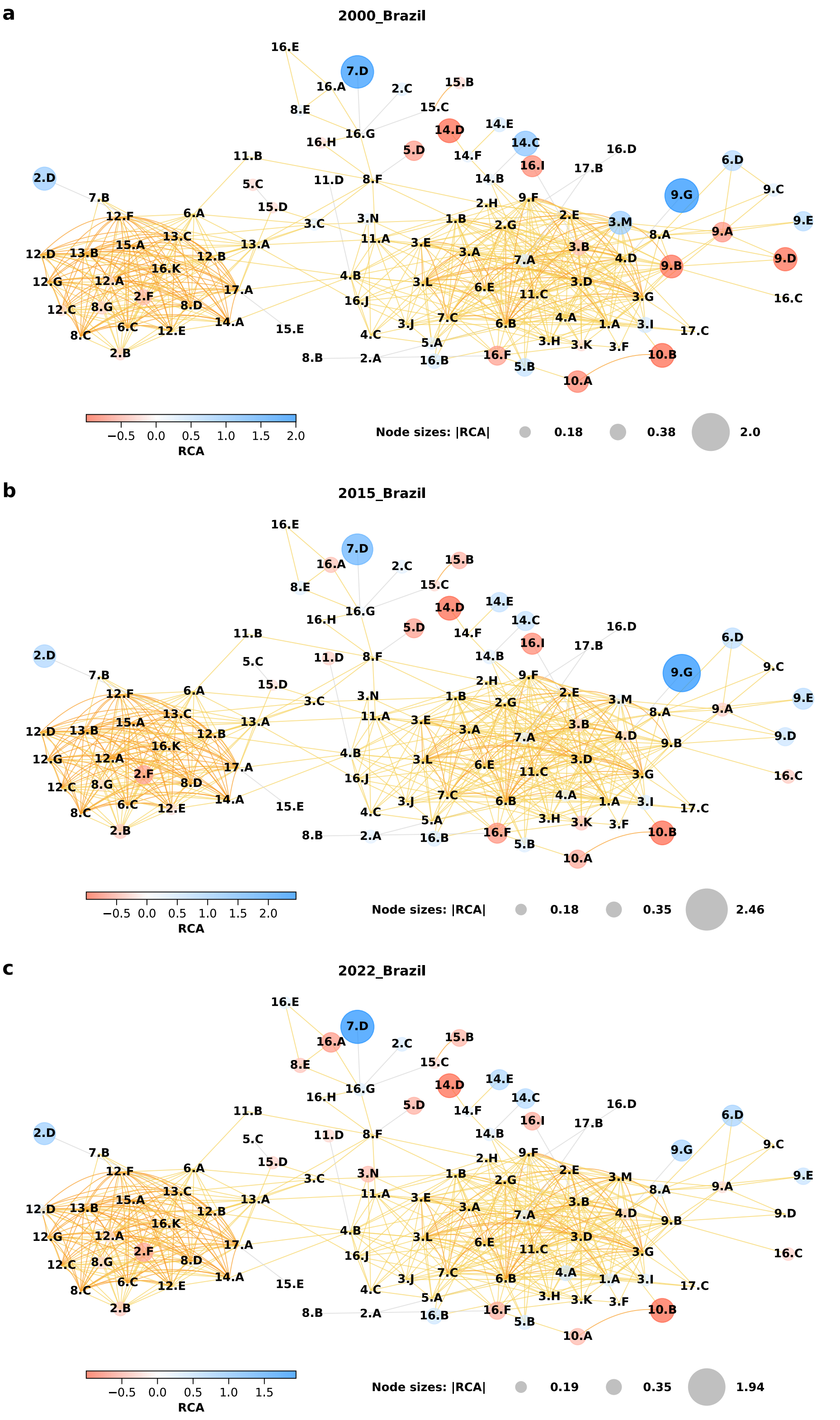

Supplementary Figure 77 | The SDG space of Brazil. Panels **a, b, c,** The SDG space in 2000, 2015, and 2022. The nodes in blue and orange represent the top 20 and bottom 20 SDG indicators in revealed comparative advantage (RCA) values, respectively. The node size represents the absolute value of RCA. From Supplementary Figure 12 to 177, countries are ranked by GDP/capita (current US$, 2022).

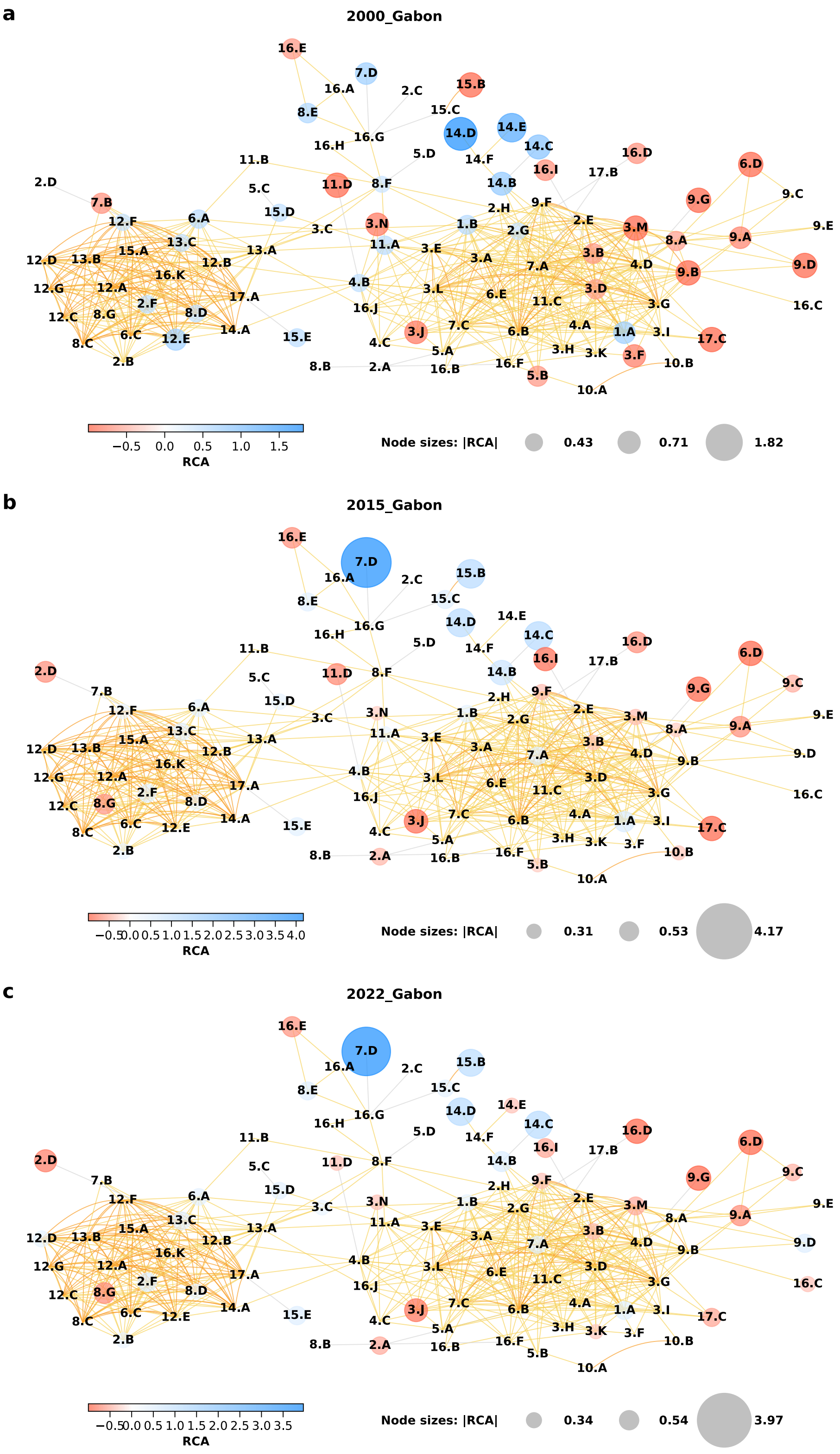

Supplementary Figure 78 | The SDG space of Gabon. Panels **a, b, c,** The SDG space in 2000, 2015, and 2022. The nodes in blue and orange represent the top 20 and bottom 20 SDG indicators in revealed comparative advantage (RCA) values, respectively. The node size represents the absolute value of RCA. From Supplementary Figure 12 to 177, countries are ranked by GDP/capita (current US$, 2022).

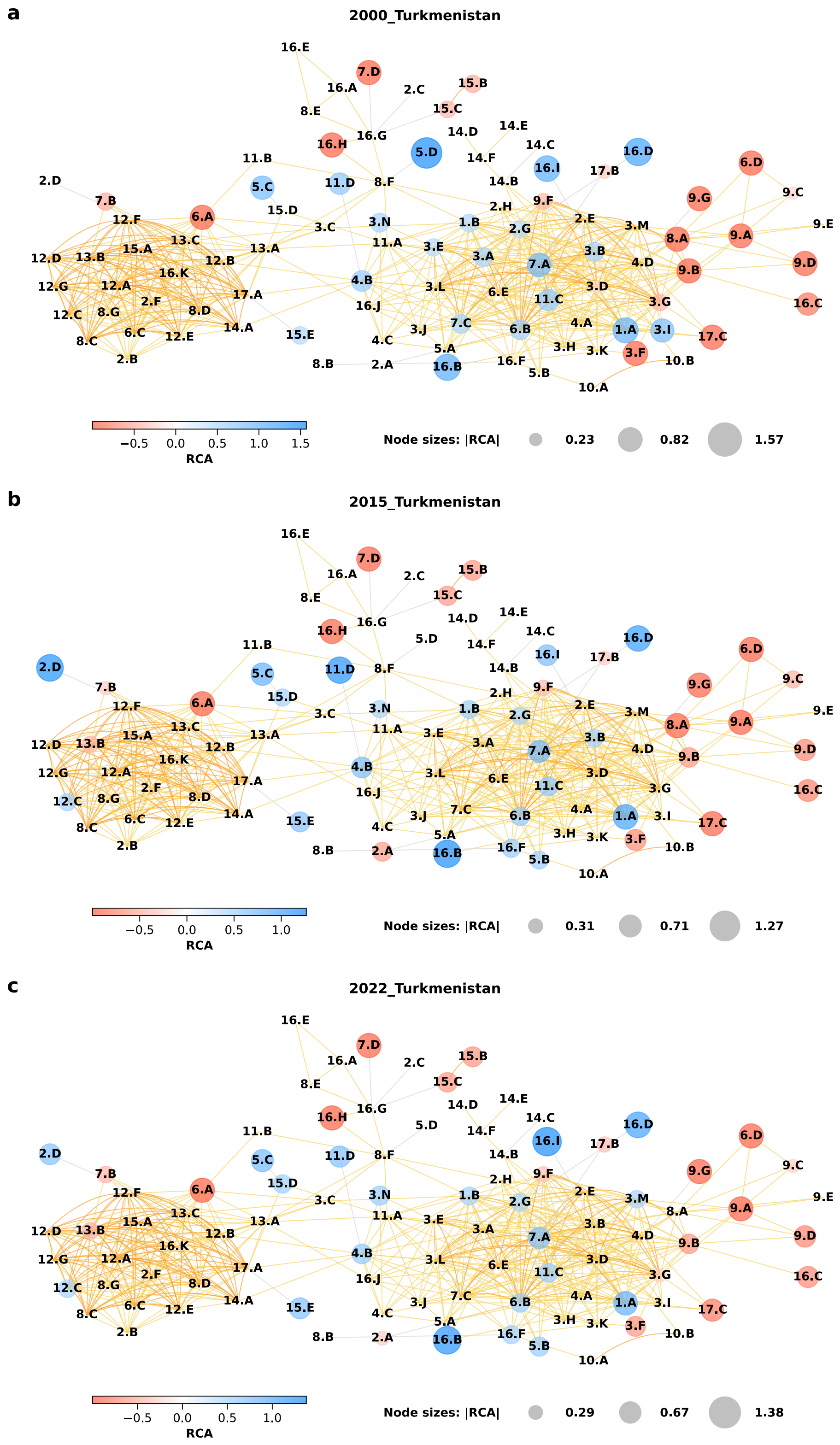

Supplementary Figure 79 | The SDG space of Turkmenistan. Panels **a, b, c,** The SDG space in 2000, 2015, and 2022. The nodes in blue and orange represent the top 20 and bottom 20 SDG indicators in revealed comparative advantage (RCA) values, respectively. The node size represents the absolute value of RCA. From Supplementary Figure 12 to 177, countries are ranked by GDP/capita (current US$, 2022).

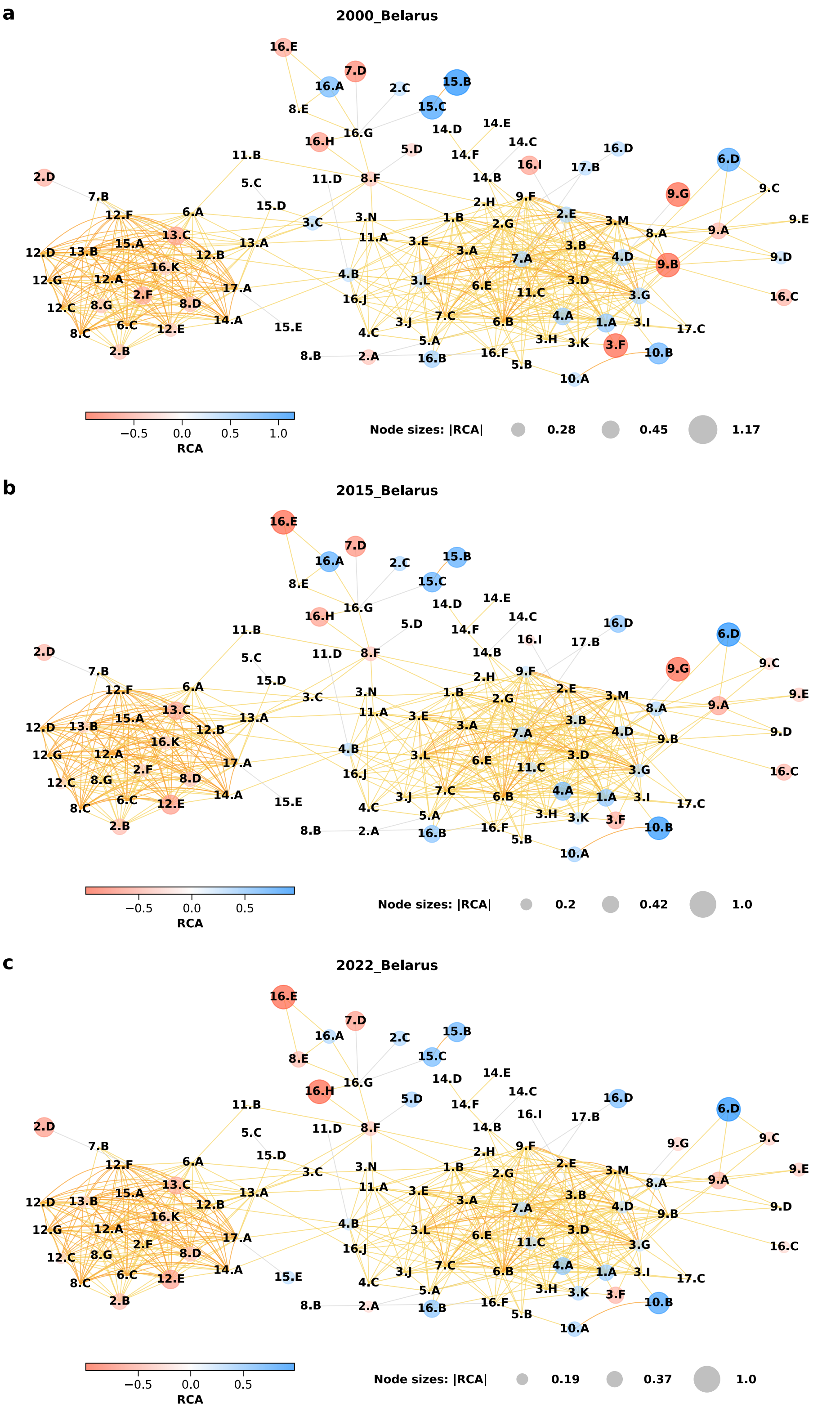

Supplementary Figure 80 | The SDG space of Belarus. Panels **a, b, c,** The SDG space in 2000, 2015, and 2022. The nodes in blue and orange represent the top 20 and bottom 20 SDG indicators in revealed comparative advantage (RCA) values, respectively. The node size represents the absolute value of RCA. From Supplementary Figure 12 to 177, countries are ranked by GDP/capita (current US$, 2022).

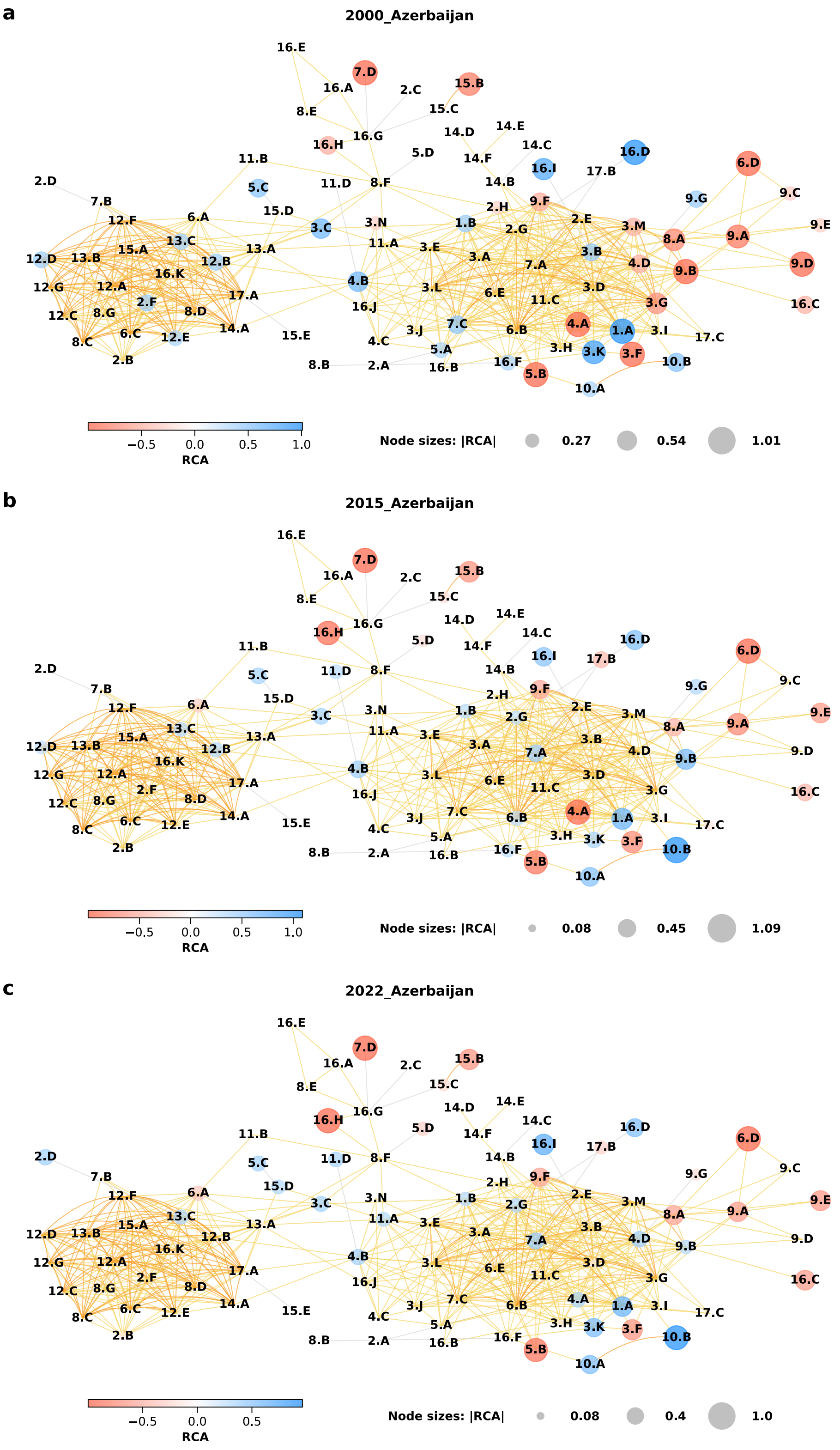

Supplementary Figure 81 | The SDG space of Azerbaijan. Panels **a, b, c,** The SDG space in 2000, 2015, and 2022. The nodes in blue and orange represent the top 20 and bottom 20 SDG indicators in revealed comparative advantage (RCA) values, respectively. The node size represents the absolute value of RCA. From Supplementary Figure 12 to 177, countries are ranked by GDP/capita (current US$, 2022).

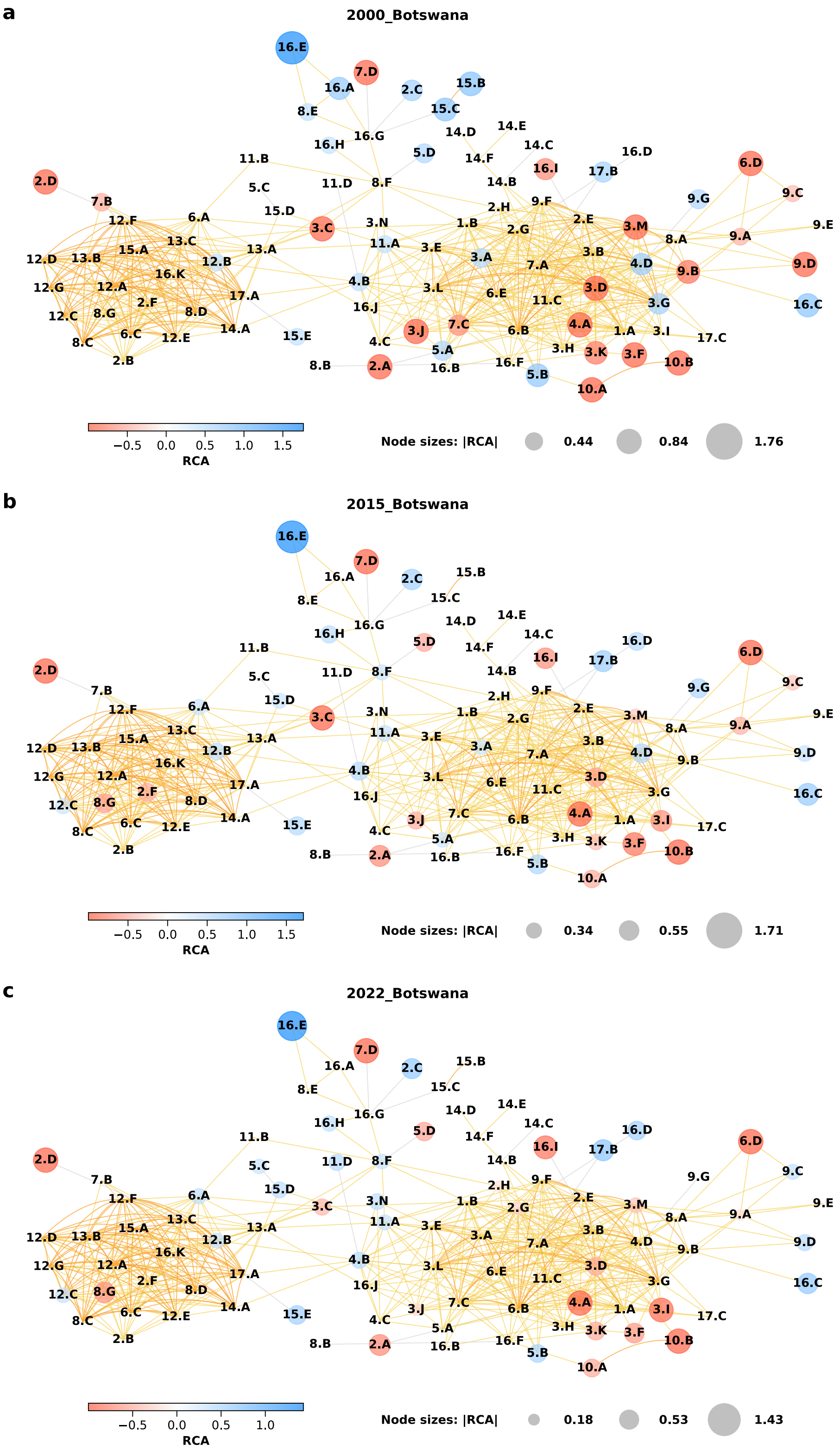

Supplementary Figure 82 | The SDG space of Botswana. Panels **a, b, c,** The SDG space in 2000, 2015, and 2022. The nodes in blue and orange represent the top 20 and bottom 20 SDG indicators in revealed comparative advantage (RCA) values, respectively. The node size represents the absolute value of RCA. From Supplementary Figure 12 to 177, countries are ranked by GDP/capita (current US$, 2022).

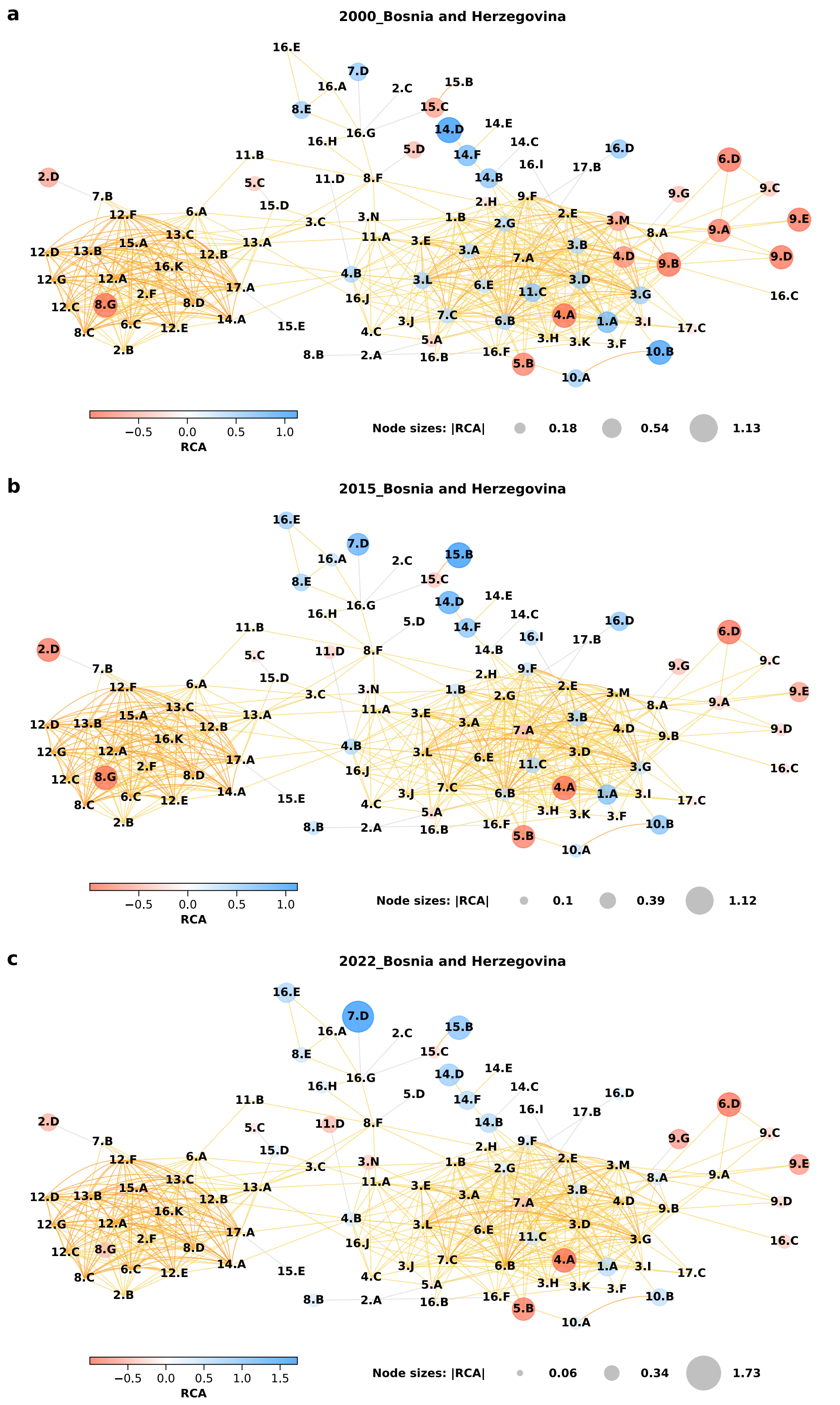

Supplementary Figure 83 | The SDG space of Bosnia and Herzegovina. Panels **a, b, c,** The SDG space in 2000, 2015, and 2022. The nodes in blue and orange represent the top 20 and bottom 20 SDG indicators in revealed comparative advantage (RCA) values, respectively. The node size represents the absolute value of RCA. From Supplementary Figure 12 to 177, countries are ranked by GDP/capita (current US$, 2022).

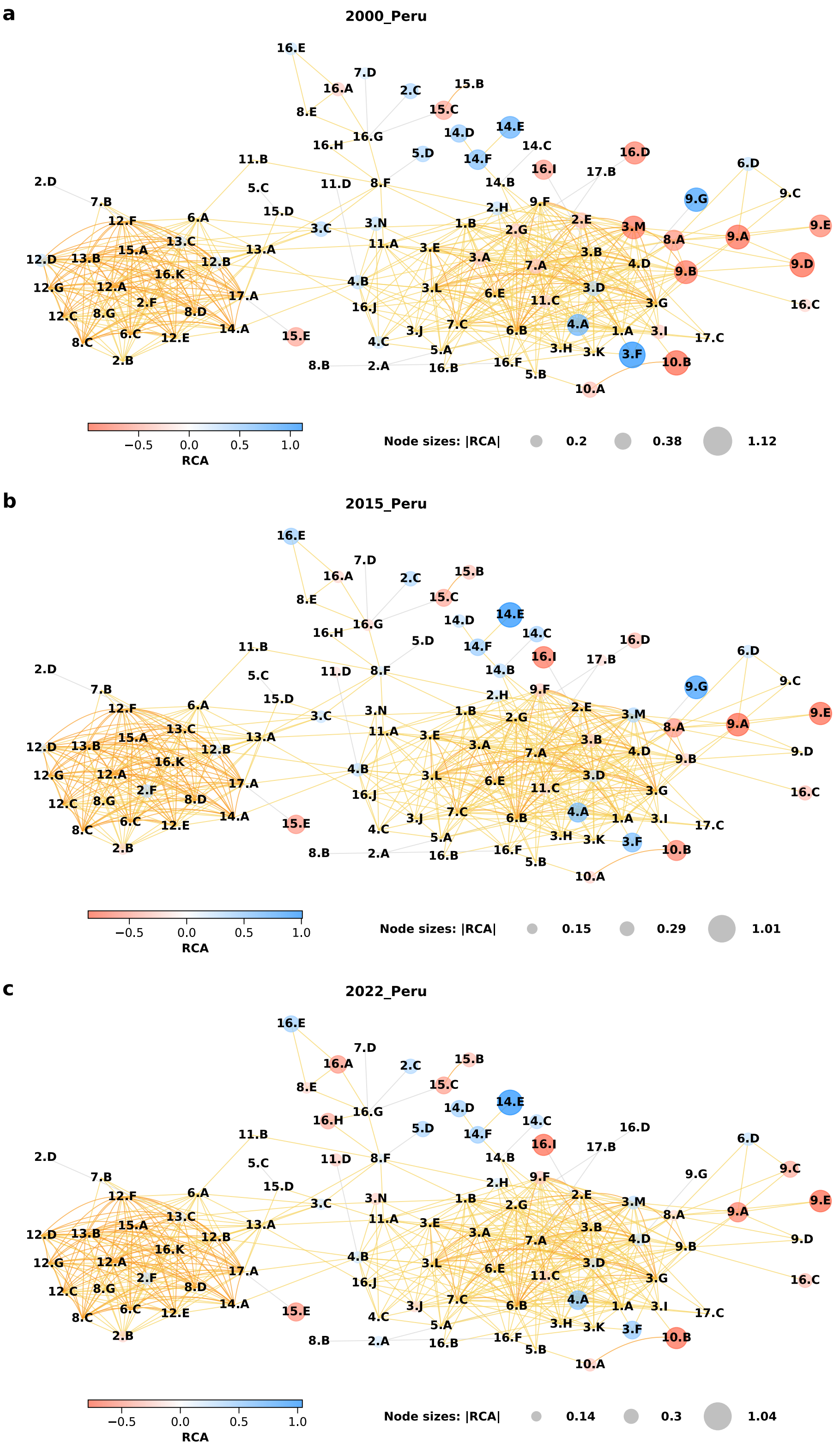

Supplementary Figure 84 | The SDG space of Peru. Panels **a, b, c,** The SDG space in 2000, 2015, and 2022. The nodes in blue and orange represent the top 20 and bottom 20 SDG indicators in revealed comparative advantage (RCA) values, respectively. The node size represents the absolute value of RCA. From Supplementary Figure 12 to 177, countries are ranked by GDP/capita (current US$, 2022).

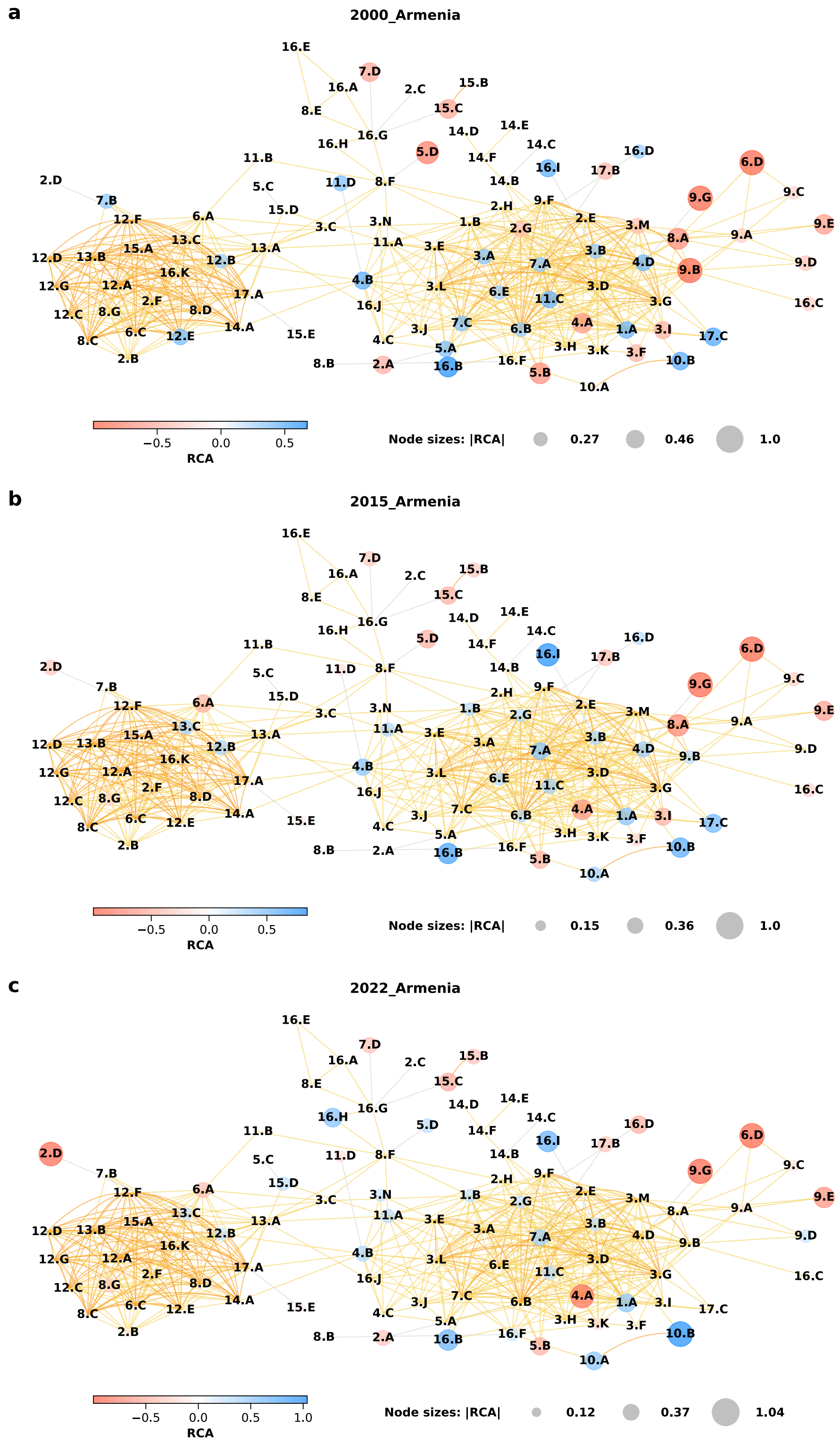

Supplementary Figure 85 | The SDG space of Armenia. Panels **a, b, c,** The SDG space in 2000, 2015, and 2022. The nodes in blue and orange represent the top 20 and bottom 20 SDG indicators in revealed comparative advantage (RCA) values, respectively. The node size represents the absolute value of RCA. From Supplementary Figure 12 to 177, countries are ranked by GDP/capita (current US$, 2022).

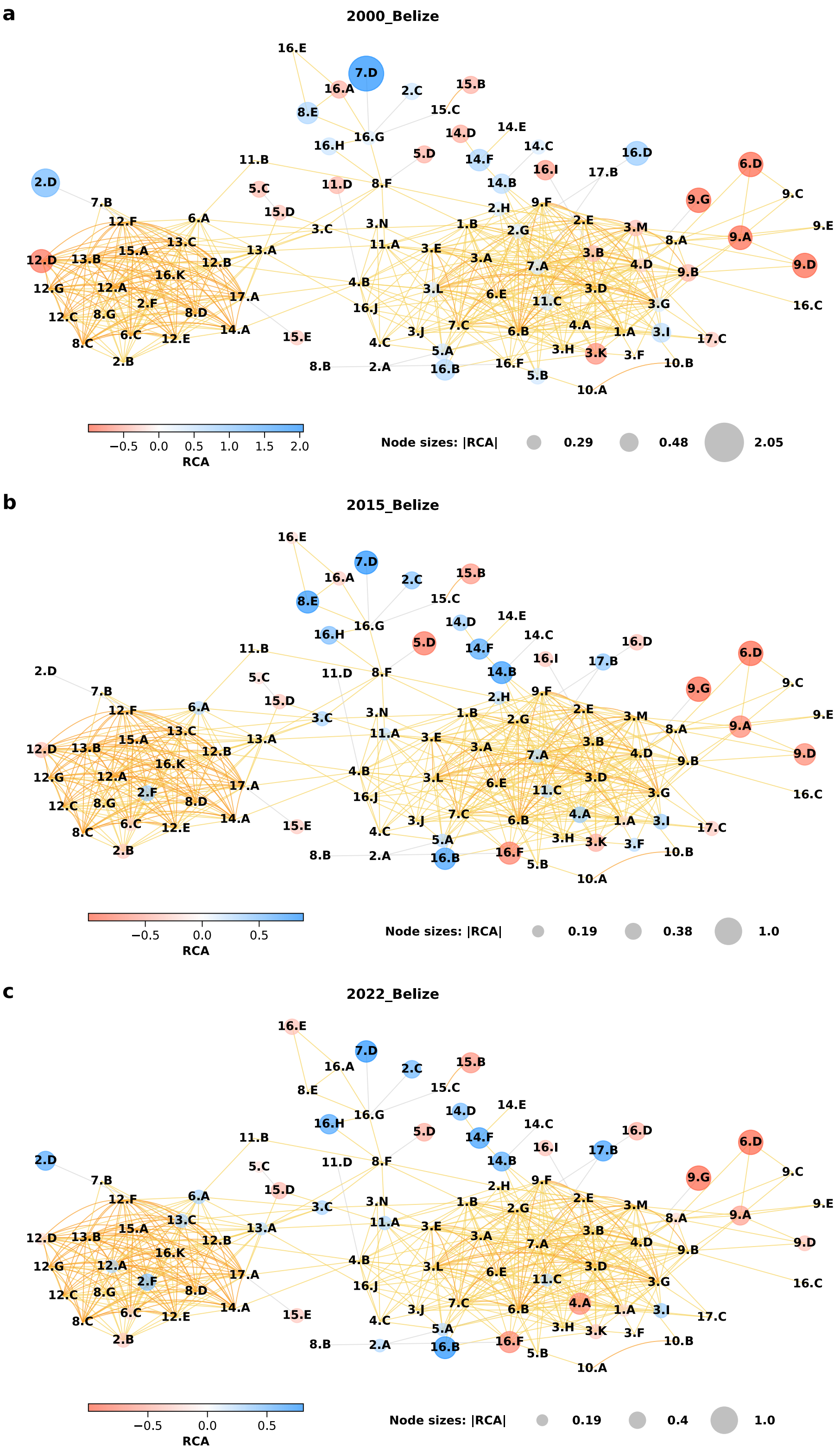

Supplementary Figure 86 | The SDG space of Belize. Panels **a, b, c,** The SDG space in 2000, 2015, and 2022. The nodes in blue and orange represent the top 20 and bottom 20 SDG indicators in revealed comparative advantage (RCA) values, respectively. The node size represents the absolute value of RCA. From Supplementary Figure 12 to 177, countries are ranked by GDP/capita (current US$, 2022).

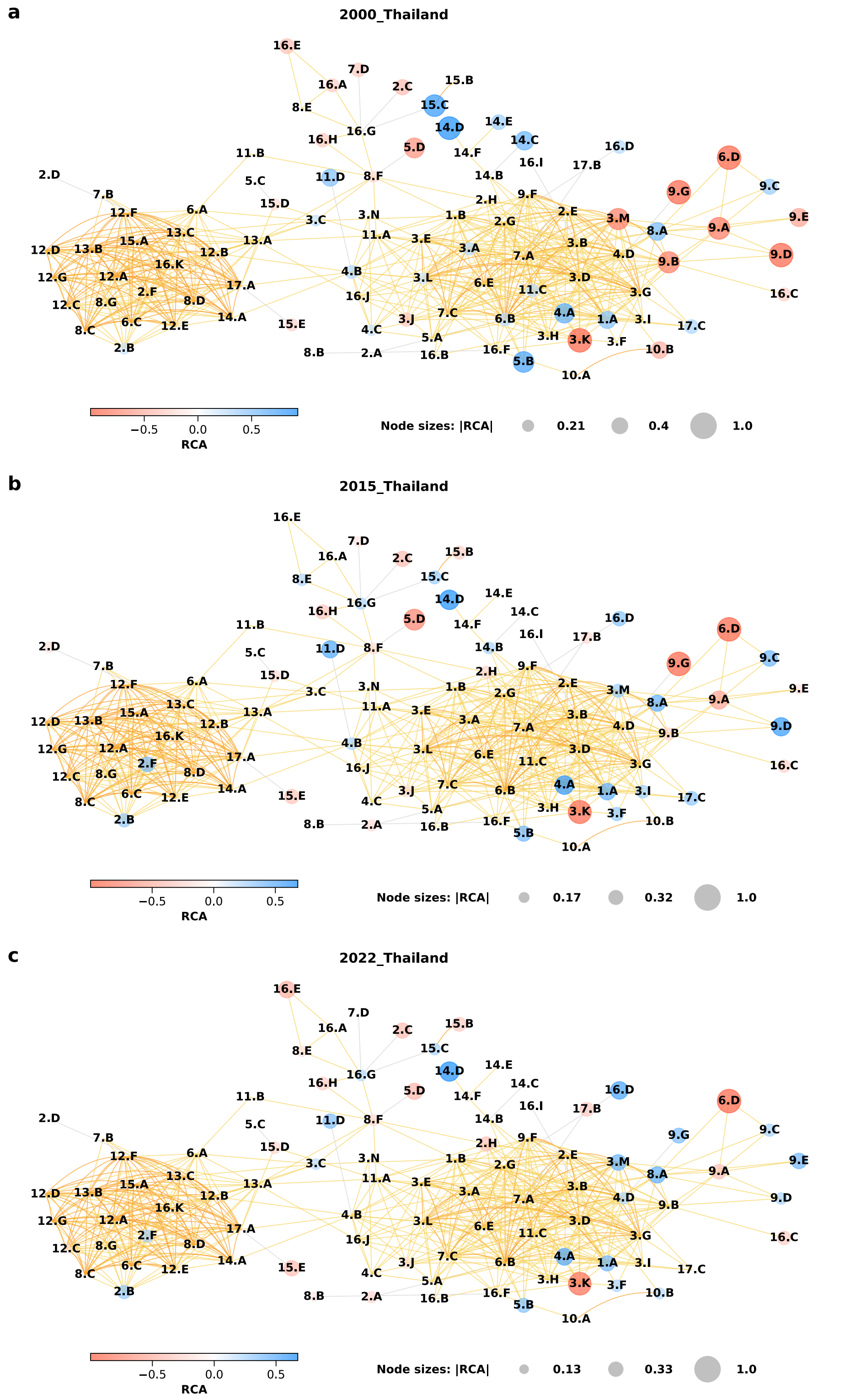

Supplementary Figure 87 | The SDG space of Thailand. Panels **a, b, c,** The SDG space in 2000, 2015, and 2022. The nodes in blue and orange represent the top 20 and bottom 20 SDG indicators in revealed comparative advantage (RCA) values, respectively. The node size represents the absolute value of RCA. From Supplementary Figure 12 to 177, countries are ranked by GDP/capita (current US$, 2022).

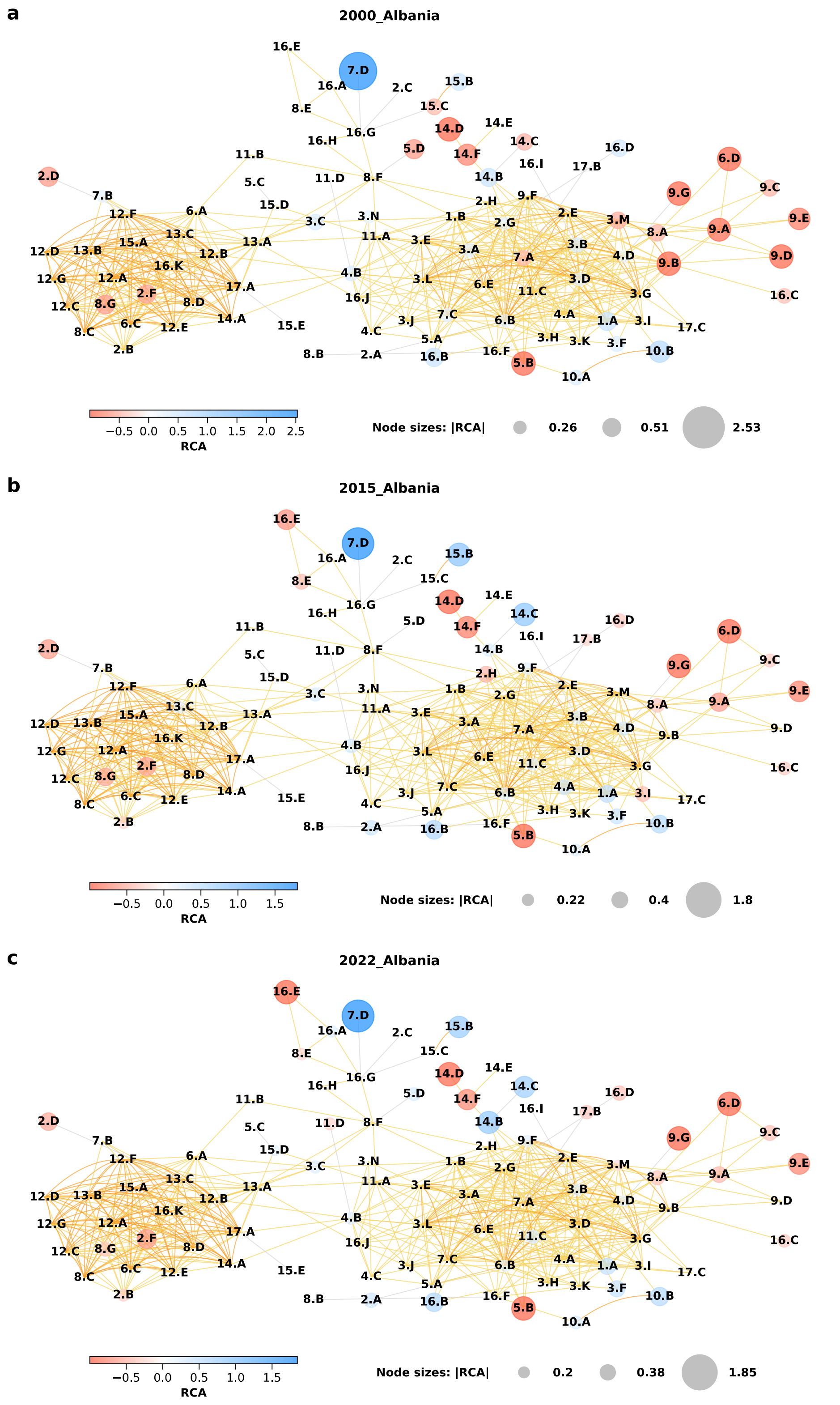

Supplementary Figure 88 | The SDG space of Albania. Panels **a, b, c,** The SDG space in 2000, 2015, and 2022. The nodes in blue and orange represent the top 20 and bottom 20 SDG indicators in revealed comparative advantage (RCA) values, respectively. The node size represents the absolute value of RCA. From Supplementary Figure 12 to 177, countries are ranked by GDP/capita (current US$, 2022).

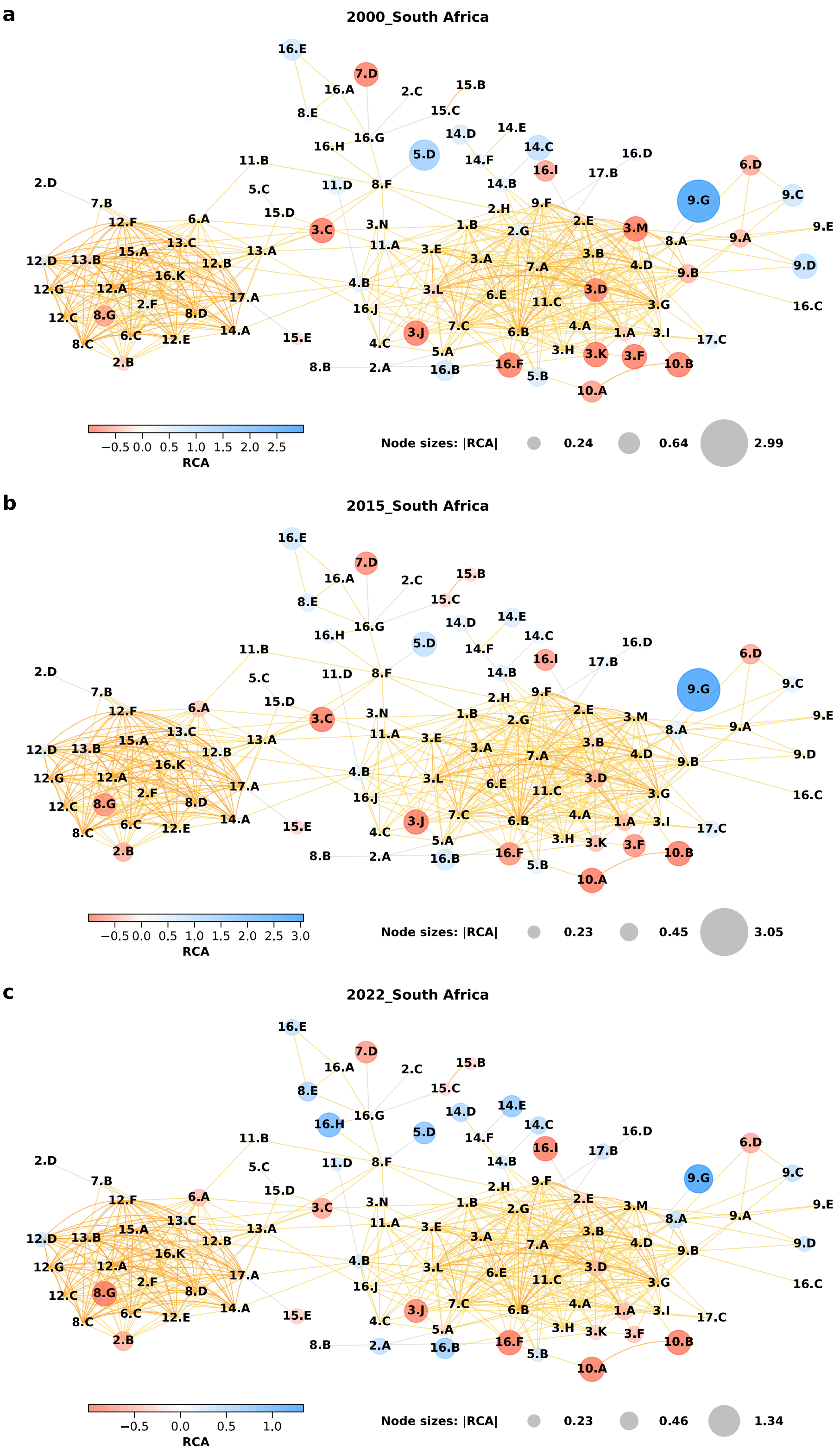

Supplementary Figure 89 | The SDG space of South Africa. Panels **a, b, c,** The SDG space in 2000, 2015, and 2022. The nodes in blue and orange represent the top 20 and bottom 20 SDG indicators in revealed comparative advantage (RCA) values, respectively. The node size represents the absolute value of RCA. From Supplementary Figure 12 to 177, countries are ranked by GDP/capita (current US$, 2022).

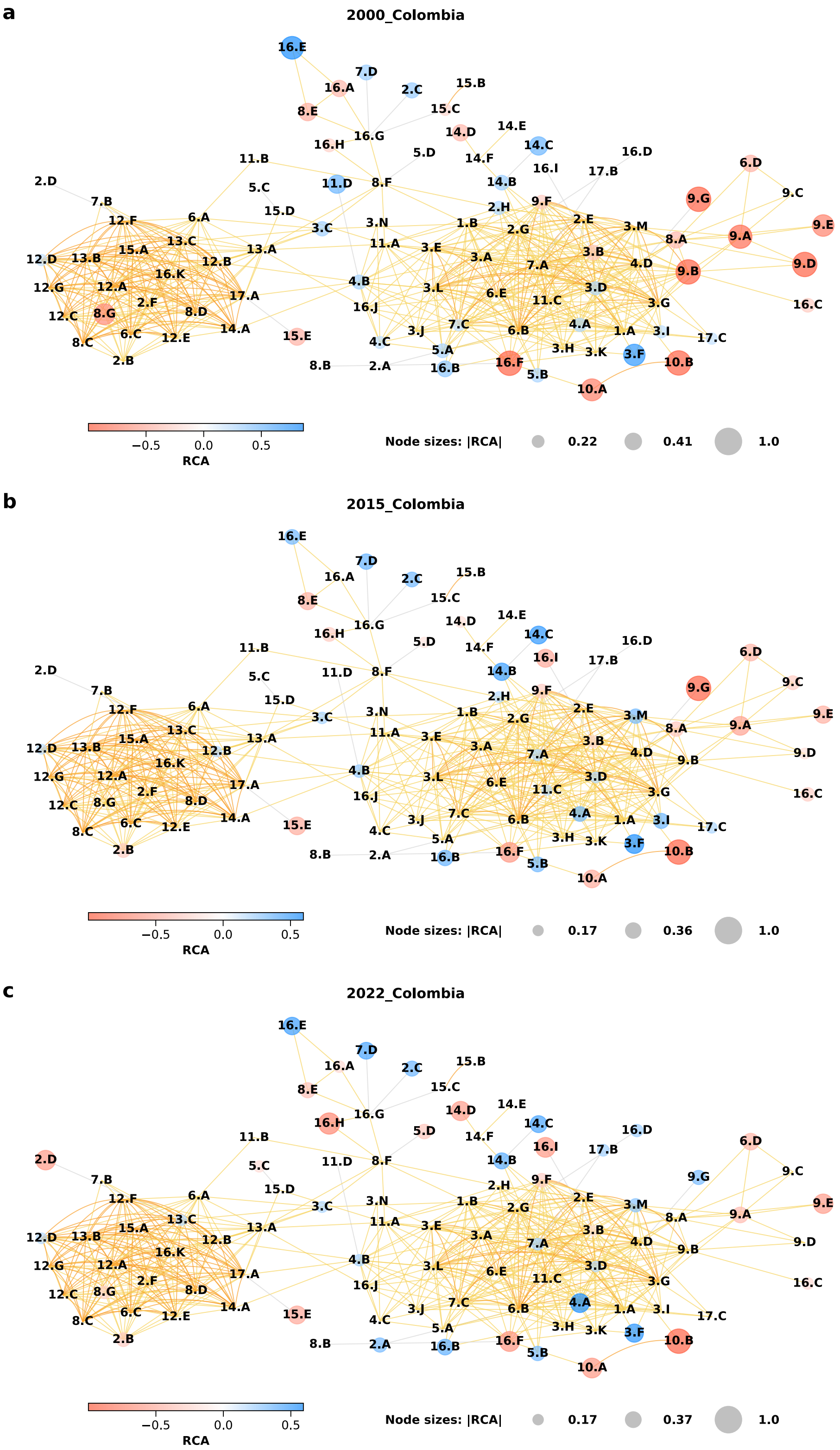

Supplementary Figure 90 | The SDG space of Colombia. Panels **a, b, c,** The SDG space in 2000, 2015, and 2022. The nodes in blue and orange represent the top 20 and bottom 20 SDG indicators in revealed comparative advantage (RCA) values, respectively. The node size represents the absolute value of RCA. From Supplementary Figure 12 to 177, countries are ranked by GDP/capita (current US$, 2022).

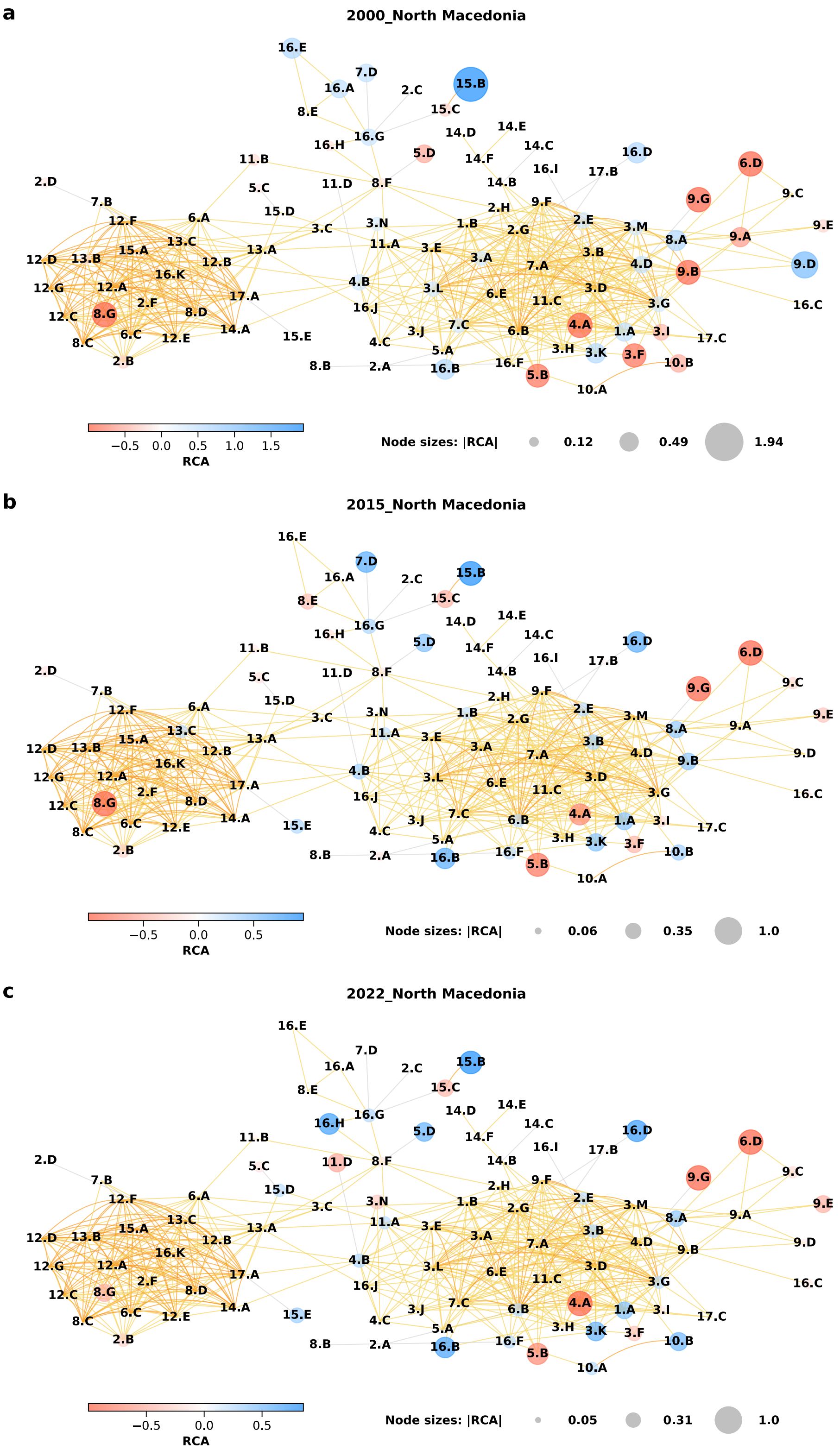

Supplementary Figure 91 | The SDG space of North Macedonia. Panels **a, b, c,** The SDG space in 2000, 2015, and 2022. The nodes in blue and orange represent the top 20 and bottom 20 SDG indicators in revealed comparative advantage (RCA) values, respectively. The node size represents the absolute value of RCA. From Supplementary Figure 12 to 177, countries are ranked by GDP/capita (current US$, 2022).

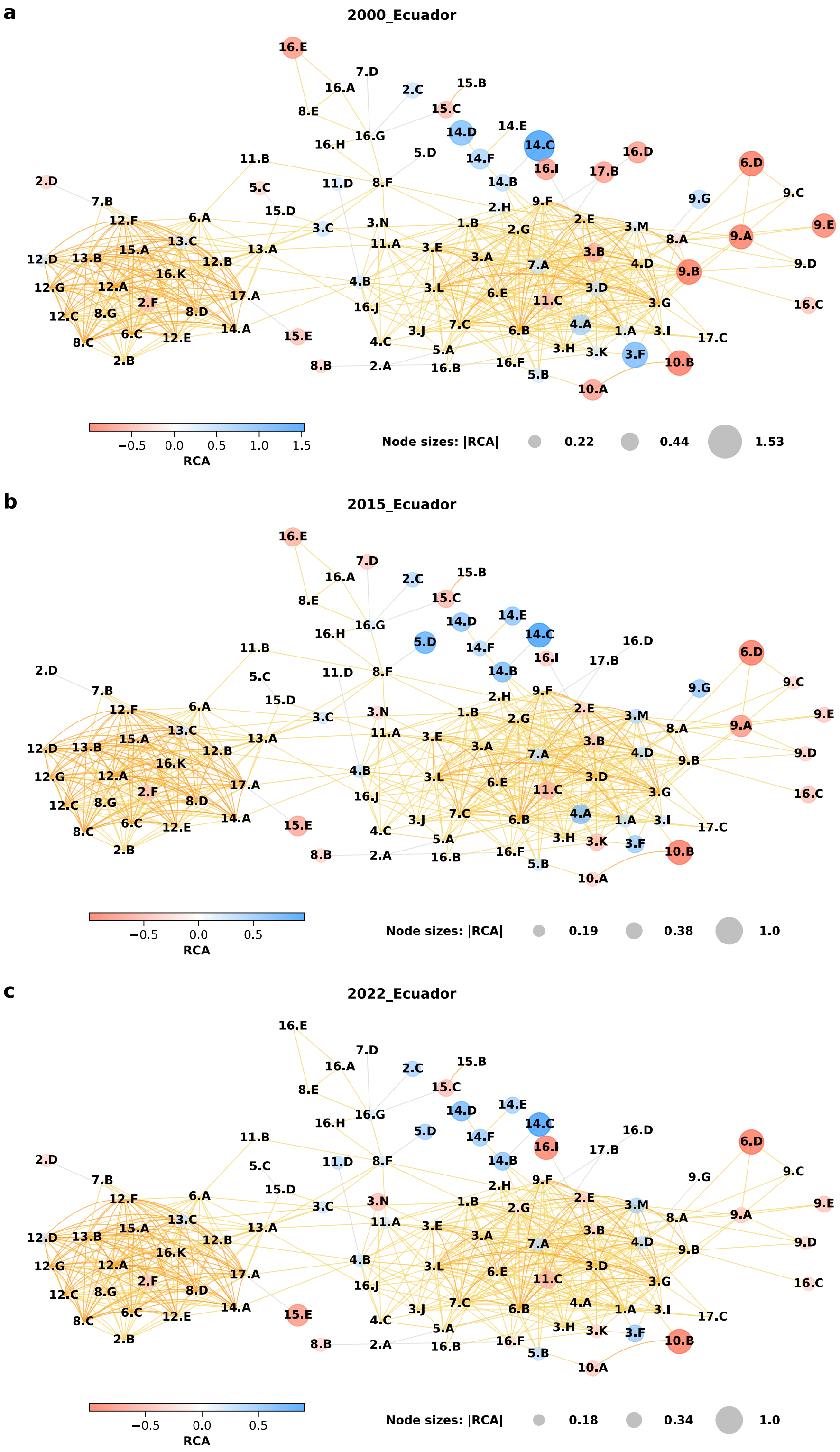

Supplementary Figure 92 | The SDG space of Ecuador. Panels **a, b, c,** The SDG space in 2000, 2015, and 2022. The nodes in blue and orange represent the top 20 and bottom 20 SDG indicators in revealed comparative advantage (RCA) values, respectively. The node size represents the absolute value of RCA. From Supplementary Figure 12 to 177, countries are ranked by GDP/capita (current US$, 2022).

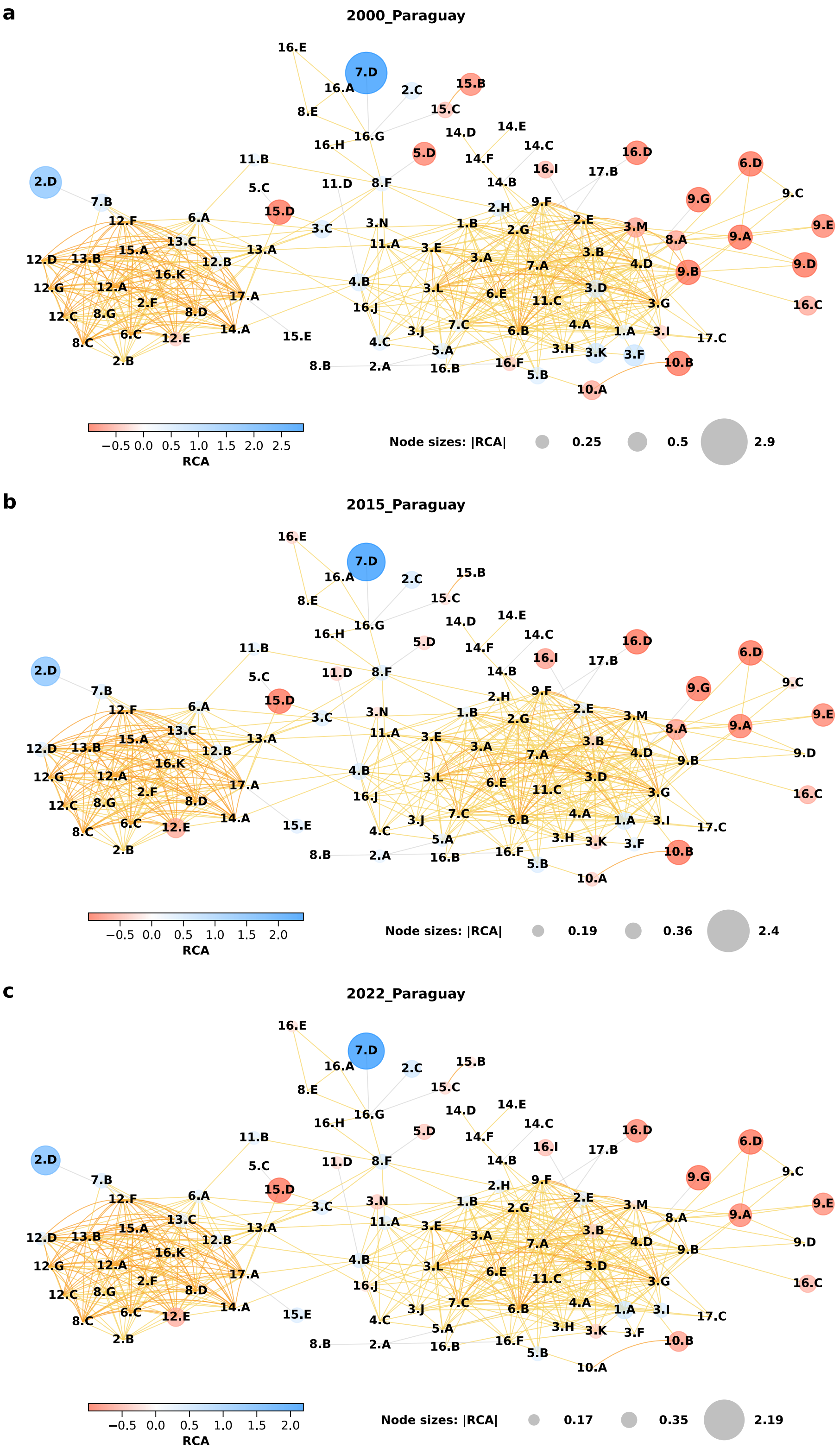

Supplementary Figure 93 | The SDG space of Paraguay. Panels **a, b, c,** The SDG space in 2000, 2015, and 2022. The nodes in blue and orange represent the top 20 and bottom 20 SDG indicators in revealed comparative advantage (RCA) values, respectively. The node size represents the absolute value of RCA. From Supplementary Figure 12 to 177, countries are ranked by GDP/capita (current US$, 2022).

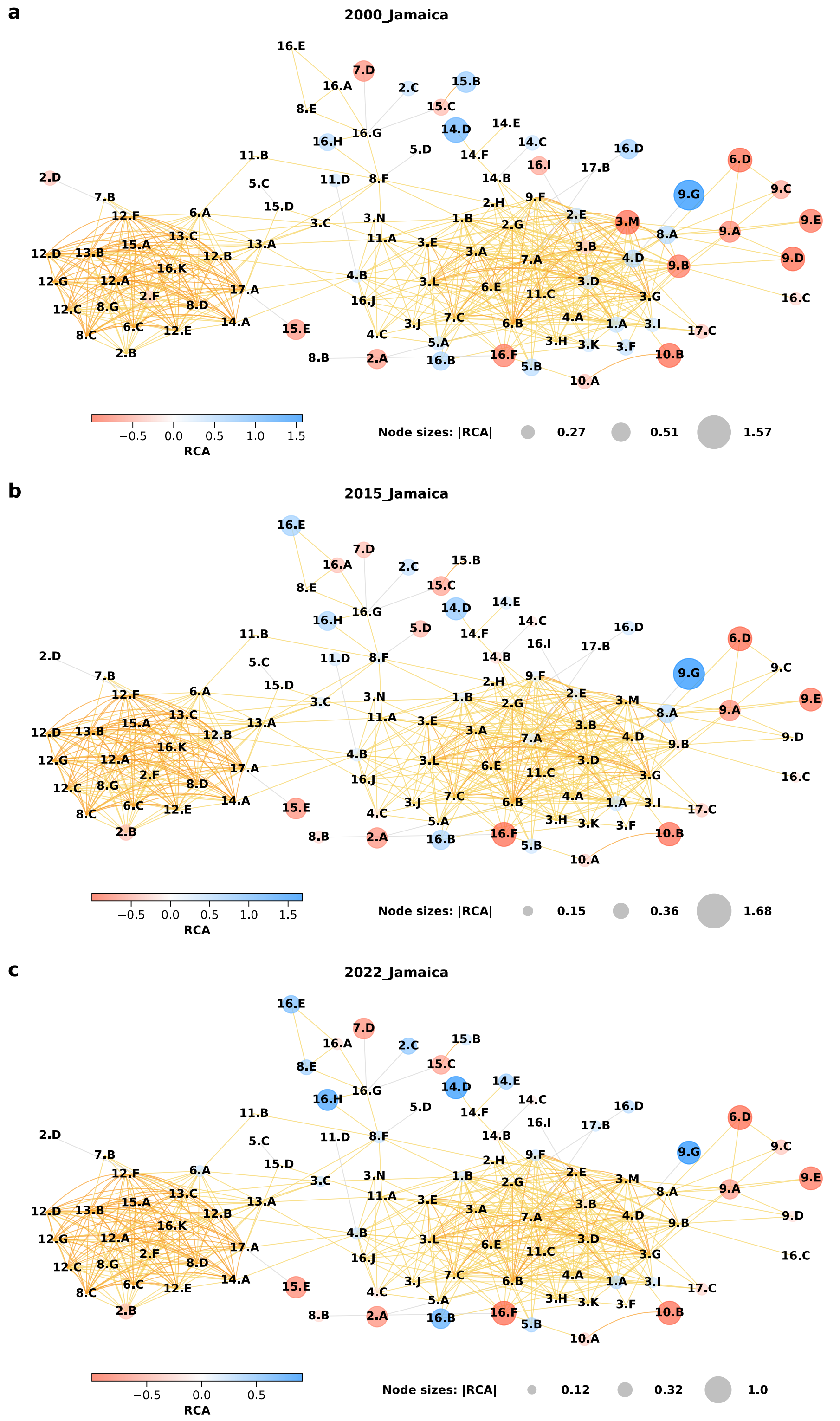

Supplementary Figure 94 | The SDG space of Jamaica. Panels **a, b, c,** The SDG space in 2000, 2015, and 2022. The nodes in blue and orange represent the top 20 and bottom 20 SDG indicators in revealed comparative advantage (RCA) values, respectively. The node size represents the absolute value of RCA. From Supplementary Figure 12 to 177, countries are ranked by GDP/capita (current US$, 2022).

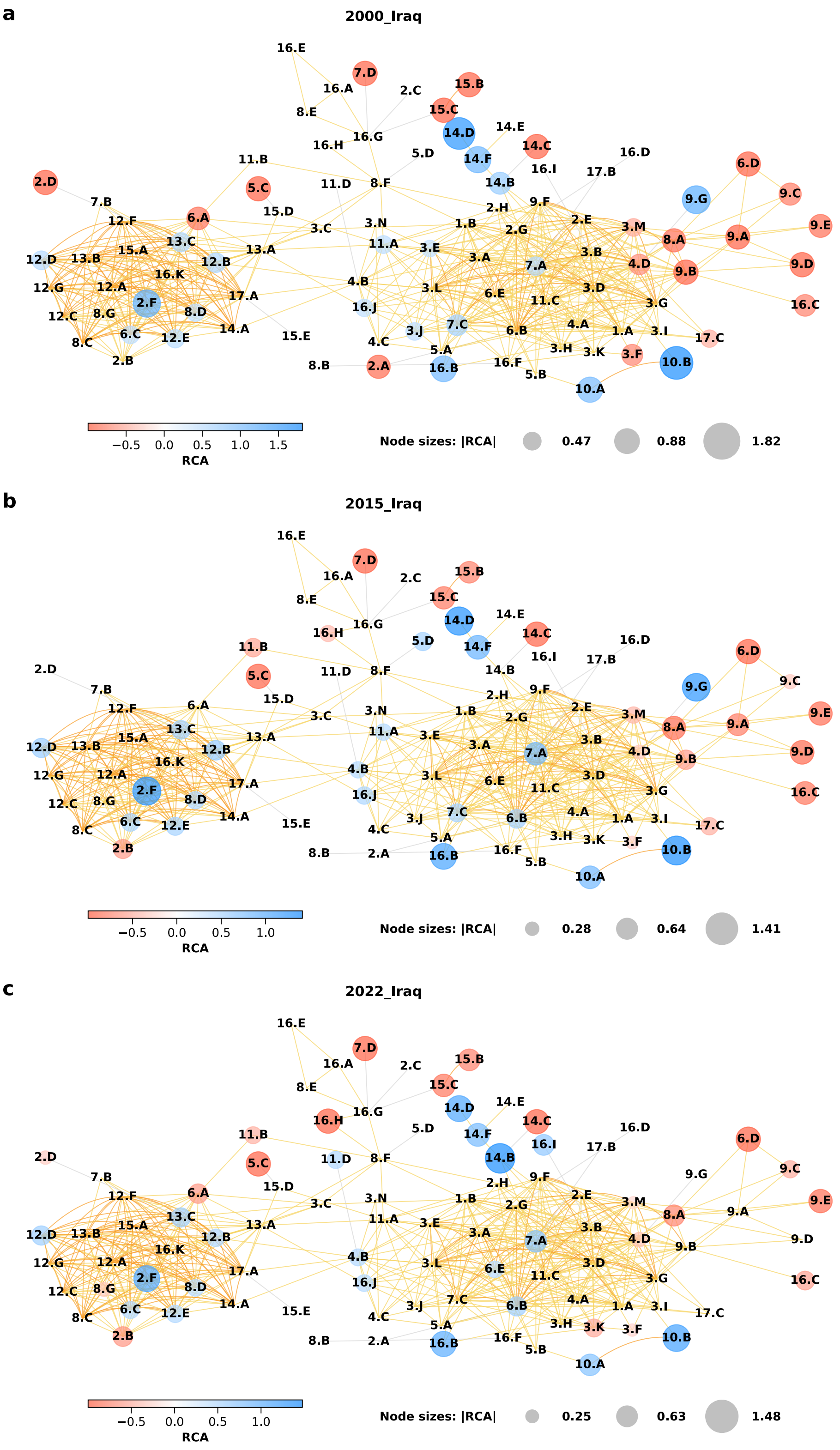

Supplementary Figure 95 | The SDG space of Iraq. Panels **a, b, c,** The SDG space in 2000, 2015, and 2022. The nodes in blue and orange represent the top 20 and bottom 20 SDG indicators in revealed comparative advantage (RCA) values, respectively. The node size represents the absolute value of RCA. From Supplementary Figure 12 to 177, countries are ranked by GDP/capita (current US$, 2022).

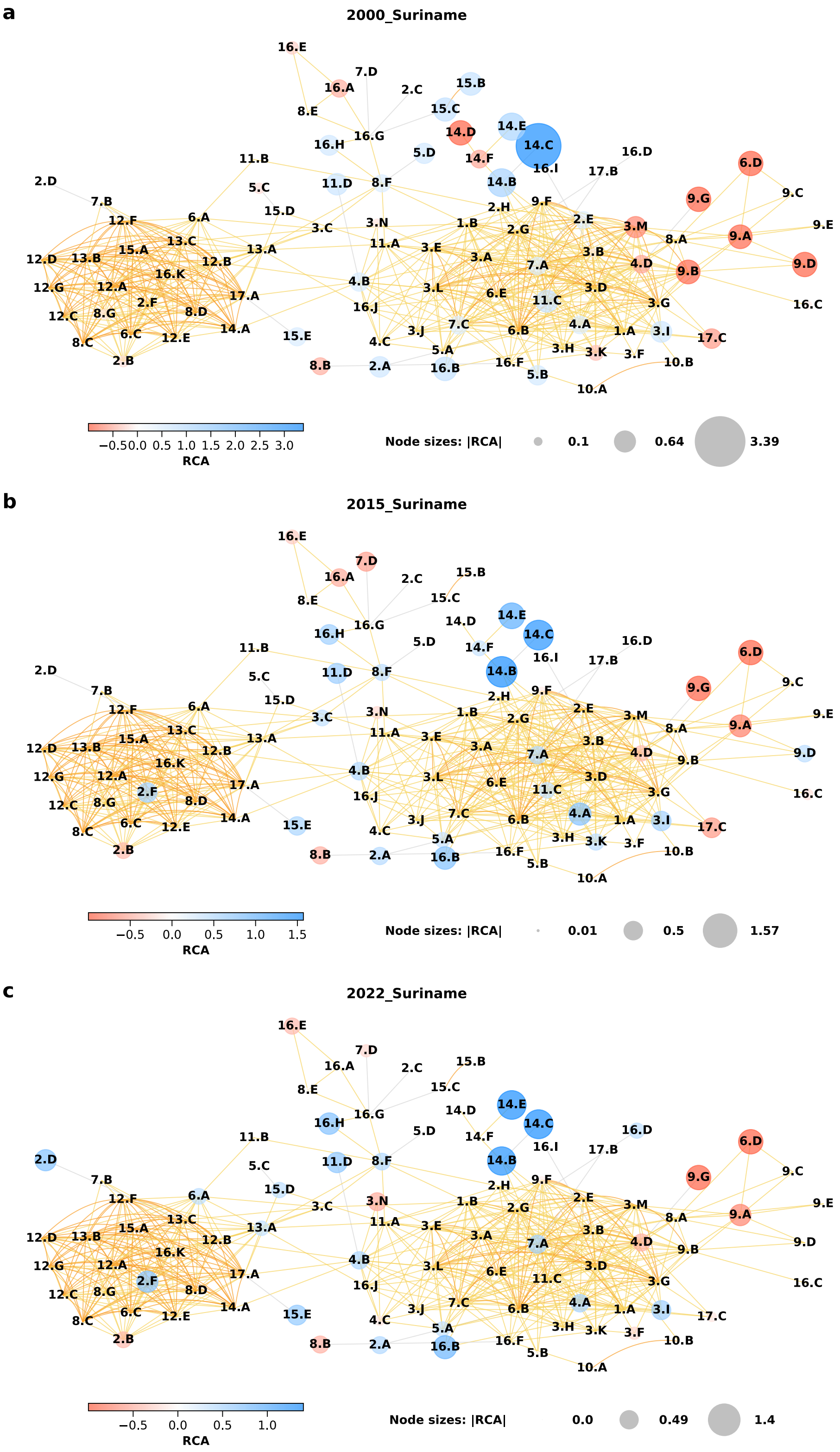

Supplementary Figure 96 | The SDG space of Suriname. Panels **a, b, c,** The SDG space in 2000, 2015, and 2022. The nodes in blue and orange represent the top 20 and bottom 20 SDG indicators in revealed comparative advantage (RCA) values, respectively. The node size represents the absolute value of RCA. From Supplementary Figure 12 to 177, countries are ranked by GDP/capita (current US$, 2022).

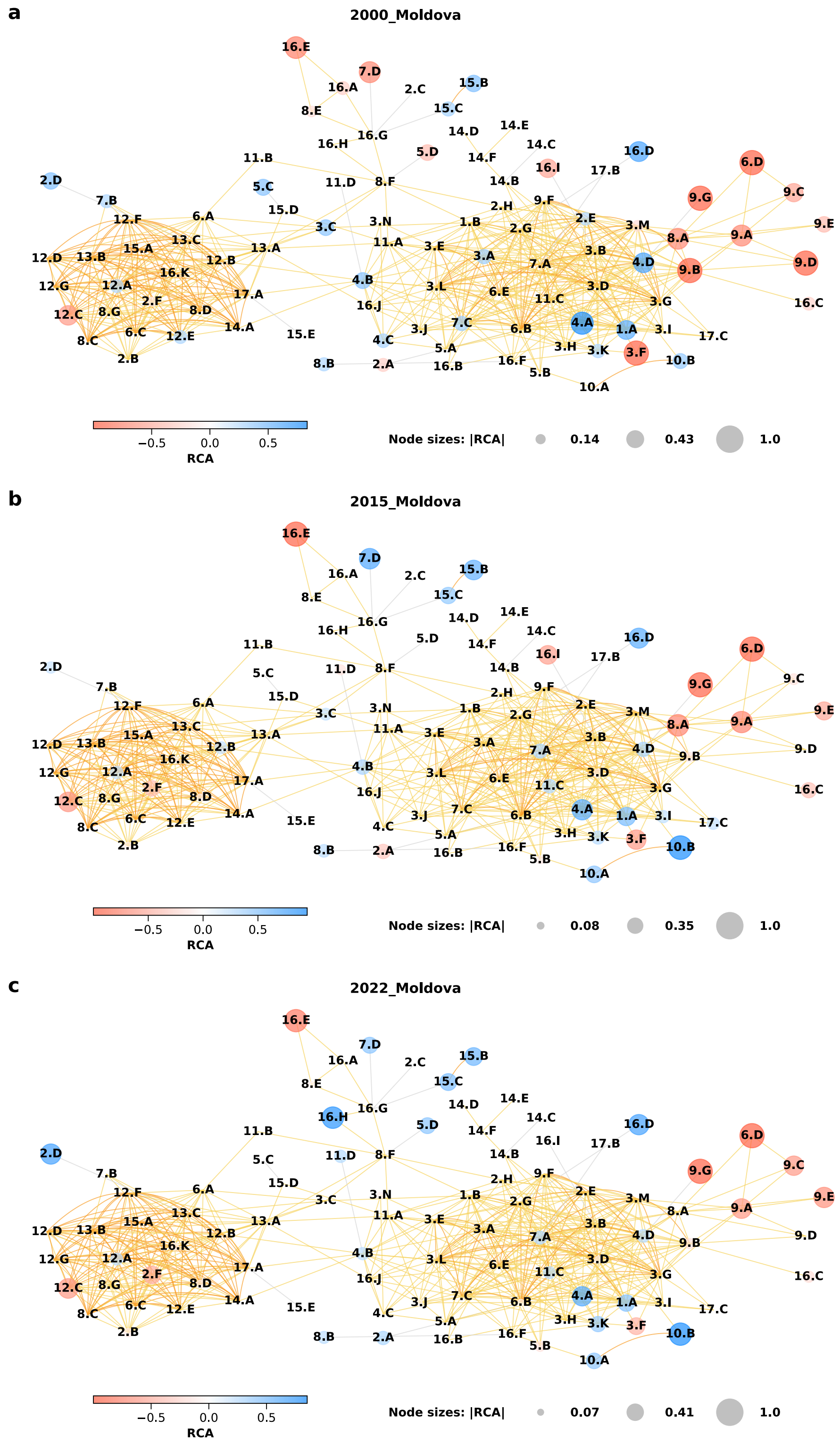

Supplementary Figure 97 | The SDG space of Moldova. Panels **a, b, c,** The SDG space in 2000, 2015, and 2022. The nodes in blue and orange represent the top 20 and bottom 20 SDG indicators in revealed comparative advantage (RCA) values, respectively. The node size represents the absolute value of RCA. From Supplementary Figure 12 to 177, countries are ranked by GDP/capita (current US$, 2022).

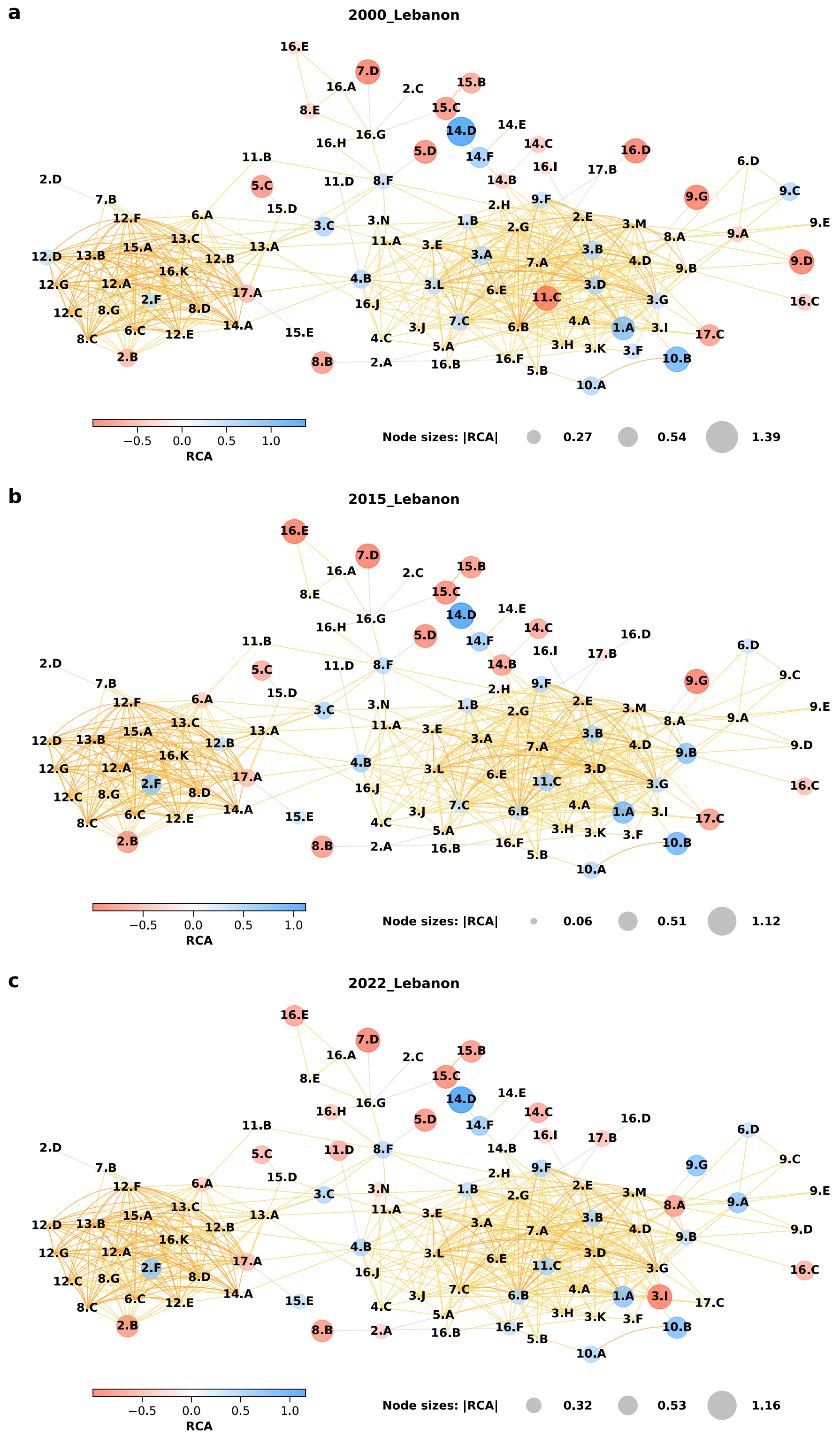

Supplementary Figure 98 | The SDG space of Lebanon. Panels **a, b, c,** The SDG space in 2000, 2015, and 2022. The nodes in blue and orange represent the top 20 and bottom 20 SDG indicators in revealed comparative advantage (RCA) values, respectively. The node size represents the absolute value of RCA. From Supplementary Figure 12 to 177, countries are ranked by GDP/capita (current US$, 2022).

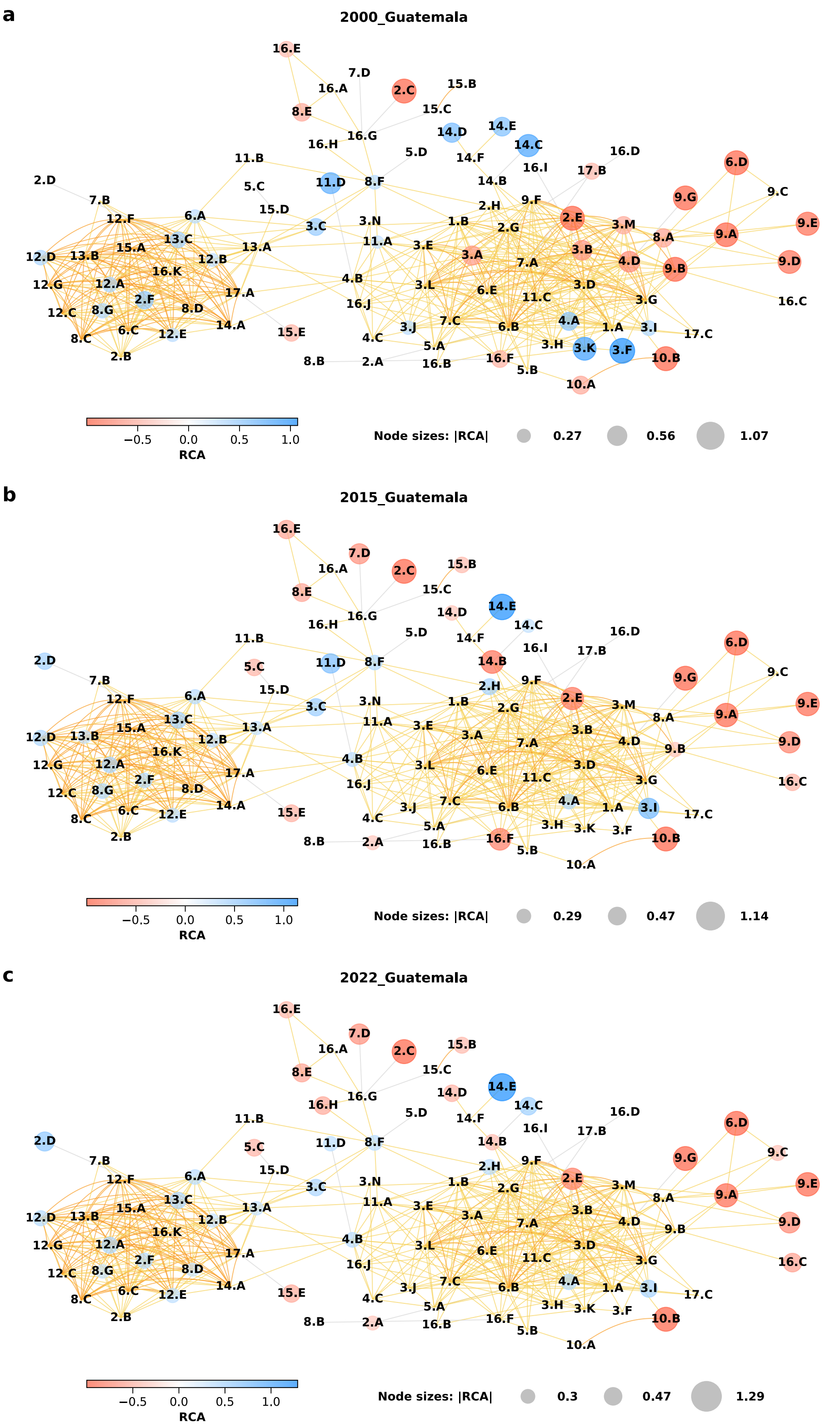

Supplementary Figure 99 | The SDG space of Guatemala. Panels **a, b, c,** The SDG space in 2000, 2015, and 2022. The nodes in blue and orange represent the top 20 and bottom 20 SDG indicators in revealed comparative advantage (RCA) values, respectively. The node size represents the absolute value of RCA. From Supplementary Figure 12 to 177, countries are ranked by GDP/capita (current US$, 2022).

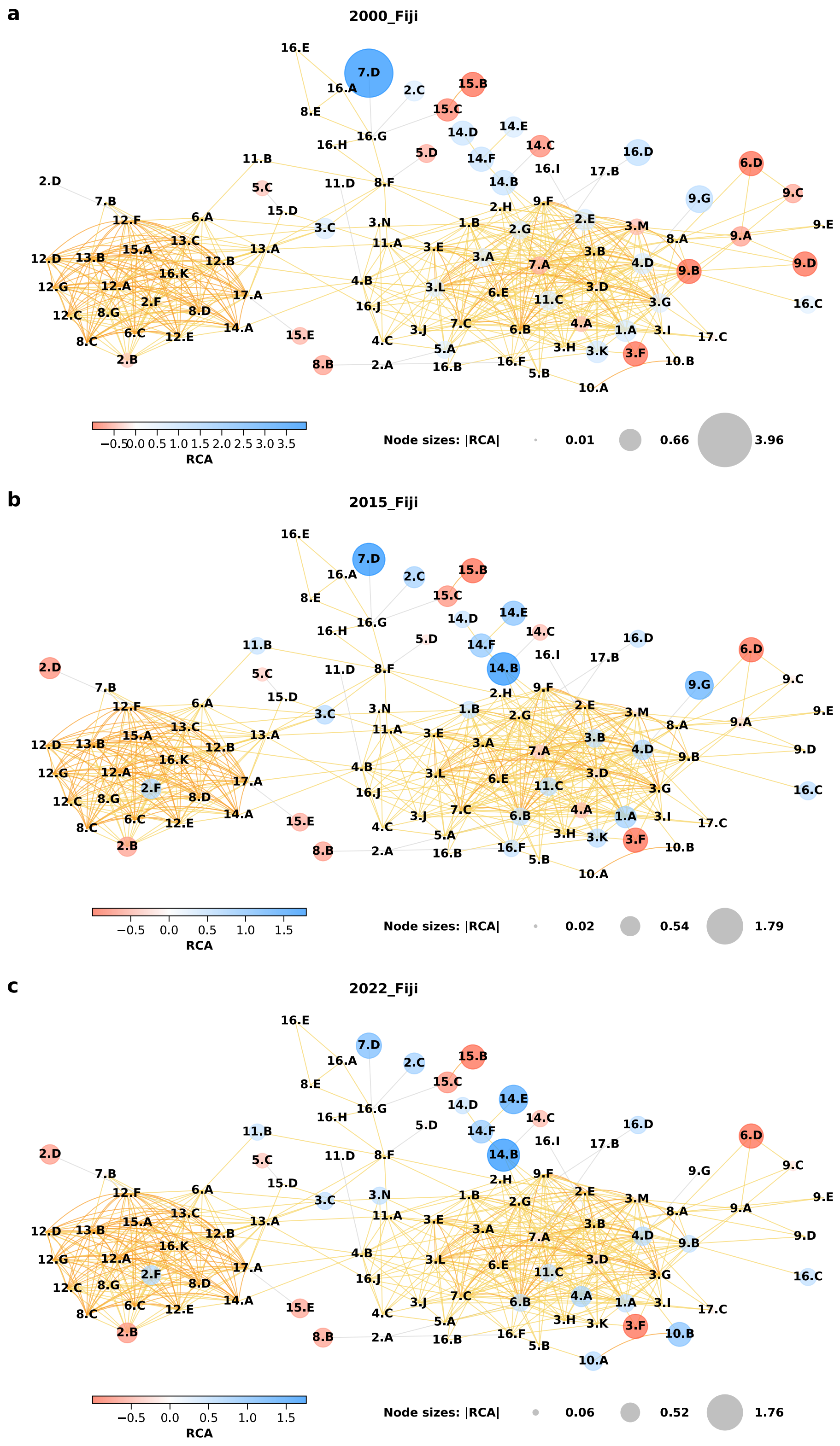

Supplementary Figure 100 | The SDG space of Fiji. Panels **a, b, c,** The SDG space in 2000, 2015, and 2022. The nodes in blue and orange represent the top 20 and bottom 20 SDG indicators in revealed comparative advantage (RCA) values, respectively. The node size represents the absolute value of RCA. From Supplementary Figure 12 to 177, countries are ranked by GDP/capita (current US$, 2022).

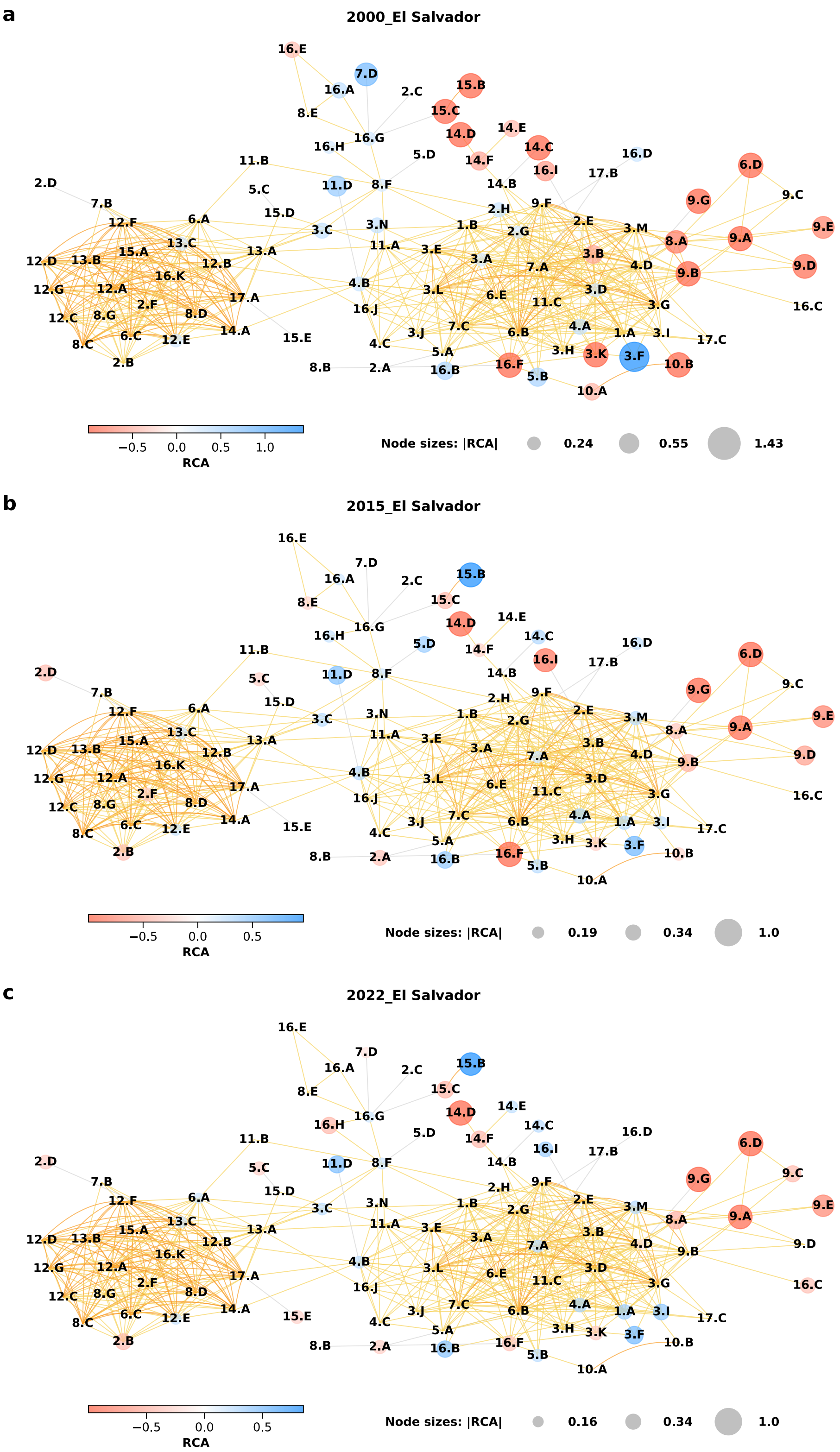

Supplementary Figure 101 | The SDG space of El Salvador. Panels **a, b, c,** The SDG space in 2000, 2015, and 2022. The nodes in blue and orange represent the top 20 and bottom 20 SDG indicators in revealed comparative advantage (RCA) values, respectively. The node size represents the absolute value of RCA. From Supplementary Figure 12 to 177, countries are ranked by GDP/capita (current US$, 2022).

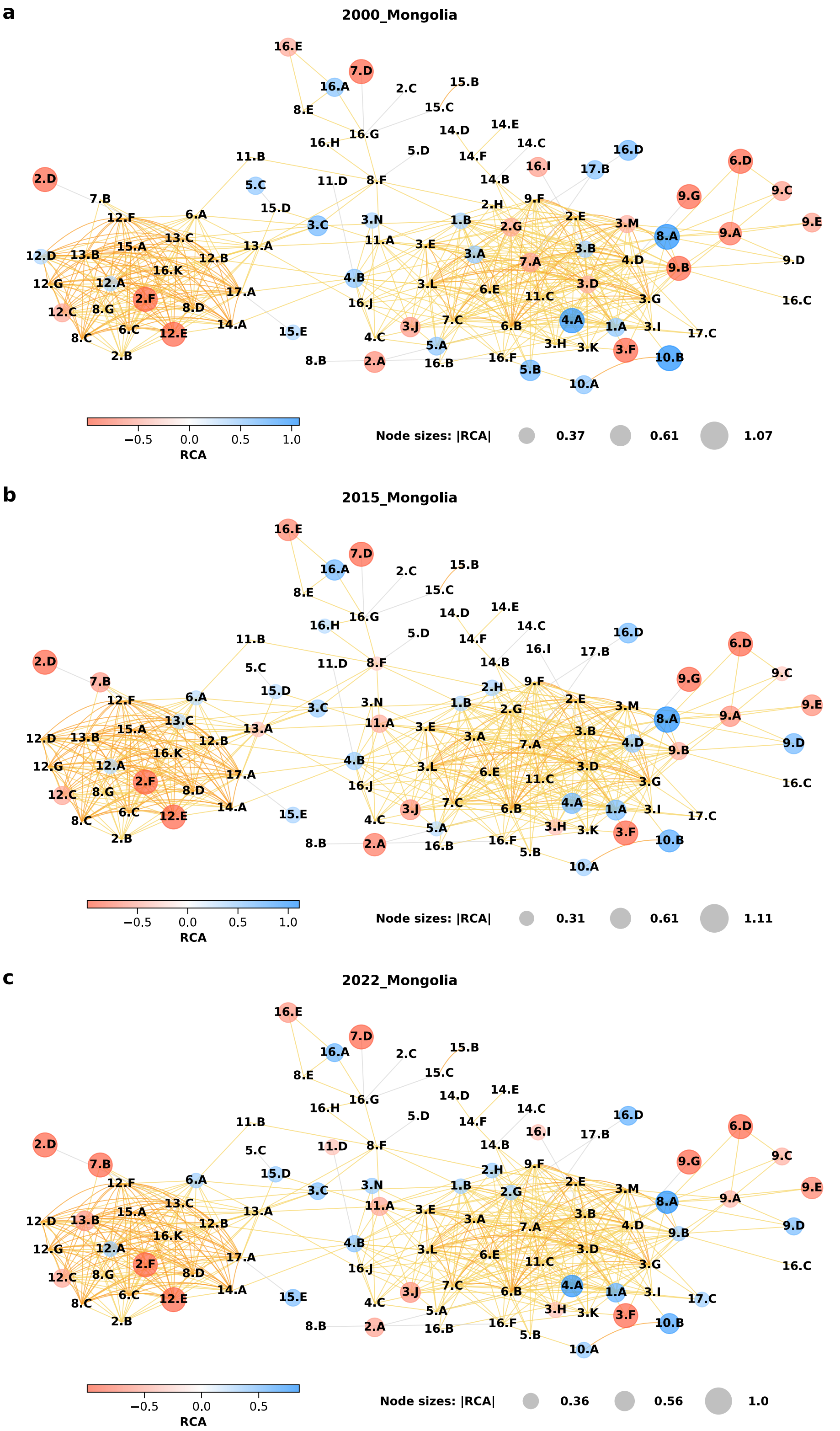

Supplementary Figure 102 | The SDG space of Mongolia. Panels **a, b, c,** The SDG space in 2000, 2015, and 2022. The nodes in blue and orange represent the top 20 and bottom 20 SDG indicators in revealed comparative advantage (RCA) values, respectively. The node size represents the absolute value of RCA. From Supplementary Figure 12 to 177, countries are ranked by GDP/capita (current US$, 2022).

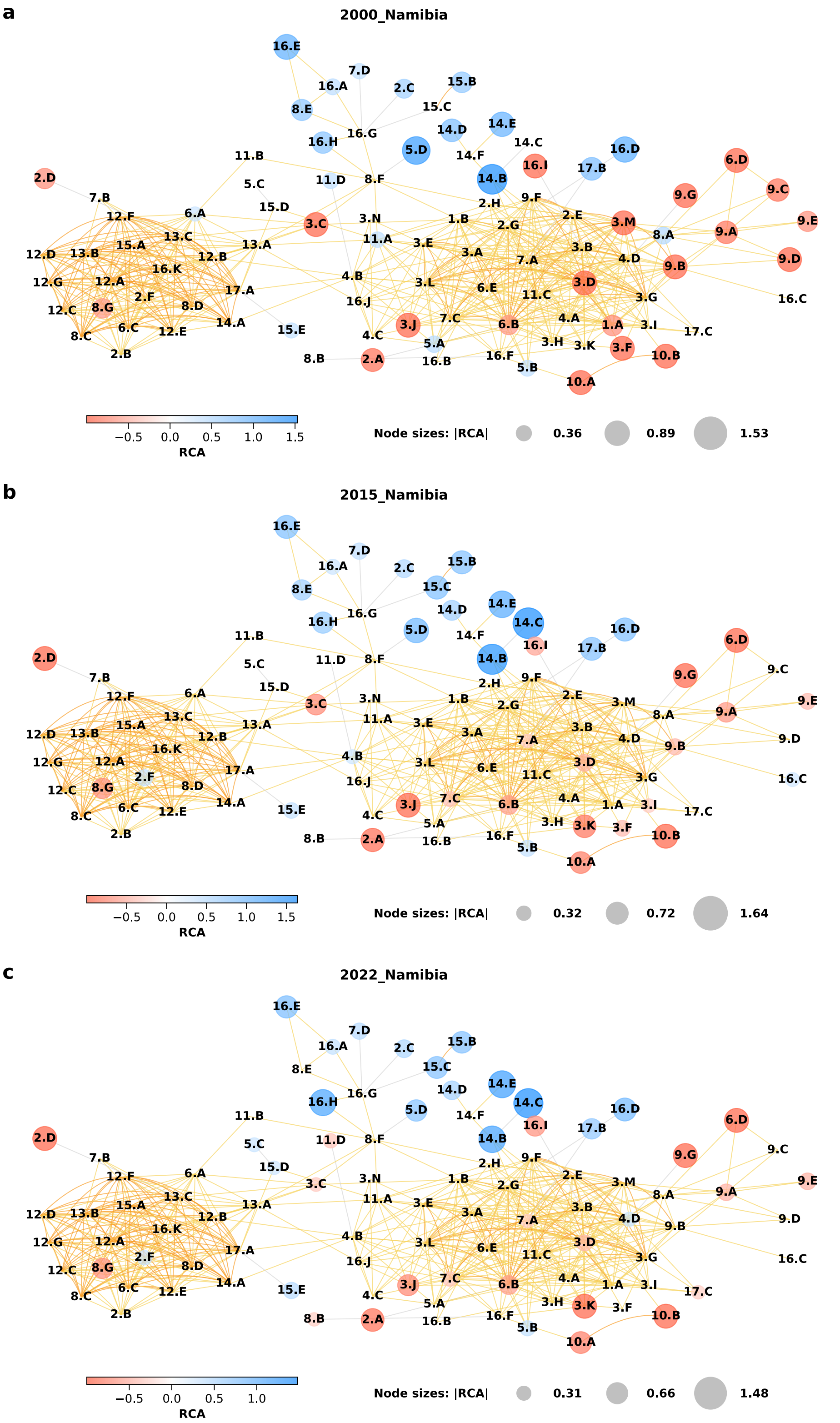

Supplementary Figure 103 | The SDG space of Namibia. Panels **a, b, c,** The SDG space in 2000, 2015, and 2022. The nodes in blue and orange represent the top 20 and bottom 20 SDG indicators in revealed comparative advantage (RCA) values, respectively. The node size represents the absolute value of RCA. From Supplementary Figure 12 to 177, countries are ranked by GDP/capita (current US$, 2022).

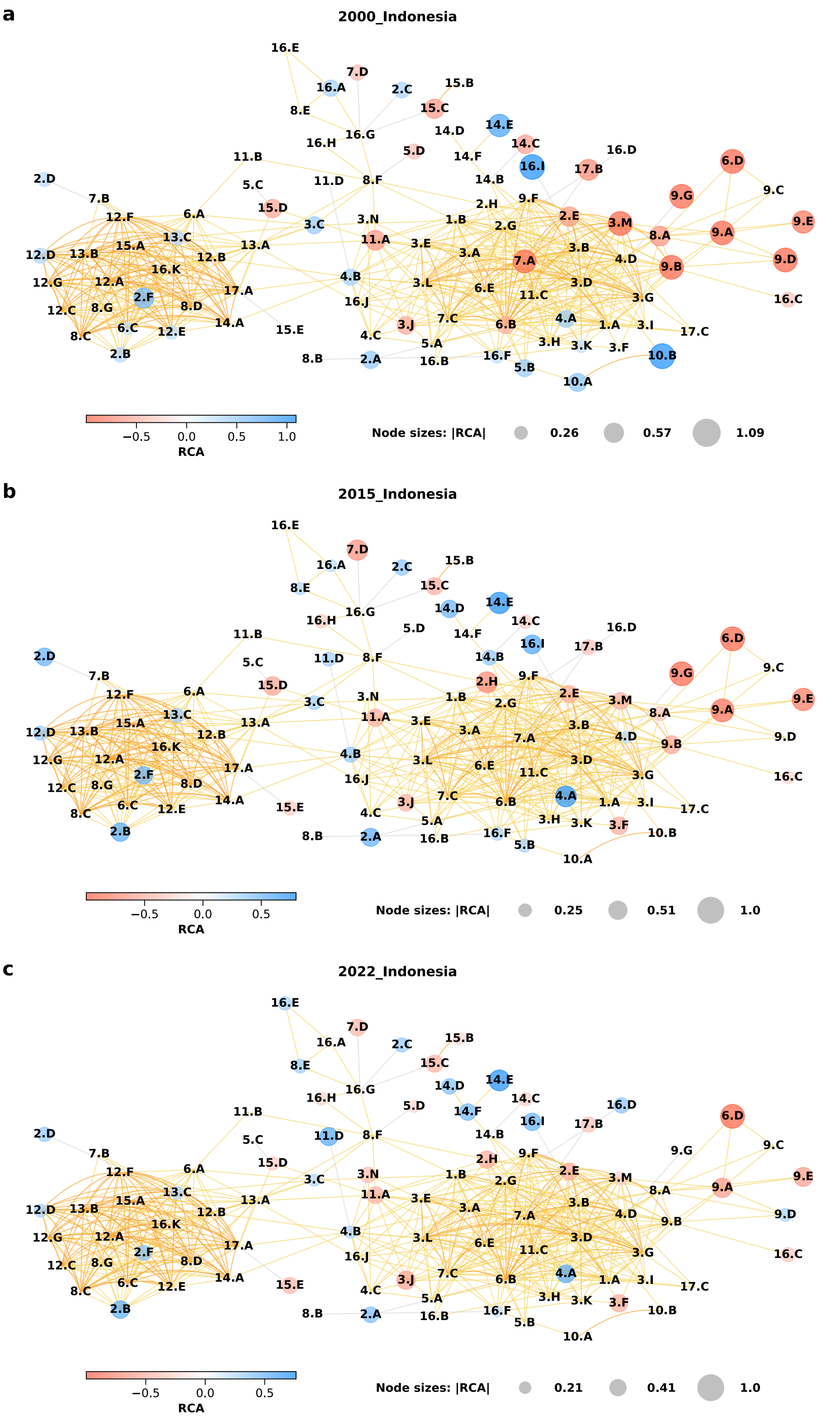

Supplementary Figure 104 | The SDG space of Indonesia. Panels **a, b, c,** The SDG space in 2000, 2015, and 2022. The nodes in blue and orange represent the top 20 and bottom 20 SDG indicators in revealed comparative advantage (RCA) values, respectively. The node size represents the absolute value of RCA. From Supplementary Figure 12 to 177, countries are ranked by GDP/capita (current US$, 2022).

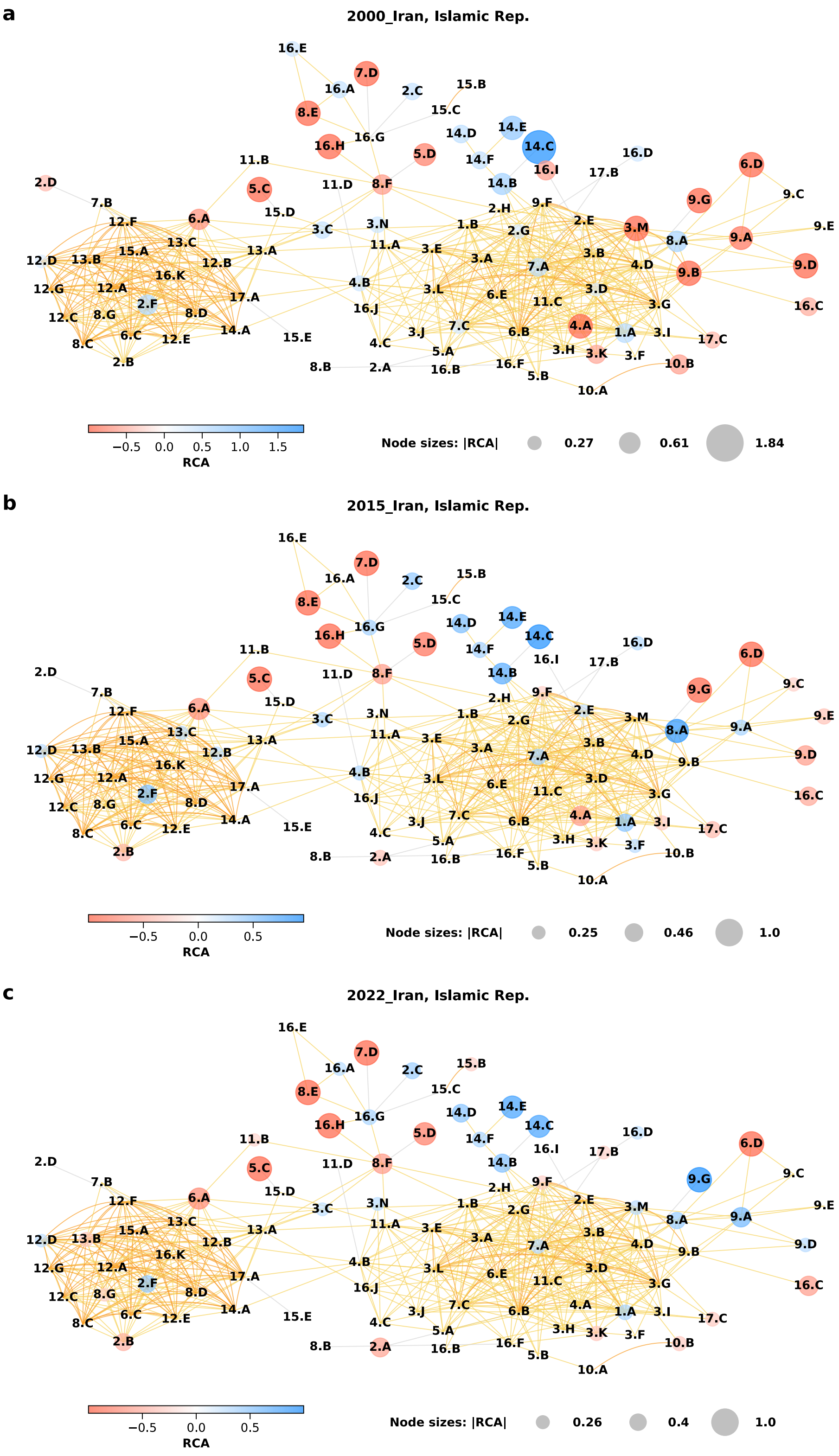

Supplementary Figure 105 | The SDG space of Iran. Panels **a, b, c,** The SDG space in 2000, 2015, and 2022. The nodes in blue and orange represent the top 20 and bottom 20 SDG indicators in revealed comparative advantage (RCA) values, respectively. The node size represents the absolute value of RCA. From Supplementary Figure 12 to 177, countries are ranked by GDP/capita (current US$, 2022).

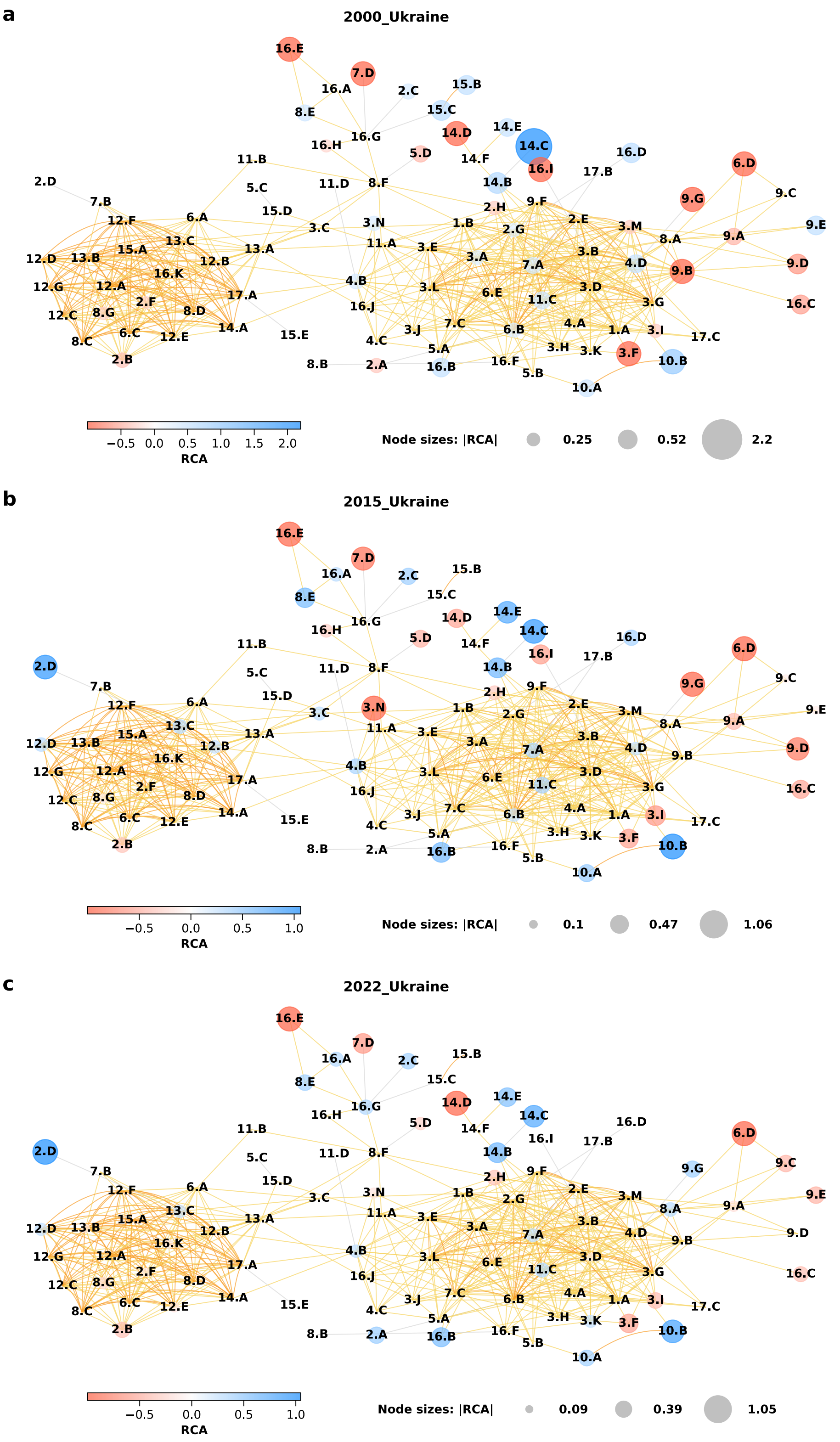

Supplementary Figure 106 | The SDG space of Ukraine. Panels **a, b, c,** The SDG space in 2000, 2015, and 2022. The nodes in blue and orange represent the top 20 and bottom 20 SDG indicators in revealed comparative advantage (RCA) values, respectively. The node size represents the absolute value of RCA. From Supplementary Figure 12 to 177, countries are ranked by GDP/capita (current US$, 2022).

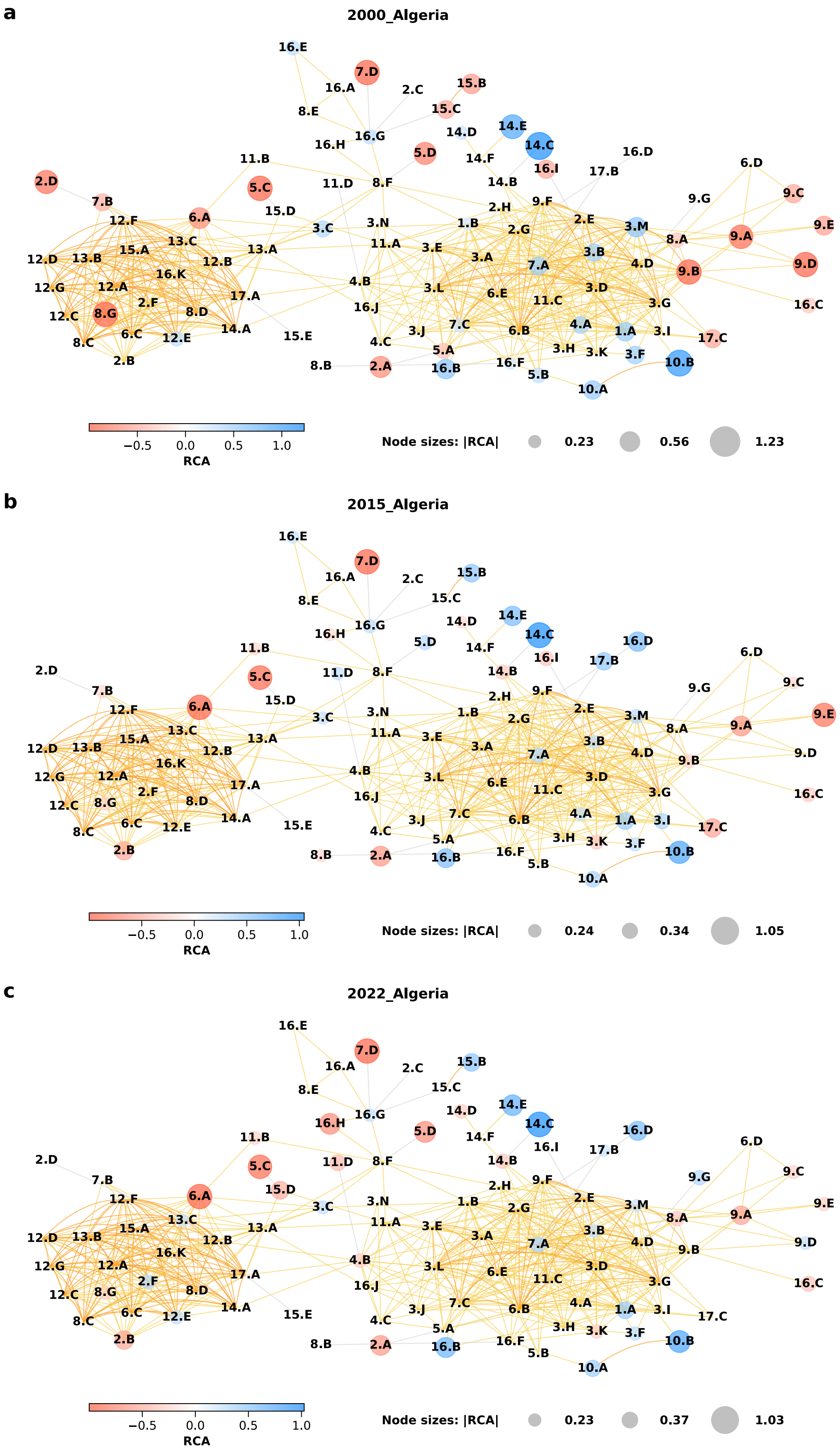

Supplementary Figure 107 | The SDG space of Algeria. Panels **a, b, c,** The SDG space in 2000, 2015, and 2022. The nodes in blue and orange represent the top 20 and bottom 20 SDG indicators in revealed comparative advantage (RCA) values, respectively. The node size represents the absolute value of RCA. From Supplementary Figure 12 to 177, countries are ranked by GDP/capita (current US$, 2022).

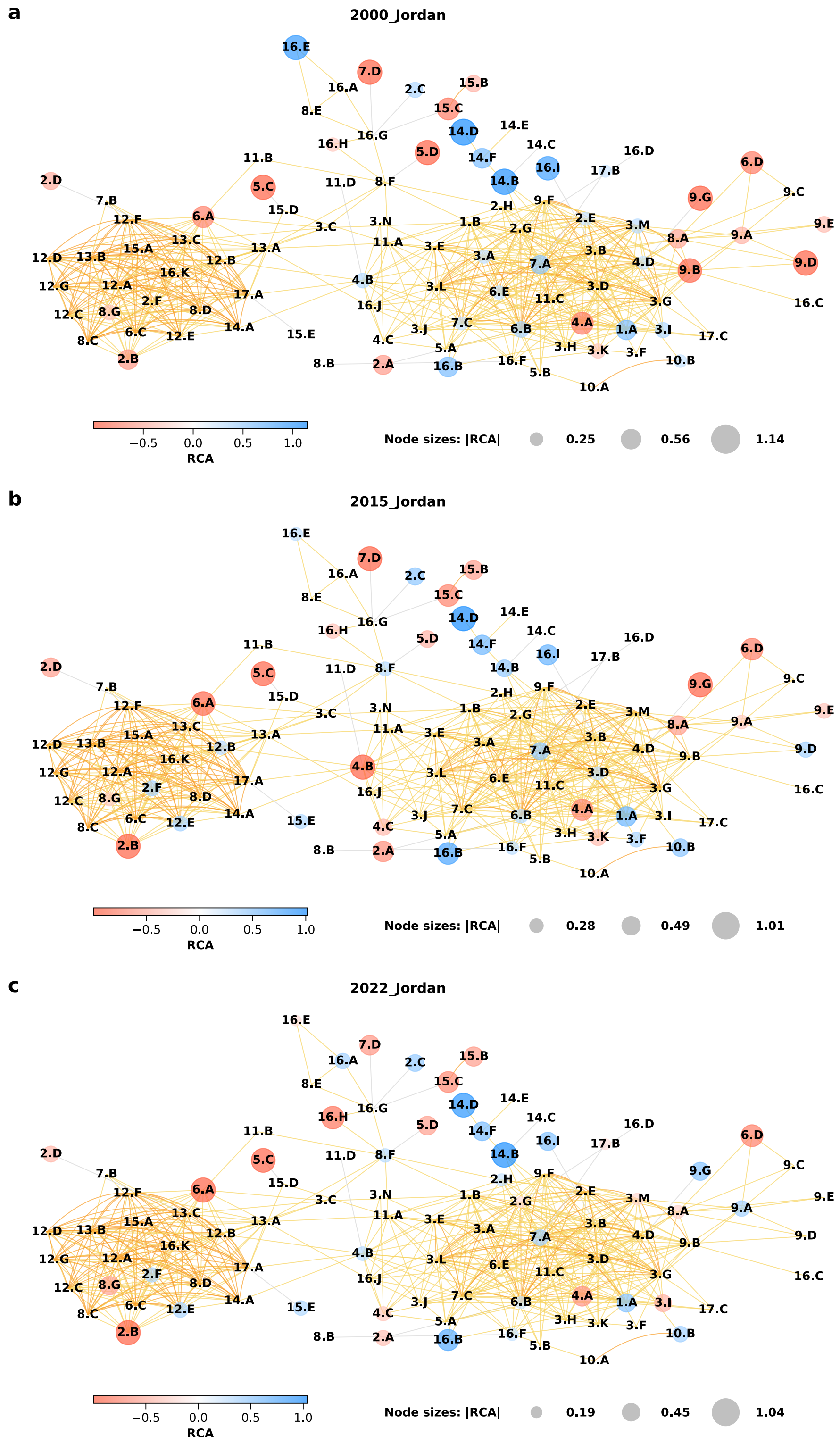

Supplementary Figure 108 | The SDG space of Jordan. Panels **a, b, c,** The SDG space in 2000, 2015, and 2022. The nodes in blue and orange represent the top 20 and bottom 20 SDG indicators in revealed comparative advantage (RCA) values, respectively. The node size represents the absolute value of RCA. From Supplementary Figure 12 to 177, countries are ranked by GDP/capita (current US$, 2022).

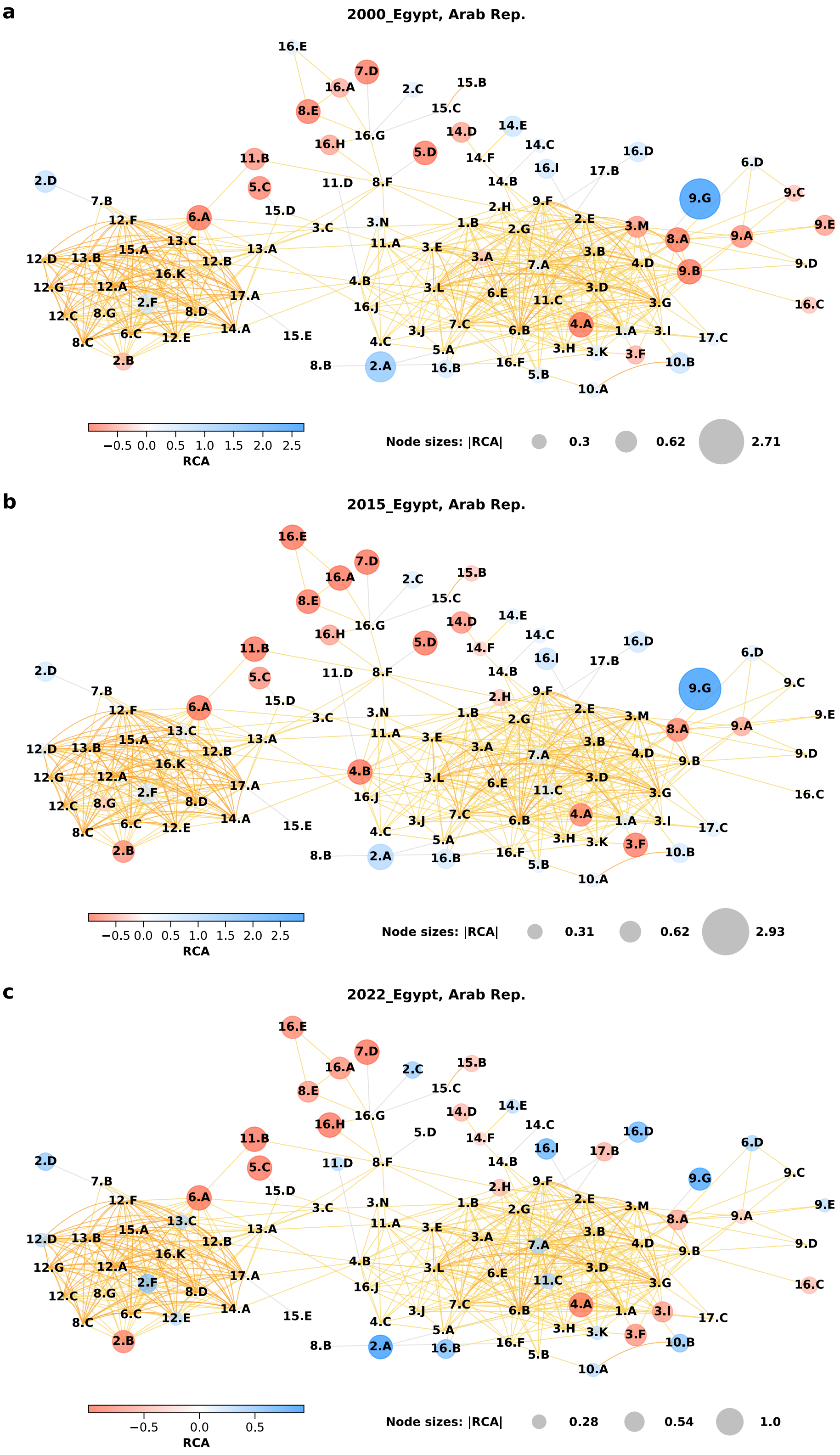

Supplementary Figure 109 | The SDG space of Egypt, Arab Rep.. Panels **a, b, c,** The SDG space in 2000, 2015, and 2022. The nodes in blue and orange represent the top 20 and bottom 20 SDG indicators in revealed comparative advantage (RCA) values, respectively. The node size represents the absolute value of RCA. From Supplementary Figure 12 to 177, countries are ranked by GDP/capita (current US$, 2022).

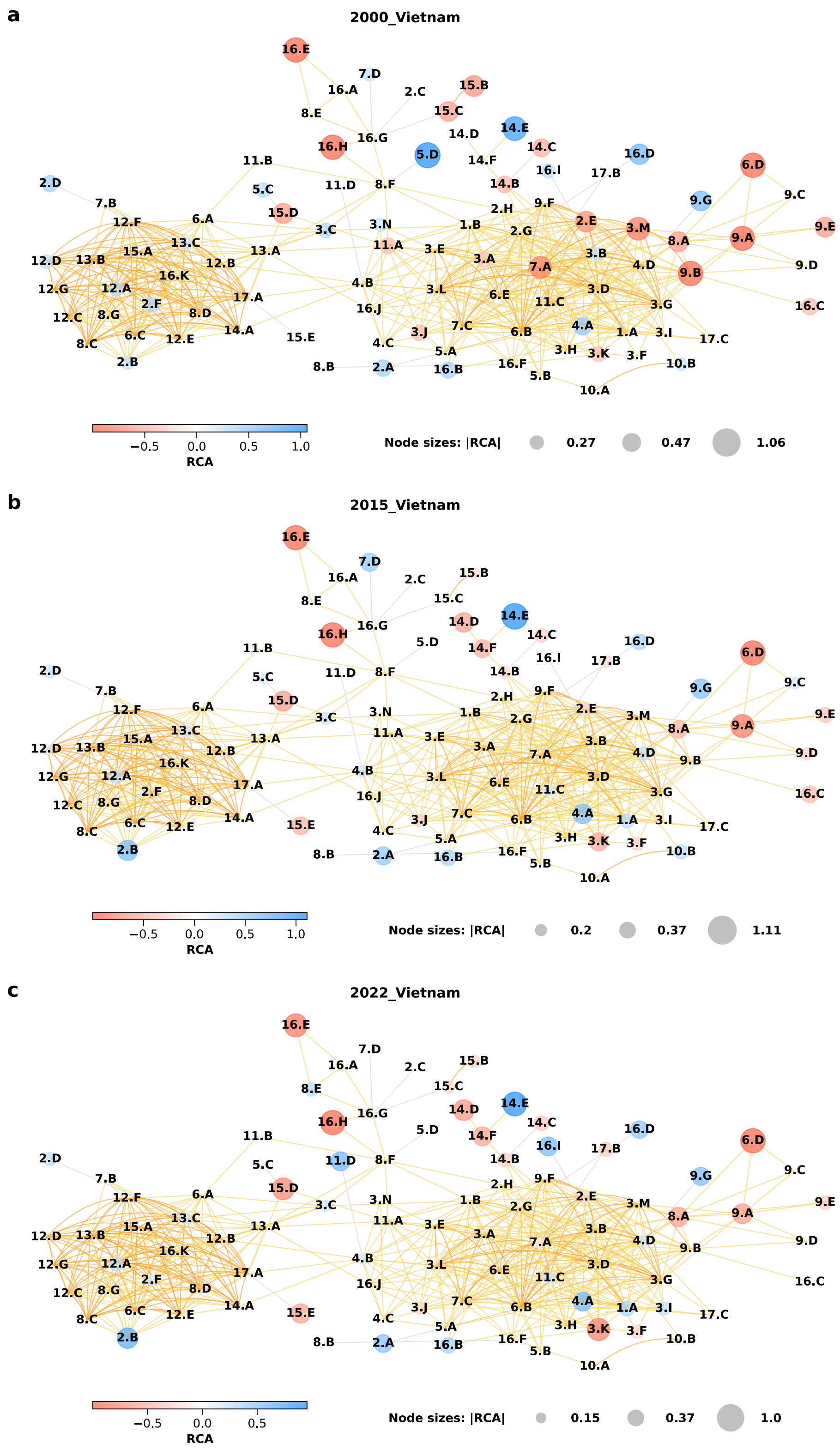

Supplementary Figure 110 | The SDG space of Vietnam. Panels **a, b, c,** The SDG space in 2000, 2015, and 2022. The nodes in blue and orange represent the top 20 and bottom 20 SDG indicators in revealed comparative advantage (RCA) values, respectively. The node size represents the absolute value of RCA. From Supplementary Figure 12 to 177, countries are ranked by GDP/capita (current US$, 2022).

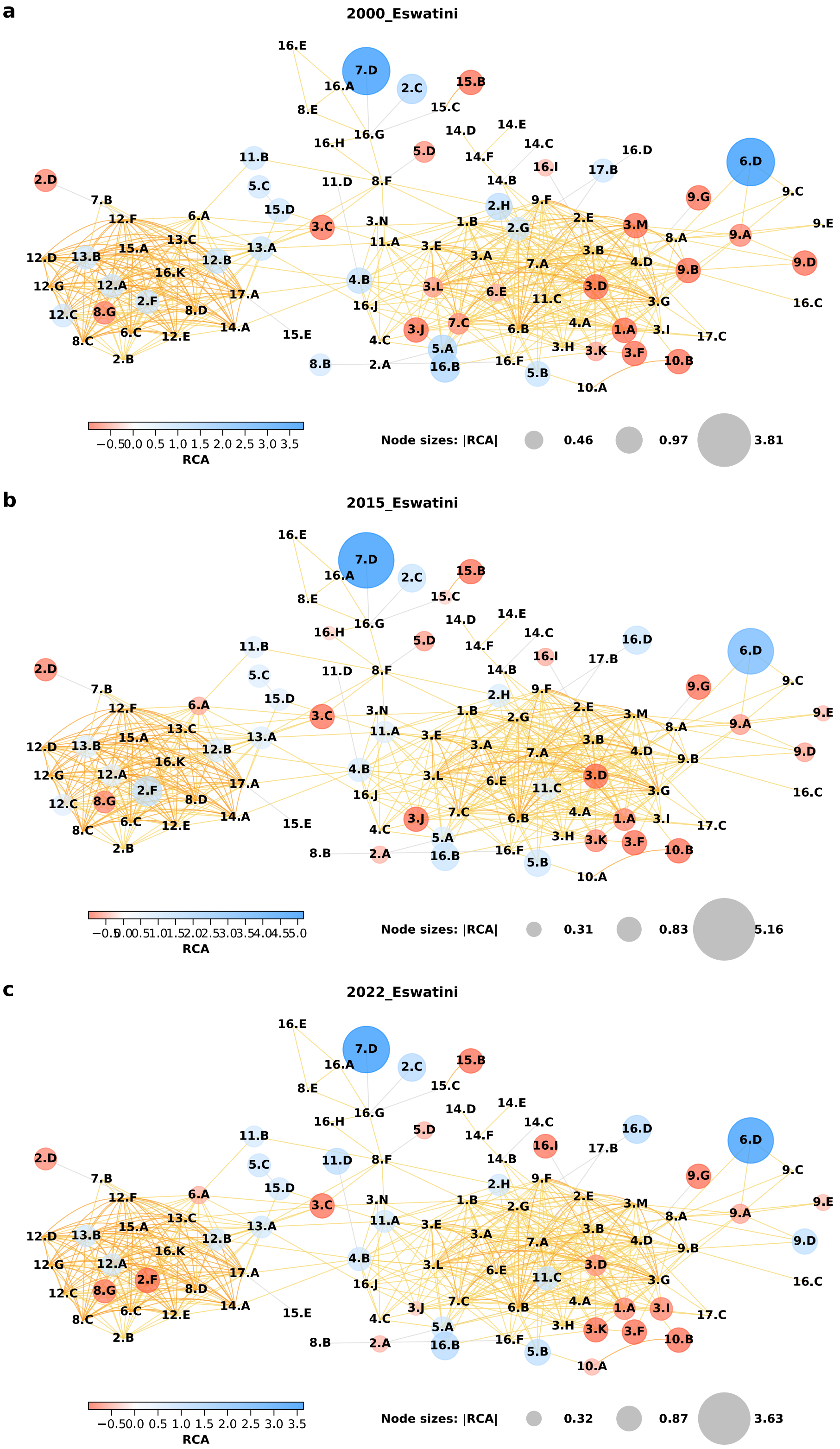

Supplementary Figure 111 | The SDG space of Eswatini. Panels **a, b, c,** The SDG space in 2000, 2015, and 2022. The nodes in blue and orange represent the top 20 and bottom 20 SDG indicators in revealed comparative advantage (RCA) values, respectively. The node size represents the absolute value of RCA. From Supplementary Figure 12 to 177, countries are ranked by GDP/capita (current US$, 2022).

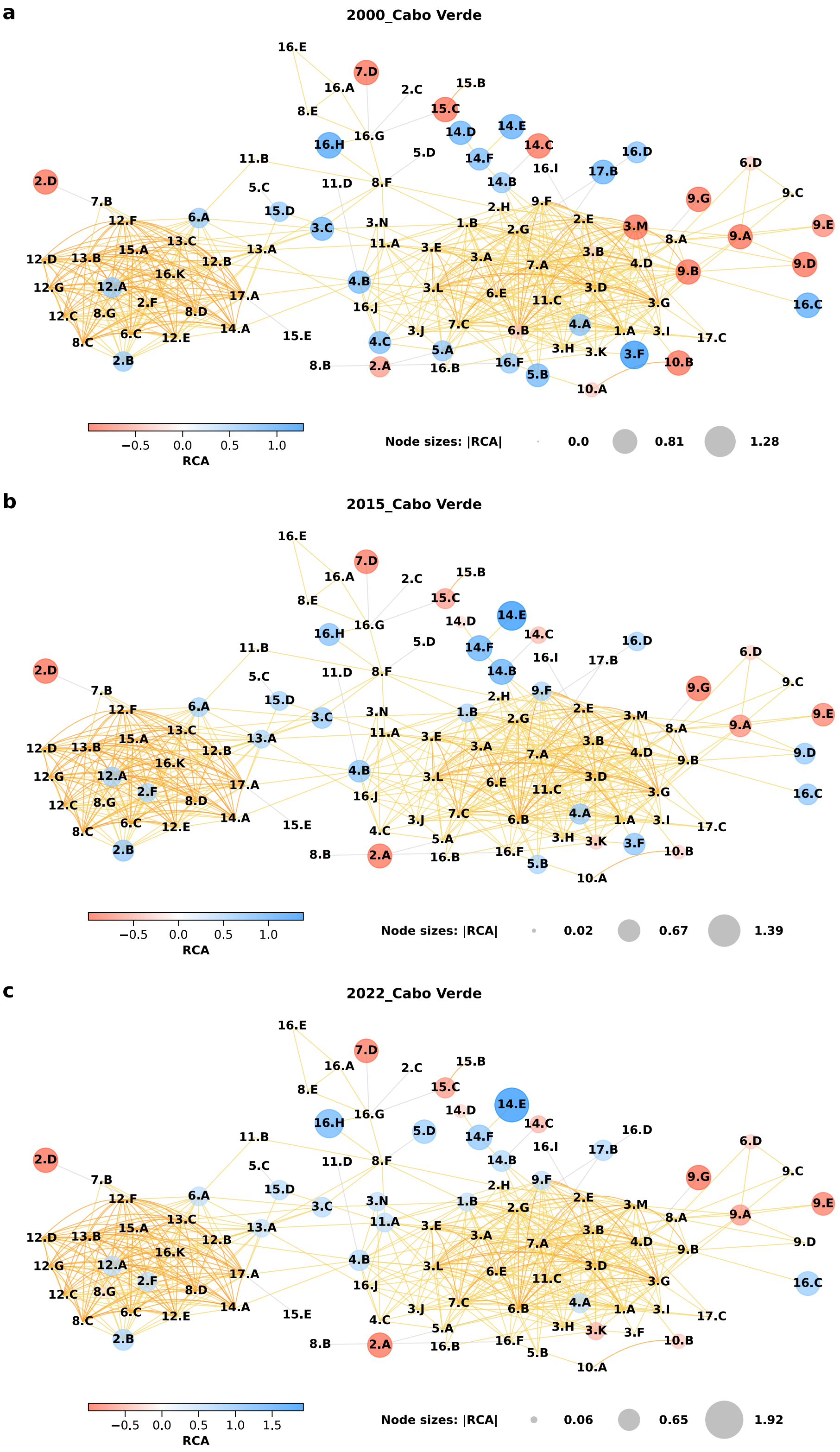

Supplementary Figure 112 | The SDG space of Cabo Verde. Panels **a, b, c,** The SDG space in 2000, 2015, and 2022. The nodes in blue and orange represent the top 20 and bottom 20 SDG indicators in revealed comparative advantage (RCA) values, respectively. The node size represents the absolute value of RCA. From Supplementary Figure 12 to 177, countries are ranked by GDP/capita (current US$, 2022).

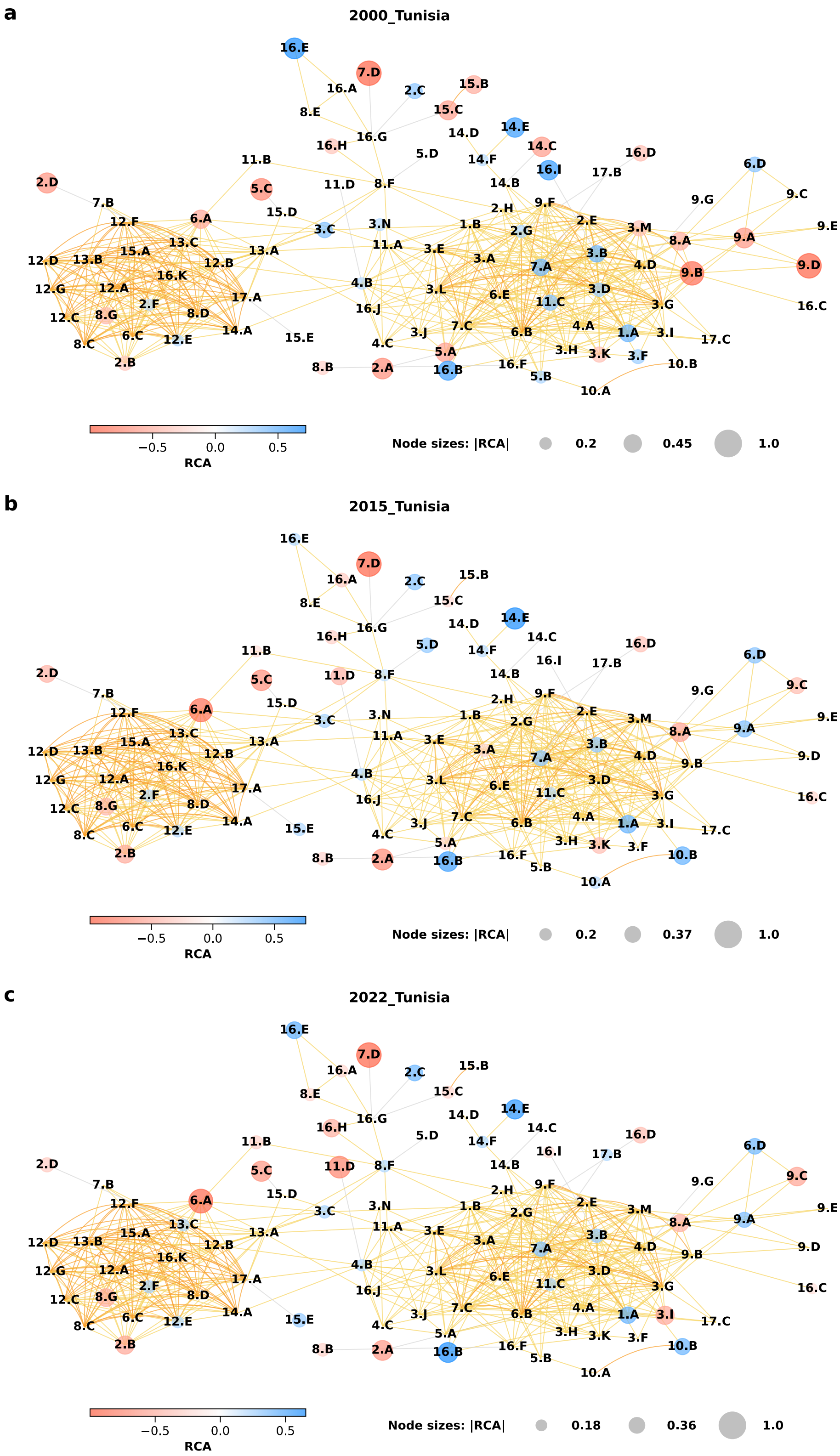

Supplementary Figure 113 | The SDG space of Tunisia. Panels **a, b, c,** The SDG space in 2000, 2015, and 2022. The nodes in blue and orange represent the top 20 and bottom 20 SDG indicators in revealed comparative advantage (RCA) values, respectively. The node size represents the absolute value of RCA. From Supplementary Figure 12 to 177, countries are ranked by GDP/capita (current US$, 2022).

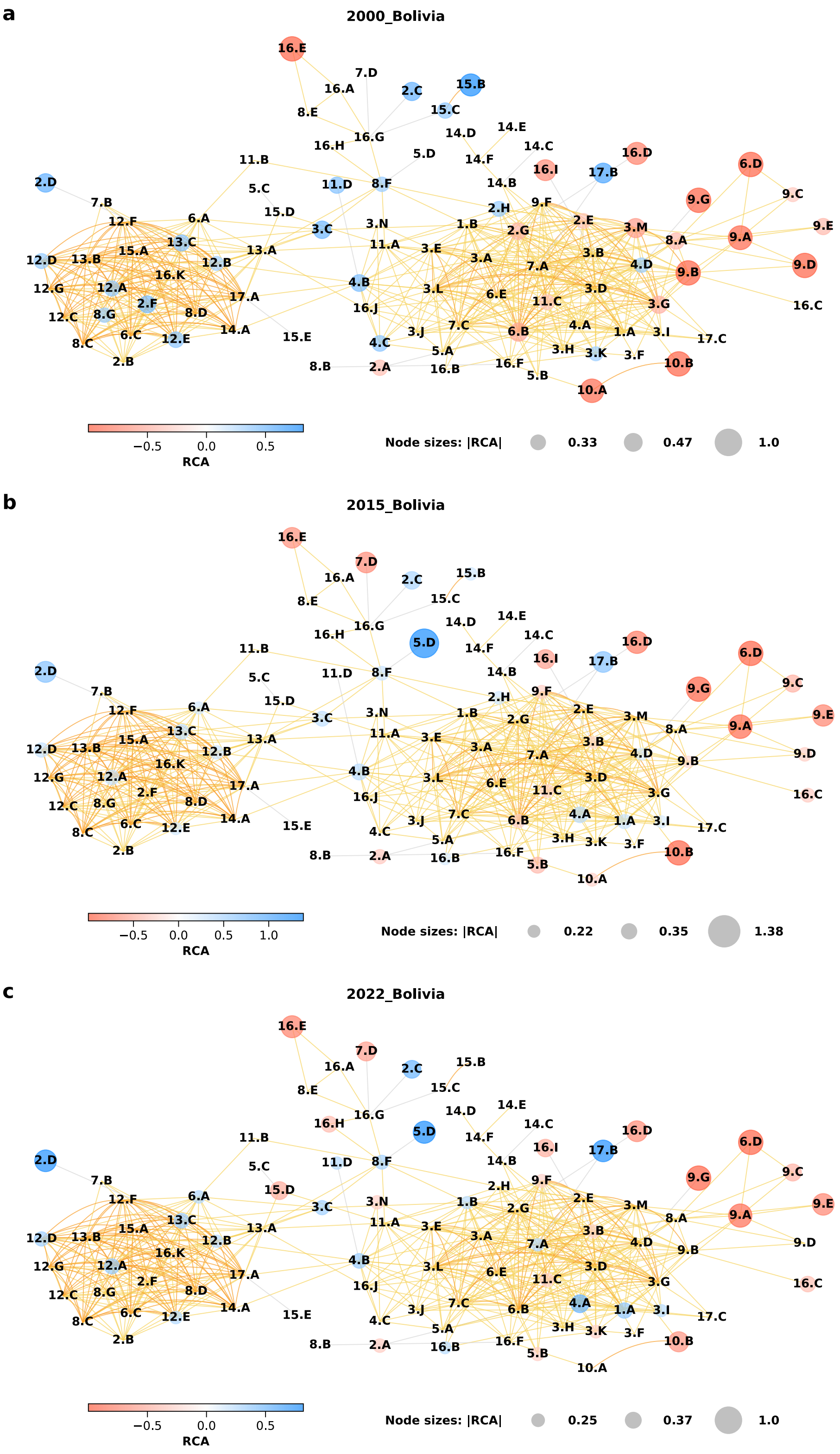

Supplementary Figure 114 | The SDG space of Bolivia. Panels **a, b, c,** The SDG space in 2000, 2015, and 2022. The nodes in blue and orange represent the top 20 and bottom 20 SDG indicators in revealed comparative advantage (RCA) values, respectively. The node size represents the absolute value of RCA. From Supplementary Figure 12 to 177, countries are ranked by GDP/capita (current US$, 2022).

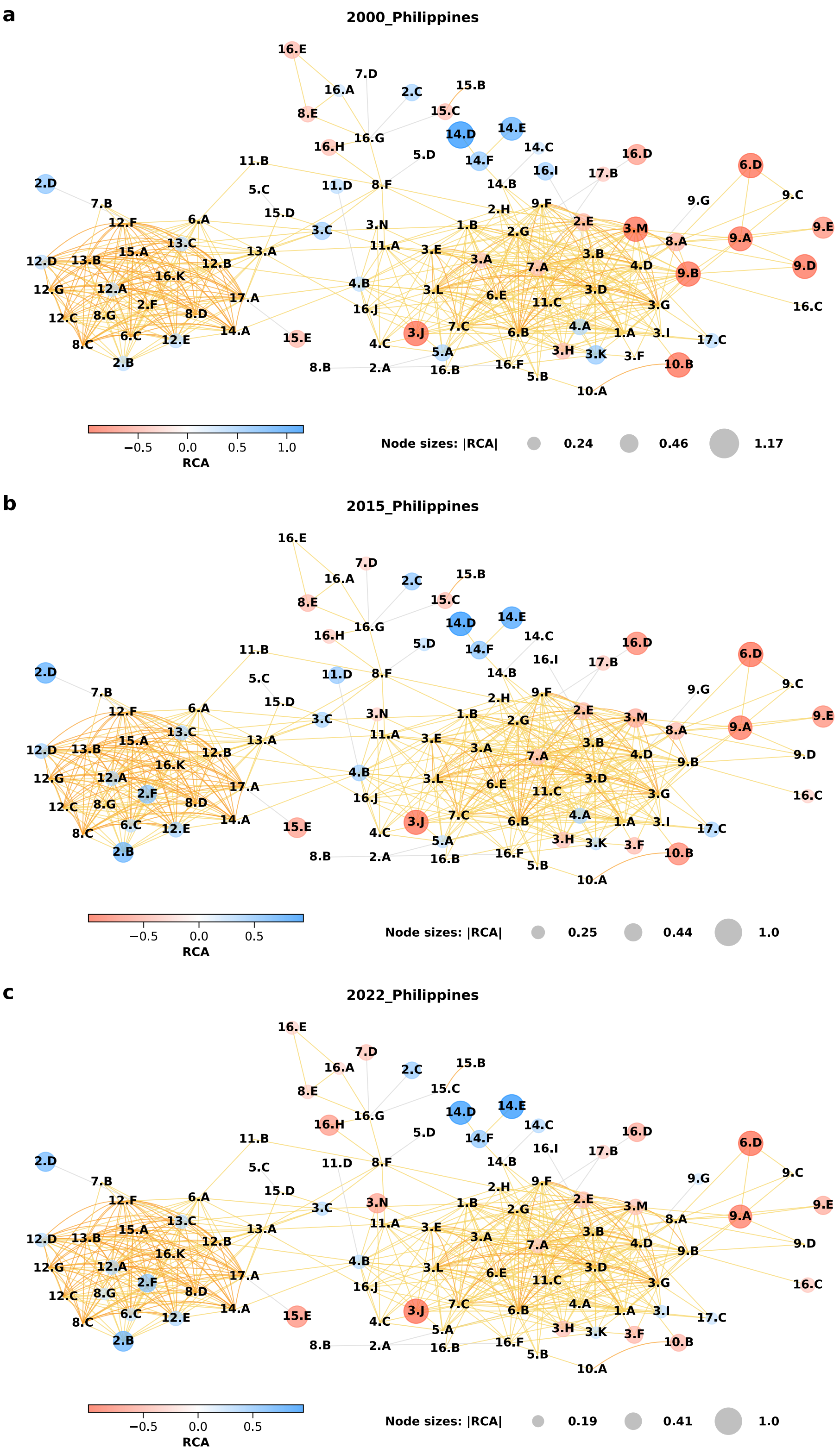

Supplementary Figure 115 | The SDG space of Philippines. Panels **a, b, c,** The SDG space in 2000, 2015, and 2022. The nodes in blue and orange represent the top 20 and bottom 20 SDG indicators in revealed comparative advantage (RCA) values, respectively. The node size represents the absolute value of RCA. From Supplementary Figure 12 to 177, countries are ranked by GDP/capita (current US$, 2022).

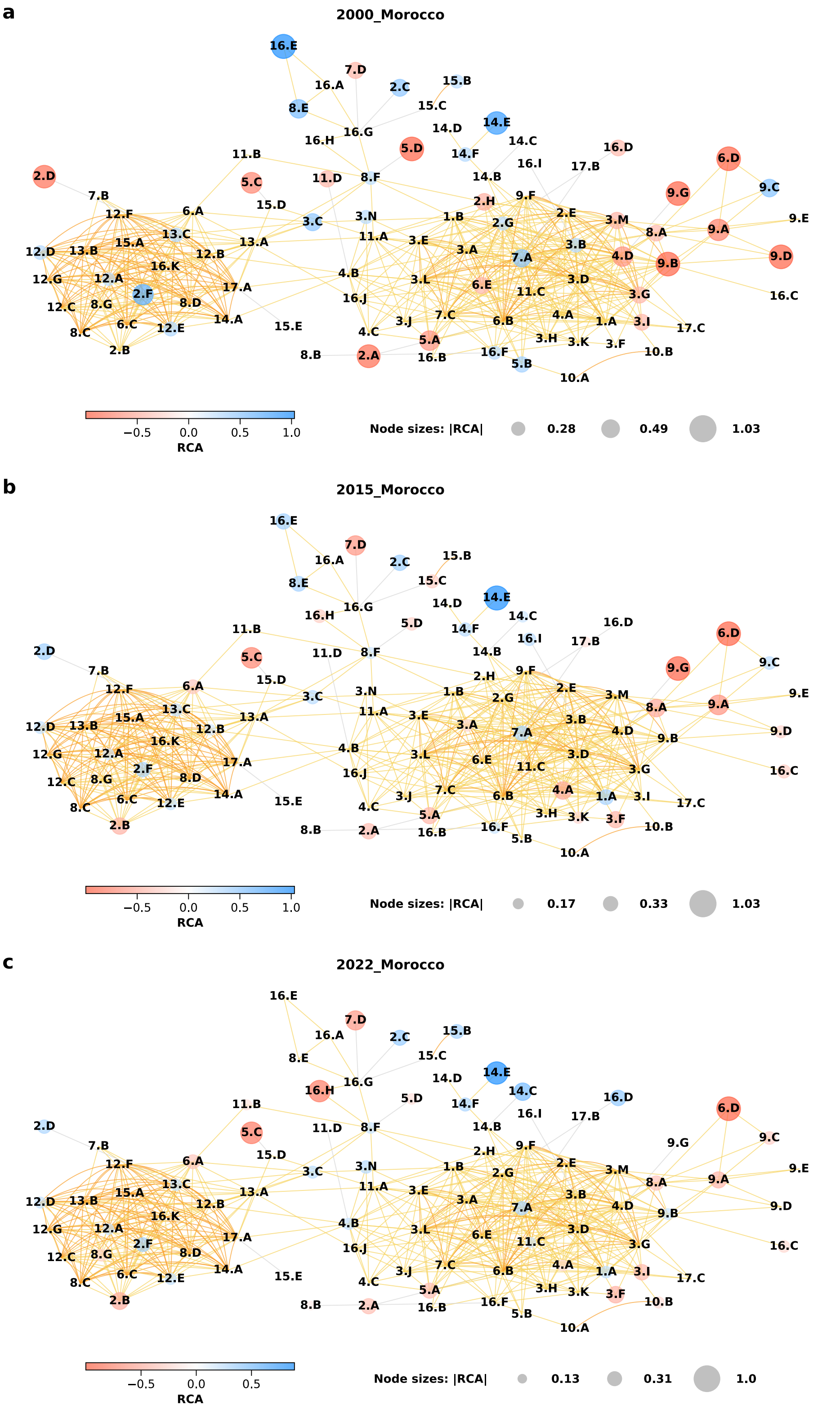

Supplementary Figure 116 | The SDG space of Morocco. Panels **a, b, c,** The SDG space in 2000, 2015, and 2022. The nodes in blue and orange represent the top 20 and bottom 20 SDG indicators in revealed comparative advantage (RCA) values, respectively. The node size represents the absolute value of RCA. From Supplementary Figure 12 to 177, countries are ranked by GDP/capita (current US$, 2022).

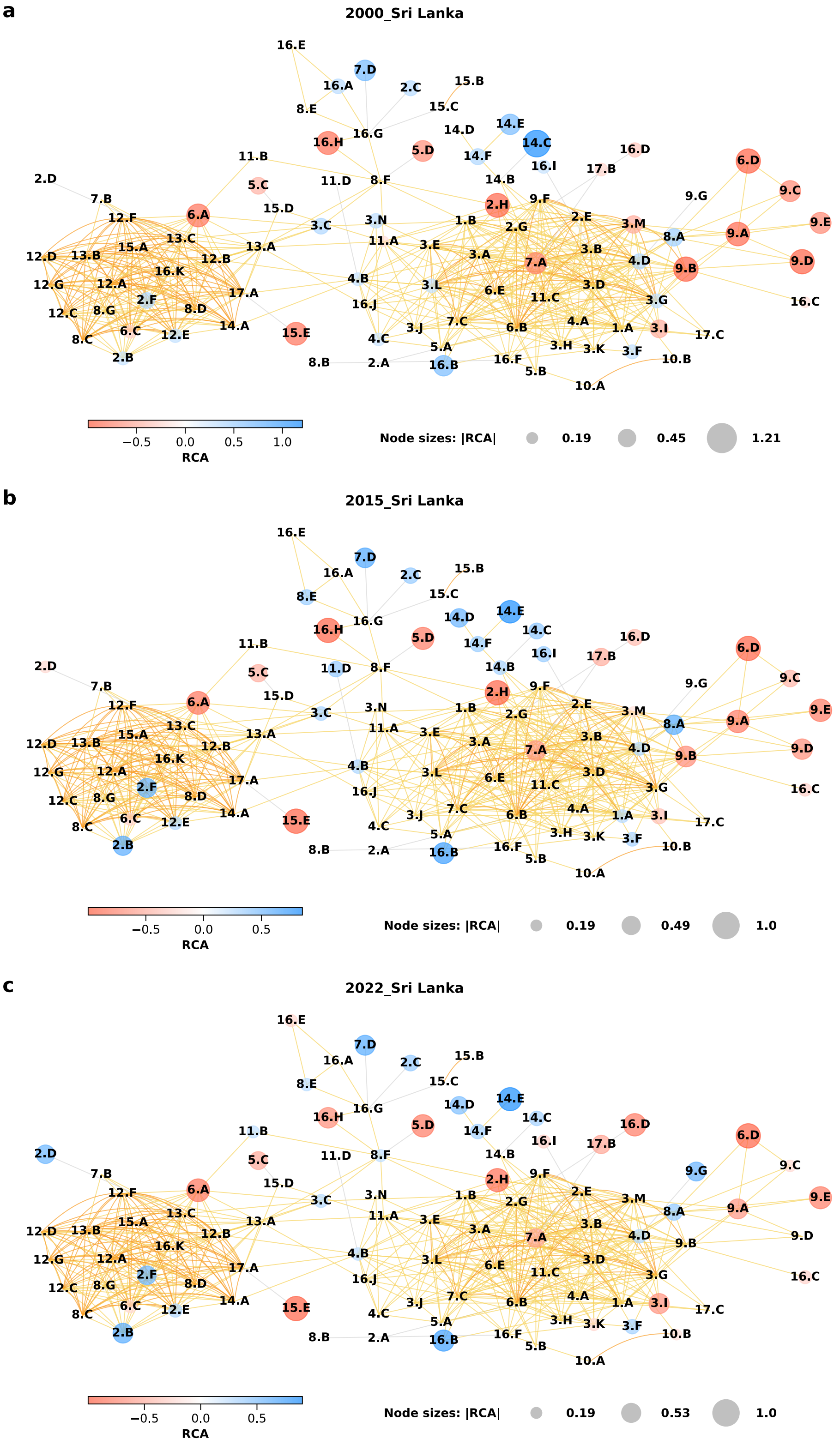

Supplementary Figure 117 | The SDG space of Sri Lanka. Panels **a, b, c,** The SDG space in 2000, 2015, and 2022. The nodes in blue and orange represent the top 20 and bottom 20 SDG indicators in revealed comparative advantage (RCA) values, respectively. The node size represents the absolute value of RCA. From Supplementary Figure 12 to 177, countries are ranked by GDP/capita (current US$, 2022).

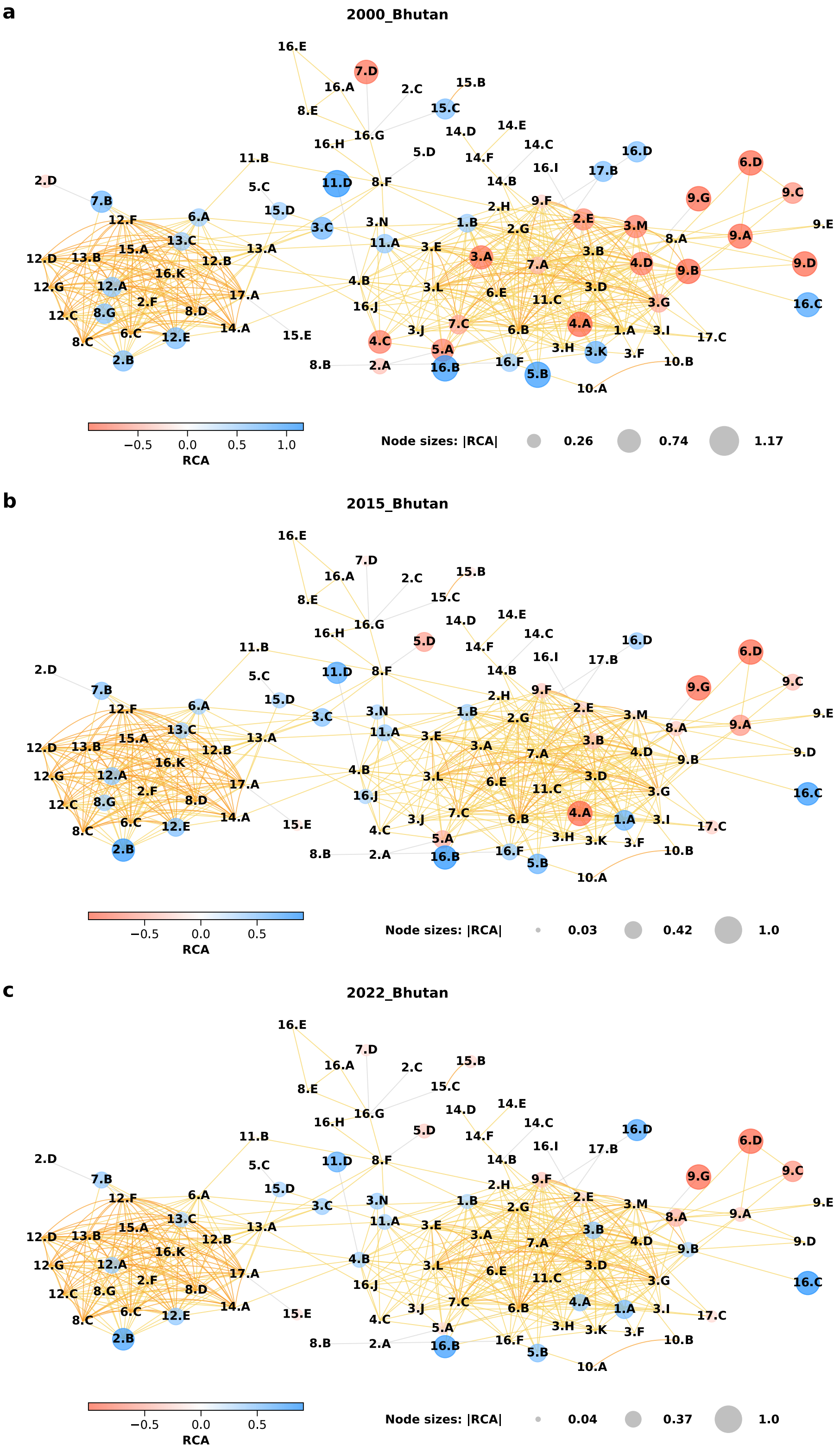

Supplementary Figure 118 | The SDG space of Bhutan. Panels **a, b, c,** The SDG space in 2000, 2015, and 2022. The nodes in blue and orange represent the top 20 and bottom 20 SDG indicators in revealed comparative advantage (RCA) values, respectively. The node size represents the absolute value of RCA. From Supplementary Figure 12 to 177, countries are ranked by GDP/capita (current US$, 2022).

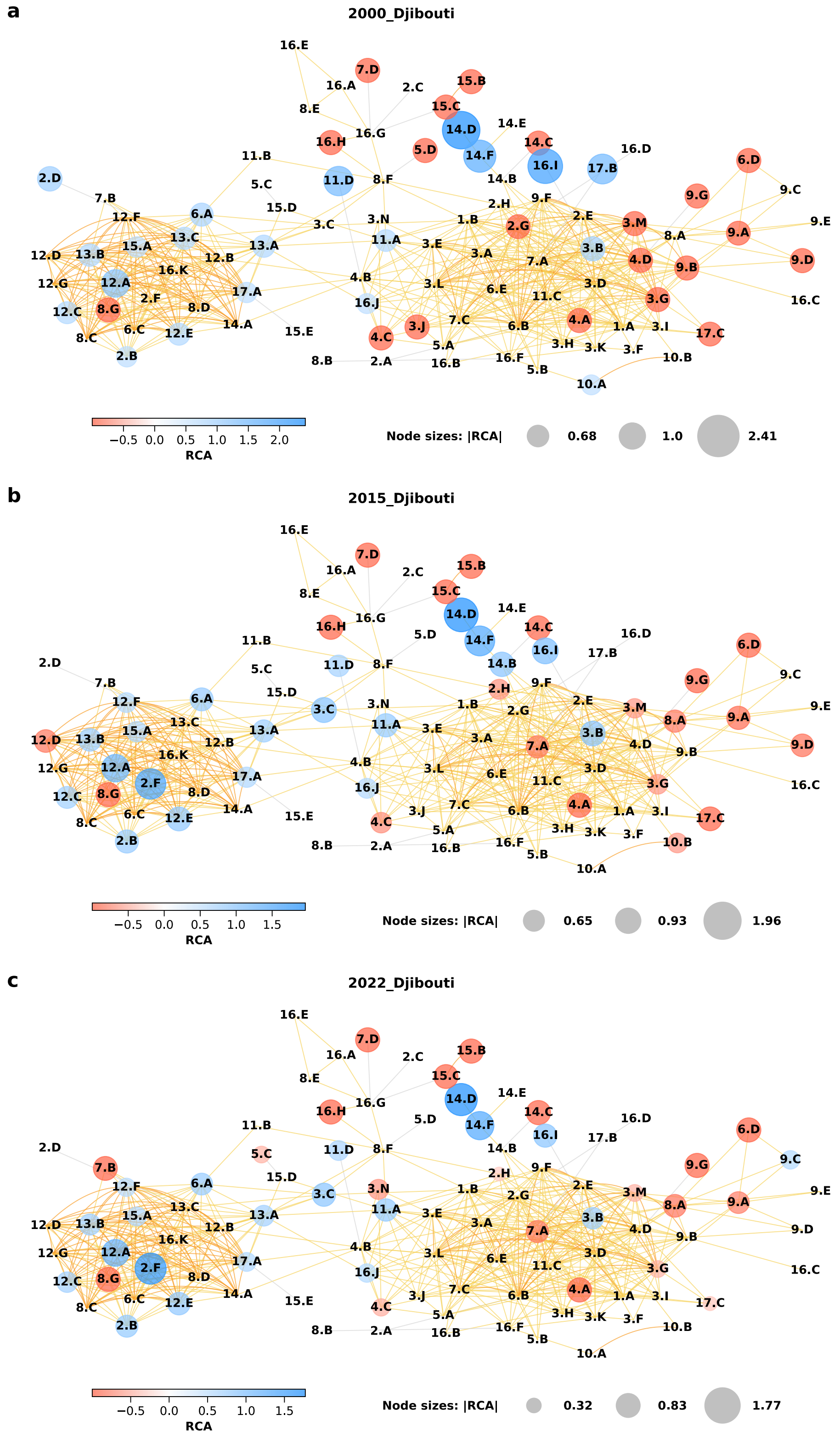

Supplementary Figure 119 | The SDG space of Djibouti. Panels **a, b, c,** The SDG space in 2000, 2015, and 2022. The nodes in blue and orange represent the top 20 and bottom 20 SDG indicators in revealed comparative advantage (RCA) values, respectively. The node size represents the absolute value of RCA. From Supplementary Figure 12 to 177, countries are ranked by GDP/capita (current US$, 2022).

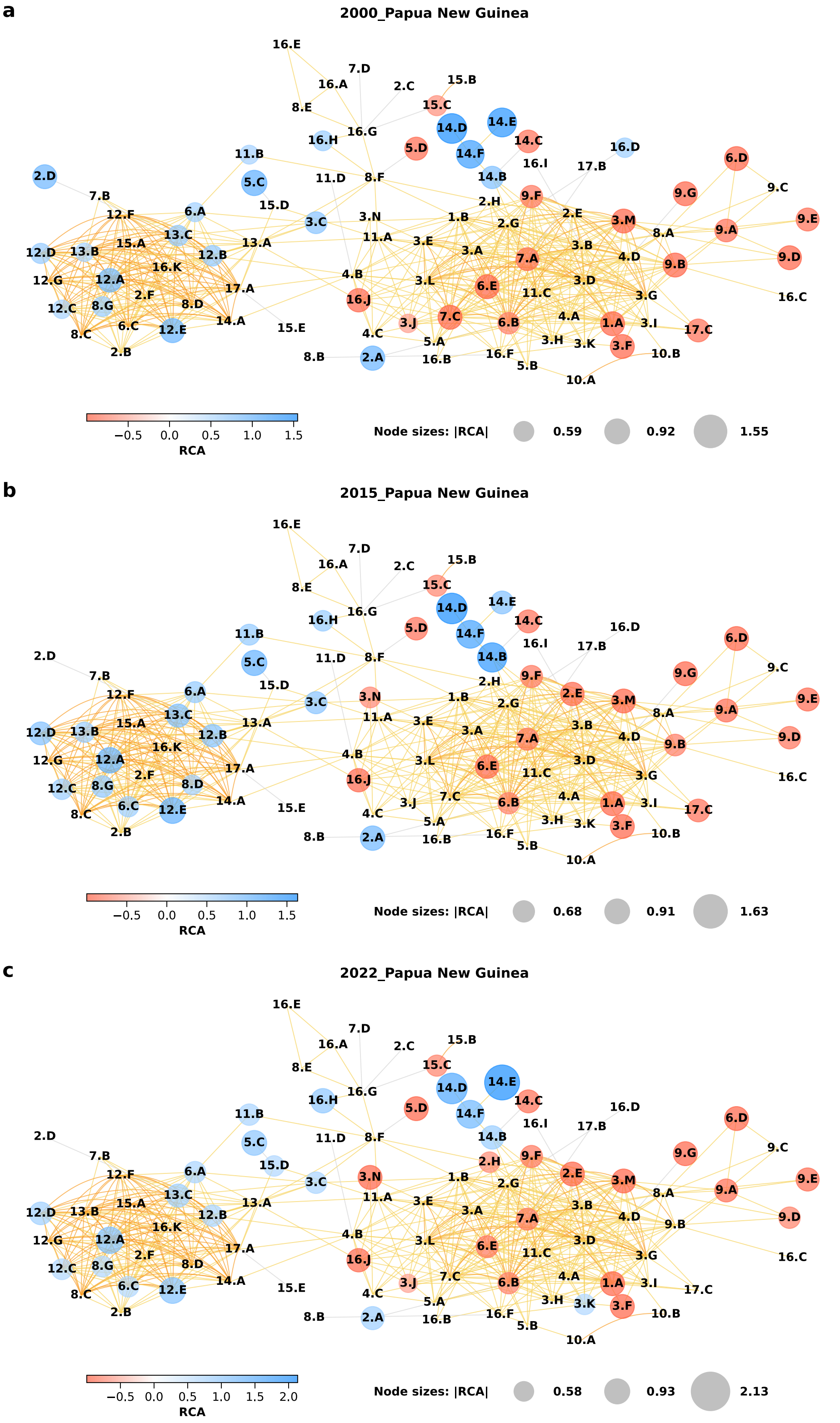

Supplementary Figure 120 | The SDG space of Papua New Guinea. Panels **a, b, c,** The SDG space in 2000, 2015, and 2022. The nodes in blue and orange represent the top 20 and bottom 20 SDG indicators in revealed comparative advantage (RCA) values, respectively. The node size represents the absolute value of RCA. From Supplementary Figure 12 to 177, countries are ranked by GDP/capita (current US$, 2022).

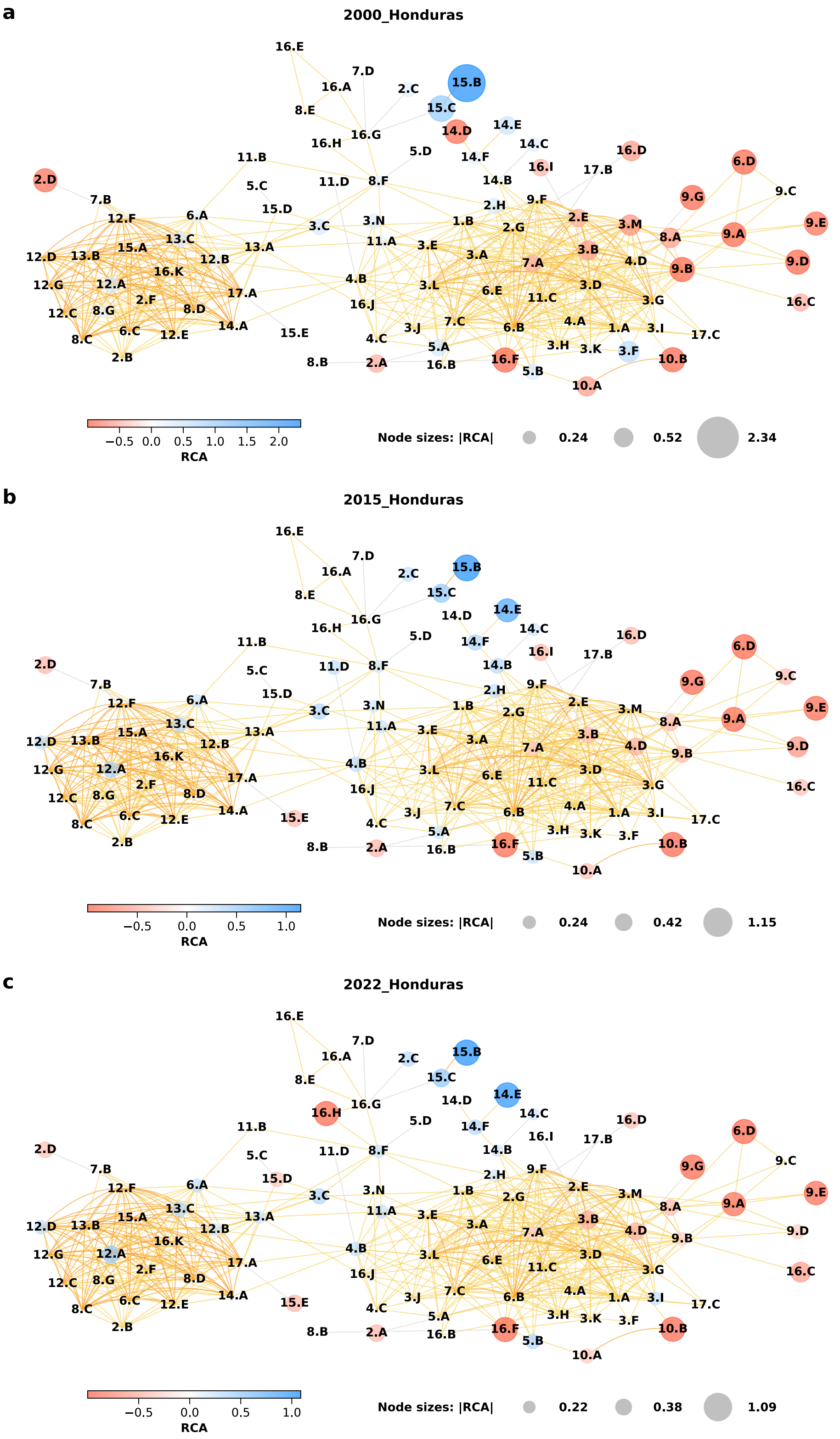

Supplementary Figure 121 | The SDG space of Honduras. Panels **a, b, c,** The SDG space in 2000, 2015, and 2022. The nodes in blue and orange represent the top 20 and bottom 20 SDG indicators in revealed comparative advantage (RCA) values, respectively. The node size represents the absolute value of RCA. From Supplementary Figure 12 to 177, countries are ranked by GDP/capita (current US$, 2022).

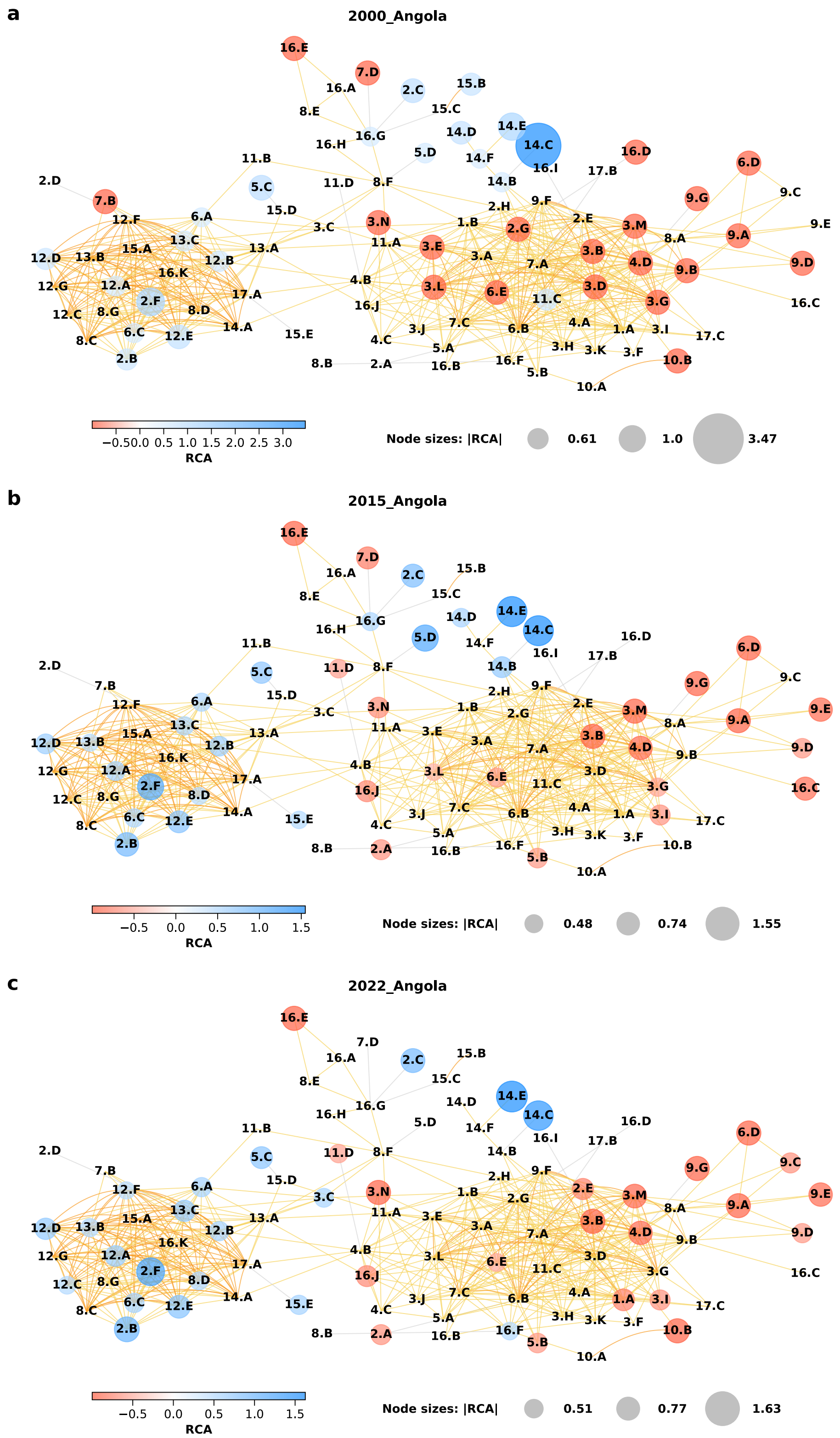

Supplementary Figure 122 | The SDG space of Angola. Panels **a, b, c,** The SDG space in 2000, 2015, and 2022. The nodes in blue and orange represent the top 20 and bottom 20 SDG indicators in revealed comparative advantage (RCA) values, respectively. The node size represents the absolute value of RCA. From Supplementary Figure 12 to 177, countries are ranked by GDP/capita (current US$, 2022).

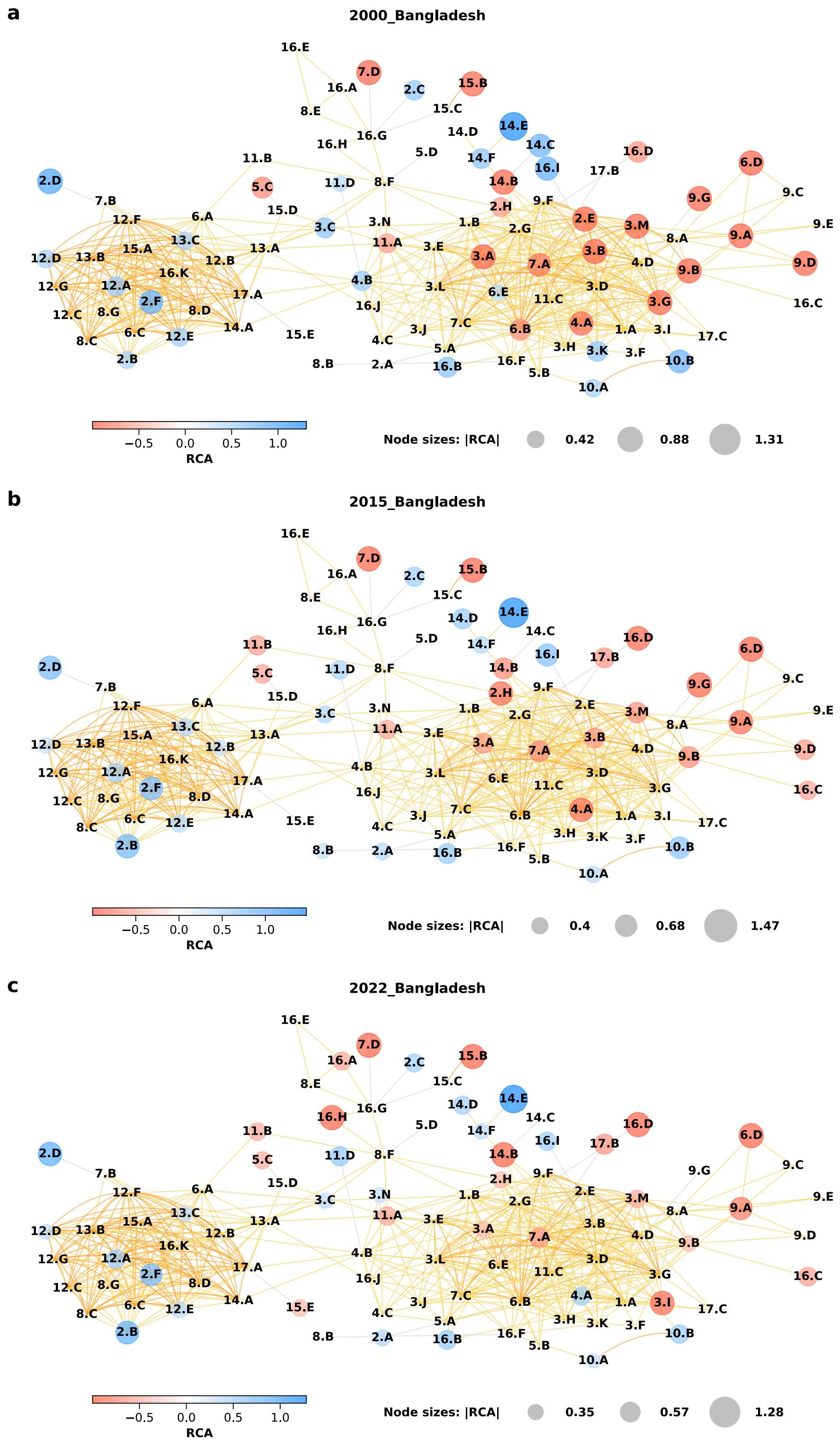

Supplementary Figure 123 | The SDG space of Bangladesh. Panels **a, b, c,** The SDG space in 2000, 2015, and 2022. The nodes in blue and orange represent the top 20 and bottom 20 SDG indicators in revealed comparative advantage (RCA) values, respectively. The node size represents the absolute value of RCA. From Supplementary Figure 12 to 177, countries are ranked by GDP/capita (current US$, 2022).

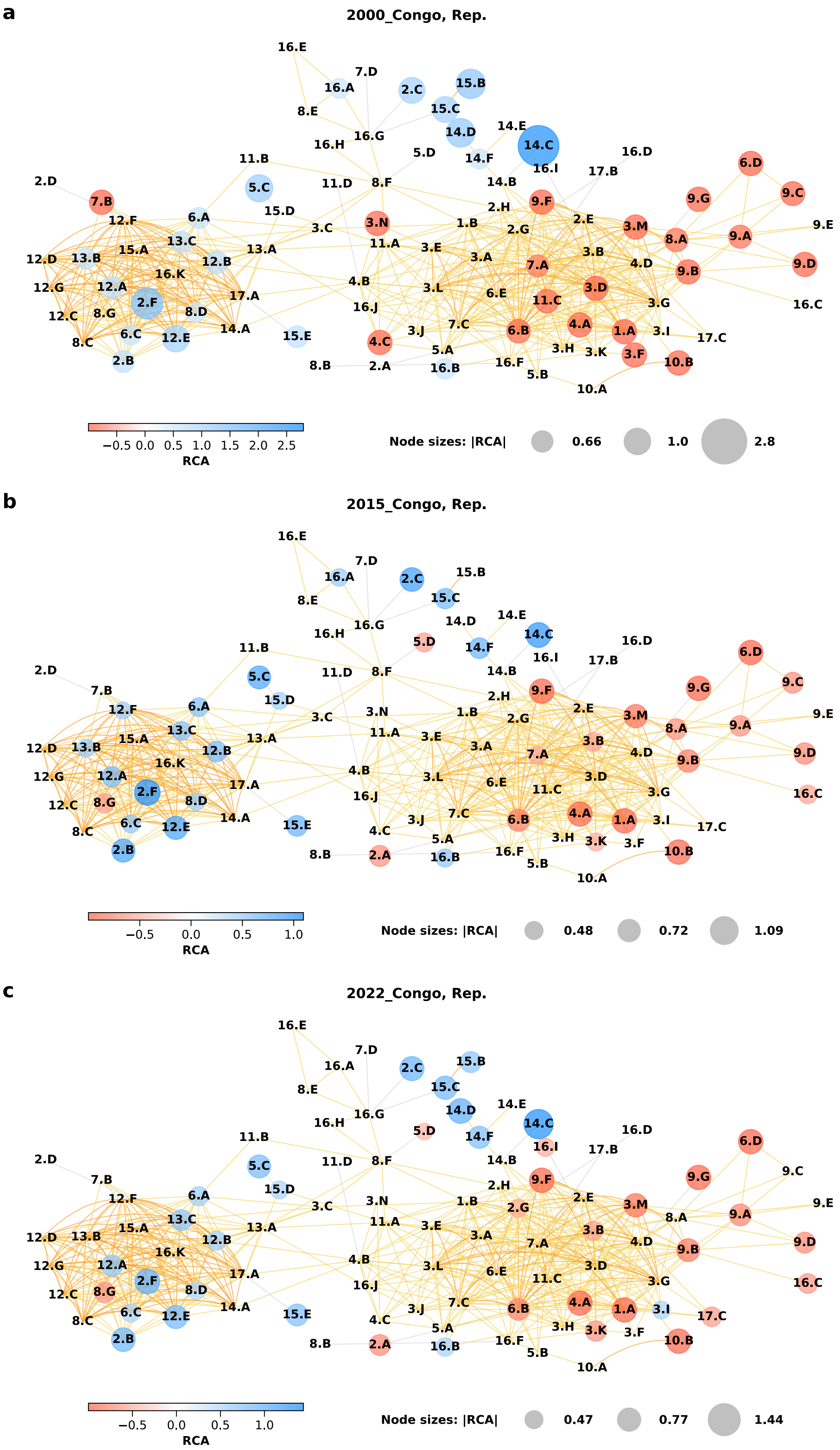

Supplementary Figure 124 | The SDG space of Congo, Rep.. Panels **a, b, c,** The SDG space in 2000, 2015, and 2022. The nodes in blue and orange represent the top 20 and bottom 20 SDG indicators in revealed comparative advantage (RCA) values, respectively. The node size represents the absolute value of RCA. From Supplementary Figure 12 to 177, countries are ranked by GDP/capita (current US$, 2022).

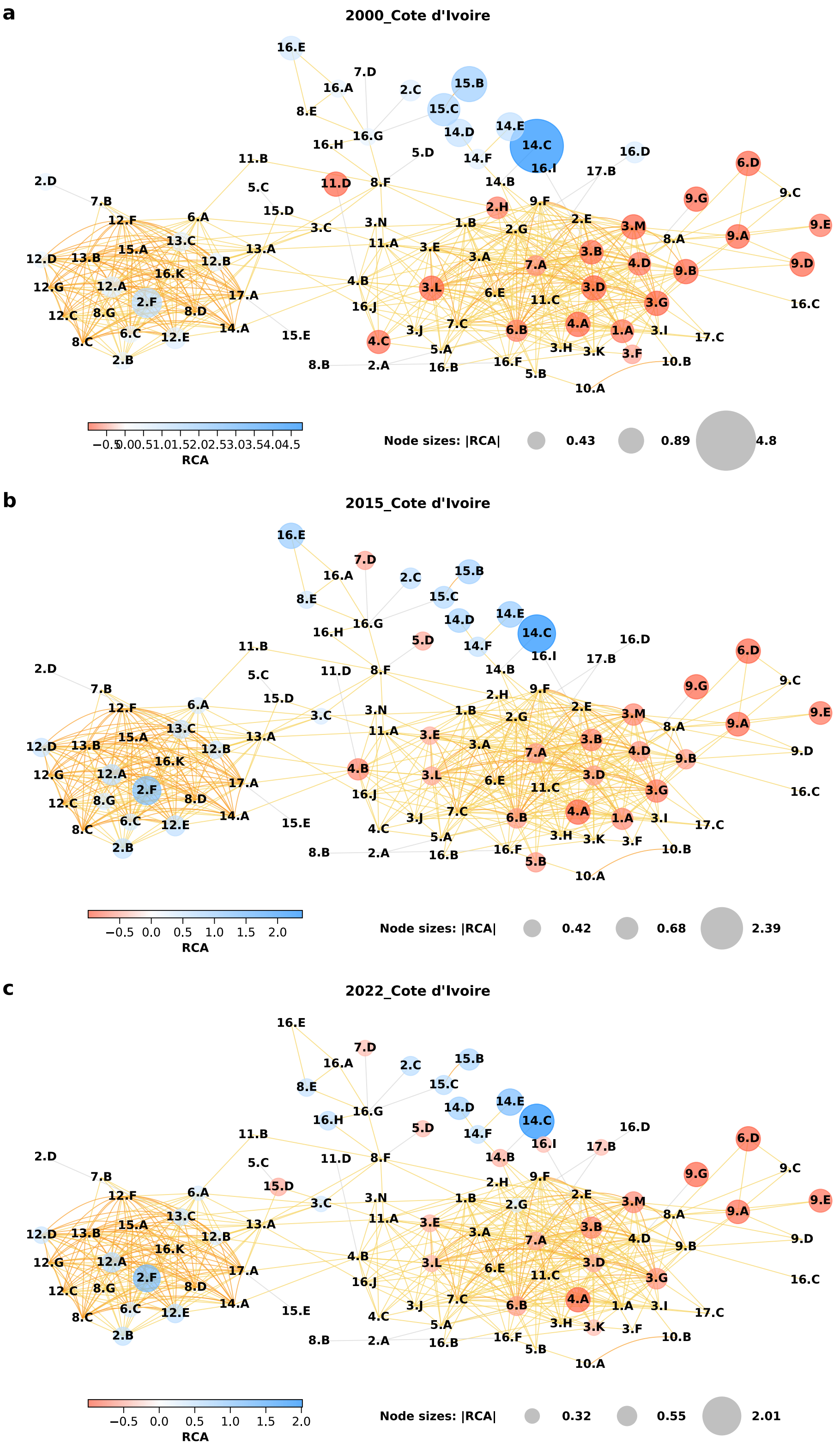

Supplementary Figure 125 | The SDG space of Cote d'Ivoire. Panels **a, b, c,** The SDG space in 2000, 2015, and 2022. The nodes in blue and orange represent the top 20 and bottom 20 SDG indicators in revealed comparative advantage (RCA) values, respectively. The node size represents the absolute value of RCA. From Supplementary Figure 12 to 177, countries are ranked by GDP/capita (current US$, 2022).

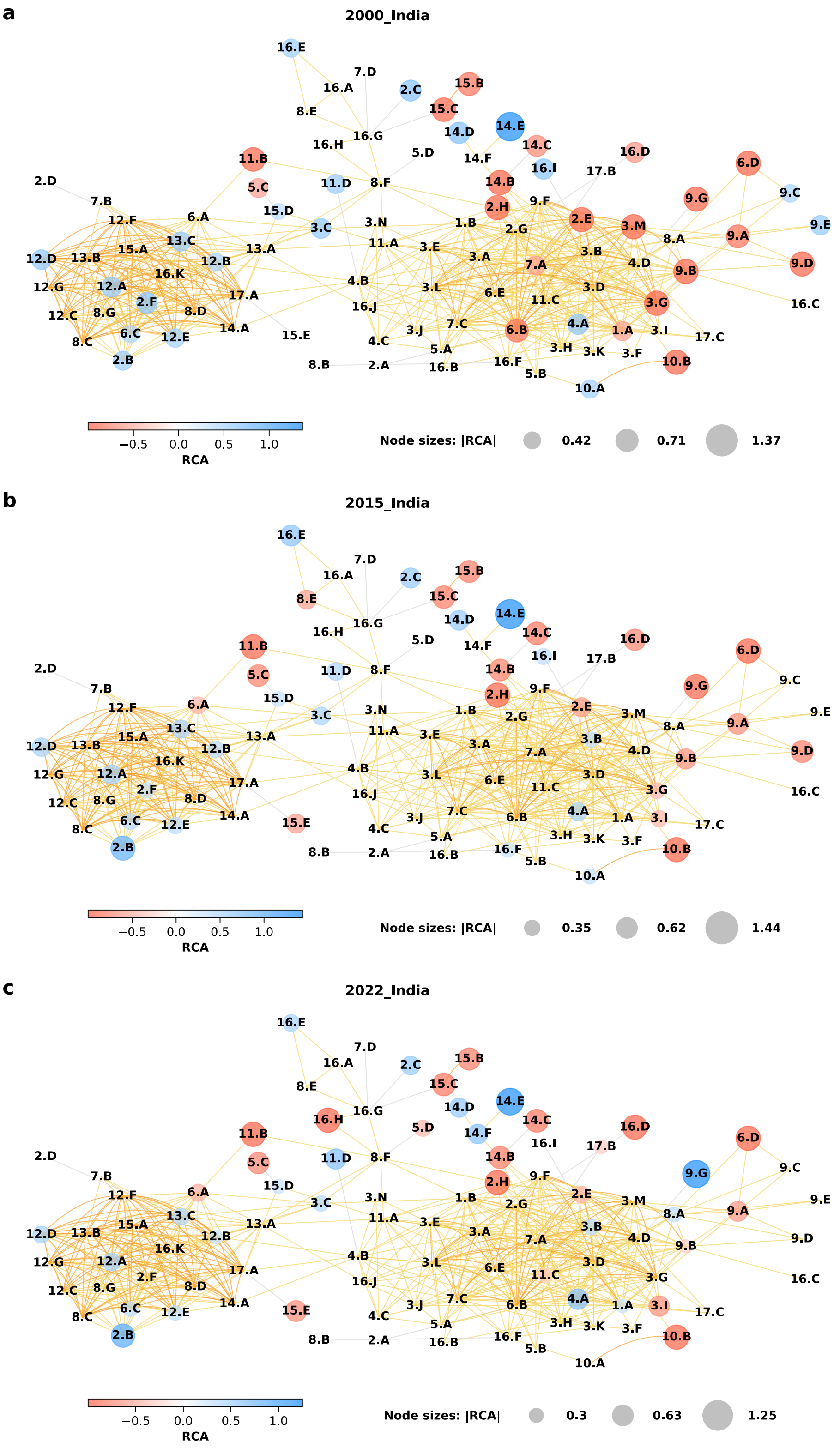

Supplementary Figure 126 | The SDG space of India. Panels **a, b, c,** The SDG space in 2000, 2015, and 2022. The nodes in blue and orange represent the top 20 and bottom 20 SDG indicators in revealed comparative advantage (RCA) values, respectively. The node size represents the absolute value of RCA. From Supplementary Figure 12 to 177, countries are ranked by GDP/capita (current US$, 2022).

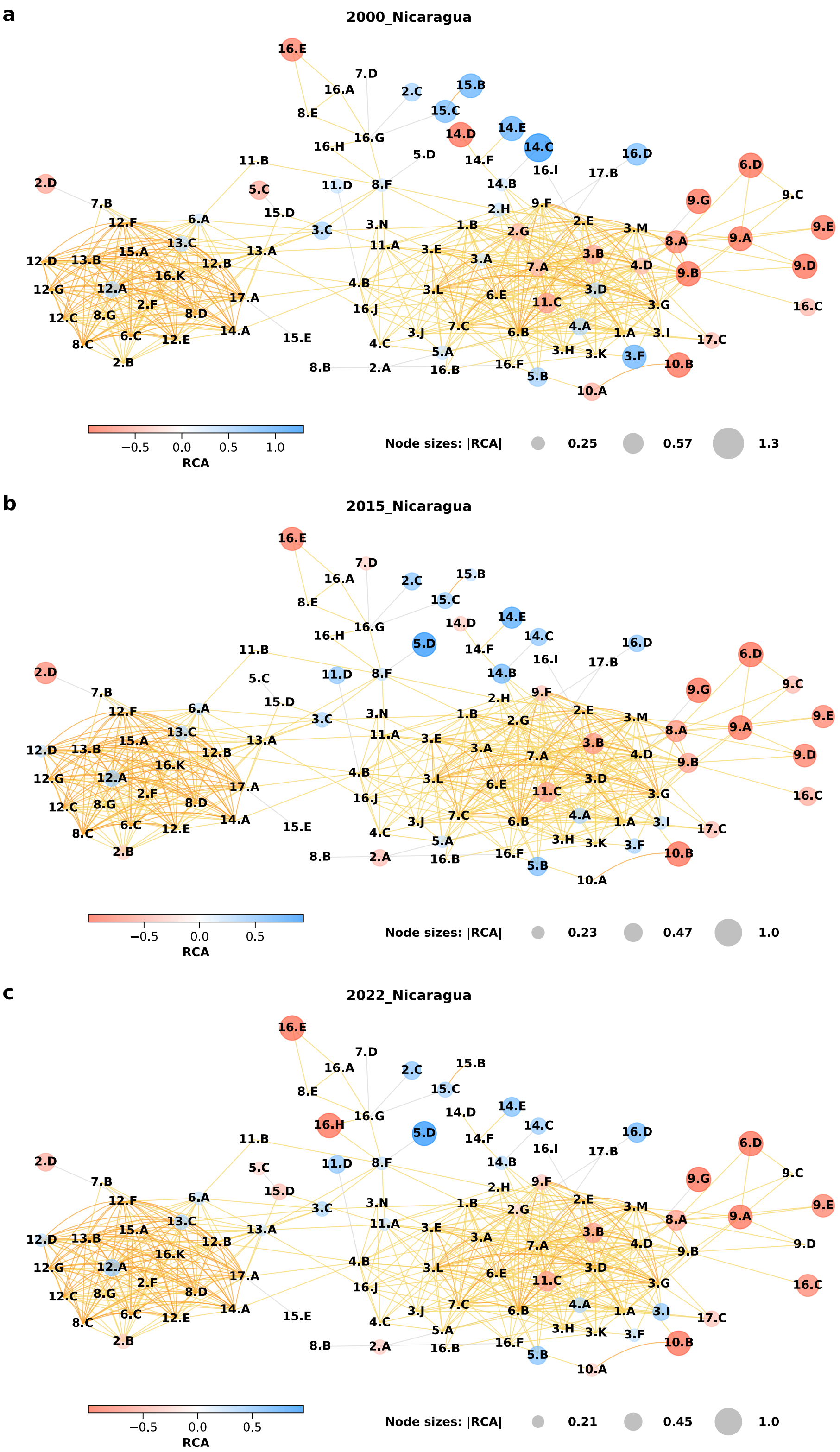

Supplementary Figure 127 | The SDG space of Nicaragua. Panels **a, b, c,** The SDG space in 2000, 2015, and 2022. The nodes in blue and orange represent the top 20 and bottom 20 SDG indicators in revealed comparative advantage (RCA) values, respectively. The node size represents the absolute value of RCA. From Supplementary Figure 12 to 177, countries are ranked by GDP/capita (current US$, 2022).

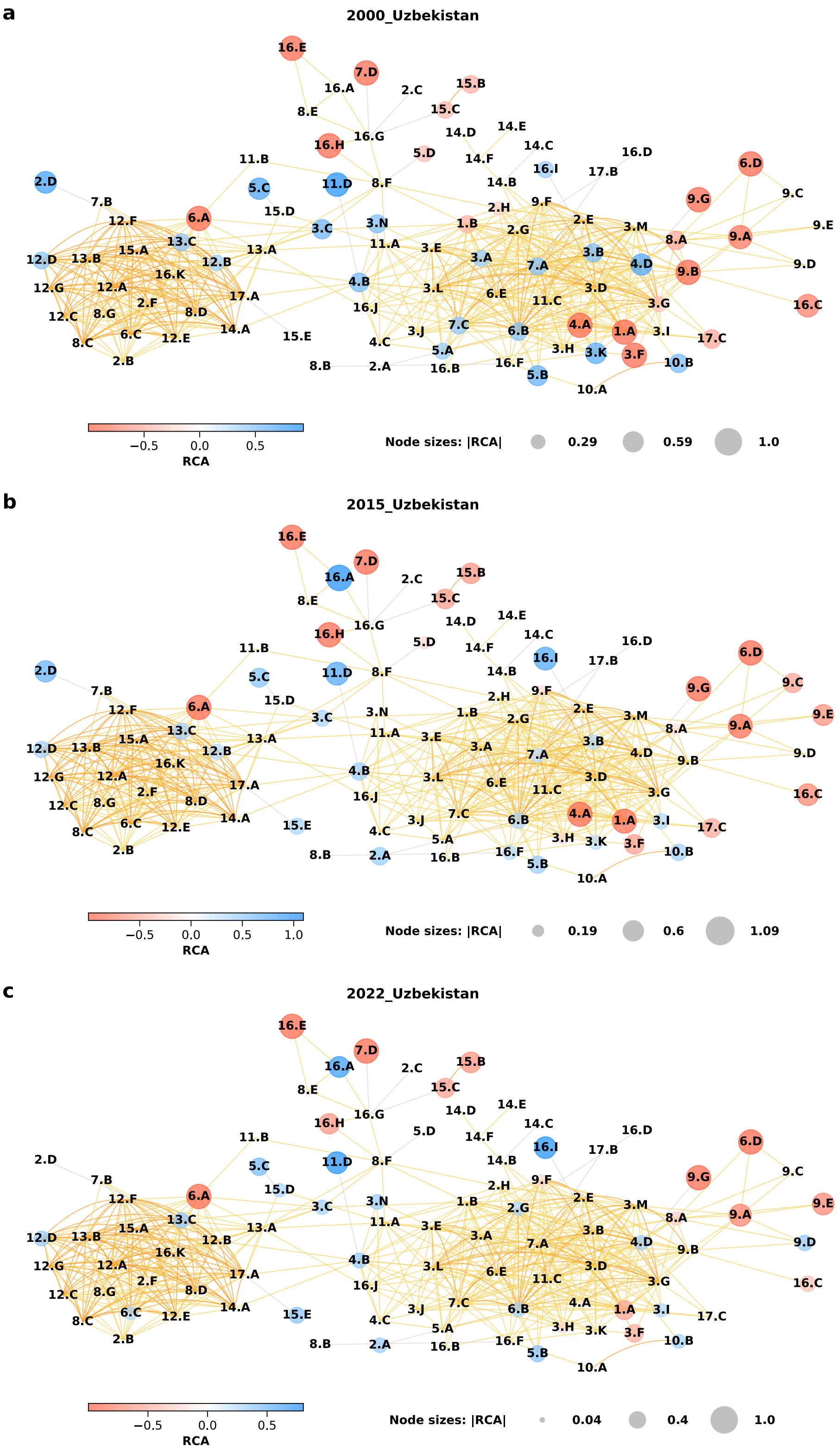

Supplementary Figure 128 | The SDG space of Uzbekistan. Panels **a, b, c,** The SDG space in 2000, 2015, and 2022. The nodes in blue and orange represent the top 20 and bottom 20 SDG indicators in revealed comparative advantage (RCA) values, respectively. The node size represents the absolute value of RCA. From Supplementary Figure 12 to 177, countries are ranked by GDP/capita (current US$, 2022).

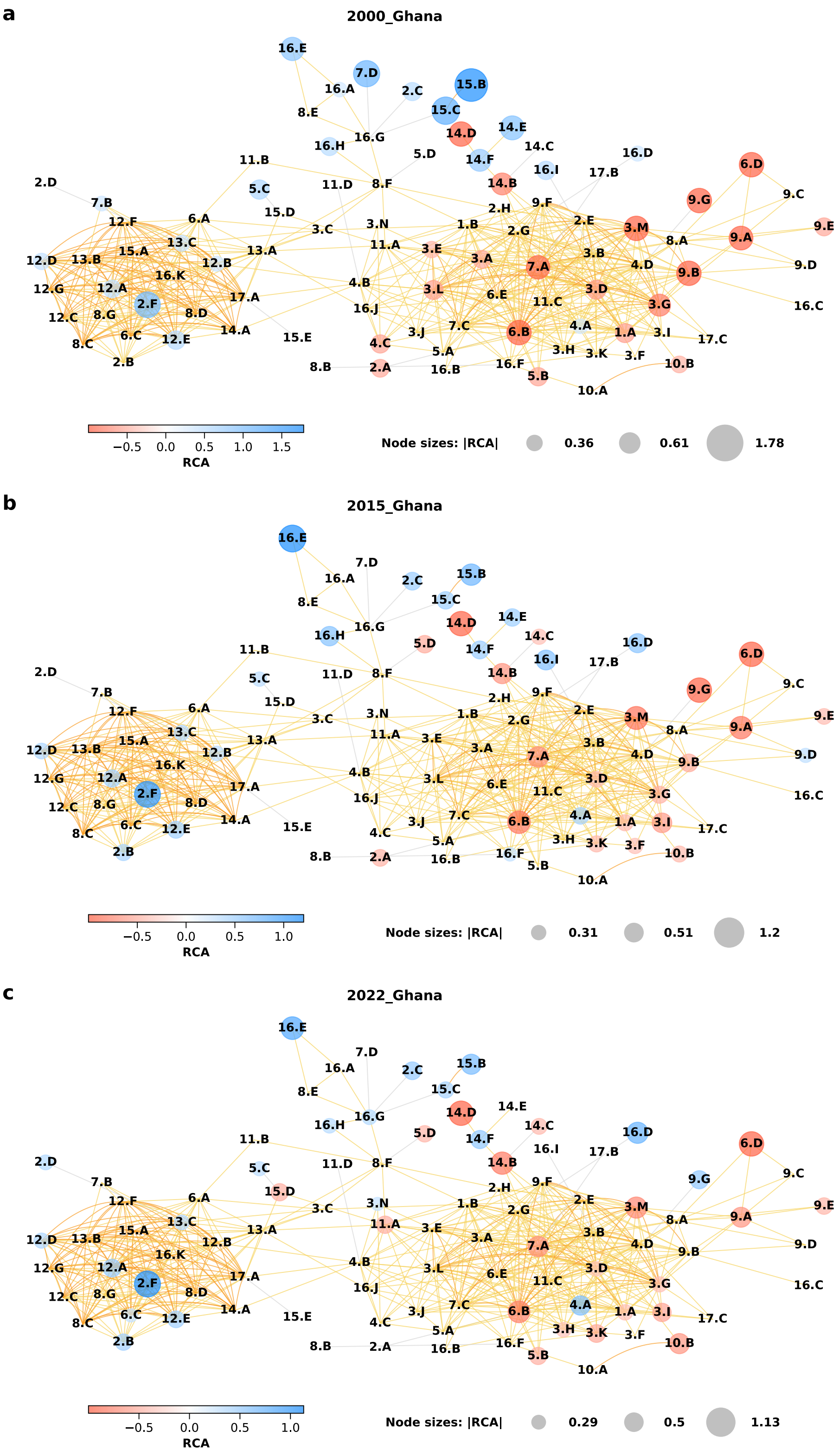

Supplementary Figure 129 | The SDG space of Ghana. Panels **a, b, c,** The SDG space in 2000, 2015, and 2022. The nodes in blue and orange represent the top 20 and bottom 20 SDG indicators in revealed comparative advantage (RCA) values, respectively. The node size represents the absolute value of RCA. From Supplementary Figure 12 to 177, countries are ranked by GDP/capita (current US$, 2022).

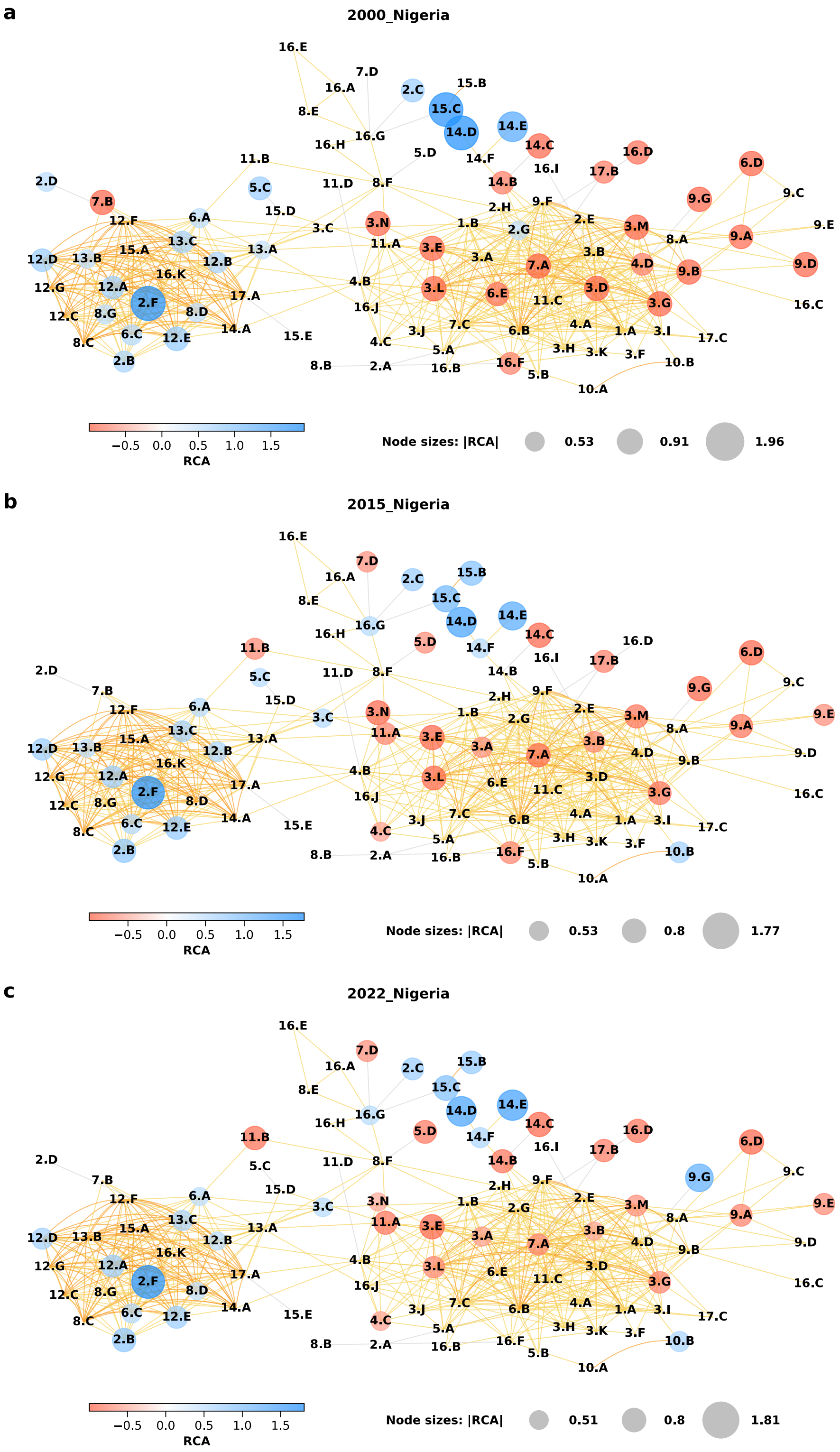

Supplementary Figure 130 | The SDG space of Nigeria. Panels **a, b, c,** The SDG space in 2000, 2015, and 2022. The nodes in blue and orange represent the top 20 and bottom 20 SDG indicators in revealed comparative advantage (RCA) values, respectively. The node size represents the absolute value of RCA. From Supplementary Figure 12 to 177, countries are ranked by GDP/capita (current US$, 2022).

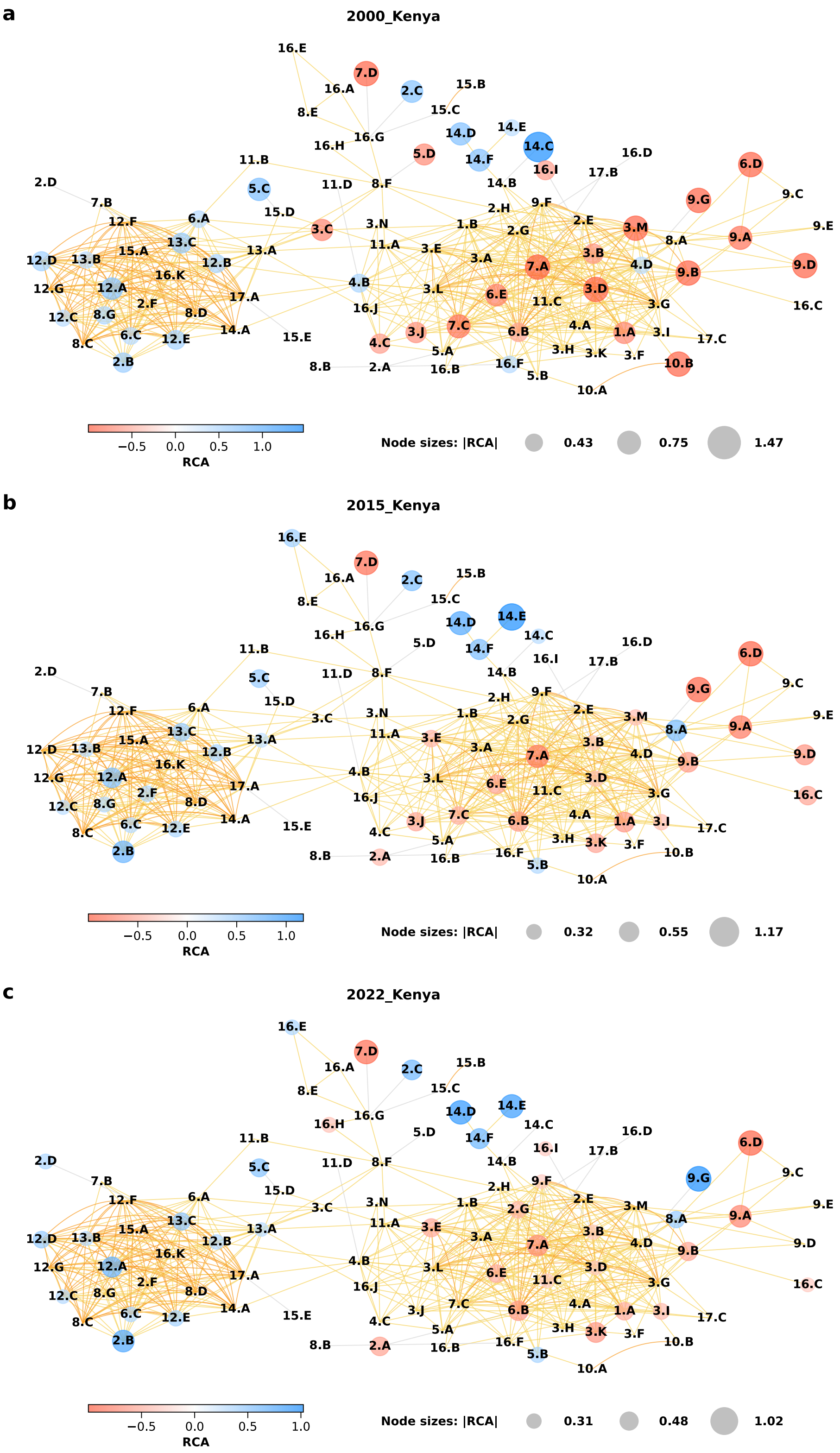

Supplementary Figure 131 | The SDG space of Kenya. Panels **a, b, c,** The SDG space in 2000, 2015, and 2022. The nodes in blue and orange represent the top 20 and bottom 20 SDG indicators in revealed comparative advantage (RCA) values, respectively. The node size represents the absolute value of RCA. From Supplementary Figure 12 to 177, countries are ranked by GDP/capita (current US$, 2022).

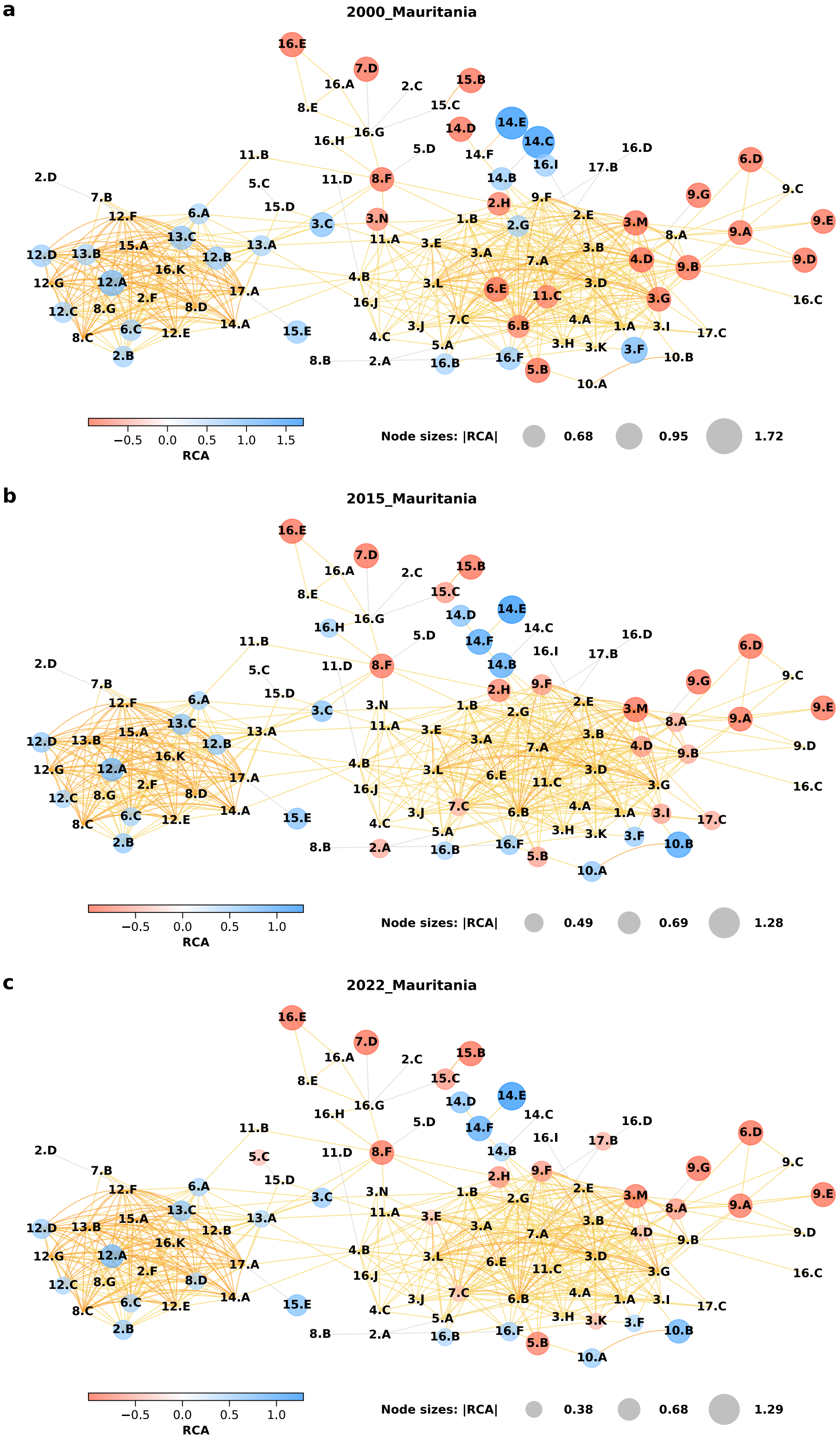

Supplementary Figure 132 | The SDG space of Mauritania. Panels **a, b, c,** The SDG space in 2000, 2015, and 2022. The nodes in blue and orange represent the top 20 and bottom 20 SDG indicators in revealed comparative advantage (RCA) values, respectively. The node size represents the absolute value of RCA. From Supplementary Figure 12 to 177, countries are ranked by GDP/capita (current US$, 2022).

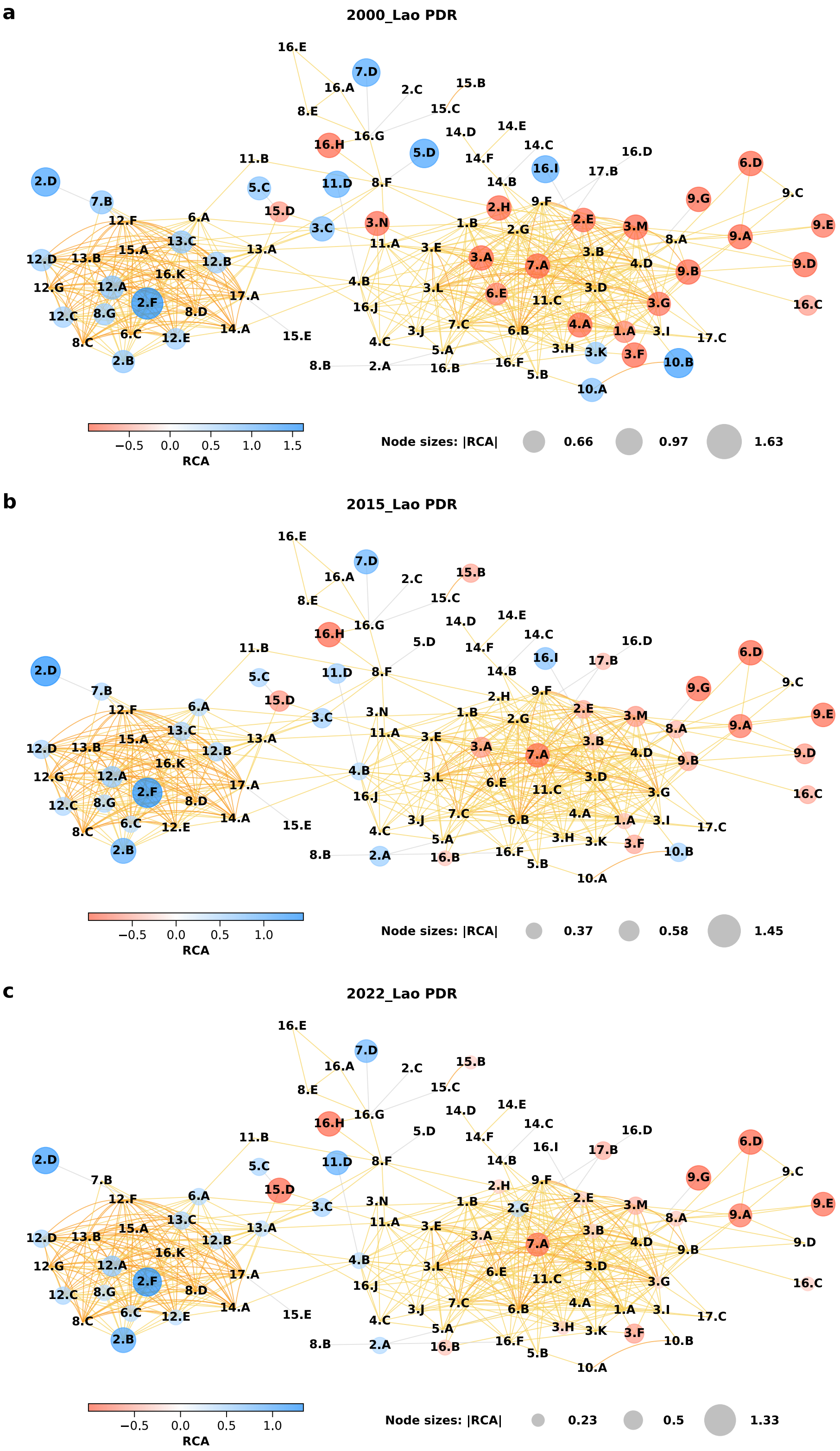

Supplementary Figure 133 | The SDG space of Lao PDR. Panels **a, b, c,** The SDG space in 2000, 2015, and 2022. The nodes in blue and orange represent the top 20 and bottom 20 SDG indicators in revealed comparative advantage (RCA) values, respectively. The node size represents the absolute value of RCA. From Supplementary Figure 12 to 177, countries are ranked by GDP/capita (current US$, 2022).

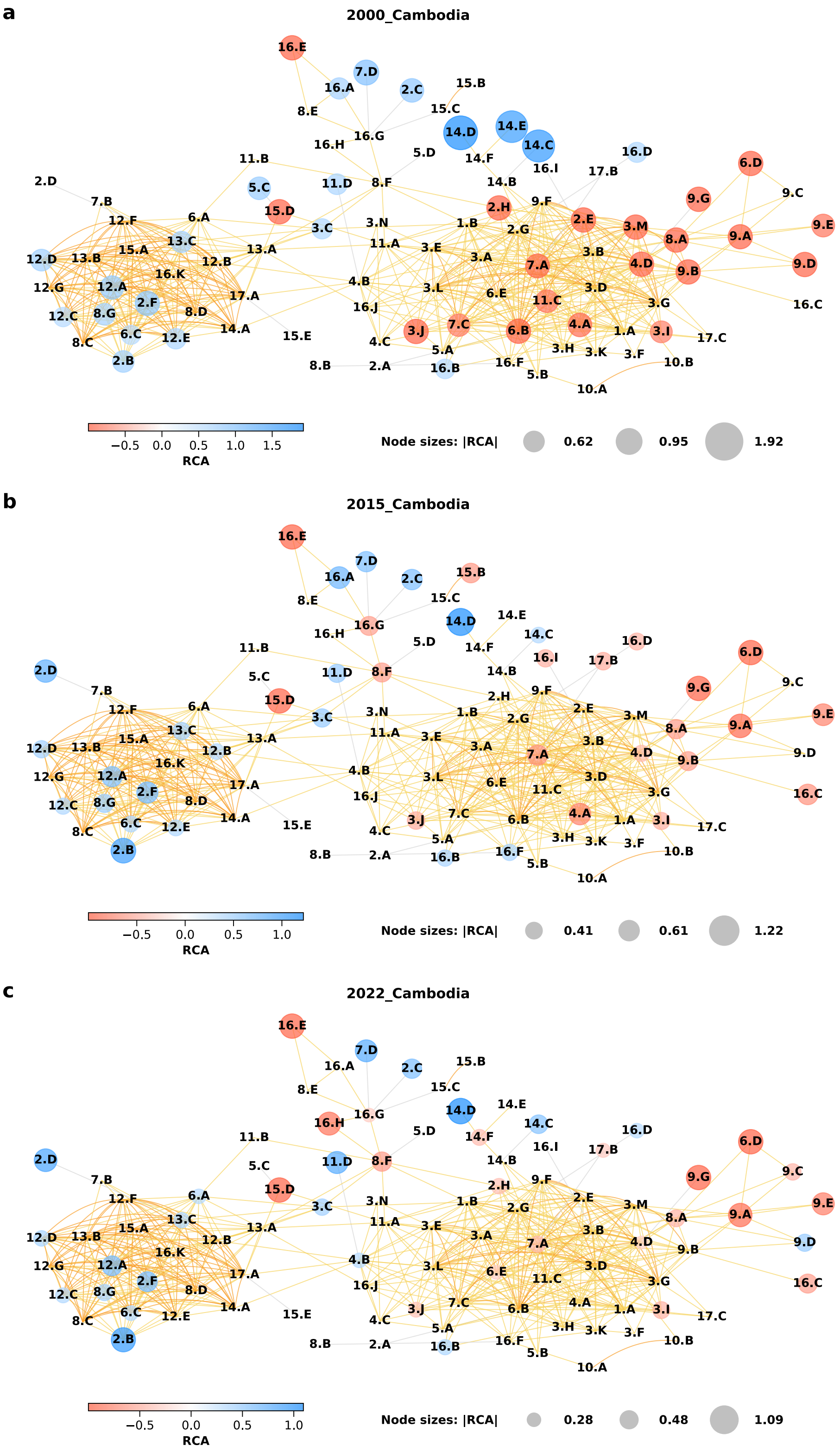

Supplementary Figure 134 | The SDG space of Cambodia. Panels **a, b, c,** The SDG space in 2000, 2015, and 2022. The nodes in blue and orange represent the top 20 and bottom 20 SDG indicators in revealed comparative advantage (RCA) values, respectively. The node size represents the absolute value of RCA. From Supplementary Figure 12 to 177, countries are ranked by GDP/capita (current US$, 2022).

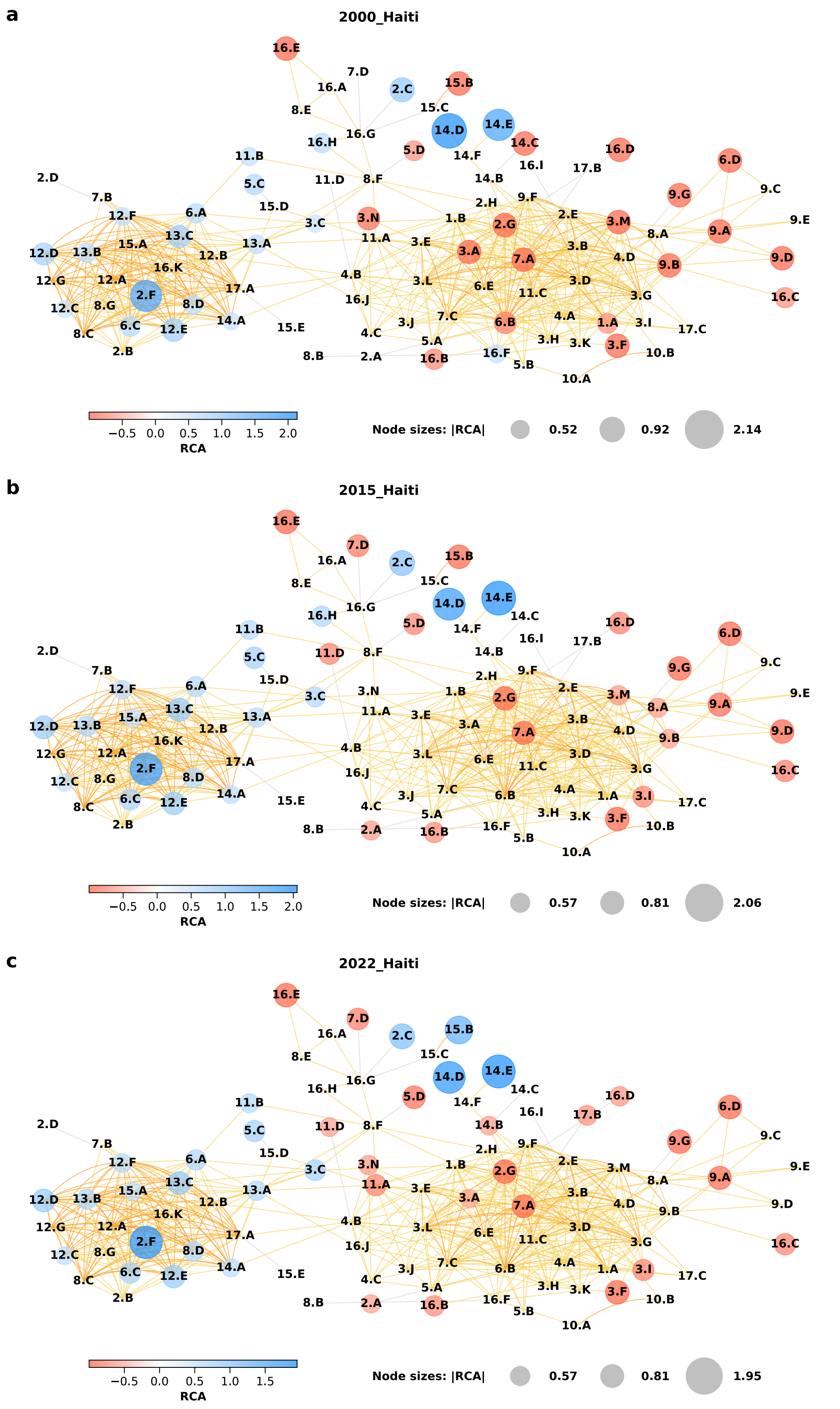

Supplementary Figure 135 | The SDG space of Haiti. Panels **a, b, c,** The SDG space in 2000, 2015, and 2022. The nodes in blue and orange represent the top 20 and bottom 20 SDG indicators in revealed comparative advantage (RCA) values, respectively. The node size represents the absolute value of RCA. From Supplementary Figure 12 to 177, countries are ranked by GDP/capita (current US$, 2022).

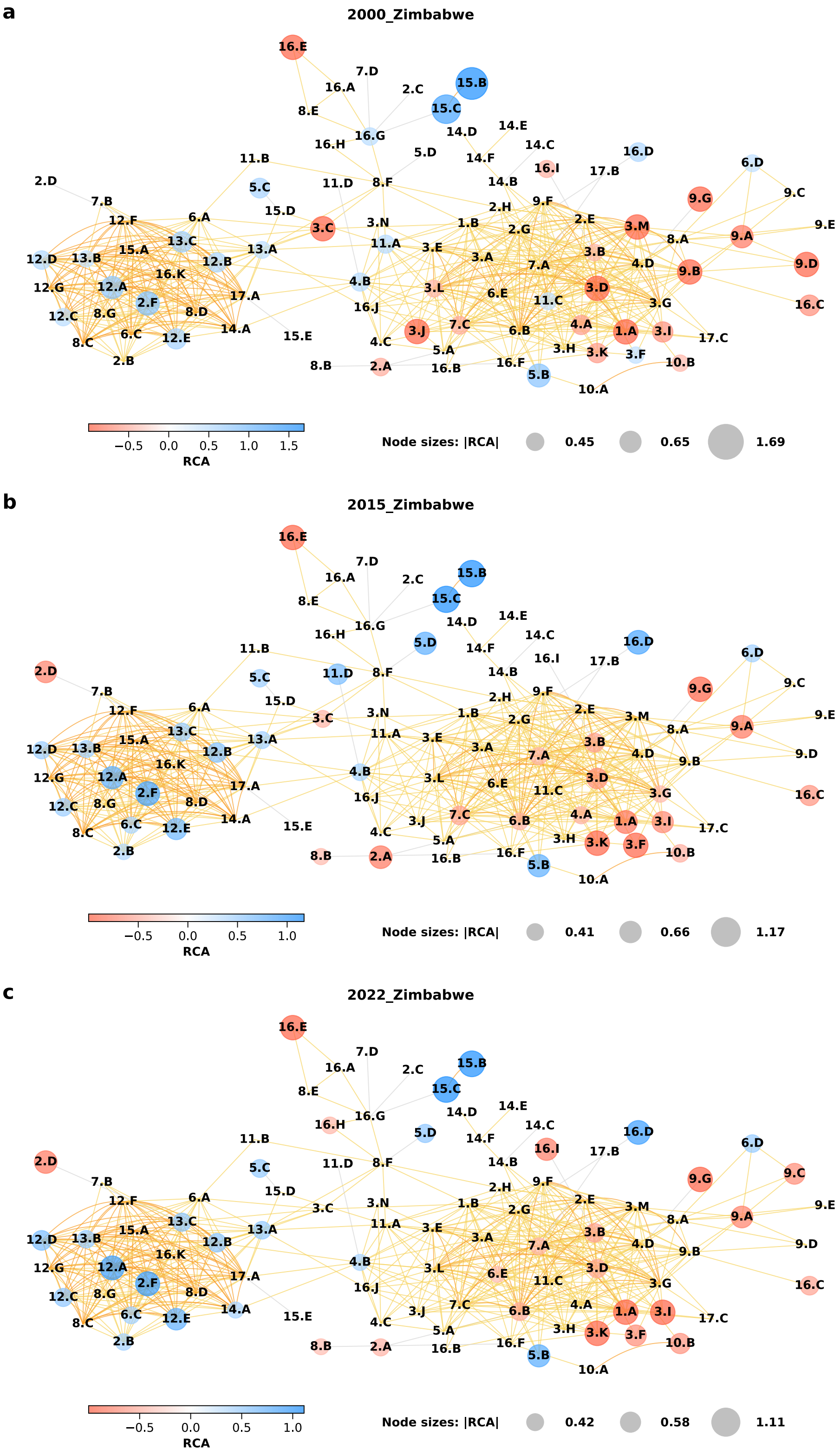

Supplementary Figure 136 | The SDG space of Zimbabwe. Panels **a, b, c,** The SDG space in 2000, 2015, and 2022. The nodes in blue and orange represent the top 20 and bottom 20 SDG indicators in revealed comparative advantage (RCA) values, respectively. The node size represents the absolute value of RCA. From Supplementary Figure 12 to 177, countries are ranked by GDP/capita (current US$, 2022).

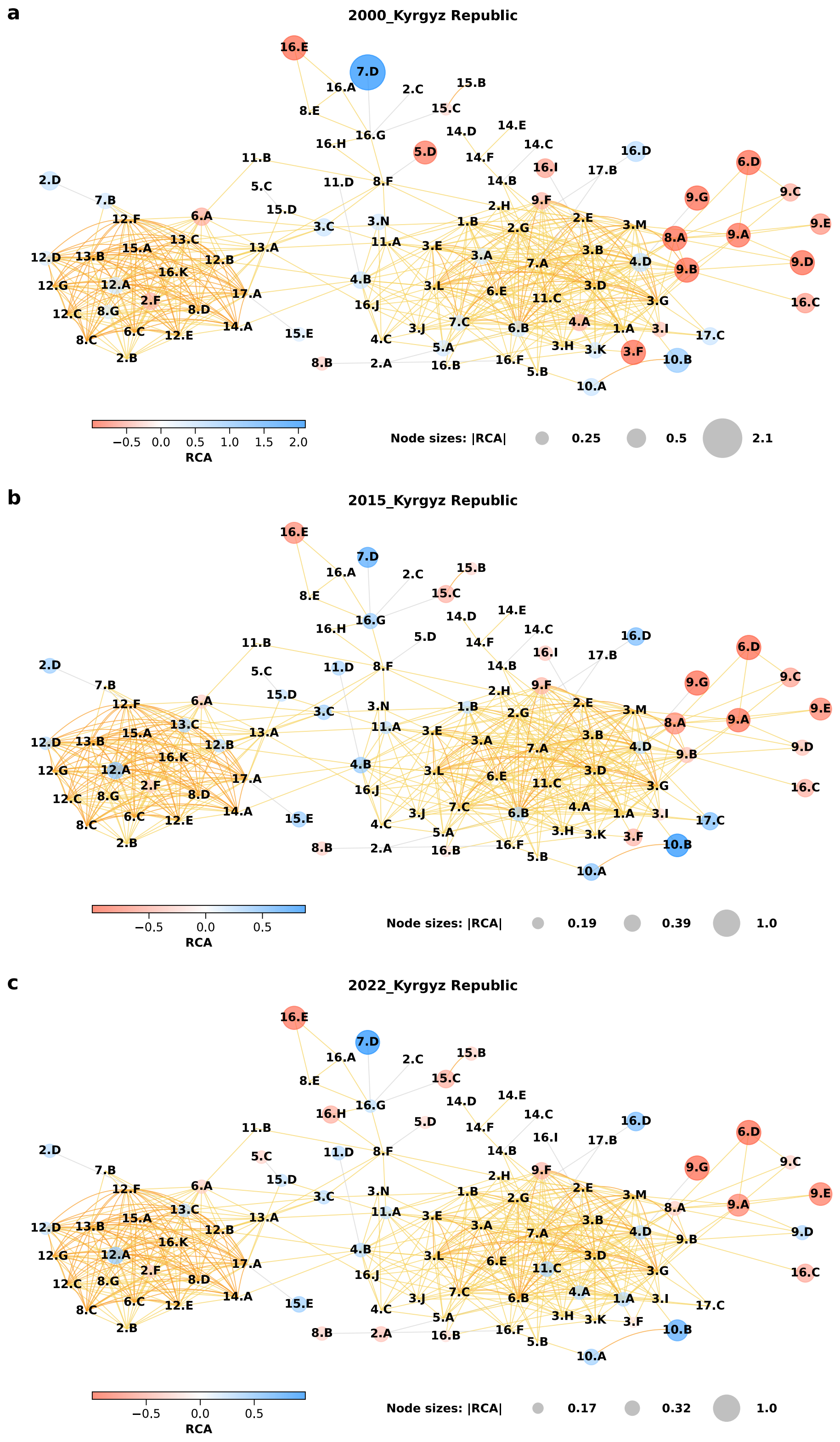

Supplementary Figure 137 | The SDG space of Kyrgyz Republic. Panels **a, b, c,** The SDG space in 2000, 2015, and 2022. The nodes in blue and orange represent the top 20 and bottom 20 SDG indicators in revealed comparative advantage (RCA) values, respectively. The node size represents the absolute value of RCA. From Supplementary Figure 12 to 177, countries are ranked by GDP/capita (current US$, 2022).

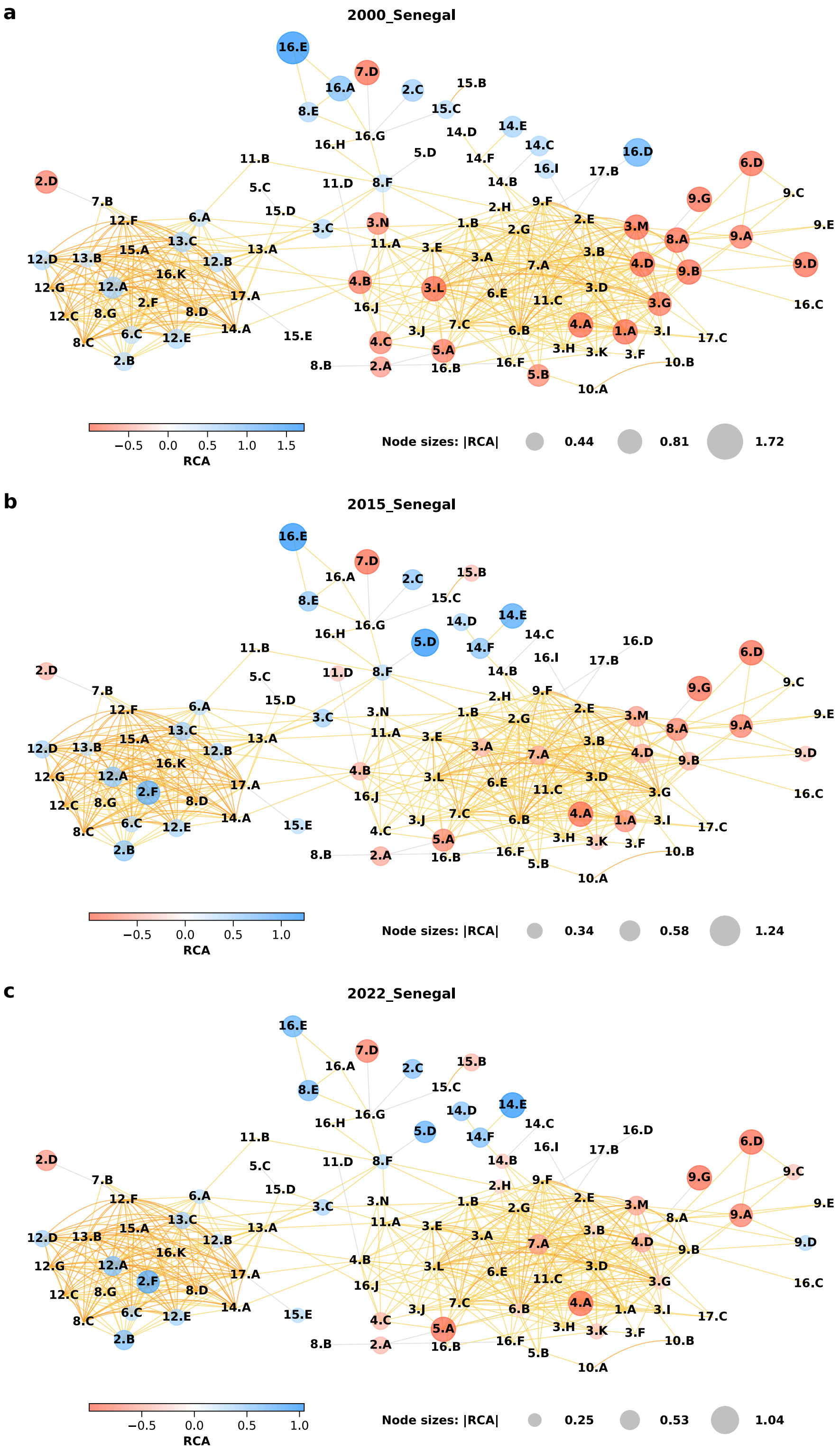

Supplementary Figure 138 | The SDG space of Senegal. Panels **a, b, c,** The SDG space in 2000, 2015, and 2022. The nodes in blue and orange represent the top 20 and bottom 20 SDG indicators in revealed comparative advantage (RCA) values, respectively. The node size represents the absolute value of RCA. From Supplementary Figure 12 to 177, countries are ranked by GDP/capita (current US$, 2022).

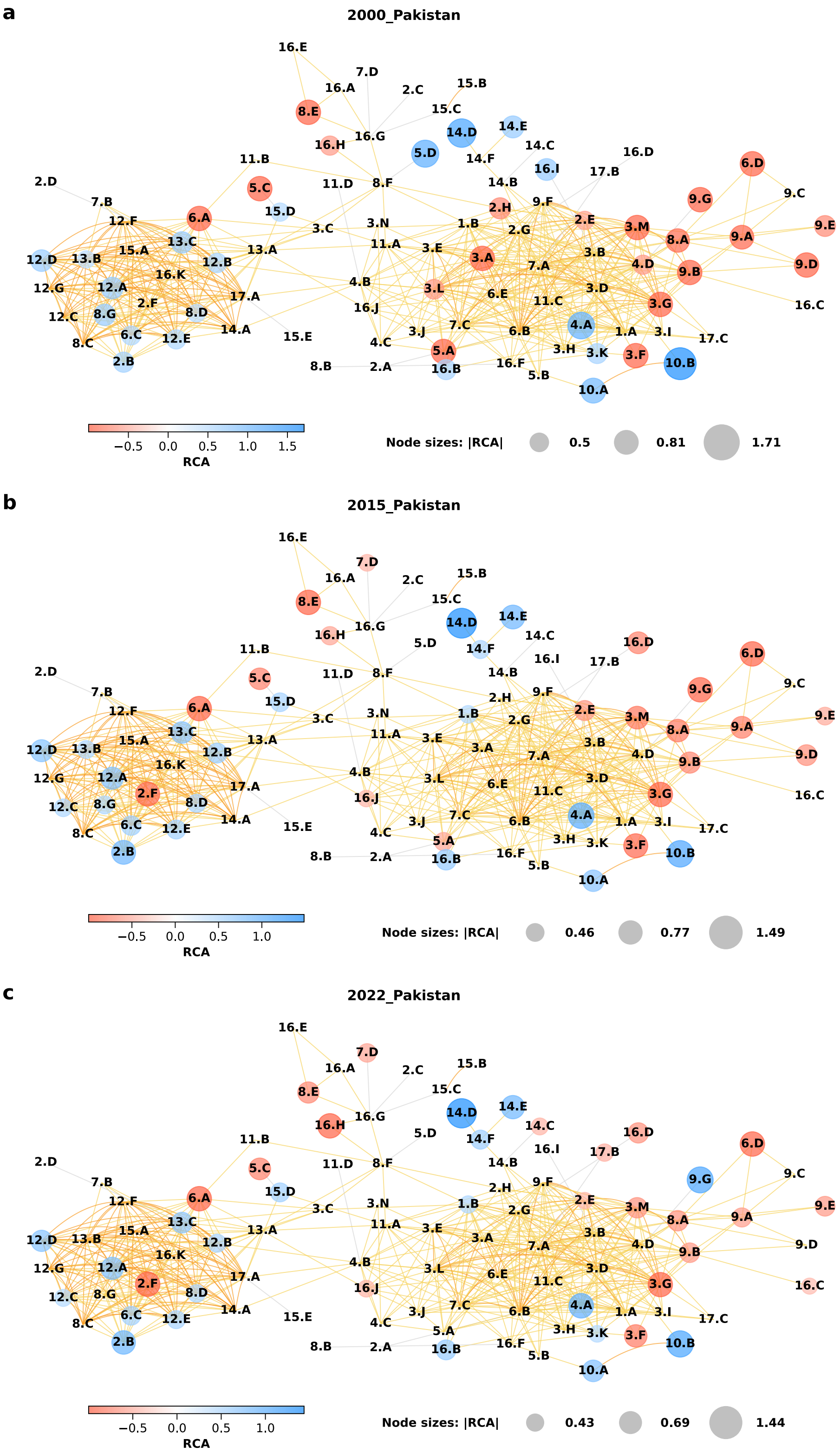

Supplementary Figure 139 | The SDG space of Pakistan. Panels **a, b, c,** The SDG space in 2000, 2015, and 2022. The nodes in blue and orange represent the top 20 and bottom 20 SDG indicators in revealed comparative advantage (RCA) values, respectively. The node size represents the absolute value of RCA. From Supplementary Figure 12 to 177, countries are ranked by GDP/capita (current US$, 2022).

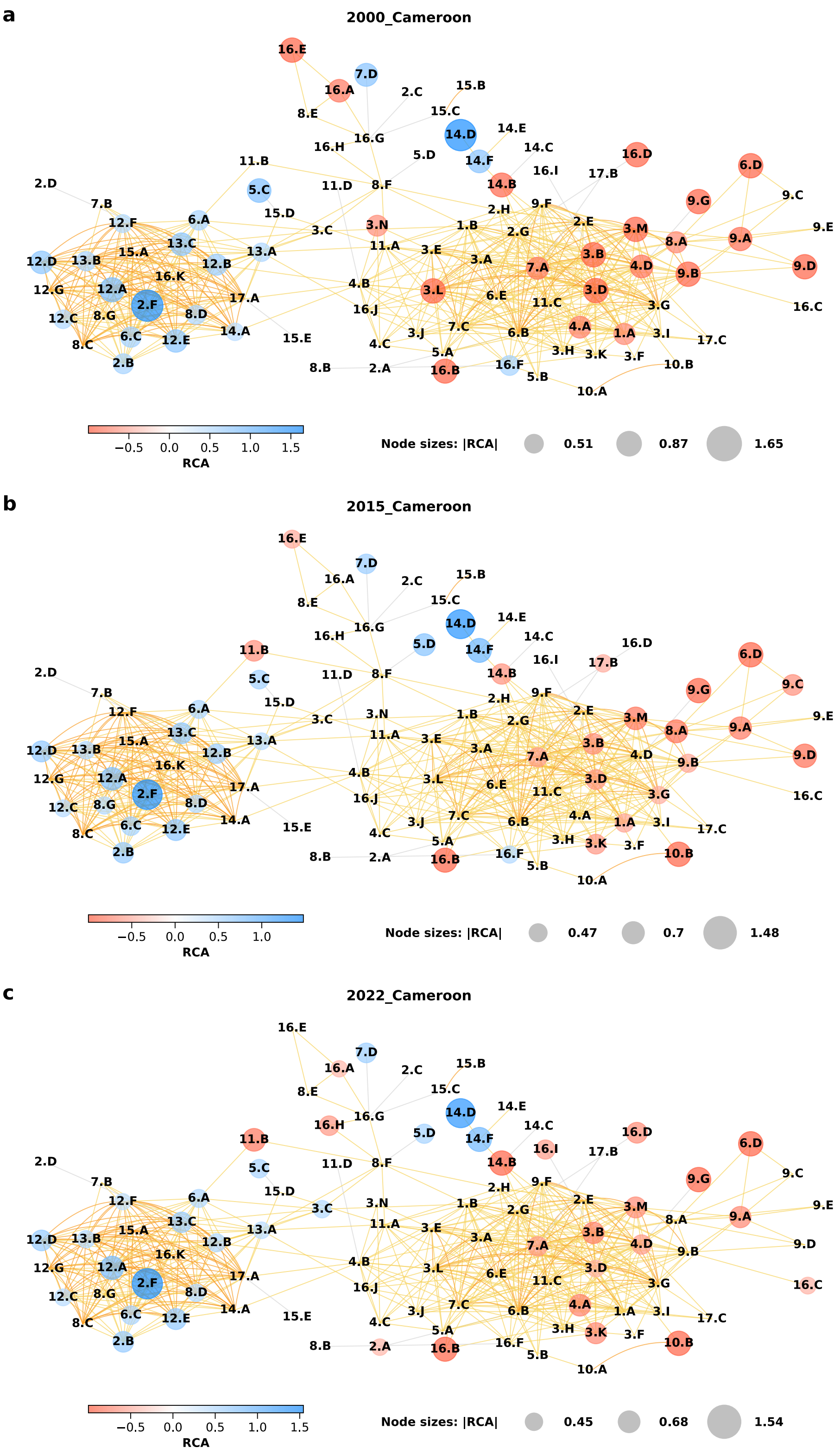

Supplementary Figure 140 | The SDG space of Cameroon. Panels **a, b, c,** The SDG space in 2000, 2015, and 2022. The nodes in blue and orange represent the top 20 and bottom 20 SDG indicators in revealed comparative advantage (RCA) values, respectively. The node size represents the absolute value of RCA. From Supplementary Figure 12 to 177, countries are ranked by GDP/capita (current US$, 2022).

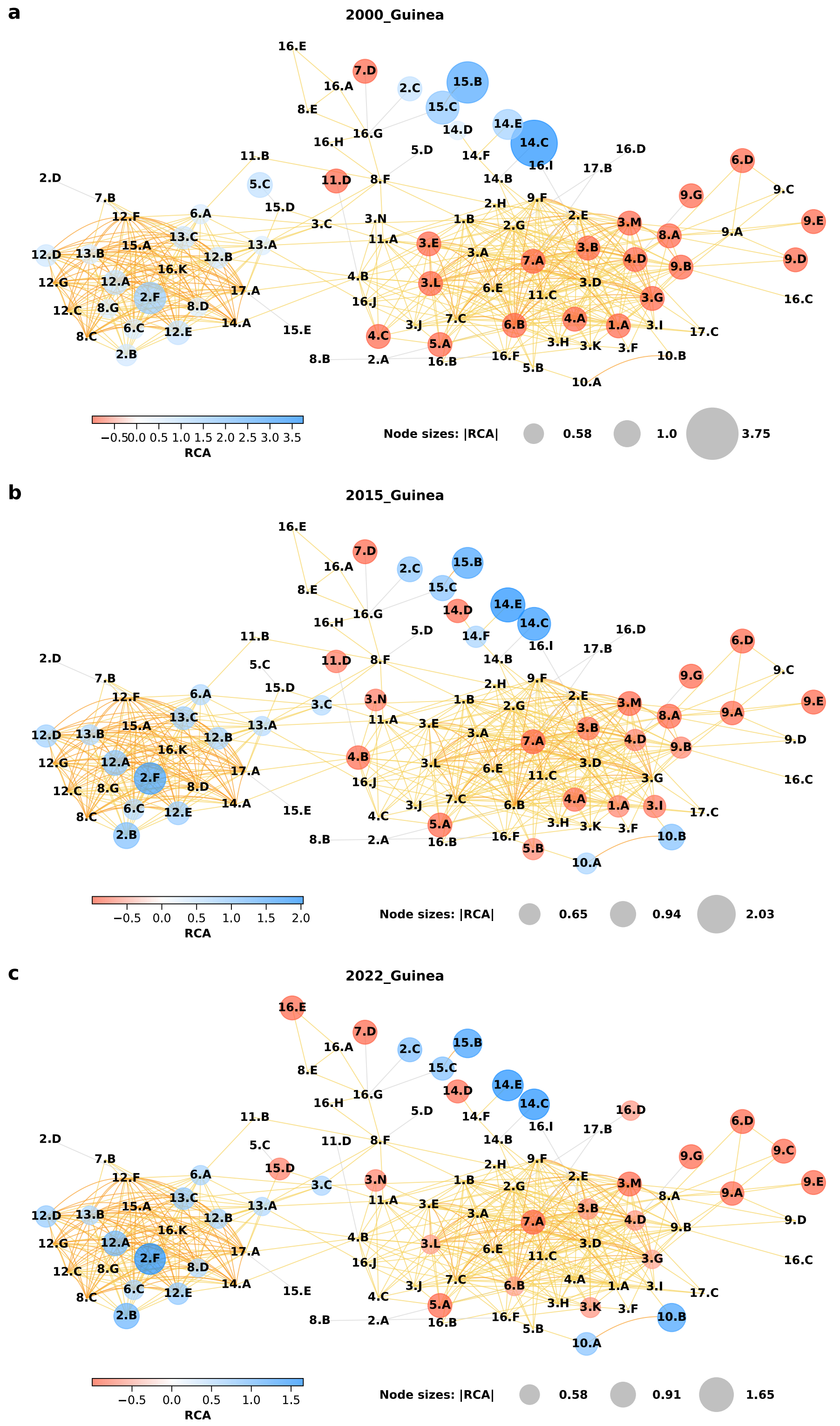

Supplementary Figure 141 | The SDG space of Guinea. Panels **a, b, c,** The SDG space in 2000, 2015, and 2022. The nodes in blue and orange represent the top 20 and bottom 20 SDG indicators in revealed comparative advantage (RCA) values, respectively. The node size represents the absolute value of RCA. From Supplementary Figure 12 to 177, countries are ranked by GDP/capita (current US$, 2022).

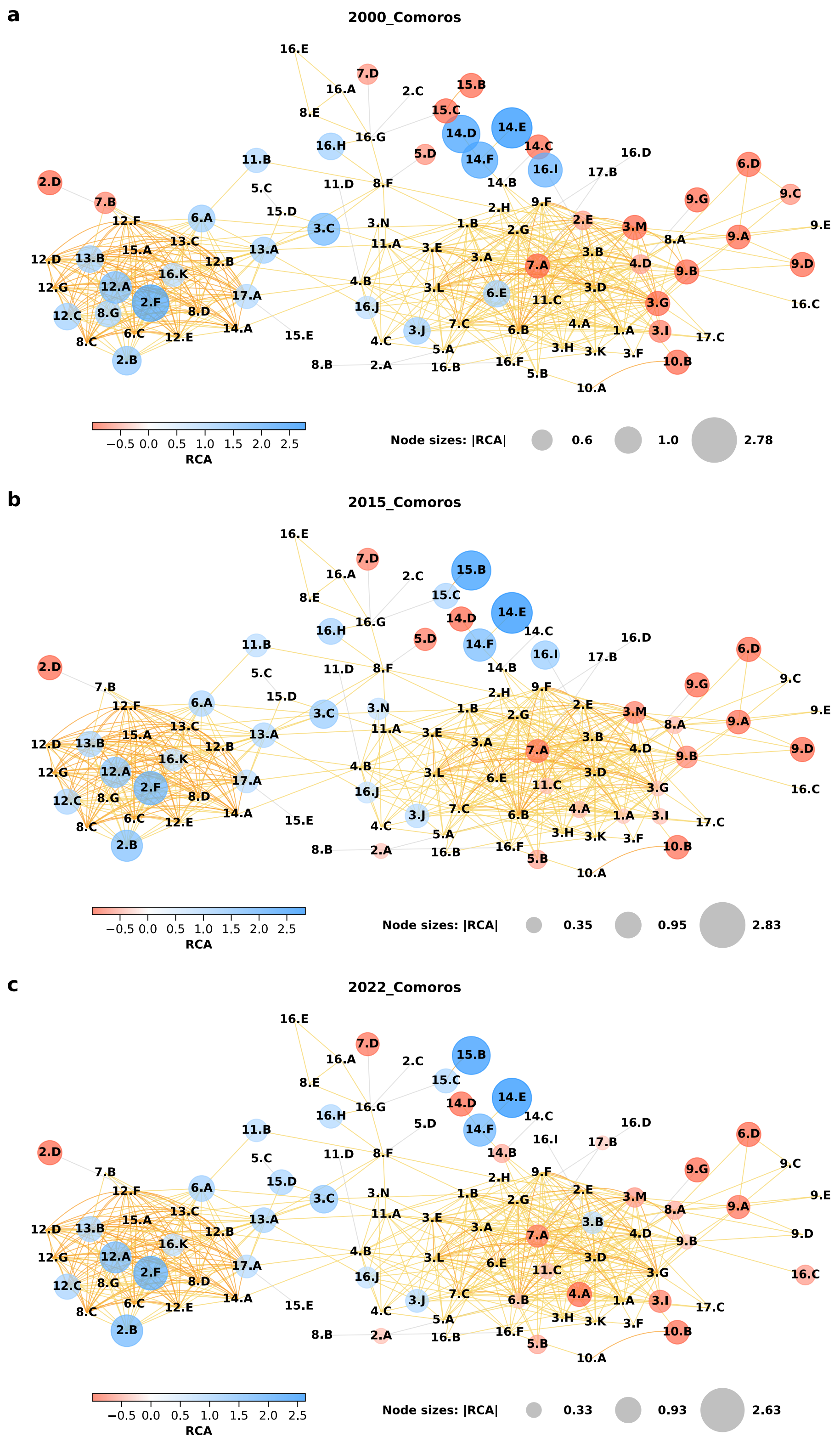

Supplementary Figure 142 | The SDG space of Comoros. Panels **a, b, c,** The SDG space in 2000, 2015, and 2022. The nodes in blue and orange represent the top 20 and bottom 20 SDG indicators in revealed comparative advantage (RCA) values, respectively. The node size represents the absolute value of RCA. From Supplementary Figure 12 to 177, countries are ranked by GDP/capita (current US$, 2022).

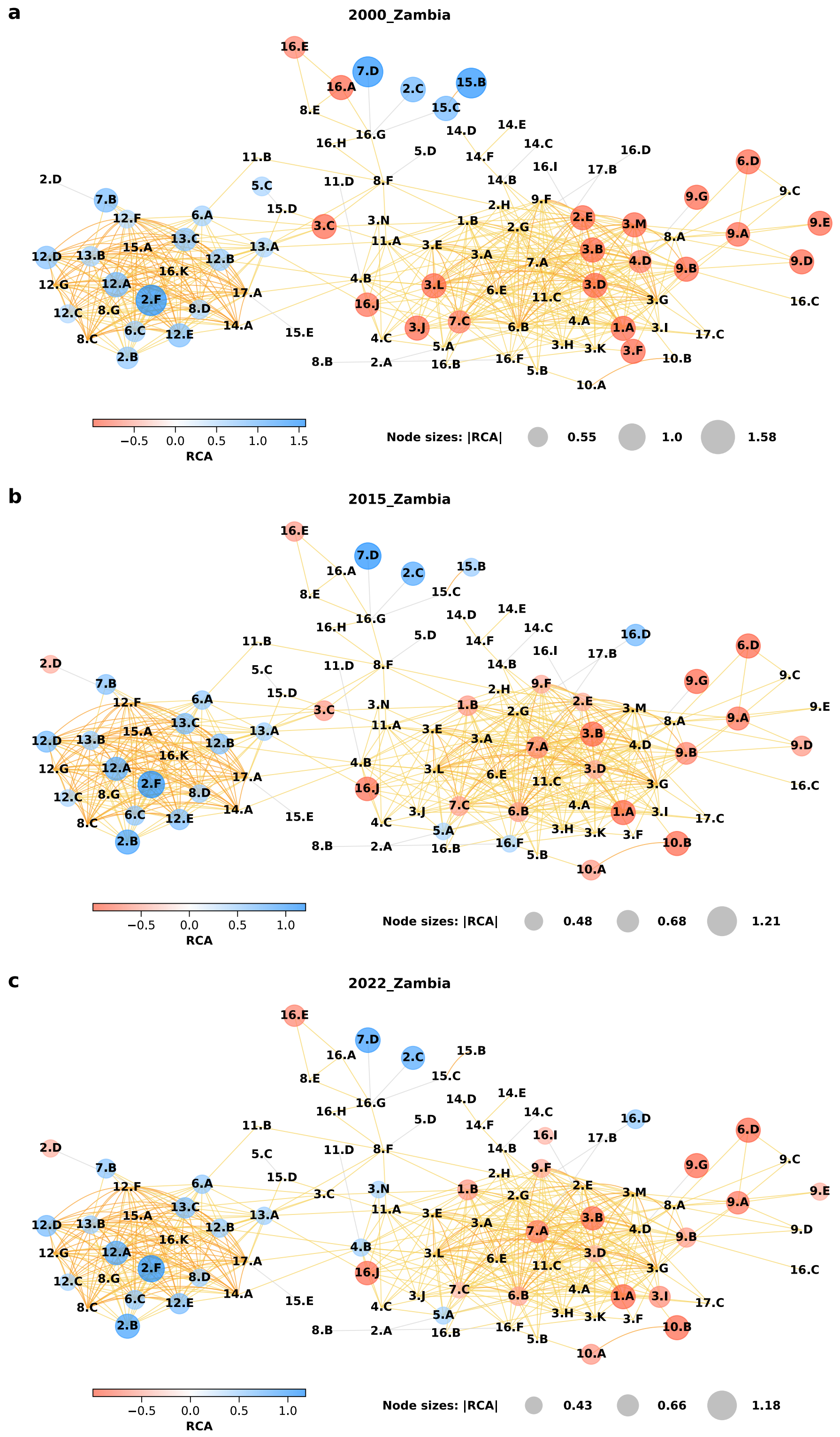

Supplementary Figure 143 | The SDG space of Zambia. Panels **a, b, c,** The SDG space in 2000, 2015, and 2022. The nodes in blue and orange represent the top 20 and bottom 20 SDG indicators in revealed comparative advantage (RCA) values, respectively. The node size represents the absolute value of RCA. From Supplementary Figure 12 to 177, countries are ranked by GDP/capita (current US$, 2022).

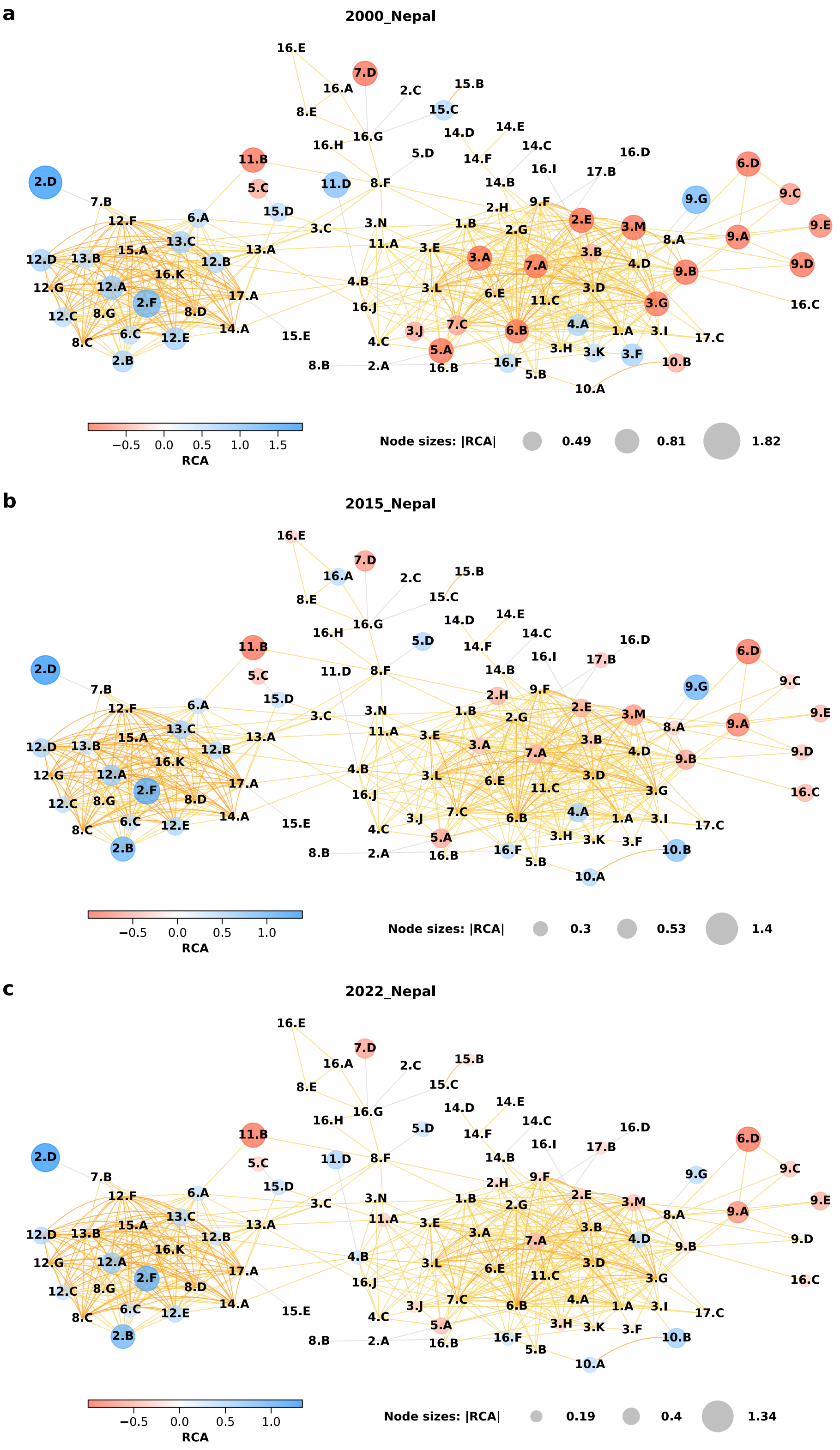

Supplementary Figure 144 | The SDG space of Nepal. Panels **a, b, c,** The SDG space in 2000, 2015, and 2022. The nodes in blue and orange represent the top 20 and bottom 20 SDG indicators in revealed comparative advantage (RCA) values, respectively. The node size represents the absolute value of RCA. From Supplementary Figure 12 to 177, countries are ranked by GDP/capita (current US$, 2022).

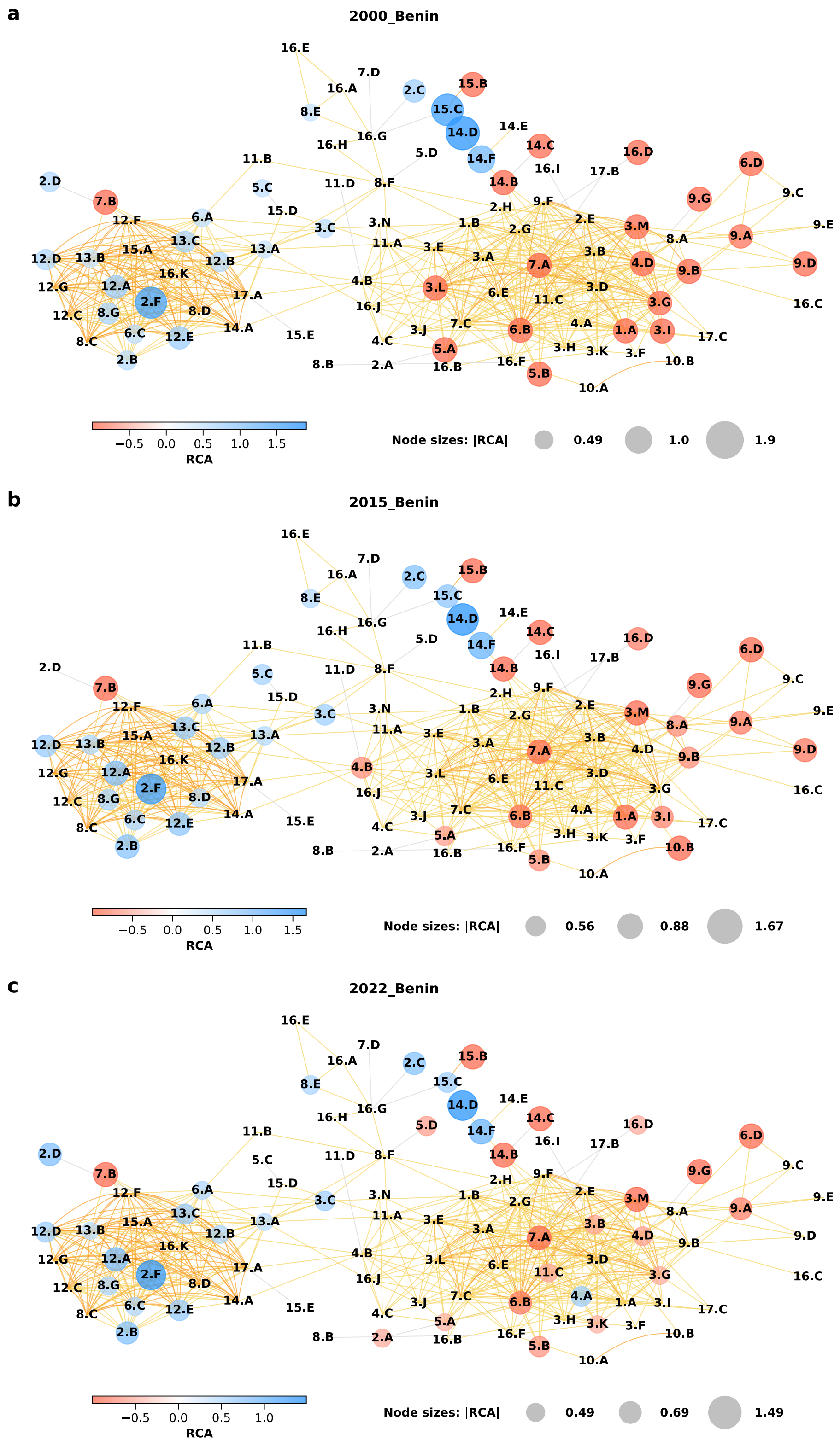

Supplementary Figure 145 | The SDG space of Benin. Panels **a, b, c,** The SDG space in 2000, 2015, and 2022. The nodes in blue and orange represent the top 20 and bottom 20 SDG indicators in revealed comparative advantage (RCA) values, respectively. The node size represents the absolute value of RCA. From Supplementary Figure 12 to 177, countries are ranked by GDP/capita (current US$, 2022).

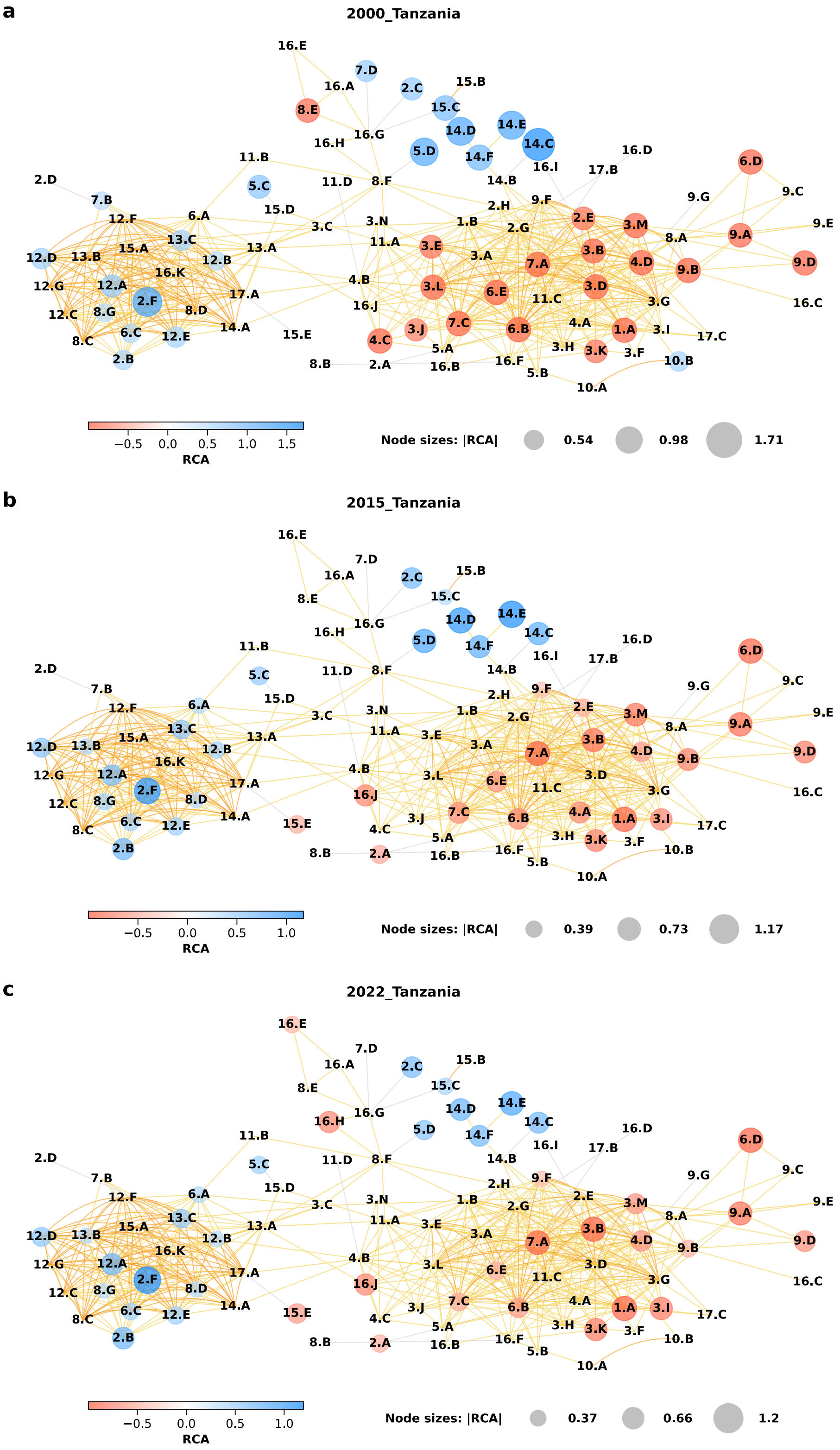

Supplementary Figure 146 | The SDG space of Tanzania. Panels **a, b, c,** The SDG space in 2000, 2015, and 2022. The nodes in blue and orange represent the top 20 and bottom 20 SDG indicators in revealed comparative advantage (RCA) values, respectively. The node size represents the absolute value of RCA. From Supplementary Figure 12 to 177, countries are ranked by GDP/capita (current US$, 2022).

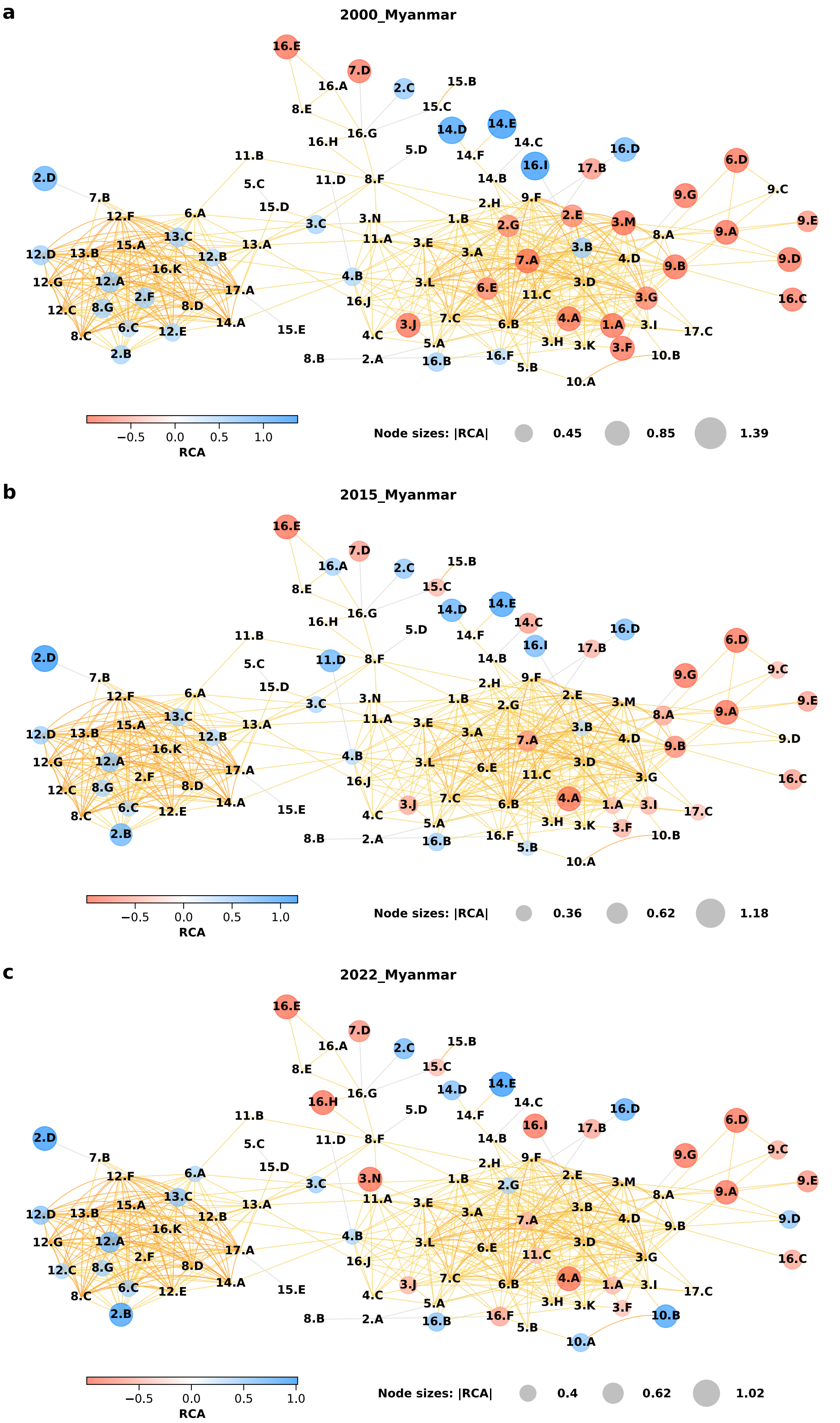

Supplementary Figure 147 | The SDG space of Myanmar. Panels **a, b, c,** The SDG space in 2000, 2015, and 2022. The nodes in blue and orange represent the top 20 and bottom 20 SDG indicators in revealed comparative advantage (RCA) values, respectively. The node size represents the absolute value of RCA. From Supplementary Figure 12 to 177, countries are ranked by GDP/capita (current US$, 2022).

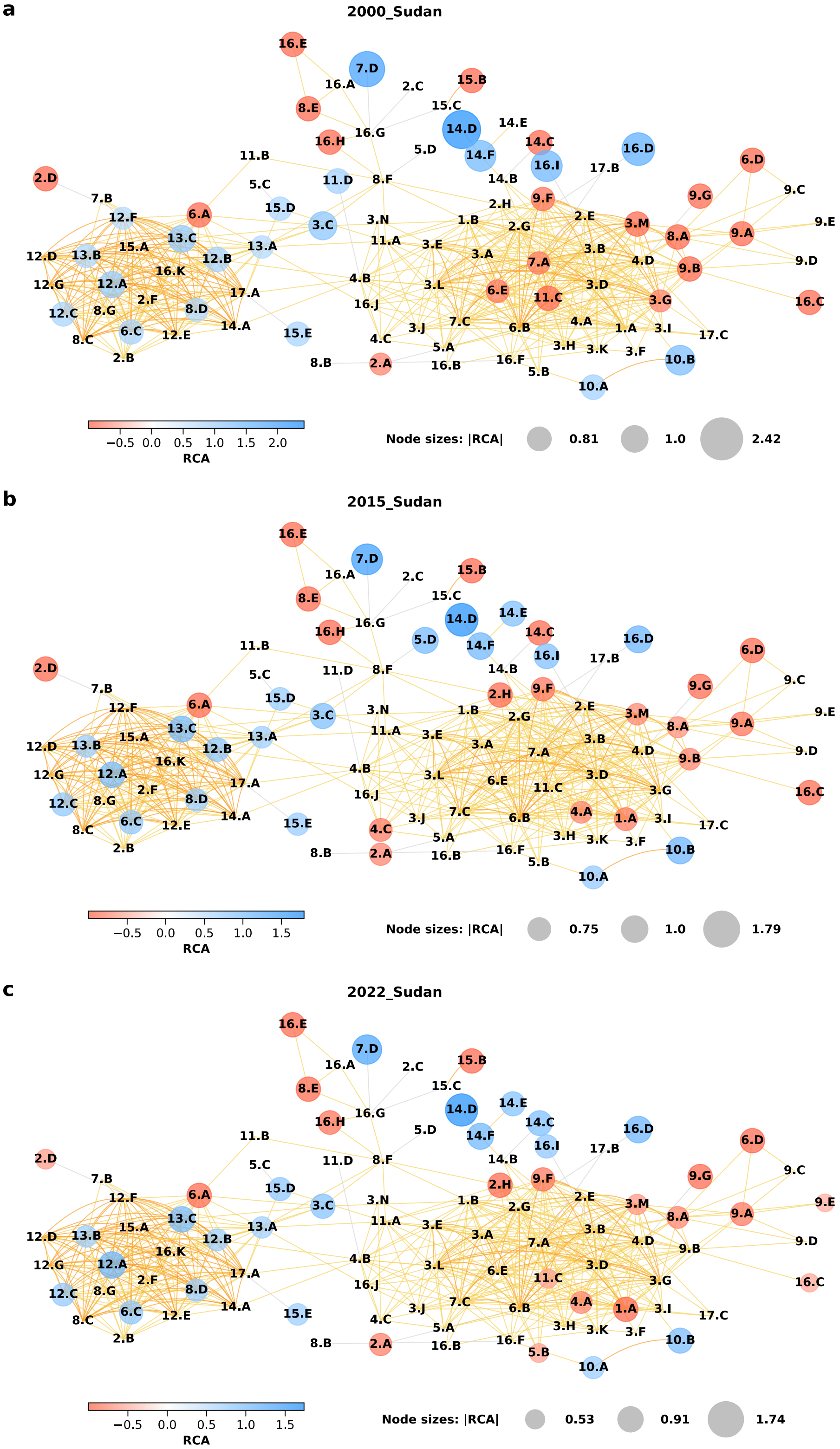

Supplementary Figure 148 | The SDG space of Sudan. Panels **a, b, c,** The SDG space in 2000, 2015, and 2022. The nodes in blue and orange represent the top 20 and bottom 20 SDG indicators in revealed comparative advantage (RCA) values, respectively. The node size represents the absolute value of RCA. From Supplementary Figure 12 to 177, countries are ranked by GDP/capita (current US$, 2022).

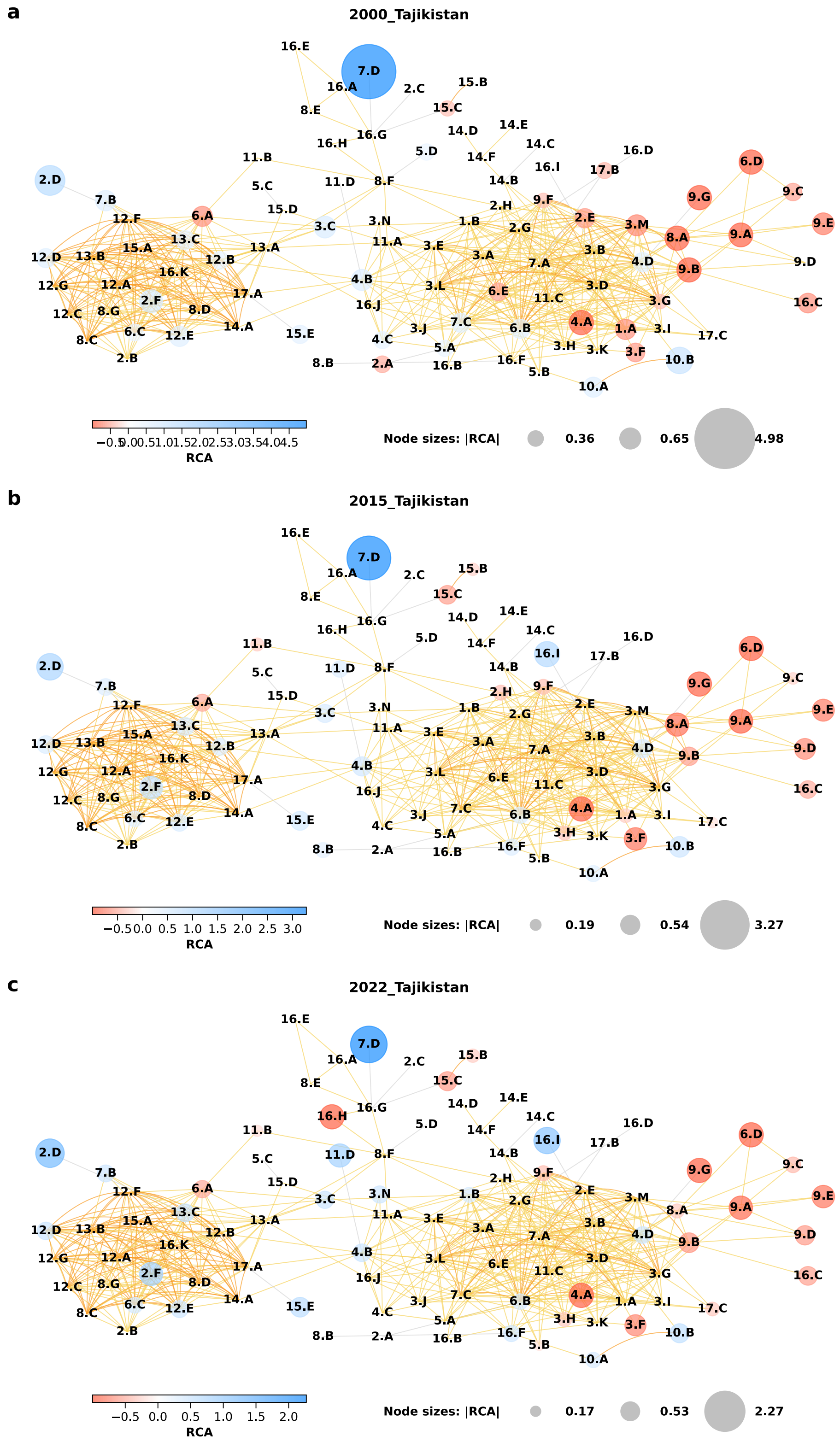

Supplementary Figure 149 | The SDG space of Tajikistan. Panels **a, b, c,** The SDG space in 2000, 2015, and 2022. The nodes in blue and orange represent the top 20 and bottom 20 SDG indicators in revealed comparative advantage (RCA) values, respectively. The node size represents the absolute value of RCA. From Supplementary Figure 12 to 177, countries are ranked by GDP/capita (current US$, 2022).

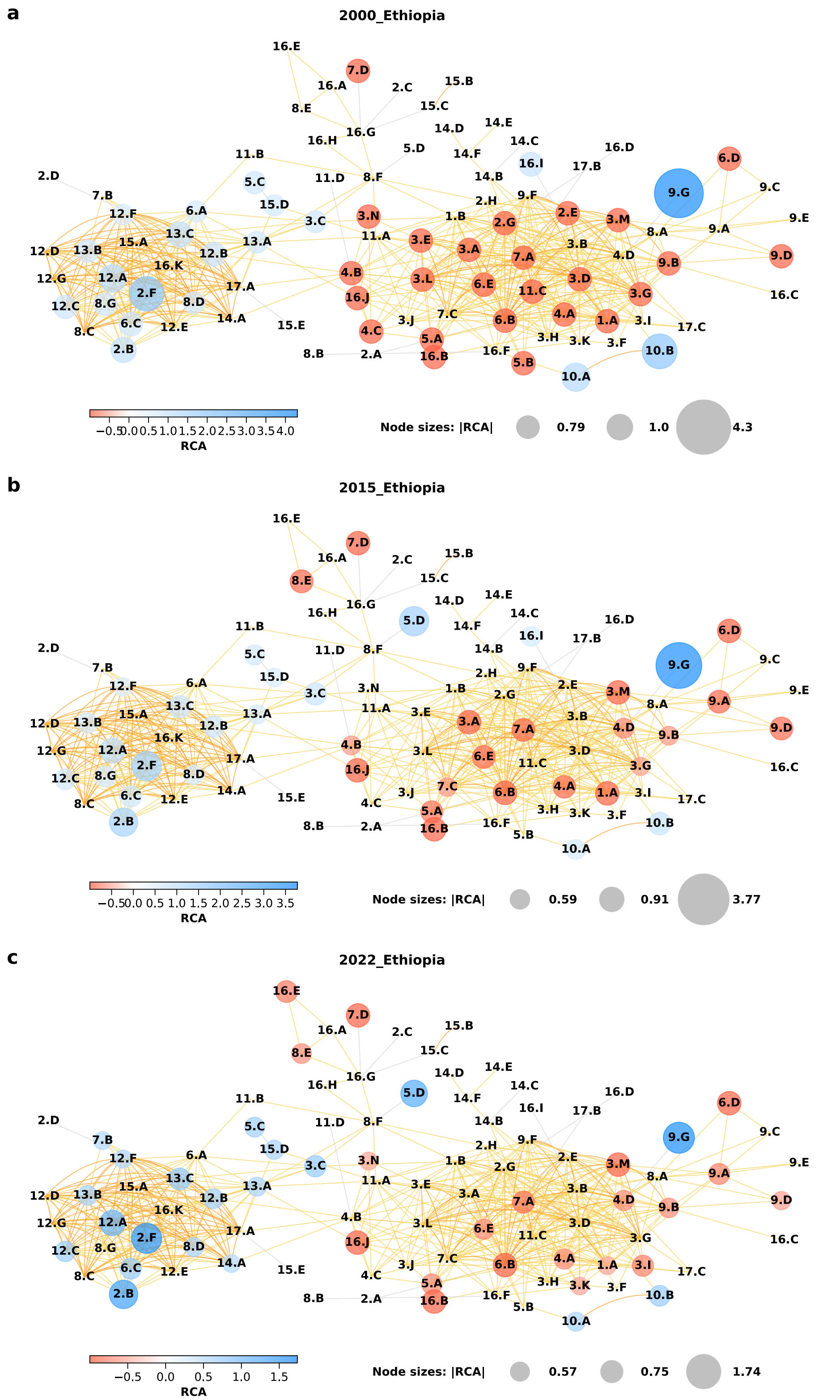

Supplementary Figure 150 | The SDG space of Ethiopia. Panels **a, b, c,** The SDG space in 2000, 2015, and 2022. The nodes in blue and orange represent the top 20 and bottom 20 SDG indicators in revealed comparative advantage (RCA) values, respectively. The node size represents the absolute value of RCA. From Supplementary Figure 12 to 177, countries are ranked by GDP/capita (current US$, 2022).

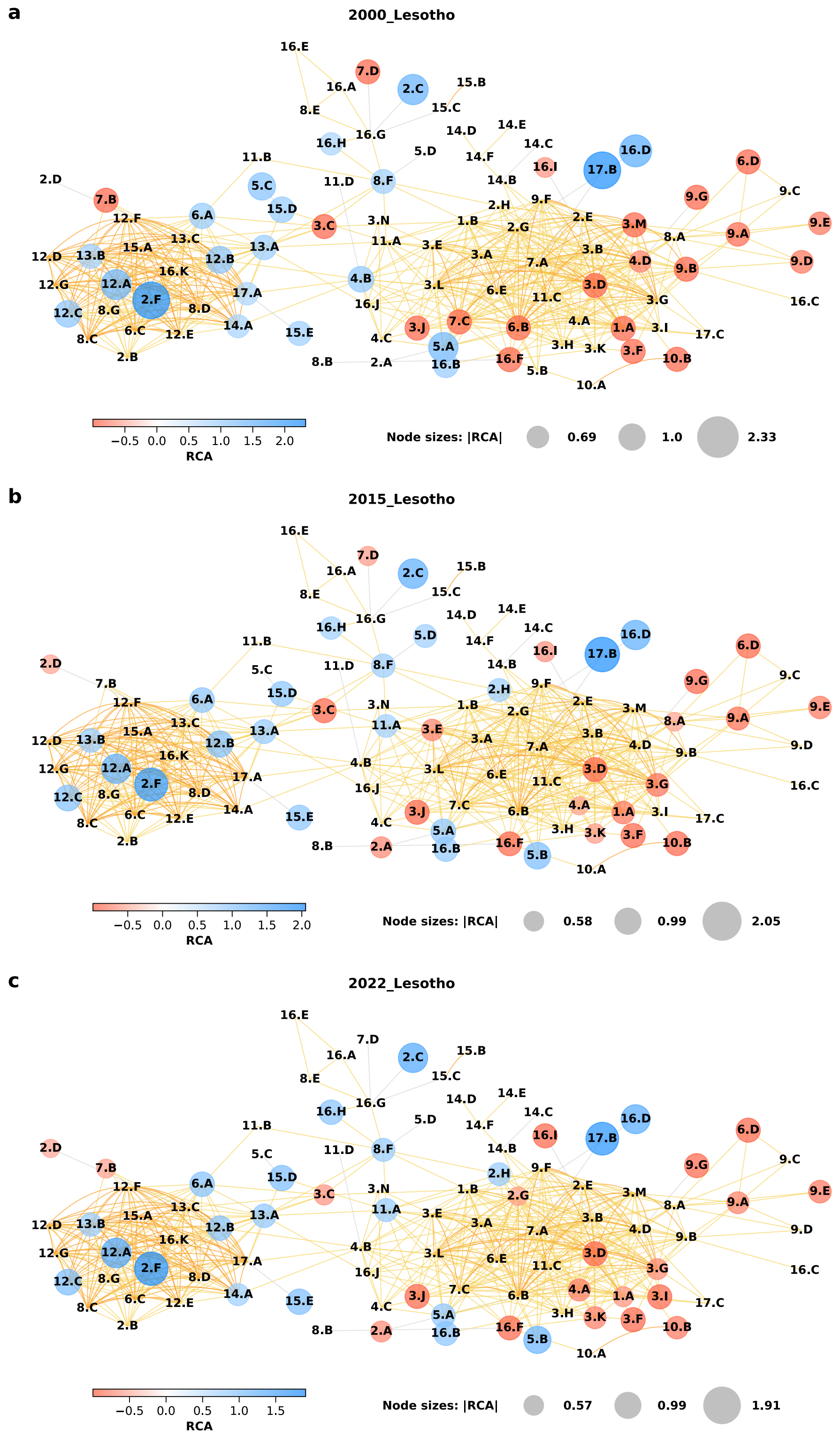

Supplementary Figure 151 | The SDG space of Lesotho. Panels **a, b, c,** The SDG space in 2000, 2015, and 2022. The nodes in blue and orange represent the top 20 and bottom 20 SDG indicators in revealed comparative advantage (RCA) values, respectively. The node size represents the absolute value of RCA. From Supplementary Figure 12 to 177, countries are ranked by GDP/capita (current US$, 2022).

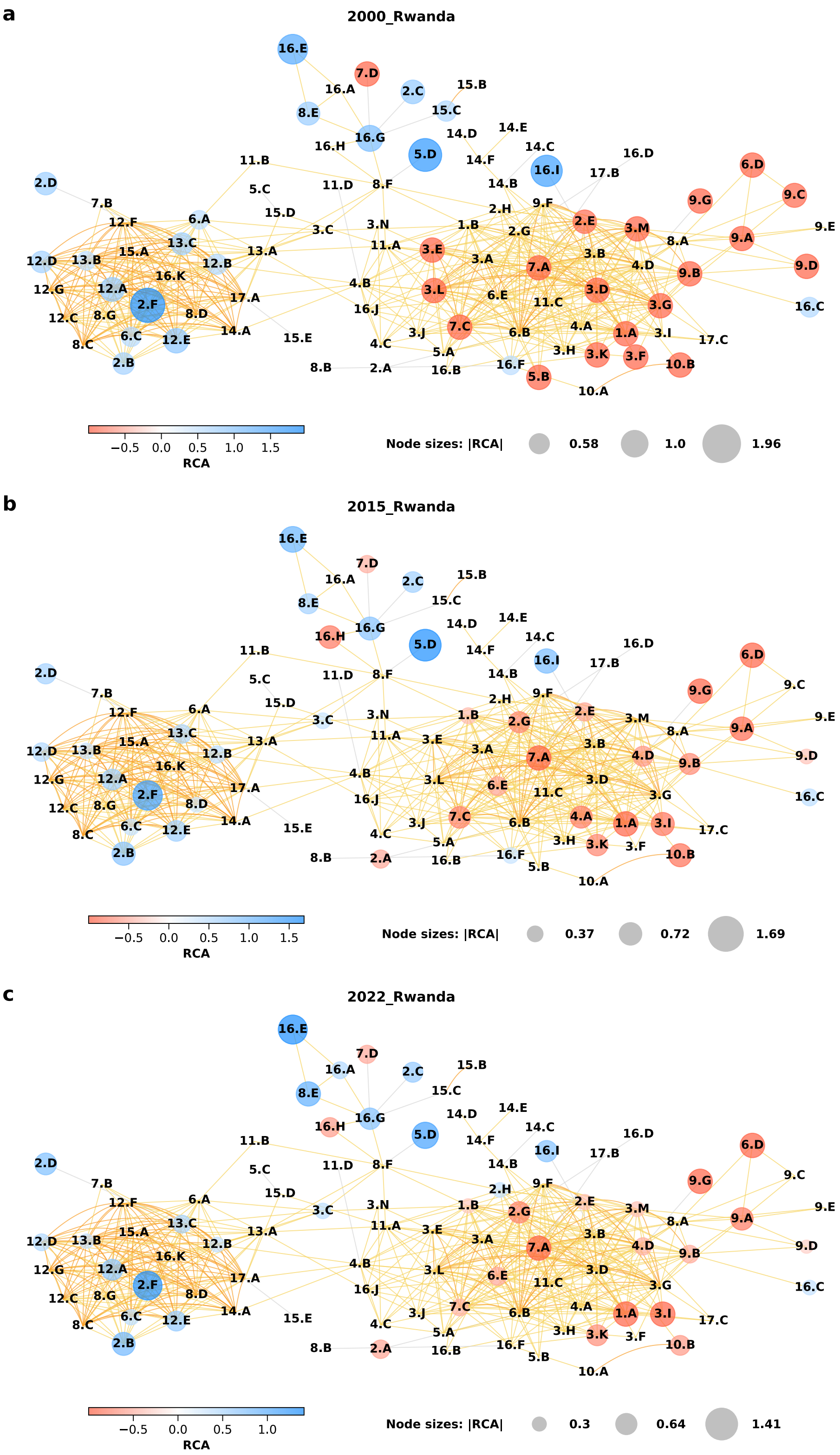

Supplementary Figure 152 | The SDG space of Rwanda. Panels **a, b, c,** The SDG space in 2000, 2015, and 2022. The nodes in blue and orange represent the top 20 and bottom 20 SDG indicators in revealed comparative advantage (RCA) values, respectively. The node size represents the absolute value of RCA. From Supplementary Figure 12 to 177, countries are ranked by GDP/capita (current US$, 2022).

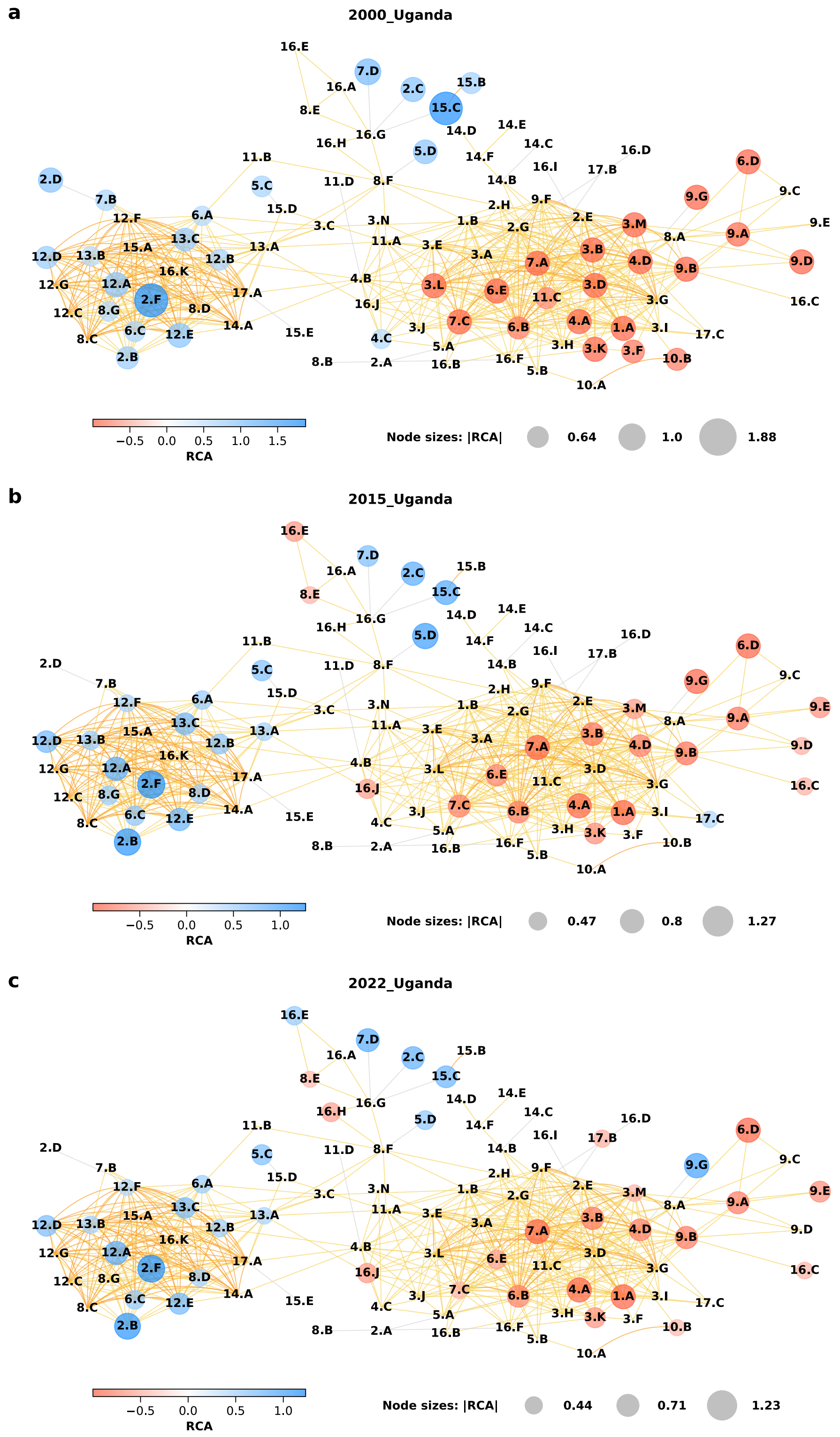

Supplementary Figure 153 | The SDG space of Uganda. Panels **a, b, c,** The SDG space in 2000, 2015, and 2022. The nodes in blue and orange represent the top 20 and bottom 20 SDG indicators in revealed comparative advantage (RCA) values, respectively. The node size represents the absolute value of RCA. From Supplementary Figure 12 to 177, countries are ranked by GDP/capita (current US$, 2022).

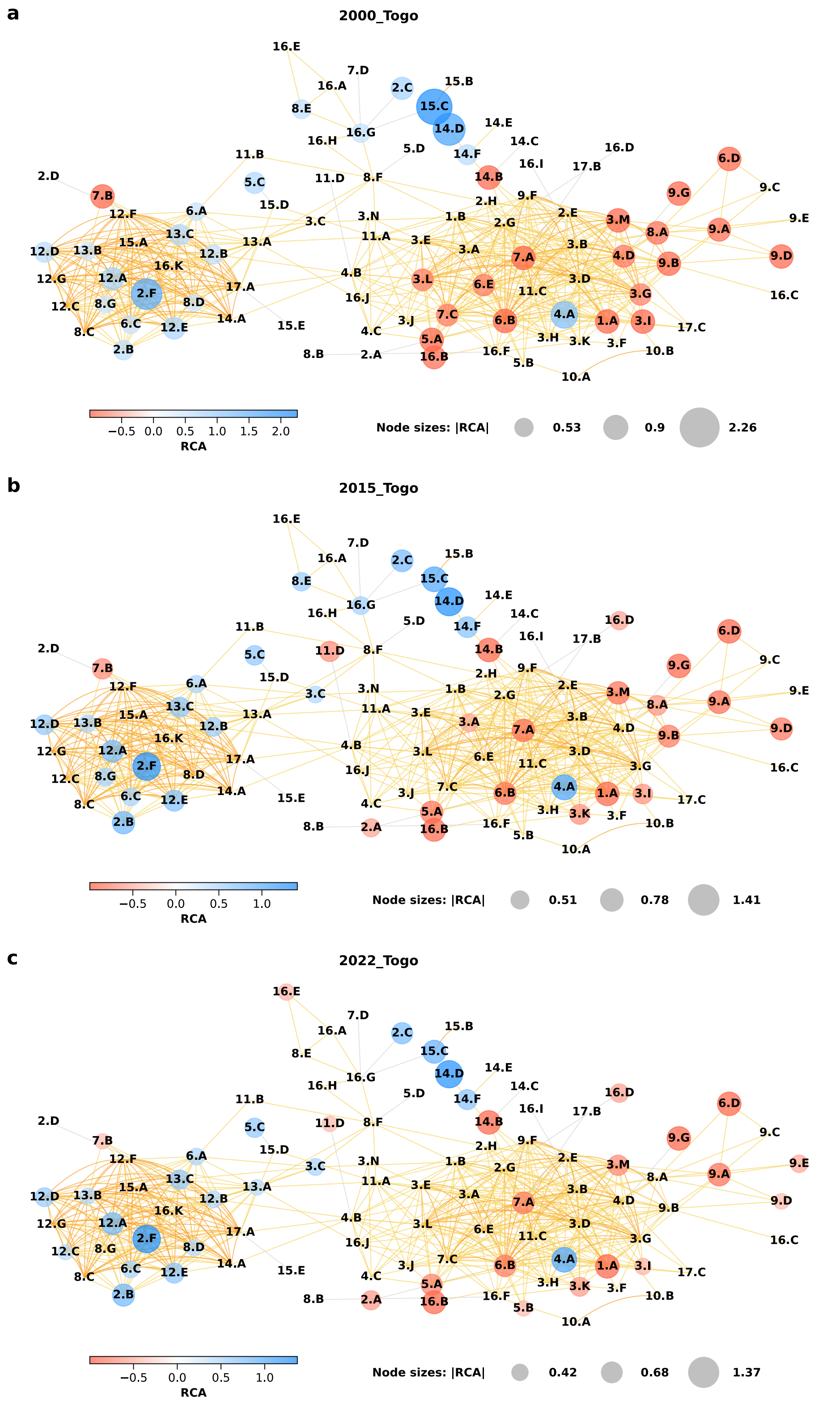

Supplementary Figure 154 | The SDG space of Togo. Panels **a, b, c,** The SDG space in 2000, 2015, and 2022. The nodes in blue and orange represent the top 20 and bottom 20 SDG indicators in revealed comparative advantage (RCA) values, respectively. The node size represents the absolute value of RCA. From Supplementary Figure 12 to 177, countries are ranked by GDP/capita (current US$, 2022).

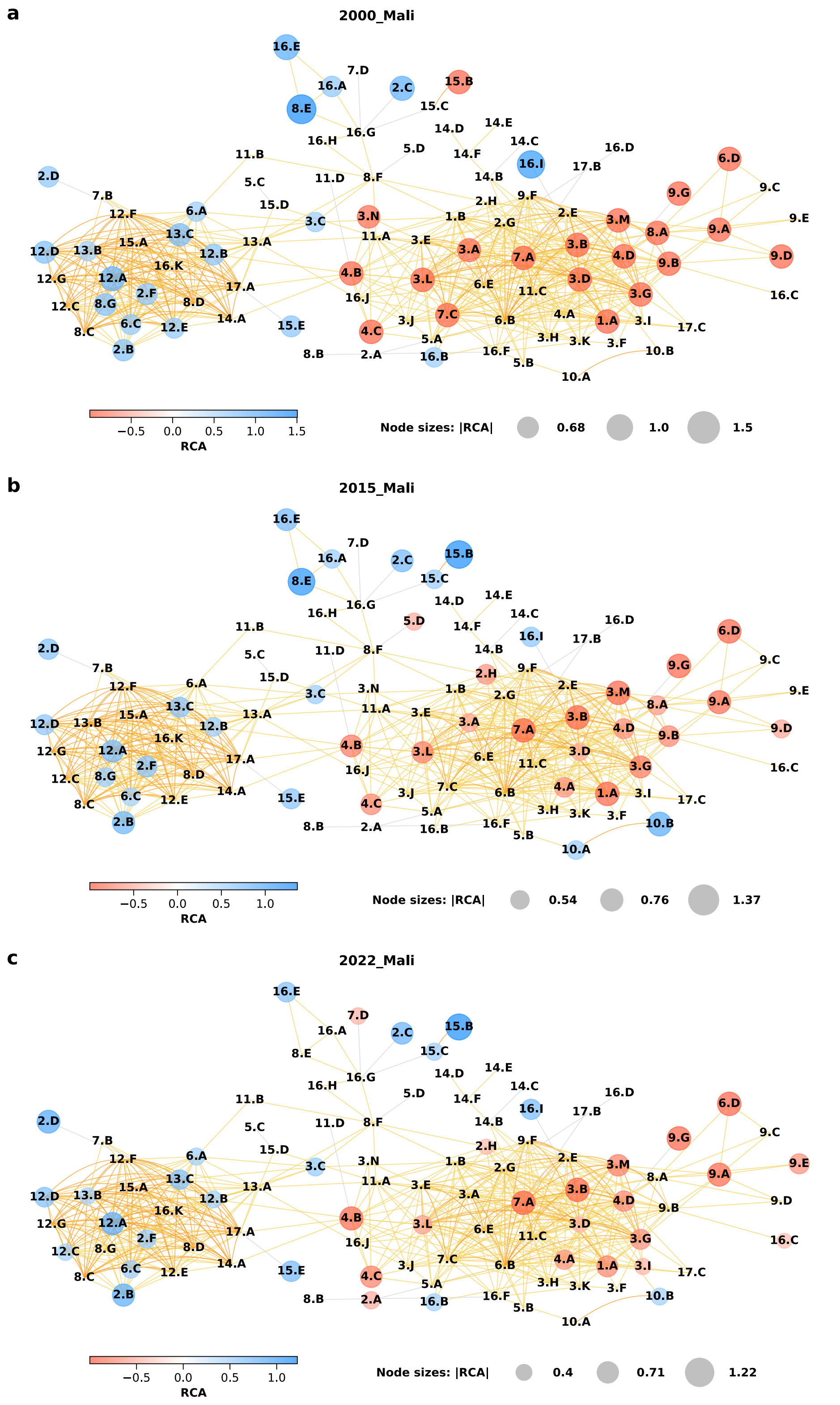

Supplementary Figure 155 | The SDG space of Mali. Panels **a, b, c,** The SDG space in 2000, 2015, and 2022. The nodes in blue and orange represent the top 20 and bottom 20 SDG indicators in revealed comparative advantage (RCA) values, respectively. The node size represents the absolute value of RCA. From Supplementary Figure 12 to 177, countries are ranked by GDP/capita (current US$, 2022).

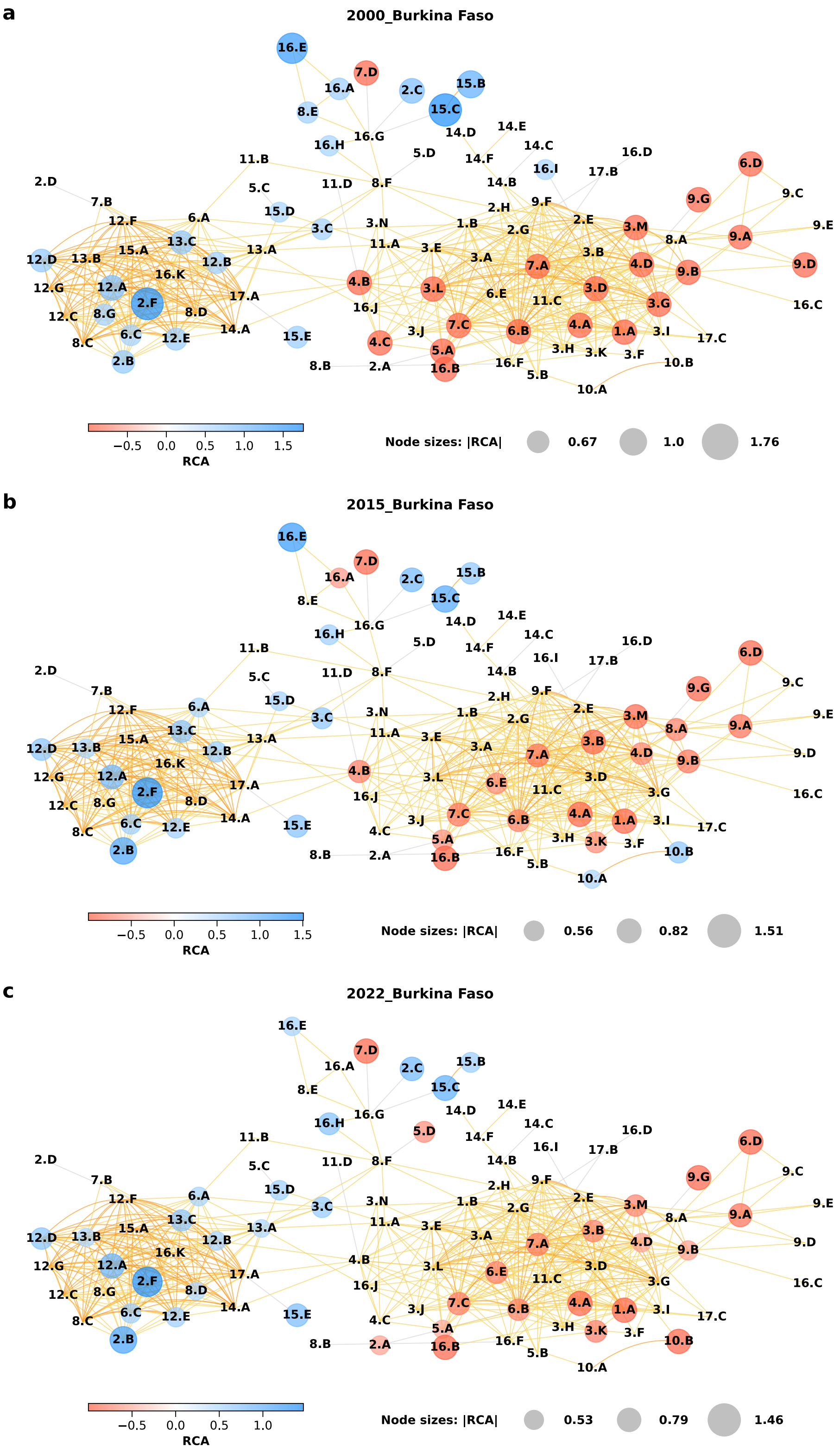

Supplementary Figure 155 | The SDG space of Burkina Faso. Panels **a, b, c,** The SDG space in 2000, 2015, and 2022. The nodes in blue and orange represent the top 20 and bottom 20 SDG indicators in revealed comparative advantage (RCA) values, respectively. The node size represents the absolute value of RCA. From Supplementary Figure 12 to 177, countries are ranked by GDP/capita (current US$, 2022).

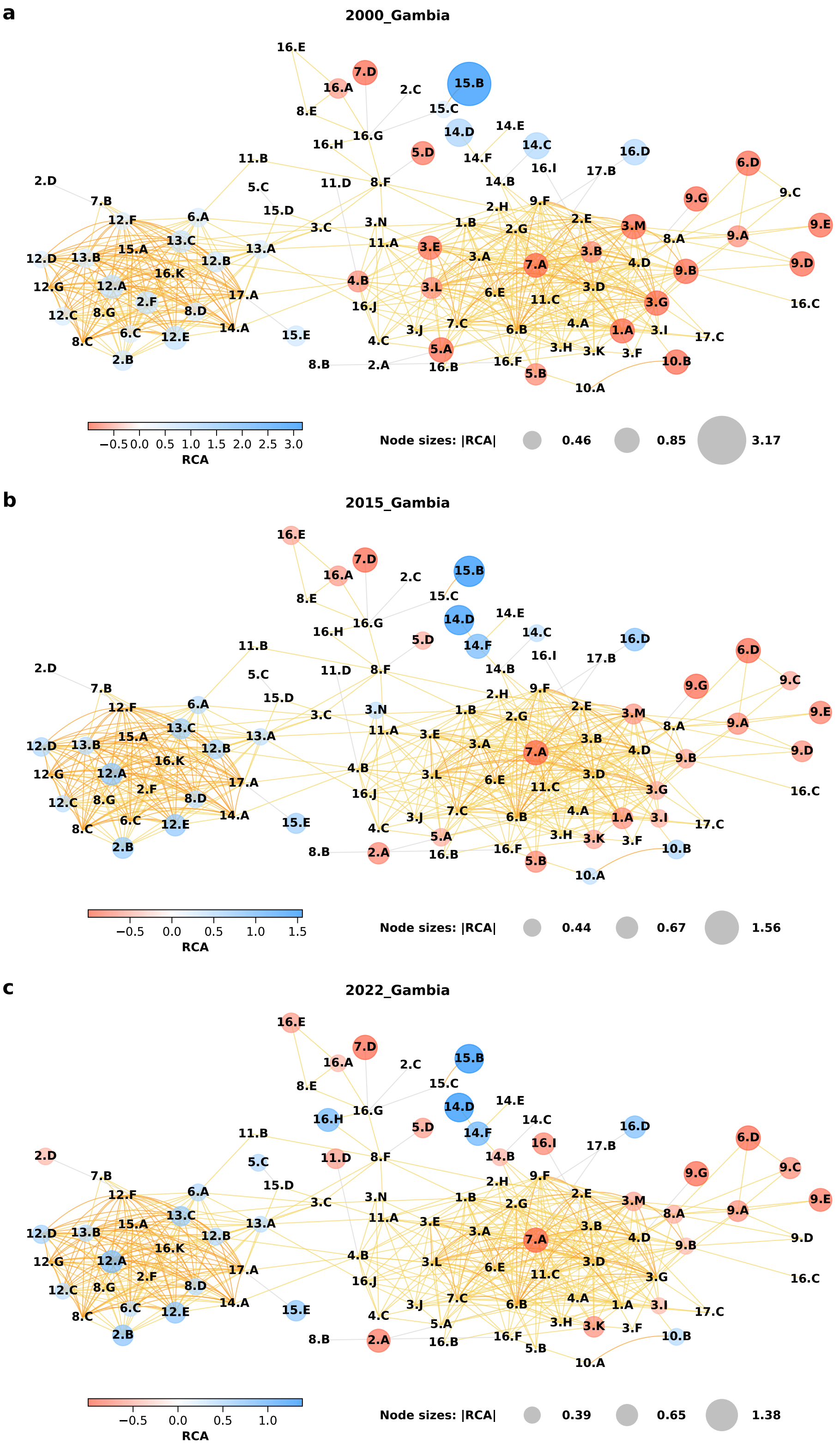

Supplementary Figure 157 | The SDG space of Gambia. Panels **a, b, c,** The SDG space in 2000, 2015, and 2022. The nodes in blue and orange represent the top 20 and bottom 20 SDG indicators in revealed comparative advantage (RCA) values, respectively. The node size represents the absolute value of RCA. From Supplementary Figure 12 to 177, countries are ranked by GDP/capita (current US$, 2022).

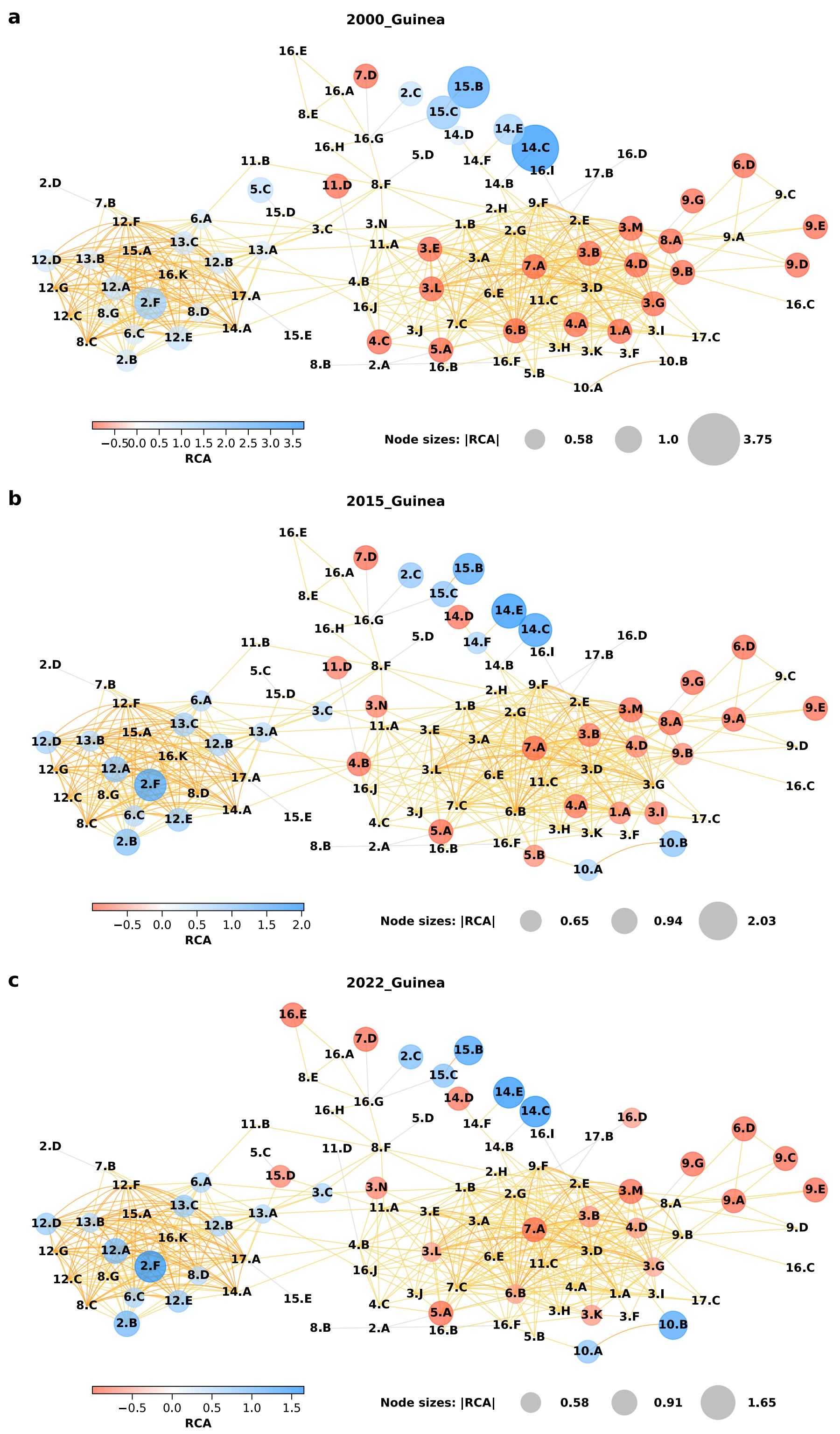

Supplementary Figure 158 | The SDG space of Guinea. Panels **a, b, c,** The SDG space in 2000, 2015, and 2022. The nodes in blue and orange represent the top 20 and bottom 20 SDG indicators in revealed comparative advantage (RCA) values, respectively. The node size represents the absolute value of RCA. From Supplementary Figure 12 to 177, countries are ranked by GDP/capita (current US$, 2022).

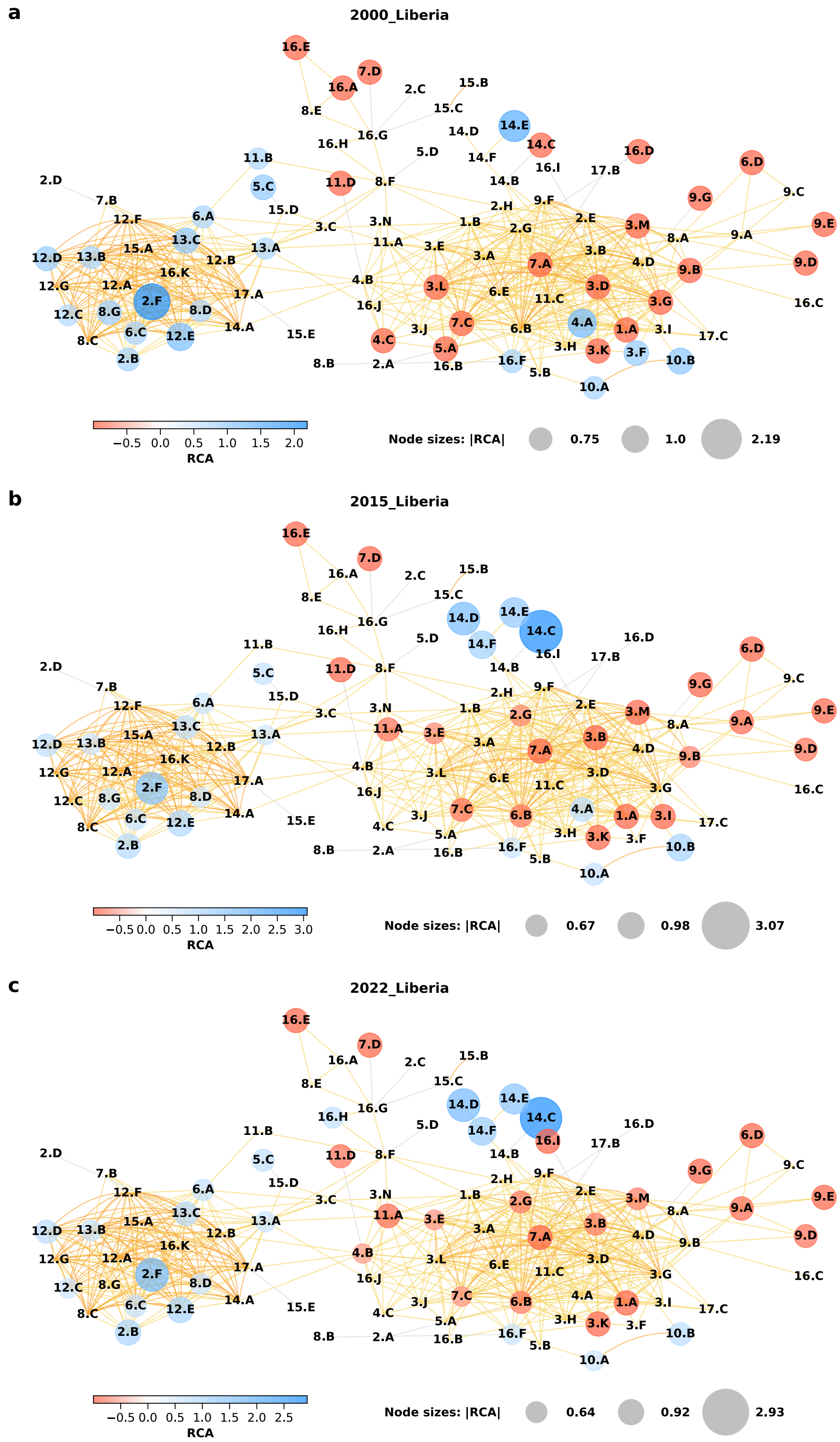

Supplementary Figure 159 | The SDG space of Liberia. Panels **a, b, c,** The SDG space in 2000, 2015, and 2022. The nodes in blue and orange represent the top 20 and bottom 20 SDG indicators in revealed comparative advantage (RCA) values, respectively. The node size represents the absolute value of RCA. From Supplementary Figure 12 to 177, countries are ranked by GDP/capita (current US$, 2022).

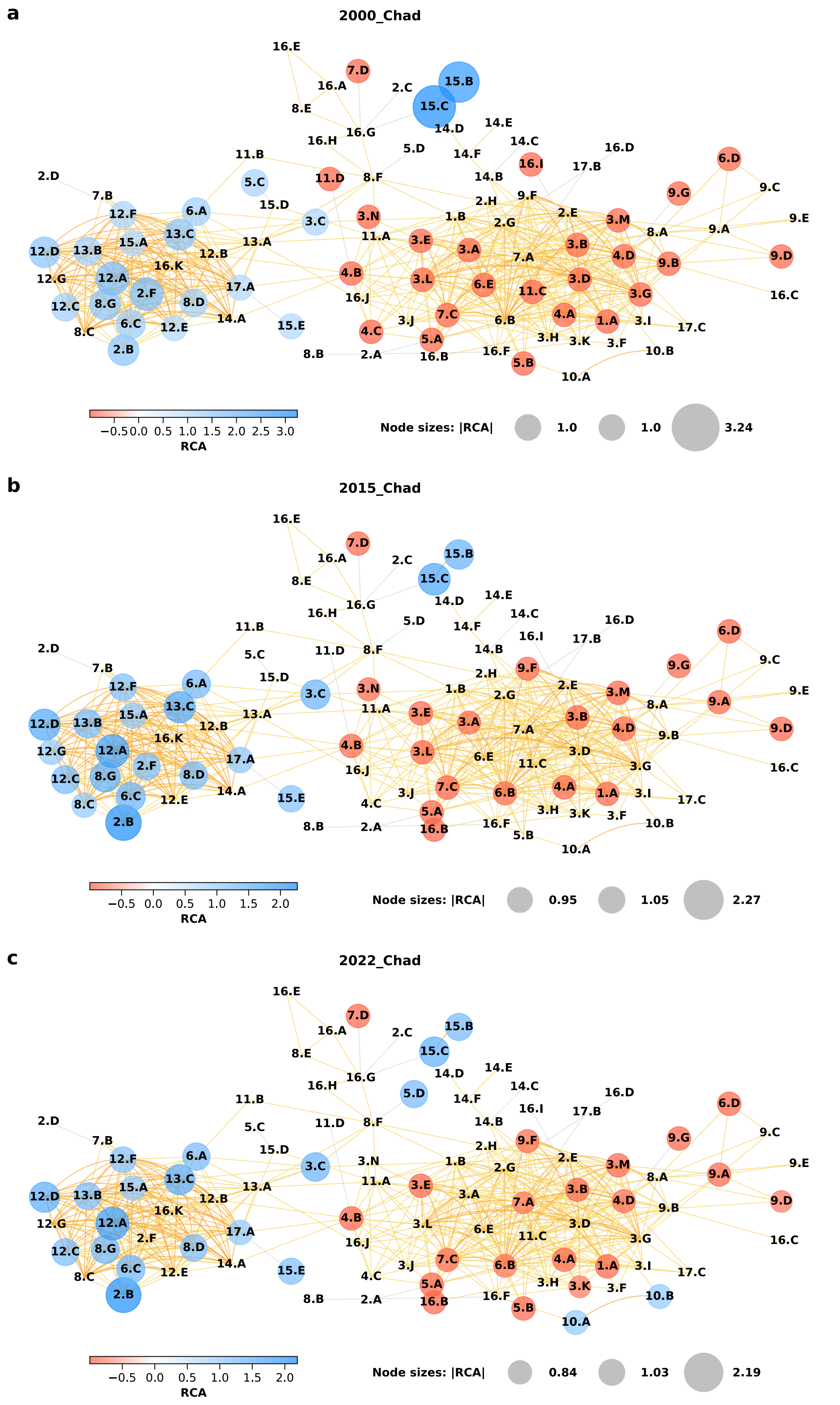

Supplementary Figure 160 | The SDG space of Chad. Panels **a, b, c,** The SDG space in 2000, 2015, and 2022. The nodes in blue and orange represent the top 20 and bottom 20 SDG indicators in revealed comparative advantage (RCA) values, respectively. The node size represents the absolute value of RCA. From Supplementary Figure 12 to 177, countries are ranked by GDP/capita (current US$, 2022).

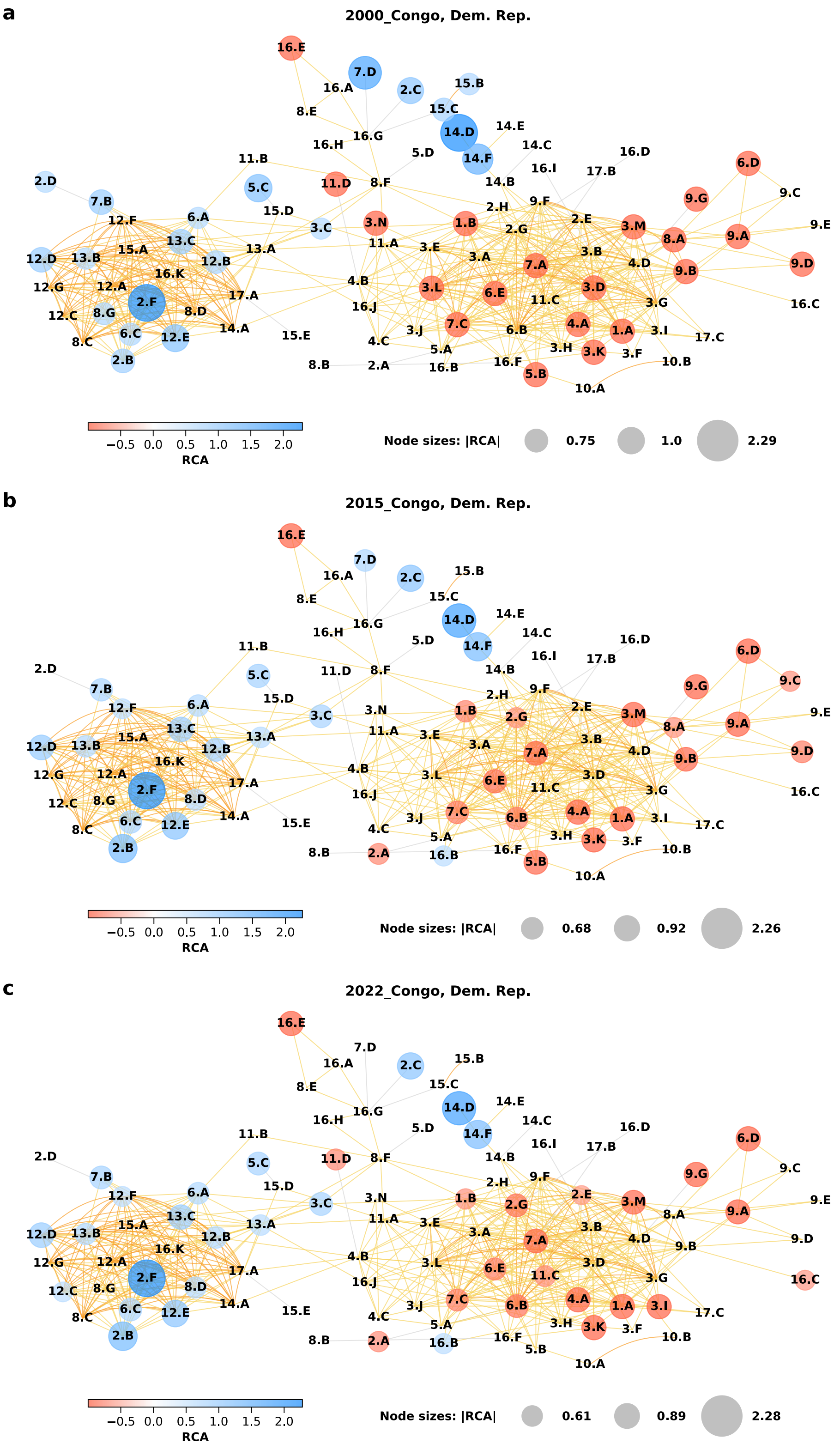

Supplementary Figure 161 | The SDG space of Congo, Dem. Rep.. Panels **a, b, c,** The SDG space in 2000, 2015, and 2022. The nodes in blue and orange represent the top 20 and bottom 20 SDG indicators in revealed comparative advantage (RCA) values, respectively. The node size represents the absolute value of RCA. From Supplementary Figure 12 to 177, countries are ranked by GDP/capita (current US$, 2022).

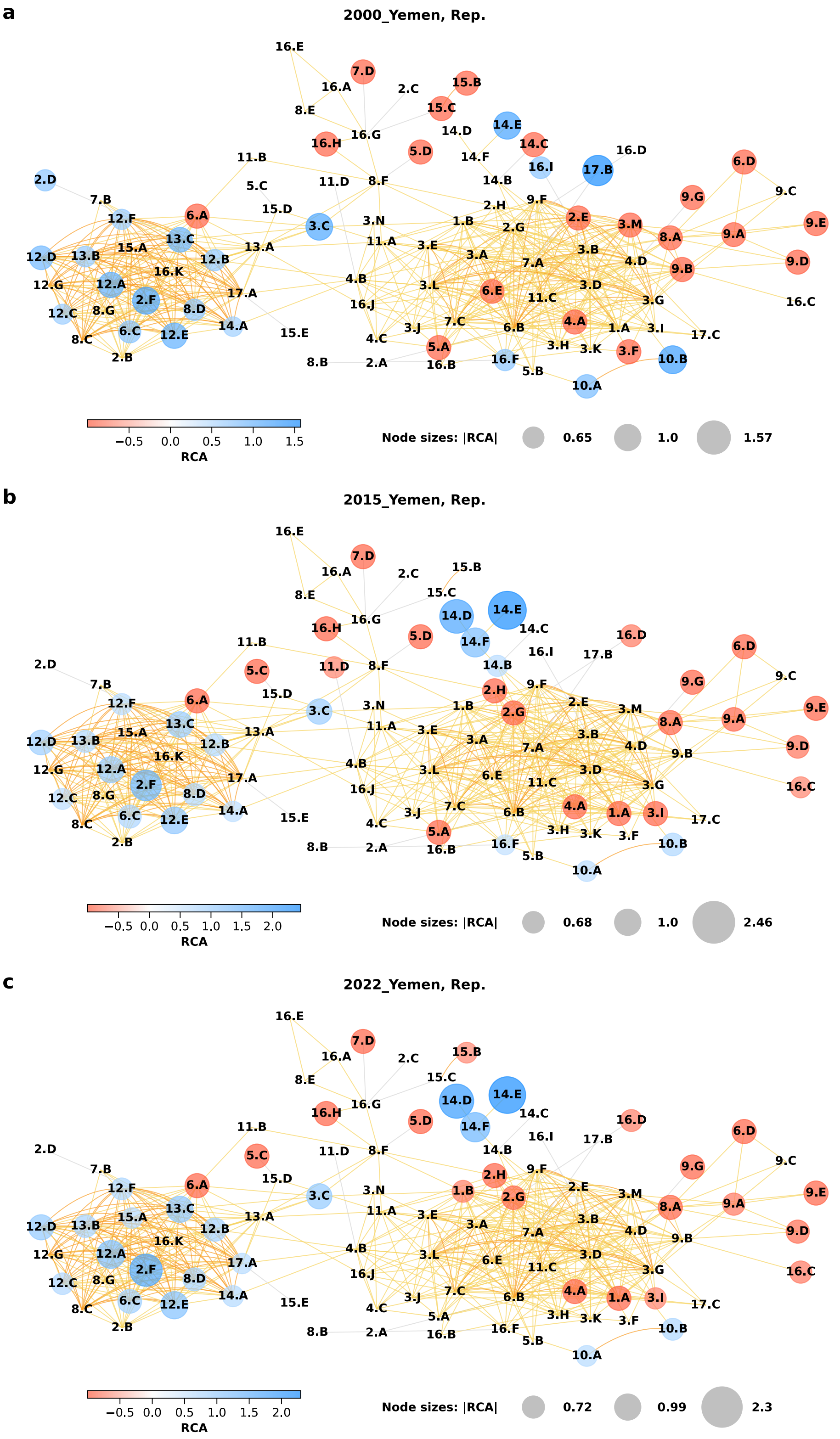

Supplementary Figure 162 | The SDG space of Yemen, Rep.. Panels **a, b, c,** The SDG space in 2000, 2015, and 2022. The nodes in blue and orange represent the top 20 and bottom 20 SDG indicators in revealed comparative advantage (RCA) values, respectively. The node size represents the absolute value of RCA. From Supplementary Figure 12 to 177, countries are ranked by GDP/capita (current US$, 2022).

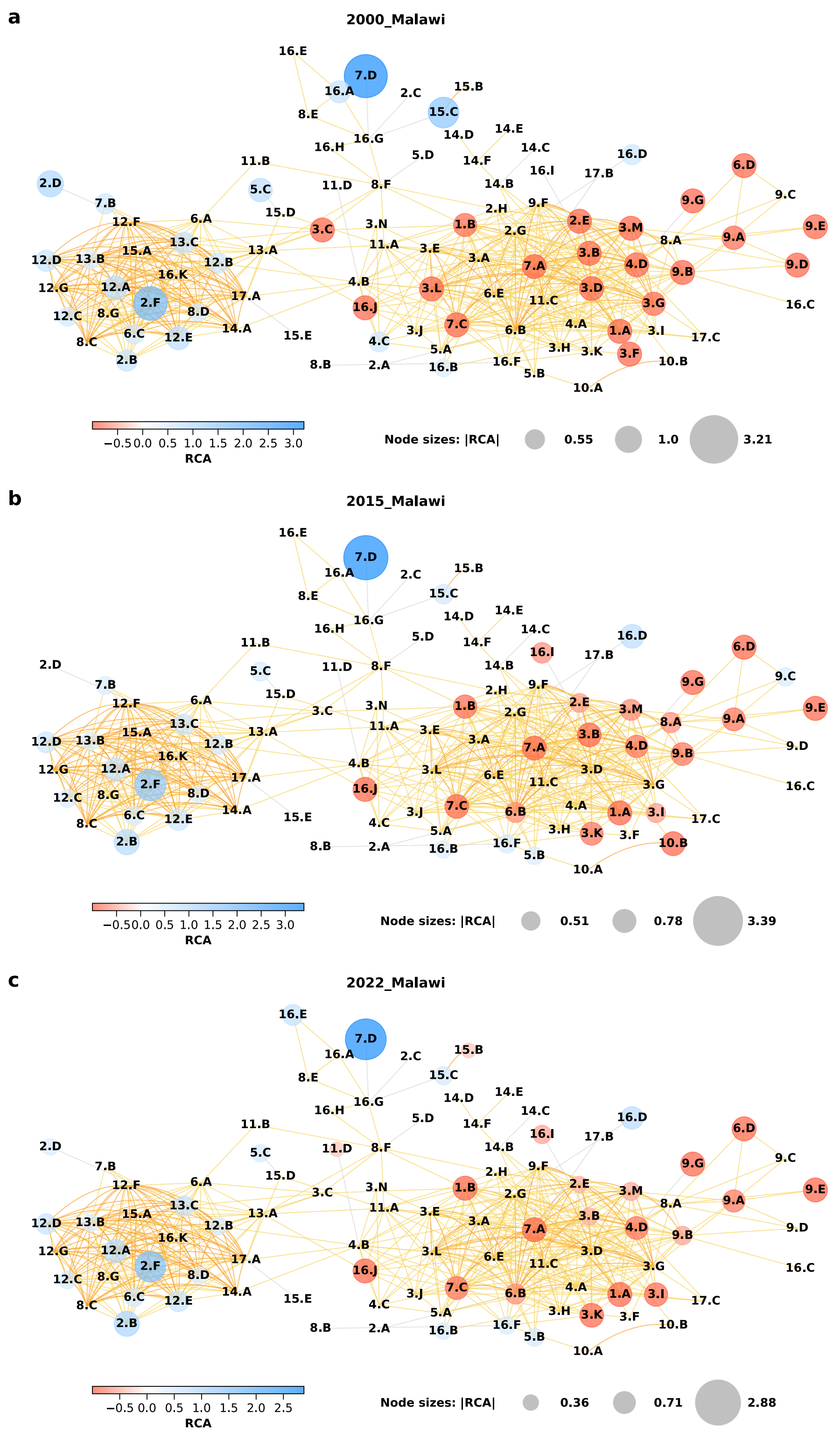

Supplementary Figure 163 | The SDG space of Malawi. Panels **a, b, c,** The SDG space in 2000, 2015, and 2022. The nodes in blue and orange represent the top 20 and bottom 20 SDG indicators in revealed comparative advantage (RCA) values, respectively. The node size represents the absolute value of RCA. From Supplementary Figure 12 to 177, countries are ranked by GDP/capita (current US$, 2022).

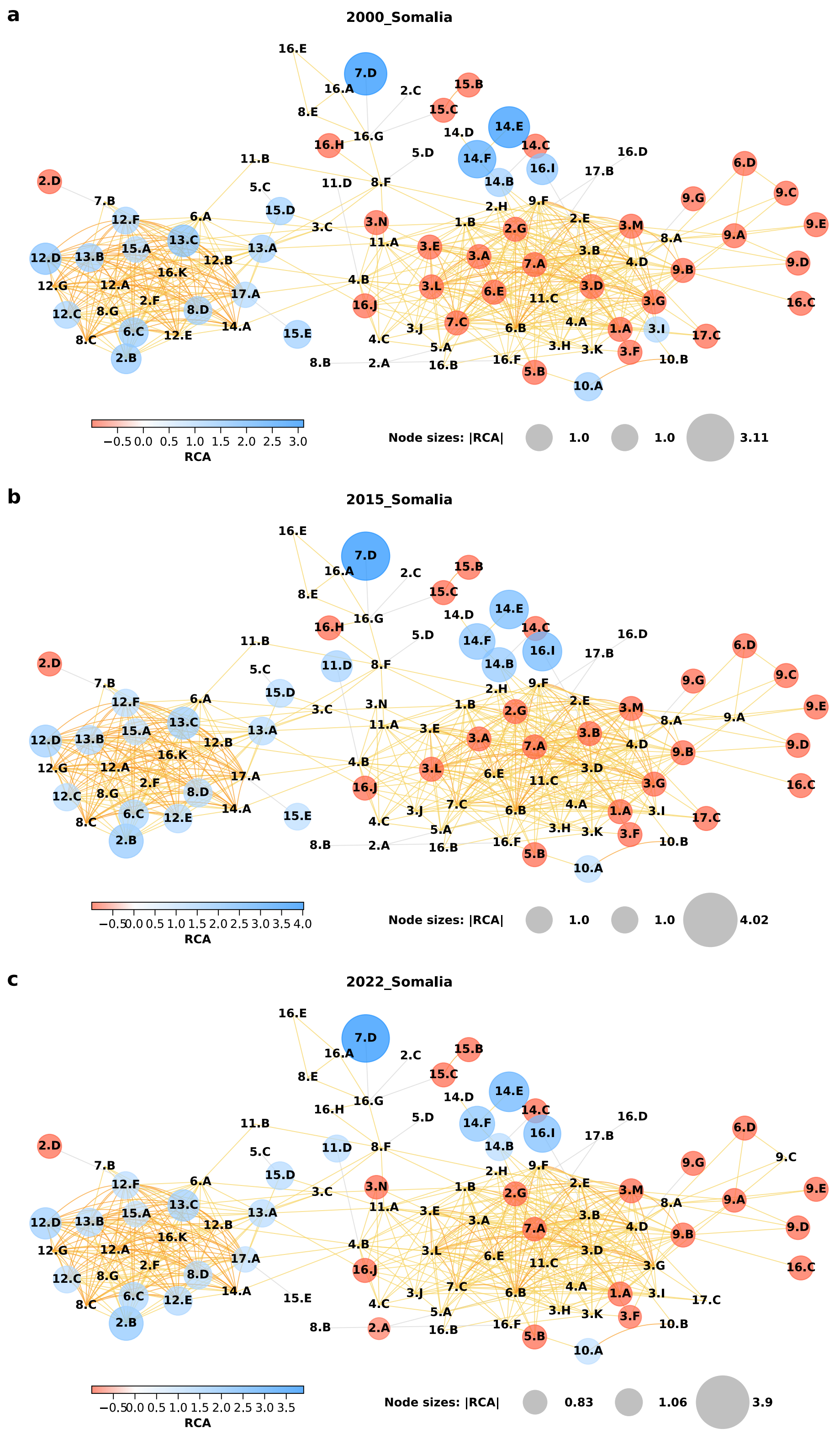

Supplementary Figure 164 | The SDG space of Somalia. Panels **a, b, c,** The SDG space in 2000, 2015, and 2022. The nodes in blue and orange represent the top 20 and bottom 20 SDG indicators in revealed comparative advantage (RCA) values, respectively. The node size represents the absolute value of RCA. From Supplementary Figure 12 to 177, countries are ranked by GDP/capita (current US$, 2022).

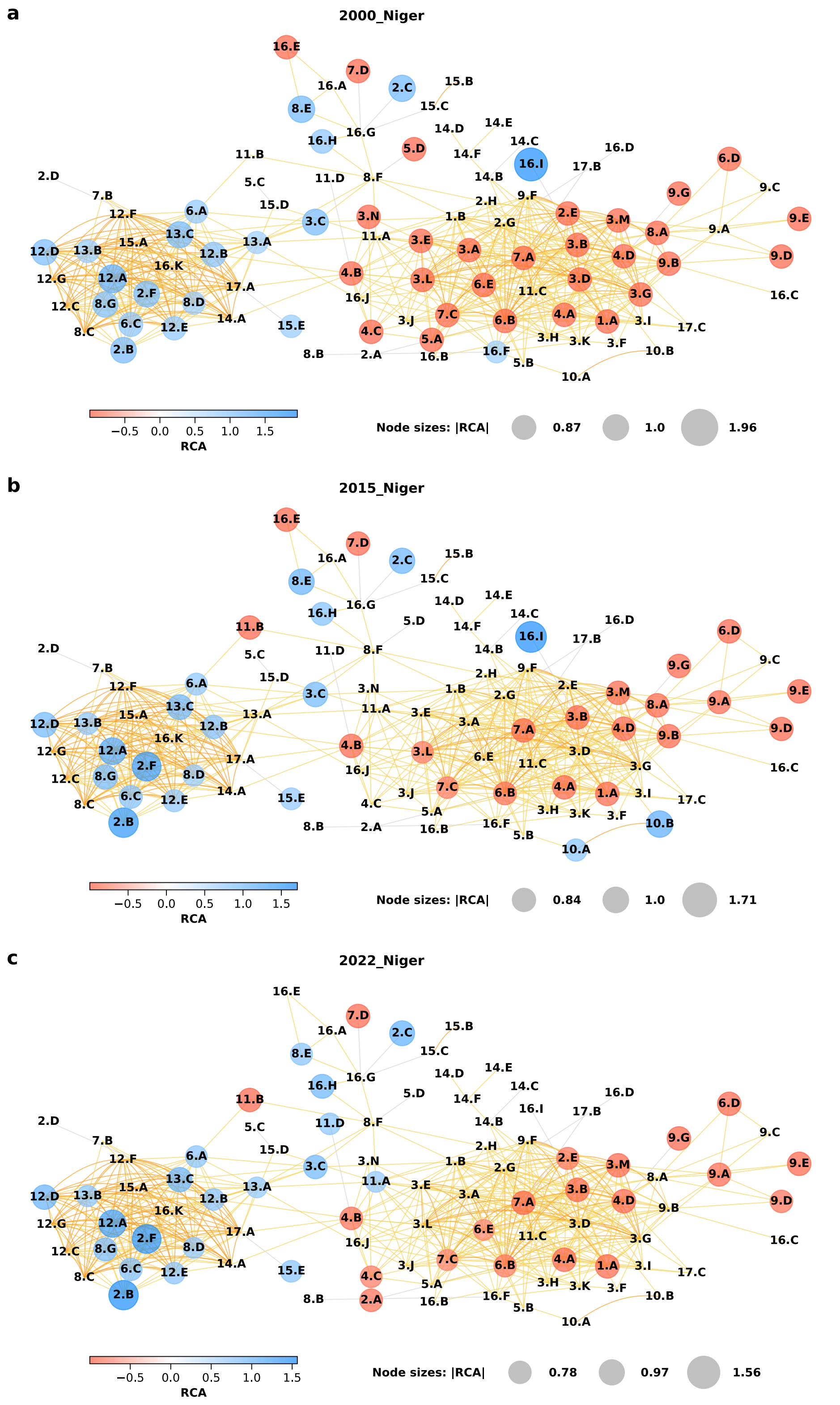

Supplementary Figure 165 | The SDG space of Niger. Panels **a, b, c,** The SDG space in 2000, 2015, and 2022. The nodes in blue and orange represent the top 20 and bottom 20 SDG indicators in revealed comparative advantage (RCA) values, respectively. The node size represents the absolute value of RCA. From Supplementary Figure 12 to 177, countries are ranked by GDP/capita (current US$, 2022).

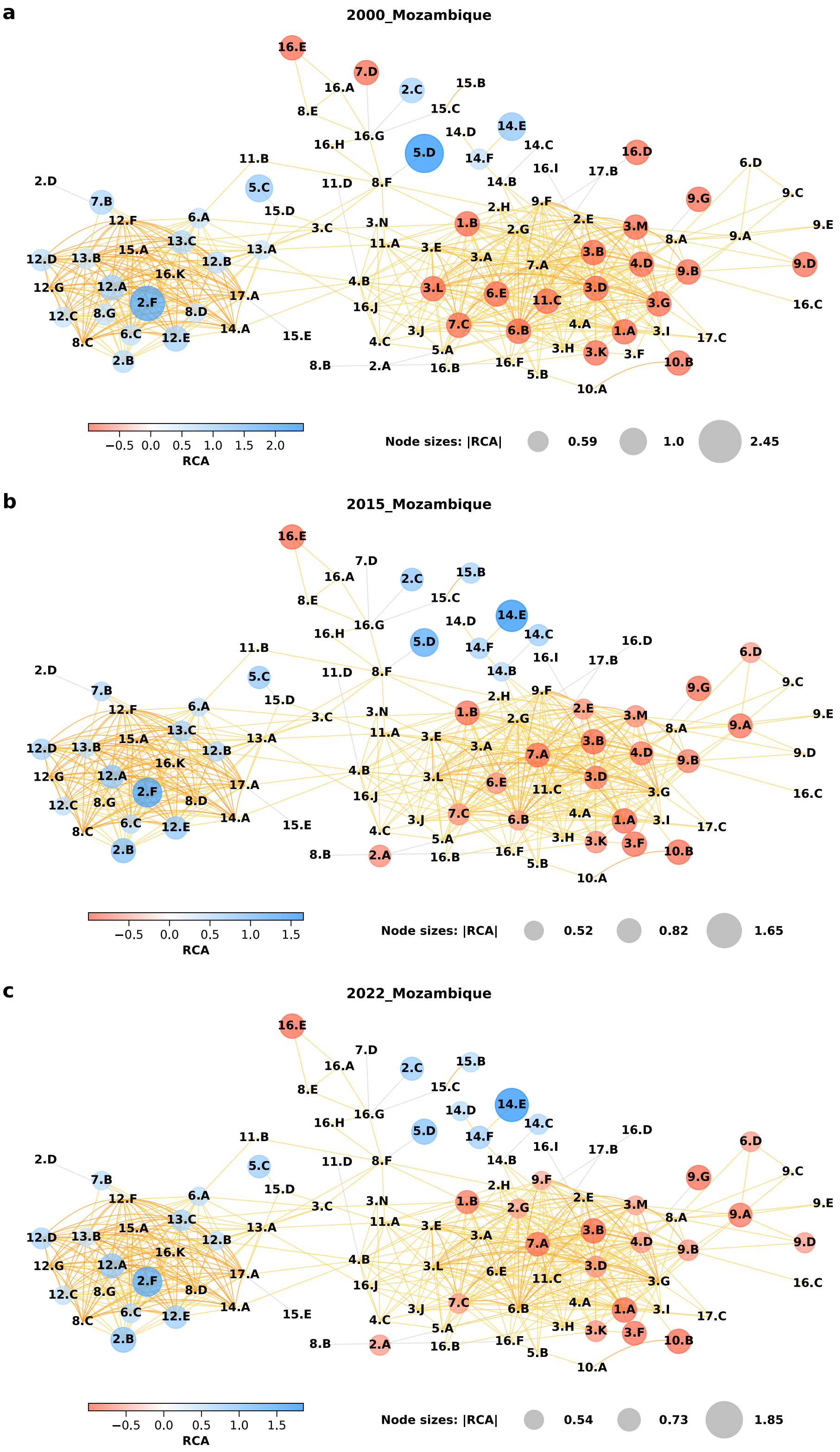

Supplementary Figure 166 | The SDG space of Mozambique. Panels **a, b, c,** The SDG space in 2000, 2015, and 2022. The nodes in blue and orange represent the top 20 and bottom 20 SDG indicators in revealed comparative advantage (RCA) values, respectively. The node size represents the absolute value of RCA. From Supplementary Figure 12 to 177, countries are ranked by GDP/capita (current US$, 2022).

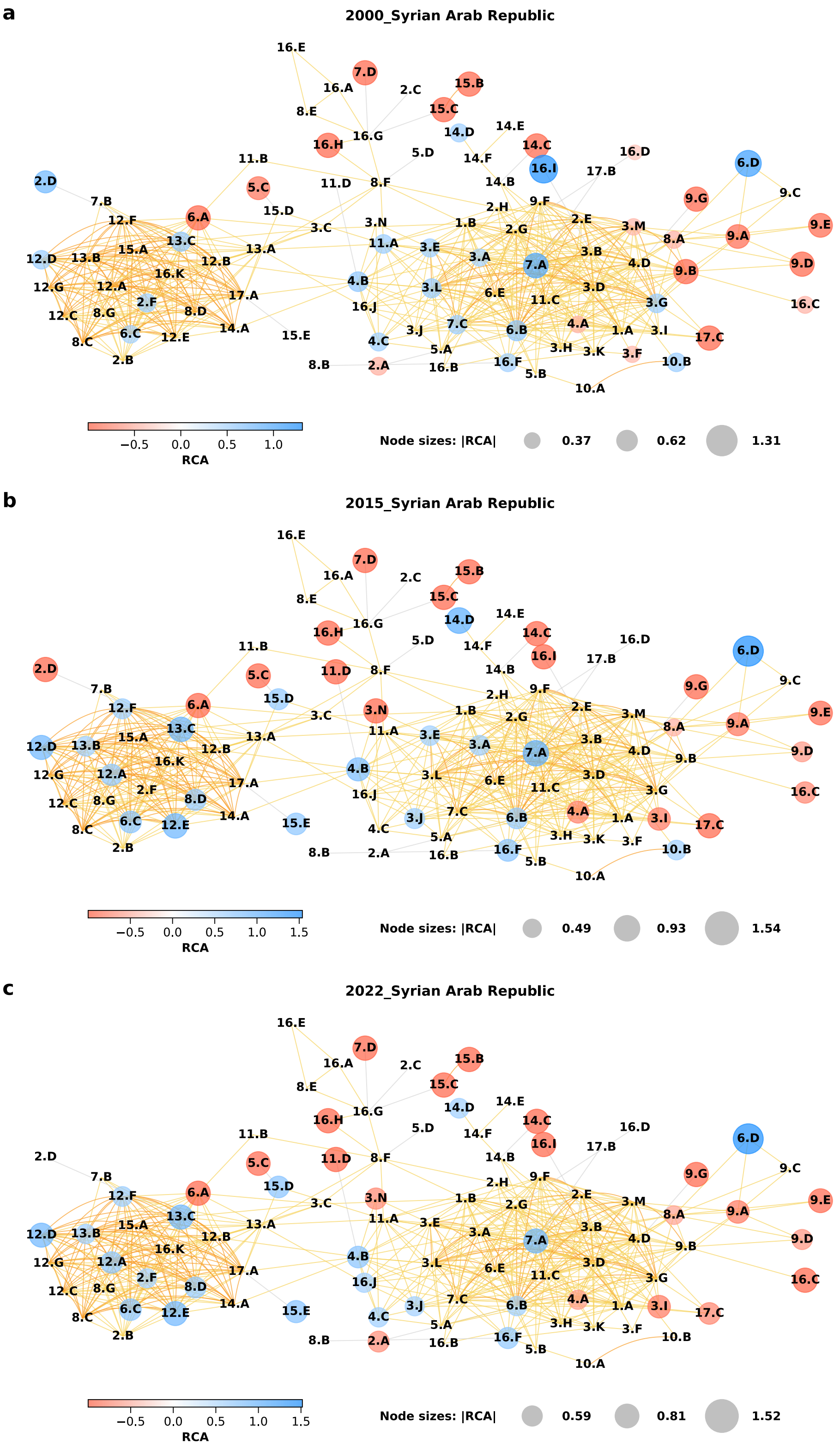

Supplementary Figure 167 | The SDG space of Syrian Arab Republic. Panels **a, b, c,** The SDG space in 2000, 2015, and 2022. The nodes in blue and orange represent the top 20 and bottom 20 SDG indicators in revealed comparative advantage (RCA) values, respectively. The node size represents the absolute value of RCA. From Supplementary Figure 12 to 177, countries are ranked by GDP/capita (current US$, 2022).

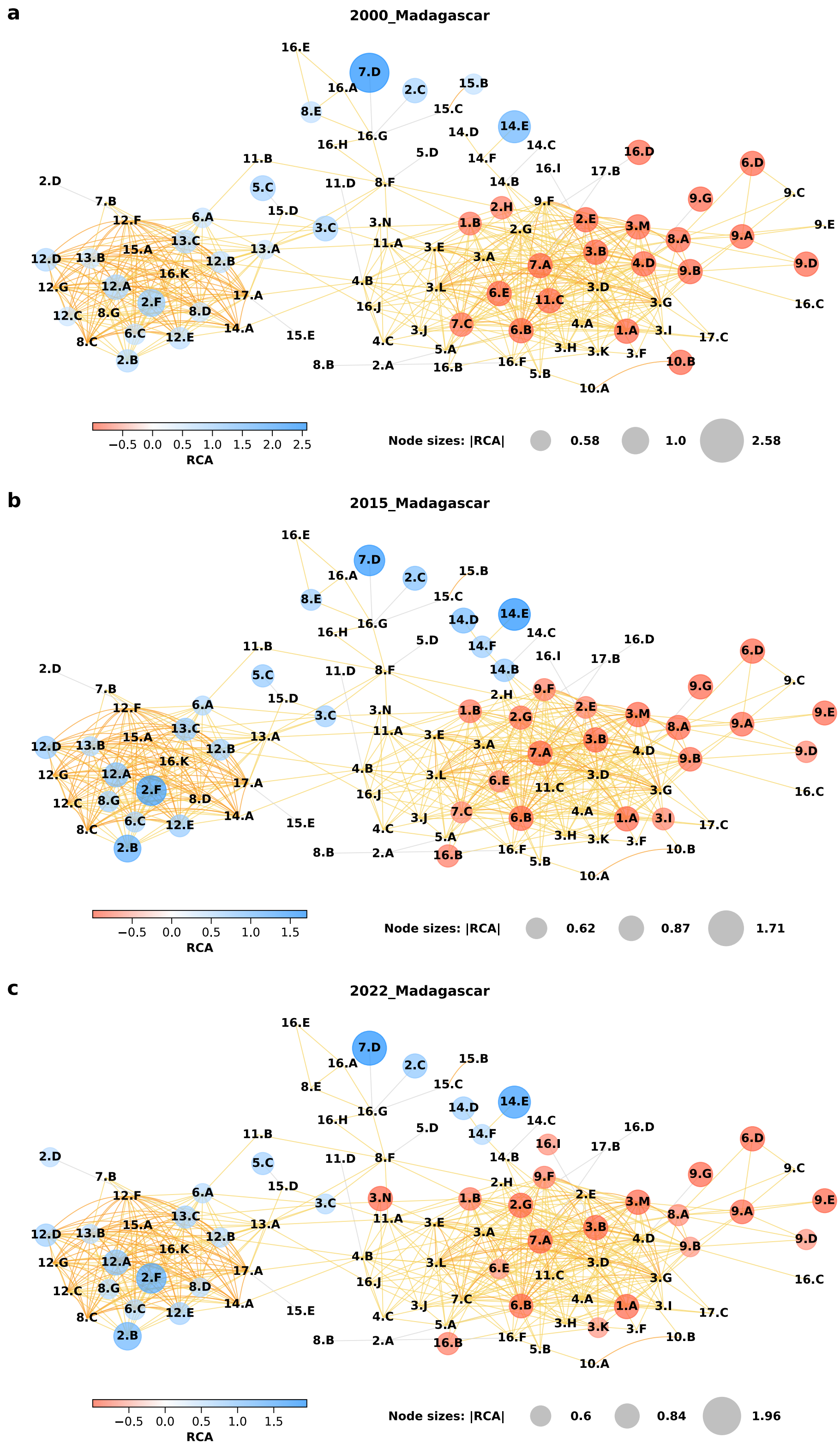

Supplementary Figure 168 | The SDG space of Madagascar. Panels **a, b, c,** The SDG space in 2000, 2015, and 2022. The nodes in blue and orange represent the top 20 and bottom 20 SDG indicators in revealed comparative advantage (RCA) values, respectively. The node size represents the absolute value of RCA. From Supplementary Figure 12 to 177, countries are ranked by GDP/capita (current US$, 2022).

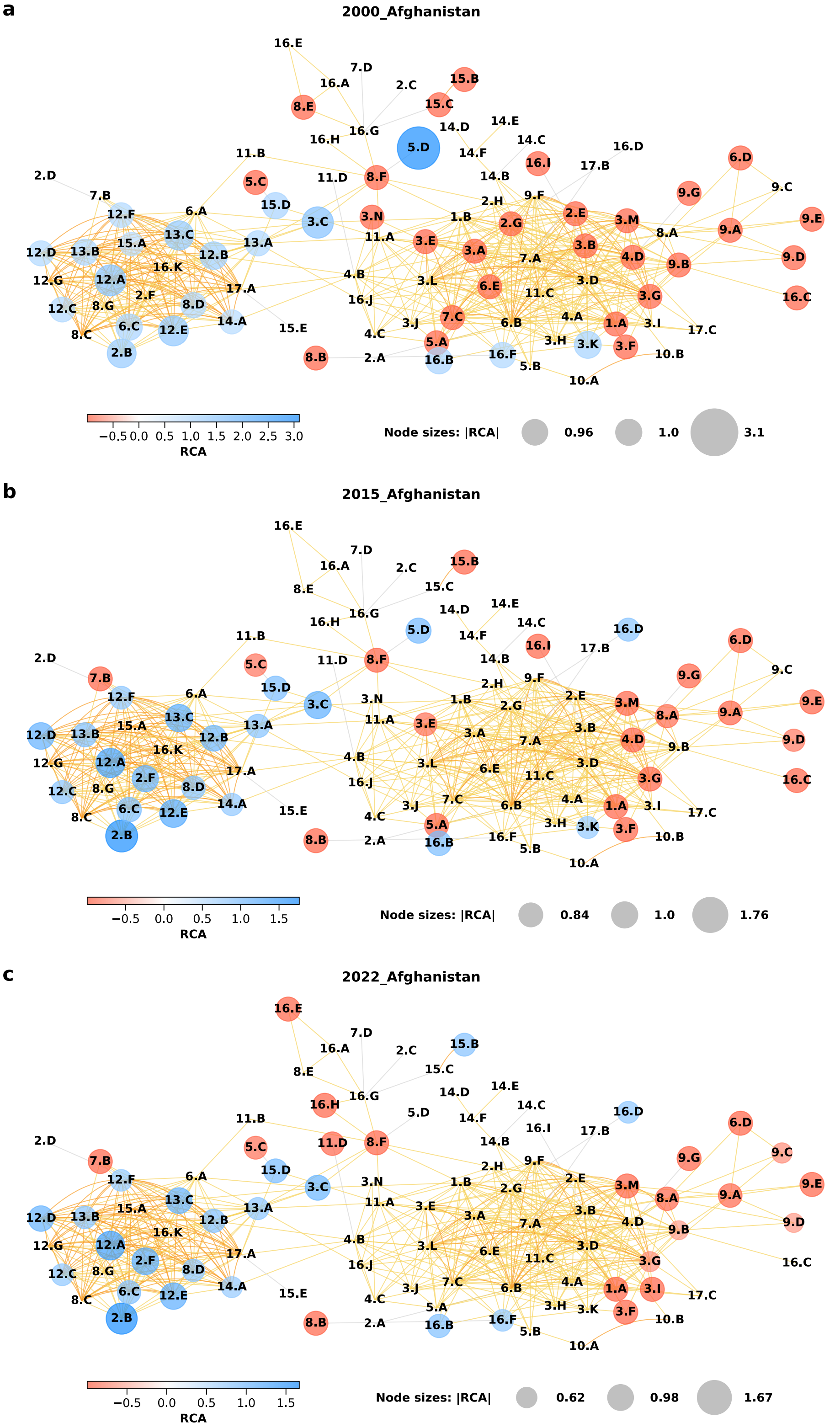

Supplementary Figure 169 | The SDG space of Afghanistan. Panels **a, b, c,** The SDG space in 2000, 2015, and 2022. The nodes in blue and orange represent the top 20 and bottom 20 SDG indicators in revealed comparative advantage (RCA) values, respectively. The node size represents the absolute value of RCA. From Supplementary Figure 12 to 177, countries are ranked by GDP/capita (current US$, 2022).

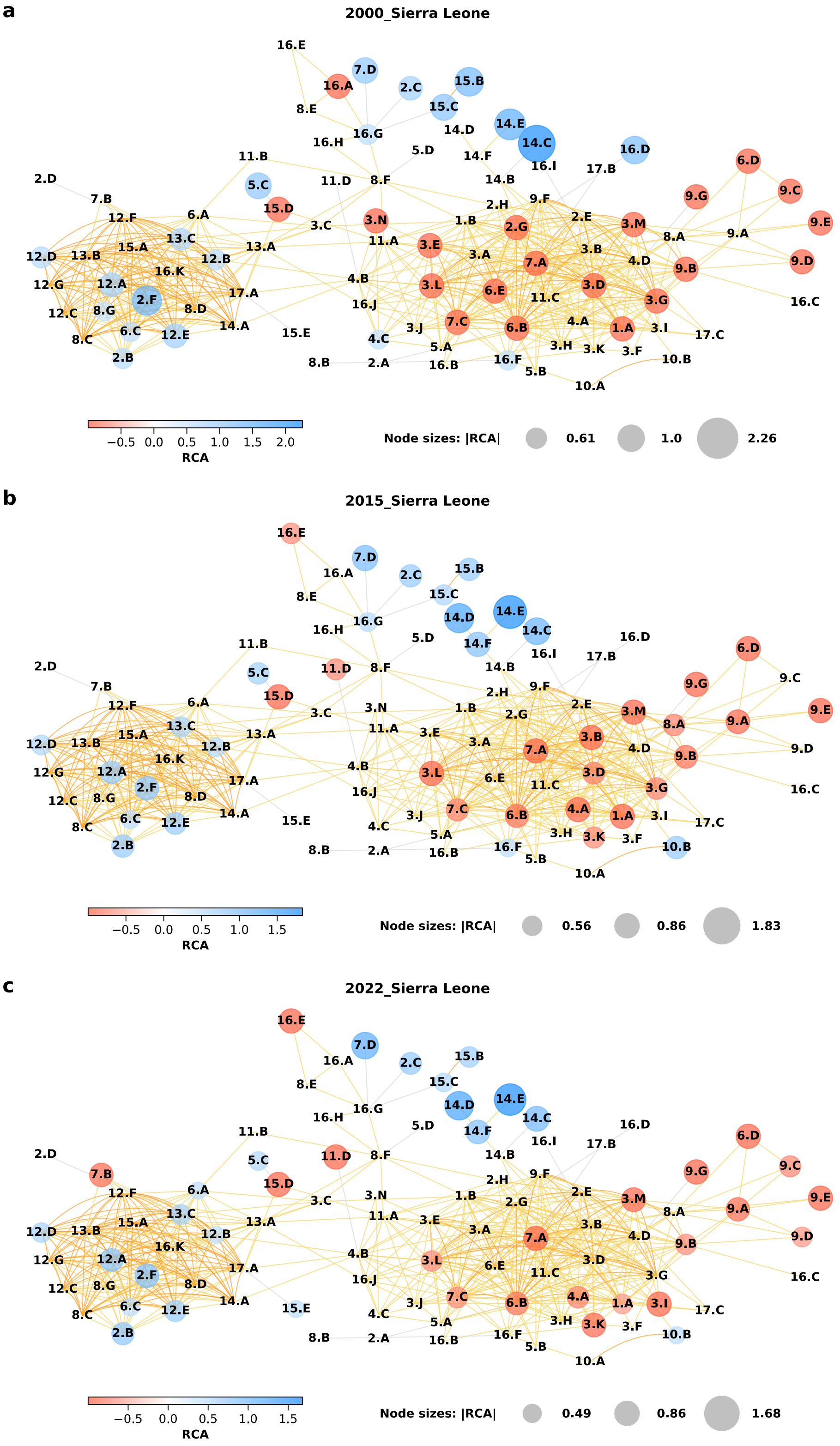

Supplementary Figure 170 | The SDG space of Sierra Leone. Panels **a, b, c,** The SDG space in 2000, 2015, and 2022. The nodes in blue and orange represent the top 20 and bottom 20 SDG indicators in revealed comparative advantage (RCA) values, respectively. The node size represents the absolute value of RCA. From Supplementary Figure 12 to 177, countries are ranked by GDP/capita (current US$, 2022).

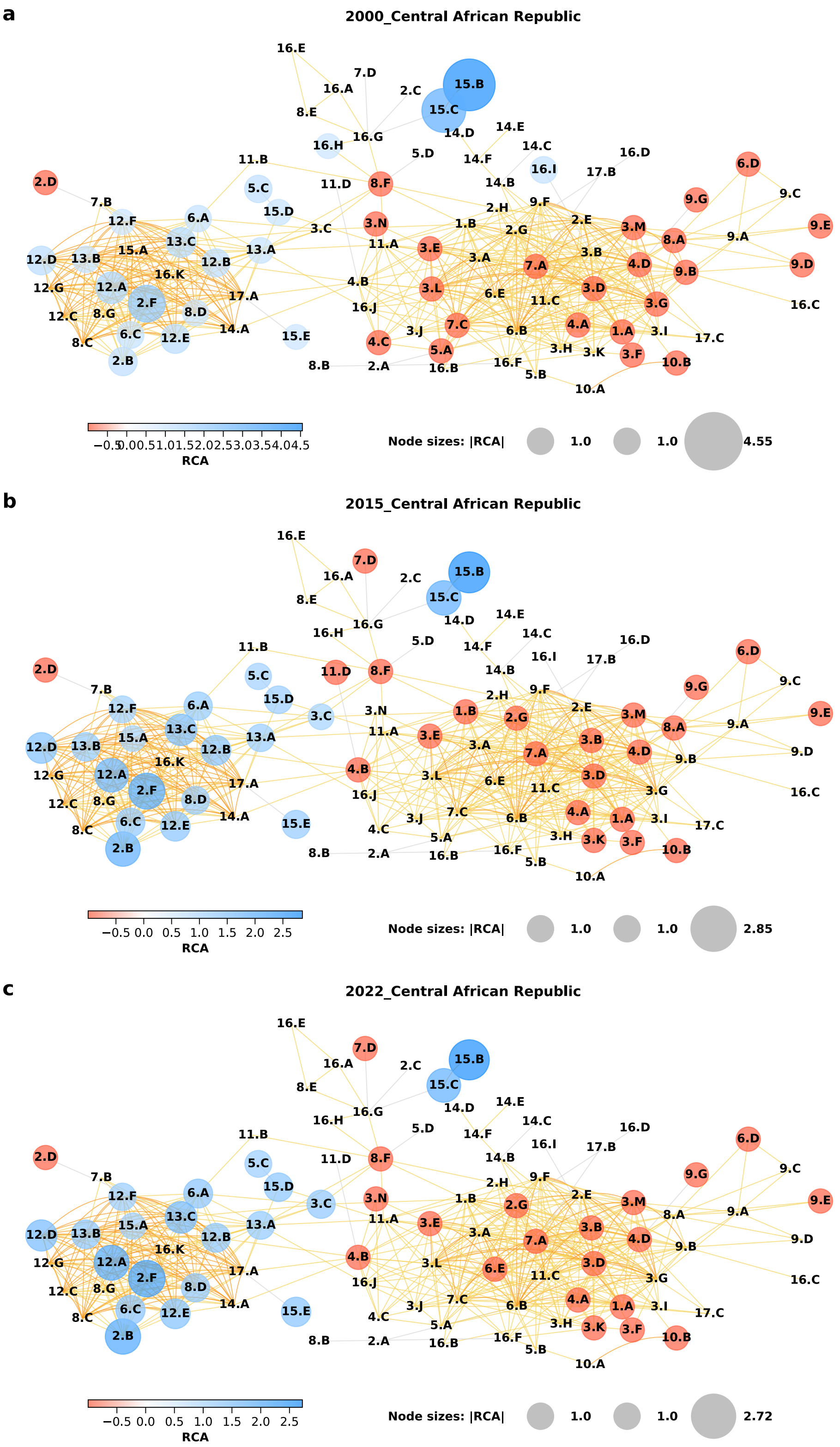

Supplementary Figure 171 | The SDG space of Central African Republic. Panels **a, b, c,** The SDG space in 2000, 2015, and 2022. The nodes in blue and orange represent the top 20 and bottom 20 SDG indicators in revealed comparative advantage (RCA) values, respectively. The node size represents the absolute value of RCA. From Supplementary Figure 12 to 177, countries are ranked by GDP/capita (current US$, 2022).

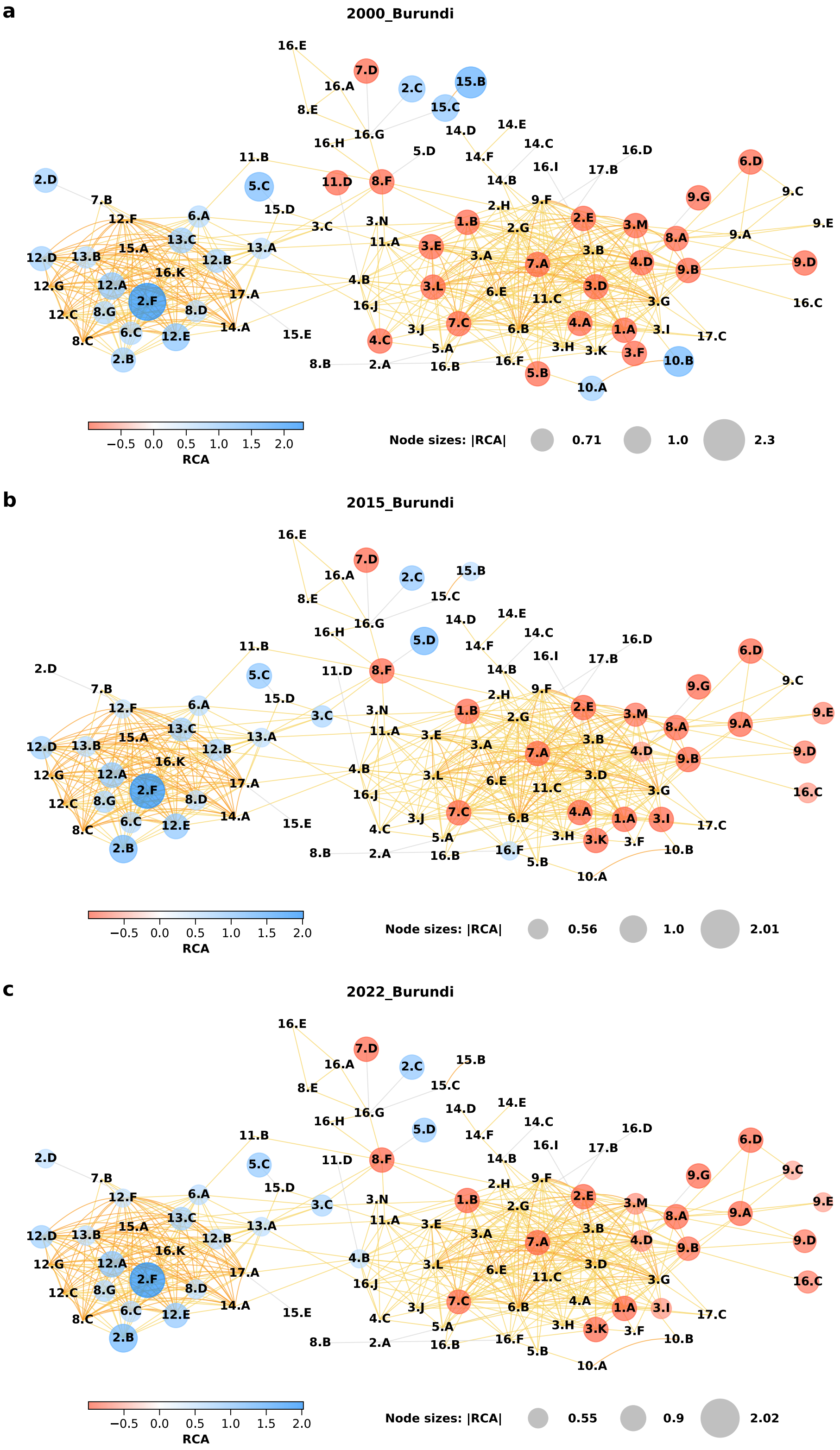

Supplementary Figure 172 | The SDG space of Burundi. Panels **a, b, c,** The SDG space in 2000, 2015, and 2022. The nodes in blue and orange represent the top 20 and bottom 20 SDG indicators in revealed comparative advantage (RCA) values, respectively. The node size represents the absolute value of RCA. From Supplementary Figure 12 to 177, countries are ranked by GDP/capita (current US$, 2022).

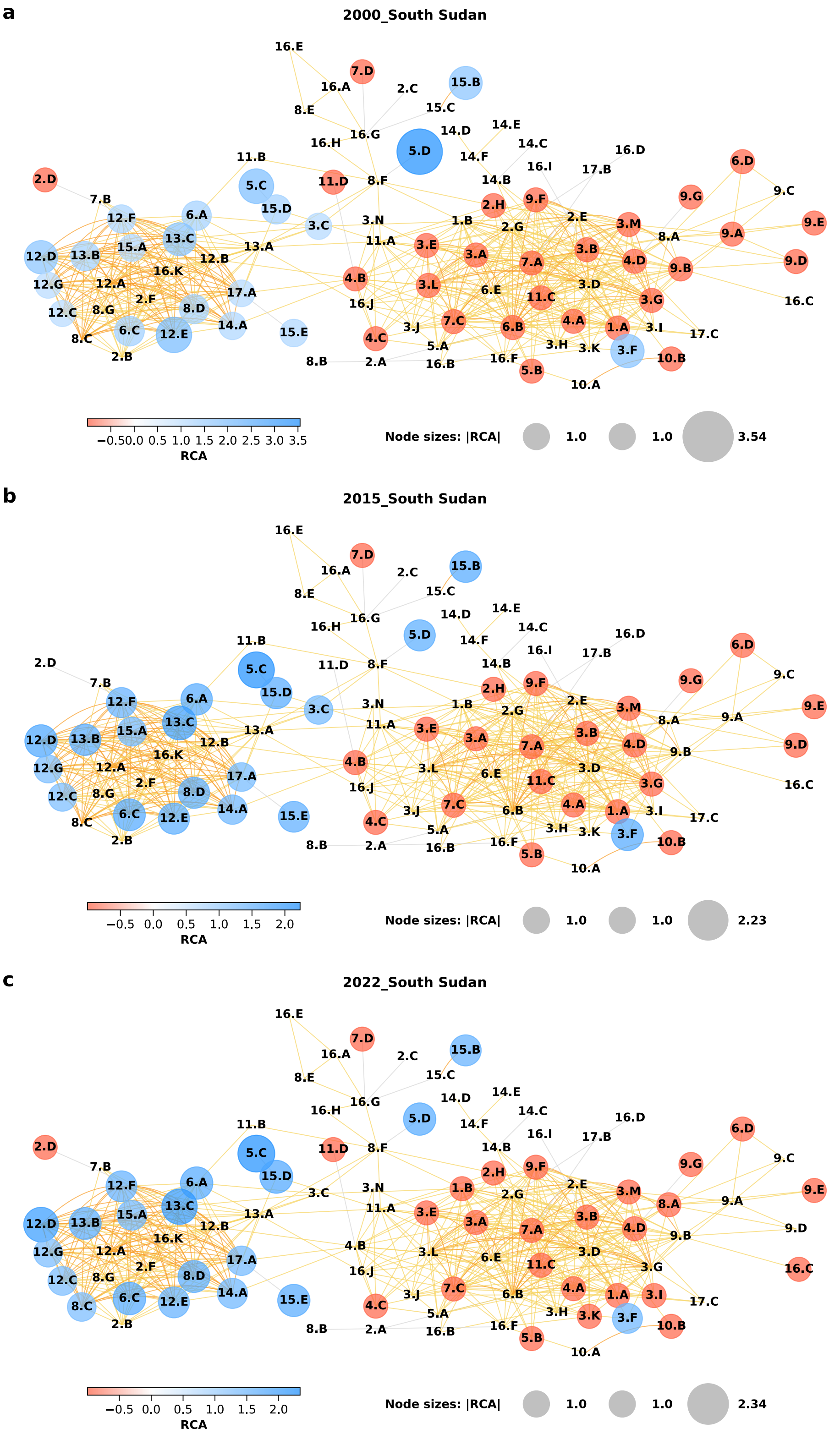

Supplementary Figure 173 | The SDG space of South Sudan. Panels **a, b, c,** The SDG space in 2000, 2015, and 2022. The nodes in blue and orange represent the top 20 and bottom 20 SDG indicators in revealed comparative advantage (RCA) values, respectively. The node size represents the absolute value of RCA. From Supplementary Figure 12 to 177, countries are ranked by GDP/capita (current US$, 2022).

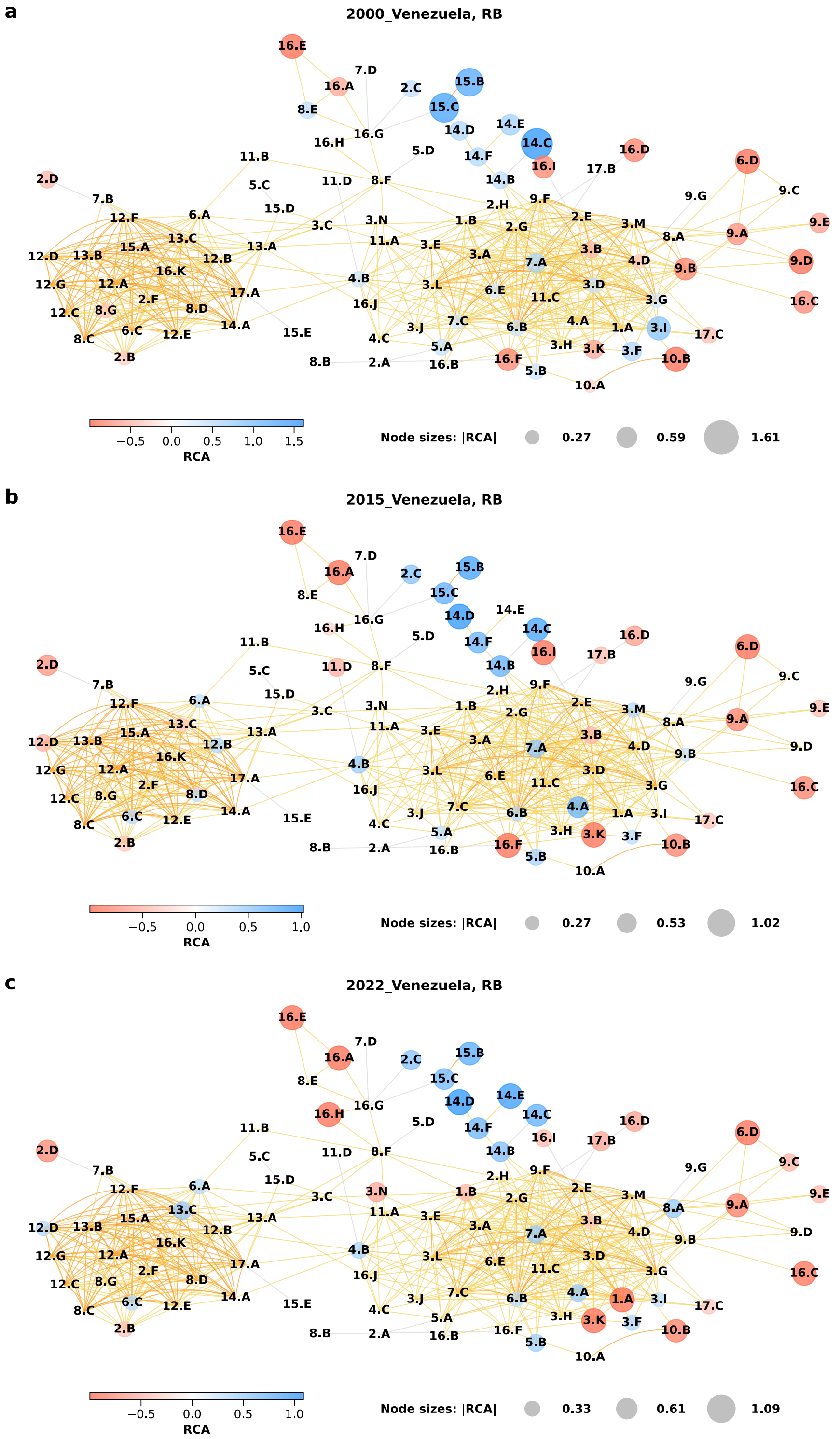

Supplementary Figure 174 | The SDG space of Venezuela, RB. Panels **a, b, c,** The SDG space in 2000, 2015, and 2022. The nodes in blue and orange represent the top 20 and bottom 20 SDG indicators in revealed comparative advantage (RCA) values, respectively. The node size represents the absolute value of RCA. From Supplementary Figure 12 to 177, countries are ranked by GDP/capita (current US$, 2022). The GDP per capita data for Venezuela, RB was unavailable, so its figure was placed at the back of the Supporting Information.

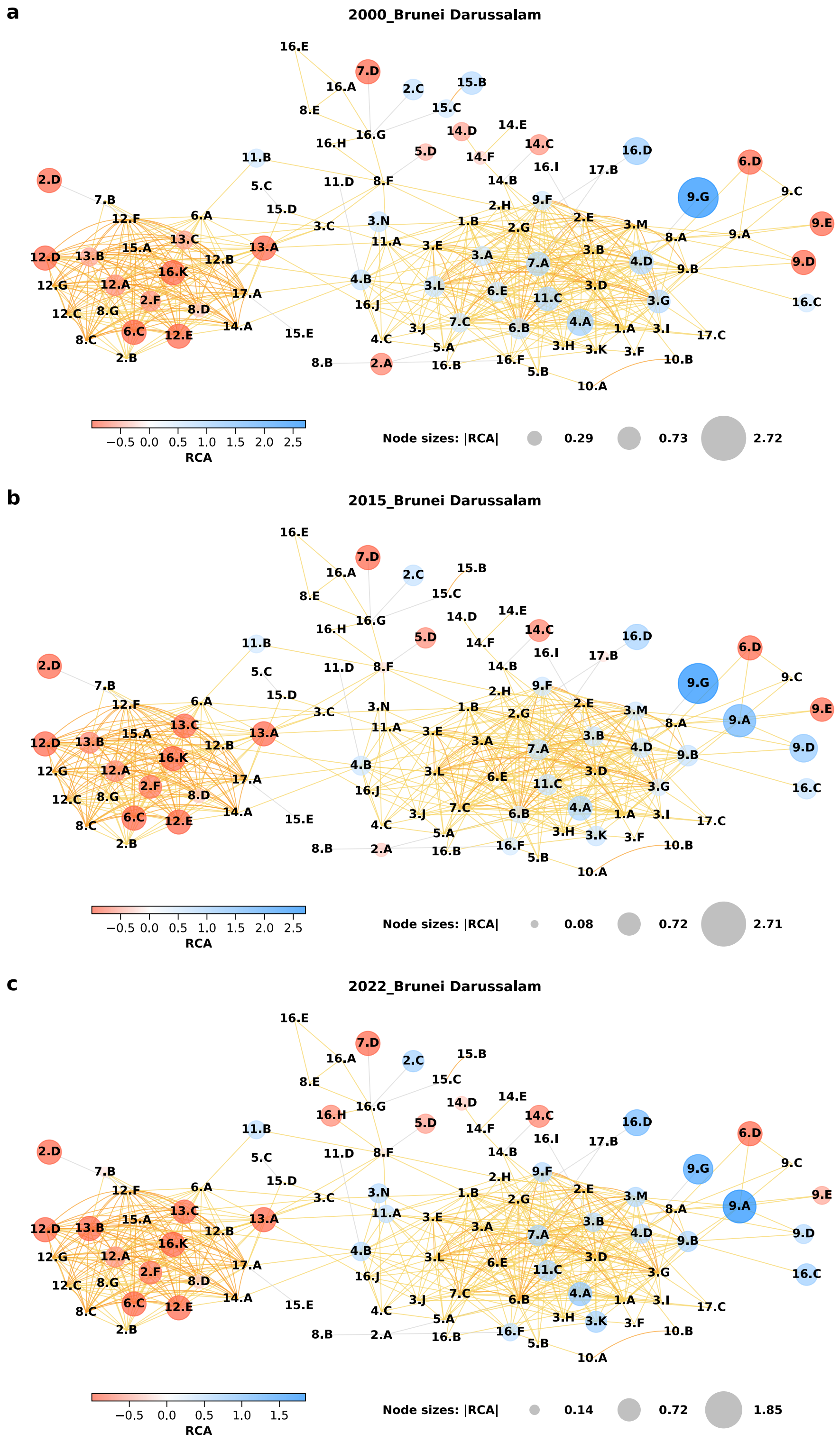

Supplementary Figure 174 | The SDG space of Brunei Darussalam. Panels **a, b, c,** The SDG space in 2000, 2015, and 2022. The nodes in blue and orange represent the top 20 and bottom 20 SDG indicators in revealed comparative advantage (RCA) values, respectively. The node size represents the absolute value of RCA. From Supplementary Figure 12 to 177, countries are ranked by GDP/capita (current US$, 2022). The GDP per capita data for Brunei Darussalam was unavailable, so its figure was placed at the back of the Supporting Information.

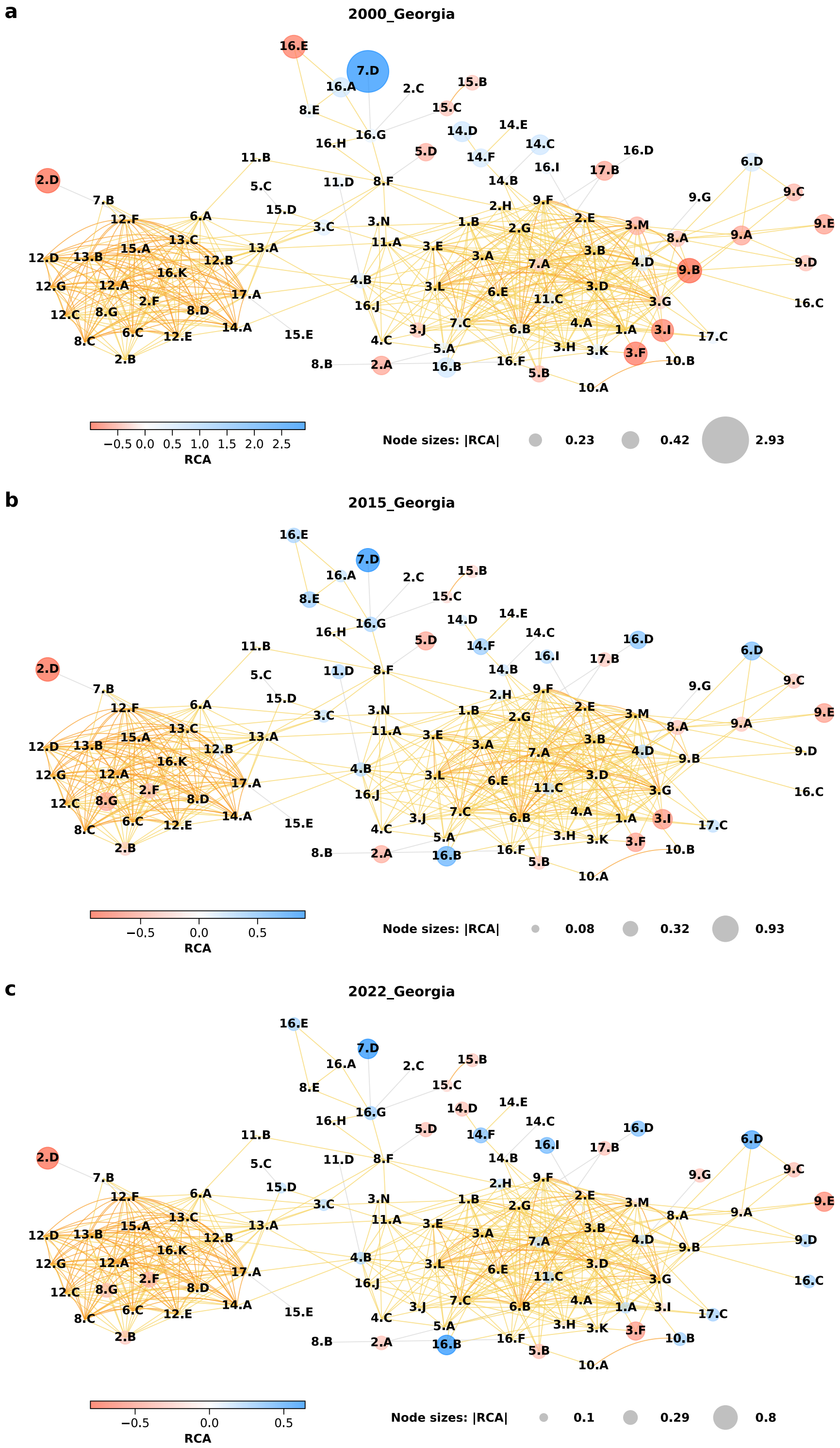

Supplementary Figure 175 | The SDG space of Georgia. Panels **a, b, c,** The SDG space in 2000, 2015, and 2022. The nodes in blue and orange represent the top 20 and bottom 20 SDG indicators in revealed comparative advantage (RCA) values, respectively. The node size represents the absolute value of RCA. From Supplementary Figure 12 to 177, countries are ranked by GDP/capita (current US$, 2022). The GDP per capita data for Georgia was unavailable, so its figure was placed at the back of the Supporting Information.

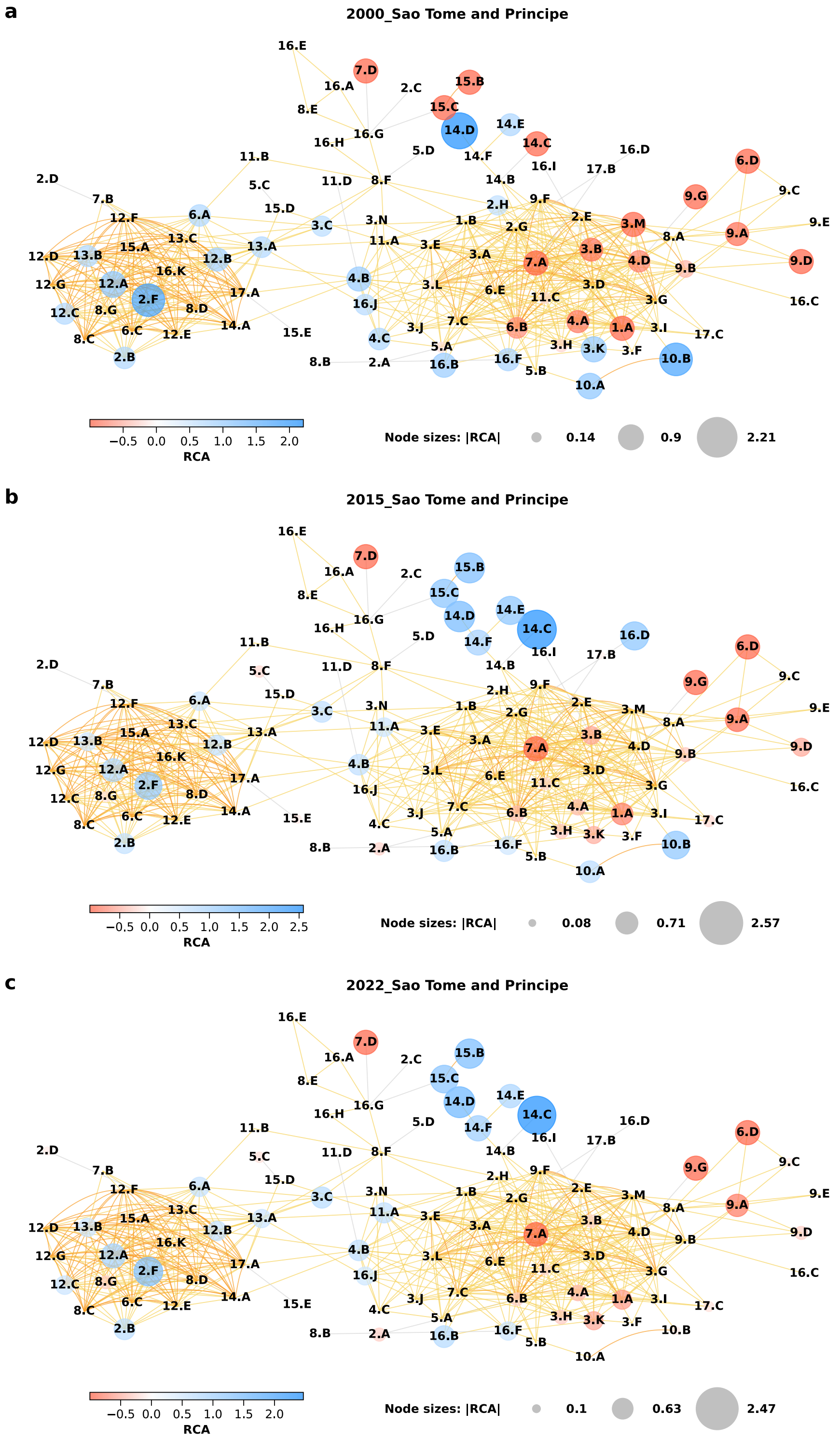

Supplementary Figure 176 | The SDG space of Sao Tome and Principe. Panels **a, b, c,** The SDG space in 2000, 2015, and 2022. The nodes in blue and orange represent the top 20 and bottom 20 SDG indicators in revealed comparative advantage (RCA) values, respectively. The node size represents the absolute value of RCA. From Supplementary Figure 12 to 177, countries are ranked by GDP/capita (current US$, 2022). The GDP per capita data for Sao Tome and Principe was unavailable, so its figure was placed at the back of the Supporting Information.

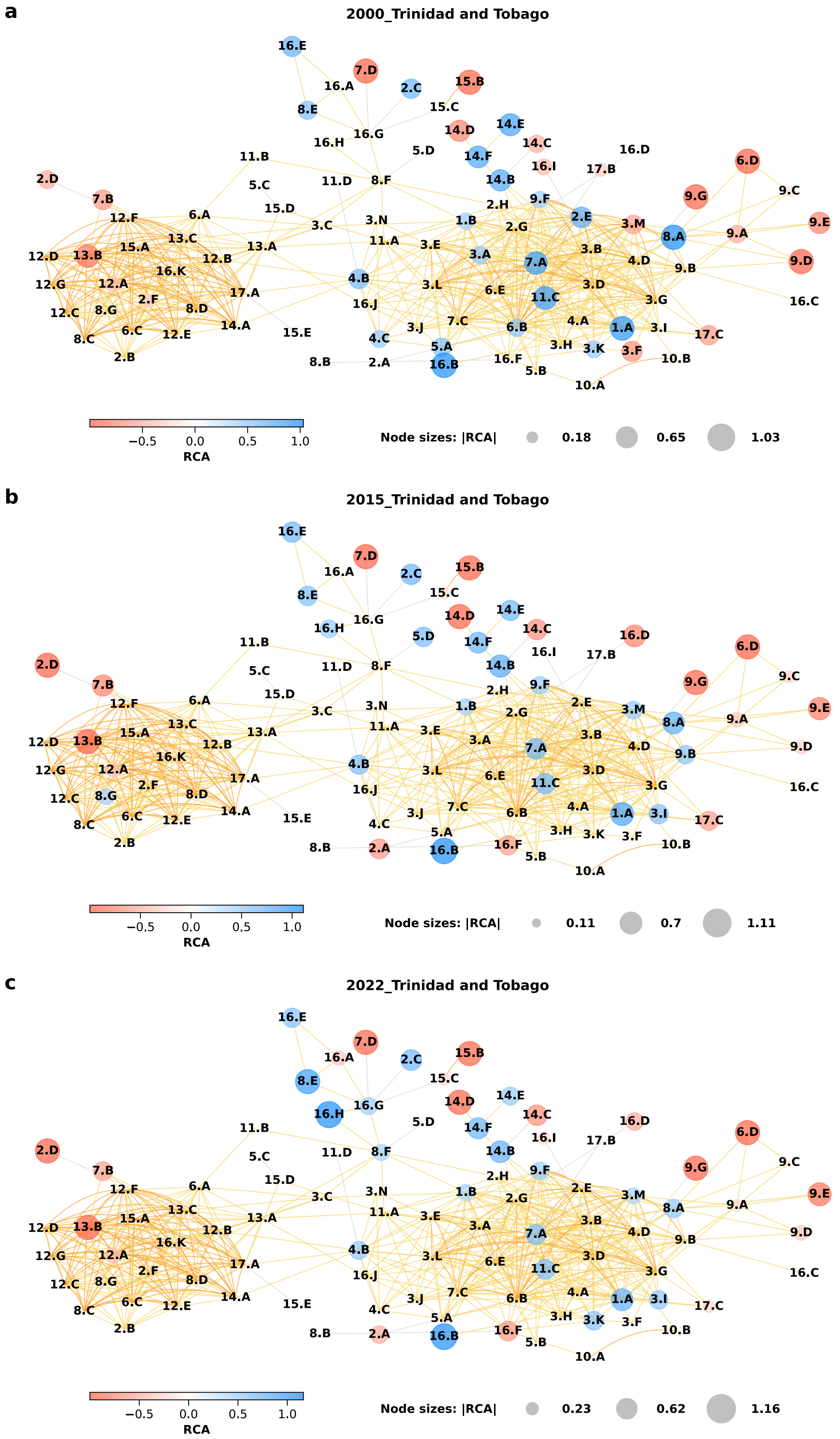

Supplementary Figure 177 | The SDG space of Trinidad and Tobago. Panels **a, b, c,** The SDG space in 2000, 2015, and 2022. The nodes in blue and orange represent the top 20 and bottom 20 SDG indicators in revealed comparative advantage (RCA) values, respectively. The node size represents the absolute value of RCA. From Supplementary Figure 12 to 177, countries are ranked by GDP/capita (current US$, 2022). The GDP per capita data for Trinidad and Tobago was unavailable, so its figure was placed at the back of the Supporting Information.
